# Metagenomic insight into drought-induced changes in the Egyptian wheat rhizosphere microbiome

**DOI:** 10.1007/s11274-025-04518-0

**Published:** 2025-08-12

**Authors:** Haytham M. Abd El-Halim, Mohamed El-Hadidi, Nourhan Fouad, Ranin R. Hamed, Islam A. Megid, Manar H. Taha, Khaled H. Radwan

**Affiliations:** 1 Agricultural Genetic Engineering Research Institute (AGERI), Agricultural Research Center (ARC), Giza, 12619 Egypt; 2 School of Biotechnology, Badr University in Cairo (BUC), Cairo, 11829 Egypt; 3 Department of Cancer and Genomic Sciences, School of Medical Sciences, College of Medicine and Health, University of Birmingham Dubai, Dubai, UAE; 4 Center for Informatics Sciences (CIS), School of Information Technology and Computer Science (ITCS), Nile University, Giza, Egypt; 5 International Center of Agricultural Research in Dry Areas (ICARDA), Giza, 11742 Egypt; 6 Department of Biotechnology, Faculty of Science, Cairo University, Giza, 12613 Egypt; 7 National Biotechnology Network of Expertise (NBNE), Academy of Scientific Research and Technology (ASRT), Cairo, 11516 Egypt

**Keywords:** Wheat rhizosphere, Drought stress, Microbiome, 16S rRNA gene sequencing, Core microbiota

## Abstract

Wheat is one of the most important cereal crops and an important source of food for billions of people worldwide. However, drought stress can pose a real threat to its productivity and lead to significant yield losses, especially in Egypt. The rhizospheric microbiome of wheat can play an important role in drought stress and help wheat to respond to this abiotic stress. Understanding this microbiome is therefore also important to improve drought stress resilience and productivity. In this study, a metagenomic analysis was performed to investigate how the composition and diversity of microbial communities associated with the wheat rhizosphere change under drought. Taxonomic and phylogenetic analyses revealed a shift in microbial abundance, with *Actinobacteria*, *Bacteroidetes*, *Proteobacteria* and *Verrucomicrobia* being the four most abundant phyla of the ethnic microbiota. Remarkably, other classes, including *Alphaproteobacteria* and *Cytophagia*, were significantly enriched under drought, which could be a promising enhancement of plant stress altruism. Differential abundance analysis showed that the control samples had higher abundance of microbial taxa such as OD1, WS2, Chlorobi, ABY1 and SHA-109 compared to the drought-treated genotypes. Functional prediction analysis using PICRUSt showed that an uncharacterized ATP-binding protein within the AAA + superfamily is overrepresented under drought conditions. This suggests that these genes may play a role in stress adaptation, possibly via energy-dependent regulation of cellular processes involved in plant survival. Our results expand our understanding of the complexity of responses of the wheat rhizosphere microbiome to drought and have practical implications for the development of microbial target combinations to improve wheat tolerance and productivity in the context of climate change challenges.

**Supplementary Information:**

The online version contains supplementary material available at 10.1007/s11274-025-04518-0.

## Introduction

Wheat (*Triticum* spp.) is one of the world’s most important crops and a staple food for thousands of millions of people. For many nations, it is a vital source of food, income and revenue. Considering its importance, it is essential to study the determinants of wheat yield and quality, which in turn are threatened by increasing abiotic stress factors such as drought, which pose a major challenge to agricultural productivity worldwide (Gu et al. 2024; Kang et al. 2022).

Drought is one of the main environmental stress factors that negatively affect wheat growth, leading to a decrease in biomass, grain yield and nutritional quality (Gargallo-Garriga et al. 2018; Hone et al. 2020). In Egypt, where climate change is exacerbating water scarcity and the occurrence of extreme drought events, drought is becoming more pronounced and directly threatens wheat productivity and thus national food security (Parejko 2024).

Researchers have explored the potential of harnessing the natural microbial communities associated with plants to improve their resilience to environmental stressors. The rhizosphere microbiome, consisting of a diverse array of microorganisms in the soil surrounding plant roots, plays an important role in plant health and stress tolerance (Hu et al. 2020). Certain microbial species can improve nutrient availability, stimulate plant growth, and activate stress-responsive metabolic pathways, which ultimately improve drought tolerance in wheat (Ali et al. 2022).

Recent metagenomic studies have revealed the complexity of interdependencies in the rhizosphere microbiome under stress conditions. In particular, the use of metagenomics enables the study of community composition and functional potential within microbial assemblages, providing previously inaccessible insights into microbial diversity and evolutionary strategies to adapt to drought conditions (Hu et al. 2020; Xu et al. 2021). For example, a few crops have shown that the composition of the microbiome changes markedly in response to drought stress, with some taxa enriched and others depleted, depending on the severity and duration of drought (Fan et al. 2023; Naylor et al. 2017).

This change in microbial composition is not only a reliable indicator of the stress to which the plants are exposed but also reveals a sophisticated system of plant adaptation that may improve the drought tolerance of wheat (Hone et al. 2021). In addition, the remnants of past droughts may continue to alter microbial community structure in ways that influence subsequent plant growth and functional responses to threats (Jochum et al. 2019; Karasov et al. 2022).

In addition to identifying the microorganisms that reside in the rhizosphere during drought, the metagenomic approach enables the differentiation of functional traits that may benefit the host plant (Gargallo-Garriga et al. 2018; Hone et al. 2021). The characterization of functional genes in microbial communities enables the identification of metabolic pathways that contribute to plant stress alleviation, nutrient cycling and pathogen suppression. *Actinobacteria*, for example, have been shown to be important for beneficial effects on plants, as these bacteria can produce metabolites that modulate stress and are also able to utilize organic substrates excreted by the plant for more drought (Naylor et al. 2017).

While it is known that drought can affect the rhizosphere microbiome, exactly how drought affects the microbiome and what this means for wheat remains to be elucidated. Recent studies have begun to shed light on the variability of microbial responses to drought. They suggest that the results may be mediated by plant genotype (Naylor et al. 2017), the soil type (Breitkreuz et al. 2021) or the ecological interactions between the microbes present (Gargallo-Garriga et al. 2018).

Since the considerable differences in drought impacts and responses are due to differences in the growth response of wheat, metagenome-based studies should be conducted to determine whether different microbiomes are present and whether specific wheat germplasm differs in their response to drought stress and subsequent repair of damage after rewatering. These results reveal microbial consortia or taxa associated with drought resistance and provide a basis for biotechnological developments such as novel microbial inoculants or improved agricultural manipulations (Liu et al. 2024).

Although research interest has focused on plant-microbiome interactions under abiotic stress, there are still significant gaps in understanding the responses of specific microbial communities in the rhizosphere to drought in different wheat genotypes, particularly in arid and semi-arid regions. Most of the research to date has focused on temperate climates, so there is little work on Mediterranean and North African wheat systems (Gu et al. 2024; Talai et al. 2023). Egyptian durum wheat, for example, is a genetic reservoir with ancient wild relatives and modern genotypes bred for the next release to survive under the extreme conditions of the same climate (Mursalova et al. 2015).

Such diverse soil microbiomes from different genotypes may contain different beneficial microbes and functions that are important for drought adaptation (Aktas 2022; Xie et al. 2021). Nevertheless, metagenomic studies are not common in the Egyptian context, and studies linking taxonomic shifts to functional responses are even rarer (Lian et al. 2023).

Insight into these interactions is essential for microbiome-based approaches to improve wheat resilience in water-scarce environments, where water availability has a direct impact on plant physiology, nutrient uptake and yield capacity (Jochum et al. 2019; Xiang et al. 2024). In addition, previous studies have shown that drought stress strongly alters the structure of microbial communities in the rhizosphere. This highlights the need to analyze the profiles of these microbial communities in different areas where they have been relatively understudied (Abd-Elhalim et al. 2024; Vries et al. 2020).

To achieve this, the current study was conducted to characterize the composition of the rhizosphere microbiome of eight Egyptian wheat genotypes, consisting of wild and cultivated lines, in response to drought and control conditions, to identify taxonomic and functional shifts in microbial communities using 16 S rRNA-based metagenomic sequencing, and to extract the major and differentially abundant taxa that might contribute to drought response through their function. By incorporating diversity metrics, correlation analysis and predictive functional profiling, this study should provide insights into the mechanistic responses of the microbiome to drought stress and identify microbial candidates that can potentially improve the resilience of wheat in Egypt and other agroecosystems.

## Materials and methods

### Material

#### Collection of rhizosphere samples

Rhizospheric soil samples (Table 1) were collected in January 2022 from eight different Egyptian wheat genotypes; representing both wild and cultivated genotypes. The wild genotypes are represented by *Triticum dicoccum* and *Triticum monococcum*, while the cultivated genotypes were *Giza 168*, *Gemiza 9*, *Gemiza 11*, *Masr 1*, *Sids 13*, and *Shandweel 1*. These cultivated genotypes represent the most promising wheat genotypes developed by the Agricultural Research Center (ARC), Giza, Egypt. The trial was conducted at the experimental field station of the Agricultural Genetic Engineering Research Institute (AGERI), ARC, in Giza, Egypt, with coordinates 30°01’25” N, 31°12’11” E.

Five specimens of each wheat variety were collected aseptically and brought to the laboratory in sterile bags. The root samples were taken from a depth of 30 cm, and the adhering soil was manually shaken or loosened from the roots. The roots were cut with sterilized scissors and placed in tubes containing 10 mL of sterile 1% NaCl solution to dissolve the microbes associated with the rhizosphere by shaking. The resulting suspension was vortexed to further release rhizosphere bacteria. Plant debris and roots were then removed, and the solution was filtered through a sterile 100 μm cell strainer.

The filtrate was centrifuged at 3000×g for five minutes, the supernatant was discarded, and the bacterial pellet was resuspended in 1% NaCl solution (McPherson et al. 2018). The samples were then stored at − 20 °C prior to DNA extraction. Genomic DNA was extracted using the PowerSoil DNA Isolation Kit (Qiagen, Cat. No. 47014) according to the manufacturer’s recommended protocol (Fouad et al. 2025).

**Table 1:** Library names, corresponding Egyptian wheat genotypes, treatment conditions (control or drought), and their respective accession numbers

No.	Library Name	Wheat Genotype Name	Control/drought	Accession Number
1	MR01	*Triticum dicoccum*	Control	SRR27488919
2	MR02	*Triticum dicoccum*	Control	SRR27488918
3	MR03	*Triticum dicoccum*	Control	SRR27488907
4	MR04	*Triticum dicoccum*	Drought	SRR27488897
5	MR05	*Triticum dicoccum*	Drought	SRR27488886
6	MR06	*Triticum dicoccum*	Drought	SRR27488877
7	MR07	Giza 168	Control	SRR27488876
8	MR08	Giza 168	Control	SRR27488875
9	MR09	Giza 168	Control	SRR27488874
10	MR10	Giza 168	Drought	SRR27488873
11	MR11	Giza 168	Drought	SRR27488917
12	MR12	Giza 168	Drought	SRR27488916
13	MR13	Gemiza 9	Control	SRR27488915
14	MR14	Gemiza 9	Control	SRR27488914
15	MR15	Gemiza 9	Control	SRR27488913
16	MR16	Gemiza 9	Drought	SRR27488912
17	MR17	Gemiza 9	Drought	SRR27488911
18	MR18	Gemiza 9	Drought	SRR27488910
19	MR19	Gemiza 11	Control	SRR27488909
20	MR20	Gemiza 11	Control	SRR27488908
21	MR21	Gemiza 11	Control	SRR27488906
22	MR22	Gemiza 11	Drought	SRR27488905
23	MR23	Gemiza 11	Drought	SRR27488904
24	MR24	Gemiza 11	Drought	SRR27488903
25	MR25	Masr 1	Control	SRR27488902
26	MR26	Masr 1	Control	SRR27488901
27	MR28	Masr 1	Drought	SRR27488900
28	MR29	Masr 1	Drought	SRR27488899
29	MR30	Masr 1	Drought	SRR27488898
30	MR31	*Triticum monococcum*	Control	SRR27488896
31	MR32	*Triticum monococcum*	Control	SRR27488895
32	MR33	*Triticum monococcum*	Control	SRR27488894
33	MR34	*Triticum monococcum*	Drought	SRR27488893
34	MR35	*Triticum monococcum*	Drought	SRR27488892
35	MR36	*Triticum monococcum*	Drought	SRR27488891
36	MR37	Sids 13	Control	SRR27488890
37	MR38	Sids 13	Control	SRR27488889
38	MR39	Sids 13	Control	SRR27488888
39	MR40	Sids 13	Drought	SRR27488887
40	MR41	Sids 13	Drought	SRR27488885
41	MR42	Sids 13	Drought	SRR27488884
42	MR43	Shandweel 1	Control	SRR27488883
43	MR44	Shandweel 1	Control	SRR27488882
44	MR45	Shandweel 1	Control	SRR27488881

### Methods

#### Metagenomic amplicon sequencing

Targeted amplicon sequencing of the 16 S rRNA gene was performed to characterize the bacterial communities in the rhizosphere of wheat. The hypervariable V3–V4 regions of the 16 S rRNA gene were amplified with region-specific primers: 341 F (5’-CTACGGGGNGGGCWGCAG-3’) and 806-R (5’-GGACTACNNGGGTATCTAAT-3’). Sequencing was performed on Illumina’s NovaSeq platform (Illumina, San Diego, CA, USA) according to the manufacturer’s protocols, which enables deep sequencing of amplified fragments at high throughput.

#### Sequence analysis

Data analyses were performed using the bioinformatics software package QIIME2 (www.qiime2.org) via the q2cli interface. Forward and reverse sequences were merged, denoised, dereplicated and filtered for chimaeras using the DADA2 plugin (q2-dada2). Amplicon sequence variants (ASVs) were aligned using MAFFT (via q2-alignment) and a phylogeny was generated using FastTree (via q2-phylogeny). Based on a preliminary analysis, a subsampling depth of 2000 was chosen.

Taxonomic classification of ASVs was performed using the 99% Greengenes 13.8 reference database (q2-feature-classifier), removing unwanted taxa such as those containing mitochondria, archaea, eukaryotes or chloroplasts in their taxonomic annotation. For visualization, taxa bar plots and collapsed tables were created and the data for each taxa level was summarized in a table. For additional analysis, R programming was used with the output files generated by QIIME2.

The package QIIME 2R enabled the import of QIIME artifacts into an R session. The package phyloseq was used to import, store, analyze, and visually display complex phylogenetic sequencing data organized into Operational Taxonomic Units (OTUs), especially when associated specimen data, a phylogenetic tree, and taxonomic assignments of the OTUs were available. The package vegan was used for diversity analysis, while the packages ggplot2, pheatmap and RColorBrewer were used to create bar charts and heatmaps for visualization.

Visual exploration of the taxonomic composition of the studied rhizosphere community was performed by comparing abundances. The taxonomic composition was considered at phylum, class and family levels, categorized according to an experimental factor that grouped the experiment into four categorical aspects: rhizosphere soil samples of the cultivated genotypes under controlled conditions, rhizosphere soil samples of the drought- treated cultivated genotypes, rhizosphere soil samples of the wild genotypes under controlled conditions and rhizosphere soil samples of the drought- treated wild genotypes.

Taxa were categorized into the categorical group based on the total sum of their counts, with the top taxa (*n* = 12) displayed to show the percentage abundance. Alpha diversity analysis was performed using the Microbiome Analyst tool (available at: http://www.microbiomeanalyst.ca) (Dhariwal et al. 2017). Observed alpha diversity was used to quantify species richness; the richness (actual number) of unique taxa observed was calculated by the index Observed. In addition, evenness (number of abundances) was calculated using the Shannon, Simpson and Fisher’s indices to represent the true diversity of the community (Iannucci et al. 2021).

Beta diversity analysis was performed using the phyloseq package in R to compare the composition of the microbial community in the different samples. Bray-Curtis distance was used to calculate dissimilarities based on the presence, absence, and abundance of taxa. Principal coordinate analysis (PCoA) served as the ordination method, using the Bray-Curtis index as the distance metric. Statistical differences between groups were assessed using PERMANOVA to detect significant variations in microbial composition.

#### Differential abundance analysis

The taxonomic counting matrix at the phylum and class level, together with the corresponding metadata file, was imported into the iDEP (Integrated Differential Expression and Pathway Analysis) software to perform differential frequency analysis using limma-voom (Ge et al. 2018). This analysis aims to identify highly significant taxa that showed a distinct presence and contributed significantly to the observed differences between the control and drought groups.

These heat maps provided an intuitive and informative representation of the taxonomic composition of the samples and enabled the identification of patterns and relationships between taxa. The resulting heatmap, created using ClustVis software (Metsalu and Vilo 2015), was subjected to further analysis and interpretation to identify meaningful relationships between the samples and patterns of taxonomic composition. This comprehensive analysis enabled a deeper understanding of taxonomic dynamics and their relationships to control and drought conditions.

#### Core Microbiome

In our study, we performed a core analysis of the microbiome to identify taxa or traits that are stable in the composition of the microbial community. This approach helps to understand the basic components that are stable within the microbiota. We determined two central parameters with the core microbiome analysis.

The first parameter, called sample prevalence, defines the minimum proportion or percentage of samples in which a taxa or trait must be present to be considered part of the core microbiome.

A value of 20% was set as the threshold for sample prevalence. The second parameter, relative abundance, defines the proportion of the occurrence of a taxa or trait in the samples that qualifies it as a core member. In our analysis, a relative abundance threshold of 0.01% was used. By using web-based Microbiome Analyst tool (available at: http://www.microbiomeanalyst.ca).

The analysis of the core microbiome was performed using the core function from the R package ‘microbiome’. This function enables efficient determination and visualization of core taxa or features.

#### Correlation analysis

A correlation analysis was performed to determine whether certain traits showed different patterns in the cultivated and wild genotypes under different conditions, i.e. drought and normal irrigation. Correlation was performed at the level of strain, class and family taxonomy. The Pearson r correlation method was used as a measurement distance to determine whether a linear relationship exists between two taxa. The MicrobiomeAnalyst tool was used for this purpose (available at: http://www.microbiomeanalyst.ca) (Dhariwal et al. 2017).

#### Machine learning-based functional profiling

The 16 S rRNA sequencing data were used to estimate the functional profiles of the microorganisms in the wheat rhizosphere by inferring their traits based on their genetic relatedness to fully sequenced and annotated organisms. This process was carried out using the Phylogenetic Investigation of Communities by Reconstruction of Unobserved States (PICRUSt13). Gene family frequencies were generated for each sample using the KEGG database (KO).

We then used the Shotgun Data Profiling (SDP) module in MicrobiomeAnalyst 2.0 to categorize the predicted metagenomes into broader functional categories and link them to metabolic pathways. We then used supervised Random Forests (RF), a classification algorithm consisting of unpruned decision trees created from training data. These trees were created using a subset of OTUs randomly selected from a bootstrap sample. The RF classifier was built by creating 5,000 classification trees and the prediction performance was evaluated using out-of-bag cross-validation and confusion matrices.

To identify the most predictive taxa for each microbiome assemblage, we determined the mean decrease in accuracy using the importance matrix. The Random Forest algorithm in MicrobiomeAnalyst 2.0 was used. This approach identified the OTUs that differed most from the different soils based on the decrease in model accuracy by leave-one-out cross-validation.

#### Results Microbiome profiling

To assess the diversity of the wheat rhizosphere microbiome, we performed alpha, and beta diversity analyzes. Through taxonomic classification, a total of 10,858 Operational Taxonomic Units (OTUs) were identified in the selected Egyptian wheat genotypes. After eliminating unwanted taxa, as previously mentioned, we classified and categorized the remaining 9,410 OTUs into seven taxonomic ranks, resulting in 356 OTUs [S1].

**Fig. 1:**
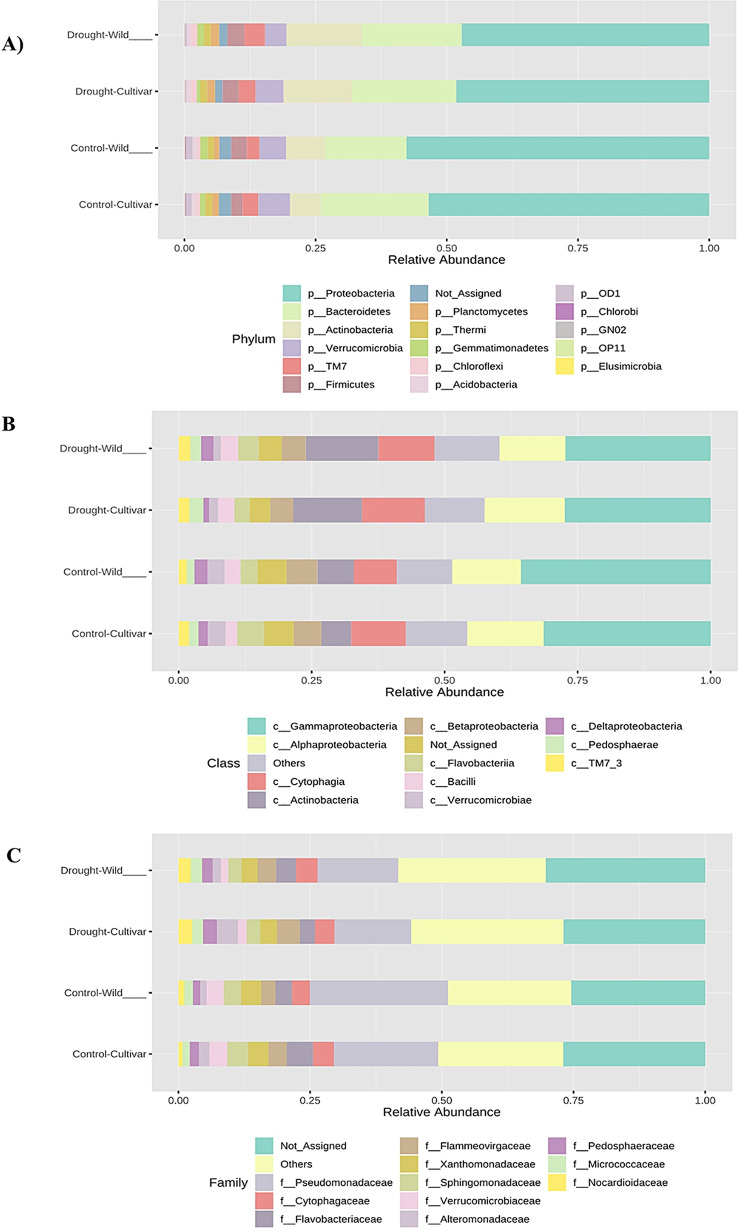
Drought- and control-induced taxonomic composition of the wheat rhizosphere microbiome. Relative abundance at the (**A**) phylum, (**B**) class and (**C**) family levels. Stacked bar graphs showing the taxonomic composition of the wheat rhizosphere microbiome under drought conditions (blue) and under control conditions (red). Only the 12 most abundant taxa are shown, with relative percentages. Common taxa accumulate in response to drought, including *Actinobacteria* and *Verrucomicrobia*, and the changes in microbial communities in response to drought are determined by changes in the abundance of these taxa

To determine the relative abundance of the different microbial groups associated with wheat plants, we performed a taxonomic analysis at the level of strains, classes and families. At the strain level, we observed four dominant and abundant phyla among the identified OTUs. The phyla *Proteobacteria* showed a prevalence of 48% under drought conditions for both wild and cultivated genotypes, with a slightly higher presence (58% and 53%) under control conditions for wild and cultivated genotypes, respectively (Fig. 1).

*Bacteroidetes* were represented at 19% and 20% in the wild and cultivated genotypes under drought conditions, respectively, and maintained similar proportions in both genotypes under control conditions (16 and 21, respectively). *Actinobacteria* were detected at 14% and 13% in the wild and cultivated genotypes under drought conditions and at 7% and 6% under control conditions, respectively. Finally, *Verrucomicrobia* was present at 4% and 5% in the wild and cultivated genotypes under drought conditions, respectively, and showed a higher percentage of 6% in both genotypes under control conditions.

The relative abundance of these phyla provided information on the composition of the microbial community associated with the wheat plants (Fig. 1A). A detailed class-level analysis assigned a substantial proportion of the Operational Taxonomic Units (OTUs) to specific classes. *Gammaproteobacteria* accounted for 27% of the taxa present under drought conditions for wild and cultivated genotypes, and 36% and 31% under control conditions, respectively.

*Alphaproteobacteria* were present in 12% and 15% of wild and cultivated wheat genotypes respectively, under drought conditions and 13% and 15% under control conditions. *Cytophagia* accounted for 11% and 12% under drought conditions and 8% and 10% under control conditions for the wild and cultivated genotypes, respectively. Bacilli were present at 4% and 3% under drought conditions and 3% and 2% under control conditions for the wild and cultivated genotypes, respectively. These results emphasize the prevalence of these specific classes under different conditions and shed light on their dynamics within the microbiota.

These classes accounted for a significant proportion of the taxonomic diversity within the wheat-associated microbial community (Fig. 1B). Microbial composition at the family level was analyzed under both control and drought conditions in the rhizosphere of wild and cultivated wheat genotypes. Pseudomonadaceae was the predominant family in these analyzes. Under controlled conditions, the rhizosphere of the wild wheat genotypes showed a higher prevalence of Pseudomonadaceae (26%) than that of the cultivated genotypes (20%). Under drought conditions, the prevalence was 15% for both genotypes (Fig. 1C).

**Fig. 2:**
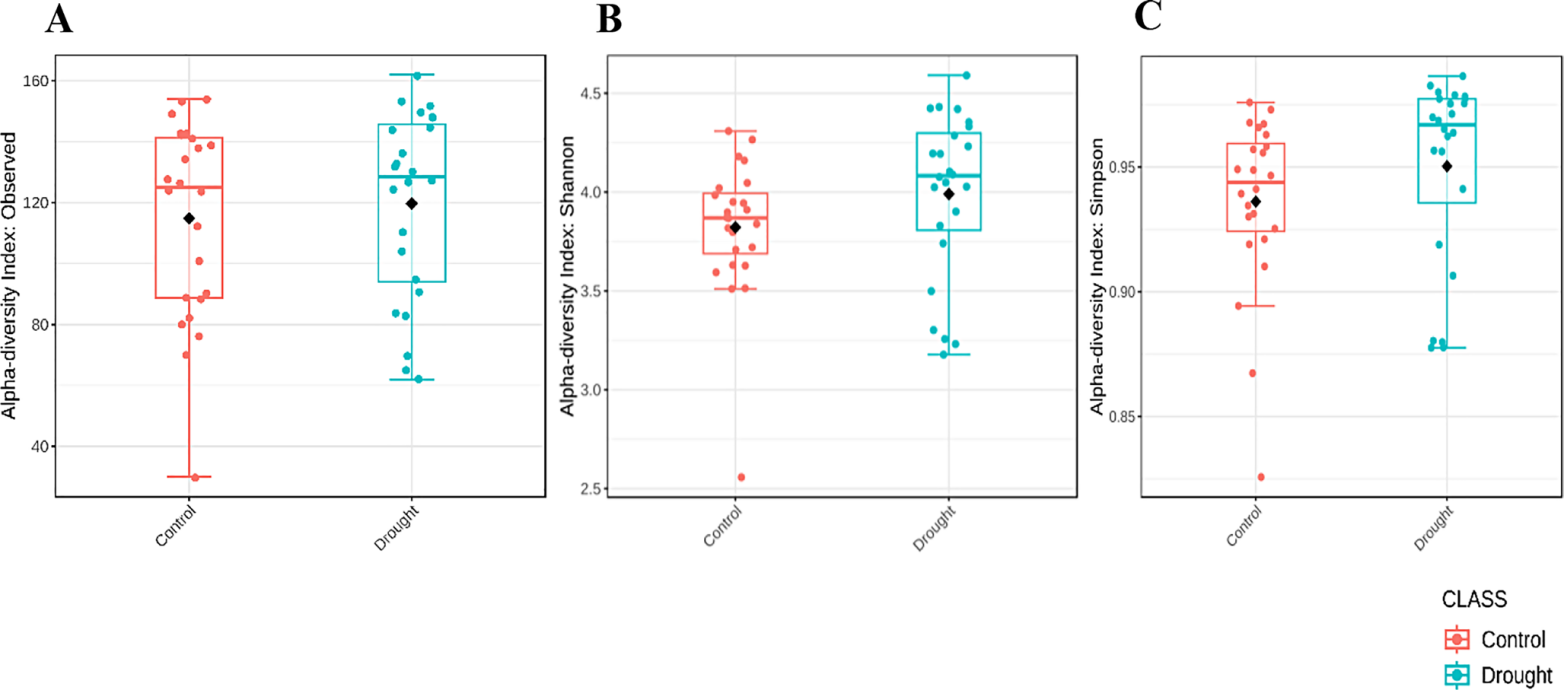
Alpha Diversity of the Microbiome in the Wheat Rhizosphere. Observed alpha diversity (species richness) using the Observed index (**A**), *Shannon* diversity index (**B**) and *Simpson diversity* index (**C**). Microbial diversity across experimental groups: control versus drought (*n* = 4) indicated by boxplots Shannon and Simpson indices of the respective microbial diversity show that microbial diversity was distinct between conditions (*P* < 0.005), suggesting shifts in community evenness and taxonomic richness. Typed statistical significances were determined using Kruskal-Wallis test

The analysis of alpha diversity at the species level, using the Kruskal-Wallis non-parametric statistical method, provided valuable insights into the diversity of the microbial community. The observed alpha diversity, which represents the actual species richness (Fig. 2A) showed no significant difference between the compared groups (p-value = 0.57059), indicating random variation in the observed number of species. However, the Simpson diversity index and the Shannon diversity index showed notable differences.

**Fig. 3:**
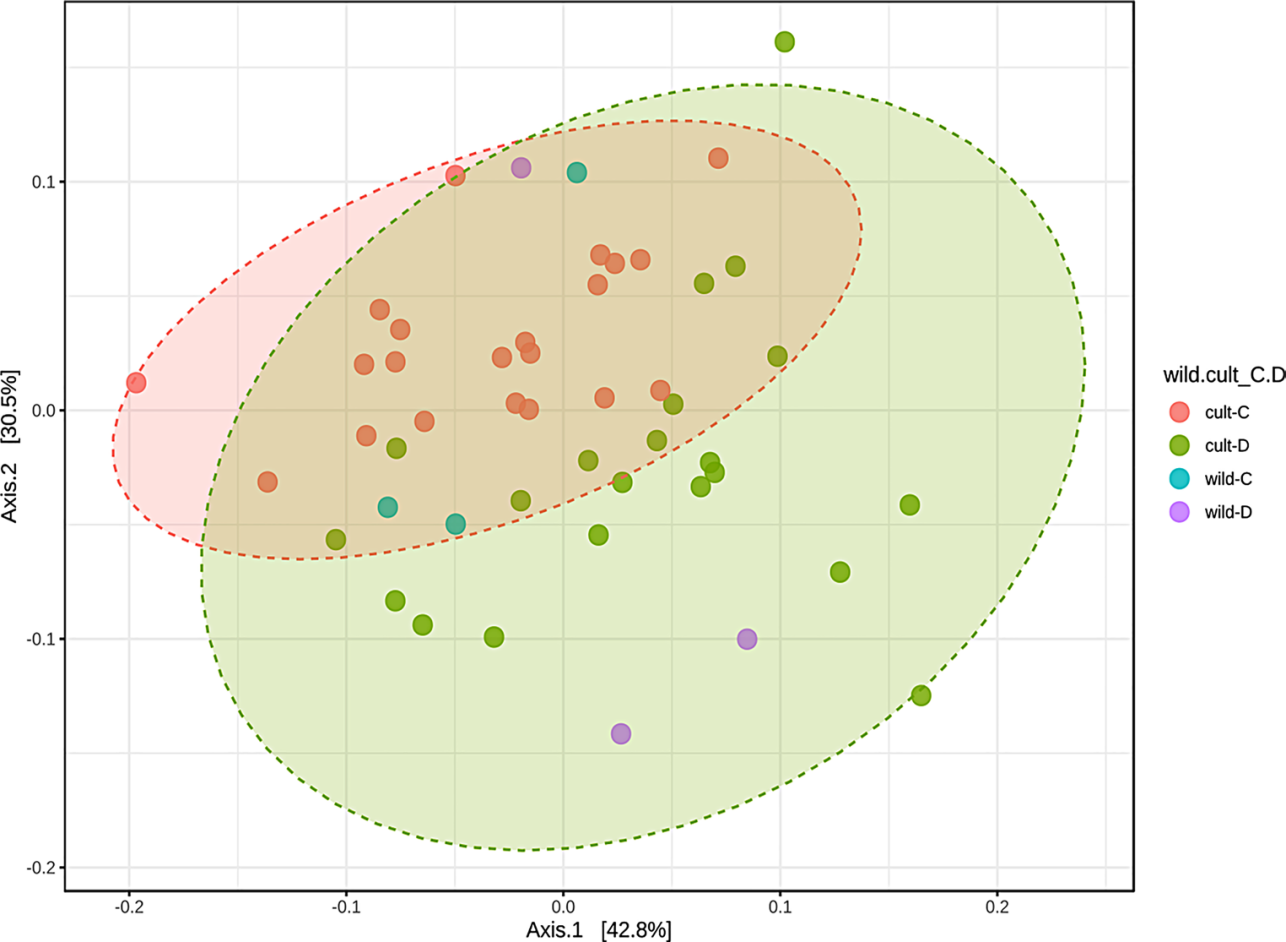
Principal coordinate analysis (PCoA) based on Bray-Curtis distance to visualize the beta diversity of the rhizosphere microbiome of wild and cultivated wheat genotypes under control and drought conditions. Distinct cluster patterns indicate shifts in the microbial community in response to genotype and environmental stress. The PERMANOVA test showed significant differences (*p* = 0.001 to 0.0285) between groups, with the highest significance observed in the cultivated genotypes. See Table 2 for detailed statistics

The Simpson index (p-value = 0.027147) (Fig. 2C) showed significant differences indicating variations in species diversity and evenness within the microbial communities. The Shannon index (p-value = 0.039408) also emphasized the different distribution and abundance of species in the groups (Fig. 2B). These results emphasize the importance of considering both abundance and evenness when assessing microbial diversity, as shown by the significant results of the Simpson and Shannon diversity indices at the species level.

Using Bray-Curtis dissimilarity analysis and weighted UniFrac analysis, we aimed to assess beta diversity and investigate the variation in microbial community composition within these genotypes. Bray-Curtis dissimilarity analysis showed similarities in microbial community composition between the control and drought-treated wheat genotypes Fig. (3). Principal component analysis revealed that PC1 explained 42.8% of the variance and PC2 explained 30.5% of the variance. These differences between the control and drought groups were not statistically significant, indicating a comparable microbial community composition.

However, the weighted UniFrac analysis revealed significant dissimilarities indicating differences in the phylogenetic composition of the microbial communities. These results provide valuable insights into the effects of drought on wheat-associated microbial communities and emphasize the role of phylogenetic relationships in shaping the observed dissimilarities (Fig. 3). The pairwise PERMANOVA test showed higher significance when comparing control and drought conditions among the cultivated genotypes, with a p-value of 0.001 (FDR-adjusted p = 0.006). This was followed by significant differences between other pairwise comparisons between wild and cultivated genotypes under drought (Table 2).”

**Table 2:** Statistical comparison of microbial community structures between drought and control genotypes for beta diversity analysis

Comparison	F-statistics	*R*-squared	*p*-value	FDR-adjusted *p*-value
Control-genotypes vs. Drought- treated genotypes	5.74899	0.144632	0.001	0.006
Drought-treated wild genotypes vs. Control-genotypes	5.15145	0.18973	0.003	0.009
Control-wild genotypes vs. Drought-treated genotypes	4.42872	0.167572	0.005	0.01
Control-wild genotypes vs. Drought-treated wild genotypes	3.81365	0.276078	0.019	0.0285

PERMANOVA F-statistics, R-squared values, and FDR-adjusted p-values indicate significant differences, particularly in the cultivated genotypes

#### Comparative assessment of microbial abundance and correlation analysis

The differential abundance analysis revealed different taxa whose abundance varied considerably between the two groups. On the other hand, correlation analysis investigated relationships and associations between taxa and different variables such as drought conditions and controlled environments.

#### Differential abundance analysis

**Fig. 4:**
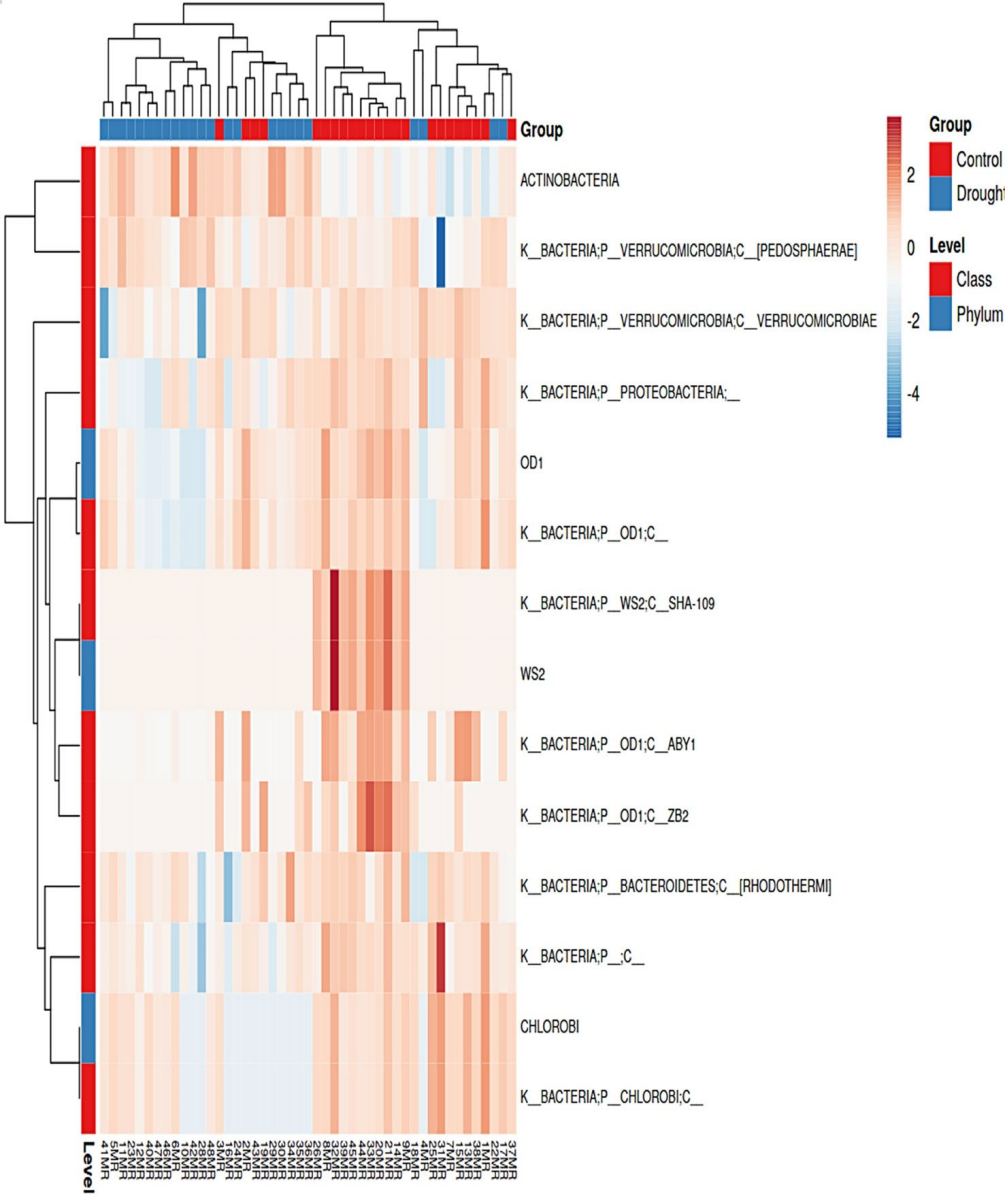
Hierarchical clustering heatmap of the differently abundant microbial taxa at phylum and class level. The color scale is based on relative abundance (from red for high abundance to blue for low abundance). Valuable taxa such as OD1, WS2, Chlorobi, ABY1 and SHA-109 show a clear difference between drought and control conditions

Differential abundance analysis was performed to identify microbial taxa that exhibited significant differences in abundance between different conditions or groups within the microbial community. For this analysis, the limma voom test implemented in the iDEP software was used. Heatmap clustering was then performed using ClustVis. At the phylum level, the analysis revealed several highly significant and abundant phyla that showed notable differences between the control and drought-treated groups (Fig. 4). The false discovery rate (FDR) threshold was set at less than 0.05%.

The identified phyla were OD1 (FDR = 3.41 × 10^− 6^), WS2 (FDR = 1.45 × 10^− 5^) and Chlorobi (FDR = 0.00549). In addition, the class-level analysis revealed highly significant and frequent classes that showed a significant difference between the control and drought-treated groups, also at an FDR threshold of less than 0.05%. These classes were ABY1 (FDR = 6.72 × 10^− 8^), *Actinobacteria* (FDR = 1.45 × 10^− 5^), SHA-109 (FDR = 1.45 × 10^− 5^), ZB2 (FDR = 9.05 × 10^− 5^), Rhodothermi (FDR = 0.00419), *Verrucomicrobiae* (FDR = 0.00419) and Pedosphaerae (FDR = 0.02398) (Fig. 4).

### Correlation analysis

In our correlation analysis, we investigated the relationship between the abundance of phyla and the different conditions in the rhizosphere of wild and cultivated wheat genotypes. Three significant phyla, *Actinobacteria*, OD1 and Chlorobi, were identified based on their respective false discovery rate (FDR) values. *Actinobacteria* (FDR = 0.00028302) showed a positive correlation with the drought-treated wild genotypes, indicating higher abundance.

**Fig. 5:**
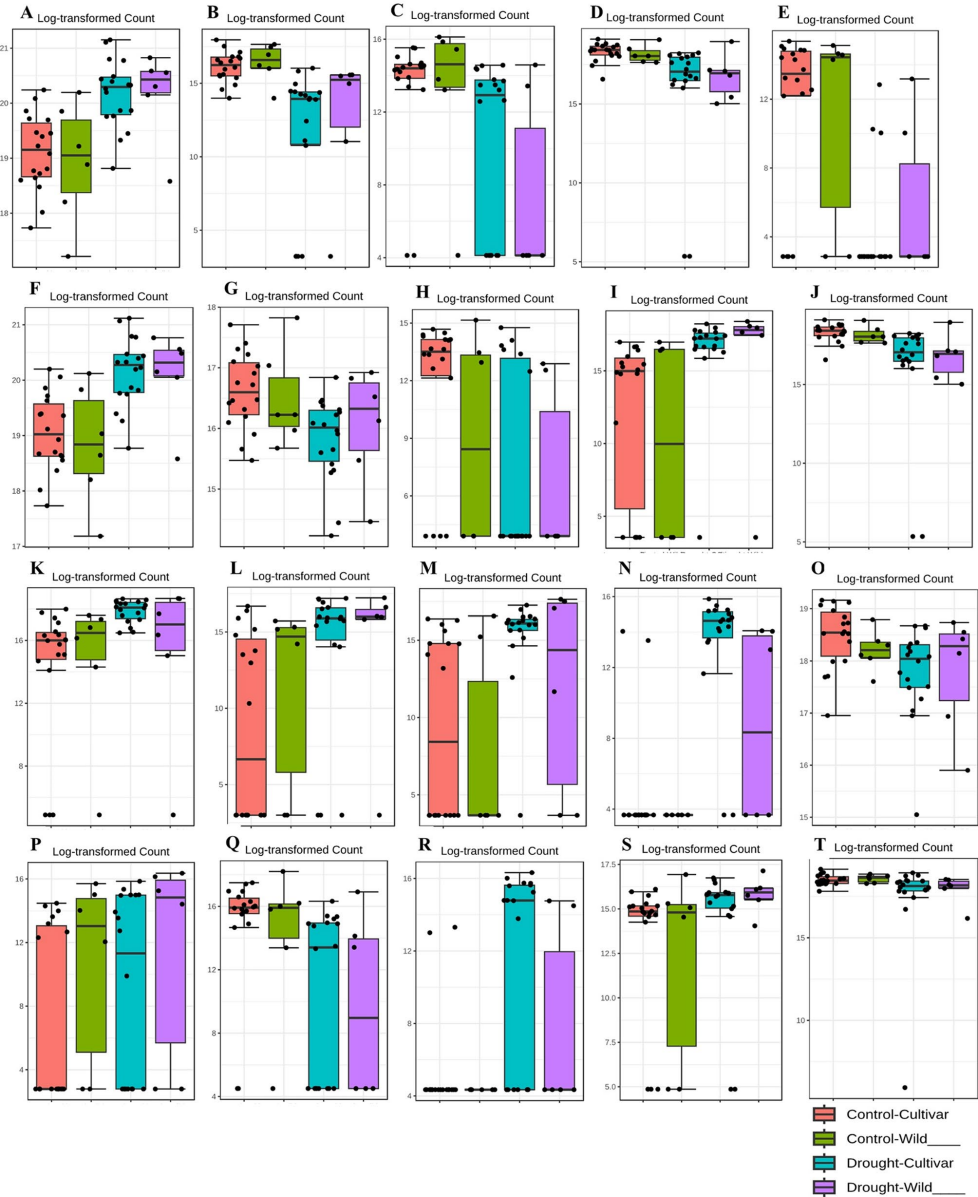
The significant microbial taxa (phylum, class and family level) with correlation patterns between drought and control are shown in boxplots. (**A**-**C**) significant phylum-level correlations to each other (*Actinobacteria*, OD1, Chlorobi). (**D**-**H**) significant class-level correlations to each other (*Verrucomicrobiae*, ABY1, *Actinobacteria*, *Bacteroidia*, TA18). (**I**-**T**) significant family-level correlations (eg. *Streptomycetaceae*, *Verrucomicrobiaceae*, *Nocardioidaceae*, *Methylobacteriaceae*). FDR-adjusted Pearson correlation values were calculated and applied

Interestingly, OD1 (FDR = 0.0012082) showed a negative correlation with both the wild and cultivated wheat genotypes under drought conditions, but showed a contrasting positive correlation under control conditions, indicating higher abundance. Similarly, Chlorobi (FDR = 0.024999) showed a negative correlation with the drought conditions but exhibited a positive correlation, indicating higher abundance under the control conditions for both the wild and cultivated genotypes (Fig. 5A, B, C) (Table 2). At the taxonomic class level, we identified five significant taxa: *Actinobacteria*, *Verrucomicrobiae*, ABY1, *Bacteroidia* and TA18, based on their respective FDR values (0.00052714, 0.00052714, 0.00065193, 0.043198, 0.043198).

Figure 5A, *Actinobacteria* showed a negative correlation with the cultivated and wild genotypes under control conditions, whereas under drought conditions they showed a contrasting positive correlation in both sample types, indicating higher abundance. Conversely, *Verrucomicrobiae*, ABY1, *Bacteroidia* and TA18 showed a positive correlation with the control genotypes, indicating higher abundance, and switched to a negative correlation under drought conditions (Fig. 5D, E, G, H) (Table 2).

We identified 12 significant taxa at the family level. The FDR was used to test the reliability of these correlations. Interestingly, the *Streptomycetaceae* showed a significantly low false discovery rate (FDR) of 1.49E-05. Moreover, under control conditions, it showed a consistent negative correlation with both cultivated and wild genotypes. However, under drought conditions, it showed a strong positive correlation and reached its highest abundance.

The *Verrucomicrobiaceae*, on the other hand, showed an FDR of 0.00087393, indicating significant correlations. This family showed a positive correlation with the cultivated genotypes under control conditions and the drought- treated wild genotypes. Conversely, it showed a negative correlation under the opposite conditions. The *Nocardioidaceae* showed an FDR of 0.0023626, also indicating a significant correlation. This family showed a positive correlation and reached its highest abundance under drought conditions in both cultivated and wild genotypes.

*Promicromonosporaceae* and *Nocardiaceae*, both with an FDR of 0.0054741, showed consistent negative correlations under control conditions and positive correlations under drought conditions, with the latter showing the highest abundance. *Brucellaceae*, despite their very low abundance, showed an FDR of 0.0079671, indicating significant correlations, especially a positive correlation under drought conditions, which stands out in terms of abundance.

*Sphingomonadaceae*, with an FDR of 0.023311, showed a positive correlation, and reached their highest abundance in the control cultivated genotypes, but showed a negative correlation under the drought-treated conditions. *Cystobacteraceae* and *Chromatiaceae* both showed an FDR of 0.028592, indicating significant correlations. *Cystobacteraceae* showed a positive correlation under drought conditions and reached its highest abundance, while *Chromatiaceae* showed a positive correlation under control conditions and also reached its highest abundance.

Finally, *Glycomycetaceae*, *Methylobacteriaceae* and *Xanthomonadaceae*, all with an FDR of 0.030596, showed significant correlations. *Glycomycetaceae* showed a positive correlation under drought conditions and reached its highest abundance, while *Methylobacteriaceae* and *Xanthomonadaceae* showed a positive correlation with the highest abundance under control conditions (Fig. 5I, J, K, L, M, N, O, P, Q, R, S, T) (Table 3).

**Table 3:** Correlations at taxa level with environmental conditions (control vs. drought)

Taxa Level	Taxa Name	Correlation (*r*)	FDR-adjusted *p*-value
Phylum			
	*Actinobacteria*	0.57839	0.000283
	OD1	-0.52191	0.001208
	Chlorobi	-0.40392	0.024999
Class			
	*Actinobacteria*	0.57355	0.000527
	*Verrucomicrobiae*	-0.56584	0.000527
	ABY1	-0.54994	0.000652
	*Bacteroidia*	-0.39237	0.043198
	TA18	-0.38789	0.043198
Family			
	*Streptomycetaceae*	0.66618	1.49E-05
	*Verrucomicrobiaceae*	-0.56584	0.000874
	*Nocardioidaceae*	0.52858	0.002363
	*Promicromonosporaceae*	0.48963	0.005474
	*Nocardiaceae*	0.48797	0.005474
	*Brucellaceae*	0.46961	0.007967
	*Sphingomonadaceae*	-0.42520	0.023311
	*Cystobacteraceae*	0.40735	0.028592
	*Chromatiaceae*	-0.40709	0.028592
	*Glycomycetaceae*	0.39963	0.030596
	*Methylobacteriaceae*	0.39337	0.030596
	*Xanthomonadaceae*	-0.39224	0.030596

The Pearson correlation values, and the p-values adjusted for the false discovery rate (FDR) ensure statistical robustness

### Core Microbiome

We defined the core microbes based on their prevalence in all analyzed rhizosphere genotypes (prevalence = 1) and their relative abundance of more than 0.01%. The result of this analysis was presented in the form of a heat map where the Y-axis shows the prevalence of core features in the detection limit (relative abundance), which is plotted on the X-axis.

**Fig. 6:**
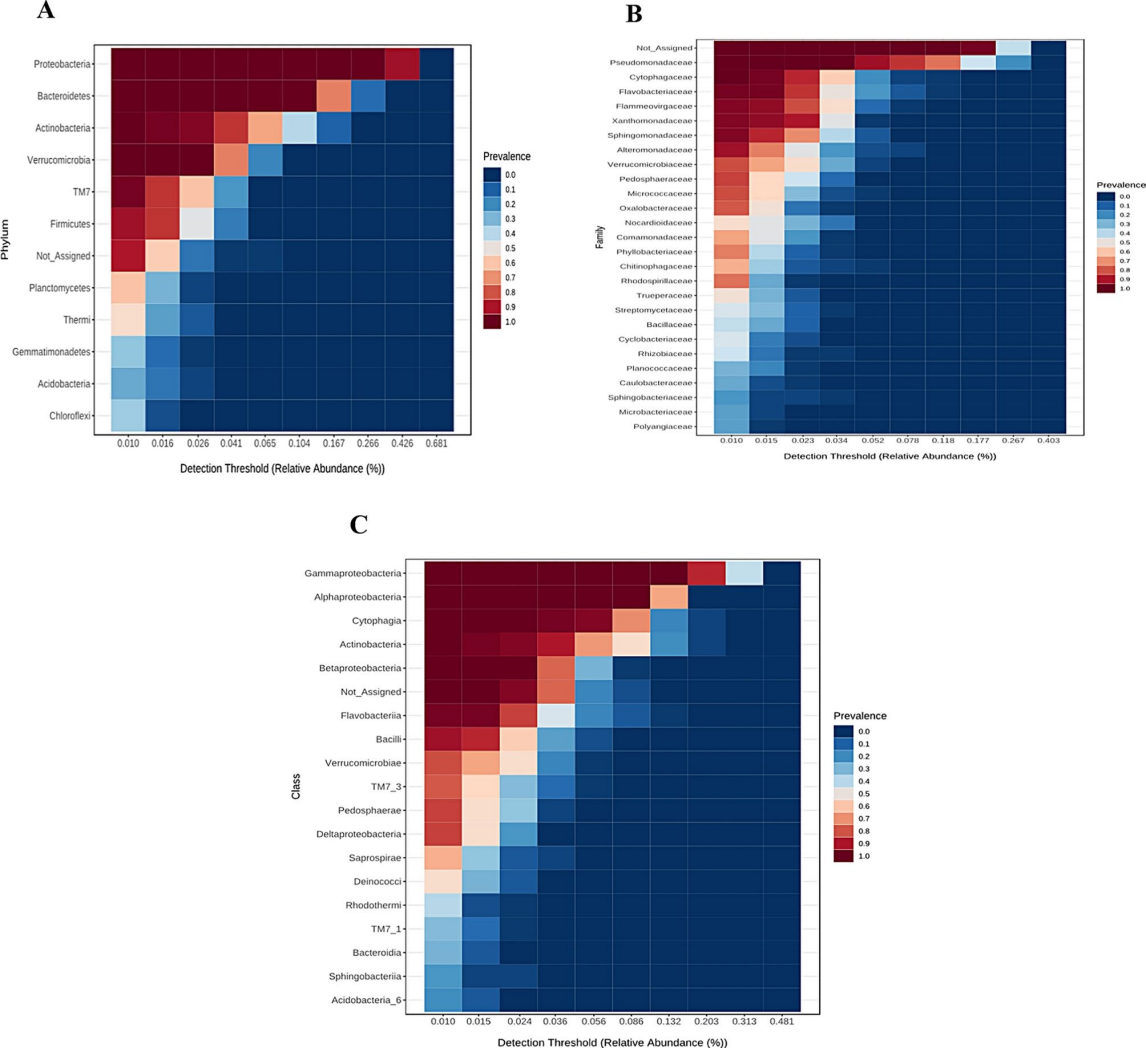
Heatmap of the composition of the core microbiome under drought and control conditions. (**A**) Core microbiome at phylum level, (**B**) core microbiome at class level, (**C**) core microbiome at family level. The persistently high prevalence of core taxa, especially *Proteobacteria*, *Actinobacteria*, and *Verrucomicrobia*, also highlights the fundamental importance of these taxonomic groups for the wheat rhizosphere ecosystem. Detection thresholds: 20% sample prevalence, relative abundance of 0.01%

In Figure (6), we present the results of the analysis of the core microbiome using a heat map that provides a clear visualization of the prevalence of core features across the spectrum of relative abundance. This visualization improved our understanding of the stable components within the microbial community and their importance in shaping the overall ecosystem. Four major phyla consistently met these criteria and together accounted for 23.5% of the observed phyla in the wheat rhizosphere. Among these major phyla, *Verrucomicrobia*, *Proteobacteria*, *Bacteroidetes* and *Actinobacteria* stood out as dominant taxa, emphasizing their significant presence and potential role in the rhizosphere ecosystem (Fig. 6A).

At the class level, however, 13.5% of the total taxa observed were assigned to five key classes: *Gammaproteobacteria*, *Cytophagia*,* Betaproteobacteria*, *Alphaproteobacteria* and *Actinobacteria*. These classes were identified as key components due to their uniform prevalence in all rhizosphere genotypes (Fig. 6B). In addition, Pseudomonadaceae and Cytophagaceae were consistent at the family level (Fig. 6C).

#### Functional prediction analysis

**Fig. 7:**
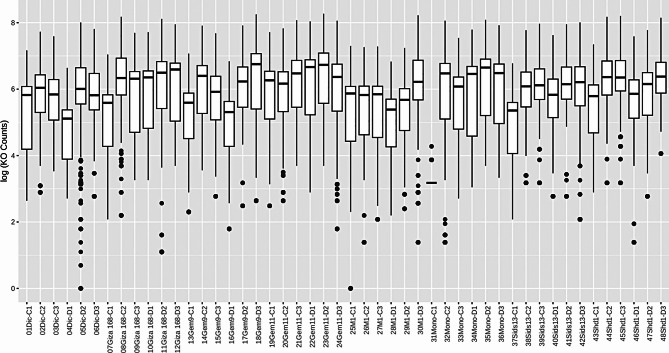
Stacked area chart showing the relative abundance of predicted metagenomic functions (KEGG pathways) in the wheat rhizosphere microbiomes under drought and control conditions using PICRUSt analysis. The Y-axis represents the relative abundance of the functional categories, indicating shifts in microbial metabolic potential

Phylogenetic Investigation of Communities by Reconstruction of Unobserved States (PICRUSt) was used to estimate the functional potential resulting from the information on the 16 S rRNA gene (Fig. 7). This approach allowed the inference of microbial functional profiles based on their genetic proximity to fully sequenced and annotated organisms. The results provided a table of gene family frequencies for each sample annotated via the KEGG database (KO) [S2].

**Fig. 8:**
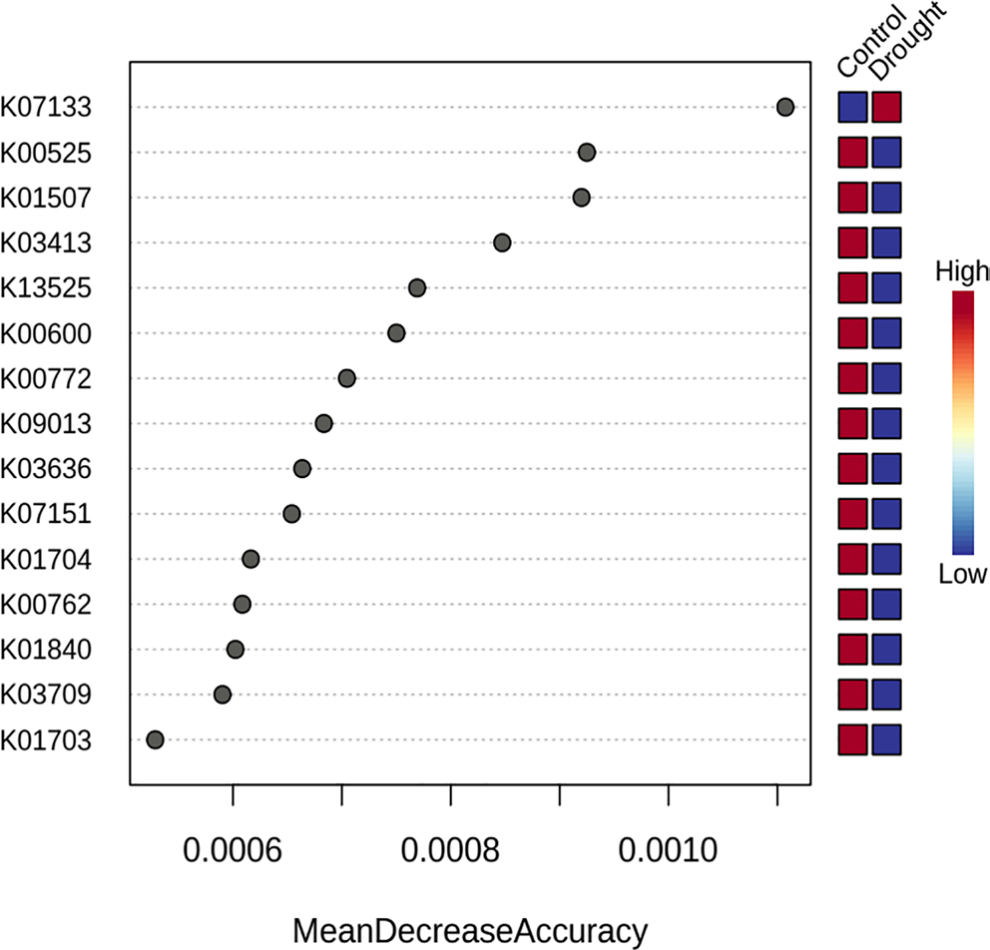
Predictive functional genes of drought-treated and control genotypes, analyzed with Random Forest (RF). (**A**) Mean Decrease Accuracy (MDA) ranking of the 15 most predictive KEGG orthologs (KOs). (**B**) Classification importance (scaled) of various key microbial functional genes. RF identified K07133 (ATP-binding protein of the AAA + superfamily) as the most predictive feature that may be involved in microbial stress adaptation under drought conditions. The out-of-bag (OOB) error rate (14.2%) and the area under the curve (AUC) score (0.91) indicate a high classification accuracy

A graph was created showing the frequency of gene families for each sample, with gene families annotated using the KEGG database (KO) (Fig. 8). In addition, the predicted metagenomes were categorized into broader functional groups using RF analysis and linked to metabolic pathways to improve the understanding of functional potential within the microbial community.

The main finding of this analysis is that the predicted metagenomic functions in the wheat rhizosphere microbiome show significant shifts under drought conditions. The analysis revealed a set of fifteen KOs with high predictive significance, including K07133, K00525, K01507, K03413, K13525, K00600, K00772, K09013, K03636, K07151, K01704, K00762, K01840, K03709 and K01703. The identification of K07133 (uncharacterized protein) as the only significantly associated KO with drought-treated genotypes suggests a unique microbial functional adaptation or stress response that does not occur under normal conditions.

This KO, recognized as an uncharacterized protein comprising an ATP-binding protein, ATPase, AAA + superfamily, showed the highest decrease in mean value, indicating its significant predictive influence in the studied microbiome (Fig. 8).

- In contrast, the other 14 KOs were predominantly associated with control genotypes, suggesting that metabolic pathways related to nucleotide metabolism (ribonucleoside diphosphate reductase, pyrE), energy metabolism (inorganic pyrophosphatase, Glycine hydroxymethyltransferase), stress response (Fe-S cluster assembly ATP-binding protein) and protein processing (transitional endoplasmic reticulum ATPase, glycosyltransferase) may be more active or stable under non-stressed conditions. This suggests that drought stress selectively alters microbial functional potential, possibly leading to the suppression of certain metabolic activities while promoting specific adaptations.

## Discussion

In this study, the composition of the rhizosphere microbiome was successfully characterized by a combination of metagenomic methods and the influence of water availability and line-specific selection on microbial diversity and abundance was investigated. Under drought conditions, certain phyla and classes showed significant enrichment in the microbiomes of drought-tolerant wheat lines. This research provides insights into the effects of drought on microbial communities associated with wheat rhizospheres.

### Microbial diversity and drought response

While neither the alpha diversity (observed species richness) nor the Bray-Curtis dissimilarity analysis indicated pronounced differences in microbial community composition between the control and drought conditions. Nevertheless, significant differences in microbial community composition between the control and drought conditions were detected using weighted UniFrac analysis, indicating a key role of phylogenetic structure in determining microbial responses to drought stress (Gu et al. 2024; Hone et al. 2020).

This discrepancy suggests that drought stress may influence evolutionary lineages rather than community richness per se. Furthermore, analysis of differential abundance revealed certain microbial taxa whose abundance differed significantly between drought and control conditions, including *Actinobacteria* and *Verrucomicrobia*, which are hypothesized to potentially play a role in drought adaptation (Fan et al. 2023; Karasov et al. 2022).

In contrast, correlation analysis provided additional insight into these relationships by revealing changes in microbial community composition associated with drought stress. This was supported by the analyzes of the Shannon and Simpson indices, which showed significant differences (*p* < 0.001) in microbial evenness and diversity, while species richness remained stable. This emphasizes the importance of implementing the complementary approaches represented by counting richness and evenness metrics based on the physiological response of microbial communities under abiotic stress (Gargallo-Garriga et al. 2018; Parejko 2024).

The weighted UniFrac analysis revealed significant dissimilarities in phylogenetic composition, emphasizing the need to understand both taxonomic and evolutionary releationships of microbial communities under drought conditions. Core taxa, including *Verrucomicrobia*, *Proteobacteria*, *Bacteroidetes*, and *Actinobacteria*, play a critical role in maintaining plant health and function (X. Li et al. 2023a, b). Nevertheless, the core wheat microbiome is primarily dominated by *Proteobacteria* and *Actinobacteria* (Naylor et al. 2017).

### Diversity metrics and contrasting evidence on microbial shifts

Drought stress conditions were characterized by significant changes in the composition of the microbial communities, with an increased relative abundance of the bacterial phyla *Actinobacteria*, *Bacteroidetes*, *Proteobacteria*, and *Verrucomicrobia*. The clear enrichment of *Actinobacteria* under drought is worth mentioning, which is consistent with previous studies that highlight high adaptive capabilities and their positive roles for plant drought tolerance (Fan et al. 2023).

In contrast, the overall abundance of *Proteobacteria* reduced moderately under drought which agrees with the trend be seen by Karasov et al. (2022) showing drought-driven shifts in microbial traits favoring certain microbes with the observed gram-negative reduction. In parallel, the concomitant declines in Shannon diversity and evenness values of dominant phyla reinforce how drought alters the dynamics and competitive interactions among microbial communities.

For microbial adaptive responses at the taxonomic class level, drought-induced responses included a marked reduction in *Gammaproteobacteria* and a significant increase in *Alphaproteobacteria* and *Cytophagia* for adaptive responses under drought conditions. A further reduction in Gammaproteobacteria is likely a sign of increased microbial competition and nutrient limitation often emphasised under drought conditions, as also described by Li et al. (2023a, b) and Romanenko et al. (2025).

On the contrary, identified enrichment of *Alphaproteobacteria* and *Cytophagia* indicates the plasticity of these microbial classes with the potential for roles such as improved nutrient cycling, organic matter breakdown, and plant nutrient acquisition under stressful environments (Upadhyay et al. 2020). These changes in microbial community structure provide insight into real-time ecological responses to drought stress and represent targets for microbe-mediated applications to promote plant performance under water-limited environments.

### Functional potentials and inferred mechanisms

The drought-induced differential abundance analyses highlighted previously uncharacterized strong drought-independent taxa such as OD1, WS2, Chlorobi, ABY1, and SHA-109, emphasizing their ecological relevance under drought conditions. Though rare, these taxa may modulate soil microbiome functional properties such as nutrient cycling, carbon fixation, and moisture holding capacity, which may in turn lead to improved plant drought tolerance (Breitkreuz et al. 2021; Hone et al. 2021). Chlorobi is associated with functioning alongside other slow-growing taxa, suggesting that even small, non-plant-associated microbial taxa can play an outsized role in the stability of the entire ecosystem, as well as the overall ability of plants to cope with abiotic stress (Hone et al. 2021).

Moreover, several important microbial classes with key functions in plant–microbe interactions, like *Alphaproteobacteria* as and *Cytophagia*, exhibited significant adaptive responses under drought. *Alphaproteobacteria* are known to enhance plant nutrient uptake during drought conditions by mobilizing nutrients (especially phosphorus), since the availability of nutrients is often limited by a small soil moisture pool at a time (Hone et al. 2021; Jochum et al. 2019). Moreover, this group also synthesizes phytohormones like auxins and cytokinin which stimulate root growth and increase plant stress tolerance that may result in increased wheat yield under drought stress (Singh et al. 2023).

In contrast, *Cytophagia* provides an important input to decomposition of organic matter and recycling of nutrients (Ahmed et al. 2020; Guizani et al. 2023; Upadhyay et al. 2020), processes that are crucial in the maintenance of soil health and water retention potential in arid areas. The presence of ABY1 and SHA-109 taxa could also indicate important roles of microbes in soil media with metabolic activities to produce exopolysaccharides that can play roles for soil structural changes and moisture retention, and it can also interact positively with plants and plants’ associated microbiomes (Ilyas et al. 2020; Manjunatha et al. 2022). Incorporating these microbial discoveries into agricultural management practices may provide tangible methods to sustainably improve drought resilience in plants.

Nevertheless, functional potentials were revealed by associated taxa (OD1, WS2, ABY1 and SHA-109) with improved resistance of wheat under drought stress. Two orthologs, OD1 and WS2, are associated with increasing nutrient availability in soil during drought periods when water is limited. These taxa may help improve the solubilization of phosphorus and other essential nutrients, making them more available for root uptake, which is critical for maintaining wheat growth under drought conditions (Breitkreuz et al. 2021).

ABY1 and SHA-109 are associated with the biosynthesis of phytohormones that can promote root development and improve water use efficiency (Manjunatha et al. 2022). In addition, these taxa may play a role in the production of exopolysaccharides that maintain soil moisture and improve soil structure, which further promotes plant growth during drought (Ilyas et al. 2020). The presence of these helpful microbes in the rhizosphere of wheat could create more favorable soils for growth, thus increasing overall drought tolerance and productivity (Jochum et al. 2019).

## Conclusion

This study expands our understanding of the responses of the wheat rhizosphere microbiome to drought and identifies key microbial taxa and functional pathways that contribute to plant resilience. The enrichment of *Actinobacteria*, *Verrucomicrobia* and *Alphaproteobacteria* under drought stress highlights their importance for nutrient cycling, root health and the drought adaptation process.

The discovery of a drought-related ATP-binding protein suggests a new molecular mechanism that can be further pursued. The role of K07133 should be further investigated as its function has not yet been characterized. It might be involved in a drought-specific survival strategy, such as osmoprotection, stress signaling, or metabolic reconfiguration within the rhizosphere microbiome.

Validation of beneficial strains alongside field deployment to test their ability to improve drought tolerance in wheat, exploration of microbial-molecular interactions (focus on ATPases, known plant-microbe signaling), and targeted engineering strategies combining synthetic microbial communities with precise resonance-related patterns for improved stress-resistance would be very valuable next steps in applying the HP mechanism of microbiome engineering.

Integrating the management of the rhizosphere microbiome with genetic selection and functional genomics will develop innovative sustainable strategies to improve climate resilience of wheat under water stress. These results suggest that drought-tolerant crops can help address the global challenge of food insecurity without the need for root bioengineering. However, they need to be integrated into multidisciplinary approaches in the face of climate change.

## Supplementary Information

Below is the link to the electronic supplementary material.

Supplementary Material 1

Supplementary Material 2

## Data Availability

Sequence data that support the findings of this study have been deposited in the National Center for Biotechnology Information with the accession number “SRA data: PRJNA1050913”.
