# Supplementary material for: Metagenomic insight into drought-induced changes in the Egyptian wheat rhizosphere microbiome
Source: World J Microbiol Biotechnol. 2025 Aug 12;41(8):310. doi: 10.1007/s11274-025-04518-0 (PMC12343654; doi:10.1007/s11274-025-04518-0)
Supplement: Supplementary file 1 — Supplementary Material 1 [file 11274_2025_4518_MOESM1_ESM.docx]

**Table S1: Operational taxonomic unit table (OTUs).**

| Kingdom | Phylum | Class | Order | Family | Genus | Species |
| --- | --- | --- | --- | --- | --- | --- |
| k__Bacteria | p__Actinobacteria | c__MB-A2-108 | o__ | f__ | g__ | s__ |
| k__Bacteria | p__Proteobacteria | c__Alphaproteobacteria | o__Rhizobiales | f__Phyllobacteriaceae | __ | __ |
| k__Bacteria | p__Proteobacteria | c__Deltaproteobacteria | o__Myxococcales | __ | __ | __ |
| k__Bacteria | p__Firmicutes | c__Bacilli | o__Bacillales | f__Planococcaceae | g__Sporosarcina | s__aquimarina |
| k__Bacteria | p__Actinobacteria | c__Actinobacteria | o__Actinomycetales | __ | __ | __ |
| k__Bacteria | p__Proteobacteria | c__Betaproteobacteria | __ | __ | __ | __ |
| k__Bacteria | p__Bacteroidetes | c__Cytophagia | o__Cytophagales | f__Cytophagaceae | g__ | s__ |
| k__Bacteria | p__Bacteroidetes | c__[Saprospirae] | o__[Saprospirales] | f__Chitinophagaceae | __ | __ |
| k__Bacteria | p__Verrucomicrobia | c__[Pedosphaerae] | o__[Pedosphaerales] | f__[Pedosphaeraceae] | g__ | s__ |
| k__Bacteria | p__Proteobacteria | c__Alphaproteobacteria | o__Rhizobiales | f__Aurantimonadaceae | g__Aurantimonas | __ |
| k__Bacteria | p__Proteobacteria | c__Alphaproteobacteria | o__Sphingomonadales | f__Sphingomonadaceae | __ | __ |
| k__Bacteria | p__Proteobacteria | c__Gammaproteobacteria | o__Xanthomonadales | f__Xanthomonadaceae | g__Aquimonas | s__voraii |
| k__Bacteria | p__Actinobacteria | c__Actinobacteria | o__Actinomycetales | f__Actinosynnemataceae | __ | __ |
| k__Bacteria | p__[Thermi] | c__Deinococci | o__Deinococcales | f__Trueperaceae | g__B-42 | s__ |
| k__Bacteria | p__Bacteroidetes | __ | __ | __ | __ | __ |
| k__Bacteria | p__Planctomycetes | c__Planctomycetia | o__Pirellulales | f__Pirellulaceae | g__planctomycete | __ |
| k__Bacteria | p__Actinobacteria | c__Actinobacteria | o__Actinomycetales | f__Microbacteriaceae | __ | __ |
| k__Bacteria | p__Proteobacteria | c__Deltaproteobacteria | __ | __ | __ | __ |
| k__Bacteria | p__Proteobacteria | c__Gammaproteobacteria | __ | __ | __ | __ |
| k__Bacteria | p__Nitrospirae | c__Nitrospira | o__Nitrospirales | f__Nitrospiraceae | g__Nitrospira | s__calida |
| k__Bacteria | p__Bacteroidetes | c__Cytophagia | o__Cytophagales | __ | __ | __ |
| k__Bacteria | p__Gemmatimonadetes | c__Gemm-3 | o__ | f__ | g__ | s__ |
| k__Bacteria | p__TM7 | c__ | o__ | f__ | g__ | s__ |
| k__Bacteria | p__Proteobacteria | c__Gammaproteobacteria | o__Alteromonadales | f__Alteromonadaceae | g__Saccharophagus | s__degradans |
| k__Bacteria | p__Proteobacteria | c__Gammaproteobacteria | o__Pseudomonadales | f__Pseudomonadaceae | g__Pseudomonas | __ |
| k__Bacteria | p__Proteobacteria | c__Betaproteobacteria | o__Burkholderiales | f__Comamonadaceae | __ | __ |
| k__Bacteria | p__Firmicutes | c__Bacilli | o__Bacillales | f__Bacillaceae | g__Bacillus | __ |
| k__Bacteria | p__Bacteroidetes | c__Cytophagia | o__Cytophagales | f__Flammeovirgaceae | __ | __ |
| k__Bacteria | p__Gemmatimonadetes | c__Gemmatimonadetes | o__ | f__ | g__ | s__ |
| k__Bacteria | p__Bacteroidetes | c__Flavobacteriia | o__Flavobacteriales | f__Flavobacteriaceae | g__Persicivirga | __ |
| k__Bacteria | p__Actinobacteria | c__Actinobacteria | o__Actinomycetales | f__Micromonosporaceae | __ | __ |
| k__Bacteria | p__Actinobacteria | c__Actinobacteria | o__Actinomycetales | f__Microbacteriaceae | g__Leifsonia | __ |
| k__Bacteria | p__Proteobacteria | c__Gammaproteobacteria | o__Alteromonadales | f__[Chromatiaceae] | g__Bacillus | s__firmus |
| k__Bacteria | p__Proteobacteria | c__Gammaproteobacteria | o__Alteromonadales | f__Alteromonadaceae | g__Gilvimarinus | s__chinensis |
| k__Bacteria | p__Proteobacteria | c__Gammaproteobacteria | o__Oceanospirillales | f__Halomonadaceae | g__Halomonas | __ |
| k__Bacteria | p__Proteobacteria | __ | __ | __ | __ | __ |
| k__Bacteria | p__Proteobacteria | c__Gammaproteobacteria | o__Xanthomonadales | f__Xanthomonadaceae | g__Lysobacter | __ |
| k__Bacteria | p__ | c__ | o__ | f__ | g__ | s__ |
| k__Bacteria | p__Chloroflexi | c__Anaerolineae | o__SBR1031 | f__A4b | g__ | s__ |
| k__Bacteria | p__Proteobacteria | c__Alphaproteobacteria | o__Sphingomonadales | f__Sphingomonadaceae | g__Sphingomonas | s__suberifaciens |
| k__Bacteria | p__Proteobacteria | c__Alphaproteobacteria | o__Rhizobiales | __ | __ | __ |
| k__Bacteria | p__Proteobacteria | c__Gammaproteobacteria | o__Xanthomonadales | f__Xanthomonadaceae | __ | __ |
| k__Bacteria | p__Bacteroidetes | c__Cytophagia | o__Cytophagales | f__Flammeovirgaceae | g__Marinoscillum | __ |
| k__Bacteria | p__Proteobacteria | c__Deltaproteobacteria | o__Myxococcales | f__Haliangiaceae | g__Haliangium | s__ |
| k__Bacteria | p__Gemmatimonadetes | c__Gemm-1 | o__ | f__ | g__ | s__ |
| k__Bacteria | p__Proteobacteria | c__Gammaproteobacteria | o__Pseudomonadales | f__Pseudomonadaceae | g__Azotobacter | s__armeniacus |
| k__Bacteria | p__Proteobacteria | c__Betaproteobacteria | o__Burkholderiales | f__Oxalobacteraceae | __ | __ |
| k__Bacteria | p__Proteobacteria | c__Alphaproteobacteria | __ | __ | __ | __ |
| k__Bacteria | p__Firmicutes | c__Bacilli | o__Bacillales | __ | __ | __ |
| k__Bacteria | p__Verrucomicrobia | c__Verrucomicrobiae | o__Verrucomicrobiales | f__Verrucomicrobiaceae | g__Haloferula | s__helveola |
| k__Bacteria | p__Bacteroidetes | c__Flavobacteriia | o__Flavobacteriales | f__Flavobacteriaceae | __ | __ |
| k__Bacteria | p__Proteobacteria | c__Alphaproteobacteria | o__Rhizobiales | f__Rhizobiaceae | g__Rhizobium | s__selenitireducens |
| k__Bacteria | p__Firmicutes | c__Bacilli | o__Bacillales | f__Paenibacillaceae | __ | __ |
| k__Bacteria | p__Proteobacteria | c__Betaproteobacteria | o__Nitrosomonadales | f__Nitrosomonadaceae | g__Nitrosospira | s__multiformis |
| k__Bacteria | p__Acidobacteria | c__Acidobacteria-6 | o__ | f__ | g__ | s__ |
| k__Bacteria | p__Actinobacteria | c__Actinobacteria | o__Actinomycetales | f__Micrococcaceae | g__Arthrobacter | __ |
| k__Bacteria | p__Planctomycetes | c__Phycisphaerae | o__WD2101 | f__ | g__ | s__ |
| k__Bacteria | p__TM7 | c__TM7-3 | __ | __ | __ | __ |
| k__Bacteria | p__Proteobacteria | c__Gammaproteobacteria | o__Xanthomonadales | f__Xanthomonadaceae | g__Lysobacter | s__pocheonensis |
| k__Bacteria | p__Actinobacteria | c__Actinobacteria | o__Actinomycetales | f__Nocardioidaceae | g__Nocardioides | __ |
| k__Bacteria | p__Proteobacteria | c__Gammaproteobacteria | o__Alteromonadales | f__Alteromonadaceae | __ | __ |
| k__Bacteria | p__Bacteroidetes | c__[Rhodothermi] | o__[Rhodothermales] | f__ | g__ | s__ |
| k__Bacteria | p__Bacteroidetes | c__Bacteroidia | o__Bacteroidales | f__ | g__ | s__ |
| k__Bacteria | p__TM7 | c__TM7-3 | o__I025 | f__ | g__ | s__ |
| k__Bacteria | p__Proteobacteria | c__Deltaproteobacteria | o__Bdellovibrionales | f__Bdellovibrionaceae | g__Bdellovibrio | s__ |
| k__Bacteria | p__Proteobacteria | c__Alphaproteobacteria | o__Rhodospirillales | f__Rhodospirillaceae | g__Skermanella | s__xinjiangensis |
| k__Bacteria | p__Firmicutes | c__Bacilli | o__Bacillales | f__Bacillaceae | __ | __ |
| k__Bacteria | p__Proteobacteria | c__Alphaproteobacteria | o__Caulobacterales | f__Caulobacteraceae | g__Brevundimonas | __ |
| k__Bacteria | p__Verrucomicrobia | c__[Spartobacteria] | o__[Chthoniobacterales] | f__[Chthoniobacteraceae] | __ | __ |
| k__Bacteria | p__Proteobacteria | c__Alphaproteobacteria | o__Sphingomonadales | f__Sphingomonadaceae | g__Sphingomonas | s__changbaiensis |
| k__Bacteria | p__Planctomycetes | c__Planctomycetia | o__Pirellulales | f__Pirellulaceae | g__planctomycete | s__DDSW3008 |
| k__Bacteria | p__Proteobacteria | c__Alphaproteobacteria | o__Rhizobiales | f__Xanthobacteraceae | g__Ancylobacter | s__abiegnus |
| k__Bacteria | p__Acidobacteria | c__Solibacteres | o__Solibacterales | f__MVS-65 | g__ | s__ |
| k__Bacteria | p__BRC1 | c__PRR-11 | o__ | f__ | g__ | s__ |
| k__Bacteria | p__WS2 | c__SHA-109 | o__ | f__ | g__ | s__ |
| k__Bacteria | p__TM7 | c__TM7-1 | o__ | f__ | g__ | s__ |
| k__Bacteria | p__Firmicutes | __ | __ | __ | __ | __ |
| k__Bacteria | p__Firmicutes | c__Bacilli | o__Bacillales | f__Thermoactinomycetaceae | __ | __ |
| k__Bacteria | p__Proteobacteria | c__Alphaproteobacteria | o__Rhodospirillales | f__Rhodospirillaceae | g__Thalassobaculum | __ |
| k__Bacteria | p__Actinobacteria | c__Actinobacteria | o__Micrococcales | f__ | g__ | s__ |
| k__Bacteria | p__NKB19 | c__TSBW08 | o__ | f__ | g__ | s__ |
| k__Bacteria | p__Actinobacteria | __ | __ | __ | __ | __ |
| k__Bacteria | p__Proteobacteria | c__Gammaproteobacteria | o__Alteromonadales | __ | __ | __ |
| k__Bacteria | p__Acidobacteria | c__Acidobacteria-6 | __ | __ | __ | __ |
| k__Bacteria | p__Actinobacteria | c__Thermoleophilia | o__Gaiellales | f__Gaiellaceae | g__Gaiella | s__occulta |
| k__Bacteria | p__Bacteroidetes | c__Sphingobacteriia | o__Sphingobacteriales | f__Sphingobacteriaceae | g__Pedobacter | s__oryzae |
| k__Bacteria | p__Proteobacteria | c__Gammaproteobacteria | o__Alteromonadales | f__Alteromonadaceae | g__Teredinibacter | s__turnerae |
| k__Bacteria | p__Proteobacteria | c__Alphaproteobacteria | o__Rhizobiales | f__Hyphomicrobiaceae | __ | __ |
| k__Bacteria | p__Proteobacteria | c__Betaproteobacteria | o__Burkholderiales | f__Oxalobacteraceae | g__Herbaspirillum | __ |
| k__Bacteria | p__Proteobacteria | c__Alphaproteobacteria | o__Caulobacterales | f__Caulobacteraceae | __ | __ |
| k__Bacteria | p__Bacteroidetes | c__Cytophagia | o__Cytophagales | f__Cyclobacteriaceae | __ | __ |
| k__Bacteria | p__Proteobacteria | c__Alphaproteobacteria | o__Rhizobiales | f__Rhizobiaceae | g__Rhizobium | __ |
| k__Bacteria | p__Proteobacteria | c__Alphaproteobacteria | o__Rickettsiales | f__ | g__ | s__ |
| k__Bacteria | p__Proteobacteria | c__Alphaproteobacteria | o__Rhodospirillales | f__Rhodospirillaceae | __ | __ |
| k__Bacteria | p__Proteobacteria | c__Deltaproteobacteria | o__Myxococcales | f__Polyangiaceae | g__Aetherobacter | s__fasciculatus |
| k__Bacteria | p__Bacteroidetes | c__[Saprospirae] | o__[Saprospirales] | f__Chitinophagaceae | g__Trachelomonas | s__volvocinopsis |
| k__Bacteria | p__Proteobacteria | c__Alphaproteobacteria | o__Sphingomonadales | __ | __ | __ |
| k__Bacteria | p__Verrucomicrobia | c__Opitutae | o__[Pelagicoccales] | f__[Pelagicoccaceae] | g__Pelagicoccus | s__ |
| k__Bacteria | p__Proteobacteria | c__Alphaproteobacteria | o__Rhodospirillales | __ | __ | __ |
| k__Bacteria | p__Verrucomicrobia | c__Verrucomicrobiae | o__Verrucomicrobiales | f__Verrucomicrobiaceae | g__Haloferula | s__rosea |
| k__Bacteria | p__Proteobacteria | c__Deltaproteobacteria | o__Bdellovibrionales | f__Bacteriovoracaceae | g__Peredibacter | s__starrii |
| k__Bacteria | p__OD1 | c__ | o__ | f__ | g__ | s__ |
| k__Bacteria | p__Bacteroidetes | c__Sphingobacteriia | o__Sphingobacteriales | f__Sphingobacteriaceae | g__Pedobacter | s__composti |
| k__Bacteria | p__Proteobacteria | c__Deltaproteobacteria | o__ | f__ | g__ | s__ |
| k__Bacteria | p__Verrucomicrobia | c__Verrucomicrobiae | o__Verrucomicrobiales | f__Verrucomicrobiaceae | g__Haloferula | s__sargassicola |
| k__Bacteria | p__Chlorobi | c__ | o__ | f__ | g__ | s__ |
| k__Bacteria | p__Proteobacteria | c__Betaproteobacteria | o__Burkholderiales | __ | __ | __ |
| k__Bacteria | p__Proteobacteria | c__Gammaproteobacteria | o__Alteromonadales | f__OM60 | g__Haliea | s__mediterranea |
| k__Bacteria | p__Proteobacteria | c__Deltaproteobacteria | o__Myxococcales | f__Polyangiaceae | __ | __ |
| k__Bacteria | p__Proteobacteria | c__TA18 | o__CV90 | f__ | g__ | s__ |
| k__Bacteria | p__Firmicutes | c__Bacilli | o__Lactobacillales | f__Lactobacillaceae | g__Pediococcus | __ |
| k__Bacteria | p__Proteobacteria | c__Gammaproteobacteria | o__Alteromonadales | f__[Chromatiaceae] | g__Rheinheimera | __ |
| k__Bacteria | p__Proteobacteria | c__Gammaproteobacteria | o__Xanthomonadales | f__Xanthomonadaceae | g__Lysobacter | s__oryzae |
| k__Bacteria | p__Proteobacteria | c__Gammaproteobacteria | o__Oceanospirillales | f__Hahellaceae | g__ | s__ |
| k__Bacteria | p__Proteobacteria | c__Betaproteobacteria | o__Burkholderiales | f__Comamonadaceae | g__[Polyangium] | s__brachysporum |
| k__Bacteria | p__Bacteroidetes | c__Flavobacteriia | o__Flavobacteriales | f__Flavobacteriaceae | g__Nonlabens | __ |
| k__Bacteria | p__Actinobacteria | c__Actinobacteria | o__Actinomycetales | f__Streptomycetaceae | g__Streptomyces | __ |
| k__Bacteria | p__Chloroflexi | c__Anaerolineae | __ | __ | __ | __ |
| k__Bacteria | p__Acidobacteria | c__Solibacteres | o__Solibacterales | f__OPB3 | g__ | s__ |
| k__Bacteria | p__Firmicutes | c__Bacilli | o__Bacillales | f__Planococcaceae | __ | __ |
| k__Bacteria | p__OD1 | c__SM2F11 | o__ | f__ | g__ | s__ |
| k__Bacteria | p__Proteobacteria | c__Gammaproteobacteria | o__Legionellales | f__Legionellaceae | g__Legionella | __ |
| k__Bacteria | p__Proteobacteria | c__Alphaproteobacteria | o__Rhizobiales | f__Brucellaceae | __ | __ |
| k__Bacteria | p__Proteobacteria | c__Betaproteobacteria | o__Nitrosomonadales | f__Nitrosomonadaceae | __ | __ |
| k__Bacteria | p__Bacteroidetes | c__Flavobacteriia | o__Flavobacteriales | f__Cryomorphaceae | g__Owenweeksia | s__hongkongensis |
| k__Bacteria | p__OD1 | __ | __ | __ | __ | __ |
| k__Bacteria | p__Actinobacteria | c__Actinobacteria | o__Actinomycetales | f__Glycomycetaceae | __ | __ |
| k__Bacteria | p__Firmicutes | c__Clostridia | o__Clostridiales | f__Lachnospiraceae | g__Ruminococcus | __ |
| k__Bacteria | p__Planctomycetes | c__Planctomycetia | o__Gemmatales | f__Isosphaeraceae | __ | __ |
| k__Bacteria | p__Bacteroidetes | c__Flavobacteriia | o__Flavobacteriales | f__Flavobacteriaceae | g__Salinimicrobium | s__catena |
| k__Bacteria | p__Proteobacteria | c__Betaproteobacteria | o__Methylophilales | f__Methylophilaceae | g__Methylovorus | __ |
| k__Bacteria | p__Proteobacteria | c__Gammaproteobacteria | o__Chromatiales | f__Ectothiorhodospiraceae | g__Thioalkalivibrio | __ |
| k__Bacteria | p__Bacteroidetes | c__Cytophagia | o__Cytophagales | f__Cytophagaceae | __ | __ |
| k__Bacteria | p__Proteobacteria | c__Gammaproteobacteria | o__Xanthomonadales | f__Xanthomonadaceae | g__Aspromonas | s__composti |
| k__Bacteria | p__Acidobacteria | c__[Chloracidobacteria] | o__[Chloracidobacterales] | f__[Chloracidobacteraceae] | g__ | s__ |
| k__Bacteria | p__Planctomycetes | c__Planctomycetia | __ | __ | __ | __ |
| k__Bacteria | p__Elusimicrobia | c__Elusimicrobia | o__IIb | f__ | g__ | s__ |
| k__Bacteria | p__Bacteroidetes | c__Cytophagia | o__Cytophagales | f__Flammeovirgaceae | g__Marinoscillum | s__furvescens |
| k__Bacteria | p__TM7 | c__TM7-3 | o__EW055 | f__ | g__ | s__ |
| k__Bacteria | p__Proteobacteria | c__Alphaproteobacteria | o__Rhodospirillales | f__Rhodospirillaceae | g__Stella | s__humosa |
| k__Bacteria | p__Planctomycetes | __ | __ | __ | __ | __ |
| k__Bacteria | p__Chloroflexi | c__Anaerolineae | o__Caldilineales | f__Caldilineaceae | g__Litorilinea | s__aerophila |
| k__Bacteria | p__Proteobacteria | c__Deltaproteobacteria | o__Myxococcales | f__Cystobacteraceae | __ | __ |
| k__Bacteria | p__OD1 | c__ABY1 | o__ | f__ | g__ | s__ |
| k__Bacteria | p__Proteobacteria | c__Alphaproteobacteria | o__Sphingomonadales | f__Sphingomonadaceae | g__Sphingosinicella | s__microcystinivorans |
| k__Bacteria | p__Proteobacteria | c__Betaproteobacteria | o__Rhodocyclales | f__Rhodocyclaceae | g__Denitromonas | s__indolicum |
| k__Bacteria | p__TM7 | c__TM7-3 | o__CW040 | f__ | g__ | s__ |
| k__Bacteria | p__Actinobacteria | c__Actinobacteria | o__Actinomycetales | f__Streptosporangiaceae | __ | __ |
| k__Bacteria | p__Proteobacteria | c__Gammaproteobacteria | o__Xanthomonadales | __ | __ | __ |
| k__Bacteria | p__Proteobacteria | c__Gammaproteobacteria | o__Pseudomonadales | f__Pseudomonadaceae | __ | __ |
| k__Bacteria | p__Actinobacteria | c__Actinobacteria | o__Actinomycetales | f__Micrococcaceae | g__Arthrobacter | s__crystallopoietes |
| k__Bacteria | p__Proteobacteria | c__Alphaproteobacteria | o__Rhizobiales | f__Methylobacteriaceae | __ | __ |
| k__Bacteria | p__Proteobacteria | c__Betaproteobacteria | o__Burkholderiales | f__Oxalobacteraceae | g__Massilia | __ |
| k__Bacteria | p__Chloroflexi | c__TK17 | o__ | f__ | g__ | s__ |
| k__Bacteria | p__Proteobacteria | c__Betaproteobacteria | o__Burkholderiales | f__Alcaligenaceae | g__Bordetella | __ |
| k__Bacteria | p__GN02 | c__GKS2-174 | o__ | f__ | g__ | s__ |
| k__Bacteria | p__Proteobacteria | c__Alphaproteobacteria | o__Rhodobacterales | f__Hyphomonadaceae | g__Woodsholea | s__maritima |
| k__Bacteria | p__Actinobacteria | c__Actinobacteria | o__Actinomycetales | f__Nocardiaceae | g__Rhodococcus | __ |
| k__Bacteria | p__Elusimicrobia | c__Elusimicrobia | o__MVP-88 | f__ | g__ | s__ |
| k__Bacteria | p__Proteobacteria | c__Alphaproteobacteria | o__Rhodospirillales | f__Acetobacteraceae | g__Gluconacetobacter | __ |
| k__Bacteria | p__Actinobacteria | c__Actinobacteria | o__Actinomycetales | f__Kineosporiaceae | g__Kineosporia | __ |
| k__Bacteria | p__Proteobacteria | c__Betaproteobacteria | o__Burkholderiales | f__Oxalobacteraceae | g__Massilia | s__aurea |
| k__Bacteria | p__Actinobacteria | c__Actinobacteria | o__Actinomycetales | f__Geodermatophilaceae | __ | __ |
| k__Bacteria | p__Proteobacteria | c__Alphaproteobacteria | o__Rhodospirillales | f__Rhodospirillaceae | g__Phaeospirillum | s__fulvum |
| k__Bacteria | p__Actinobacteria | c__Actinobacteria | o__Actinomycetales | f__Nocardioidaceae | g__Nocardioides | s__exalbidus |
| k__Bacteria | p__Bacteroidetes | c__Cytophagia | o__Cytophagales | f__Cyclobacteriaceae | g__Algoriphagus | s__terrigena |
| k__Bacteria | p__Chloroflexi | c__Thermomicrobia | o__JG30-KF-CM45 | f__ | g__ | s__ |
| k__Bacteria | p__Proteobacteria | c__Alphaproteobacteria | o__Sphingomonadales | f__Erythrobacteraceae | g__Porphyrobacter | __ |
| k__Bacteria | p__Proteobacteria | c__Alphaproteobacteria | o__Sphingomonadales | f__Erythrobacteraceae | g__Altererythrobacter | s__epoxidivorans |
| k__Bacteria | p__Proteobacteria | c__Alphaproteobacteria | o__Sphingomonadales | f__Erythrobacteraceae | g__Erythrobacter | s__gaetbuli |
| k__Bacteria | p__Proteobacteria | c__Alphaproteobacteria | o__Sphingomonadales | f__Erythrobacteraceae | __ | __ |
| k__Bacteria | p__Bacteroidetes | c__Flavobacteriia | o__Flavobacteriales | f__Cryomorphaceae | g__Lishizhenia | s__caseinilytica |
| k__Bacteria | p__Proteobacteria | c__Betaproteobacteria | o__Burkholderiales | f__Alcaligenaceae | g__Bordetella | s__holmesii |
| k__Bacteria | p__Proteobacteria | c__Alphaproteobacteria | o__Sphingomonadales | f__Sphingomonadaceae | g__Sphingomonas | __ |
| k__Bacteria | p__Verrucomicrobia | c__Verrucomicrobiae | o__Verrucomicrobiales | f__Verrucomicrobiaceae | g__Verrucomicrobium | s__spinosum |
| k__Bacteria | p__Chloroflexi | c__Gitt-GS-136 | o__ | f__ | g__ | s__ |
| k__Bacteria | p__Bacteroidetes | c__Flavobacteriia | o__Flavobacteriales | __ | __ | __ |
| k__Bacteria | p__Proteobacteria | c__Betaproteobacteria | o__Burkholderiales | f__Comamonadaceae | g__Limnobacter | s__litoralis |
| k__Bacteria | p__Proteobacteria | c__Alphaproteobacteria | o__Caulobacterales | f__Caulobacteraceae | g__Brevundimonas | s__vesicularis |
| k__Bacteria | p__OP11 | c__OP11-4 | o__ | f__ | g__ | s__ |
| k__Bacteria | p__Proteobacteria | c__Betaproteobacteria | o__Burkholderiales | f__Comamonadaceae | g__Aquamonas | s__fontana |
| k__Bacteria | p__Actinobacteria | c__Actinobacteria | o__Actinomycetales | f__Thermomonosporaceae | g__Actinomadura | __ |
| k__Bacteria | p__Proteobacteria | c__Alphaproteobacteria | o__Rhizobiales | f__Rhizobiaceae | __ | __ |
| k__Bacteria | p__Proteobacteria | c__Alphaproteobacteria | o__Rhizobiales | f__Aurantimonadaceae | g__Aurantimonas | s__ureilytica |
| k__Bacteria | p__[Thermi] | c__Deinococci | o__Deinococcales | __ | __ | __ |
| k__Bacteria | p__Chloroflexi | c__Anaerolineae | o__CFB-26 | f__ | g__ | s__ |
| k__Bacteria | p__[Thermi] | c__Deinococci | o__Deinococcales | f__Trueperaceae | g__ | s__ |
| k__Bacteria | p__Planctomycetes | c__BD7-11 | o__ | f__ | g__ | s__ |
| k__Bacteria | p__Fibrobacteres | c__Fibrobacteria | o__258ds10 | f__ | g__ | s__ |
| k__Bacteria | p__Proteobacteria | c__Alphaproteobacteria | o__Rickettsiales | f__Rickettsiaceae | g__Rickettsia | s__Rickettsia endosymbiont of Deronectes platynotus |
| k__Bacteria | p__Bacteroidetes | c__[Saprospirae] | o__[Saprospirales] | f__Chitinophagaceae | g__Parasegitibacter | s__luojiensis |
| k__Bacteria | p__Actinobacteria | c__Actinobacteria | o__Actinomycetales | f__Geodermatophilaceae | g__Blastococcus | s__jejuensis |
| k__Bacteria | p__Proteobacteria | c__Alphaproteobacteria | o__Rhodospirillales | f__Acetobacteraceae | __ | __ |
| k__Bacteria | p__Firmicutes | c__Clostridia | o__Clostridiales | f__Ruminococcaceae | g__Faecalibacterium | s__ |
| k__Bacteria | p__Proteobacteria | c__Alphaproteobacteria | o__Rhizobiales | f__Phyllobacteriaceae | g__Hoeflea | __ |
| k__Bacteria | p__Proteobacteria | c__Alphaproteobacteria | o__Rhodobacterales | f__Rhodobacteraceae | __ | __ |
| k__Bacteria | p__Acidobacteria | c__Solibacteres | o__Solibacterales | __ | __ | __ |
| k__Bacteria | p__Proteobacteria | c__Alphaproteobacteria | o__Rhizobiales | f__Beijerinckiaceae | __ | __ |
| k__Bacteria | p__Bacteroidetes | c__Cytophagia | o__Cytophagales | f__Flammeovirgaceae | g__Cesiribacter | s__andamanensis |
| k__Bacteria | p__Firmicutes | c__Clostridia | o__Clostridiales | f__Lachnospiraceae | g__Roseburia | s__inulinivorans |
| k__Bacteria | p__Bacteroidetes | c__Sphingobacteriia | o__Sphingobacteriales | f__Sphingobacteriaceae | g__Pedobacter | __ |
| k__Bacteria | p__Firmicutes | c__Bacilli | o__Lactobacillales | f__Lactobacillaceae | g__Lactobacillus | s__paraplantarum |
| k__Bacteria | p__Actinobacteria | c__Actinobacteria | o__Actinomycetales | f__Promicromonosporaceae | __ | __ |
| k__Bacteria | p__Bacteroidetes | c__Cytophagia | o__Cytophagales | f__Flammeovirgaceae | g__Porifericola | s__rhodea |
| k__Bacteria | p__Proteobacteria | c__Alphaproteobacteria | o__Rhizobiales | f__Xanthobacteraceae | __ | __ |
| k__Bacteria | p__Proteobacteria | c__Gammaproteobacteria | o__Enterobacteriales | f__Enterobacteriaceae | __ | __ |
| k__Bacteria | p__Verrucomicrobia | c__Verrucomicrobiae | o__Verrucomicrobiales | f__Verrucomicrobiaceae | g__Haloferula | __ |
| k__Bacteria | p__Chloroflexi | c__TK17 | o__mle1-48 | f__ | g__ | s__ |
| k__Bacteria | p__Proteobacteria | c__Gammaproteobacteria | o__Xanthomonadales | f__Xanthomonadaceae | g__Silanimonas | s__mangrovi |
| k__Bacteria | p__Firmicutes | c__Clostridia | o__Clostridiales | f__Lachnospiraceae | __ | __ |
| k__Bacteria | p__Actinobacteria | c__Actinobacteria | o__Actinomycetales | f__Nocardioidaceae | __ | __ |
| k__Bacteria | p__Actinobacteria | c__Actinobacteria | o__Actinomycetales | f__Promicromonosporaceae | g__Luteimicrobium | s__subarcticum |
| k__Bacteria | p__Bacteroidetes | c__[Rhodothermi] | o__[Rhodothermales] | f__[Balneolaceae] | g__Balneola | s__ |
| k__Bacteria | p__Proteobacteria | c__Gammaproteobacteria | o__Xanthomonadales | f__Sinobacteraceae | g__Panacagrimonas | s__perspica |
| k__Bacteria | p__Proteobacteria | c__Gammaproteobacteria | o__Alteromonadales | f__[Chromatiaceae] | g__Rheinheimera | s__nanhaiensis |
| k__Bacteria | p__Chloroflexi | c__Anaerolineae | o__Anaerolineales | f__Anaerolinaceae | g__Leptolinea | s__tardivitalis |
| k__Bacteria | p__Proteobacteria | c__Betaproteobacteria | o__Rhodocyclales | f__Rhodocyclaceae | __ | __ |
| k__Bacteria | p__Cyanobacteria | c__ML635J-21 | o__ | f__ | g__ | s__ |
| k__Bacteria | p__Proteobacteria | c__Gammaproteobacteria | o__Xanthomonadales | f__Xanthomonadaceae | g__Pseudoxanthomonas | __ |
| k__Bacteria | p__Bacteroidetes | c__Cytophagia | o__Cytophagales | f__Cyclobacteriaceae | g__Algoriphagus | __ |
| k__Bacteria | p__Firmicutes | c__Bacilli | __ | __ | __ | __ |
| k__Bacteria | p__Chloroflexi | c__SAR202 | o__ | f__ | g__ | s__ |
| k__Bacteria | p__Proteobacteria | c__Gammaproteobacteria | o__Xanthomonadales | f__Xanthomonadaceae | g__Lysobacter | s__niabensis |
| k__Bacteria | p__Actinobacteria | c__Acidimicrobiia | o__Acidimicrobiales | f__C111 | g__Ilumatobacter | s__fluminis |
| k__Bacteria | p__Proteobacteria | c__Gammaproteobacteria | o__Oceanospirillales | __ | __ | __ |
| k__Bacteria | p__OD1 | c__ZB2 | o__ | f__ | g__ | s__ |
| k__Bacteria | p__Proteobacteria | c__Gammaproteobacteria | o__Alteromonadales | f__Alteromonadaceae | g__Marinobacter | s__zhejiangensis |
| k__Bacteria | p__Chloroflexi | c__Chloroflexi | o__Chloroflexales | f__FFCH7168 | g__ | s__ |
| k__Bacteria | p__Planctomycetes | c__Planctomycetia | o__Pirellulales | f__Pirellulaceae | __ | __ |
| k__Bacteria | p__Actinobacteria | c__Actinobacteria | o__Actinomycetales | f__Streptosporangiaceae | g__Planomonospora | s__venezuelensis |
| k__Bacteria | p__Proteobacteria | c__Gammaproteobacteria | o__Alteromonadales | f__Alteromonadaceae | g__Marinobacter | __ |
| k__Bacteria | p__Firmicutes | c__Bacilli | o__Bacillales | f__Planococcaceae | g__Staphylococcus | s__saprophyticus |
| k__Bacteria | p__Proteobacteria | c__Alphaproteobacteria | o__Rhizobiales | f__Bradyrhizobiaceae | __ | __ |
| k__Bacteria | p__OP11 | c__WCHB1-64 | o__d153 | f__ | g__ | s__ |
| k__Bacteria | p__Proteobacteria | c__Gammaproteobacteria | o__Chromatiales | __ | __ | __ |
| k__Bacteria | p__Bacteroidetes | c__Flavobacteriia | o__Flavobacteriales | f__Flavobacteriaceae | g__Flavobacterium | s__gelidilacus |
| k__Bacteria | p__OP3 | c__koll11 | o__ | f__ | g__ | s__ |
| k__Bacteria | p__Chloroflexi | c__TK10 | o__AKYG885 | f__Dolo_23 | g__ | s__ |
| k__Bacteria | p__Proteobacteria | c__Deltaproteobacteria | o__Syntrophobacterales | f__Syntrophaceae | g__Smithella | s__ |
| k__Bacteria | p__TM7 | c__TM7-3 | o__Blgi18 | f__ | g__ | s__ |
| k__Bacteria | p__Planctomycetes | c__Phycisphaerae | o__Phycisphaerales | f__ | g__ | s__ |
| k__Bacteria | p__Gemmatimonadetes | c__Gemmatimonadetes | o__KD8-87 | f__ | g__ | s__ |
| k__Bacteria | p__Kazan-3B-28 | c__ | o__ | f__ | g__ | s__ |
| k__Bacteria | p__Chloroflexi | c__Chloroflexi | o__AKIW781 | f__ | g__ | s__ |
| k__Bacteria | p__Proteobacteria | c__Alphaproteobacteria | o__Sphingomonadales | f__Sphingomonadaceae | g__Sphingobium | s__estrogenivorans |
| k__Bacteria | p__Gemmatimonadetes | c__Gemmatimonadetes | __ | __ | __ | __ |
| k__Bacteria | p__Verrucomicrobia | __ | __ | __ | __ | __ |
| k__Bacteria | p__Proteobacteria | c__Deltaproteobacteria | o__Myxococcales | f__Cystobacteraceae | g__Cystobacter | __ |
| k__Bacteria | p__Planctomycetes | c__Phycisphaerae | o__Phycisphaerales | f__Phycisphaeraceae | g__Phycisphaera | s__mikurensis |
| k__Bacteria | p__Bacteroidetes | c__Flavobacteriia | o__Flavobacteriales | f__Flavobacteriaceae | g__Flavobacterium | s__frigidarium |
| k__Bacteria | p__Verrucomicrobia | c__[Spartobacteria] | o__[Chthoniobacterales] | f__[Chthoniobacteraceae] | g__ | s__ |
| k__Bacteria | p__Bacteroidetes | c__Bacteroidia | o__Bacteroidales | f__Bacteroidaceae | g__Bacteroides | s__ |
| k__Bacteria | p__Planctomycetes | c__Planctomycetia | o__Pirellulales | f__Pirellulaceae | g__Pirellula | s__ |
| k__Bacteria | p__Proteobacteria | c__Deltaproteobacteria | o__Myxococcales | f__Cystobacteraceae | g__Cystobacter | s__fuscus |
| k__Bacteria | p__Proteobacteria | c__Deltaproteobacteria | o__Myxococcales | f__Polyangiaceae | g__Polyangium | __ |
| k__Bacteria | p__Planctomycetes | c__Planctomycetia | o__Gemmatales | f__Isosphaeraceae | g__Nostocoida | s__limicola III |
| k__Bacteria | p__Bacteroidetes | c__Bacteroidia | o__Bacteroidales | __ | __ | __ |
| k__Bacteria | p__Chlamydiae | c__Chlamydiia | o__Chlamydiales | __ | __ | __ |
| k__Bacteria | p__Chloroflexi | c__C0119 | o__ | f__ | g__ | s__ |
| k__Bacteria | p__Actinobacteria | c__Actinobacteria | o__Actinomycetales | f__Streptomycetaceae | __ | __ |
| k__Bacteria | p__Actinobacteria | c__Actinobacteria | o__Actinomycetales | f__Glycomycetaceae | g__Glycomyces | s__harbinensis |
| k__Bacteria | p__Proteobacteria | c__Alphaproteobacteria | o__Rhodospirillales | f__Rhodospirillaceae | g__Reyranella | s__massiliensis |
| k__Bacteria | p__Bacteroidetes | c__Cytophagia | o__Cytophagales | f__Cytophagaceae | g__Rhodocytophaga | s__ |
| k__Bacteria | p__Proteobacteria | c__Alphaproteobacteria | o__Rhizobiales | f__Phyllobacteriaceae | g__Thermovum | s__composti |
| k__Bacteria | p__Proteobacteria | c__Alphaproteobacteria | o__Rhodospirillales | f__Rhodospirillaceae | g__Constrictibacter | s__antarcticus |
| k__Bacteria | p__Bacteroidetes | c__Bacteroidia | o__Bacteroidales | f__Bacteroidaceae | g__Bacteroides | s__plebeius |
| k__Bacteria | p__Verrucomicrobia | c__Verrucomicrobiae | o__Verrucomicrobiales | f__Verrucomicrobiaceae | __ | __ |
| k__Bacteria | p__Proteobacteria | c__Alphaproteobacteria | o__Rhizobiales | f__Rhizobiaceae | g__Rhizobium | s__daejeonense |
| k__Bacteria | p__Proteobacteria | c__Gammaproteobacteria | o__Xanthomonadales | f__Sinobacteraceae | g__Solimonas | s__soli |
| k__Bacteria | p__Bacteroidetes | c__[Saprospirae] | o__[Saprospirales] | f__Saprospiraceae | __ | __ |
| k__Bacteria | p__Actinobacteria | c__Actinobacteria | o__Actinomycetales | f__Dietziaceae | __ | __ |
| k__Bacteria | p__Firmicutes | c__Bacilli | o__Lactobacillales | f__Streptococcaceae | g__Streptococcus | s__equi |
| k__Bacteria | p__Firmicutes | c__Clostridia | o__Clostridiales | __ | __ | __ |
| k__Bacteria | p__Actinobacteria | c__Actinobacteria | o__Actinomycetales | f__Nocardiaceae | __ | __ |
| k__Bacteria | p__Chloroflexi | c__Chloroflexi | o__Herpetosiphonales | f__ | g__ | s__ |
| k__Bacteria | p__Bacteroidetes | c__[Saprospirae] | o__[Saprospirales] | f__Saprospiraceae | g__ | s__ |
| k__Bacteria | p__Proteobacteria | c__Alphaproteobacteria | o__Rhodospirillales | f__Rhodospirillaceae | g__Stella | __ |
| k__Bacteria | p__Bacteroidetes | c__Bacteroidia | o__Bacteroidales | f__Bacteroidaceae | g__Bacteroides | __ |
| k__Bacteria | p__Planctomycetes | c__Planctomycetia | o__Pirellulales | f__Pirellulaceae | g__planctomycete | s__MS3071 |
| k__Bacteria | p__Proteobacteria | c__Alphaproteobacteria | o__Rhodospirillales | f__Acetobacteraceae | g__Roseomonas | __ |
| k__Bacteria | p__TM7 | __ | __ | __ | __ | __ |
| k__Bacteria | p__Bacteroidetes | c__Sphingobacteriia | o__Sphingobacteriales | f__Sphingobacteriaceae | g__Parapedobacter | s__soli |
| k__Bacteria | p__Actinobacteria | c__Actinobacteria | o__Actinomycetales | f__Glycomycetaceae | g__Haloglycomyces | s__albus |
| k__Bacteria | p__Actinobacteria | c__Actinobacteria | o__Actinomycetales | f__Geodermatophilaceae | g__Blastococcus | __ |
| k__Bacteria | p__Chlamydiae | c__Chlamydiia | o__Chlamydiales | f__Simkaniaceae | g__Simkania | s__negevensis Z |
| k__Bacteria | p__Proteobacteria | c__Deltaproteobacteria | o__Myxococcales | f__OM27 | g__ | s__ |
| k__Bacteria | p__Gemmatimonadetes | c__Gemm-2 | o__ | f__ | g__ | s__ |
| k__Bacteria | p__Chloroflexi | c__Anaerolineae | o__envOPS12 | f__ | g__ | s__ |
| k__Bacteria | p__Chlamydiae | c__Chlamydiia | o__Chlamydiales | f__Simkaniaceae | g__Simkania | __ |
| k__Bacteria | p__Proteobacteria | c__Betaproteobacteria | o__Burkholderiales | f__Alcaligenaceae | __ | __ |
| k__Bacteria | p__Firmicutes | c__Bacilli | o__Bacillales | f__Bacillaceae | g__Bacillus | s__koreensis |
| k__Bacteria | p__Proteobacteria | c__Deltaproteobacteria | o__Myxococcales | f__Nannocystaceae | g__Nannocystis | s__exedens |
| k__Bacteria | p__Proteobacteria | c__Gammaproteobacteria | o__Pseudomonadales | f__Moraxellaceae | g__Alkanindiges | s__illinoisensis |
| k__Bacteria | p__Bacteroidetes | c__[Saprospirae] | o__[Saprospirales] | f__Chitinophagaceae | g__Terrimonas | s__ferruginea |
| k__Bacteria | p__Planctomycetes | c__Phycisphaerae | o__Phycisphaerales | __ | __ | __ |
| k__Bacteria | p__Acidobacteria | __ | __ | __ | __ | __ |
| k__Bacteria | p__[Thermi] | c__Deinococci | o__Deinococcales | f__Deinococcaceae | g__R18-435 | s__ |
| k__Bacteria | p__Actinobacteria | c__Thermoleophilia | o__Solirubrobacterales | f__Solirubrobacteraceae | g__Solirubrobacter | s__ |
| k__Bacteria | p__Proteobacteria | c__Gammaproteobacteria | o__Alteromonadales | f__Alteromonadaceae | g__Glaciecola | __ |
| k__Bacteria | p__Proteobacteria | c__Gammaproteobacteria | o__Xanthomonadales | f__Sinobacteraceae | __ | __ |
| k__Bacteria | p__Cyanobacteria | __ | __ | __ | __ | __ |
| k__Bacteria | p__GN02 | c__GN07 | o__ | f__ | g__ | s__ |
| k__Bacteria | p__Actinobacteria | c__Nitriliruptoria | o__Nitriliruptorales | f__Nitriliruptoraceae | g__Nitriliruptor | s__alkaliphilus |
| k__Bacteria | p__FBP | c__ | o__ | f__ | g__ | s__ |
| k__Bacteria | p__Chloroflexi | c__Anaerolineae | o__Ardenscatenales | f__Ardenscatenaceae | g__Ardenscatena | s__ |
| k__Bacteria | p__Firmicutes | c__Clostridia | __ | __ | __ | __ |
| k__Bacteria | p__Gemmatimonadetes | c__ | o__ | f__ | g__ | s__ |
| k__Bacteria | p__Firmicutes | c__Bacilli | o__Bacillales | f__Thermoactinomycetaceae | g__Laceyella | s__ |
| k__Bacteria | p__Chloroflexi | c__Chloroflexi | __ | __ | __ | __ |
| k__Bacteria | p__Proteobacteria | c__Deltaproteobacteria | o__Myxococcales | f__ | g__ | s__ |
| k__Bacteria | p__Proteobacteria | c__Deltaproteobacteria | o__Desulfuromonadales | f__Pelobacteraceae | g__Desulfuromonas | s__michiganensis |
| k__Bacteria | p__Actinobacteria | c__Actinobacteria | o__Actinomycetales | f__Geodermatophilaceae | g__Blastococcus | s__aggregatus |
| k__Bacteria | p__Proteobacteria | c__Gammaproteobacteria | o__Legionellales | f__Legionellaceae | __ | __ |
| k__Bacteria | p__Chloroflexi | c__Chloroflexi | o__[Roseiflexales] | f__[Roseiflexaceae] | g__ | s__ |
| k__Bacteria | p__Bacteroidetes | c__Cytophagia | o__Cytophagales | f__Cyclobacteriaceae | g__Echinicola | s__shivajiensis |
| k__Bacteria | p__Chloroflexi | c__Anaerolineae | o__H39 | f__ | g__ | s__ |
| k__Bacteria | p__Planctomycetes | c__Planctomycetia | o__Pirellulales | f__Pirellulaceae | g__planctomycete | s__MS1399 |
| k__Bacteria | p__Actinobacteria | c__Actinobacteria | o__Actinomycetales | f__Micromonosporaceae | g__Micromonospora | __ |
| k__Bacteria | p__Bacteroidetes | c__Bacteroidia | o__Bacteroidales | f__Rikenellaceae | g__Alistipes | s__finegoldii |
| k__Bacteria | p__Actinobacteria | c__Actinobacteria | o__Actinomycetales | f__Propionibacteriaceae | g__Microlunatus | __ |
| k__Bacteria | p__Proteobacteria | c__Alphaproteobacteria | o__Sphingomonadales | f__Erythrobacteraceae | g__Erythrobacter | __ |
| k__Bacteria | p__Firmicutes | c__Clostridia | o__Clostridiales | f__Ruminococcaceae | g__Clostridium | s__cellobioparum |
| k__Bacteria | p__Planctomycetes | c__Planctomycetia | o__Gemmatales | f__Gemmataceae | g__ | s__ |
| k__Bacteria | p__Chlamydiae | c__Chlamydiia | o__Chlamydiales | f__Parachlamydiaceae | __ | __ |
| k__Bacteria | p__Proteobacteria | c__Deltaproteobacteria | o__Desulfovibrionales | f__Desulfovibrionaceae | g__Desulfovibrio | __ |
| k__Bacteria | p__Proteobacteria | c__Alphaproteobacteria | o__Sphingomonadales | f__Sphingomonadaceae | g__Novosphingobium | s__nitrogenifigens |
| k__Bacteria | p__Firmicutes | c__Clostridia | o__Clostridiales | f__Lachnospiraceae | g__Ruminococcus | s__lactaris |
| k__Bacteria | p__Proteobacteria | c__Gammaproteobacteria | o__Pseudomonadales | f__Moraxellaceae | __ | __ |
| k__Bacteria | p__Chloroflexi | c__TK10 | o__B07_WMSP1 | f__ | g__ | s__ |
| k__Bacteria | p__OP11 | c__WCHB1-64 | o__ | f__ | g__ | s__ |
| k__Bacteria | p__Verrucomicrobia | c__[Spartobacteria] | o__[Chthoniobacterales] | f__[Chthoniobacteraceae] | g__OR-59 | s__ |
| k__Bacteria | p__Actinobacteria | c__Thermoleophilia | o__Gaiellales | f__AK1AB1_02E | g__ | s__ |
| k__Bacteria | p__Proteobacteria | c__Gammaproteobacteria | o__Legionellales | f__Legionellaceae | g__Legionella | s__adelaidensis |
| k__Bacteria | p__Firmicutes | c__Bacilli | o__Bacillales | f__Bacillaceae | g__Bacillus | s__alkalinitrilicus |
| k__Bacteria | p__Proteobacteria | c__Betaproteobacteria | o__Burkholderiales | f__Alcaligenaceae | g__Pigmentiphaga | s__daeguensis |
| k__Bacteria | p__Chloroflexi | __ | __ | __ | __ | __ |
| k__Bacteria | p__Verrucomicrobia | c__Opitutae | o__Opitutales | f__Opitutaceae | g__Opitutus | s__ |
| k__Bacteria | p__Verrucomicrobia | c__[Pedosphaerae] | o__[Pedosphaerales] | f__OPB35 | g__ | s__ |
| k__Bacteria | p__Bacteroidetes | c__[Rhodothermi] | o__[Rhodothermales] | f__Rhodothermaceae | g__Rubricoccus | s__ |
| k__Bacteria | p__Proteobacteria | c__Betaproteobacteria | o__Burkholderiales | f__Oxalobacteraceae | g__Massilia | s__dura |
| k__Bacteria | p__Bacteroidetes | c__Flavobacteriia | o__Flavobacteriales | f__Flavobacteriaceae | g__Flavobacterium | __ |
| k__Bacteria | p__Firmicutes | c__Clostridia | o__Halanaerobiales | f__Halanaerobiaceae | g__Halocella | s__cellulolsilytica |
| k__Bacteria | p__Bacteroidetes | c__Sphingobacteriia | o__Sphingobacteriales | f__Sphingobacteriaceae | __ | __ |
| k__Bacteria | p__Chloroflexi | c__Chloroflexi | o__Chloroflexales | __ | __ | __ |
| k__Bacteria | p__[Thermi] | c__Deinococci | __ | __ | __ | __ |
| k__Bacteria | p__TM6 | c__SJA-4 | o__ | f__ | g__ | s__ |
| k__Bacteria | p__Firmicutes | c__Bacilli | o__Bacillales | f__Thermoactinomycetaceae | g__Thermoactinomyces | s__ |
| k__Bacteria | p__TM6 | __ | __ | __ | __ | __ |
| k__Bacteria | p__Cyanobacteria | c__4C0d-2 | o__SM1D11 | f__ | g__ | s__ |
| k__Bacteria | p__Bacteroidetes | c__Sphingobacteriia | o__Sphingobacteriales | f__Sphingobacteriaceae | g__Pedobacter | s__xinjiangensis |
| k__Bacteria | p__Actinobacteria | c__Actinobacteria | o__Actinomycetales | f__Actinosynnemataceae | g__Umezawaea | s__tangerina |
| k__Bacteria | p__Planctomycetes | c__Phycisphaerae | __ | __ | __ | __ |
| k__Bacteria | p__Proteobacteria | c__Betaproteobacteria | o__Neisseriales | f__Neisseriaceae | g__Gulbenkiania | s__mobilis |
| k__Bacteria | p__Gemmatimonadetes | c__Gemm-5 | o__ | f__ | g__ | s__ |
| k__Bacteria | p__Bacteroidetes | c__Bacteroidia | o__Bacteroidales | f__Rikenellaceae | g__Alistipes | s__onderdonkii |
| k__Bacteria | p__Bacteroidetes | c__Cytophagia | o__Cytophagales | f__Cyclobacteriaceae | g__Algoriphagus | s__aquatilis |
