# Supplementary material for: Metagenomic insight into drought-induced changes in the Egyptian wheat rhizosphere microbiome
Source: World J Microbiol Biotechnol. 2025 Aug 12;41(8):310. doi: 10.1007/s11274-025-04518-0 (PMC12343654; doi:10.1007/s11274-025-04518-0)
Supplement: Supplementary file 2 — Supplementary Material 2 [file 11274_2025_4518_MOESM2_ESM.docx]

**Table (S2)**: Kyoto Encyclopedia of Genes and Genomes (KEGG) orthology, predicted by Phylogenetic Investigation of Communities by Reconstruction of Unobserved States (PICRUSt).

#NAME 01Dic-C1 02Dic-C2 03Dic-C3 04Dic-D1 05Dic-D2 06Dic-D3 07Giza 168-C1 08Giza 168-C2 09Giza 168-C3 10Giza 168-D1 11Giza 168-D2 12Giza 168-D3 13Gem9-C1 14Gem9-C2 15Gem9-C3 16Gem9-D1 17Gem9-D2 18Gem9-D3 19Gem11-C1 20Gem11-C2 21Gem11-C3 22Gem11-D1 23Gem11-D2 24Gem11-D3 25M1-C1 26M1-C2 27M1-C3 28M1-D1 29M1-D2 30M1-D3 31Mono-C1 32Mono-C2 33Mono-C3 34Mono-D1 35Mono-D2 36Mono-D3 37Sids13-C1 38Sids13-C2 39Sids13-C3 40Sids13-D1 41Sids13-D2 42Sids13-D3 43Shd1-C1 44Shd1-C2 45Shd1-C3 46Shd1-D1 47Shd1-D2 48Shd1-D3

K00001 0 0 0 0 0 0 0 0 0 0 0 0 0 0 0 0 0 0 0 0 0 0 0 0 0 0 0 0 0 0 0 0 0 0 0 0 0 0 0 0 0 0 0 0 0 0 0 0

K00002 52 40 107 47 252 200 64 338 84 124 192 86 28 72 108 27 101 208 59 44 106 150 124 172 12 30 21 9 38 274 0 50 50 64 83 77 37 54 90 122 168 145 28 103 96 144 59 230

K00003 451 524 486 214 826 650 348 1074 662 698 917 814 296 794 576 275 685 1173 584 636 853 970 1102 868 388 368 378 290 342 963 24 874 580 824 912 798 249 524 620 511 750 748 430 833 780 533 604 891

K00004 0 0 0 0 0 0 0 0 0 0 0 0 0 0 0 0 0 0 0 0 0 0 0 0 0 0 0 0 0 0 0 0 0 0 0 0 0 0 0 0 0 0 0 0 0 0 0 0

K00005 0 0 0 0 0 0 0 0 0 0 0 0 0 0 0 0 0 0 0 0 0 0 0 0 0 0 0 0 0 0 0 0 0 0 0 0 0 0 0 0 0 0 0 0 0 0 0 0

K00007 0 0 0 0 0 0 0 0 0 0 0 0 0 0 0 0 0 0 0 0 0 0 0 0 0 0 0 0 0 0 0 0 0 0 0 0 0 0 0 0 0 0 0 0 0 0 0 0

K00008 0 0 0 0 0 0 0 0 0 0 0 0 0 0 0 0 0 0 0 0 0 0 0 0 0 0 0 0 0 0 0 0 0 0 0 0 0 0 0 0 0 0 0 0 0 0 0 0

K00009 0 0 0 0 0 0 0 0 0 0 0 0 0 0 0 0 0 0 0 0 0 0 0 0 0 0 0 0 0 0 0 0 0 0 0 0 0 0 0 0 0 0 0 0 0 0 0 0

K00010 0 0 0 0 0 0 0 0 0 0 0 0 0 0 0 0 0 0 0 0 0 0 0 0 0 0 0 0 0 0 0 0 0 0 0 0 0 0 0 0 0 0 0 0 0 0 0 0

K00011 0 0 0 0 0 0 0 0 0 0 0 0 0 0 0 0 0 0 0 0 0 0 0 0 0 0 0 0 0 0 0 0 0 0 0 0 0 0 0 0 0 0 0 0 0 0 0 0

K00012 456 542 559 246 1040 820 382 1382 702 797 1070 859 314 832 622 296 774 1354 632 614 918 1104 1186 978 390 394 392 290 362 1185 24 876 608 817 952 825 278 548 686 617 903 836 430 912 851 673 647 1064

K00013 437 524 486 214 819 650 340 1074 662 698 917 814 296 794 560 275 685 1173 584 618 853 970 1102 855 388 368 378 290 342 963 24 874 580 799 912 798 249 524 620 511 750 740 430 833 780 533 604 891

K00014 437 524 486 214 795 634 340 1066 662 698 914 814 296 794 560 275 685 1159 584 618 853 970 1102 832 388 368 378 290 342 946 24 844 580 799 912 798 249 524 620 511 750 740 430 833 780 533 604 891

K00015 42 200 159 49 158 147 64 144 106 120 225 154 91 132 160 46 234 210 164 205 234 156 234 208 84 102 94 19 116 243 0 164 68 67 182 137 58 236 295 157 201 244 80 337 382 125 184 244

K00016 0 0 0 0 0 0 0 0 0 0 0 0 0 0 0 0 0 0 0 0 0 0 0 0 0 0 0 0 0 0 0 0 0 0 0 0 0 0 0 0 0 0 0 0 0 0 0 0

K00018 42 200 159 49 158 147 64 144 106 120 225 154 91 132 160 46 234 210 164 205 234 156 234 208 84 102 94 19 116 243 0 164 68 67 182 137 58 236 295 157 201 244 80 337 382 125 184 244

K00019 0 0 0 0 0 0 0 0 0 0 0 0 0 0 0 0 0 0 0 0 0 0 0 0 0 0 0 0 0 0 0 0 0 0 0 0 0 0 0 0 0 0 0 0 0 0 0 0

K00020 338 420 344 166 406 320 267 564 552 574 661 728 268 601 372 202 508 856 526 476 648 784 838 582 354 338 346 218 292 502 24 648 436 638 772 654 212 438 429 340 452 498 327 580 572 350 470 533

K00021 99 104 142 47 414 330 72 511 110 124 256 86 28 194 188 74 176 317 59 142 204 186 263 274 35 30 32 71 49 461 0 226 144 161 140 143 37 85 192 171 298 242 103 254 208 184 134 358

K00022 0 0 0 0 0 0 0 0 0 0 0 0 0 0 0 0 0 0 0 0 0 0 0 0 0 0 0 0 0 0 0 0 0 0 0 0 0 0 0 0 0 0 0 0 0 0 0 0

K00023 0 0 0 0 0 0 0 0 0 0 0 0 0 0 0 0 0 0 0 0 0 0 0 0 0 0 0 0 0 0 0 0 0 0 0 0 0 0 0 0 0 0 0 0 0 0 0 0

K00024 404 460 451 214 712 552 340 920 636 698 858 814 296 673 496 228 610 1092 584 538 754 935 962 813 365 368 367 228 330 811 24 758 486 726 856 732 249 493 518 462 620 652 355 682 668 493 530 764

K00025 0 0 0 0 0 0 0 0 0 0 0 0 0 0 0 0 0 0 0 0 0 0 0 0 0 0 0 0 0 0 0 0 0 0 0 0 0 0 0 0 0 0 0 0 0 0 0 0

K00026 0 0 0 0 0 0 0 0 0 0 0 0 0 0 0 0 0 0 0 0 0 0 0 0 0 0 0 0 0 0 0 0 0 0 0 0 0 0 0 0 0 0 0 0 0 0 0 0

K00027 42 200 159 49 160 147 64 144 106 120 228 154 91 132 160 46 234 210 164 205 234 156 234 208 84 102 94 19 116 243 0 164 68 67 182 137 58 236 295 157 201 244 80 337 382 125 184 244

K00028 0 0 0 0 0 0 0 0 0 0 0 0 0 0 0 0 0 0 0 0 0 0 0 0 0 0 0 0 0 0 0 0 0 0 0 0 0 0 0 0 0 0 0 0 0 0 0 0

K00029 0 0 0 0 0 0 0 0 0 0 0 0 0 0 0 0 0 0 0 0 0 0 0 0 0 0 0 0 0 0 0 0 0 0 0 0 0 0 0 0 0 0 0 0 0 0 0 0

K00030 42 200 159 49 158 147 64 144 106 120 225 154 91 132 160 46 234 210 164 205 234 156 234 208 84 102 94 19 116 243 0 164 68 67 182 137 58 236 295 157 201 244 80 337 382 125 184 244

K00031 338 420 344 166 406 320 267 564 552 574 661 728 268 601 372 202 508 856 526 476 648 784 838 582 354 338 346 218 292 502 24 648 436 638 772 654 212 438 429 340 452 498 327 580 572 350 470 533

K00032 0 0 0 0 0 0 0 0 0 0 0 0 0 0 0 0 0 0 0 0 0 0 0 0 0 0 0 0 0 0 0 0 0 0 0 0 0 0 0 0 0 0 0 0 0 0 0 0

K00033 0 0 0 0 0 0 0 0 0 0 0 0 0 0 0 0 0 0 0 0 0 0 0 0 0 0 0 0 0 0 0 0 0 0 0 0 0 0 0 0 0 0 0 0 0 0 0 0

K00034 0 0 0 0 23 16 0 9 0 0 0 0 0 0 0 0 0 14 0 0 0 0 0 6 0 0 0 0 0 13 0 0 0 0 0 0 0 0 0 0 0 0 0 0 0 0 0 0

K00035 0 0 0 0 0 0 0 0 0 0 0 0 0 0 0 0 0 0 0 0 0 0 0 0 0 0 0 0 0 0 0 0 0 0 0 0 0 0 0 0 0 0 0 0 0 0 0 0

K00036 0 0 0 0 0 0 0 0 0 0 0 0 0 0 0 0 0 0 0 0 0 0 0 0 0 0 0 0 0 0 0 0 0 0 0 0 0 0 0 0 0 0 0 0 0 0 0 0

K00037 0 0 0 0 0 0 0 0 0 0 0 0 0 0 0 0 0 0 0 0 0 0 0 0 0 0 0 0 0 0 0 0 0 0 0 0 0 0 0 0 0 0 0 0 0 0 0 0

K00038 0 0 0 0 1 0 0 0 0 0 0 0 0 0 0 0 0 0 0 0 0 0 0 0 0 0 0 0 0 0 0 0 0 0 0 0 0 0 0 0 0 0 0 0 0 0 0 0

K00039 0 0 0 0 0 0 0 0 0 0 0 0 0 0 0 0 0 0 0 0 0 0 0 0 0 0 0 0 0 0 0 0 0 0 0 0 0 0 0 0 0 0 0 0 0 0 0 0

K00040 0 0 0 0 0 0 0 0 0 0 0 0 0 0 0 0 0 0 0 0 0 0 0 0 0 0 0 0 0 0 0 0 0 0 0 0 0 0 0 0 0 0 0 0 0 0 0 0

K00041 0 0 0 0 0 0 0 0 0 0 0 0 0 0 0 0 0 0 0 0 0 0 0 0 0 0 0 0 0 0 0 0 0 0 0 0 0 0 0 0 0 0 0 0 0 0 0 0

K00042 0 0 0 0 0 0 0 0 0 0 0 0 0 0 0 0 0 0 0 0 0 0 0 0 0 0 0 0 0 0 0 0 0 0 0 0 0 0 0 0 0 0 0 0 0 0 0 0

K00043 0 0 0 0 0 0 0 0 0 0 0 0 0 0 0 0 0 0 0 0 0 0 0 0 0 0 0 0 0 0 0 0 0 0 0 0 0 0 0 0 0 0 0 0 0 0 0 0

K00044 0 0 0 0 0 0 0 0 0 0 0 0 0 0 0 0 0 0 0 0 0 0 0 0 0 0 0 0 0 0 0 0 0 0 0 0 0 0 0 0 0 0 0 0 0 0 0 0

K00045 0 0 0 0 0 0 0 0 0 0 0 0 0 0 0 0 0 0 0 0 0 0 0 0 0 0 0 0 0 0 0 0 0 0 0 0 0 0 0 0 0 0 0 0 0 0 0 0

K00046 33 18 73 32 205 154 52 298 40 98 154 45 18 37 78 20 89 167 48 14 66 133 84 130 1 26 13 0 21 209 0 5 28 42 40 28 28 24 66 106 152 105 0 78 72 140 43 173

K00046.1 33 18 73 32 205 154 52 298 40 98 154 45 18 37 78 20 89 167 48 14 66 133 84 130 1 26 13 0 21 209 0 5 28 42 40 28 28 24 66 106 152 105 0 78 72 140 43 173

K00048 0 0 0 0 0 0 0 0 0 0 0 0 0 0 0 0 0 0 0 0 0 0 0 0 0 0 0 0 0 0 0 0 0 0 0 0 0 0 0 0 0 0 0 0 0 0 0 0

K00050 42 200 159 49 158 147 64 144 106 120 225 154 91 132 160 46 234 210 164 205 234 156 234 208 84 102 94 19 116 243 0 164 68 67 182 137 58 236 295 157 201 244 80 337 382 125 184 244

K00051 0 0 0 0 0 0 0 0 0 0 0 0 0 0 0 0 0 0 0 0 0 0 0 0 0 0 0 0 0 0 0 0 0 0 0 0 0 0 0 0 0 0 0 0 0 0 0 0

K00052 545 764 752 310 1260 1013 476 1566 852 942 1336 1054 414 998 842 348 1020 1604 808 885 1193 1278 1458 1272 484 501 493 318 496 1498 24 1119 699 954 1177 1012 344 814 1005 790 1120 1138 538 1273 1257 802 847 1365

K00053 437 524 486 214 819 650 340 1074 662 698 917 814 296 794 560 275 685 1173 584 618 853 970 1102 855 388 368 378 290 342 963 24 874 580 799 912 798 249 524 620 511 750 740 430 833 780 533 604 891

K00054 42 200 159 49 158 147 64 144 106 120 225 154 91 132 160 46 234 210 164 205 234 156 234 208 84 102 94 19 116 243 0 164 68 67 182 137 58 236 295 157 201 244 80 337 382 125 184 244

K00055 0 0 0 0 0 0 0 0 0 0 0 0 0 0 0 0 0 0 0 0 0 0 0 0 0 0 0 0 0 0 0 0 0 0 0 0 0 0 0 0 0 0 0 0 0 0 0 0

K00057 371 438 417 198 640 490 327 871 592 672 814 774 286 638 466 222 598 1037 573 490 714 918 922 731 354 364 359 218 314 724 24 652 464 680 813 682 240 463 494 446 604 612 327 658 643 490 514 706

K00058 478 724 645 262 978 798 404 1219 768 818 1142 968 386 926 719 320 919 1382 748 822 1088 1127 1335 1064 473 471 472 308 458 1206 24 1038 648 866 1094 934 308 759 916 668 952 984 510 1170 1161 658 788 1134

K00059 940 1786 1467 561 1913 1612 842 2330 1618 1724 2500 2118 918 2008 1588 698 2193 2907 1754 1944 2498 2399 2974 2257 1093 1112 1103 637 1094 2524 48 2250 1360 1732 2426 2016 686 1904 2308 1512 2122 2254 1122 2886 2964 1419 1869 2468

K00060 0 0 0 0 0 0 0 0 0 0 0 0 0 0 0 0 0 0 0 0 0 0 0 0 0 0 0 0 0 0 0 0 0 0 0 0 0 0 0 0 0 0 0 0 0 0 0 0

K00061 0 0 0 0 0 0 0 0 0 0 0 0 0 0 0 0 0 0 0 0 0 0 0 0 0 0 0 0 0 0 0 0 0 0 0 0 0 0 0 0 0 0 0 0 0 0 0 0

K00064 0 0 0 0 0 0 0 0 0 0 0 0 0 0 0 0 0 0 0 0 0 0 0 0 0 0 0 0 0 0 0 0 0 0 0 0 0 0 0 0 0 0 0 0 0 0 0 0

K00065 0 0 0 0 0 0 0 0 0 0 0 0 0 0 0 0 0 0 0 0 0 0 0 0 0 0 0 0 0 0 0 0 0 0 0 0 0 0 0 0 0 0 0 0 0 0 0 0

K00066 0 0 0 0 0 0 0 0 0 0 0 0 0 0 0 0 0 0 0 0 0 0 0 0 0 0 0 0 0 0 0 0 0 0 0 0 0 0 0 0 0 0 0 0 0 0 0 0

K00067 404 460 451 214 688 536 340 911 636 698 856 814 296 673 496 228 610 1078 584 538 754 935 962 790 365 368 367 228 330 794 24 724 486 726 856 732 249 493 518 462 620 652 355 682 668 493 530 764

K00068 0 0 0 0 0 0 0 0 0 0 0 0 0 0 0 0 0 0 0 0 0 0 0 0 0 0 0 0 0 0 0 0 0 0 0 0 0 0 0 0 0 0 0 0 0 0 0 0

K00069 0 0 0 0 0 0 0 0 0 0 0 0 0 0 0 0 0 0 0 0 0 0 0 0 0 0 0 0 0 0 0 0 0 0 0 0 0 0 0 0 0 0 0 0 0 0 0 0

K00071 0 0 0 0 0 0 0 0 0 0 0 0 0 0 0 0 0 0 0 0 0 0 0 0 0 0 0 0 0 0 0 0 0 0 0 0 0 0 0 0 0 0 0 0 0 0 0 0

K00073 0 0 0 0 0 0 0 0 0 0 0 0 0 0 0 0 0 0 0 0 0 0 0 0 0 0 0 0 0 0 0 0 0 0 0 0 0 0 0 0 0 0 0 0 0 0 0 0

K00074 432 548 506 239 742 618 370 965 676 754 1040 864 351 774 542 274 708 1180 725 632 894 1067 1182 928 428 471 450 246 418 888 24 787 502 746 924 835 308 698 688 497 646 730 373 810 866 579 608 952

K00075 338 420 344 166 406 320 267 564 552 574 661 728 268 601 372 202 508 856 526 476 648 784 838 582 354 338 346 218 292 502 24 648 436 638 772 654 212 438 429 340 452 498 327 580 572 350 470 533

K00076 0 0 0 0 0 0 0 0 0 0 0 0 0 0 0 0 0 0 0 0 0 0 0 0 0 0 0 0 0 0 0 0 0 0 0 0 0 0 0 0 0 0 0 0 0 0 0 0

K00077 432 660 610 262 863 699 395 1064 741 818 1081 968 386 804 639 274 844 1302 748 724 988 1092 1196 991 450 471 460 246 447 1050 24 896 554 768 1038 868 308 728 814 619 821 888 434 1020 1049 618 714 1007

K00078 0 0 0 0 0 0 0 0 0 0 0 0 0 0 0 0 0 0 0 0 0 0 0 0 0 0 0 0 0 0 0 0 0 0 0 0 0 0 0 0 0 0 0 0 0 0 0 0

K00079 0 0 0 0 0 0 0 0 0 0 0 0 0 0 0 0 0 0 0 0 0 0 0 0 0 0 0 0 0 0 0 0 0 0 0 0 0 0 0 0 0 0 0 0 0 0 0 0

K00082 0 0 0 0 47 32 0 18 0 0 5 0 0 0 0 0 0 28 0 0 0 0 0 29 0 0 0 0 0 30 0 26 0 0 0 0 0 0 0 0 0 0 0 0 0 0 0 0

K00086 0 0 0 0 0 0 0 0 0 0 0 0 0 0 0 0 0 0 0 0 0 0 0 0 0 0 0 0 0 0 0 0 0 0 0 0 0 0 0 0 0 0 0 0 0 0 0 0

K00087 0 0 0 0 0 0 0 0 0 0 0 0 0 0 0 0 0 0 0 0 0 0 0 0 0 0 0 0 0 0 0 0 0 0 0 0 0 0 0 0 0 0 0 0 0 0 0 0

K00088 545 764 752 310 1260 1013 476 1566 852 942 1336 1054 414 998 842 348 1020 1604 808 885 1193 1278 1458 1272 484 501 493 318 496 1498 24 1119 699 954 1177 1012 344 814 1005 790 1120 1138 538 1273 1257 802 847 1365

K00090 0 0 0 0 0 0 0 0 0 0 0 0 0 0 0 0 0 0 0 0 0 0 0 0 0 0 0 0 0 0 0 0 0 0 0 0 0 0 0 0 0 0 0 0 0 0 0 0

K00091 0 0 0 0 0 0 0 0 0 0 0 0 0 0 0 0 0 0 0 0 0 0 0 0 0 0 0 0 0 0 0 0 0 0 0 0 0 0 0 0 0 0 0 0 0 0 0 0

K00094 0 0 0 0 0 0 0 0 0 0 0 0 0 0 0 0 0 0 0 0 0 0 0 0 0 0 0 0 0 0 0 0 0 0 0 0 0 0 0 0 0 0 0 0 0 0 0 0

K00096 140 304 301 96 572 477 136 656 216 244 481 240 119 325 347 119 410 526 223 347 439 342 496 482 120 132 126 90 166 704 0 391 212 228 322 280 96 320 486 328 500 486 182 590 590 308 318 602

K00097 338 420 344 166 406 320 267 564 552 574 661 728 268 601 372 202 508 856 526 476 648 784 838 582 354 338 346 218 292 502 24 648 436 638 772 654 212 438 429 340 452 498 327 580 572 350 470 533

K00098 0 0 0 0 0 0 0 0 0 0 0 0 0 0 0 0 0 0 0 0 0 0 0 0 0 0 0 0 0 0 0 0 0 0 0 0 0 0 0 0 0 0 0 0 0 0 0 0

K00099 338 420 344 166 406 320 267 564 552 574 661 728 268 601 372 202 508 856 526 476 648 784 838 582 354 338 346 218 292 502 24 648 436 638 772 654 212 438 429 340 452 498 327 580 572 350 470 533

K00100 338 420 344 166 498 384 267 600 552 574 664 728 268 601 372 202 508 912 526 476 648 784 838 622 354 338 346 218 292 558 24 674 436 638 772 654 212 438 429 340 452 498 327 580 572 350 470 533

K00101 0 0 0 0 0 0 0 0 0 0 0 0 0 0 0 0 0 0 0 0 0 0 0 0 0 0 0 0 0 0 0 0 0 0 0 0 0 0 0 0 0 0 0 0 0 0 0 0

K00102 0 0 0 0 0 0 0 0 0 0 0 0 0 0 0 0 0 0 0 0 0 0 0 0 0 0 0 0 0 0 0 0 0 0 0 0 0 0 0 0 0 0 0 0 0 0 0 0

K00103 0 0 0 0 0 0 0 0 0 0 0 0 0 0 0 0 0 0 0 0 0 0 0 0 0 0 0 0 0 0 0 0 0 0 0 0 0 0 0 0 0 0 0 0 0 0 0 0

K00104 338 420 344 166 406 320 267 564 552 574 661 728 268 601 372 202 508 856 526 476 648 784 838 582 354 338 346 218 292 502 24 648 436 638 772 654 212 438 429 340 452 498 327 580 572 350 470 533

K00105 0 0 0 0 0 0 0 0 0 0 0 0 0 0 0 0 0 0 0 0 0 0 0 0 0 0 0 0 0 0 0 0 0 0 0 0 0 0 0 0 0 0 0 0 0 0 0 0

K00106 0 0 0 0 0 0 0 0 0 0 0 0 0 0 0 0 0 0 0 0 0 0 0 0 0 0 0 0 0 0 0 0 0 0 0 0 0 0 0 0 0 0 0 0 0 0 0 0

K00108 0 0 0 0 0 0 0 0 0 0 0 0 0 0 0 0 0 0 0 0 0 0 0 0 0 0 0 0 0 0 0 0 0 0 0 0 0 0 0 0 0 0 0 0 0 0 0 0

K00109 0 0 0 0 0 0 0 0 0 0 0 0 0 0 0 0 0 0 0 0 0 0 0 0 0 0 0 0 0 0 0 0 0 0 0 0 0 0 0 0 0 0 0 0 0 0 0 0

K00111 380 620 503 216 564 468 331 708 657 694 886 882 358 732 532 247 742 1066 690 680 883 941 1072 790 438 441 440 238 409 745 24 812 504 705 954 792 270 674 724 497 653 742 406 916 953 474 654 776

K00112 0 0 0 0 0 0 0 0 0 0 0 0 0 0 0 0 0 0 0 0 0 0 0 0 0 0 0 0 0 0 0 0 0 0 0 0 0 0 0 0 0 0 0 0 0 0 0 0

K00113 0 0 0 0 0 0 0 0 0 0 0 0 0 0 0 0 0 0 0 0 0 0 0 0 0 0 0 0 0 0 0 0 0 0 0 0 0 0 0 0 0 0 0 0 0 0 0 0

K00114 0 0 0 0 0 0 0 0 0 0 0 0 0 0 0 0 0 0 0 0 0 0 0 0 0 0 0 0 0 0 0 0 0 0 0 0 0 0 0 0 0 0 0 0 0 0 0 0

K00115 0 0 0 0 0 0 0 0 0 0 0 0 0 0 0 0 0 0 0 0 0 0 0 0 0 0 0 0 0 0 0 0 0 0 0 0 0 0 0 0 0 0 0 0 0 0 0 0

K00116 0 0 0 0 0 0 0 0 0 0 0 0 0 0 0 0 0 0 0 0 0 0 0 0 0 0 0 0 0 0 0 0 0 0 0 0 0 0 0 0 0 0 0 0 0 0 0 0

K00117 0 0 0 0 0 0 0 0 0 0 0 0 0 0 0 0 0 0 0 0 0 0 0 0 0 0 0 0 0 0 0 0 0 0 0 0 0 0 0 0 0 0 0 0 0 0 0 0

K00118 0 0 0 0 0 0 0 0 0 0 0 0 0 0 0 0 0 0 0 0 0 0 0 0 0 0 0 0 0 0 0 0 0 0 0 0 0 0 0 0 0 0 0 0 0 0 0 0

K00119 0 0 0 0 0 0 0 0 0 0 0 0 0 0 0 0 0 0 0 0 0 0 0 0 0 0 0 0 0 0 0 0 0 0 0 0 0 0 0 0 0 0 0 0 0 0 0 0

K00120 0 0 0 0 0 0 0 0 0 0 0 0 0 0 0 0 0 0 0 0 0 0 0 0 0 0 0 0 0 0 0 0 0 0 0 0 0 0 0 0 0 0 0 0 0 0 0 0

K00121 0 0 0 0 0 0 0 0 0 0 0 0 0 0 0 0 0 0 0 0 0 0 0 0 0 0 0 0 0 0 0 0 0 0 0 0 0 0 0 0 0 0 0 0 0 0 0 0

K00122 42 200 159 49 158 147 64 144 106 120 225 154 91 132 160 46 234 210 164 205 234 156 234 208 84 102 94 19 116 243 0 164 68 67 182 137 58 236 295 157 201 244 80 337 382 125 184 244

K00123 526 742 718 294 1236 984 464 1536 808 916 1301 1013 405 963 814 341 1008 1578 796 856 1153 1260 1419 1236 474 496 485 308 479 1446 24 1070 676 933 1134 962 336 784 981 774 1104 1098 510 1248 1232 798 831 1308

K00124 465 524 486 214 802 634 348 1066 662 698 914 814 296 794 576 275 685 1159 584 654 853 970 1102 846 388 368 378 290 342 946 24 844 580 848 912 798 249 524 620 511 750 748 430 833 780 533 604 891

K00125 119 80 214 94 582 447 136 704 168 249 392 171 56 144 231 54 202 458 118 106 211 301 247 410 23 60 42 18 76 596 0 158 101 152 166 154 74 109 179 244 336 298 56 206 192 287 118 461

K00126 0 0 0 0 0 0 0 0 0 0 0 0 0 0 0 0 0 0 0 0 0 0 0 0 0 0 0 0 0 0 0 0 0 0 0 0 0 0 0 0 0 0 0 0 0 0 0 0

K00127 0 0 0 0 0 0 0 0 0 0 0 0 0 0 0 0 0 0 0 0 0 0 0 0 0 0 0 0 0 0 0 0 0 0 0 0 0 0 0 0 0 0 0 0 0 0 0 0

K00128 380 508 398 192 490 418 306 626 592 630 848 778 323 702 435 247 608 972 666 588 789 916 1058 756 417 441 429 238 380 614 24 736 451 682 840 758 270 644 598 375 478 586 345 706 770 436 548 722

K00129 0 0 0 0 0 0 0 0 0 0 0 0 0 0 0 0 0 0 0 0 0 0 0 0 0 0 0 0 0 0 0 0 0 0 0 0 0 0 0 0 0 0 0 0 0 0 0 0

K00130 0 0 0 0 23 16 0 9 0 0 0 0 0 0 0 0 0 14 0 0 0 0 0 6 0 0 0 0 0 13 0 4 0 0 0 0 0 0 0 0 0 0 0 0 0 0 0 0

K00131 0 0 0 0 0 0 0 0 0 0 0 0 0 0 0 0 0 0 0 0 0 0 0 0 0 0 0 0 0 0 0 0 0 0 0 0 0 0 0 0 0 0 0 0 0 0 0 0

K00132 0 0 0 0 0 0 0 0 0 0 0 0 0 0 0 0 0 0 0 0 0 0 0 0 0 0 0 0 0 0 0 0 0 0 0 0 0 0 0 0 0 0 0 0 0 0 0 0

K00133 437 524 486 214 819 650 340 1074 662 698 917 814 296 794 560 275 685 1173 584 618 853 970 1102 855 388 368 378 290 342 963 24 874 580 799 912 798 249 524 620 511 750 740 430 833 780 533 604 891

K00134 338 420 344 166 406 320 267 564 552 574 661 728 268 601 372 202 508 856 526 476 648 784 838 582 354 338 346 218 292 502 24 648 436 638 772 654 212 438 429 340 452 498 327 580 572 350 470 533

K00135 0 0 0 0 1 0 0 0 0 0 3 0 0 0 0 0 0 0 0 0 0 0 0 17 0 0 0 0 0 4 0 26 0 0 0 0 0 0 0 0 0 0 0 0 0 0 0 0

K00137 0 0 0 0 0 0 0 0 0 0 0 0 0 0 0 0 0 0 0 0 0 0 0 0 0 0 0 0 0 0 0 0 0 0 0 0 0 0 0 0 0 0 0 0 0 0 0 0

K00138 0 0 0 0 0 0 0 0 0 0 0 0 0 0 0 0 0 0 0 0 0 0 0 0 0 0 0 0 0 0 0 0 0 0 0 0 0 0 0 0 0 0 0 0 0 0 0 0

K00139 0 0 0 0 0 0 0 0 0 0 0 0 0 0 0 0 0 0 0 0 0 0 0 0 0 0 0 0 0 0 0 0 0 0 0 0 0 0 0 0 0 0 0 0 0 0 0 0

K00140 338 420 344 166 406 320 267 564 552 574 661 728 268 601 372 202 508 856 526 476 648 784 838 582 354 338 346 218 292 502 24 648 436 638 772 654 212 438 429 340 452 498 327 580 572 350 470 533

K00141 0 0 0 0 0 0 0 0 0 0 0 0 0 0 0 0 0 0 0 0 0 0 0 0 0 0 0 0 0 0 0 0 0 0 0 0 0 0 0 0 0 0 0 0 0 0 0 0

K00145 842 984 937 427 1507 1186 679 1986 1298 1397 1772 1628 591 1468 1055 504 1294 2251 1169 1156 1607 1906 2064 1645 754 737 745 517 672 1756 48 1602 1067 1525 1768 1529 498 1016 1139 973 1370 1392 785 1516 1447 1026 1134 1654

K00146 0 0 0 0 0 0 0 0 0 0 0 0 0 0 0 0 0 0 0 0 0 0 0 0 0 0 0 0 0 0 0 0 0 0 0 0 0 0 0 0 0 0 0 0 0 0 0 0

K00147 338 420 344 166 406 320 267 564 552 574 661 728 268 601 372 202 508 856 526 476 648 784 838 582 354 338 346 218 292 502 24 648 436 638 772 654 212 438 429 340 452 498 327 580 572 350 470 533

K00148 0 0 0 0 0 0 0 0 0 0 0 0 0 0 0 0 0 0 0 0 0 0 0 0 0 0 0 0 0 0 0 0 0 0 0 0 0 0 0 0 0 0 0 0 0 0 0 0

K00150 140 304 301 96 573 477 136 656 216 244 481 240 119 325 347 119 410 526 223 347 439 342 496 482 120 132 126 90 166 704 0 391 212 228 322 280 96 320 486 328 500 486 182 590 590 308 318 602

K00151 0 0 0 0 0 0 0 0 0 0 0 0 0 0 0 0 0 0 0 0 0 0 0 0 0 0 0 0 0 0 0 0 0 0 0 0 0 0 0 0 0 0 0 0 0 0 0 0

K00152 0 0 0 0 0 0 0 0 0 0 0 0 0 0 0 0 0 0 0 0 0 0 0 0 0 0 0 0 0 0 0 0 0 0 0 0 0 0 0 0 0 0 0 0 0 0 0 0

K00153 0 0 0 0 0 0 0 0 0 0 0 0 0 0 0 0 0 0 0 0 0 0 0 0 0 0 0 0 0 0 0 0 0 0 0 0 0 0 0 0 0 0 0 0 0 0 0 0

K00154 0 0 0 0 0 0 0 0 0 0 0 0 0 0 0 0 0 0 0 0 0 0 0 0 0 0 0 0 0 0 0 0 0 0 0 0 0 0 0 0 0 0 0 0 0 0 0 0

K00155 0 0 0 0 0 0 0 0 0 0 0 0 0 0 0 0 0 0 0 0 0 0 0 0 0 0 0 0 0 0 0 0 0 0 0 0 0 0 0 0 0 0 0 0 0 0 0 0

K00156 0 0 0 0 0 0 0 0 0 0 0 0 0 0 0 0 0 0 0 0 0 0 0 0 0 0 0 0 0 0 0 0 0 0 0 0 0 0 0 0 0 0 0 0 0 0 0 0

K00157 0 0 0 0 0 0 0 0 0 0 0 0 0 0 0 0 0 0 0 0 0 0 0 0 0 0 0 0 0 0 0 0 0 0 0 0 0 0 0 0 0 0 0 0 0 0 0 0

K00158 0 0 0 0 23 16 0 9 0 0 3 0 0 0 0 0 0 14 0 0 0 0 0 23 0 0 0 0 0 18 0 30 0 0 0 0 0 0 0 0 0 0 0 0 0 0 0 0

K00161 338 420 344 166 406 320 267 564 552 574 661 728 268 601 372 202 508 856 526 476 648 784 838 582 354 338 346 218 292 502 24 648 436 638 772 654 212 438 429 340 452 498 327 580 572 350 470 533

K00162 338 420 344 166 406 320 267 564 552 574 661 728 268 601 372 202 508 856 526 476 648 784 838 582 354 338 346 218 292 502 24 648 436 638 772 654 212 438 429 340 452 498 327 580 572 350 470 533

K00163 0 0 0 0 0 0 0 0 0 0 0 0 0 0 0 0 0 0 0 0 0 0 0 0 0 0 0 0 0 0 0 0 0 0 0 0 0 0 0 0 0 0 0 0 0 0 0 0

K00164 0 0 0 0 0 0 0 0 0 0 0 0 0 0 0 0 0 0 0 0 0 0 0 0 0 0 0 0 0 0 0 0 0 0 0 0 0 0 0 0 0 0 0 0 0 0 0 0

K00166 0 0 0 0 0 0 0 0 0 0 0 0 0 0 0 0 0 0 0 0 0 0 0 0 0 0 0 0 0 0 0 0 0 0 0 0 0 0 0 0 0 0 0 0 0 0 0 0

K00167 0 0 0 0 0 0 0 0 0 0 0 0 0 0 0 0 0 0 0 0 0 0 0 0 0 0 0 0 0 0 0 0 0 0 0 0 0 0 0 0 0 0 0 0 0 0 0 0

K00169 478 835 750 286 1076 862 428 1310 832 882 1182 1072 422 956 816 320 1054 1490 772 914 1182 1152 1348 1121 494 471 482 308 488 1354 24 1144 702 889 1208 968 308 790 1041 790 1126 1141 571 1380 1344 697 894 1190

K00170 478 835 750 286 1076 862 428 1310 832 882 1182 1072 422 956 816 320 1054 1490 772 914 1182 1152 1348 1121 494 471 482 308 488 1354 24 1144 702 889 1208 968 308 790 1041 790 1126 1141 571 1380 1344 697 894 1190

K00171 478 835 750 286 1070 862 420 1310 832 882 1182 1072 422 956 800 320 1054 1490 772 914 1182 1152 1348 1108 494 471 482 308 488 1354 24 1144 702 889 1208 968 308 790 1041 790 1126 1132 571 1380 1344 697 894 1190

K00172 478 835 750 286 1076 862 428 1310 832 882 1182 1072 422 956 816 320 1054 1490 772 914 1182 1152 1348 1121 494 471 482 308 488 1354 24 1144 702 889 1208 968 308 790 1041 790 1126 1141 571 1380 1344 697 894 1190

K00174 492 724 645 262 1008 814 412 1228 768 818 1144 968 386 926 735 320 919 1396 748 841 1088 1127 1335 1100 473 471 472 308 458 1224 24 1068 648 890 1094 934 308 759 916 668 952 992 510 1170 1161 658 788 1134

K00175 492 724 645 262 1008 814 412 1228 768 818 1144 968 386 926 735 320 919 1396 748 841 1088 1127 1335 1100 473 471 472 308 458 1224 24 1068 648 890 1094 934 308 759 916 668 952 992 510 1170 1161 658 788 1134

K00176 478 724 645 262 978 798 404 1219 768 818 1144 968 386 926 719 320 919 1382 748 822 1088 1127 1335 1080 473 471 472 308 458 1210 24 1068 648 866 1094 934 308 759 916 668 952 984 510 1170 1161 658 788 1134

K00177 478 724 645 262 978 798 404 1219 768 818 1142 968 386 926 719 320 919 1382 748 822 1088 1127 1335 1064 473 471 472 308 458 1206 24 1038 648 866 1094 934 308 759 916 668 952 984 510 1170 1161 658 788 1134

K00178 0 0 0 0 0 0 0 0 0 0 0 0 0 0 0 0 0 0 0 0 0 0 0 0 0 0 0 0 0 0 0 0 0 0 0 0 0 0 0 0 0 0 0 0 0 0 0 0

K00179 478 724 645 262 978 798 404 1219 768 818 1142 968 386 926 719 320 919 1382 748 822 1088 1127 1335 1064 473 471 472 308 458 1206 24 1038 648 866 1094 934 308 759 916 668 952 984 510 1170 1161 658 788 1134

K00180 478 724 645 262 978 798 404 1219 768 818 1142 968 386 926 719 320 919 1382 748 822 1088 1127 1335 1064 473 471 472 308 458 1206 24 1038 648 866 1094 934 308 759 916 668 952 984 510 1170 1161 658 788 1134

K00183 0 0 0 0 0 0 0 0 0 0 0 0 0 0 0 0 0 0 0 0 0 0 0 0 0 0 0 0 0 0 0 0 0 0 0 0 0 0 0 0 0 0 0 0 0 0 0 0

K00184 0 0 0 0 0 0 0 0 0 0 0 0 0 0 0 0 0 0 0 0 0 0 0 0 0 0 0 0 0 0 0 0 0 0 0 0 0 0 0 0 0 0 0 0 0 0 0 0

K00185 0 0 0 0 0 0 0 0 0 0 0 0 0 0 0 0 0 0 0 0 0 0 0 0 0 0 0 0 0 0 0 0 0 0 0 0 0 0 0 0 0 0 0 0 0 0 0 0

K00186 66 40 107 47 258 200 72 338 84 124 192 86 28 72 124 27 101 208 59 62 106 150 124 186 12 30 21 9 38 274 0 50 50 88 83 77 37 54 90 122 168 154 28 103 96 144 59 230

K00187 66 40 107 47 258 200 72 338 84 124 192 86 28 72 124 27 101 208 59 62 106 150 124 186 12 30 21 9 38 274 0 50 50 88 83 77 37 54 90 122 168 154 28 103 96 144 59 230

K00188 66 40 107 47 258 200 72 338 84 124 192 86 28 72 124 27 101 208 59 62 106 150 124 186 12 30 21 9 38 274 0 50 50 88 83 77 37 54 90 122 168 154 28 103 96 144 59 230

K00189 0 0 0 0 0 0 0 0 0 0 0 0 0 0 0 0 0 0 0 0 0 0 0 0 0 0 0 0 0 0 0 0 0 0 0 0 0 0 0 0 0 0 0 0 0 0 0 0

K00190 0 0 0 0 0 0 0 0 0 0 0 0 0 0 0 0 0 0 0 0 0 0 0 0 0 0 0 0 0 0 0 0 0 0 0 0 0 0 0 0 0 0 0 0 0 0 0 0

K00191 0 0 0 0 0 0 0 0 0 0 0 0 0 0 0 0 0 0 0 0 0 0 0 0 0 0 0 0 0 0 0 0 0 0 0 0 0 0 0 0 0 0 0 0 0 0 0 0

K00192 42 0 0 0 14 0 17 0 0 0 0 0 0 0 32 0 0 0 0 56 0 0 0 27 0 0 0 0 0 0 0 0 0 74 0 0 0 0 0 0 0 17 0 0 0 0 0 0

K00193 14 0 0 0 8 0 8 0 0 0 0 0 0 0 16 0 0 0 0 18 0 0 0 14 0 0 0 0 0 0 0 0 0 24 0 0 0 0 0 0 0 8 0 0 0 0 0 0

K00194 14 0 0 0 8 0 8 0 0 0 3 0 0 0 16 0 0 0 0 18 0 0 0 14 0 0 0 0 0 0 0 0 0 24 0 0 0 0 0 0 0 8 0 0 0 0 0 0

K00195 28 0 0 0 8 0 8 0 0 0 0 0 0 0 16 0 0 0 0 37 0 0 0 14 0 0 0 0 0 0 0 0 0 49 0 0 0 0 0 0 0 8 0 0 0 0 0 0

K00196 66 40 107 47 258 200 72 338 84 124 192 86 28 72 124 27 101 208 59 62 106 150 124 186 12 30 21 9 38 274 0 50 50 88 83 77 37 54 90 122 168 154 28 103 96 144 59 230

K00197 14 0 0 0 8 0 8 0 0 0 3 0 0 0 16 0 0 0 0 18 0 0 0 14 0 0 0 0 0 0 0 0 0 24 0 0 0 0 0 0 0 8 0 0 0 0 0 0

K00198 0 0 0 0 1 0 0 0 0 0 3 0 0 0 0 0 0 0 0 0 0 0 0 17 0 0 0 0 0 4 0 34 0 0 0 0 0 0 0 0 0 0 0 0 0 0 0 0

K00200 99 104 142 47 460 362 72 529 110 124 261 86 28 194 188 74 176 345 59 142 204 186 263 302 35 30 32 71 49 492 0 256 144 161 140 143 37 85 192 171 298 242 103 254 208 184 134 358

K00201 113 104 142 47 490 378 81 538 110 124 261 86 28 194 204 74 176 359 59 160 204 186 263 339 35 30 32 71 49 509 0 283 144 186 140 143 37 85 192 171 298 250 103 254 208 184 134 358

K00202 113 104 142 47 467 362 81 529 110 124 261 86 28 194 204 74 176 345 59 160 204 186 263 316 35 30 32 71 49 492 0 256 144 186 140 143 37 85 192 171 298 250 103 254 208 184 134 358

K00203 80 40 107 47 312 232 81 356 84 124 197 86 28 72 140 27 101 236 59 81 106 150 124 245 12 30 21 9 38 309 0 110 50 112 83 77 37 54 90 122 168 162 28 103 96 144 59 230

K00204 66 40 107 47 258 200 72 338 84 124 192 86 28 72 124 27 101 208 59 62 106 150 124 186 12 30 21 9 38 274 0 50 50 88 83 77 37 54 90 122 168 154 28 103 96 144 59 230

K00205 166 144 249 94 672 530 145 850 194 249 448 171 56 266 311 100 278 525 118 204 310 336 386 459 46 60 53 80 87 735 0 277 195 249 222 220 74 140 281 293 466 395 131 356 304 327 192 588

K00206 0 0 0 0 0 0 0 0 0 0 0 0 0 0 0 0 0 0 0 0 0 0 0 0 0 0 0 0 0 0 0 0 0 0 0 0 0 0 0 0 0 0 0 0 0 0 0 0

K00207 0 0 0 0 0 0 0 0 0 0 0 0 0 0 0 0 0 0 0 0 0 0 0 0 0 0 0 0 0 0 0 0 0 0 0 0 0 0 0 0 0 0 0 0 0 0 0 0

K00208 0 0 0 0 0 0 0 0 0 0 0 0 0 0 0 0 0 0 0 0 0 0 0 0 0 0 0 0 0 0 0 0 0 0 0 0 0 0 0 0 0 0 0 0 0 0 0 0

K00209 0 0 0 0 0 0 0 0 0 0 0 0 0 0 0 0 0 0 0 0 0 0 0 0 0 0 0 0 0 0 0 0 0 0 0 0 0 0 0 0 0 0 0 0 0 0 0 0

K00210 0 0 0 0 0 0 0 0 0 0 0 0 0 0 0 0 0 0 0 0 0 0 0 0 0 0 0 0 0 0 0 0 0 0 0 0 0 0 0 0 0 0 0 0 0 0 0 0

K00211 0 0 0 0 0 0 0 0 0 0 0 0 0 0 0 0 0 0 0 0 0 0 0 0 0 0 0 0 0 0 0 0 0 0 0 0 0 0 0 0 0 0 0 0 0 0 0 0

K00213 0 0 0 0 0 0 0 0 0 0 0 0 0 0 0 0 0 0 0 0 0 0 0 0 0 0 0 0 0 0 0 0 0 0 0 0 0 0 0 0 0 0 0 0 0 0 0 0

K00214 0 0 0 0 0 0 0 0 0 0 0 0 0 0 0 0 0 0 0 0 0 0 0 0 0 0 0 0 0 0 0 0 0 0 0 0 0 0 0 0 0 0 0 0 0 0 0 0

K00215 437 524 486 214 819 650 340 1074 662 698 917 814 296 794 560 275 685 1173 584 618 853 970 1102 855 388 368 378 290 342 963 24 874 580 799 912 798 249 524 620 511 750 740 430 833 780 533 604 891

K00216 0 0 0 0 0 0 0 0 0 0 0 0 0 0 0 0 0 0 0 0 0 0 0 0 0 0 0 0 0 0 0 0 0 0 0 0 0 0 0 0 0 0 0 0 0 0 0 0

K00217 0 0 0 0 0 0 0 0 0 0 0 0 0 0 0 0 0 0 0 0 0 0 0 0 0 0 0 0 0 0 0 0 0 0 0 0 0 0 0 0 0 0 0 0 0 0 0 0

K00218 0 0 0 0 0 0 0 0 0 0 0 0 0 0 0 0 0 0 0 0 0 0 0 0 0 0 0 0 0 0 0 0 0 0 0 0 0 0 0 0 0 0 0 0 0 0 0 0

K00219 0 0 0 0 0 0 0 0 0 0 0 0 0 0 0 0 0 0 0 0 0 0 0 0 0 0 0 0 0 0 0 0 0 0 0 0 0 0 0 0 0 0 0 0 0 0 0 0

K00220 0 0 0 0 0 0 0 0 0 0 0 0 0 0 0 0 0 0 0 0 0 0 0 0 0 0 0 0 0 0 0 0 0 0 0 0 0 0 0 0 0 0 0 0 0 0 0 0

K00221 0 0 0 0 0 0 0 0 0 0 0 0 0 0 0 0 0 0 0 0 0 0 0 0 0 0 0 0 0 0 0 0 0 0 0 0 0 0 0 0 0 0 0 0 0 0 0 0

K00222 0 0 0 0 0 0 0 0 0 0 0 0 0 0 0 0 0 0 0 0 0 0 0 0 0 0 0 0 0 0 0 0 0 0 0 0 0 0 0 0 0 0 0 0 0 0 0 0

K00223 0 0 0 0 0 0 0 0 0 0 0 0 0 0 0 0 0 0 0 0 0 0 0 0 0 0 0 0 0 0 0 0 0 0 0 0 0 0 0 0 0 0 0 0 0 0 0 0

K00224 0 0 0 0 0 0 0 0 0 0 0 0 0 0 0 0 0 0 0 0 0 0 0 0 0 0 0 0 0 0 0 0 0 0 0 0 0 0 0 0 0 0 0 0 0 0 0 0

K00226 478 724 645 262 978 798 404 1219 768 818 1142 968 386 926 719 320 919 1382 748 822 1088 1127 1335 1064 473 471 472 308 458 1206 24 1038 648 866 1094 934 308 759 916 668 952 984 510 1170 1161 658 788 1134

K00227 0 0 0 0 0 0 0 0 0 0 0 0 0 0 0 0 0 0 0 0 0 0 0 0 0 0 0 0 0 0 0 0 0 0 0 0 0 0 0 0 0 0 0 0 0 0 0 0

K00228 0 0 0 0 0 0 0 0 0 0 0 0 0 0 0 0 0 0 0 0 0 0 0 0 0 0 0 0 0 0 0 0 0 0 0 0 0 0 0 0 0 0 0 0 0 0 0 0

K00230 52 40 107 47 275 216 64 348 84 124 195 86 28 72 108 27 101 222 59 44 106 150 124 195 12 30 21 9 38 292 0 84 50 64 83 77 37 54 90 122 168 145 28 103 96 144 59 230

K00231 0 0 0 0 0 0 0 0 0 0 0 0 0 0 0 0 0 0 0 0 0 0 0 0 0 0 0 0 0 0 0 0 0 0 0 0 0 0 0 0 0 0 0 0 0 0 0 0

K00232 0 0 0 0 0 0 0 0 0 0 0 0 0 0 0 0 0 0 0 0 0 0 0 0 0 0 0 0 0 0 0 0 0 0 0 0 0 0 0 0 0 0 0 0 0 0 0 0

K00234 0 0 0 0 0 0 0 0 0 0 0 0 0 0 0 0 0 0 0 0 0 0 0 0 0 0 0 0 0 0 0 0 0 0 0 0 0 0 0 0 0 0 0 0 0 0 0 0

K00239 478 724 645 262 978 798 404 1219 768 818 1142 968 386 926 719 320 919 1382 748 822 1088 1127 1335 1064 473 471 472 308 458 1206 24 1038 648 866 1094 934 308 759 916 668 952 984 510 1170 1161 658 788 1134

K00240 338 420 344 166 406 320 267 564 552 574 661 728 268 601 372 202 508 856 526 476 648 784 838 582 354 338 346 218 292 502 24 648 436 638 772 654 212 438 429 340 452 498 327 580 572 350 470 533

K00241 338 420 344 166 406 320 267 564 552 574 661 728 268 601 372 202 508 856 526 476 648 784 838 582 354 338 346 218 292 502 24 648 436 638 772 654 212 438 429 340 452 498 327 580 572 350 470 533

K00242 0 0 0 0 0 0 0 0 0 0 0 0 0 0 0 0 0 0 0 0 0 0 0 0 0 0 0 0 0 0 0 0 0 0 0 0 0 0 0 0 0 0 0 0 0 0 0 0

K00243 0 0 0 0 0 0 0 0 0 0 0 0 0 0 0 0 0 0 0 0 0 0 0 0 0 0 0 0 0 0 0 0 0 0 0 0 0 0 0 0 0 0 0 0 0 0 0 0

K00244 0 0 0 0 0 0 0 0 0 0 0 0 0 0 0 0 0 0 0 0 0 0 0 0 0 0 0 0 0 0 0 0 0 0 0 0 0 0 0 0 0 0 0 0 0 0 0 0

K00245 66 40 107 47 282 216 72 348 84 124 195 86 28 72 124 27 101 222 59 62 106 150 124 208 12 30 21 9 38 292 0 80 50 88 83 77 37 54 90 122 168 154 28 103 96 144 59 230

K00246 0 0 0 0 0 0 0 0 0 0 0 0 0 0 0 0 0 0 0 0 0 0 0 0 0 0 0 0 0 0 0 0 0 0 0 0 0 0 0 0 0 0 0 0 0 0 0 0

K00247 0 0 0 0 0 0 0 0 0 0 0 0 0 0 0 0 0 0 0 0 0 0 0 0 0 0 0 0 0 0 0 0 0 0 0 0 0 0 0 0 0 0 0 0 0 0 0 0

K00248 0 0 0 0 0 0 0 0 0 0 0 0 0 0 0 0 0 0 0 0 0 0 0 0 0 0 0 0 0 0 0 0 0 0 0 0 0 0 0 0 0 0 0 0 0 0 0 0

K00249 380 620 503 216 564 468 331 708 657 694 886 882 358 732 532 247 742 1066 690 680 883 941 1072 790 438 441 440 238 409 745 24 812 504 705 954 792 270 674 724 497 653 742 406 916 953 474 654 776

K00252 0 0 0 0 0 0 0 0 0 0 0 0 0 0 0 0 0 0 0 0 0 0 0 0 0 0 0 0 0 0 0 0 0 0 0 0 0 0 0 0 0 0 0 0 0 0 0 0

K00253 0 0 0 0 0 0 0 0 0 0 0 0 0 0 0 0 0 0 0 0 0 0 0 0 0 0 0 0 0 0 0 0 0 0 0 0 0 0 0 0 0 0 0 0 0 0 0 0

K00254 0 0 0 0 0 0 0 0 0 0 0 0 0 0 0 0 0 0 0 0 0 0 0 0 0 0 0 0 0 0 0 0 0 0 0 0 0 0 0 0 0 0 0 0 0 0 0 0

K00255 0 0 0 0 0 0 0 0 0 0 0 0 0 0 0 0 0 0 0 0 0 0 0 0 0 0 0 0 0 0 0 0 0 0 0 0 0 0 0 0 0 0 0 0 0 0 0 0

K00256 0 0 0 0 0 0 0 0 0 0 0 0 0 0 0 0 0 0 0 0 0 0 0 0 0 0 0 0 0 0 0 0 0 0 0 0 0 0 0 0 0 0 0 0 0 0 0 0

K00257 338 420 344 166 406 320 267 564 552 574 661 728 268 601 372 202 508 856 526 476 648 784 838 582 354 338 346 218 292 502 24 648 436 638 772 654 212 438 429 340 452 498 327 580 572 350 470 533

K00258 0 0 0 0 0 0 0 0 0 0 0 0 0 0 0 0 0 0 0 0 0 0 0 0 0 0 0 0 0 0 0 0 0 0 0 0 0 0 0 0 0 0 0 0 0 0 0 0

K00259 0 0 0 0 6 0 8 0 0 0 0 0 0 0 16 0 0 0 0 0 0 0 0 14 0 0 0 0 0 0 0 0 0 0 0 0 0 0 0 0 0 8 0 0 0 0 0 0

K00260 0 0 0 0 0 0 0 0 0 0 0 0 0 0 0 0 0 0 0 0 0 0 0 0 0 0 0 0 0 0 0 0 0 0 0 0 0 0 0 0 0 0 0 0 0 0 0 0

K00261 32 64 35 0 131 114 0 164 26 0 61 0 0 122 64 46 76 95 0 80 99 36 140 65 24 0 12 62 11 170 0 146 94 73 56 66 0 30 102 49 130 88 75 150 112 40 74 128

K00262 94 240 266 96 410 346 128 483 190 244 417 240 119 204 267 72 335 418 223 249 340 307 357 380 96 132 114 28 154 517 0 215 118 130 265 214 96 290 384 279 369 389 108 440 478 268 243 474

K00263 0 0 0 0 0 0 0 0 0 0 0 0 0 0 0 0 0 0 0 0 0 0 0 0 0 0 0 0 0 0 0 0 0 0 0 0 0 0 0 0 0 0 0 0 0 0 0 0

K00265 338 420 344 166 406 320 267 564 552 574 661 728 268 601 372 202 508 856 526 476 648 784 838 582 354 338 346 218 292 502 24 648 436 638 772 654 212 438 429 340 452 498 327 580 572 350 470 533

K00266 750 1214 986 406 1220 984 622 1534 1300 1332 1651 1715 662 1485 1064 495 1462 2138 1238 1328 1724 1786 2064 1517 836 780 808 518 742 1582 48 1741 1087 1439 1898 1546 482 1174 1380 1008 1410 1486 870 1856 1820 903 1306 1492

K00270 0 0 0 0 0 0 0 0 0 0 0 0 0 0 0 0 0 0 0 0 0 0 0 0 0 0 0 0 0 0 0 0 0 0 0 0 0 0 0 0 0 0 0 0 0 0 0 0

K00271 0 0 0 0 0 0 0 0 0 0 0 0 0 0 0 0 0 0 0 0 0 0 0 0 0 0 0 0 0 0 0 0 0 0 0 0 0 0 0 0 0 0 0 0 0 0 0 0

K00272 0 0 0 0 0 0 0 0 0 0 0 0 0 0 0 0 0 0 0 0 0 0 0 0 0 0 0 0 0 0 0 0 0 0 0 0 0 0 0 0 0 0 0 0 0 0 0 0

K00273 0 0 0 0 0 0 0 0 0 0 0 0 0 0 0 0 0 0 0 0 0 0 0 0 0 0 0 0 0 0 0 0 0 0 0 0 0 0 0 0 0 0 0 0 0 0 0 0

K00274 0 0 0 0 0 0 0 0 0 0 0 0 0 0 0 0 0 0 0 0 0 0 0 0 0 0 0 0 0 0 0 0 0 0 0 0 0 0 0 0 0 0 0 0 0 0 0 0

K00275 0 0 0 0 0 0 0 0 0 0 0 0 0 0 0 0 0 0 0 0 0 0 0 0 0 0 0 0 0 0 0 0 0 0 0 0 0 0 0 0 0 0 0 0 0 0 0 0

K00276 0 0 0 0 0 0 0 0 0 0 0 0 0 0 0 0 0 0 0 0 0 0 0 0 0 0 0 0 0 0 0 0 0 0 0 0 0 0 0 0 0 0 0 0 0 0 0 0

K00278 338 420 344 166 406 320 267 564 552 574 661 728 268 601 372 202 508 856 526 476 648 784 838 582 354 338 346 218 292 502 24 648 436 638 772 654 212 438 429 340 452 498 327 580 572 350 470 533

K00279 0 0 0 0 0 0 0 0 0 0 0 0 0 0 0 0 0 0 0 0 0 0 0 0 0 0 0 0 0 0 0 0 0 0 0 0 0 0 0 0 0 0 0 0 0 0 0 0

K00280 0 0 0 0 0 0 0 0 0 0 0 0 0 0 0 0 0 0 0 0 0 0 0 0 0 0 0 0 0 0 0 0 0 0 0 0 0 0 0 0 0 0 0 0 0 0 0 0

K00281 0 0 0 0 0 0 0 0 0 0 0 0 0 0 0 0 0 0 0 0 0 0 0 0 0 0 0 0 0 0 0 0 0 0 0 0 0 0 0 0 0 0 0 0 0 0 0 0

K00282 42 200 159 49 158 147 64 144 106 120 225 154 91 132 160 46 234 210 164 205 234 156 234 208 84 102 94 19 116 243 0 164 68 67 182 137 58 236 295 157 201 244 80 337 382 125 184 244

K00283 42 200 159 49 158 147 64 144 106 120 225 154 91 132 160 46 234 210 164 205 234 156 234 208 84 102 94 19 116 243 0 164 68 67 182 137 58 236 295 157 201 244 80 337 382 125 184 244

K00284 0 0 0 0 0 0 0 0 0 0 0 0 0 0 0 0 0 0 0 0 0 0 0 0 0 0 0 0 0 0 0 0 0 0 0 0 0 0 0 0 0 0 0 0 0 0 0 0

K00285 0 0 0 0 0 0 0 0 0 0 0 0 0 0 0 0 0 0 0 0 0 0 0 0 0 0 0 0 0 0 0 0 0 0 0 0 0 0 0 0 0 0 0 0 0 0 0 0

K00286 404 460 451 214 687 536 340 911 636 698 856 814 296 673 496 228 610 1078 584 538 754 935 962 773 365 368 367 228 330 789 24 702 486 726 856 732 249 493 518 462 620 652 355 682 668 493 530 764

K00287 0 0 0 0 0 0 0 0 0 0 0 0 0 0 0 0 0 0 0 0 0 0 0 0 0 0 0 0 0 0 0 0 0 0 0 0 0 0 0 0 0 0 0 0 0 0 0 0

K00288 0 0 0 0 0 0 0 0 0 0 0 0 0 0 0 0 0 0 0 0 0 0 0 0 0 0 0 0 0 0 0 0 0 0 0 0 0 0 0 0 0 0 0 0 0 0 0 0

K00290 0 0 0 0 0 0 0 0 0 0 0 0 0 0 0 0 0 0 0 0 0 0 0 0 0 0 0 0 0 0 0 0 0 0 0 0 0 0 0 0 0 0 0 0 0 0 0 0

K00291 0 0 0 0 0 0 0 0 0 0 0 0 0 0 0 0 0 0 0 0 0 0 0 0 0 0 0 0 0 0 0 0 0 0 0 0 0 0 0 0 0 0 0 0 0 0 0 0

K00292 0 0 0 0 0 0 0 0 0 0 0 0 0 0 0 0 0 0 0 0 0 0 0 0 0 0 0 0 0 0 0 0 0 0 0 0 0 0 0 0 0 0 0 0 0 0 0 0

K00293 0 0 0 0 0 0 0 0 0 0 0 0 0 0 0 0 0 0 0 0 0 0 0 0 0 0 0 0 0 0 0 0 0 0 0 0 0 0 0 0 0 0 0 0 0 0 0 0

K00294 0 0 0 0 0 0 0 0 0 0 0 0 0 0 0 0 0 0 0 0 0 0 0 0 0 0 0 0 0 0 0 4 0 0 0 0 0 0 0 0 0 0 0 0 0 0 0 0

K00296 0 0 0 0 0 0 0 0 0 0 0 0 0 0 0 0 0 0 0 0 0 0 0 0 0 0 0 0 0 0 0 0 0 0 0 0 0 0 0 0 0 0 0 0 0 0 0 0

K00297 0 0 0 0 0 0 0 0 0 0 0 0 0 0 0 0 0 0 0 0 0 0 0 0 0 0 0 0 0 0 0 0 0 0 0 0 0 0 0 0 0 0 0 0 0 0 0 0

K00298 0 0 0 0 0 0 0 0 0 0 0 0 0 0 0 0 0 0 0 0 0 0 0 0 0 0 0 0 0 0 0 0 0 0 0 0 0 0 0 0 0 0 0 0 0 0 0 0

K00299 0 0 0 0 0 0 0 0 0 0 0 0 0 0 0 0 0 0 0 0 0 0 0 0 0 0 0 0 0 0 0 0 0 0 0 0 0 0 0 0 0 0 0 0 0 0 0 0

K00300 0 0 0 0 0 0 0 0 0 0 0 0 0 0 0 0 0 0 0 0 0 0 0 0 0 0 0 0 0 0 0 0 0 0 0 0 0 0 0 0 0 0 0 0 0 0 0 0

K00301 0 0 0 0 0 0 0 0 0 0 0 0 0 0 0 0 0 0 0 0 0 0 0 0 0 0 0 0 0 0 0 0 0 0 0 0 0 0 0 0 0 0 0 0 0 0 0 0

K00302 42 200 159 49 158 147 64 144 106 120 225 154 91 132 160 46 234 210 164 205 234 156 234 208 84 102 94 19 116 243 0 164 68 67 182 137 58 236 295 157 201 244 80 337 382 125 184 244

K00303 42 200 159 49 158 147 64 144 106 120 225 154 91 132 160 46 234 210 164 205 234 156 234 208 84 102 94 19 116 243 0 164 68 67 182 137 58 236 295 157 201 244 80 337 382 125 184 244

K00304 0 0 0 0 0 0 0 0 0 0 0 0 0 0 0 0 0 0 0 0 0 0 0 0 0 0 0 0 0 0 0 0 0 0 0 0 0 0 0 0 0 0 0 0 0 0 0 0

K00305 0 0 0 0 0 0 0 0 0 0 0 0 0 0 0 0 0 0 0 0 0 0 0 0 0 0 0 0 0 0 0 0 0 0 0 0 0 0 0 0 0 0 0 0 0 0 0 0

K00306 0 0 0 0 0 0 0 0 0 0 0 0 0 0 0 0 0 0 0 0 0 0 0 0 0 0 0 0 0 0 0 0 0 0 0 0 0 0 0 0 0 0 0 0 0 0 0 0

K00309 0 0 0 0 0 0 0 0 0 0 0 0 0 0 0 0 0 0 0 0 0 0 0 0 0 0 0 0 0 0 0 0 0 0 0 0 0 0 0 0 0 0 0 0 0 0 0 0

K00311 0 0 0 0 0 0 0 0 0 0 0 0 0 0 0 0 0 0 0 0 0 0 0 0 0 0 0 0 0 0 0 0 0 0 0 0 0 0 0 0 0 0 0 0 0 0 0 0

K00313 0 0 0 0 0 0 0 0 0 0 0 0 0 0 0 0 0 0 0 0 0 0 0 0 0 0 0 0 0 0 0 0 0 0 0 0 0 0 0 0 0 0 0 0 0 0 0 0

K00314 0 0 0 0 0 0 0 0 0 0 0 0 0 0 0 0 0 0 0 0 0 0 0 0 0 0 0 0 0 0 0 0 0 0 0 0 0 0 0 0 0 0 0 0 0 0 0 0

K00315 0 0 0 0 0 0 0 0 0 0 0 0 0 0 0 0 0 0 0 0 0 0 0 0 0 0 0 0 0 0 0 0 0 0 0 0 0 0 0 0 0 0 0 0 0 0 0 0

K00316 0 0 0 0 0 0 0 0 0 0 0 0 0 0 0 0 0 0 0 0 0 0 0 0 0 0 0 0 0 0 0 0 0 0 0 0 0 0 0 0 0 0 0 0 0 0 0 0

K00317 0 0 0 0 0 0 0 0 0 0 0 0 0 0 0 0 0 0 0 0 0 0 0 0 0 0 0 0 0 0 0 0 0 0 0 0 0 0 0 0 0 0 0 0 0 0 0 0

K00318 0 0 0 0 0 0 0 0 0 0 0 0 0 0 0 0 0 0 0 0 0 0 0 0 0 0 0 0 0 0 0 4 0 0 0 0 0 0 0 0 0 0 0 0 0 0 0 0

K00319 66 40 107 47 282 216 72 348 84 124 195 86 28 72 124 27 101 222 59 62 106 150 124 208 12 30 21 9 38 292 0 80 50 88 83 77 37 54 90 122 168 154 28 103 96 144 59 230

K00320 99 104 142 47 414 330 72 511 110 124 256 86 28 194 188 74 176 317 59 142 204 186 263 274 35 30 32 71 49 461 0 226 144 161 140 143 37 85 192 171 298 242 103 254 208 184 134 358

K00321 0 0 0 0 0 0 0 0 0 0 0 0 0 0 0 0 0 0 0 0 0 0 0 0 0 0 0 0 0 0 0 0 0 0 0 0 0 0 0 0 0 0 0 0 0 0 0 0

K00322 0 0 0 0 0 0 0 0 0 0 0 0 0 0 0 0 0 0 0 0 0 0 0 0 0 0 0 0 0 0 0 0 0 0 0 0 0 0 0 0 0 0 0 0 0 0 0 0

K00324 338 420 344 166 408 320 267 564 552 574 661 728 268 601 372 202 508 856 526 476 648 784 838 582 354 338 346 218 292 502 24 648 436 638 772 654 212 438 429 340 452 498 327 580 572 350 470 533

K00325 0 0 0 0 1 0 0 0 0 0 0 0 0 0 0 0 0 0 0 0 0 0 0 0 0 0 0 0 0 0 0 0 0 0 0 0 0 0 0 0 0 0 0 0 0 0 0 0

K00326 0 0 0 0 0 0 0 0 0 0 0 0 0 0 0 0 0 0 0 0 0 0 0 0 0 0 0 0 0 0 0 0 0 0 0 0 0 0 0 0 0 0 0 0 0 0 0 0

K00329 42 200 159 49 158 147 64 144 106 120 228 154 91 132 160 46 234 210 164 205 234 156 234 226 84 102 94 19 116 248 0 194 68 67 182 137 58 236 295 157 201 244 80 337 382 125 184 244

K00330 338 420 344 166 406 320 267 564 552 574 661 728 268 601 372 202 508 856 526 476 648 784 838 582 354 338 346 218 292 502 24 648 436 638 772 654 212 438 429 340 452 498 327 580 572 350 470 533

K00331 412 684 538 216 695 582 331 872 684 694 947 882 358 854 596 294 818 1160 690 760 982 976 1212 855 462 441 451 300 420 914 24 958 598 778 1011 858 270 704 826 546 784 830 482 1067 1065 514 729 904

K00332 380 620 503 216 564 468 331 708 657 694 886 882 358 732 532 247 742 1066 690 680 883 941 1072 790 438 441 440 238 409 745 24 812 504 705 954 792 270 674 724 497 653 742 406 916 953 474 654 776

K00333 412 684 538 216 695 582 331 872 684 694 947 882 358 854 596 294 818 1160 690 760 982 976 1212 855 462 441 451 300 420 914 24 958 598 778 1011 858 270 704 826 546 784 830 482 1067 1065 514 729 904

K00334 394 620 503 216 570 468 340 708 657 694 886 882 358 732 548 247 742 1066 690 699 883 941 1072 804 438 441 440 238 409 745 24 812 504 730 954 792 270 674 724 497 653 751 406 916 953 474 654 776

K00335 394 620 503 216 570 468 340 708 657 694 886 882 358 732 548 247 742 1066 690 699 883 941 1072 804 438 441 440 238 409 745 24 812 504 730 954 792 270 674 724 497 653 751 406 916 953 474 654 776

K00336 0 0 0 0 0 0 0 0 0 0 0 0 0 0 0 0 0 0 0 0 0 0 0 0 0 0 0 0 0 0 0 0 0 0 0 0 0 0 0 0 0 0 0 0 0 0 0 0

K00337 412 684 538 216 695 582 331 872 684 694 947 882 358 854 596 294 818 1160 690 760 982 976 1212 855 462 441 451 300 420 914 24 958 598 778 1011 858 270 704 826 546 784 830 482 1067 1065 514 729 904

K00338 412 684 538 216 695 582 331 872 684 694 947 882 358 854 596 294 818 1160 690 760 982 976 1212 855 462 441 451 300 420 914 24 958 598 778 1011 858 270 704 826 546 784 830 482 1067 1065 514 729 904

K00339 380 508 398 192 490 418 306 626 592 630 848 778 323 702 435 247 608 972 666 588 789 916 1058 756 417 441 429 238 380 614 24 736 451 682 840 758 270 644 598 375 478 586 345 706 770 436 548 722

K00340 338 420 344 166 406 320 267 564 552 574 661 728 268 601 372 202 508 856 526 476 648 784 838 582 354 338 346 218 292 502 24 648 436 638 772 654 212 438 429 340 452 498 327 580 572 350 470 533

K00341 370 484 379 166 536 435 267 727 578 574 722 728 268 722 436 248 584 951 526 555 748 820 978 646 377 338 358 280 304 672 24 794 530 711 829 720 212 469 531 389 582 586 402 730 684 390 545 660

K00342 412 684 538 216 695 582 331 872 684 694 947 882 358 854 596 294 818 1160 690 760 982 976 1212 855 462 441 451 300 420 914 24 958 598 778 1011 858 270 704 826 546 784 830 482 1067 1065 514 729 904

K00343 338 420 344 166 406 320 267 564 552 574 661 728 268 601 372 202 508 856 526 476 648 784 838 582 354 338 346 218 292 502 24 648 436 638 772 654 212 438 429 340 452 498 327 580 572 350 470 533

K00344 0 0 0 0 0 0 0 0 0 0 0 0 0 0 0 0 0 0 0 0 0 0 0 0 0 0 0 0 0 0 0 0 0 0 0 0 0 0 0 0 0 0 0 0 0 0 0 0

K00346 0 0 0 0 0 0 0 0 0 0 0 0 0 0 0 0 0 0 0 0 0 0 0 0 0 0 0 0 0 0 0 0 0 0 0 0 0 0 0 0 0 0 0 0 0 0 0 0

K00347 0 0 0 0 0 0 0 0 0 0 0 0 0 0 0 0 0 0 0 0 0 0 0 0 0 0 0 0 0 0 0 0 0 0 0 0 0 0 0 0 0 0 0 0 0 0 0 0

K00348 0 0 0 0 0 0 0 0 0 0 0 0 0 0 0 0 0 0 0 0 0 0 0 0 0 0 0 0 0 0 0 0 0 0 0 0 0 0 0 0 0 0 0 0 0 0 0 0

K00349 0 0 0 0 0 0 0 0 0 0 0 0 0 0 0 0 0 0 0 0 0 0 0 0 0 0 0 0 0 0 0 0 0 0 0 0 0 0 0 0 0 0 0 0 0 0 0 0

K00350 0 0 0 0 0 0 0 0 0 0 0 0 0 0 0 0 0 0 0 0 0 0 0 0 0 0 0 0 0 0 0 0 0 0 0 0 0 0 0 0 0 0 0 0 0 0 0 0

K00351 0 0 0 0 0 0 0 0 0 0 0 0 0 0 0 0 0 0 0 0 0 0 0 0 0 0 0 0 0 0 0 0 0 0 0 0 0 0 0 0 0 0 0 0 0 0 0 0

K00353 0 0 0 0 0 0 0 0 0 0 0 0 0 0 0 0 0 0 0 0 0 0 0 0 0 0 0 0 0 0 0 0 0 0 0 0 0 0 0 0 0 0 0 0 0 0 0 0

K00354 0 0 0 0 0 0 0 0 0 0 0 0 0 0 0 0 0 0 0 0 0 0 0 0 0 0 0 0 0 0 0 0 0 0 0 0 0 0 0 0 0 0 0 0 0 0 0 0

K00355 0 0 0 0 0 0 0 0 0 0 0 0 0 0 0 0 0 0 0 0 0 0 0 0 0 0 0 0 0 0 0 0 0 0 0 0 0 0 0 0 0 0 0 0 0 0 0 0

K00356 0 0 0 0 0 0 0 0 0 0 0 0 0 0 0 0 0 0 0 0 0 0 0 0 0 0 0 0 0 0 0 0 0 0 0 0 0 0 0 0 0 0 0 0 0 0 0 0

K00358 14 0 0 0 0 0 0 0 0 0 0 0 0 0 0 0 0 0 0 18 0 0 0 0 0 0 0 0 0 0 0 4 0 24 0 0 0 0 0 0 0 0 0 0 0 0 0 0

K00359 0 0 0 0 0 0 0 0 0 0 0 0 0 0 0 0 0 0 0 0 0 0 0 0 0 0 0 0 0 0 0 0 0 0 0 0 0 0 0 0 0 0 0 0 0 0 0 0

K00360 0 0 0 0 0 0 0 0 0 0 0 0 0 0 0 0 0 0 0 0 0 0 0 0 0 0 0 0 0 0 0 0 0 0 0 0 0 0 0 0 0 0 0 0 0 0 0 0

K00362 0 0 0 0 0 0 0 0 0 0 0 0 0 0 0 0 0 0 0 0 0 0 0 0 0 0 0 0 0 0 0 0 0 0 0 0 0 0 0 0 0 0 0 0 0 0 0 0

K00363 0 0 0 0 0 0 0 0 0 0 0 0 0 0 0 0 0 0 0 0 0 0 0 0 0 0 0 0 0 0 0 0 0 0 0 0 0 0 0 0 0 0 0 0 0 0 0 0

K00364 0 0 0 0 0 0 0 0 0 0 0 0 0 0 0 0 0 0 0 0 0 0 0 0 0 0 0 0 0 0 0 0 0 0 0 0 0 0 0 0 0 0 0 0 0 0 0 0

K00365 0 0 0 0 0 0 0 0 0 0 0 0 0 0 0 0 0 0 0 0 0 0 0 0 0 0 0 0 0 0 0 0 0 0 0 0 0 0 0 0 0 0 0 0 0 0 0 0

K00366 0 0 0 0 0 0 0 0 0 0 0 0 0 0 0 0 0 0 0 0 0 0 0 0 0 0 0 0 0 0 0 0 0 0 0 0 0 0 0 0 0 0 0 0 0 0 0 0

K00367 0 0 0 0 0 0 0 0 0 0 0 0 0 0 0 0 0 0 0 0 0 0 0 0 0 0 0 0 0 0 0 0 0 0 0 0 0 0 0 0 0 0 0 0 0 0 0 0

K00368 0 0 0 0 0 0 0 0 0 0 0 0 0 0 0 0 0 0 0 0 0 0 0 0 0 0 0 0 0 0 0 0 0 0 0 0 0 0 0 0 0 0 0 0 0 0 0 0

K00369 0 0 0 0 0 0 0 0 0 0 0 0 0 0 0 0 0 0 0 0 0 0 0 0 0 0 0 0 0 0 0 0 0 0 0 0 0 0 0 0 0 0 0 0 0 0 0 0

K00370 0 0 0 0 0 0 0 0 0 0 0 0 0 0 0 0 0 0 0 0 0 0 0 0 0 0 0 0 0 0 0 0 0 0 0 0 0 0 0 0 0 0 0 0 0 0 0 0

K00371 0 0 0 0 0 0 0 0 0 0 0 0 0 0 0 0 0 0 0 0 0 0 0 0 0 0 0 0 0 0 0 0 0 0 0 0 0 0 0 0 0 0 0 0 0 0 0 0

K00372 0 0 0 0 0 0 0 0 0 0 0 0 0 0 0 0 0 0 0 0 0 0 0 0 0 0 0 0 0 0 0 0 0 0 0 0 0 0 0 0 0 0 0 0 0 0 0 0

K00373 0 0 0 0 0 0 0 0 0 0 0 0 0 0 0 0 0 0 0 0 0 0 0 0 0 0 0 0 0 0 0 0 0 0 0 0 0 0 0 0 0 0 0 0 0 0 0 0

K00374 0 0 0 0 0 0 0 0 0 0 0 0 0 0 0 0 0 0 0 0 0 0 0 0 0 0 0 0 0 0 0 0 0 0 0 0 0 0 0 0 0 0 0 0 0 0 0 0

K00375 0 0 0 0 0 0 0 0 0 0 0 0 0 0 0 0 0 0 0 0 0 0 0 0 0 0 0 0 0 0 0 0 0 0 0 0 0 0 0 0 0 0 0 0 0 0 0 0

K00376 0 0 0 0 0 0 0 0 0 0 0 0 0 0 0 0 0 0 0 0 0 0 0 0 0 0 0 0 0 0 0 0 0 0 0 0 0 0 0 0 0 0 0 0 0 0 0 0

K00378 66 40 107 47 260 200 72 338 84 124 195 86 28 72 124 27 101 208 59 62 106 150 124 202 12 30 21 9 38 278 0 80 50 88 83 77 37 54 90 122 168 154 28 103 96 144 59 230

K00380 0 0 0 0 0 0 0 0 0 0 0 0 0 0 0 0 0 0 0 0 0 0 0 0 0 0 0 0 0 0 0 0 0 0 0 0 0 0 0 0 0 0 0 0 0 0 0 0

K00381 0 0 0 0 0 0 0 0 0 0 0 0 0 0 0 0 0 0 0 0 0 0 0 0 0 0 0 0 0 0 0 0 0 0 0 0 0 0 0 0 0 0 0 0 0 0 0 0

K00382 478 724 645 262 978 798 404 1219 768 818 1142 968 386 926 719 320 919 1382 748 822 1088 1127 1335 1064 473 471 472 308 458 1206 24 1038 648 866 1094 934 308 759 916 668 952 984 510 1170 1161 658 788 1134

K00383 33 18 73 32 228 170 52 308 40 98 154 45 18 37 78 20 89 181 48 14 66 133 84 136 1 26 13 0 21 222 0 5 28 42 40 28 28 24 66 106 152 105 0 78 72 140 43 173

K00384 478 724 645 262 954 782 404 1210 768 818 1139 968 386 926 719 320 919 1368 748 822 1088 1127 1335 1040 473 471 472 308 458 1188 24 1008 648 866 1094 934 308 759 916 668 952 984 510 1170 1161 658 788 1134

K00385 0 0 0 0 0 0 0 0 0 0 0 0 0 0 0 0 0 0 0 0 0 0 0 0 0 0 0 0 0 0 0 0 0 0 0 0 0 0 0 0 0 0 0 0 0 0 0 0

K00386 0 0 0 0 0 0 0 0 0 0 0 0 0 0 0 0 0 0 0 0 0 0 0 0 0 0 0 0 0 0 0 0 0 0 0 0 0 0 0 0 0 0 0 0 0 0 0 0

K00387 0 0 0 0 0 0 0 0 0 0 0 0 0 0 0 0 0 0 0 0 0 0 0 0 0 0 0 0 0 0 0 0 0 0 0 0 0 0 0 0 0 0 0 0 0 0 0 0

K00389 0 0 0 0 0 0 0 0 0 0 0 0 0 0 0 0 0 0 0 0 0 0 0 0 0 0 0 0 0 0 0 0 0 0 0 0 0 0 0 0 0 0 0 0 0 0 0 0

K00390 412 684 538 216 719 598 331 880 684 694 950 882 358 854 596 294 818 1174 690 760 982 976 1212 878 462 441 451 300 420 932 24 992 598 778 1011 858 270 704 826 546 784 830 482 1067 1065 514 729 904

K00392 0 0 0 0 0 0 0 0 0 0 0 0 0 0 0 0 0 0 0 0 0 0 0 0 0 0 0 0 0 0 0 0 0 0 0 0 0 0 0 0 0 0 0 0 0 0 0 0

K00394 0 0 0 0 0 0 0 0 0 0 0 0 0 0 0 0 0 0 0 0 0 0 0 0 0 0 0 0 0 0 0 0 0 0 0 0 0 0 0 0 0 0 0 0 0 0 0 0

K00395 0 0 0 0 0 0 0 0 0 0 0 0 0 0 0 0 0 0 0 0 0 0 0 0 0 0 0 0 0 0 0 0 0 0 0 0 0 0 0 0 0 0 0 0 0 0 0 0

K00397 0 0 0 0 0 0 0 0 0 0 0 0 0 0 0 0 0 0 0 0 0 0 0 0 0 0 0 0 0 0 0 0 0 0 0 0 0 0 0 0 0 0 0 0 0 0 0 0

K00399 113 104 142 47 443 346 81 520 110 124 256 86 28 194 204 74 176 331 59 160 204 186 263 310 35 30 32 71 49 478 0 256 144 186 140 143 37 85 192 171 298 250 103 254 208 184 134 358

K00400 166 144 249 94 672 530 145 850 194 249 448 171 56 266 311 100 278 525 118 204 310 336 386 459 46 60 53 80 87 735 0 277 195 249 222 220 74 140 281 293 466 395 131 356 304 327 192 588

K00401 113 104 142 47 443 346 81 520 110 124 256 86 28 194 204 74 176 331 59 160 204 186 263 310 35 30 32 71 49 478 0 256 144 186 140 143 37 85 192 171 298 250 103 254 208 184 134 358

K00402 113 104 142 47 443 346 81 520 110 124 256 86 28 194 204 74 176 331 59 160 204 186 263 310 35 30 32 71 49 478 0 256 144 186 140 143 37 85 192 171 298 250 103 254 208 184 134 358

K00404 0 0 0 0 0 0 0 0 0 0 0 0 0 0 0 0 0 0 0 0 0 0 0 0 0 0 0 0 0 0 0 0 0 0 0 0 0 0 0 0 0 0 0 0 0 0 0 0

K00405 0 0 0 0 0 0 0 0 0 0 0 0 0 0 0 0 0 0 0 0 0 0 0 0 0 0 0 0 0 0 0 0 0 0 0 0 0 0 0 0 0 0 0 0 0 0 0 0

K00406 0 0 0 0 0 0 0 0 0 0 0 0 0 0 0 0 0 0 0 0 0 0 0 0 0 0 0 0 0 0 0 0 0 0 0 0 0 0 0 0 0 0 0 0 0 0 0 0

K00407 0 0 0 0 0 0 0 0 0 0 0 0 0 0 0 0 0 0 0 0 0 0 0 0 0 0 0 0 0 0 0 0 0 0 0 0 0 0 0 0 0 0 0 0 0 0 0 0

K00411 0 0 0 0 0 0 0 0 0 0 0 0 0 0 0 0 0 0 0 0 0 0 0 0 0 0 0 0 0 0 0 0 0 0 0 0 0 0 0 0 0 0 0 0 0 0 0 0

K00412 0 0 0 0 0 0 0 0 0 0 0 0 0 0 0 0 0 0 0 0 0 0 0 0 0 0 0 0 0 0 0 0 0 0 0 0 0 0 0 0 0 0 0 0 0 0 0 0

K00413 0 0 0 0 0 0 0 0 0 0 0 0 0 0 0 0 0 0 0 0 0 0 0 0 0 0 0 0 0 0 0 0 0 0 0 0 0 0 0 0 0 0 0 0 0 0 0 0

K00421 0 0 0 0 0 0 0 0 0 0 0 0 0 0 0 0 0 0 0 0 0 0 0 0 0 0 0 0 0 0 0 0 0 0 0 0 0 0 0 0 0 0 0 0 0 0 0 0

K00423 0 0 0 0 0 0 0 0 0 0 0 0 0 0 0 0 0 0 0 0 0 0 0 0 0 0 0 0 0 0 0 0 0 0 0 0 0 0 0 0 0 0 0 0 0 0 0 0

K00425 370 484 379 166 536 435 267 727 578 574 722 728 268 722 436 248 584 951 526 555 748 820 978 646 377 338 358 280 304 672 24 794 530 711 829 720 212 469 531 389 582 586 402 730 684 390 545 660

K00426 0 0 0 0 0 0 0 0 0 0 0 0 0 0 0 0 0 0 0 0 0 0 0 0 0 0 0 0 0 0 0 0 0 0 0 0 0 0 0 0 0 0 0 0 0 0 0 0

K00427 0 0 0 0 0 0 0 0 0 0 0 0 0 0 0 0 0 0 0 0 0 0 0 0 0 0 0 0 0 0 0 0 0 0 0 0 0 0 0 0 0 0 0 0 0 0 0 0

K00428 0 0 0 0 0 0 0 0 0 0 0 0 0 0 0 0 0 0 0 0 0 0 0 0 0 0 0 0 0 0 0 0 0 0 0 0 0 0 0 0 0 0 0 0 0 0 0 0

K00429 0 0 0 0 0 0 0 0 0 0 0 0 0 0 0 0 0 0 0 0 0 0 0 0 0 0 0 0 0 0 0 0 0 0 0 0 0 0 0 0 0 0 0 0 0 0 0 0

K00430 0 0 0 0 0 0 0 0 0 0 0 0 0 0 0 0 0 0 0 0 0 0 0 0 0 0 0 0 0 0 0 0 0 0 0 0 0 0 0 0 0 0 0 0 0 0 0 0

K00432 33 18 73 32 205 154 52 298 40 98 154 45 18 37 78 20 89 167 48 14 66 133 84 130 1 26 13 0 21 209 0 5 28 42 40 28 28 24 66 106 152 105 0 78 72 140 43 173

K00433 0 0 0 0 0 0 0 0 0 0 0 0 0 0 0 0 0 0 0 0 0 0 0 0 0 0 0 0 0 0 0 0 0 0 0 0 0 0 0 0 0 0 0 0 0 0 0 0

K00435 0 0 0 0 0 0 0 0 0 0 0 0 0 0 0 0 0 0 0 0 0 0 0 0 0 0 0 0 0 0 0 0 0 0 0 0 0 0 0 0 0 0 0 0 0 0 0 0

K00436 0 0 0 0 0 0 0 0 0 0 0 0 0 0 0 0 0 0 0 0 0 0 0 0 0 0 0 0 0 0 0 0 0 0 0 0 0 0 0 0 0 0 0 0 0 0 0 0

K00437 0 0 0 0 0 0 0 0 0 0 0 0 0 0 0 0 0 0 0 0 0 0 0 0 0 0 0 0 0 0 0 0 0 0 0 0 0 0 0 0 0 0 0 0 0 0 0 0

K00438 0 0 0 0 0 0 0 0 0 0 0 0 0 0 0 0 0 0 0 0 0 0 0 0 0 0 0 0 0 0 0 0 0 0 0 0 0 0 0 0 0 0 0 0 0 0 0 0

K00440 66 40 107 47 284 216 72 348 84 124 195 86 28 72 124 27 101 222 59 62 106 150 124 208 12 30 21 9 38 292 0 80 50 88 83 77 37 54 90 122 168 154 28 103 96 144 59 230

K00441 518 564 593 260 1114 866 429 1422 746 823 1111 900 324 866 715 302 786 1395 644 698 958 1121 1225 1090 400 398 399 298 380 1254 24 958 631 912 995 874 286 578 710 633 918 910 458 936 876 676 663 1122

K00442 66 40 107 47 282 216 72 348 84 124 195 86 28 72 124 27 101 222 59 62 106 150 124 208 12 30 21 9 38 292 0 80 50 88 83 77 37 54 90 122 168 154 28 103 96 144 59 230

K00443 66 40 107 47 282 216 72 348 84 124 195 86 28 72 124 27 101 222 59 62 106 150 124 208 12 30 21 9 38 292 0 80 50 88 83 77 37 54 90 122 168 154 28 103 96 144 59 230

K00446 0 0 0 0 0 0 0 0 0 0 0 0 0 0 0 0 0 0 0 0 0 0 0 0 0 0 0 0 0 0 0 0 0 0 0 0 0 0 0 0 0 0 0 0 0 0 0 0

K00448 0 0 0 0 0 0 0 0 0 0 0 0 0 0 0 0 0 0 0 0 0 0 0 0 0 0 0 0 0 0 0 0 0 0 0 0 0 0 0 0 0 0 0 0 0 0 0 0

K00449 0 0 0 0 0 0 0 0 0 0 0 0 0 0 0 0 0 0 0 0 0 0 0 0 0 0 0 0 0 0 0 0 0 0 0 0 0 0 0 0 0 0 0 0 0 0 0 0

K00450 0 0 0 0 0 0 0 0 0 0 0 0 0 0 0 0 0 0 0 0 0 0 0 0 0 0 0 0 0 0 0 0 0 0 0 0 0 0 0 0 0 0 0 0 0 0 0 0

K00451 0 0 0 0 0 0 0 0 0 0 0 0 0 0 0 0 0 0 0 0 0 0 0 0 0 0 0 0 0 0 0 0 0 0 0 0 0 0 0 0 0 0 0 0 0 0 0 0

K00452 0 0 0 0 0 0 0 0 0 0 0 0 0 0 0 0 0 0 0 0 0 0 0 0 0 0 0 0 0 0 0 0 0 0 0 0 0 0 0 0 0 0 0 0 0 0 0 0

K00453 0 0 0 0 0 0 0 0 0 0 0 0 0 0 0 0 0 0 0 0 0 0 0 0 0 0 0 0 0 0 0 0 0 0 0 0 0 0 0 0 0 0 0 0 0 0 0 0

K00455 0 0 0 0 0 0 0 0 0 0 0 0 0 0 0 0 0 0 0 0 0 0 0 0 0 0 0 0 0 0 0 0 0 0 0 0 0 0 0 0 0 0 0 0 0 0 0 0

K00456 0 0 0 0 0 0 0 0 0 0 0 0 0 0 0 0 0 0 0 0 0 0 0 0 0 0 0 0 0 0 0 0 0 0 0 0 0 0 0 0 0 0 0 0 0 0 0 0

K00457 0 0 0 0 0 0 0 0 0 0 0 0 0 0 0 0 0 0 0 0 0 0 0 0 0 0 0 0 0 0 0 0 0 0 0 0 0 0 0 0 0 0 0 0 0 0 0 0

K00459 0 0 0 0 0 0 0 0 0 0 0 0 0 0 0 0 0 0 0 0 0 0 0 0 0 0 0 0 0 0 0 0 0 0 0 0 0 0 0 0 0 0 0 0 0 0 0 0

K00460 0 0 0 0 0 0 0 0 0 0 0 0 0 0 0 0 0 0 0 0 0 0 0 0 0 0 0 0 0 0 0 0 0 0 0 0 0 0 0 0 0 0 0 0 0 0 0 0

K00461 0 0 0 0 0 0 0 0 0 0 0 0 0 0 0 0 0 0 0 0 0 0 0 0 0 0 0 0 0 0 0 0 0 0 0 0 0 0 0 0 0 0 0 0 0 0 0 0

K00462 0 0 0 0 0 0 0 0 0 0 0 0 0 0 0 0 0 0 0 0 0 0 0 0 0 0 0 0 0 0 0 0 0 0 0 0 0 0 0 0 0 0 0 0 0 0 0 0

K00463 0 0 0 0 0 0 0 0 0 0 0 0 0 0 0 0 0 0 0 0 0 0 0 0 0 0 0 0 0 0 0 0 0 0 0 0 0 0 0 0 0 0 0 0 0 0 0 0

K00464 0 0 0 0 0 0 0 0 0 0 0 0 0 0 0 0 0 0 0 0 0 0 0 0 0 0 0 0 0 0 0 0 0 0 0 0 0 0 0 0 0 0 0 0 0 0 0 0

K00466 0 0 0 0 0 0 0 0 0 0 0 0 0 0 0 0 0 0 0 0 0 0 0 0 0 0 0 0 0 0 0 0 0 0 0 0 0 0 0 0 0 0 0 0 0 0 0 0

K00467 0 0 0 0 0 0 0 0 0 0 0 0 0 0 0 0 0 0 0 0 0 0 0 0 0 0 0 0 0 0 0 0 0 0 0 0 0 0 0 0 0 0 0 0 0 0 0 0

K00468 0 0 0 0 0 0 0 0 0 0 0 0 0 0 0 0 0 0 0 0 0 0 0 0 0 0 0 0 0 0 0 0 0 0 0 0 0 0 0 0 0 0 0 0 0 0 0 0

K00469 0 0 0 0 0 0 0 0 0 0 0 0 0 0 0 0 0 0 0 0 0 0 0 0 0 0 0 0 0 0 0 0 0 0 0 0 0 0 0 0 0 0 0 0 0 0 0 0

K00470 0 0 0 0 0 0 0 0 0 0 0 0 0 0 0 0 0 0 0 0 0 0 0 0 0 0 0 0 0 0 0 0 0 0 0 0 0 0 0 0 0 0 0 0 0 0 0 0

K00471 0 0 0 0 0 0 0 0 0 0 0 0 0 0 0 0 0 0 0 0 0 0 0 0 0 0 0 0 0 0 0 0 0 0 0 0 0 0 0 0 0 0 0 0 0 0 0 0

K00472 0 0 0 0 0 0 0 0 0 0 0 0 0 0 0 0 0 0 0 0 0 0 0 0 0 0 0 0 0 0 0 0 0 0 0 0 0 0 0 0 0 0 0 0 0 0 0 0

K00474 0 0 0 0 0 0 0 0 0 0 0 0 0 0 0 0 0 0 0 0 0 0 0 0 0 0 0 0 0 0 0 0 0 0 0 0 0 0 0 0 0 0 0 0 0 0 0 0

K00476 0 0 0 0 0 0 0 0 0 0 0 0 0 0 0 0 0 0 0 0 0 0 0 0 0 0 0 0 0 0 0 0 0 0 0 0 0 0 0 0 0 0 0 0 0 0 0 0

K00477 0 0 0 0 0 0 0 0 0 0 0 0 0 0 0 0 0 0 0 0 0 0 0 0 0 0 0 0 0 0 0 0 0 0 0 0 0 0 0 0 0 0 0 0 0 0 0 0

K00478 0 0 0 0 0 0 0 0 0 0 0 0 0 0 0 0 0 0 0 0 0 0 0 0 0 0 0 0 0 0 0 0 0 0 0 0 0 0 0 0 0 0 0 0 0 0 0 0

K00479 0 0 0 0 0 0 0 0 0 0 0 0 0 0 0 0 0 0 0 0 0 0 0 0 0 0 0 0 0 0 0 0 0 0 0 0 0 0 0 0 0 0 0 0 0 0 0 0

K00480 0 0 0 0 0 0 0 0 0 0 0 0 0 0 0 0 0 0 0 0 0 0 0 0 0 0 0 0 0 0 0 0 0 0 0 0 0 0 0 0 0 0 0 0 0 0 0 0

K00481 0 0 0 0 0 0 0 0 0 0 0 0 0 0 0 0 0 0 0 0 0 0 0 0 0 0 0 0 0 0 0 0 0 0 0 0 0 0 0 0 0 0 0 0 0 0 0 0

K00483 0 0 0 0 0 0 0 0 0 0 0 0 0 0 0 0 0 0 0 0 0 0 0 0 0 0 0 0 0 0 0 0 0 0 0 0 0 0 0 0 0 0 0 0 0 0 0 0

K00484 0 0 0 0 0 0 0 0 0 0 0 0 0 0 0 0 0 0 0 0 0 0 0 0 0 0 0 0 0 0 0 0 0 0 0 0 0 0 0 0 0 0 0 0 0 0 0 0

K00485 0 0 0 0 0 0 0 0 0 0 0 0 0 0 0 0 0 0 0 0 0 0 0 0 0 0 0 0 0 0 0 0 0 0 0 0 0 0 0 0 0 0 0 0 0 0 0 0

K00486 0 0 0 0 0 0 0 0 0 0 0 0 0 0 0 0 0 0 0 0 0 0 0 0 0 0 0 0 0 0 0 0 0 0 0 0 0 0 0 0 0 0 0 0 0 0 0 0

K00491 0 0 0 0 0 0 0 0 0 0 0 0 0 0 0 0 0 0 0 0 0 0 0 0 0 0 0 0 0 0 0 0 0 0 0 0 0 0 0 0 0 0 0 0 0 0 0 0

K00492 0 0 0 0 0 0 0 0 0 0 0 0 0 0 0 0 0 0 0 0 0 0 0 0 0 0 0 0 0 0 0 0 0 0 0 0 0 0 0 0 0 0 0 0 0 0 0 0

K00493 0 0 0 0 0 0 0 0 0 0 0 0 0 0 0 0 0 0 0 0 0 0 0 0 0 0 0 0 0 0 0 0 0 0 0 0 0 0 0 0 0 0 0 0 0 0 0 0

K00494 0 0 0 0 0 0 0 0 0 0 0 0 0 0 0 0 0 0 0 0 0 0 0 0 0 0 0 0 0 0 0 0 0 0 0 0 0 0 0 0 0 0 0 0 0 0 0 0

K00495 0 0 0 0 0 0 0 0 0 0 0 0 0 0 0 0 0 0 0 0 0 0 0 0 0 0 0 0 0 0 0 0 0 0 0 0 0 0 0 0 0 0 0 0 0 0 0 0

K00496 0 0 0 0 0 0 0 0 0 0 0 0 0 0 0 0 0 0 0 0 0 0 0 0 0 0 0 0 0 0 0 0 0 0 0 0 0 0 0 0 0 0 0 0 0 0 0 0

K00499 0 0 0 0 0 0 0 0 0 0 0 0 0 0 0 0 0 0 0 0 0 0 0 0 0 0 0 0 0 0 0 0 0 0 0 0 0 0 0 0 0 0 0 0 0 0 0 0

K00500 0 0 0 0 0 0 0 0 0 0 0 0 0 0 0 0 0 0 0 0 0 0 0 0 0 0 0 0 0 0 0 0 0 0 0 0 0 0 0 0 0 0 0 0 0 0 0 0

K00504 0 0 0 0 0 0 0 0 0 0 0 0 0 0 0 0 0 0 0 0 0 0 0 0 0 0 0 0 0 0 0 0 0 0 0 0 0 0 0 0 0 0 0 0 0 0 0 0

K00505 0 0 0 0 0 0 0 0 0 0 0 0 0 0 0 0 0 0 0 0 0 0 0 0 0 0 0 0 0 0 0 0 0 0 0 0 0 0 0 0 0 0 0 0 0 0 0 0

K00507 0 0 0 0 0 0 0 0 0 0 0 0 0 0 0 0 0 0 0 0 0 0 0 0 0 0 0 0 0 0 0 0 0 0 0 0 0 0 0 0 0 0 0 0 0 0 0 0

K00508 0 0 0 0 0 0 0 0 0 0 0 0 0 0 0 0 0 0 0 0 0 0 0 0 0 0 0 0 0 0 0 0 0 0 0 0 0 0 0 0 0 0 0 0 0 0 0 0

K00510 0 0 0 0 0 0 0 0 0 0 0 0 0 0 0 0 0 0 0 0 0 0 0 0 0 0 0 0 0 0 0 0 0 0 0 0 0 0 0 0 0 0 0 0 0 0 0 0

K00511 0 0 0 0 0 0 0 0 0 0 0 0 0 0 0 0 0 0 0 0 0 0 0 0 0 0 0 0 0 0 0 0 0 0 0 0 0 0 0 0 0 0 0 0 0 0 0 0

K00514 0 0 0 0 0 0 0 0 0 0 0 0 0 0 0 0 0 0 0 0 0 0 0 0 0 0 0 0 0 0 0 0 0 0 0 0 0 0 0 0 0 0 0 0 0 0 0 0

K00515 0 0 0 0 0 0 0 0 0 0 0 0 0 0 0 0 0 0 0 0 0 0 0 0 0 0 0 0 0 0 0 0 0 0 0 0 0 0 0 0 0 0 0 0 0 0 0 0

K00517 0 0 0 0 0 0 0 0 0 0 0 0 0 0 0 0 0 0 0 0 0 0 0 0 0 0 0 0 0 0 0 0 0 0 0 0 0 0 0 0 0 0 0 0 0 0 0 0

K00518 0 0 0 0 0 0 0 0 0 0 0 0 0 0 0 0 0 0 0 0 0 0 0 0 0 0 0 0 0 0 0 0 0 0 0 0 0 0 0 0 0 0 0 0 0 0 0 0

K00519 0 0 0 0 0 0 0 0 0 0 0 0 0 0 0 0 0 0 0 0 0 0 0 0 0 0 0 0 0 0 0 0 0 0 0 0 0 0 0 0 0 0 0 0 0 0 0 0

K00520 0 0 0 0 1 0 0 0 0 0 0 0 0 0 0 0 0 0 0 0 0 0 0 0 0 0 0 0 0 0 0 0 0 0 0 0 0 0 0 0 0 0 0 0 0 0 0 0

K00522 0 0 0 0 0 0 0 0 0 0 0 0 0 0 0 0 0 0 0 0 0 0 0 0 0 0 0 0 0 0 0 0 0 0 0 0 0 0 0 0 0 0 0 0 0 0 0 0

K00523 0 0 0 0 0 0 0 0 0 0 0 0 0 0 0 0 0 0 0 0 0 0 0 0 0 0 0 0 0 0 0 0 0 0 0 0 0 0 0 0 0 0 0 0 0 0 0 0

K00524 0 0 0 0 0 0 0 0 0 0 0 0 0 0 0 0 0 0 0 0 0 0 0 0 0 0 0 0 0 0 0 0 0 0 0 0 0 0 0 0 0 0 0 0 0 0 0 0

K00525 468 884 697 264 884 745 404 1025 789 813 1175 1036 450 986 771 339 1052 1384 854 984 1216 1133 1445 1100 546 544 545 318 536 1175 24 1152 666 870 1193 994 329 940 1121 703 984 1083 561 1404 1446 640 913 1148

K00526 0 0 0 0 0 0 0 0 0 0 0 0 0 0 0 0 0 0 0 0 0 0 0 0 0 0 0 0 0 0 0 0 0 0 0 0 0 0 0 0 0 0 0 0 0 0 0 0

K00527 478 724 645 262 978 798 404 1219 768 818 1142 968 386 926 719 320 919 1382 748 822 1088 1127 1335 1064 473 471 472 308 458 1206 24 1038 648 866 1094 934 308 759 916 668 952 984 510 1170 1161 658 788 1134

K00528 42 200 159 49 182 163 64 154 106 120 228 154 91 132 160 46 234 224 164 205 234 156 234 232 84 102 94 19 116 260 0 194 68 67 182 137 58 236 295 157 201 244 80 337 382 125 184 244

K00529 0 0 0 0 0 0 0 0 0 0 0 0 0 0 0 0 0 0 0 0 0 0 0 0 0 0 0 0 0 0 0 0 0 0 0 0 0 0 0 0 0 0 0 0 0 0 0 0

K00530 0 0 0 0 0 0 0 0 0 0 0 0 0 0 0 0 0 0 0 0 0 0 0 0 0 0 0 0 0 0 0 0 0 0 0 0 0 0 0 0 0 0 0 0 0 0 0 0

K00531 0 0 0 0 0 0 0 0 0 0 0 0 0 0 0 0 0 0 0 0 0 0 0 0 0 0 0 0 0 0 0 0 0 0 0 0 0 0 0 0 0 0 0 0 0 0 0 0

K00532 0 0 0 0 0 0 0 0 0 0 0 0 0 0 0 0 0 0 0 0 0 0 0 0 0 0 0 0 0 0 0 0 0 0 0 0 0 0 0 0 0 0 0 0 0 0 0 0

K00533 0 0 0 0 0 0 0 0 0 0 0 0 0 0 0 0 0 0 0 0 0 0 0 0 0 0 0 0 0 0 0 0 0 0 0 0 0 0 0 0 0 0 0 0 0 0 0 0

K00534 0 0 0 0 0 0 0 0 0 0 0 0 0 0 0 0 0 0 0 0 0 0 0 0 0 0 0 0 0 0 0 0 0 0 0 0 0 0 0 0 0 0 0 0 0 0 0 0

K00535 0 0 0 0 24 16 0 9 0 0 0 0 0 0 0 0 0 14 0 0 0 0 0 23 0 0 0 0 0 18 0 30 0 0 0 0 0 0 0 0 0 0 0 0 0 0 0 0

K00536 0 0 0 0 0 0 0 0 0 0 0 0 0 0 0 0 0 0 0 0 0 0 0 0 0 0 0 0 0 0 0 0 0 0 0 0 0 0 0 0 0 0 0 0 0 0 0 0

K00537 33 18 73 32 205 154 52 298 40 98 154 45 18 37 78 20 89 167 48 14 66 133 84 130 1 26 13 0 21 209 0 5 28 42 40 28 28 24 66 106 152 105 0 78 72 140 43 173

K00538 0 0 0 0 0 0 0 0 0 0 0 0 0 0 0 0 0 0 0 0 0 0 0 0 0 0 0 0 0 0 0 0 0 0 0 0 0 0 0 0 0 0 0 0 0 0 0 0

K00539 0 0 0 0 0 0 0 0 0 0 0 0 0 0 0 0 0 0 0 0 0 0 0 0 0 0 0 0 0 0 0 0 0 0 0 0 0 0 0 0 0 0 0 0 0 0 0 0

K00540 440 684 538 216 732 598 348 880 684 694 947 882 358 854 628 294 818 1174 690 797 982 976 1212 905 462 441 451 300 420 932 24 988 598 827 1011 858 270 704 826 546 784 848 482 1067 1065 514 729 904

K00542 0 0 0 0 0 0 0 0 0 0 0 0 0 0 0 0 0 0 0 0 0 0 0 0 0 0 0 0 0 0 0 0 0 0 0 0 0 0 0 0 0 0 0 0 0 0 0 0

K00543 0 0 0 0 0 0 0 0 0 0 0 0 0 0 0 0 0 0 0 0 0 0 0 0 0 0 0 0 0 0 0 0 0 0 0 0 0 0 0 0 0 0 0 0 0 0 0 0

K00544 0 0 0 0 0 0 0 0 0 0 0 0 0 0 0 0 0 0 0 0 0 0 0 0 0 0 0 0 0 0 0 0 0 0 0 0 0 0 0 0 0 0 0 0 0 0 0 0

K00545 0 0 0 0 0 0 0 0 0 0 0 0 0 0 0 0 0 0 0 0 0 0 0 0 0 0 0 0 0 0 0 0 0 0 0 0 0 0 0 0 0 0 0 0 0 0 0 0

K00547 0 0 0 0 0 0 0 0 0 0 0 0 0 0 0 0 0 0 0 0 0 0 0 0 0 0 0 0 0 0 0 0 0 0 0 0 0 0 0 0 0 0 0 0 0 0 0 0

K00548 338 420 344 166 406 320 267 564 552 574 661 728 268 601 372 202 508 856 526 476 648 784 838 582 354 338 346 218 292 502 24 648 436 638 772 654 212 438 429 340 452 498 327 580 572 350 470 533

K00549 437 524 486 214 819 650 340 1074 662 698 917 814 296 794 560 275 685 1173 584 618 853 970 1102 855 388 368 378 290 342 963 24 874 580 799 912 798 249 524 620 511 750 740 430 833 780 533 604 891

K00551 0 0 0 0 0 0 0 0 0 0 0 0 0 0 0 0 0 0 0 0 0 0 0 0 0 0 0 0 0 0 0 0 0 0 0 0 0 0 0 0 0 0 0 0 0 0 0 0

K00554 338 420 344 166 406 320 267 564 552 574 661 728 268 601 372 202 508 856 526 476 648 784 838 582 354 338 346 218 292 502 24 648 436 638 772 654 212 438 429 340 452 498 327 580 572 350 470 533

K00555 140 304 301 96 572 477 136 656 216 244 481 240 119 325 347 119 410 526 223 347 439 342 496 482 120 132 126 90 166 704 0 391 212 228 322 280 96 320 486 328 500 486 182 590 590 308 318 602

K00556 0 0 0 0 0 0 0 0 0 0 0 0 0 0 0 0 0 0 0 0 0 0 0 0 0 0 0 0 0 0 0 0 0 0 0 0 0 0 0 0 0 0 0 0 0 0 0 0

K00557 0 0 0 0 0 0 0 0 0 0 0 0 0 0 0 0 0 0 0 0 0 0 0 0 0 0 0 0 0 0 0 0 0 0 0 0 0 0 0 0 0 0 0 0 0 0 0 0

K00558 404 456 490 230 822 628 378 1160 632 771 968 818 304 675 545 242 686 1190 620 504 780 1050 1006 855 356 390 372 218 334 920 24 658 492 723 854 710 269 488 560 552 757 717 327 736 714 630 556 879

K00559 0 0 0 0 0 0 0 0 0 0 0 0 0 0 0 0 0 0 0 0 0 0 0 0 0 0 0 0 0 0 0 0 0 0 0 0 0 0 0 0 0 0 0 0 0 0 0 0

K00560 86 62 141 62 330 261 85 388 128 150 233 126 38 107 152 34 113 263 70 92 146 168 163 250 22 34 28 18 55 356 0 126 73 109 126 126 46 84 114 138 184 194 56 128 120 147 75 288

K00561 0 0 0 0 0 0 0 0 0 0 0 0 0 0 0 0 0 0 0 0 0 0 0 0 0 0 0 0 0 0 0 0 0 0 0 0 0 0 0 0 0 0 0 0 0 0 0 0

K00563 0 0 0 0 0 0 0 0 0 0 0 0 0 0 0 0 0 0 0 0 0 0 0 0 0 0 0 0 0 0 0 0 0 0 0 0 0 0 0 0 0 0 0 0 0 0 0 0

K00564 0 0 0 0 0 0 0 0 0 0 0 0 0 0 0 0 0 0 0 0 0 0 0 0 0 0 0 0 0 0 0 0 0 0 0 0 0 0 0 0 0 0 0 0 0 0 0 0

K00566 338 420 344 166 406 320 267 564 552 574 661 728 268 601 372 202 508 856 526 476 648 784 838 582 354 338 346 218 292 502 24 648 436 638 772 654 212 438 429 340 452 498 327 580 572 350 470 533

K00567 437 524 486 214 819 650 340 1074 662 698 917 814 296 794 560 275 685 1173 584 618 853 970 1102 855 388 368 378 290 342 963 24 874 580 799 912 798 249 524 620 511 750 740 430 833 780 533 604 891

K00568 0 0 0 0 0 0 0 0 0 0 0 0 0 0 0 0 0 0 0 0 0 0 0 0 0 0 0 0 0 0 0 0 0 0 0 0 0 0 0 0 0 0 0 0 0 0 0 0

K00569 0 0 0 0 0 0 0 0 0 0 0 0 0 0 0 0 0 0 0 0 0 0 0 0 0 0 0 0 0 0 0 0 0 0 0 0 0 0 0 0 0 0 0 0 0 0 0 0

K00570 0 0 0 0 0 0 0 0 0 0 0 0 0 0 0 0 0 0 0 0 0 0 0 0 0 0 0 0 0 0 0 0 0 0 0 0 0 0 0 0 0 0 0 0 0 0 0 0

K00571 0 0 0 0 23 16 0 9 0 0 0 0 0 0 0 0 0 14 0 0 0 0 0 6 0 0 0 0 0 13 0 0 0 0 0 0 0 0 0 0 0 0 0 0 0 0 0 0

K00573 426 684 538 216 726 598 340 880 684 694 950 882 358 854 612 294 818 1174 690 778 982 976 1212 892 462 441 451 300 420 932 24 988 598 802 1011 858 270 704 826 546 784 839 482 1067 1065 514 729 904

K00574 0 0 0 0 0 0 0 0 0 0 0 0 0 0 0 0 0 0 0 0 0 0 0 0 0 0 0 0 0 0 0 0 0 0 0 0 0 0 0 0 0 0 0 0 0 0 0 0

K00575 352 420 344 166 436 336 276 572 552 574 664 728 268 601 388 202 508 870 526 494 648 784 838 618 354 338 346 218 292 520 24 678 436 662 772 654 212 438 429 340 452 507 327 580 572 350 470 533

K00577 146 122 215 79 649 500 132 818 151 223 412 130 46 230 282 94 266 498 106 175 270 319 347 440 36 56 46 71 70 688 0 262 172 228 180 170 66 110 257 277 451 355 103 332 280 324 176 531

K00578 66 40 107 47 282 216 72 348 84 124 195 86 28 72 124 27 101 222 59 62 106 150 124 208 12 30 21 9 38 292 0 80 50 88 83 77 37 54 90 122 168 154 28 103 96 144 59 230

K00579 66 40 107 47 282 216 72 348 84 124 195 86 28 72 124 27 101 222 59 62 106 150 124 208 12 30 21 9 38 292 0 80 50 88 83 77 37 54 90 122 168 154 28 103 96 144 59 230

K00580 66 40 107 47 282 216 72 348 84 124 195 86 28 72 124 27 101 222 59 62 106 150 124 208 12 30 21 9 38 292 0 80 50 88 83 77 37 54 90 122 168 154 28 103 96 144 59 230

K00581 66 40 107 47 282 216 72 348 84 124 195 86 28 72 124 27 101 222 59 62 106 150 124 208 12 30 21 9 38 292 0 80 50 88 83 77 37 54 90 122 168 154 28 103 96 144 59 230

K00582 66 40 107 47 282 216 72 348 84 124 195 86 28 72 124 27 101 222 59 62 106 150 124 208 12 30 21 9 38 292 0 80 50 88 83 77 37 54 90 122 168 154 28 103 96 144 59 230

K00583 66 40 107 47 258 200 72 338 84 124 192 86 28 72 124 27 101 208 59 62 106 150 124 186 12 30 21 9 38 274 0 50 50 88 83 77 37 54 90 122 168 154 28 103 96 144 59 230

K00584 66 40 107 47 306 232 72 356 84 124 195 86 28 72 124 27 101 236 59 62 106 150 124 214 12 30 21 9 38 304 0 80 50 88 83 77 37 54 90 122 168 154 28 103 96 144 59 230

K00586 140 304 301 96 572 477 136 656 216 244 481 240 119 325 347 119 410 526 223 347 439 342 496 482 120 132 126 90 166 704 0 391 212 228 322 280 96 320 486 328 500 486 182 590 590 308 318 602

K00587 0 0 0 0 0 0 0 0 0 0 0 0 0 0 0 0 0 0 0 0 0 0 0 0 0 0 0 0 0 0 0 0 0 0 0 0 0 0 0 0 0 0 0 0 0 0 0 0

K00588 0 0 0 0 0 0 0 0 0 0 0 0 0 0 0 0 0 0 0 0 0 0 0 0 0 0 0 0 0 0 0 0 0 0 0 0 0 0 0 0 0 0 0 0 0 0 0 0

K00590 0 0 0 0 23 16 0 9 0 0 0 0 0 0 0 0 0 14 0 0 0 0 0 6 0 0 0 0 0 13 0 0 0 0 0 0 0 0 0 0 0 0 0 0 0 0 0 0

K00594 0 0 0 0 0 0 0 0 0 0 0 0 0 0 0 0 0 0 0 0 0 0 0 0 0 0 0 0 0 0 0 0 0 0 0 0 0 0 0 0 0 0 0 0 0 0 0 0

K00595 371 438 417 198 610 474 318 862 592 672 814 774 286 638 450 222 598 1023 573 490 714 918 922 712 354 364 359 218 314 711 24 652 464 680 813 682 240 463 494 446 604 604 327 658 643 490 514 706

K00596 0 0 0 0 0 0 0 0 0 0 0 0 0 0 0 0 0 0 0 0 0 0 0 0 0 0 0 0 0 0 0 0 0 0 0 0 0 0 0 0 0 0 0 0 0 0 0 0

K00598 0 0 0 0 0 0 0 0 0 0 0 0 0 0 0 0 0 0 0 0 0 0 0 0 0 0 0 0 0 0 0 0 0 0 0 0 0 0 0 0 0 0 0 0 0 0 0 0

K00599 338 530 448 190 480 370 292 645 616 638 702 832 303 631 468 202 644 950 549 568 742 809 852 616 374 338 356 218 322 633 24 726 489 661 886 688 212 469 554 462 627 656 388 790 754 388 576 588

K00600 520 924 804 312 1136 944 468 1364 873 938 1367 1122 478 1058 878 366 1153 1592 912 1028 1322 1284 1568 1272 558 574 566 328 574 1449 24 1203 716 933 1276 1072 366 994 1210 825 1152 1228 589 1507 1542 783 972 1378

K00601 0 0 0 0 0 0 0 0 0 0 0 0 0 0 0 0 0 0 0 0 0 0 0 0 0 0 0 0 0 0 0 0 0 0 0 0 0 0 0 0 0 0 0 0 0 0 0 0

K00602 421 820 662 264 770 646 395 870 762 813 1116 1036 450 864 691 292 976 1303 854 886 1118 1098 1306 1044 522 544 533 256 526 1023 24 1036 572 772 1136 928 329 910 1019 654 854 986 486 1254 1334 600 838 1020

K00603 0 0 0 0 0 0 0 0 0 0 0 0 0 0 0 0 0 0 0 0 0 0 0 0 0 0 0 0 0 0 0 0 0 0 0 0 0 0 0 0 0 0 0 0 0 0 0 0

K00604 338 420 344 166 406 320 267 564 552 574 661 728 268 601 372 202 508 856 526 476 648 784 838 582 354 338 346 218 292 502 24 648 436 638 772 654 212 438 429 340 452 498 327 580 572 350 470 533

K00605 380 620 503 216 564 468 331 708 657 694 886 882 358 732 532 247 742 1066 690 680 883 941 1072 790 438 441 440 238 409 745 24 812 504 705 954 792 270 674 724 497 653 742 406 916 953 474 654 776

K00606 338 420 344 166 406 320 267 564 552 574 661 728 268 601 372 202 508 856 526 476 648 784 838 582 354 338 346 218 292 502 24 648 436 638 772 654 212 438 429 340 452 498 327 580 572 350 470 533

K00607 0 0 0 0 0 0 0 0 0 0 0 0 0 0 0 0 0 0 0 0 0 0 0 0 0 0 0 0 0 0 0 0 0 0 0 0 0 0 0 0 0 0 0 0 0 0 0 0

K00608 0 0 0 0 0 0 0 0 0 0 0 0 0 0 0 0 0 0 0 0 0 0 0 0 0 0 0 0 0 0 0 0 0 0 0 0 0 0 0 0 0 0 0 0 0 0 0 0

K00609 478 724 645 262 978 798 404 1219 768 818 1142 968 386 926 719 320 919 1382 748 822 1088 1127 1335 1064 473 471 472 308 458 1206 24 1038 648 866 1094 934 308 759 916 668 952 984 510 1170 1161 658 788 1134

K00610 140 304 301 96 572 477 136 656 216 244 481 240 119 325 347 119 410 526 223 347 439 342 496 482 120 132 126 90 166 704 0 391 212 228 322 280 96 320 486 328 500 486 182 590 590 308 318 602

K00611 478 724 645 262 978 798 404 1219 768 818 1142 968 386 926 719 320 919 1382 748 822 1088 1127 1335 1064 473 471 472 308 458 1206 24 1038 648 866 1094 934 308 759 916 668 952 984 510 1170 1161 658 788 1134

K00612 0 0 0 0 0 0 0 0 0 0 0 0 0 0 0 0 0 0 0 0 0 0 0 0 0 0 0 0 0 0 0 0 0 0 0 0 0 0 0 0 0 0 0 0 0 0 0 0

K00613 0 0 0 0 0 0 0 0 0 0 0 0 0 0 0 0 0 0 0 0 0 0 0 0 0 0 0 0 0 0 0 0 0 0 0 0 0 0 0 0 0 0 0 0 0 0 0 0

K00614 0 0 0 0 0 0 0 0 0 0 0 0 0 0 0 0 0 0 0 0 0 0 0 0 0 0 0 0 0 0 0 0 0 0 0 0 0 0 0 0 0 0 0 0 0 0 0 0

K00615 454 884 697 264 876 745 395 1025 789 813 1172 1036 450 986 755 339 1052 1384 854 965 1216 1133 1445 1070 546 544 545 318 536 1170 24 1122 666 845 1193 994 329 940 1121 703 984 1074 561 1404 1446 640 913 1148

K00616 380 620 503 216 564 468 331 708 657 694 886 882 358 732 532 247 742 1066 690 680 883 941 1072 790 438 441 440 238 409 745 24 812 504 705 954 792 270 674 724 497 653 742 406 916 953 474 654 776

K00617 0 0 0 0 0 0 0 0 0 0 0 0 0 0 0 0 0 0 0 0 0 0 0 0 0 0 0 0 0 0 0 0 0 0 0 0 0 0 0 0 0 0 0 0 0 0 0 0

K00619 0 0 0 0 0 0 0 0 0 0 0 0 0 0 0 0 0 0 0 0 0 0 0 0 0 0 0 0 0 0 0 0 0 0 0 0 0 0 0 0 0 0 0 0 0 0 0 0

K00620 504 564 593 260 1102 866 412 1422 746 823 1111 900 324 866 683 302 786 1395 644 680 958 1121 1225 1064 400 398 399 298 380 1254 24 954 631 887 995 874 286 578 710 633 918 894 458 936 876 676 663 1122

K00621 0 0 0 0 0 0 0 0 0 0 0 0 0 0 0 0 0 0 0 0 0 0 0 0 0 0 0 0 0 0 0 0 0 0 0 0 0 0 0 0 0 0 0 0 0 0 0 0

K00622 0 0 0 0 0 0 0 0 0 0 0 0 0 0 0 0 0 0 0 0 0 0 0 0 0 0 0 0 0 0 0 0 0 0 0 0 0 0 0 0 0 0 0 0 0 0 0 0

K00624 0 0 0 0 0 0 0 0 0 0 0 0 0 0 0 0 0 0 0 0 0 0 0 0 0 0 0 0 0 0 0 0 0 0 0 0 0 0 0 0 0 0 0 0 0 0 0 0

K00625 0 0 0 0 0 0 0 0 0 0 0 0 0 0 0 0 0 0 0 0 0 0 0 0 0 0 0 0 0 0 0 0 0 0 0 0 0 0 0 0 0 0 0 0 0 0 0 0

K00626 890 1408 1183 478 1672 1380 734 2090 1451 1512 2089 1850 745 1780 1314 614 1737 2543 1438 1582 2070 2104 2546 1918 934 912 923 608 878 2120 48 1996 1246 1644 2105 1792 578 1464 1742 1214 1735 1814 991 2237 2226 1172 1517 2038

K00627 338 420 344 166 406 320 267 564 552 574 661 728 268 601 372 202 508 856 526 476 648 784 838 582 354 338 346 218 292 502 24 648 436 638 772 654 212 438 429 340 452 498 327 580 572 350 470 533

K00630 0 0 0 0 0 0 0 0 0 0 0 0 0 0 0 0 0 0 0 0 0 0 0 0 0 0 0 0 0 0 0 0 0 0 0 0 0 0 0 0 0 0 0 0 0 0 0 0

K00631 0 0 0 0 0 0 0 0 0 0 0 0 0 0 0 0 0 0 0 0 0 0 0 0 0 0 0 0 0 0 0 0 0 0 0 0 0 0 0 0 0 0 0 0 0 0 0 0

K00632 0 0 0 0 0 0 0 0 0 0 0 0 0 0 0 0 0 0 0 0 0 0 0 0 0 0 0 0 0 0 0 0 0 0 0 0 0 0 0 0 0 0 0 0 0 0 0 0

K00633 0 0 0 0 0 0 0 0 0 0 0 0 0 0 0 0 0 0 0 0 0 0 0 0 0 0 0 0 0 0 0 0 0 0 0 0 0 0 0 0 0 0 0 0 0 0 0 0

K00634 0 0 0 0 0 0 0 0 0 0 0 0 0 0 0 0 0 0 0 0 0 0 0 0 0 0 0 0 0 0 0 0 0 0 0 0 0 0 0 0 0 0 0 0 0 0 0 0

K00635 0 0 0 0 0 0 0 0 0 0 0 0 0 0 0 0 0 0 0 0 0 0 0 0 0 0 0 0 0 0 0 0 0 0 0 0 0 0 0 0 0 0 0 0 0 0 0 0

K00638 52 40 107 47 252 200 64 338 84 124 192 86 28 72 108 27 101 208 59 44 106 150 124 172 12 30 21 9 38 274 0 50 50 64 83 77 37 54 90 122 168 145 28 103 96 144 59 230

K00639 74 218 232 81 364 301 116 443 146 218 378 199 110 168 238 66 323 376 212 220 300 290 318 338 86 128 107 19 138 452 0 170 96 110 222 164 87 260 360 263 354 349 80 416 453 265 227 416

K00640 390 460 451 214 682 536 331 911 636 698 856 814 296 673 480 228 610 1078 584 520 754 935 962 776 365 368 367 228 330 794 24 728 486 702 856 732 249 493 518 462 620 644 355 682 668 493 530 764

K00641 119 80 214 94 512 399 136 677 168 249 387 171 56 144 231 54 202 416 118 106 211 301 247 374 23 60 42 18 76 552 0 128 101 152 166 154 74 109 179 244 336 298 56 206 192 287 118 461

K00643 0 0 0 0 0 0 0 0 0 0 0 0 0 0 0 0 0 0 0 0 0 0 0 0 0 0 0 0 0 0 0 0 0 0 0 0 0 0 0 0 0 0 0 0 0 0 0 0

K00645 338 420 344 166 406 320 267 564 552 574 661 728 268 601 372 202 508 856 526 476 648 784 838 582 354 338 346 218 292 502 24 648 436 638 772 654 212 438 429 340 452 498 327 580 572 350 470 533

K00646 0 0 0 0 0 0 0 0 0 0 0 0 0 0 0 0 0 0 0 0 0 0 0 0 0 0 0 0 0 0 0 0 0 0 0 0 0 0 0 0 0 0 0 0 0 0 0 0

K00647 0 0 0 0 0 0 0 0 0 0 0 0 0 0 0 0 0 0 0 0 0 0 0 0 0 0 0 0 0 0 0 0 0 0 0 0 0 0 0 0 0 0 0 0 0 0 0 0

K00648 338 420 344 166 406 320 267 564 552 574 661 728 268 601 372 202 508 856 526 476 648 784 838 582 354 338 346 218 292 502 24 648 436 638 772 654 212 438 429 340 452 498 327 580 572 350 470 533

K00650 0 0 0 0 0 0 0 0 0 0 0 0 0 0 0 0 0 0 0 0 0 0 0 0 0 0 0 0 0 0 0 0 0 0 0 0 0 0 0 0 0 0 0 0 0 0 0 0

K00651 0 0 0 0 0 0 0 0 0 0 0 0 0 0 0 0 0 0 0 0 0 0 0 0 0 0 0 0 0 0 0 0 0 0 0 0 0 0 0 0 0 0 0 0 0 0 0 0

K00652 338 420 344 166 406 320 267 564 552 574 661 728 268 601 372 202 508 856 526 476 648 784 838 582 354 338 346 218 292 502 24 648 436 638 772 654 212 438 429 340 452 498 327 580 572 350 470 533

K00653 0 0 0 0 0 0 0 0 0 0 0 0 0 0 0 0 0 0 0 0 0 0 0 0 0 0 0 0 0 0 0 0 0 0 0 0 0 0 0 0 0 0 0 0 0 0 0 0

K00654 0 0 0 0 0 0 0 0 0 0 0 0 0 0 0 0 0 0 0 0 0 0 0 0 0 0 0 0 0 0 0 0 0 0 0 0 0 0 0 0 0 0 0 0 0 0 0 0

K00655 338 420 344 166 406 320 267 564 552 574 661 728 268 601 372 202 508 856 526 476 648 784 838 582 354 338 346 218 292 502 24 648 436 638 772 654 212 438 429 340 452 498 327 580 572 350 470 533

K00656 74 218 232 81 370 301 124 443 146 218 378 199 110 168 254 66 323 376 212 220 300 290 318 352 86 128 107 19 138 452 0 170 96 110 222 164 87 260 360 263 354 358 80 416 453 265 227 416

K00657 0 0 0 0 0 0 0 0 0 0 0 0 0 0 0 0 0 0 0 0 0 0 0 0 0 0 0 0 0 0 0 0 0 0 0 0 0 0 0 0 0 0 0 0 0 0 0 0

K00658 0 0 0 0 0 0 0 0 0 0 0 0 0 0 0 0 0 0 0 0 0 0 0 0 0 0 0 0 0 0 0 0 0 0 0 0 0 0 0 0 0 0 0 0 0 0 0 0

K00660 0 0 0 0 0 0 0 0 0 0 0 0 0 0 0 0 0 0 0 0 0 0 0 0 0 0 0 0 0 0 0 0 0 0 0 0 0 0 0 0 0 0 0 0 0 0 0 0

K00661 33 18 73 32 205 154 52 298 40 98 154 45 18 37 78 20 89 167 48 14 66 133 84 130 1 26 13 0 21 209 0 5 28 42 40 28 28 24 66 106 152 105 0 78 72 140 43 173

K00662 0 0 0 0 0 0 0 0 0 0 0 0 0 0 0 0 0 0 0 0 0 0 0 0 0 0 0 0 0 0 0 0 0 0 0 0 0 0 0 0 0 0 0 0 0 0 0 0

K00663 0 0 0 0 0 0 0 0 0 0 0 0 0 0 0 0 0 0 0 0 0 0 0 0 0 0 0 0 0 0 0 0 0 0 0 0 0 0 0 0 0 0 0 0 0 0 0 0

K00666 404 460 451 214 688 536 340 911 636 698 856 814 296 673 496 228 610 1078 584 538 754 935 962 790 365 368 367 228 330 794 24 728 486 726 856 732 249 493 518 462 620 652 355 682 668 493 530 764

K00672 166 144 249 94 673 530 145 850 194 249 448 171 56 266 311 100 278 525 118 204 310 336 386 459 46 60 53 80 87 735 0 277 195 249 222 220 74 140 281 293 466 395 131 356 304 327 192 588

K00673 0 0 0 0 0 0 0 0 0 0 0 0 0 0 0 0 0 0 0 0 0 0 0 0 0 0 0 0 0 0 0 0 0 0 0 0 0 0 0 0 0 0 0 0 0 0 0 0

K00674 0 0 0 0 0 0 0 0 0 0 0 0 0 0 0 0 0 0 0 0 0 0 0 0 0 0 0 0 0 0 0 0 0 0 0 0 0 0 0 0 0 0 0 0 0 0 0 0

K00675 0 0 0 0 0 0 0 0 0 0 0 0 0 0 0 0 0 0 0 0 0 0 0 0 0 0 0 0 0 0 0 0 0 0 0 0 0 0 0 0 0 0 0 0 0 0 0 0

K00676 0 0 0 0 0 0 0 0 0 0 0 0 0 0 0 0 0 0 0 0 0 0 0 0 0 0 0 0 0 0 0 0 0 0 0 0 0 0 0 0 0 0 0 0 0 0 0 0

K00677 338 420 344 166 406 320 267 564 552 574 661 728 268 601 372 202 508 856 526 476 648 784 838 582 354 338 346 218 292 502 24 648 436 638 772 654 212 438 429 340 452 498 327 580 572 350 470 533

K00680 42 200 159 49 182 163 64 154 106 120 225 154 91 132 160 46 234 224 164 205 234 156 234 214 84 102 94 19 116 256 0 168 68 67 182 137 58 236 295 157 201 244 80 337 382 125 184 244

K00681 338 420 344 166 406 320 267 564 552 574 661 728 268 601 372 202 508 856 526 476 648 784 838 582 354 338 346 218 292 502 24 648 436 638 772 654 212 438 429 340 452 498 327 580 572 350 470 533

K00683 0 0 0 0 0 0 0 0 0 0 0 0 0 0 0 0 0 0 0 0 0 0 0 0 0 0 0 0 0 0 0 0 0 0 0 0 0 0 0 0 0 0 0 0 0 0 0 0

K00684 0 0 0 0 0 0 0 0 0 0 0 0 0 0 0 0 0 0 0 0 0 0 0 0 0 0 0 0 0 0 0 0 0 0 0 0 0 0 0 0 0 0 0 0 0 0 0 0

K00685 0 0 0 0 0 0 0 0 0 0 0 0 0 0 0 0 0 0 0 0 0 0 0 0 0 0 0 0 0 0 0 0 0 0 0 0 0 0 0 0 0 0 0 0 0 0 0 0

K00686 0 0 0 0 0 0 0 0 0 0 0 0 0 0 0 0 0 0 0 0 0 0 0 0 0 0 0 0 0 0 0 0 0 0 0 0 0 0 0 0 0 0 0 0 0 0 0 0

K00687 0 0 0 0 0 0 0 0 0 0 0 0 0 0 0 0 0 0 0 0 0 0 0 0 0 0 0 0 0 0 0 0 0 0 0 0 0 0 0 0 0 0 0 0 0 0 0 0

K00688 338 420 344 166 430 336 267 572 552 574 664 728 268 601 372 202 508 870 526 476 648 784 838 604 354 338 346 218 292 520 24 678 436 638 772 654 212 438 429 340 452 498 327 580 572 350 470 533

K00689 0 0 0 0 0 0 0 0 0 0 0 0 0 0 0 0 0 0 0 0 0 0 0 0 0 0 0 0 0 0 0 0 0 0 0 0 0 0 0 0 0 0 0 0 0 0 0 0

K00690 0 0 0 0 0 0 0 0 0 0 0 0 0 0 0 0 0 0 0 0 0 0 0 0 0 0 0 0 0 0 0 0 0 0 0 0 0 0 0 0 0 0 0 0 0 0 0 0

K00691 0 0 0 0 0 0 0 0 0 0 0 0 0 0 0 0 0 0 0 0 0 0 0 0 0 0 0 0 0 0 0 0 0 0 0 0 0 0 0 0 0 0 0 0 0 0 0 0

K00692 0 0 0 0 0 0 0 0 0 0 0 0 0 0 0 0 0 0 0 0 0 0 0 0 0 0 0 0 0 0 0 0 0 0 0 0 0 0 0 0 0 0 0 0 0 0 0 0

K00693 0 0 0 0 0 0 0 0 0 0 0 0 0 0 0 0 0 0 0 0 0 0 0 0 0 0 0 0 0 0 0 0 0 0 0 0 0 0 0 0 0 0 0 0 0 0 0 0

K00694 42 200 159 49 182 163 64 154 106 120 225 154 91 132 160 46 234 224 164 205 234 156 234 214 84 102 94 19 116 256 0 164 68 67 182 137 58 236 295 157 201 244 80 337 382 125 184 244

K00695 0 0 0 0 0 0 0 0 0 0 0 0 0 0 0 0 0 0 0 0 0 0 0 0 0 0 0 0 0 0 0 0 0 0 0 0 0 0 0 0 0 0 0 0 0 0 0 0

K00696 0 0 0 0 0 0 0 0 0 0 0 0 0 0 0 0 0 0 0 0 0 0 0 0 0 0 0 0 0 0 0 0 0 0 0 0 0 0 0 0 0 0 0 0 0 0 0 0

K00697 14 0 0 0 30 16 8 9 0 0 0 0 0 0 16 0 0 14 0 18 0 0 0 20 0 0 0 0 0 13 0 0 0 24 0 0 0 0 0 0 0 8 0 0 0 0 0 0

K00698 0 0 0 0 0 0 0 0 0 0 0 0 0 0 0 0 0 0 0 0 0 0 0 0 0 0 0 0 0 0 0 0 0 0 0 0 0 0 0 0 0 0 0 0 0 0 0 0

K00700 0 0 0 0 23 16 0 9 0 0 0 0 0 0 0 0 0 14 0 0 0 0 0 6 0 0 0 0 0 13 0 0 0 0 0 0 0 0 0 0 0 0 0 0 0 0 0 0

K00701 0 0 0 0 0 0 0 0 0 0 0 0 0 0 0 0 0 0 0 0 0 0 0 0 0 0 0 0 0 0 0 0 0 0 0 0 0 0 0 0 0 0 0 0 0 0 0 0

K00702 0 0 0 0 0 0 0 0 0 0 0 0 0 0 0 0 0 0 0 0 0 0 0 0 0 0 0 0 0 0 0 0 0 0 0 0 0 0 0 0 0 0 0 0 0 0 0 0

K00703 338 420 344 166 406 320 267 564 552 574 661 728 268 601 372 202 508 856 526 476 648 784 838 582 354 338 346 218 292 502 24 648 436 638 772 654 212 438 429 340 452 498 327 580 572 350 470 533

K00705 0 0 0 0 23 16 0 9 0 0 3 0 0 0 0 0 0 14 0 0 0 0 0 6 0 0 0 0 0 13 0 0 0 0 0 0 0 0 0 0 0 0 0 0 0 0 0 0

K00712 0 0 0 0 0 0 0 0 0 0 0 0 0 0 0 0 0 0 0 0 0 0 0 0 0 0 0 0 0 0 0 0 0 0 0 0 0 0 0 0 0 0 0 0 0 0 0 0

K00713 0 0 0 0 0 0 0 0 0 0 0 0 0 0 0 0 0 0 0 0 0 0 0 0 0 0 0 0 0 0 0 0 0 0 0 0 0 0 0 0 0 0 0 0 0 0 0 0

K00720 0 0 0 0 0 0 0 0 0 0 0 0 0 0 0 0 0 0 0 0 0 0 0 0 0 0 0 0 0 0 0 0 0 0 0 0 0 0 0 0 0 0 0 0 0 0 0 0

K00721 426 573 434 192 692 581 306 817 618 630 914 778 323 824 499 294 683 1109 666 686 888 952 1198 872 440 441 441 300 390 832 24 942 545 780 897 824 270 674 700 424 608 674 420 857 882 476 623 849

K00728 0 0 0 0 0 0 0 0 0 0 0 0 0 0 0 0 0 0 0 0 0 0 0 0 0 0 0 0 0 0 0 0 0 0 0 0 0 0 0 0 0 0 0 0 0 0 0 0

K00729 0 0 0 0 0 0 0 0 0 0 0 0 0 0 0 0 0 0 0 0 0 0 0 0 0 0 0 0 0 0 0 0 0 0 0 0 0 0 0 0 0 0 0 0 0 0 0 0

K00733 0 0 0 0 0 0 0 0 0 0 0 0 0 0 0 0 0 0 0 0 0 0 0 0 0 0 0 0 0 0 0 0 0 0 0 0 0 0 0 0 0 0 0 0 0 0 0 0

K00737 0 0 0 0 0 0 0 0 0 0 0 0 0 0 0 0 0 0 0 0 0 0 0 0 0 0 0 0 0 0 0 0 0 0 0 0 0 0 0 0 0 0 0 0 0 0 0 0

K00743 0 0 0 0 0 0 0 0 0 0 0 0 0 0 0 0 0 0 0 0 0 0 0 0 0 0 0 0 0 0 0 0 0 0 0 0 0 0 0 0 0 0 0 0 0 0 0 0

K00745 0 0 0 0 0 0 0 0 0 0 0 0 0 0 0 0 0 0 0 0 0 0 0 0 0 0 0 0 0 0 0 0 0 0 0 0 0 0 0 0 0 0 0 0 0 0 0 0

K00748 338 420 344 166 406 320 267 564 552 574 661 728 268 601 372 202 508 856 526 476 648 784 838 582 354 338 346 218 292 502 24 648 436 638 772 654 212 438 429 340 452 498 327 580 572 350 470 533

K00752 0 0 0 0 0 0 0 0 0 0 0 0 0 0 0 0 0 0 0 0 0 0 0 0 0 0 0 0 0 0 0 0 0 0 0 0 0 0 0 0 0 0 0 0 0 0 0 0

K00754 338 420 344 166 406 320 267 564 552 574 661 728 268 601 372 202 508 856 526 476 648 784 838 582 354 338 346 218 292 502 24 648 436 638 772 654 212 438 429 340 452 498 327 580 572 350 470 533

K00756 0 0 0 0 0 0 0 0 0 0 0 0 0 0 0 0 0 0 0 0 0 0 0 0 0 0 0 0 0 0 0 0 0 0 0 0 0 0 0 0 0 0 0 0 0 0 0 0

K00757 42 200 159 49 158 147 64 144 106 120 225 154 91 132 160 46 234 210 164 205 234 156 234 208 84 102 94 19 116 243 0 164 68 67 182 137 58 236 295 157 201 244 80 337 382 125 184 244

K00758 42 200 159 49 182 163 64 154 106 120 228 154 91 132 160 46 234 224 164 205 234 156 234 232 84 102 94 19 116 260 0 194 68 67 182 137 58 236 295 157 201 244 80 337 382 125 184 244

K00759 478 724 645 262 978 798 404 1219 768 818 1142 968 386 926 719 320 919 1382 748 822 1088 1127 1335 1064 473 471 472 308 458 1206 24 1038 648 866 1094 934 308 759 916 668 952 984 510 1170 1161 658 788 1134

K00760 0 0 0 0 0 0 0 0 0 0 0 0 0 0 0 0 0 0 0 0 0 0 0 0 0 0 0 0 0 0 0 0 0 0 0 0 0 0 0 0 0 0 0 0 0 0 0 0

K00761 66 40 107 47 258 200 72 338 84 124 192 86 28 72 124 27 101 208 59 62 106 150 124 186 12 30 21 9 38 274 0 50 50 88 83 77 37 54 90 122 168 154 28 103 96 144 59 230

K00762 586 964 911 358 1418 1160 540 1711 957 1062 1561 1208 506 1130 1002 393 1254 1814 972 1090 1428 1434 1692 1480 569 604 586 336 612 1740 24 1284 767 1021 1359 1148 403 1049 1300 947 1320 1382 617 1610 1638 926 1031 1608

K00763 412 684 538 216 719 598 331 880 684 694 950 882 358 854 596 294 818 1174 690 760 982 976 1212 878 462 441 451 300 420 932 24 988 598 778 1011 858 270 704 826 546 784 830 482 1067 1065 514 729 904

K00764 478 724 645 262 978 798 404 1219 768 818 1142 968 386 926 719 320 919 1382 748 822 1088 1127 1335 1064 473 471 472 308 458 1206 24 1038 648 866 1094 934 308 759 916 668 952 984 510 1170 1161 658 788 1134

K00765 437 524 486 214 843 666 340 1084 662 698 919 814 296 794 560 275 685 1187 584 618 853 970 1102 861 388 368 378 290 342 976 24 874 580 799 912 798 249 524 620 511 750 740 430 833 780 533 604 891

K00766 437 524 486 214 819 650 340 1074 662 698 917 814 296 794 560 275 685 1173 584 618 853 970 1102 855 388 368 378 290 342 963 24 874 580 799 912 798 249 524 620 511 750 740 430 833 780 533 604 891

K00767 478 724 645 262 978 798 404 1219 768 818 1142 968 386 926 719 320 919 1382 748 822 1088 1127 1335 1064 473 471 472 308 458 1206 24 1038 648 866 1094 934 308 759 916 668 952 984 510 1170 1161 658 788 1134

K00768 0 0 0 0 0 0 0 0 0 0 0 0 0 0 0 0 0 0 0 0 0 0 0 0 0 0 0 0 0 0 0 0 0 0 0 0 0 0 0 0 0 0 0 0 0 0 0 0

K00769 0 0 0 0 0 0 0 0 0 0 0 0 0 0 0 0 0 0 0 0 0 0 0 0 0 0 0 0 0 0 0 0 0 0 0 0 0 0 0 0 0 0 0 0 0 0 0 0

K00772 520 924 804 312 1136 944 468 1364 873 938 1367 1122 478 1058 878 366 1153 1592 912 1028 1322 1284 1568 1272 558 574 566 328 574 1449 24 1203 716 933 1276 1072 366 994 1210 825 1152 1228 589 1507 1542 783 972 1378

K00773 412 684 538 216 719 598 331 880 684 694 950 882 358 854 596 294 818 1174 690 760 982 976 1212 878 462 441 451 300 420 932 24 988 598 778 1011 858 270 704 826 546 784 830 482 1067 1065 514 729 904

K00776 0 0 0 0 0 0 0 0 0 0 0 0 0 0 0 0 0 0 0 0 0 0 0 0 0 0 0 0 0 0 0 0 0 0 0 0 0 0 0 0 0 0 0 0 0 0 0 0

K00777 0 0 0 0 0 0 0 0 0 0 0 0 0 0 0 0 0 0 0 0 0 0 0 0 0 0 0 0 0 0 0 0 0 0 0 0 0 0 0 0 0 0 0 0 0 0 0 0

K00782 108 240 266 96 417 346 136 483 190 244 417 240 119 204 283 72 335 418 223 268 340 307 357 394 96 132 114 28 154 517 0 215 118 155 265 214 96 290 384 279 369 398 108 440 478 268 243 474

K00783 42 200 159 49 160 147 64 144 106 120 228 154 91 132 160 46 234 210 164 205 234 156 234 226 84 102 94 19 116 248 0 194 68 67 182 137 58 236 295 157 201 244 80 337 382 125 184 244

K00784 478 724 645 262 978 798 404 1219 768 818 1142 968 386 926 719 320 919 1382 748 822 1088 1127 1335 1064 473 471 472 308 458 1206 24 1038 648 866 1094 934 308 759 916 668 952 984 510 1170 1161 658 788 1134

K00785 0 0 0 0 0 0 0 0 0 0 0 0 0 0 0 0 0 0 0 0 0 0 0 0 0 0 0 0 0 0 0 0 0 0 0 0 0 0 0 0 0 0 0 0 0 0 0 0

K00786 42 311 264 72 255 212 88 235 170 184 263 258 126 162 256 46 369 317 188 297 328 181 247 249 106 102 104 19 146 387 0 244 121 90 296 170 58 266 420 279 376 401 141 547 564 164 290 298

K00788 432 660 610 262 840 683 395 1056 741 818 1081 968 386 804 639 274 844 1288 748 724 988 1092 1196 985 450 471 460 246 447 1036 24 892 554 768 1038 868 308 728 814 619 821 888 434 1020 1049 618 714 1007

K00789 478 724 645 262 978 798 404 1219 768 818 1142 968 386 926 719 320 919 1382 748 822 1088 1127 1335 1064 473 471 472 308 458 1206 24 1038 648 866 1094 934 308 759 916 668 952 984 510 1170 1161 658 788 1134

K00790 338 420 344 166 406 320 267 564 552 574 661 728 268 601 372 202 508 856 526 476 648 784 838 582 354 338 346 218 292 502 24 648 436 638 772 654 212 438 429 340 452 498 327 580 572 350 470 533

K00791 338 420 344 166 406 320 267 564 552 574 661 728 268 601 372 202 508 856 526 476 648 784 838 582 354 338 346 218 292 502 24 648 436 638 772 654 212 438 429 340 452 498 327 580 572 350 470 533

K00793 478 724 645 262 978 798 404 1219 768 818 1142 968 386 926 719 320 919 1382 748 822 1088 1127 1335 1064 473 471 472 308 458 1206 24 1038 648 866 1094 934 308 759 916 668 952 984 510 1170 1161 658 788 1134

K00794 478 724 645 262 1002 814 404 1228 768 818 1144 968 386 926 719 320 919 1396 748 822 1088 1127 1335 1070 473 471 472 308 458 1219 24 1038 648 866 1094 934 308 759 916 668 952 984 510 1170 1161 658 788 1134

K00795 0 0 0 0 0 0 0 0 0 0 0 0 0 0 0 0 0 0 0 0 0 0 0 0 0 0 0 0 0 0 0 0 0 0 0 0 0 0 0 0 0 0 0 0 0 0 0 0

K00796 380 620 503 216 588 484 331 717 657 694 889 882 358 732 532 247 742 1080 690 680 883 941 1072 813 438 441 440 238 409 762 24 842 504 705 954 792 270 674 724 497 653 742 406 916 953 474 654 776

K00797 412 684 538 216 695 582 331 872 684 694 950 882 358 854 596 294 818 1160 690 760 982 976 1212 855 462 441 451 300 420 914 24 958 598 778 1011 858 270 704 826 546 784 830 482 1067 1065 514 729 904

K00798 380 620 503 216 564 468 331 708 657 694 886 882 358 732 532 247 742 1066 690 680 883 941 1072 790 438 441 440 238 409 745 24 812 504 705 954 792 270 674 724 497 653 742 406 916 953 474 654 776

K00799 338 420 344 166 406 320 267 564 552 574 661 728 268 601 372 202 508 856 526 476 648 784 838 582 354 338 346 218 292 502 24 648 436 638 772 654 212 438 429 340 452 498 327 580 572 350 470 533

K00800 437 524 486 214 819 650 340 1074 662 698 917 814 296 794 560 275 685 1173 584 618 853 970 1102 855 388 368 378 290 342 963 24 874 580 799 912 798 249 524 620 511 750 740 430 833 780 533 604 891

K00801 0 0 0 0 0 0 0 0 0 0 0 0 0 0 0 0 0 0 0 0 0 0 0 0 0 0 0 0 0 0 0 0 0 0 0 0 0 0 0 0 0 0 0 0 0 0 0 0

K00802 0 0 0 0 0 0 0 0 0 0 0 0 0 0 0 0 0 0 0 0 0 0 0 0 0 0 0 0 0 0 0 0 0 0 0 0 0 0 0 0 0 0 0 0 0 0 0 0

K00803 0 0 0 0 0 0 0 0 0 0 0 0 0 0 0 0 0 0 0 0 0 0 0 0 0 0 0 0 0 0 0 0 0 0 0 0 0 0 0 0 0 0 0 0 0 0 0 0

K00804 0 0 0 0 0 0 0 0 0 0 0 0 0 0 0 0 0 0 0 0 0 0 0 0 0 0 0 0 0 0 0 0 0 0 0 0 0 0 0 0 0 0 0 0 0 0 0 0

K00805 0 0 0 0 0 0 0 0 0 0 0 0 0 0 0 0 0 0 0 0 0 0 0 0 0 0 0 0 0 0 0 0 0 0 0 0 0 0 0 0 0 0 0 0 0 0 0 0

K00806 478 724 645 262 1002 814 404 1228 768 818 1144 968 386 926 719 320 919 1396 748 822 1088 1127 1335 1086 473 471 472 308 458 1224 24 1068 648 866 1094 934 308 759 916 668 952 984 510 1170 1161 658 788 1134

K00808 0 0 0 0 1 0 0 0 0 0 0 0 0 0 0 0 0 0 0 0 0 0 0 0 0 0 0 0 0 0 0 0 0 0 0 0 0 0 0 0 0 0 0 0 0 0 0 0

K00809 478 724 645 262 1000 814 404 1228 768 818 1144 968 386 926 719 320 919 1396 748 822 1088 1127 1335 1086 473 471 472 308 458 1224 24 1068 648 866 1094 934 308 759 916 668 952 984 510 1170 1161 658 788 1134

K00810 0 0 0 0 0 0 0 0 0 0 0 0 0 0 0 0 0 0 0 0 0 0 0 0 0 0 0 0 0 0 0 0 0 0 0 0 0 0 0 0 0 0 0 0 0 0 0 0

K00811 0 0 0 0 0 0 0 0 0 0 0 0 0 0 0 0 0 0 0 0 0 0 0 0 0 0 0 0 0 0 0 0 0 0 0 0 0 0 0 0 0 0 0 0 0 0 0 0

K00812 552 988 839 312 1291 1075 468 1536 900 938 1430 1122 478 1179 942 412 1228 1701 912 1107 1421 1319 1708 1360 581 574 577 390 586 1636 24 1382 810 1006 1332 1138 366 1025 1312 874 1283 1316 664 1658 1654 823 1046 1506

K00813 0 0 0 0 0 0 0 0 0 0 0 0 0 0 0 0 0 0 0 0 0 0 0 0 0 0 0 0 0 0 0 0 0 0 0 0 0 0 0 0 0 0 0 0 0 0 0 0

K00814 0 0 0 0 0 0 0 0 0 0 0 0 0 0 0 0 0 0 0 0 0 0 0 0 0 0 0 0 0 0 0 0 0 0 0 0 0 0 0 0 0 0 0 0 0 0 0 0

K00817 437 524 486 214 819 650 340 1074 662 698 917 814 296 794 560 275 685 1173 584 618 853 970 1102 855 388 368 378 290 342 963 24 874 580 799 912 798 249 524 620 511 750 740 430 833 780 533 604 891

K00818 0 0 0 0 0 0 0 0 0 0 0 0 0 0 0 0 0 0 0 0 0 0 0 0 0 0 0 0 0 0 0 0 0 0 0 0 0 0 0 0 0 0 0 0 0 0 0 0

K00819 0 0 0 0 0 0 0 0 0 0 0 0 0 0 0 0 0 0 0 0 0 0 0 0 0 0 0 0 0 0 0 0 0 0 0 0 0 0 0 0 0 0 0 0 0 0 0 0

K00820 578 782 825 342 1441 1151 528 1856 892 1041 1487 1098 433 1035 921 368 1109 1758 855 900 1258 1410 1542 1379 486 526 506 318 517 1689 24 1094 727 996 1218 1039 373 838 1070 896 1272 1242 538 1352 1328 942 890 1538

K00821 504 564 593 260 1102 866 412 1422 746 823 1111 900 324 866 683 302 786 1395 644 680 958 1121 1225 1064 400 398 399 298 380 1254 24 954 631 887 995 874 286 578 710 633 918 894 458 936 876 676 663 1122

K00822 0 0 0 0 0 0 0 0 0 0 0 0 0 0 0 0 0 0 0 0 0 0 0 0 0 0 0 0 0 0 0 0 0 0 0 0 0 0 0 0 0 0 0 0 0 0 0 0

K00823 32 64 35 0 131 114 0 164 26 0 61 0 0 122 64 46 76 95 0 80 99 36 140 65 24 0 12 62 11 170 0 150 94 73 56 66 0 30 102 49 130 88 75 150 112 40 74 128

K00824 0 0 0 0 0 0 0 0 0 0 0 0 0 0 0 0 0 0 0 0 0 0 0 0 0 0 0 0 0 0 0 0 0 0 0 0 0 0 0 0 0 0 0 0 0 0 0 0

K00826 437 524 486 214 819 650 340 1074 662 698 917 814 296 794 560 275 685 1173 584 618 853 970 1102 855 388 368 378 290 342 963 24 874 580 799 912 798 249 524 620 511 750 740 430 833 780 533 604 891

K00827 0 0 0 0 0 0 0 0 0 0 0 0 0 0 0 0 0 0 0 0 0 0 0 0 0 0 0 0 0 0 0 0 0 0 0 0 0 0 0 0 0 0 0 0 0 0 0 0

K00828 0 0 0 0 0 0 0 0 0 0 0 0 0 0 0 0 0 0 0 0 0 0 0 0 0 0 0 0 0 0 0 0 0 0 0 0 0 0 0 0 0 0 0 0 0 0 0 0

K00829 0 0 0 0 0 0 0 0 0 0 0 0 0 0 0 0 0 0 0 0 0 0 0 0 0 0 0 0 0 0 0 0 0 0 0 0 0 0 0 0 0 0 0 0 0 0 0 0

K00830 338 420 344 166 406 320 267 564 552 574 664 728 268 601 372 202 508 856 526 476 648 784 838 582 354 338 346 218 292 502 24 648 436 638 772 654 212 438 429 340 452 498 327 580 572 350 470 533

K00831 0 0 0 0 0 0 0 0 0 0 0 0 0 0 0 0 0 0 0 0 0 0 0 0 0 0 0 0 0 0 0 0 0 0 0 0 0 0 0 0 0 0 0 0 0 0 0 0

K00832 0 0 0 0 0 0 0 0 0 0 0 0 0 0 0 0 0 0 0 0 0 0 0 0 0 0 0 0 0 0 0 0 0 0 0 0 0 0 0 0 0 0 0 0 0 0 0 0

K00833 33 18 73 32 205 154 52 298 40 98 154 45 18 37 78 20 89 167 48 14 66 133 84 130 1 26 13 0 21 209 0 5 28 42 40 28 28 24 66 106 152 105 0 78 72 140 43 173

K00835 0 0 0 0 0 0 0 0 0 0 0 0 0 0 0 0 0 0 0 0 0 0 0 0 0 0 0 0 0 0 0 0 0 0 0 0 0 0 0 0 0 0 0 0 0 0 0 0

K00836 0 0 0 0 0 0 0 0 0 0 0 0 0 0 0 0 0 0 0 0 0 0 0 0 0 0 0 0 0 0 0 0 0 0 0 0 0 0 0 0 0 0 0 0 0 0 0 0

K00837 0 0 0 0 0 0 0 0 0 0 0 0 0 0 0 0 0 0 0 0 0 0 0 0 0 0 0 0 0 0 0 0 0 0 0 0 0 0 0 0 0 0 0 0 0 0 0 0

K00839 108 240 266 96 441 362 136 492 190 244 420 240 119 204 283 72 335 432 223 268 340 307 357 417 96 132 114 28 154 534 0 245 118 155 265 214 96 290 384 279 369 398 108 440 478 268 243 474

K00840 0 0 0 0 0 0 0 0 0 0 0 0 0 0 0 0 0 0 0 0 0 0 0 0 0 0 0 0 0 0 0 0 0 0 0 0 0 0 0 0 0 0 0 0 0 0 0 0

K00841 0 0 0 0 0 0 0 0 0 0 0 0 0 0 0 0 0 0 0 0 0 0 0 0 0 0 0 0 0 0 0 0 0 0 0 0 0 0 0 0 0 0 0 0 0 0 0 0

K00842 0 0 0 0 0 0 0 0 0 0 0 0 0 0 0 0 0 0 0 0 0 0 0 0 0 0 0 0 0 0 0 0 0 0 0 0 0 0 0 0 0 0 0 0 0 0 0 0

K00843 0 0 0 0 0 0 0 0 0 0 0 0 0 0 0 0 0 0 0 0 0 0 0 0 0 0 0 0 0 0 0 0 0 0 0 0 0 0 0 0 0 0 0 0 0 0 0 0

K00844 0 0 0 0 0 0 0 0 0 0 0 0 0 0 0 0 0 0 0 0 0 0 0 0 0 0 0 0 0 0 0 0 0 0 0 0 0 0 0 0 0 0 0 0 0 0 0 0

K00845 380 620 503 216 587 484 331 717 657 694 889 882 358 732 532 247 742 1080 690 680 883 941 1072 813 438 441 440 238 409 762 24 842 504 705 954 792 270 674 724 497 653 742 406 916 953 474 654 776

K00846 0 0 0 0 0 0 0 0 0 0 0 0 0 0 0 0 0 0 0 0 0 0 0 0 0 0 0 0 0 0 0 0 0 0 0 0 0 0 0 0 0 0 0 0 0 0 0 0

K00847 338 420 344 166 406 320 267 564 552 574 661 728 268 601 372 202 508 856 526 476 648 784 838 582 354 338 346 218 292 502 24 648 436 638 772 654 212 438 429 340 452 498 327 580 572 350 470 533

K00848 0 0 0 0 0 0 0 0 0 0 0 0 0 0 0 0 0 0 0 0 0 0 0 0 0 0 0 0 0 0 0 0 0 0 0 0 0 0 0 0 0 0 0 0 0 0 0 0

K00849 0 0 0 0 0 0 0 0 0 0 0 0 0 0 0 0 0 0 0 0 0 0 0 0 0 0 0 0 0 0 0 0 0 0 0 0 0 0 0 0 0 0 0 0 0 0 0 0

K00850 338 420 344 166 430 336 267 572 552 574 664 728 268 601 372 202 508 870 526 476 648 784 838 604 354 338 346 218 292 520 24 674 436 638 772 654 212 438 429 340 452 498 327 580 572 350 470 533

K00851 0 0 0 0 0 0 0 0 0 0 0 0 0 0 0 0 0 0 0 0 0 0 0 0 0 0 0 0 0 0 0 0 0 0 0 0 0 0 0 0 0 0 0 0 0 0 0 0

K00852 559 764 752 310 1242 997 484 1558 852 942 1334 1054 414 998 858 348 1020 1590 808 904 1193 1278 1458 1262 484 501 493 318 496 1480 24 1089 699 978 1177 1012 344 814 1005 790 1120 1146 538 1273 1257 802 847 1365

K00853 0 0 0 0 0 0 0 0 0 0 0 0 0 0 0 0 0 0 0 0 0 0 0 0 0 0 0 0 0 0 0 0 0 0 0 0 0 0 0 0 0 0 0 0 0 0 0 0

K00854 0 0 0 0 0 0 0 0 0 0 0 0 0 0 0 0 0 0 0 0 0 0 0 0 0 0 0 0 0 0 0 0 0 0 0 0 0 0 0 0 0 0 0 0 0 0 0 0

K00855 42 200 159 49 182 163 64 154 106 120 228 154 91 132 160 46 234 224 164 205 234 156 234 232 84 102 94 19 116 260 0 194 68 67 182 137 58 236 295 157 201 244 80 337 382 125 184 244

K00856 0 0 0 0 0 0 0 0 0 0 0 0 0 0 0 0 0 0 0 0 0 0 0 0 0 0 0 0 0 0 0 0 0 0 0 0 0 0 0 0 0 0 0 0 0 0 0 0

K00857 0 0 0 0 0 0 0 0 0 0 0 0 0 0 0 0 0 0 0 0 0 0 0 0 0 0 0 0 0 0 0 0 0 0 0 0 0 0 0 0 0 0 0 0 0 0 0 0

K00858 512 742 718 294 1182 952 455 1518 808 916 1295 1013 405 963 798 341 1008 1550 796 837 1153 1260 1419 1194 474 496 485 308 479 1415 24 1044 676 908 1134 962 336 784 981 774 1104 1089 510 1248 1232 798 831 1308

K00859 338 420 344 166 406 320 267 564 552 574 661 728 268 601 372 202 508 856 526 476 648 784 838 582 354 338 346 218 292 502 24 648 436 638 772 654 212 438 429 340 452 498 327 580 572 350 470 533

K00860 0 0 0 0 0 0 0 0 0 0 0 0 0 0 0 0 0 0 0 0 0 0 0 0 0 0 0 0 0 0 0 0 0 0 0 0 0 0 0 0 0 0 0 0 0 0 0 0

K00861 0 0 0 0 0 0 0 0 0 0 0 0 0 0 0 0 0 0 0 0 0 0 0 0 0 0 0 0 0 0 0 0 0 0 0 0 0 0 0 0 0 0 0 0 0 0 0 0

K00862 0 0 0 0 0 0 0 0 0 0 0 0 0 0 0 0 0 0 0 0 0 0 0 0 0 0 0 0 0 0 0 0 0 0 0 0 0 0 0 0 0 0 0 0 0 0 0 0

K00863 0 0 0 0 0 0 0 0 0 0 0 0 0 0 0 0 0 0 0 0 0 0 0 0 0 0 0 0 0 0 0 0 0 0 0 0 0 0 0 0 0 0 0 0 0 0 0 0

K00864 42 200 159 49 158 147 64 144 106 120 225 154 91 132 160 46 234 210 164 205 234 156 234 208 84 102 94 19 116 243 0 164 68 67 182 137 58 236 295 157 201 244 80 337 382 125 184 244

K00865 0 0 0 0 0 0 0 0 0 0 0 0 0 0 0 0 0 0 0 0 0 0 0 0 0 0 0 0 0 0 0 0 0 0 0 0 0 0 0 0 0 0 0 0 0 0 0 0

K00866 0 0 0 0 0 0 0 0 0 0 0 0 0 0 0 0 0 0 0 0 0 0 0 0 0 0 0 0 0 0 0 0 0 0 0 0 0 0 0 0 0 0 0 0 0 0 0 0

K00867 0 0 0 0 0 0 0 0 0 0 0 0 0 0 0 0 0 0 0 0 0 0 0 0 0 0 0 0 0 0 0 0 0 0 0 0 0 0 0 0 0 0 0 0 0 0 0 0

K00868 33 18 73 32 205 154 52 298 40 98 154 45 18 37 78 20 89 167 48 14 66 133 84 130 1 26 13 0 21 209 0 5 28 42 40 28 28 24 66 106 152 105 0 78 72 140 43 173

K00869 140 304 301 96 572 477 136 656 216 244 481 240 119 325 347 119 410 526 223 347 439 342 496 482 120 132 126 90 166 704 0 391 212 228 322 280 96 320 486 328 500 486 182 590 590 308 318 602

K00870 0 0 0 0 0 0 0 0 0 0 0 0 0 0 0 0 0 0 0 0 0 0 0 0 0 0 0 0 0 0 0 0 0 0 0 0 0 0 0 0 0 0 0 0 0 0 0 0

K00872 0 0 0 0 0 0 0 0 0 0 0 0 0 0 0 0 0 0 0 0 0 0 0 0 0 0 0 0 0 0 0 0 0 0 0 0 0 0 0 0 0 0 0 0 0 0 0 0

K00873 445 702 611 248 923 752 382 1179 724 792 1103 928 377 891 674 314 907 1342 737 774 1048 1110 1296 991 462 466 464 300 441 1136 24 963 626 820 1052 885 299 729 892 652 936 936 482 1146 1136 654 772 1077

K00874 0 0 0 0 0 0 0 0 0 0 0 0 0 0 0 0 0 0 0 0 0 0 0 0 0 0 0 0 0 0 0 0 0 0 0 0 0 0 0 0 0 0 0 0 0 0 0 0

K00875 0 0 0 0 0 0 0 0 0 0 0 0 0 0 0 0 0 0 0 0 0 0 0 0 0 0 0 0 0 0 0 0 0 0 0 0 0 0 0 0 0 0 0 0 0 0 0 0

K00876 0 0 0 0 0 0 0 0 0 0 0 0 0 0 0 0 0 0 0 0 0 0 0 0 0 0 0 0 0 0 0 0 0 0 0 0 0 0 0 0 0 0 0 0 0 0 0 0

K00877 0 0 0 0 0 0 0 0 0 0 0 0 0 0 0 0 0 0 0 0 0 0 0 0 0 0 0 0 0 0 0 0 0 0 0 0 0 0 0 0 0 0 0 0 0 0 0 0

K00878 52 40 107 47 276 216 64 348 84 124 195 86 28 72 108 27 101 222 59 44 106 150 124 195 12 30 21 9 38 292 0 80 50 64 83 77 37 54 90 122 168 145 28 103 96 144 59 230

K00879 0 0 0 0 0 0 0 0 0 0 0 0 0 0 0 0 0 0 0 0 0 0 0 0 0 0 0 0 0 0 0 0 0 0 0 0 0 0 0 0 0 0 0 0 0 0 0 0

K00880 0 0 0 0 0 0 0 0 0 0 0 0 0 0 0 0 0 0 0 0 0 0 0 0 0 0 0 0 0 0 0 0 0 0 0 0 0 0 0 0 0 0 0 0 0 0 0 0

K00881 0 0 0 0 0 0 0 0 0 0 0 0 0 0 0 0 0 0 0 0 0 0 0 0 0 0 0 0 0 0 0 0 0 0 0 0 0 0 0 0 0 0 0 0 0 0 0 0

K00882 0 0 0 0 0 0 0 0 0 0 0 0 0 0 0 0 0 0 0 0 0 0 0 0 0 0 0 0 0 0 0 0 0 0 0 0 0 0 0 0 0 0 0 0 0 0 0 0

K00883 0 0 0 0 0 0 0 0 0 0 0 0 0 0 0 0 0 0 0 0 0 0 0 0 0 0 0 0 0 0 0 0 0 0 0 0 0 0 0 0 0 0 0 0 0 0 0 0

K00884 0 0 0 0 0 0 0 0 0 0 0 0 0 0 0 0 0 0 0 0 0 0 0 0 0 0 0 0 0 0 0 0 0 0 0 0 0 0 0 0 0 0 0 0 0 0 0 0

K00885 0 0 0 0 0 0 0 0 0 0 0 0 0 0 0 0 0 0 0 0 0 0 0 0 0 0 0 0 0 0 0 0 0 0 0 0 0 0 0 0 0 0 0 0 0 0 0 0

K00886 0 0 0 0 0 0 0 0 0 0 0 0 0 0 0 0 0 0 0 0 0 0 0 0 0 0 0 0 0 0 0 0 0 0 0 0 0 0 0 0 0 0 0 0 0 0 0 0

K00887 0 0 0 0 0 0 0 0 0 0 0 0 0 0 0 0 0 0 0 0 0 0 0 0 0 0 0 0 0 0 0 0 0 0 0 0 0 0 0 0 0 0 0 0 0 0 0 0

K00891 437 524 486 214 843 666 340 1084 662 698 919 814 296 794 560 275 685 1187 584 618 853 970 1102 878 388 368 378 290 342 980 24 904 580 799 912 798 249 524 620 511 750 740 430 833 780 533 604 891

K00892 0 0 0 0 0 0 0 0 0 0 0 0 0 0 0 0 0 0 0 0 0 0 0 0 0 0 0 0 0 0 0 0 0 0 0 0 0 0 0 0 0 0 0 0 0 0 0 0

K00893 0 0 0 0 0 0 0 0 0 0 0 0 0 0 0 0 0 0 0 0 0 0 0 0 0 0 0 0 0 0 0 0 0 0 0 0 0 0 0 0 0 0 0 0 0 0 0 0

K00895 0 0 0 0 0 0 0 0 0 0 0 0 0 0 0 0 0 0 0 0 0 0 0 0 0 0 0 0 0 0 0 0 0 0 0 0 0 0 0 0 0 0 0 0 0 0 0 0

K00897 0 0 0 0 0 0 0 0 0 0 0 0 0 0 0 0 0 0 0 0 0 0 0 0 0 0 0 0 0 0 0 0 0 0 0 0 0 0 0 0 0 0 0 0 0 0 0 0

K00899 0 0 0 0 0 0 0 0 0 0 0 0 0 0 0 0 0 0 0 0 0 0 0 0 0 0 0 0 0 0 0 0 0 0 0 0 0 0 0 0 0 0 0 0 0 0 0 0

K00901 74 218 232 81 364 301 116 443 146 218 378 199 110 168 238 66 323 376 212 220 300 290 318 338 86 128 107 19 138 452 0 170 96 110 222 164 87 260 360 263 354 349 80 416 453 265 227 416

K00903 0 0 0 0 0 0 0 0 0 0 0 0 0 0 0 0 0 0 0 0 0 0 0 0 0 0 0 0 0 0 0 0 0 0 0 0 0 0 0 0 0 0 0 0 0 0 0 0

K00904 0 0 0 0 0 0 0 0 0 0 0 0 0 0 0 0 0 0 0 0 0 0 0 0 0 0 0 0 0 0 0 0 0 0 0 0 0 0 0 0 0 0 0 0 0 0 0 0

K00906 0 0 0 0 0 0 0 0 0 0 0 0 0 0 0 0 0 0 0 0 0 0 0 0 0 0 0 0 0 0 0 0 0 0 0 0 0 0 0 0 0 0 0 0 0 0 0 0

K00908 0 0 0 0 0 0 0 0 0 0 0 0 0 0 0 0 0 0 0 0 0 0 0 0 0 0 0 0 0 0 0 0 0 0 0 0 0 0 0 0 0 0 0 0 0 0 0 0

K00912 0 0 0 0 0 0 0 0 0 0 0 0 0 0 0 0 0 0 0 0 0 0 0 0 0 0 0 0 0 0 0 0 0 0 0 0 0 0 0 0 0 0 0 0 0 0 0 0

K00917 0 0 0 0 0 0 0 0 0 0 0 0 0 0 0 0 0 0 0 0 0 0 0 0 0 0 0 0 0 0 0 0 0 0 0 0 0 0 0 0 0 0 0 0 0 0 0 0

K00918 32 64 35 0 132 114 0 164 26 0 61 0 0 122 64 46 76 95 0 80 99 36 140 65 24 0 12 62 11 170 0 146 94 73 56 66 0 30 102 49 130 88 75 150 112 40 74 128

K00919 338 420 344 166 406 320 267 564 552 574 661 728 268 601 372 202 508 856 526 476 648 784 838 582 354 338 346 218 292 502 24 648 436 638 772 654 212 438 429 340 452 498 327 580 572 350 470 533

K00924 0 0 0 0 0 0 0 0 0 0 0 0 0 0 0 0 0 0 0 0 0 0 0 0 0 0 0 0 0 0 0 0 0 0 0 0 0 0 0 0 0 0 0 0 0 0 0 0

K00925 0 0 0 0 0 0 0 0 0 0 0 0 0 0 0 0 0 0 0 0 0 0 0 0 0 0 0 0 0 0 0 0 0 0 0 0 0 0 0 0 0 0 0 0 0 0 0 0

K00926 42 200 159 49 182 163 64 154 106 120 225 154 91 132 160 46 234 224 164 205 234 156 234 232 84 102 94 19 116 260 0 194 68 67 182 137 58 236 295 157 201 244 80 337 382 125 184 244

K00927 478 724 645 262 1002 814 404 1228 768 818 1142 968 386 926 719 320 919 1396 748 822 1088 1127 1335 1070 473 471 472 308 458 1219 24 1038 648 866 1094 934 308 759 916 668 952 984 510 1170 1161 658 788 1134

K00928 437 524 486 214 819 650 340 1074 662 698 917 814 296 794 560 275 685 1173 584 618 853 970 1102 855 388 368 378 290 342 963 24 874 580 799 912 798 249 524 620 511 750 740 430 833 780 533 604 891

K00929 0 0 0 0 0 0 0 0 0 0 0 0 0 0 0 0 0 0 0 0 0 0 0 0 0 0 0 0 0 0 0 0 0 0 0 0 0 0 0 0 0 0 0 0 0 0 0 0

K00930 842 984 937 427 1507 1186 679 1986 1298 1397 1772 1628 591 1468 1055 504 1294 2251 1169 1156 1607 1906 2064 1645 754 737 745 517 672 1756 48 1602 1067 1525 1768 1529 498 1016 1139 973 1370 1392 785 1516 1447 1026 1134 1654

K00931 338 420 344 166 406 320 267 564 552 574 661 728 268 601 372 202 508 856 526 476 648 784 838 582 354 338 346 218 292 502 24 648 436 638 772 654 212 438 429 340 452 498 327 580 572 350 470 533

K00932 0 0 0 0 0 0 0 0 0 0 0 0 0 0 0 0 0 0 0 0 0 0 0 0 0 0 0 0 0 0 0 0 0 0 0 0 0 0 0 0 0 0 0 0 0 0 0 0

K00933 0 0 0 0 0 0 0 0 0 0 0 0 0 0 0 0 0 0 0 0 0 0 0 0 0 0 0 0 0 0 0 0 0 0 0 0 0 0 0 0 0 0 0 0 0 0 0 0

K00934 0 0 0 0 0 0 0 0 0 0 0 0 0 0 0 0 0 0 0 0 0 0 0 0 0 0 0 0 0 0 0 0 0 0 0 0 0 0 0 0 0 0 0 0 0 0 0 0

K00935 0 0 0 0 0 0 0 0 0 0 0 0 0 0 0 0 0 0 0 0 0 0 0 0 0 0 0 0 0 0 0 0 0 0 0 0 0 0 0 0 0 0 0 0 0 0 0 0

K00936 676 839 688 333 812 641 534 1127 1103 1148 1325 1457 535 1202 744 403 1017 1712 1051 951 1297 1569 1677 1180 707 677 692 437 585 1008 48 1328 872 1276 1545 1309 424 877 858 680 904 997 654 1159 1143 699 941 1066

K00937 20 22 34 15 48 46 12 40 44 26 41 40 10 35 29 6 12 41 12 30 40 18 40 59 10 4 8 9 17 70 0 79 22 21 42 50 8 30 24 16 16 40 28 24 24 4 16 58

K00938 0 0 0 0 0 0 0 0 0 0 0 0 0 0 0 0 0 0 0 0 0 0 0 0 0 0 0 0 0 0 0 0 0 0 0 0 0 0 0 0 0 0 0 0 0 0 0 0

K00939 492 724 645 262 984 798 412 1219 768 818 1142 968 386 926 735 320 919 1382 748 841 1088 1127 1335 1077 473 471 472 308 458 1206 24 1038 648 890 1094 934 308 759 916 668 952 992 510 1170 1161 658 788 1134

K00940 478 724 645 262 978 798 404 1219 768 818 1142 968 386 926 719 320 919 1382 748 822 1088 1127 1335 1064 473 471 472 308 458 1206 24 1038 648 866 1094 934 308 759 916 668 952 984 510 1170 1161 658 788 1134

K00941 647 1028 946 358 1608 1306 557 1892 984 1062 1628 1208 506 1251 1098 440 1330 1937 972 1206 1526 1470 1832 1618 592 604 598 398 624 1945 24 1490 861 1143 1416 1214 403 1080 1402 996 1451 1486 692 1760 1750 966 1106 1736

K00942 338 420 344 166 406 320 267 564 552 574 661 728 268 601 372 202 508 856 526 476 648 784 838 582 354 338 346 218 292 502 24 648 436 638 772 654 212 438 429 340 452 498 327 580 572 350 470 533

K00943 545 764 752 310 1236 997 476 1558 852 942 1334 1054 414 998 842 348 1020 1590 808 885 1193 1278 1458 1249 484 501 493 318 496 1480 24 1089 699 954 1177 1012 344 814 1005 790 1120 1138 538 1273 1257 802 847 1365

K00944 0 0 0 0 0 0 0 0 0 0 0 0 0 0 0 0 0 0 0 0 0 0 0 0 0 0 0 0 0 0 0 0 0 0 0 0 0 0 0 0 0 0 0 0 0 0 0 0

K00945 478 724 645 262 978 798 404 1219 768 818 1142 968 386 926 719 320 919 1382 748 822 1088 1127 1335 1064 473 471 472 308 458 1206 24 1038 648 866 1094 934 308 759 916 668 952 984 510 1170 1161 658 788 1134

K00946 478 724 645 262 978 798 404 1219 768 818 1142 968 386 926 719 320 919 1382 748 822 1088 1127 1335 1064 473 471 472 308 458 1206 24 1038 648 866 1094 934 308 759 916 668 952 984 510 1170 1161 658 788 1134

K00947 0 0 0 0 0 0 0 0 0 0 0 0 0 0 0 0 0 0 0 0 0 0 0 0 0 0 0 0 0 0 0 0 0 0 0 0 0 0 0 0 0 0 0 0 0 0 0 0

K00948 478 724 645 262 978 798 404 1219 768 818 1142 968 386 926 719 320 919 1382 748 822 1088 1127 1335 1064 473 471 472 308 458 1206 24 1038 648 866 1094 934 308 759 916 668 952 984 510 1170 1161 658 788 1134

K00949 0 0 0 0 0 0 0 0 0 0 0 0 0 0 0 0 0 0 0 0 0 0 0 0 0 0 0 0 0 0 0 0 0 0 0 0 0 0 0 0 0 0 0 0 0 0 0 0

K00950 338 420 344 166 406 320 267 564 552 574 661 728 268 601 372 202 508 856 526 476 648 784 838 582 354 338 346 218 292 502 24 648 436 638 772 654 212 438 429 340 452 498 327 580 572 350 470 533

K00951 338 420 344 166 406 320 267 564 552 574 661 728 268 601 372 202 508 856 526 476 648 784 838 582 354 338 346 218 292 502 24 648 436 638 772 654 212 438 429 340 452 498 327 580 572 350 470 533

K00952 140 304 301 96 572 477 136 656 216 244 481 240 119 325 347 119 410 526 223 347 439 342 496 482 120 132 126 90 166 704 0 391 212 228 322 280 96 320 486 328 500 486 182 590 590 308 318 602

K00954 338 420 344 166 406 320 267 564 552 574 661 728 268 601 372 202 508 856 526 476 648 784 838 582 354 338 346 218 292 502 24 648 436 638 772 654 212 438 429 340 452 498 327 580 572 350 470 533

K00955 0 0 0 0 0 0 0 0 0 0 0 0 0 0 0 0 0 0 0 0 0 0 0 0 0 0 0 0 0 0 0 0 0 0 0 0 0 0 0 0 0 0 0 0 0 0 0 0

K00956 0 0 0 0 0 0 0 0 0 0 0 0 0 0 0 0 0 0 0 0 0 0 0 0 0 0 0 0 0 0 0 0 0 0 0 0 0 0 0 0 0 0 0 0 0 0 0 0

K00957 0 0 0 0 0 0 0 0 0 0 0 0 0 0 0 0 0 0 0 0 0 0 0 0 0 0 0 0 0 0 0 0 0 0 0 0 0 0 0 0 0 0 0 0 0 0 0 0

K00958 0 0 0 0 0 0 0 0 0 0 0 0 0 0 0 0 0 0 0 0 0 0 0 0 0 0 0 0 0 0 0 0 0 0 0 0 0 0 0 0 0 0 0 0 0 0 0 0

K00960 0 0 0 0 0 0 0 0 0 0 0 0 0 0 0 0 0 0 0 0 0 0 0 0 0 0 0 0 0 0 0 0 0 0 0 0 0 0 0 0 0 0 0 0 0 0 0 0

K00961 0 0 0 0 0 0 0 0 0 0 0 0 0 0 0 0 0 0 0 0 0 0 0 0 0 0 0 0 0 0 0 0 0 0 0 0 0 0 0 0 0 0 0 0 0 0 0 0

K00962 338 420 344 166 406 320 267 564 552 574 661 728 268 601 372 202 508 856 526 476 648 784 838 582 354 338 346 218 292 502 24 648 436 638 772 654 212 438 429 340 452 498 327 580 572 350 470 533

K00963 437 524 486 214 796 634 340 1066 662 698 917 814 296 794 560 275 685 1159 584 618 853 970 1102 832 388 368 378 290 342 946 24 844 580 799 912 798 249 524 620 511 750 740 430 833 780 533 604 891

K00964 0 0 0 0 0 0 0 0 0 0 0 0 0 0 0 0 0 0 0 0 0 0 0 0 0 0 0 0 0 0 0 0 0 0 0 0 0 0 0 0 0 0 0 0 0 0 0 0

K00965 0 0 0 0 0 0 0 0 0 0 0 0 0 0 0 0 0 0 0 0 0 0 0 0 0 0 0 0 0 0 0 0 0 0 0 0 0 0 0 0 0 0 0 0 0 0 0 0

K00966 394 508 398 192 521 434 315 636 592 630 851 778 323 702 451 247 608 986 666 607 789 916 1058 792 417 441 429 238 380 632 24 770 451 706 840 758 270 644 598 375 478 594 345 706 770 436 548 722

K00968 0 0 0 0 0 0 0 0 0 0 0 0 0 0 0 0 0 0 0 0 0 0 0 0 0 0 0 0 0 0 0 0 0 0 0 0 0 0 0 0 0 0 0 0 0 0 0 0

K00969 338 420 344 166 406 320 267 564 552 574 661 728 268 601 372 202 508 856 526 476 648 784 838 582 354 338 346 218 292 502 24 648 436 638 772 654 212 438 429 340 452 498 327 580 572 350 470 533

K00970 338 420 344 166 406 320 267 564 552 574 661 728 268 601 372 202 508 856 526 476 648 784 838 582 354 338 346 218 292 502 24 648 436 638 772 654 212 438 429 340 452 498 327 580 572 350 470 533

K00971 338 420 344 166 430 336 267 572 552 574 664 728 268 601 372 202 508 870 526 476 648 784 838 588 354 338 346 218 292 515 24 648 436 638 772 654 212 438 429 340 452 498 327 580 572 350 470 533

K00972 0 0 0 0 0 0 0 0 0 0 0 0 0 0 0 0 0 0 0 0 0 0 0 0 0 0 0 0 0 0 0 0 0 0 0 0 0 0 0 0 0 0 0 0 0 0 0 0

K00973 553 942 877 344 1365 1114 519 1671 914 1036 1523 1167 496 1094 957 386 1242 1773 960 1042 1388 1416 1652 1425 558 599 579 328 596 1676 24 1238 744 976 1316 1099 394 1019 1276 931 1305 1333 589 1586 1614 923 1015 1551

K00974 750 992 778 358 1027 854 574 1354 1170 1204 1570 1507 590 1425 871 495 1192 1923 1192 1144 1536 1736 2036 1402 794 780 787 518 683 1286 48 1530 981 1393 1670 1478 482 1112 1130 764 1060 1172 747 1436 1454 825 1094 1382

K00975 0 0 0 0 0 0 0 0 0 0 0 0 0 0 0 0 0 0 0 0 0 0 0 0 0 0 0 0 0 0 0 0 0 0 0 0 0 0 0 0 0 0 0 0 0 0 0 0

K00978 0 0 0 0 0 0 0 0 0 0 0 0 0 0 0 0 0 0 0 0 0 0 0 0 0 0 0 0 0 0 0 0 0 0 0 0 0 0 0 0 0 0 0 0 0 0 0 0

K00979 0 0 0 0 0 0 0 0 0 0 0 0 0 0 0 0 0 0 0 0 0 0 0 0 0 0 0 0 0 0 0 0 0 0 0 0 0 0 0 0 0 0 0 0 0 0 0 0

K00980 0 0 0 0 24 16 0 9 0 0 3 0 0 0 0 0 0 14 0 0 0 0 0 23 0 0 0 0 0 18 0 26 0 0 0 0 0 0 0 0 0 0 0 0 0 0 0 0

K00981 338 420 344 166 406 320 267 564 552 574 661 728 268 601 372 202 508 856 526 476 648 784 838 582 354 338 346 218 292 502 24 648 436 638 772 654 212 438 429 340 452 498 327 580 572 350 470 533

K00982 0 0 0 0 0 0 0 0 0 0 0 0 0 0 0 0 0 0 0 0 0 0 0 0 0 0 0 0 0 0 0 0 0 0 0 0 0 0 0 0 0 0 0 0 0 0 0 0

K00983 0 0 0 0 0 0 0 0 0 0 0 0 0 0 0 0 0 0 0 0 0 0 0 0 0 0 0 0 0 0 0 0 0 0 0 0 0 0 0 0 0 0 0 0 0 0 0 0

K00984 0 0 0 0 0 0 0 0 0 0 0 0 0 0 0 0 0 0 0 0 0 0 0 0 0 0 0 0 0 0 0 0 0 0 0 0 0 0 0 0 0 0 0 0 0 0 0 0

K00985 0 0 0 0 0 0 0 0 0 0 0 0 0 0 0 0 0 0 0 0 0 0 0 0 0 0 0 0 0 0 0 0 0 0 0 0 0 0 0 0 0 0 0 0 0 0 0 0

K00986 0 0 0 0 0 0 0 0 0 0 5 0 0 0 0 0 0 0 0 0 0 0 0 0 0 0 0 0 0 0 0 0 0 0 0 0 0 0 0 0 0 0 0 0 0 0 0 0

K00988 0 0 0 0 0 0 0 0 0 0 0 0 0 0 0 0 0 0 0 0 0 0 0 0 0 0 0 0 0 0 0 0 0 0 0 0 0 0 0 0 0 0 0 0 0 0 0 0

K00989 42 200 159 49 158 147 64 144 106 120 225 154 91 132 160 46 234 210 164 205 234 156 234 208 84 102 94 19 116 243 0 164 68 67 182 137 58 236 295 157 201 244 80 337 382 125 184 244

K00990 0 0 0 0 0 0 0 0 0 0 0 0 0 0 0 0 0 0 0 0 0 0 0 0 0 0 0 0 0 0 0 0 0 0 0 0 0 0 0 0 0 0 0 0 0 0 0 0

K00991 33 18 73 32 205 154 52 298 40 98 154 45 18 37 78 20 89 167 48 14 66 133 84 130 1 26 13 0 21 209 0 5 28 42 40 28 28 24 66 106 152 105 0 78 72 140 43 173

K00992 20 133 138 38 120 94 37 122 108 90 76 144 45 65 126 6 147 134 35 122 134 42 53 76 32 4 18 9 46 196 0 121 76 44 156 83 8 60 150 138 190 197 90 234 208 42 122 112

K00995 498 746 679 278 1048 859 416 1268 811 844 1180 1008 396 961 748 327 931 1438 760 852 1128 1144 1374 1112 484 476 480 318 475 1284 24 1084 671 887 1136 984 316 789 940 684 967 1024 538 1194 1186 662 804 1192

K00996 0 0 0 0 0 0 0 0 0 0 0 0 0 0 0 0 0 0 0 0 0 0 0 0 0 0 0 0 0 0 0 0 0 0 0 0 0 0 0 0 0 0 0 0 0 0 0 0

K00997 0 0 0 0 1 0 0 0 0 0 3 0 0 0 0 0 0 0 0 0 0 0 0 0 0 0 0 0 0 0 0 0 0 0 0 0 0 0 0 0 0 0 0 0 0 0 0 0

K00998 404 460 451 214 664 520 340 902 636 698 853 814 296 673 496 228 610 1064 584 538 754 935 962 767 365 368 367 228 330 776 24 698 486 726 856 732 249 493 518 462 620 652 355 682 668 493 530 764

K00999 0 0 0 0 0 0 0 0 0 0 0 0 0 0 0 0 0 0 0 0 0 0 0 0 0 0 0 0 0 0 0 0 0 0 0 0 0 0 0 0 0 0 0 0 0 0 0 0

K01000 404 460 451 214 664 520 340 902 636 698 853 814 296 673 496 228 610 1064 584 538 754 935 962 767 365 368 367 228 330 776 24 698 486 726 856 732 249 493 518 462 620 652 355 682 668 493 530 764

K01001 140 304 301 96 548 461 136 646 216 244 478 240 119 325 347 119 410 512 223 347 439 342 496 459 120 132 126 90 166 686 0 361 212 228 322 280 96 320 486 328 500 486 182 590 590 308 318 602

K01002 0 0 0 0 0 0 0 0 0 0 0 0 0 0 0 0 0 0 0 0 0 0 0 0 0 0 0 0 0 0 0 0 0 0 0 0 0 0 0 0 0 0 0 0 0 0 0 0

K01003 0 0 0 0 0 0 0 0 0 0 0 0 0 0 0 0 0 0 0 0 0 0 0 0 0 0 0 0 0 0 0 0 0 0 0 0 0 0 0 0 0 0 0 0 0 0 0 0

K01004 0 0 0 0 0 0 0 0 0 0 0 0 0 0 0 0 0 0 0 0 0 0 0 0 0 0 0 0 0 0 0 0 0 0 0 0 0 0 0 0 0 0 0 0 0 0 0 0

K01005 0 0 0 0 0 0 0 0 0 0 0 0 0 0 0 0 0 0 0 0 0 0 0 0 0 0 0 0 0 0 0 0 0 0 0 0 0 0 0 0 0 0 0 0 0 0 0 0

K01006 42 200 159 49 158 147 64 144 106 120 225 154 91 132 160 46 234 210 164 205 234 156 234 208 84 102 94 19 116 243 0 164 68 67 182 137 58 236 295 157 201 244 80 337 382 125 184 244

K01007 437 524 486 214 820 650 340 1074 662 698 917 814 296 794 560 275 685 1173 584 618 853 970 1102 855 388 368 378 290 342 963 24 874 580 799 912 798 249 524 620 511 750 740 430 833 780 533 604 891

K01008 0 0 0 0 0 0 0 0 0 0 0 0 0 0 0 0 0 0 0 0 0 0 0 0 0 0 0 0 0 0 0 0 0 0 0 0 0 0 0 0 0 0 0 0 0 0 0 0

K01010 0 0 0 0 0 0 0 0 0 0 0 0 0 0 0 0 0 0 0 0 0 0 0 0 0 0 0 0 0 0 0 0 0 0 0 0 0 0 0 0 0 0 0 0 0 0 0 0

K01011 398 484 379 166 572 451 284 736 578 574 722 728 268 722 468 248 584 965 526 592 748 820 978 680 377 338 358 280 304 684 24 794 530 760 829 720 212 469 531 389 582 604 402 730 684 390 545 660

K01012 492 720 684 280 1112 890 442 1468 764 890 1254 972 396 928 768 334 996 1494 784 808 1113 1242 1380 1128 464 492 478 300 462 1332 24 968 654 888 1092 912 328 754 957 758 1088 1049 482 1224 1208 794 815 1250

K01013 0 0 0 0 0 0 0 0 0 0 0 0 0 0 0 0 0 0 0 0 0 0 0 0 0 0 0 0 0 0 0 0 0 0 0 0 0 0 0 0 0 0 0 0 0 0 0 0

K01014 0 0 0 0 0 0 0 0 0 0 0 0 0 0 0 0 0 0 0 0 0 0 0 0 0 0 0 0 0 0 0 0 0 0 0 0 0 0 0 0 0 0 0 0 0 0 0 0

K01015 0 0 0 0 0 0 0 0 0 0 0 0 0 0 0 0 0 0 0 0 0 0 0 0 0 0 0 0 0 0 0 0 0 0 0 0 0 0 0 0 0 0 0 0 0 0 0 0

K01023 0 0 0 0 0 0 0 0 0 0 0 0 0 0 0 0 0 0 0 0 0 0 0 0 0 0 0 0 0 0 0 0 0 0 0 0 0 0 0 0 0 0 0 0 0 0 0 0

K01025 0 0 0 0 0 0 0 0 0 0 0 0 0 0 0 0 0 0 0 0 0 0 0 0 0 0 0 0 0 0 0 0 0 0 0 0 0 0 0 0 0 0 0 0 0 0 0 0

K01026 0 0 0 0 0 0 0 0 0 0 0 0 0 0 0 0 0 0 0 0 0 0 0 0 0 0 0 0 0 0 0 0 0 0 0 0 0 0 0 0 0 0 0 0 0 0 0 0

K01027 0 0 0 0 0 0 0 0 0 0 0 0 0 0 0 0 0 0 0 0 0 0 0 0 0 0 0 0 0 0 0 0 0 0 0 0 0 0 0 0 0 0 0 0 0 0 0 0

K01028 0 0 0 0 0 0 0 0 0 0 0 0 0 0 0 0 0 0 0 0 0 0 0 0 0 0 0 0 0 0 0 0 0 0 0 0 0 0 0 0 0 0 0 0 0 0 0 0

K01029 0 0 0 0 0 0 0 0 0 0 0 0 0 0 0 0 0 0 0 0 0 0 0 0 0 0 0 0 0 0 0 0 0 0 0 0 0 0 0 0 0 0 0 0 0 0 0 0

K01031 0 0 0 0 0 0 0 0 0 0 0 0 0 0 0 0 0 0 0 0 0 0 0 0 0 0 0 0 0 0 0 0 0 0 0 0 0 0 0 0 0 0 0 0 0 0 0 0

K01032 0 0 0 0 0 0 0 0 0 0 0 0 0 0 0 0 0 0 0 0 0 0 0 0 0 0 0 0 0 0 0 0 0 0 0 0 0 0 0 0 0 0 0 0 0 0 0 0

K01034 0 0 0 0 0 0 0 0 0 0 0 0 0 0 0 0 0 0 0 0 0 0 0 0 0 0 0 0 0 0 0 0 0 0 0 0 0 0 0 0 0 0 0 0 0 0 0 0

K01035 0 0 0 0 0 0 0 0 0 0 0 0 0 0 0 0 0 0 0 0 0 0 0 0 0 0 0 0 0 0 0 0 0 0 0 0 0 0 0 0 0 0 0 0 0 0 0 0

K01036 0 0 0 0 0 0 0 0 0 0 0 0 0 0 0 0 0 0 0 0 0 0 0 0 0 0 0 0 0 0 0 0 0 0 0 0 0 0 0 0 0 0 0 0 0 0 0 0

K01039 0 0 0 0 0 0 0 0 0 0 0 0 0 0 0 0 0 0 0 0 0 0 0 0 0 0 0 0 0 0 0 0 0 0 0 0 0 0 0 0 0 0 0 0 0 0 0 0

K01040 0 0 0 0 0 0 0 0 0 0 0 0 0 0 0 0 0 0 0 0 0 0 0 0 0 0 0 0 0 0 0 0 0 0 0 0 0 0 0 0 0 0 0 0 0 0 0 0

K01041 0 0 0 0 0 0 0 0 0 0 0 0 0 0 0 0 0 0 0 0 0 0 0 0 0 0 0 0 0 0 0 0 0 0 0 0 0 0 0 0 0 0 0 0 0 0 0 0

K01042 66 40 107 47 258 200 72 338 84 124 192 86 28 72 124 27 101 208 59 62 106 150 124 186 12 30 21 9 38 274 0 50 50 88 83 77 37 54 90 122 168 154 28 103 96 144 59 230

K01043 0 0 0 0 0 0 0 0 0 0 0 0 0 0 0 0 0 0 0 0 0 0 0 0 0 0 0 0 0 0 0 0 0 0 0 0 0 0 0 0 0 0 0 0 0 0 0 0

K01044 0 0 0 0 0 0 0 0 0 0 0 0 0 0 0 0 0 0 0 0 0 0 0 0 0 0 0 0 0 0 0 0 0 0 0 0 0 0 0 0 0 0 0 0 0 0 0 0

K01045 0 0 0 0 0 0 0 0 0 0 0 0 0 0 0 0 0 0 0 0 0 0 0 0 0 0 0 0 0 0 0 0 0 0 0 0 0 0 0 0 0 0 0 0 0 0 0 0

K01046 0 0 0 0 0 0 0 0 0 0 0 0 0 0 0 0 0 0 0 0 0 0 0 0 0 0 0 0 0 0 0 0 0 0 0 0 0 0 0 0 0 0 0 0 0 0 0 0

K01047 0 0 0 0 0 0 0 0 0 0 0 0 0 0 0 0 0 0 0 0 0 0 0 0 0 0 0 0 0 0 0 0 0 0 0 0 0 0 0 0 0 0 0 0 0 0 0 0

K01048 0 0 0 0 0 0 0 0 0 0 0 0 0 0 0 0 0 0 0 0 0 0 0 0 0 0 0 0 0 0 0 0 0 0 0 0 0 0 0 0 0 0 0 0 0 0 0 0

K01049 0 0 0 0 0 0 0 0 0 0 0 0 0 0 0 0 0 0 0 0 0 0 0 0 0 0 0 0 0 0 0 0 0 0 0 0 0 0 0 0 0 0 0 0 0 0 0 0

K01051 0 0 0 0 0 0 0 0 0 0 0 0 0 0 0 0 0 0 0 0 0 0 0 0 0 0 0 0 0 0 0 0 0 0 0 0 0 0 0 0 0 0 0 0 0 0 0 0

K01053 0 0 0 0 0 0 0 0 0 0 0 0 0 0 0 0 0 0 0 0 0 0 0 0 0 0 0 0 0 0 0 0 0 0 0 0 0 0 0 0 0 0 0 0 0 0 0 0

K01054 0 0 0 0 0 0 0 0 0 0 0 0 0 0 0 0 0 0 0 0 0 0 0 0 0 0 0 0 0 0 0 0 0 0 0 0 0 0 0 0 0 0 0 0 0 0 0 0

K01055 0 0 0 0 0 0 0 0 0 0 0 0 0 0 0 0 0 0 0 0 0 0 0 0 0 0 0 0 0 0 0 0 0 0 0 0 0 0 0 0 0 0 0 0 0 0 0 0

K01056 338 420 344 166 406 320 267 564 552 574 661 728 268 601 372 202 508 856 526 476 648 784 838 582 354 338 346 218 292 502 24 648 436 638 772 654 212 438 429 340 452 498 327 580 572 350 470 533

K01057 0 0 0 0 0 0 0 0 0 0 0 0 0 0 0 0 0 0 0 0 0 0 0 0 0 0 0 0 0 0 0 0 0 0 0 0 0 0 0 0 0 0 0 0 0 0 0 0

K01058 0 0 0 0 0 0 0 0 0 0 0 0 0 0 0 0 0 0 0 0 0 0 0 0 0 0 0 0 0 0 0 0 0 0 0 0 0 0 0 0 0 0 0 0 0 0 0 0

K01060 0 0 0 0 0 0 0 0 0 0 0 0 0 0 0 0 0 0 0 0 0 0 0 0 0 0 0 0 0 0 0 0 0 0 0 0 0 0 0 0 0 0 0 0 0 0 0 0

K01061 0 0 0 0 23 16 0 9 0 0 0 0 0 0 0 0 0 14 0 0 0 0 0 6 0 0 0 0 0 13 0 0 0 0 0 0 0 0 0 0 0 0 0 0 0 0 0 0

K01062 0 0 0 0 0 0 0 0 0 0 0 0 0 0 0 0 0 0 0 0 0 0 0 0 0 0 0 0 0 0 0 0 0 0 0 0 0 0 0 0 0 0 0 0 0 0 0 0

K01066 0 0 0 0 0 0 0 0 0 0 0 0 0 0 0 0 0 0 0 0 0 0 0 0 0 0 0 0 0 0 0 0 0 0 0 0 0 0 0 0 0 0 0 0 0 0 0 0

K01067 0 0 0 0 0 0 0 0 0 0 0 0 0 0 0 0 0 0 0 0 0 0 0 0 0 0 0 0 0 0 0 0 0 0 0 0 0 0 0 0 0 0 0 0 0 0 0 0

K01068 0 0 0 0 0 0 0 0 0 0 0 0 0 0 0 0 0 0 0 0 0 0 0 0 0 0 0 0 0 0 0 0 0 0 0 0 0 0 0 0 0 0 0 0 0 0 0 0

K01069 352 420 344 166 428 336 267 572 552 574 661 728 268 601 372 202 508 870 526 494 648 784 838 604 354 338 346 218 292 520 24 678 436 662 772 654 212 438 429 340 452 498 327 580 572 350 470 533

K01070 0 0 0 0 0 0 0 0 0 0 0 0 0 0 0 0 0 0 0 0 0 0 0 0 0 0 0 0 0 0 0 0 0 0 0 0 0 0 0 0 0 0 0 0 0 0 0 0

K01071 0 0 0 0 0 0 0 0 0 0 0 0 0 0 0 0 0 0 0 0 0 0 0 0 0 0 0 0 0 0 0 0 0 0 0 0 0 0 0 0 0 0 0 0 0 0 0 0

K01073 0 0 0 0 0 0 0 0 0 0 0 0 0 0 0 0 0 0 0 0 0 0 0 0 0 0 0 0 0 0 0 0 0 0 0 0 0 0 0 0 0 0 0 0 0 0 0 0

K01075 0 0 0 0 0 0 0 0 0 0 0 0 0 0 0 0 0 0 0 0 0 0 0 0 0 0 0 0 0 0 0 0 0 0 0 0 0 0 0 0 0 0 0 0 0 0 0 0

K01076 0 0 0 0 0 0 0 0 0 0 0 0 0 0 0 0 0 0 0 0 0 0 0 0 0 0 0 0 0 0 0 0 0 0 0 0 0 0 0 0 0 0 0 0 0 0 0 0

K01077 0 0 0 0 0 0 0 0 0 0 0 0 0 0 0 0 0 0 0 0 0 0 0 0 0 0 0 0 0 0 0 0 0 0 0 0 0 0 0 0 0 0 0 0 0 0 0 0

K01078 0 0 0 0 0 0 0 0 0 0 0 0 0 0 0 0 0 0 0 0 0 0 0 0 0 0 0 0 0 0 0 0 0 0 0 0 0 0 0 0 0 0 0 0 0 0 0 0

K01079 478 724 645 262 978 798 404 1219 768 818 1142 968 386 926 719 320 919 1382 748 822 1088 1127 1335 1064 473 471 472 308 458 1206 24 1038 648 866 1094 934 308 759 916 668 952 984 510 1170 1161 658 788 1134

K01080 0 0 0 0 0 0 0 0 0 0 0 0 0 0 0 0 0 0 0 0 0 0 0 0 0 0 0 0 0 0 0 0 0 0 0 0 0 0 0 0 0 0 0 0 0 0 0 0

K01081 0 0 0 0 24 16 0 9 0 0 3 0 0 0 0 0 0 14 0 0 0 0 0 23 0 0 0 0 0 18 0 30 0 0 0 0 0 0 0 0 0 0 0 0 0 0 0 0

K01082 0 0 0 0 0 0 0 0 0 0 0 0 0 0 0 0 0 0 0 0 0 0 0 0 0 0 0 0 0 0 0 0 0 0 0 0 0 0 0 0 0 0 0 0 0 0 0 0

K01083 0 0 0 0 0 0 0 0 0 0 0 0 0 0 0 0 0 0 0 0 0 0 0 0 0 0 0 0 0 0 0 0 0 0 0 0 0 0 0 0 0 0 0 0 0 0 0 0

K01084 0 0 0 0 0 0 0 0 0 0 0 0 0 0 0 0 0 0 0 0 0 0 0 0 0 0 0 0 0 0 0 0 0 0 0 0 0 0 0 0 0 0 0 0 0 0 0 0

K01085 0 0 0 0 0 0 0 0 0 0 0 0 0 0 0 0 0 0 0 0 0 0 0 0 0 0 0 0 0 0 0 0 0 0 0 0 0 0 0 0 0 0 0 0 0 0 0 0

K01087 14 0 0 0 30 16 8 9 0 0 0 0 0 0 16 0 0 14 0 18 0 0 0 20 0 0 0 0 0 13 0 0 0 24 0 0 0 0 0 0 0 8 0 0 0 0 0 0

K01089 0 0 0 0 0 0 0 0 0 0 0 0 0 0 0 0 0 0 0 0 0 0 0 0 0 0 0 0 0 0 0 0 0 0 0 0 0 0 0 0 0 0 0 0 0 0 0 0

K01090 412 684 538 216 718 598 331 880 684 694 950 882 358 854 596 294 818 1174 690 760 982 976 1212 878 462 441 451 300 420 932 24 988 598 778 1011 858 270 704 826 546 784 830 482 1067 1065 514 729 904

K01091 380 730 608 239 638 516 356 790 722 758 924 986 394 762 628 247 878 1159 713 772 977 966 1086 824 459 441 450 238 438 876 24 888 557 728 1068 825 270 704 850 619 828 900 468 1126 1136 514 760 832

K01092 446 706 572 230 772 644 352 920 727 720 988 923 368 889 640 300 830 1216 701 808 1022 994 1251 934 472 446 459 308 437 997 24 1034 620 824 1054 907 279 734 850 562 799 879 510 1092 1090 518 745 962

K01093 0 0 0 0 0 0 0 0 0 0 0 0 0 0 0 0 0 0 0 0 0 0 0 0 0 0 0 0 0 0 0 0 0 0 0 0 0 0 0 0 0 0 0 0 0 0 0 0

K01095 0 0 0 0 0 0 0 0 0 0 0 0 0 0 0 0 0 0 0 0 0 0 0 0 0 0 0 0 0 0 0 0 0 0 0 0 0 0 0 0 0 0 0 0 0 0 0 0

K01096 0 0 0 0 0 0 0 0 0 0 0 0 0 0 0 0 0 0 0 0 0 0 0 0 0 0 0 0 0 0 0 0 0 0 0 0 0 0 0 0 0 0 0 0 0 0 0 0

K01097 0 0 0 0 0 0 0 0 0 0 0 0 0 0 0 0 0 0 0 0 0 0 0 0 0 0 0 0 0 0 0 0 0 0 0 0 0 0 0 0 0 0 0 0 0 0 0 0

K01101 42 200 159 49 158 147 64 144 106 120 225 154 91 132 160 46 234 210 164 205 234 156 234 208 84 102 94 19 116 243 0 164 68 67 182 137 58 236 295 157 201 244 80 337 382 125 184 244

K01103 0 0 0 0 0 0 0 0 0 0 0 0 0 0 0 0 0 0 0 0 0 0 0 0 0 0 0 0 0 0 0 0 0 0 0 0 0 0 0 0 0 0 0 0 0 0 0 0

K01104 371 438 417 198 610 474 318 862 592 672 814 774 286 638 450 222 598 1023 573 490 714 918 922 712 354 364 359 218 314 711 24 652 464 680 813 682 240 463 494 446 604 604 327 658 643 490 514 706

K01112 0 0 0 0 0 0 0 0 0 0 0 0 0 0 0 0 0 0 0 0 0 0 0 0 0 0 0 0 0 0 0 0 0 0 0 0 0 0 0 0 0 0 0 0 0 0 0 0

K01113 0 0 0 0 0 0 0 0 0 0 0 0 0 0 0 0 0 0 0 0 0 0 0 0 0 0 0 0 0 0 0 0 0 0 0 0 0 0 0 0 0 0 0 0 0 0 0 0

K01114 0 0 0 0 0 0 0 0 0 0 0 0 0 0 0 0 0 0 0 0 0 0 0 0 0 0 0 0 0 0 0 0 0 0 0 0 0 0 0 0 0 0 0 0 0 0 0 0

K01115 0 0 0 0 0 0 0 0 0 0 0 0 0 0 0 0 0 0 0 0 0 0 0 0 0 0 0 0 0 0 0 0 0 0 0 0 0 0 0 0 0 0 0 0 0 0 0 0

K01117 0 0 0 0 0 0 0 0 0 0 0 0 0 0 0 0 0 0 0 0 0 0 0 0 0 0 0 0 0 0 0 0 0 0 0 0 0 0 0 0 0 0 0 0 0 0 0 0

K01118 0 0 0 0 0 0 0 0 0 0 0 0 0 0 0 0 0 0 0 0 0 0 0 0 0 0 0 0 0 0 0 0 0 0 0 0 0 0 0 0 0 0 0 0 0 0 0 0

K01119 0 0 0 0 0 0 0 0 0 0 0 0 0 0 0 0 0 0 0 0 0 0 0 0 0 0 0 0 0 0 0 0 0 0 0 0 0 0 0 0 0 0 0 0 0 0 0 0

K01120 0 0 0 0 0 0 0 0 0 0 0 0 0 0 0 0 0 0 0 0 0 0 0 0 0 0 0 0 0 0 0 0 0 0 0 0 0 0 0 0 0 0 0 0 0 0 0 0

K01121 0 0 0 0 0 0 0 0 0 0 0 0 0 0 0 0 0 0 0 0 0 0 0 0 0 0 0 0 0 0 0 0 0 0 0 0 0 0 0 0 0 0 0 0 0 0 0 0

K01126 412 795 642 239 792 647 356 962 748 758 985 986 394 884 692 294 953 1268 713 852 1076 1001 1225 896 482 441 462 300 450 1058 24 1037 651 801 1125 891 270 735 952 668 958 988 543 1277 1248 554 835 959

K01127 0 0 0 0 0 0 0 0 0 0 0 0 0 0 0 0 0 0 0 0 0 0 0 0 0 0 0 0 0 0 0 0 0 0 0 0 0 0 0 0 0 0 0 0 0 0 0 0

K01128 0 0 0 0 0 0 0 0 0 0 0 0 0 0 0 0 0 0 0 0 0 0 0 0 0 0 0 0 0 0 0 0 0 0 0 0 0 0 0 0 0 0 0 0 0 0 0 0

K01129 0 0 0 0 24 16 0 9 0 0 3 0 0 0 0 0 0 14 0 0 0 0 0 23 0 0 0 0 0 18 0 34 0 0 0 0 0 0 0 0 0 0 0 0 0 0 0 0

K01130 0 0 0 0 0 0 0 0 0 0 0 0 0 0 0 0 0 0 0 0 0 0 0 0 0 0 0 0 0 0 0 0 0 0 0 0 0 0 0 0 0 0 0 0 0 0 0 0

K01132 0 0 0 0 0 0 0 0 0 0 0 0 0 0 0 0 0 0 0 0 0 0 0 0 0 0 0 0 0 0 0 0 0 0 0 0 0 0 0 0 0 0 0 0 0 0 0 0

K01133 0 0 0 0 0 0 0 0 0 0 0 0 0 0 0 0 0 0 0 0 0 0 0 0 0 0 0 0 0 0 0 0 0 0 0 0 0 0 0 0 0 0 0 0 0 0 0 0

K01134 0 0 0 0 0 0 0 0 0 0 0 0 0 0 0 0 0 0 0 0 0 0 0 0 0 0 0 0 0 0 0 0 0 0 0 0 0 0 0 0 0 0 0 0 0 0 0 0

K01135 0 0 0 0 0 0 0 0 0 0 0 0 0 0 0 0 0 0 0 0 0 0 0 0 0 0 0 0 0 0 0 0 0 0 0 0 0 0 0 0 0 0 0 0 0 0 0 0

K01136 0 0 0 0 0 0 0 0 0 0 0 0 0 0 0 0 0 0 0 0 0 0 0 0 0 0 0 0 0 0 0 0 0 0 0 0 0 0 0 0 0 0 0 0 0 0 0 0

K01137 0 0 0 0 0 0 0 0 0 0 0 0 0 0 0 0 0 0 0 0 0 0 0 0 0 0 0 0 0 0 0 0 0 0 0 0 0 0 0 0 0 0 0 0 0 0 0 0

K01138 0 0 0 0 0 0 0 0 0 0 0 0 0 0 0 0 0 0 0 0 0 0 0 0 0 0 0 0 0 0 0 0 0 0 0 0 0 0 0 0 0 0 0 0 0 0 0 0

K01139 0 0 0 0 0 0 0 0 0 0 0 0 0 0 0 0 0 0 0 0 0 0 0 0 0 0 0 0 0 0 0 0 0 0 0 0 0 0 0 0 0 0 0 0 0 0 0 0

K01141 0 0 0 0 0 0 0 0 0 0 0 0 0 0 0 0 0 0 0 0 0 0 0 0 0 0 0 0 0 0 0 0 0 0 0 0 0 0 0 0 0 0 0 0 0 0 0 0

K01142 479 678 683 294 1028 821 455 1345 782 916 1234 1013 405 842 734 294 932 1440 796 758 1054 1224 1280 1106 450 496 474 246 468 1228 24 871 582 836 1078 896 336 753 879 725 974 1001 434 1098 1120 758 756 1180

K01143 0 0 0 0 0 0 0 0 0 0 0 0 0 0 0 0 0 0 0 0 0 0 0 0 0 0 0 0 0 0 0 0 0 0 0 0 0 0 0 0 0 0 0 0 0 0 0 0

K01144 0 0 0 0 0 0 0 0 0 0 0 0 0 0 0 0 0 0 0 0 0 0 0 0 0 0 0 0 0 0 0 0 0 0 0 0 0 0 0 0 0 0 0 0 0 0 0 0

K01145 0 0 0 0 0 0 0 0 0 0 0 0 0 0 0 0 0 0 0 0 0 0 0 0 0 0 0 0 0 0 0 0 0 0 0 0 0 0 0 0 0 0 0 0 0 0 0 0

K01146 0 0 0 0 0 0 0 0 0 0 0 0 0 0 0 0 0 0 0 0 0 0 0 0 0 0 0 0 0 0 0 0 0 0 0 0 0 0 0 0 0 0 0 0 0 0 0 0

K01147 0 0 0 0 24 16 0 9 0 0 3 0 0 0 0 0 0 14 0 0 0 0 0 23 0 0 0 0 0 18 0 30 0 0 0 0 0 0 0 0 0 0 0 0 0 0 0 0

K01150 0 0 0 0 0 0 0 0 0 0 0 0 0 0 0 0 0 0 0 0 0 0 0 0 0 0 0 0 0 0 0 0 0 0 0 0 0 0 0 0 0 0 0 0 0 0 0 0

K01151 478 724 645 262 978 798 404 1219 768 818 1142 968 386 926 719 320 919 1382 748 822 1088 1127 1335 1064 473 471 472 308 458 1206 24 1038 648 866 1094 934 308 759 916 668 952 984 510 1170 1161 658 788 1134

K01152 0 0 0 0 0 0 0 0 0 0 0 0 0 0 0 0 0 0 0 0 0 0 0 0 0 0 0 0 0 0 0 0 0 0 0 0 0 0 0 0 0 0 0 0 0 0 0 0

K01153 427 642 537 230 642 529 352 757 700 720 927 923 368 768 576 254 754 1120 701 747 923 958 1112 868 448 446 447 246 426 828 24 888 526 775 997 841 279 704 748 513 668 791 434 941 978 478 670 834

K01154 460 660 610 262 846 683 404 1056 741 818 1081 968 386 804 655 274 844 1288 748 762 988 1092 1196 998 450 471 460 246 447 1036 24 892 554 818 1038 868 308 728 814 619 821 896 434 1020 1049 618 714 1007

K01155 0 0 0 0 0 0 0 0 0 0 0 0 0 0 0 0 0 0 0 0 0 0 0 0 0 0 0 0 0 0 0 0 0 0 0 0 0 0 0 0 0 0 0 0 0 0 0 0

K01156 20 22 34 15 47 46 12 40 44 26 38 40 10 35 29 6 12 41 12 30 40 18 40 42 10 4 8 9 17 65 0 46 22 21 42 50 8 30 24 16 16 40 28 24 24 4 16 58

K01157 0 0 0 0 0 0 0 0 0 0 0 0 0 0 0 0 0 0 0 0 0 0 0 0 0 0 0 0 0 0 0 0 0 0 0 0 0 0 0 0 0 0 0 0 0 0 0 0

K01158 0 0 0 0 0 0 0 0 0 0 0 0 0 0 0 0 0 0 0 0 0 0 0 0 0 0 0 0 0 0 0 0 0 0 0 0 0 0 0 0 0 0 0 0 0 0 0 0

K01159 338 420 344 166 406 320 267 564 552 574 664 728 268 601 372 202 508 856 526 476 648 784 838 582 354 338 346 218 292 502 24 651 436 638 772 654 212 438 429 340 452 498 327 580 572 350 470 533

K01160 0 0 0 0 0 0 0 0 0 0 0 0 0 0 0 0 0 0 0 0 0 0 0 0 0 0 0 0 0 0 0 0 0 0 0 0 0 0 0 0 0 0 0 0 0 0 0 0

K01161 0 0 0 0 0 0 0 0 0 0 0 0 0 0 0 0 0 0 0 0 0 0 0 0 0 0 0 0 0 0 0 0 0 0 0 0 0 0 0 0 0 0 0 0 0 0 0 0

K01163 0 0 0 0 24 16 0 9 0 0 3 0 0 0 0 0 0 14 0 0 0 0 0 23 0 0 0 0 0 18 0 30 0 0 0 0 0 0 0 0 0 0 0 0 0 0 0 0

K01166 0 0 0 0 0 0 0 0 0 0 0 0 0 0 0 0 0 0 0 0 0 0 0 0 0 0 0 0 0 0 0 0 0 0 0 0 0 0 0 0 0 0 0 0 0 0 0 0

K01167 0 0 0 0 0 0 0 0 0 0 0 0 0 0 0 0 0 0 0 0 0 0 0 0 0 0 0 0 0 0 0 0 0 0 0 0 0 0 0 0 0 0 0 0 0 0 0 0

K01169 0 0 0 0 0 0 0 0 0 0 0 0 0 0 0 0 0 0 0 0 0 0 0 0 0 0 0 0 0 0 0 0 0 0 0 0 0 0 0 0 0 0 0 0 0 0 0 0

K01170 478 724 645 262 978 798 404 1219 768 818 1142 968 386 926 719 320 919 1382 748 822 1088 1127 1335 1064 473 471 472 308 458 1206 24 1038 648 866 1094 934 308 759 916 668 952 984 510 1170 1161 658 788 1134

K01172 0 0 0 0 0 0 0 0 0 0 0 0 0 0 0 0 0 0 0 0 0 0 0 0 0 0 0 0 0 0 0 0 0 0 0 0 0 0 0 0 0 0 0 0 0 0 0 0

K01173 0 0 0 0 0 0 0 0 0 0 0 0 0 0 0 0 0 0 0 0 0 0 0 0 0 0 0 0 0 0 0 0 0 0 0 0 0 0 0 0 0 0 0 0 0 0 0 0

K01174 0 0 0 0 0 0 0 0 0 0 0 0 0 0 0 0 0 0 0 0 0 0 0 0 0 0 0 0 0 0 0 0 0 0 0 0 0 0 0 0 0 0 0 0 0 0 0 0

K01175 0 0 0 0 0 0 0 0 0 0 0 0 0 0 0 0 0 0 0 0 0 0 0 0 0 0 0 0 0 0 0 0 0 0 0 0 0 0 0 0 0 0 0 0 0 0 0 0

K01176 0 0 0 0 0 0 0 0 0 0 0 0 0 0 0 0 0 0 0 0 0 0 0 0 0 0 0 0 0 0 0 0 0 0 0 0 0 0 0 0 0 0 0 0 0 0 0 0

K01178 0 0 0 0 0 0 0 0 0 0 0 0 0 0 0 0 0 0 0 0 0 0 0 0 0 0 0 0 0 0 0 0 0 0 0 0 0 0 0 0 0 0 0 0 0 0 0 0

K01179 478 724 645 262 978 798 404 1219 768 818 1142 968 386 926 719 320 919 1382 748 822 1088 1127 1335 1064 473 471 472 308 458 1206 24 1038 648 866 1094 934 308 759 916 668 952 984 510 1170 1161 658 788 1134

K01180 0 0 0 0 0 0 0 0 0 0 0 0 0 0 0 0 0 0 0 0 0 0 0 0 0 0 0 0 0 0 0 0 0 0 0 0 0 0 0 0 0 0 0 0 0 0 0 0

K01181 0 0 0 0 0 0 0 0 0 0 0 0 0 0 0 0 0 0 0 0 0 0 0 0 0 0 0 0 0 0 0 0 0 0 0 0 0 0 0 0 0 0 0 0 0 0 0 0

K01182 0 0 0 0 0 0 0 0 0 0 0 0 0 0 0 0 0 0 0 0 0 0 0 0 0 0 0 0 0 0 0 0 0 0 0 0 0 0 0 0 0 0 0 0 0 0 0 0

K01183 0 0 0 0 0 0 0 0 0 0 0 0 0 0 0 0 0 0 0 0 0 0 0 0 0 0 0 0 0 0 0 0 0 0 0 0 0 0 0 0 0 0 0 0 0 0 0 0

K01184 0 0 0 0 0 0 0 0 0 0 0 0 0 0 0 0 0 0 0 0 0 0 0 0 0 0 0 0 0 0 0 0 0 0 0 0 0 0 0 0 0 0 0 0 0 0 0 0

K01185 0 0 0 0 0 0 0 0 0 0 0 0 0 0 0 0 0 0 0 0 0 0 0 0 0 0 0 0 0 0 0 0 0 0 0 0 0 0 0 0 0 0 0 0 0 0 0 0

K01186 0 0 0 0 0 0 0 0 0 0 0 0 0 0 0 0 0 0 0 0 0 0 0 0 0 0 0 0 0 0 0 0 0 0 0 0 0 0 0 0 0 0 0 0 0 0 0 0

K01187 0 0 0 0 0 0 0 0 0 0 0 0 0 0 0 0 0 0 0 0 0 0 0 0 0 0 0 0 0 0 0 0 0 0 0 0 0 0 0 0 0 0 0 0 0 0 0 0

K01188 0 0 0 0 0 0 0 0 0 0 0 0 0 0 0 0 0 0 0 0 0 0 0 0 0 0 0 0 0 0 0 0 0 0 0 0 0 0 0 0 0 0 0 0 0 0 0 0

K01190 0 0 0 0 0 0 0 0 0 0 0 0 0 0 0 0 0 0 0 0 0 0 0 0 0 0 0 0 0 0 0 0 0 0 0 0 0 0 0 0 0 0 0 0 0 0 0 0

K01191 0 0 0 0 0 0 0 0 0 0 0 0 0 0 0 0 0 0 0 0 0 0 0 0 0 0 0 0 0 0 0 0 0 0 0 0 0 0 0 0 0 0 0 0 0 0 0 0

K01192 0 0 0 0 0 0 0 0 0 0 0 0 0 0 0 0 0 0 0 0 0 0 0 0 0 0 0 0 0 0 0 0 0 0 0 0 0 0 0 0 0 0 0 0 0 0 0 0

K01193 0 0 0 0 0 0 0 0 0 0 0 0 0 0 0 0 0 0 0 0 0 0 0 0 0 0 0 0 0 0 0 0 0 0 0 0 0 0 0 0 0 0 0 0 0 0 0 0

K01194 0 0 0 0 0 0 0 0 0 0 0 0 0 0 0 0 0 0 0 0 0 0 0 0 0 0 0 0 0 0 0 0 0 0 0 0 0 0 0 0 0 0 0 0 0 0 0 0

K01195 0 0 0 0 0 0 0 0 0 0 0 0 0 0 0 0 0 0 0 0 0 0 0 0 0 0 0 0 0 0 0 0 0 0 0 0 0 0 0 0 0 0 0 0 0 0 0 0

K01197 0 0 0 0 0 0 0 0 0 0 0 0 0 0 0 0 0 0 0 0 0 0 0 0 0 0 0 0 0 0 0 0 0 0 0 0 0 0 0 0 0 0 0 0 0 0 0 0

K01198 0 0 0 0 0 0 0 0 0 0 0 0 0 0 0 0 0 0 0 0 0 0 0 0 0 0 0 0 0 0 0 0 0 0 0 0 0 0 0 0 0 0 0 0 0 0 0 0

K01199 0 0 0 0 0 0 0 0 0 0 0 0 0 0 0 0 0 0 0 0 0 0 0 0 0 0 0 0 0 0 0 0 0 0 0 0 0 0 0 0 0 0 0 0 0 0 0 0

K01200 0 0 0 0 0 0 0 0 0 0 0 0 0 0 0 0 0 0 0 0 0 0 0 0 0 0 0 0 0 0 0 0 0 0 0 0 0 0 0 0 0 0 0 0 0 0 0 0

K01201 0 0 0 0 0 0 0 0 0 0 0 0 0 0 0 0 0 0 0 0 0 0 0 0 0 0 0 0 0 0 0 0 0 0 0 0 0 0 0 0 0 0 0 0 0 0 0 0

K01205 0 0 0 0 0 0 0 0 0 0 0 0 0 0 0 0 0 0 0 0 0 0 0 0 0 0 0 0 0 0 0 0 0 0 0 0 0 0 0 0 0 0 0 0 0 0 0 0

K01206 0 0 0 0 0 0 0 0 0 0 0 0 0 0 0 0 0 0 0 0 0 0 0 0 0 0 0 0 0 0 0 0 0 0 0 0 0 0 0 0 0 0 0 0 0 0 0 0

K01207 0 0 0 0 0 0 0 0 0 0 0 0 0 0 0 0 0 0 0 0 0 0 0 0 0 0 0 0 0 0 0 0 0 0 0 0 0 0 0 0 0 0 0 0 0 0 0 0

K01208 0 0 0 0 0 0 0 0 0 0 0 0 0 0 0 0 0 0 0 0 0 0 0 0 0 0 0 0 0 0 0 0 0 0 0 0 0 0 0 0 0 0 0 0 0 0 0 0

K01209 0 0 0 0 0 0 0 0 0 0 0 0 0 0 0 0 0 0 0 0 0 0 0 0 0 0 0 0 0 0 0 0 0 0 0 0 0 0 0 0 0 0 0 0 0 0 0 0

K01210 0 0 0 0 0 0 0 0 0 0 0 0 0 0 0 0 0 0 0 0 0 0 0 0 0 0 0 0 0 0 0 0 0 0 0 0 0 0 0 0 0 0 0 0 0 0 0 0

K01212 0 0 0 0 0 0 0 0 0 0 0 0 0 0 0 0 0 0 0 0 0 0 0 0 0 0 0 0 0 0 0 0 0 0 0 0 0 0 0 0 0 0 0 0 0 0 0 0

K01213 0 0 0 0 0 0 0 0 0 0 0 0 0 0 0 0 0 0 0 0 0 0 0 0 0 0 0 0 0 0 0 0 0 0 0 0 0 0 0 0 0 0 0 0 0 0 0 0

K01214 0 0 0 0 0 0 0 0 0 0 0 0 0 0 0 0 0 0 0 0 0 0 0 0 0 0 0 0 0 0 0 0 0 0 0 0 0 0 0 0 0 0 0 0 0 0 0 0

K01215 0 0 0 0 0 0 0 0 0 0 0 0 0 0 0 0 0 0 0 0 0 0 0 0 0 0 0 0 0 0 0 0 0 0 0 0 0 0 0 0 0 0 0 0 0 0 0 0

K01216 0 0 0 0 0 0 0 0 0 0 0 0 0 0 0 0 0 0 0 0 0 0 0 0 0 0 0 0 0 0 0 0 0 0 0 0 0 0 0 0 0 0 0 0 0 0 0 0

K01217 0 0 0 0 0 0 0 0 0 0 0 0 0 0 0 0 0 0 0 0 0 0 0 0 0 0 0 0 0 0 0 0 0 0 0 0 0 0 0 0 0 0 0 0 0 0 0 0

K01218 0 0 0 0 0 0 0 0 0 0 0 0 0 0 0 0 0 0 0 0 0 0 0 0 0 0 0 0 0 0 0 0 0 0 0 0 0 0 0 0 0 0 0 0 0 0 0 0

K01219 0 0 0 0 0 0 0 0 0 0 0 0 0 0 0 0 0 0 0 0 0 0 0 0 0 0 0 0 0 0 0 0 0 0 0 0 0 0 0 0 0 0 0 0 0 0 0 0

K01220 0 0 0 0 0 0 0 0 0 0 0 0 0 0 0 0 0 0 0 0 0 0 0 0 0 0 0 0 0 0 0 0 0 0 0 0 0 0 0 0 0 0 0 0 0 0 0 0

K01222 0 0 0 0 0 0 0 0 0 0 0 0 0 0 0 0 0 0 0 0 0 0 0 0 0 0 0 0 0 0 0 0 0 0 0 0 0 0 0 0 0 0 0 0 0 0 0 0

K01223 0 0 0 0 0 0 0 0 0 0 0 0 0 0 0 0 0 0 0 0 0 0 0 0 0 0 0 0 0 0 0 0 0 0 0 0 0 0 0 0 0 0 0 0 0 0 0 0

K01224 0 0 0 0 0 0 0 0 0 0 0 0 0 0 0 0 0 0 0 0 0 0 0 0 0 0 0 0 0 0 0 0 0 0 0 0 0 0 0 0 0 0 0 0 0 0 0 0

K01225 0 0 0 0 0 0 0 0 0 0 0 0 0 0 0 0 0 0 0 0 0 0 0 0 0 0 0 0 0 0 0 0 0 0 0 0 0 0 0 0 0 0 0 0 0 0 0 0

K01226 0 0 0 0 0 0 0 0 0 0 0 0 0 0 0 0 0 0 0 0 0 0 0 0 0 0 0 0 0 0 0 0 0 0 0 0 0 0 0 0 0 0 0 0 0 0 0 0

K01227 0 0 0 0 0 0 0 0 0 0 0 0 0 0 0 0 0 0 0 0 0 0 0 0 0 0 0 0 0 0 0 0 0 0 0 0 0 0 0 0 0 0 0 0 0 0 0 0

K01230 0 0 0 0 0 0 0 0 0 0 0 0 0 0 0 0 0 0 0 0 0 0 0 0 0 0 0 0 0 0 0 0 0 0 0 0 0 0 0 0 0 0 0 0 0 0 0 0

K01232 0 0 0 0 0 0 0 0 0 0 0 0 0 0 0 0 0 0 0 0 0 0 0 0 0 0 0 0 0 0 0 0 0 0 0 0 0 0 0 0 0 0 0 0 0 0 0 0

K01233 0 0 0 0 0 0 0 0 0 0 0 0 0 0 0 0 0 0 0 0 0 0 0 0 0 0 0 0 0 0 0 0 0 0 0 0 0 0 0 0 0 0 0 0 0 0 0 0

K01234 0 0 0 0 0 0 0 0 0 0 0 0 0 0 0 0 0 0 0 0 0 0 0 0 0 0 0 0 0 0 0 0 0 0 0 0 0 0 0 0 0 0 0 0 0 0 0 0

K01235 0 0 0 0 0 0 0 0 0 0 0 0 0 0 0 0 0 0 0 0 0 0 0 0 0 0 0 0 0 0 0 0 0 0 0 0 0 0 0 0 0 0 0 0 0 0 0 0

K01236 0 0 0 0 0 0 0 0 0 0 0 0 0 0 0 0 0 0 0 0 0 0 0 0 0 0 0 0 0 0 0 0 0 0 0 0 0 0 0 0 0 0 0 0 0 0 0 0

K01238 0 0 0 0 0 0 0 0 0 0 0 0 0 0 0 0 0 0 0 0 0 0 0 0 0 0 0 0 0 0 0 0 0 0 0 0 0 0 0 0 0 0 0 0 0 0 0 0

K01239 0 0 0 0 0 0 0 0 0 0 0 0 0 0 0 0 0 0 0 0 0 0 0 0 0 0 0 0 0 0 0 0 0 0 0 0 0 0 0 0 0 0 0 0 0 0 0 0

K01241 0 0 0 0 0 0 0 0 0 0 0 0 0 0 0 0 0 0 0 0 0 0 0 0 0 0 0 0 0 0 0 0 0 0 0 0 0 0 0 0 0 0 0 0 0 0 0 0

K01243 0 0 0 0 0 0 0 0 0 0 0 0 0 0 0 0 0 0 0 0 0 0 0 0 0 0 0 0 0 0 0 0 0 0 0 0 0 0 0 0 0 0 0 0 0 0 0 0

K01244 0 0 0 0 0 0 0 0 0 0 0 0 0 0 0 0 0 0 0 0 0 0 0 0 0 0 0 0 0 0 0 0 0 0 0 0 0 0 0 0 0 0 0 0 0 0 0 0

K01246 52 40 107 47 252 200 64 338 84 124 192 86 28 72 108 27 101 208 59 44 106 150 124 172 12 30 21 9 38 274 0 50 50 64 83 77 37 54 90 122 168 145 28 103 96 144 59 230

K01247 0 0 0 0 0 0 0 0 0 0 0 0 0 0 0 0 0 0 0 0 0 0 0 0 0 0 0 0 0 0 0 0 0 0 0 0 0 0 0 0 0 0 0 0 0 0 0 0

K01249 0 0 0 0 0 0 0 0 0 0 0 0 0 0 0 0 0 0 0 0 0 0 0 0 0 0 0 0 0 0 0 0 0 0 0 0 0 0 0 0 0 0 0 0 0 0 0 0

K01250 0 0 0 0 0 0 0 0 0 0 0 0 0 0 0 0 0 0 0 0 0 0 0 0 0 0 0 0 0 0 0 0 0 0 0 0 0 0 0 0 0 0 0 0 0 0 0 0

K01251 478 724 645 262 978 798 404 1219 768 818 1142 968 386 926 719 320 919 1382 748 822 1088 1127 1335 1080 473 471 472 308 458 1210 24 1068 648 866 1094 934 308 759 916 668 952 984 510 1170 1161 658 788 1134

K01252 0 0 0 0 0 0 0 0 0 0 0 0 0 0 0 0 0 0 0 0 0 0 0 0 0 0 0 0 0 0 0 0 0 0 0 0 0 0 0 0 0 0 0 0 0 0 0 0

K01253 0 0 0 0 23 16 0 9 0 0 0 0 0 0 0 0 0 14 0 0 0 0 0 6 0 0 0 0 0 13 0 0 0 0 0 0 0 0 0 0 0 0 0 0 0 0 0 0

K01255 338 420 344 166 406 320 267 564 552 574 661 728 268 601 372 202 508 856 526 476 648 784 838 582 354 338 346 218 292 502 24 648 436 638 772 654 212 438 429 340 452 498 327 580 572 350 470 533

K01256 0 0 0 0 23 16 0 9 0 0 0 0 0 0 0 0 0 14 0 0 0 0 0 23 0 0 0 0 0 18 0 30 0 0 0 0 0 0 0 0 0 0 0 0 0 0 0 0

K01258 0 0 0 0 0 0 0 0 0 0 0 0 0 0 0 0 0 0 0 0 0 0 0 0 0 0 0 0 0 0 0 0 0 0 0 0 0 0 0 0 0 0 0 0 0 0 0 0

K01259 0 0 0 0 0 0 0 0 0 0 3 0 0 0 0 0 0 0 0 0 0 0 0 0 0 0 0 0 0 0 0 4 0 0 0 0 0 0 0 0 0 0 0 0 0 0 0 0

K01261 0 0 0 0 0 0 0 0 0 0 0 0 0 0 0 0 0 0 0 0 0 0 0 0 0 0 0 0 0 0 0 0 0 0 0 0 0 0 0 0 0 0 0 0 0 0 0 0

K01262 338 420 344 166 459 352 276 582 552 574 666 728 268 601 388 202 508 884 526 476 648 784 838 641 354 338 346 218 292 537 24 704 436 638 772 654 212 438 429 340 452 507 327 580 572 350 470 533

K01263 0 0 0 0 0 0 0 0 0 0 0 0 0 0 0 0 0 0 0 0 0 0 0 0 0 0 0 0 0 0 0 0 0 0 0 0 0 0 0 0 0 0 0 0 0 0 0 0

K01264 0 0 0 0 0 0 0 0 0 0 0 0 0 0 0 0 0 0 0 0 0 0 0 0 0 0 0 0 0 0 0 0 0 0 0 0 0 0 0 0 0 0 0 0 0 0 0 0

K01265 478 724 645 262 978 798 404 1219 768 818 1142 968 386 926 719 320 919 1382 748 822 1088 1127 1335 1064 473 471 472 308 458 1206 24 1038 648 866 1094 934 308 759 916 668 952 984 510 1170 1161 658 788 1134

K01266 0 0 0 0 0 0 0 0 0 0 0 0 0 0 0 0 0 0 0 0 0 0 0 0 0 0 0 0 0 0 0 0 0 0 0 0 0 0 0 0 0 0 0 0 0 0 0 0

K01267 0 0 0 0 0 0 0 0 0 0 0 0 0 0 0 0 0 0 0 0 0 0 0 0 0 0 0 0 0 0 0 0 0 0 0 0 0 0 0 0 0 0 0 0 0 0 0 0

K01268 0 0 0 0 0 0 0 0 0 0 0 0 0 0 0 0 0 0 0 0 0 0 0 0 0 0 0 0 0 0 0 0 0 0 0 0 0 0 0 0 0 0 0 0 0 0 0 0

K01269 42 200 159 49 158 147 64 144 106 120 225 154 91 132 160 46 234 210 164 205 234 156 234 208 84 102 94 19 116 243 0 164 68 67 182 137 58 236 295 157 201 244 80 337 382 125 184 244

K01270 0 0 0 0 0 0 0 0 0 0 0 0 0 0 0 0 0 0 0 0 0 0 0 0 0 0 0 0 0 0 0 0 0 0 0 0 0 0 0 0 0 0 0 0 0 0 0 0

K01271 140 304 301 96 542 461 128 646 216 244 478 240 119 325 331 119 410 512 223 347 439 342 496 446 120 132 126 90 166 686 0 361 212 228 322 280 96 320 486 328 500 477 182 590 590 308 318 602

K01273 0 0 0 0 0 0 0 0 0 0 0 0 0 0 0 0 0 0 0 0 0 0 0 0 0 0 0 0 0 0 0 0 0 0 0 0 0 0 0 0 0 0 0 0 0 0 0 0

K01274 0 0 0 0 0 0 0 0 0 0 0 0 0 0 0 0 0 0 0 0 0 0 0 0 0 0 0 0 0 0 0 0 0 0 0 0 0 0 0 0 0 0 0 0 0 0 0 0

K01277 0 0 0 0 0 0 0 0 0 0 0 0 0 0 0 0 0 0 0 0 0 0 0 0 0 0 0 0 0 0 0 0 0 0 0 0 0 0 0 0 0 0 0 0 0 0 0 0

K01278 0 0 0 0 0 0 0 0 0 0 0 0 0 0 0 0 0 0 0 0 0 0 0 0 0 0 0 0 0 0 0 0 0 0 0 0 0 0 0 0 0 0 0 0 0 0 0 0

K01280 0 0 0 0 0 0 0 0 0 0 0 0 0 0 0 0 0 0 0 0 0 0 0 0 0 0 0 0 0 0 0 0 0 0 0 0 0 0 0 0 0 0 0 0 0 0 0 0

K01281 0 0 0 0 0 0 0 0 0 0 0 0 0 0 0 0 0 0 0 0 0 0 0 0 0 0 0 0 0 0 0 0 0 0 0 0 0 0 0 0 0 0 0 0 0 0 0 0

K01282 0 0 0 0 0 0 0 0 0 0 0 0 0 0 0 0 0 0 0 0 0 0 0 0 0 0 0 0 0 0 0 0 0 0 0 0 0 0 0 0 0 0 0 0 0 0 0 0

K01283 0 0 0 0 0 0 0 0 0 0 0 0 0 0 0 0 0 0 0 0 0 0 0 0 0 0 0 0 0 0 0 0 0 0 0 0 0 0 0 0 0 0 0 0 0 0 0 0

K01284 0 0 0 0 0 0 0 0 0 0 0 0 0 0 0 0 0 0 0 0 0 0 0 0 0 0 0 0 0 0 0 0 0 0 0 0 0 0 0 0 0 0 0 0 0 0 0 0

K01286 0 0 0 0 0 0 0 0 0 0 0 0 0 0 0 0 0 0 0 0 0 0 0 0 0 0 0 0 0 0 0 0 0 0 0 0 0 0 0 0 0 0 0 0 0 0 0 0

K01289 0 0 0 0 0 0 0 0 0 0 0 0 0 0 0 0 0 0 0 0 0 0 0 0 0 0 0 0 0 0 0 0 0 0 0 0 0 0 0 0 0 0 0 0 0 0 0 0

K01295 0 0 0 0 0 0 0 0 0 0 0 0 0 0 0 0 0 0 0 0 0 0 0 0 0 0 0 0 0 0 0 0 0 0 0 0 0 0 0 0 0 0 0 0 0 0 0 0

K01297 0 0 0 0 0 0 0 0 0 0 0 0 0 0 0 0 0 0 0 0 0 0 0 0 0 0 0 0 0 0 0 0 0 0 0 0 0 0 0 0 0 0 0 0 0 0 0 0

K01299 42 200 159 49 158 147 64 144 106 120 225 154 91 132 160 46 234 210 164 205 234 156 234 208 84 102 94 19 116 243 0 164 68 67 182 137 58 236 295 157 201 244 80 337 382 125 184 244

K01301 0 0 0 0 0 0 0 0 0 0 0 0 0 0 0 0 0 0 0 0 0 0 0 0 0 0 0 0 0 0 0 0 0 0 0 0 0 0 0 0 0 0 0 0 0 0 0 0

K01303 0 0 0 0 0 0 0 0 0 0 0 0 0 0 0 0 0 0 0 0 0 0 0 0 0 0 0 0 0 0 0 0 0 0 0 0 0 0 0 0 0 0 0 0 0 0 0 0

K01304 42 200 159 49 158 147 64 144 106 120 225 154 91 132 160 46 234 210 164 205 234 156 234 208 84 102 94 19 116 243 0 164 68 67 182 137 58 236 295 157 201 244 80 337 382 125 184 244

K01305 0 0 0 0 0 0 0 0 0 0 0 0 0 0 0 0 0 0 0 0 0 0 0 0 0 0 0 0 0 0 0 0 0 0 0 0 0 0 0 0 0 0 0 0 0 0 0 0

K01308 0 0 0 0 0 0 0 0 0 0 0 0 0 0 0 0 0 0 0 0 0 0 0 0 0 0 0 0 0 0 0 0 0 0 0 0 0 0 0 0 0 0 0 0 0 0 0 0

K01312 0 0 0 0 0 0 0 0 0 0 0 0 0 0 0 0 0 0 0 0 0 0 0 0 0 0 0 0 0 0 0 0 0 0 0 0 0 0 0 0 0 0 0 0 0 0 0 0

K01315 0 0 0 0 0 0 0 0 0 0 0 0 0 0 0 0 0 0 0 0 0 0 0 0 0 0 0 0 0 0 0 0 0 0 0 0 0 0 0 0 0 0 0 0 0 0 0 0

K01317 0 0 0 0 0 0 0 0 0 0 0 0 0 0 0 0 0 0 0 0 0 0 0 0 0 0 0 0 0 0 0 0 0 0 0 0 0 0 0 0 0 0 0 0 0 0 0 0

K01318 0 0 0 0 0 0 0 0 0 0 0 0 0 0 0 0 0 0 0 0 0 0 0 0 0 0 0 0 0 0 0 0 0 0 0 0 0 0 0 0 0 0 0 0 0 0 0 0

K01319 0 0 0 0 0 0 0 0 0 0 0 0 0 0 0 0 0 0 0 0 0 0 0 0 0 0 0 0 0 0 0 0 0 0 0 0 0 0 0 0 0 0 0 0 0 0 0 0

K01322 0 0 0 0 0 0 0 0 0 0 0 0 0 0 0 0 0 0 0 0 0 0 0 0 0 0 0 0 0 0 0 0 0 0 0 0 0 0 0 0 0 0 0 0 0 0 0 0

K01325 0 0 0 0 0 0 0 0 0 0 0 0 0 0 0 0 0 0 0 0 0 0 0 0 0 0 0 0 0 0 0 0 0 0 0 0 0 0 0 0 0 0 0 0 0 0 0 0

K01326 0 0 0 0 0 0 0 0 0 0 0 0 0 0 0 0 0 0 0 0 0 0 0 0 0 0 0 0 0 0 0 0 0 0 0 0 0 0 0 0 0 0 0 0 0 0 0 0

K01337 0 0 0 0 0 0 0 0 0 0 0 0 0 0 0 0 0 0 0 0 0 0 0 0 0 0 0 0 0 0 0 0 0 0 0 0 0 0 0 0 0 0 0 0 0 0 0 0

K01338 413 642 537 230 642 529 352 757 700 720 927 923 368 768 576 254 754 1120 701 728 923 958 1112 868 448 446 447 246 426 828 24 888 526 750 997 841 279 704 748 513 668 791 434 941 978 478 670 834

K01340 0 0 0 0 0 0 0 0 0 0 0 0 0 0 0 0 0 0 0 0 0 0 0 0 0 0 0 0 0 0 0 0 0 0 0 0 0 0 0 0 0 0 0 0 0 0 0 0

K01342 0 0 0 0 0 0 0 0 0 0 0 0 0 0 0 0 0 0 0 0 0 0 0 0 0 0 0 0 0 0 0 0 0 0 0 0 0 0 0 0 0 0 0 0 0 0 0 0

K01343 0 0 0 0 0 0 0 0 0 0 0 0 0 0 0 0 0 0 0 0 0 0 0 0 0 0 0 0 0 0 0 0 0 0 0 0 0 0 0 0 0 0 0 0 0 0 0 0

K01344 0 0 0 0 0 0 0 0 0 0 0 0 0 0 0 0 0 0 0 0 0 0 0 0 0 0 0 0 0 0 0 0 0 0 0 0 0 0 0 0 0 0 0 0 0 0 0 0

K01346 0 0 0 0 0 0 0 0 0 0 0 0 0 0 0 0 0 0 0 0 0 0 0 0 0 0 0 0 0 0 0 0 0 0 0 0 0 0 0 0 0 0 0 0 0 0 0 0

K01347 0 0 0 0 0 0 0 0 0 0 0 0 0 0 0 0 0 0 0 0 0 0 0 0 0 0 0 0 0 0 0 0 0 0 0 0 0 0 0 0 0 0 0 0 0 0 0 0

K01354 0 0 0 0 0 0 0 0 0 0 0 0 0 0 0 0 0 0 0 0 0 0 0 0 0 0 0 0 0 0 0 0 0 0 0 0 0 0 0 0 0 0 0 0 0 0 0 0

K01355 0 0 0 0 0 0 0 0 0 0 0 0 0 0 0 0 0 0 0 0 0 0 0 0 0 0 0 0 0 0 0 0 0 0 0 0 0 0 0 0 0 0 0 0 0 0 0 0

K01356 338 420 344 166 406 320 267 564 552 574 661 728 268 601 372 202 508 856 526 476 648 784 838 582 354 338 346 218 292 502 24 648 436 638 772 654 212 438 429 340 452 498 327 580 572 350 470 533

K01358 338 420 344 166 406 320 267 564 552 574 661 728 268 601 372 202 508 856 526 476 648 784 838 582 354 338 346 218 292 502 24 648 436 638 772 654 212 438 429 340 452 498 327 580 572 350 470 533

K01360 0 0 0 0 0 0 0 0 0 0 0 0 0 0 0 0 0 0 0 0 0 0 0 0 0 0 0 0 0 0 0 0 0 0 0 0 0 0 0 0 0 0 0 0 0 0 0 0

K01361 0 0 0 0 0 0 0 0 0 0 0 0 0 0 0 0 0 0 0 0 0 0 0 0 0 0 0 0 0 0 0 0 0 0 0 0 0 0 0 0 0 0 0 0 0 0 0 0

K01362 690 839 688 333 840 657 542 1136 1103 1148 1322 1457 535 1202 760 403 1017 1726 1051 970 1297 1569 1677 1182 707 677 692 437 585 1017 48 1295 872 1300 1545 1309 424 877 858 680 904 1006 654 1159 1143 699 941 1066

K01364 0 0 0 0 0 0 0 0 0 0 0 0 0 0 0 0 0 0 0 0 0 0 0 0 0 0 0 0 0 0 0 0 0 0 0 0 0 0 0 0 0 0 0 0 0 0 0 0

K01365 0 0 0 0 0 0 0 0 0 0 0 0 0 0 0 0 0 0 0 0 0 0 0 0 0 0 0 0 0 0 0 0 0 0 0 0 0 0 0 0 0 0 0 0 0 0 0 0

K01372 0 0 0 0 0 0 0 0 0 0 0 0 0 0 0 0 0 0 0 0 0 0 0 0 0 0 0 0 0 0 0 0 0 0 0 0 0 0 0 0 0 0 0 0 0 0 0 0

K01376 0 0 0 0 0 0 0 0 0 0 0 0 0 0 0 0 0 0 0 0 0 0 0 0 0 0 0 0 0 0 0 0 0 0 0 0 0 0 0 0 0 0 0 0 0 0 0 0

K01385 0 0 0 0 0 0 0 0 0 0 0 0 0 0 0 0 0 0 0 0 0 0 0 0 0 0 0 0 0 0 0 0 0 0 0 0 0 0 0 0 0 0 0 0 0 0 0 0

K01387 0 0 0 0 0 0 0 0 0 0 0 0 0 0 0 0 0 0 0 0 0 0 0 0 0 0 0 0 0 0 0 0 0 0 0 0 0 0 0 0 0 0 0 0 0 0 0 0

K01389 0 0 0 0 0 0 0 0 0 0 0 0 0 0 0 0 0 0 0 0 0 0 0 0 0 0 0 0 0 0 0 0 0 0 0 0 0 0 0 0 0 0 0 0 0 0 0 0

K01390 0 0 0 0 0 0 0 0 0 0 0 0 0 0 0 0 0 0 0 0 0 0 0 0 0 0 0 0 0 0 0 0 0 0 0 0 0 0 0 0 0 0 0 0 0 0 0 0

K01392 0 0 0 0 1 0 0 0 0 0 0 0 0 0 0 0 0 0 0 0 0 0 0 0 0 0 0 0 0 0 0 0 0 0 0 0 0 0 0 0 0 0 0 0 0 0 0 0

K01399 0 0 0 0 0 0 0 0 0 0 0 0 0 0 0 0 0 0 0 0 0 0 0 0 0 0 0 0 0 0 0 0 0 0 0 0 0 0 0 0 0 0 0 0 0 0 0 0

K01400 0 0 0 0 0 0 0 0 0 0 0 0 0 0 0 0 0 0 0 0 0 0 0 0 0 0 0 0 0 0 0 0 0 0 0 0 0 0 0 0 0 0 0 0 0 0 0 0

K01401 0 0 0 0 0 0 0 0 0 0 0 0 0 0 0 0 0 0 0 0 0 0 0 0 0 0 0 0 0 0 0 0 0 0 0 0 0 0 0 0 0 0 0 0 0 0 0 0

K01406 0 0 0 0 0 0 0 0 0 0 0 0 0 0 0 0 0 0 0 0 0 0 0 0 0 0 0 0 0 0 0 0 0 0 0 0 0 0 0 0 0 0 0 0 0 0 0 0

K01407 0 0 0 0 0 0 0 0 0 0 0 0 0 0 0 0 0 0 0 0 0 0 0 0 0 0 0 0 0 0 0 0 0 0 0 0 0 0 0 0 0 0 0 0 0 0 0 0

K01408 0 0 0 0 0 0 0 0 0 0 0 0 0 0 0 0 0 0 0 0 0 0 0 0 0 0 0 0 0 0 0 0 0 0 0 0 0 0 0 0 0 0 0 0 0 0 0 0

K01409 412 684 538 216 719 598 331 880 684 694 950 882 358 854 596 294 818 1174 690 760 982 976 1212 895 462 441 451 300 420 936 24 1011 598 778 1011 858 270 704 826 546 784 830 482 1067 1065 514 729 904

K01412 0 0 0 0 0 0 0 0 0 0 0 0 0 0 0 0 0 0 0 0 0 0 0 0 0 0 0 0 0 0 0 0 0 0 0 0 0 0 0 0 0 0 0 0 0 0 0 0

K01414 0 0 0 0 0 0 0 0 0 0 0 0 0 0 0 0 0 0 0 0 0 0 0 0 0 0 0 0 0 0 0 0 0 0 0 0 0 0 0 0 0 0 0 0 0 0 0 0

K01415 0 0 0 0 0 0 0 0 0 0 0 0 0 0 0 0 0 0 0 0 0 0 0 0 0 0 0 0 0 0 0 0 0 0 0 0 0 0 0 0 0 0 0 0 0 0 0 0

K01416 0 0 0 0 0 0 0 0 0 0 0 0 0 0 0 0 0 0 0 0 0 0 0 0 0 0 0 0 0 0 0 0 0 0 0 0 0 0 0 0 0 0 0 0 0 0 0 0

K01417 338 420 344 166 406 320 267 564 552 574 661 728 268 601 372 202 508 856 526 476 648 784 838 582 354 338 346 218 292 502 24 648 436 638 772 654 212 438 429 340 452 498 327 580 572 350 470 533

K01419 0 0 0 0 0 0 0 0 0 0 0 0 0 0 0 0 0 0 0 0 0 0 0 0 0 0 0 0 0 0 0 0 0 0 0 0 0 0 0 0 0 0 0 0 0 0 0 0

K01420 338 420 344 166 406 320 267 564 552 574 661 728 268 601 372 202 508 856 526 476 648 784 838 582 354 338 346 218 292 502 24 648 436 638 772 654 212 438 429 340 452 498 327 580 572 350 470 533

K01421 100 58 180 79 464 354 124 637 124 223 346 130 46 109 202 48 190 375 106 77 171 284 208 316 12 56 34 9 59 483 0 56 78 130 124 104 66 79 155 228 320 258 28 182 168 284 102 404

K01422 0 0 0 0 0 0 0 0 0 0 0 0 0 0 0 0 0 0 0 0 0 0 0 0 0 0 0 0 0 0 0 0 0 0 0 0 0 0 0 0 0 0 0 0 0 0 0 0

K01423 0 0 0 0 0 0 0 0 0 0 0 0 0 0 0 0 0 0 0 0 0 0 0 0 0 0 0 0 0 0 0 0 0 0 0 0 0 0 0 0 0 0 0 0 0 0 0 0

K01424 0 0 0 0 0 0 0 0 0 0 0 0 0 0 0 0 0 0 0 0 0 0 0 0 0 0 0 0 0 0 0 0 0 0 0 0 0 0 0 0 0 0 0 0 0 0 0 0

K01425 0 0 0 0 0 0 0 0 0 0 0 0 0 0 0 0 0 0 0 0 0 0 0 0 0 0 0 0 0 0 0 0 0 0 0 0 0 0 0 0 0 0 0 0 0 0 0 0

K01426 0 0 0 0 0 0 0 0 0 0 0 0 0 0 0 0 0 0 0 0 0 0 0 0 0 0 0 0 0 0 0 0 0 0 0 0 0 0 0 0 0 0 0 0 0 0 0 0

K01427 0 0 0 0 0 0 0 0 0 0 0 0 0 0 0 0 0 0 0 0 0 0 0 0 0 0 0 0 0 0 0 0 0 0 0 0 0 0 0 0 0 0 0 0 0 0 0 0

K01428 0 0 0 0 0 0 0 0 0 0 0 0 0 0 0 0 0 0 0 0 0 0 0 0 0 0 0 0 0 0 0 0 0 0 0 0 0 0 0 0 0 0 0 0 0 0 0 0

K01429 0 0 0 0 0 0 0 0 0 0 0 0 0 0 0 0 0 0 0 0 0 0 0 0 0 0 0 0 0 0 0 0 0 0 0 0 0 0 0 0 0 0 0 0 0 0 0 0

K01430 0 0 0 0 0 0 0 0 0 0 0 0 0 0 0 0 0 0 0 0 0 0 0 0 0 0 0 0 0 0 0 0 0 0 0 0 0 0 0 0 0 0 0 0 0 0 0 0

K01431 0 0 0 0 0 0 0 0 0 0 0 0 0 0 0 0 0 0 0 0 0 0 0 0 0 0 0 0 0 0 0 0 0 0 0 0 0 0 0 0 0 0 0 0 0 0 0 0

K01432 0 0 0 0 0 0 0 0 0 0 0 0 0 0 0 0 0 0 0 0 0 0 0 0 0 0 0 0 0 0 0 0 0 0 0 0 0 0 0 0 0 0 0 0 0 0 0 0

K01433 0 0 0 0 0 0 0 0 0 0 0 0 0 0 0 0 0 0 0 0 0 0 0 0 0 0 0 0 0 0 0 0 0 0 0 0 0 0 0 0 0 0 0 0 0 0 0 0

K01434 0 0 0 0 0 0 0 0 0 0 0 0 0 0 0 0 0 0 0 0 0 0 0 0 0 0 0 0 0 0 0 0 0 0 0 0 0 0 0 0 0 0 0 0 0 0 0 0

K01436 0 0 0 0 0 0 0 0 0 0 0 0 0 0 0 0 0 0 0 0 0 0 0 0 0 0 0 0 0 0 0 0 0 0 0 0 0 0 0 0 0 0 0 0 0 0 0 0

K01437 0 0 0 0 0 0 0 0 0 0 0 0 0 0 0 0 0 0 0 0 0 0 0 0 0 0 0 0 0 0 0 0 0 0 0 0 0 0 0 0 0 0 0 0 0 0 0 0

K01438 0 0 0 0 0 0 0 0 0 0 0 0 0 0 0 0 0 0 0 0 0 0 0 0 0 0 0 0 0 0 0 0 0 0 0 0 0 0 0 0 0 0 0 0 0 0 0 0

K01439 412 795 642 239 792 647 356 962 748 758 988 986 394 884 692 294 953 1268 713 852 1076 1001 1225 912 482 441 462 300 450 1063 24 1064 651 801 1125 891 270 735 952 668 958 988 543 1277 1248 554 835 959

K01442 52 40 107 47 252 200 64 338 84 124 192 86 28 72 108 27 101 208 59 44 106 150 124 172 12 30 21 9 38 274 0 50 50 64 83 77 37 54 90 122 168 145 28 103 96 144 59 230

K01443 0 0 0 0 0 0 0 0 0 0 0 0 0 0 0 0 0 0 0 0 0 0 0 0 0 0 0 0 0 0 0 0 0 0 0 0 0 0 0 0 0 0 0 0 0 0 0 0

K01444 0 0 0 0 0 0 0 0 0 0 0 0 0 0 0 0 0 0 0 0 0 0 0 0 0 0 0 0 0 0 0 0 0 0 0 0 0 0 0 0 0 0 0 0 0 0 0 0

K01446 0 0 0 0 0 0 0 0 0 0 0 0 0 0 0 0 0 0 0 0 0 0 0 0 0 0 0 0 0 0 0 0 0 0 0 0 0 0 0 0 0 0 0 0 0 0 0 0

K01447 0 0 0 0 0 0 0 0 0 0 0 0 0 0 0 0 0 0 0 0 0 0 0 0 0 0 0 0 0 0 0 0 0 0 0 0 0 0 0 0 0 0 0 0 0 0 0 0

K01448 338 420 344 166 406 320 267 564 552 574 661 728 268 601 372 202 508 856 526 476 648 784 838 582 354 338 346 218 292 502 24 648 436 638 772 654 212 438 429 340 452 498 327 580 572 350 470 533

K01449 0 0 0 0 0 0 0 0 0 0 0 0 0 0 0 0 0 0 0 0 0 0 0 0 0 0 0 0 0 0 0 0 0 0 0 0 0 0 0 0 0 0 0 0 0 0 0 0

K01450 0 0 0 0 0 0 0 0 0 0 0 0 0 0 0 0 0 0 0 0 0 0 0 0 0 0 0 0 0 0 0 0 0 0 0 0 0 0 0 0 0 0 0 0 0 0 0 0

K01451 0 0 0 0 0 0 0 0 0 0 0 0 0 0 0 0 0 0 0 0 0 0 0 0 0 0 0 0 0 0 0 4 0 0 0 0 0 0 0 0 0 0 0 0 0 0 0 0

K01452 0 0 0 0 0 0 0 0 0 0 0 0 0 0 0 0 0 0 0 0 0 0 0 0 0 0 0 0 0 0 0 0 0 0 0 0 0 0 0 0 0 0 0 0 0 0 0 0

K01453 0 0 0 0 0 0 0 0 0 0 0 0 0 0 0 0 0 0 0 0 0 0 0 0 0 0 0 0 0 0 0 0 0 0 0 0 0 0 0 0 0 0 0 0 0 0 0 0

K01454 0 0 0 0 0 0 0 0 0 0 0 0 0 0 0 0 0 0 0 0 0 0 0 0 0 0 0 0 0 0 0 0 0 0 0 0 0 0 0 0 0 0 0 0 0 0 0 0

K01455 0 0 0 0 0 0 0 0 0 0 0 0 0 0 0 0 0 0 0 0 0 0 0 0 0 0 0 0 0 0 0 0 0 0 0 0 0 0 0 0 0 0 0 0 0 0 0 0

K01457 0 0 0 0 0 0 0 0 0 0 0 0 0 0 0 0 0 0 0 0 0 0 0 0 0 0 0 0 0 0 0 0 0 0 0 0 0 0 0 0 0 0 0 0 0 0 0 0

K01458 0 0 0 0 0 0 0 0 0 0 0 0 0 0 0 0 0 0 0 0 0 0 0 0 0 0 0 0 0 0 0 0 0 0 0 0 0 0 0 0 0 0 0 0 0 0 0 0

K01459 0 0 0 0 0 0 0 0 0 0 0 0 0 0 0 0 0 0 0 0 0 0 0 0 0 0 0 0 0 0 0 0 0 0 0 0 0 0 0 0 0 0 0 0 0 0 0 0

K01460 0 0 0 0 0 0 0 0 0 0 0 0 0 0 0 0 0 0 0 0 0 0 0 0 0 0 0 0 0 0 0 0 0 0 0 0 0 0 0 0 0 0 0 0 0 0 0 0

K01461 0 0 0 0 0 0 0 0 0 0 0 0 0 0 0 0 0 0 0 0 0 0 0 0 0 0 0 0 0 0 0 0 0 0 0 0 0 0 0 0 0 0 0 0 0 0 0 0

K01462 338 420 344 166 406 320 267 564 552 574 661 728 268 601 372 202 508 856 526 476 648 784 838 582 354 338 346 218 292 502 24 648 436 638 772 654 212 438 429 340 452 498 327 580 572 350 470 533

K01463 0 0 0 0 0 0 0 0 0 0 0 0 0 0 0 0 0 0 0 0 0 0 0 0 0 0 0 0 0 0 0 0 0 0 0 0 0 0 0 0 0 0 0 0 0 0 0 0

K01464 0 0 0 0 0 0 0 0 0 0 0 0 0 0 0 0 0 0 0 0 0 0 0 0 0 0 0 0 0 0 0 0 0 0 0 0 0 0 0 0 0 0 0 0 0 0 0 0

K01465 478 724 645 262 978 798 404 1219 768 818 1142 968 386 926 719 320 919 1382 748 822 1088 1127 1335 1064 473 471 472 308 458 1206 24 1038 648 866 1094 934 308 759 916 668 952 984 510 1170 1161 658 788 1134

K01466 0 0 0 0 0 0 0 0 0 0 0 0 0 0 0 0 0 0 0 0 0 0 0 0 0 0 0 0 0 0 0 0 0 0 0 0 0 0 0 0 0 0 0 0 0 0 0 0

K01467 0 0 0 0 1 0 0 0 0 0 0 0 0 0 0 0 0 0 0 0 0 0 0 0 0 0 0 0 0 0 0 0 0 0 0 0 0 0 0 0 0 0 0 0 0 0 0 0

K01468 42 200 159 49 158 147 64 144 106 120 225 154 91 132 160 46 234 210 164 205 234 156 234 208 84 102 94 19 116 243 0 164 68 67 182 137 58 236 295 157 201 244 80 337 382 125 184 244

K01469 0 0 0 0 0 0 0 0 0 0 0 0 0 0 0 0 0 0 0 0 0 0 0 0 0 0 0 0 0 0 0 0 0 0 0 0 0 0 0 0 0 0 0 0 0 0 0 0

K01470 412 684 538 216 695 582 331 872 684 694 947 882 358 854 596 294 818 1160 690 760 982 976 1212 855 462 441 451 300 420 914 24 958 598 778 1011 858 270 704 826 546 784 830 482 1067 1065 514 729 904

K01471 0 0 0 0 0 0 0 0 0 0 0 0 0 0 0 0 0 0 0 0 0 0 0 0 0 0 0 0 0 0 0 0 0 0 0 0 0 0 0 0 0 0 0 0 0 0 0 0

K01473 0 0 0 0 0 0 0 0 0 0 0 0 0 0 0 0 0 0 0 0 0 0 0 0 0 0 0 0 0 0 0 0 0 0 0 0 0 0 0 0 0 0 0 0 0 0 0 0

K01474 0 0 0 0 0 0 0 0 0 0 0 0 0 0 0 0 0 0 0 0 0 0 0 0 0 0 0 0 0 0 0 0 0 0 0 0 0 0 0 0 0 0 0 0 0 0 0 0

K01476 0 0 0 0 0 0 0 0 0 0 0 0 0 0 0 0 0 0 0 0 0 0 0 0 0 0 0 0 0 0 0 0 0 0 0 0 0 0 0 0 0 0 0 0 0 0 0 0

K01477 0 0 0 0 0 0 0 0 0 0 0 0 0 0 0 0 0 0 0 0 0 0 0 0 0 0 0 0 0 0 0 0 0 0 0 0 0 0 0 0 0 0 0 0 0 0 0 0

K01478 0 0 0 0 23 16 0 9 0 0 0 0 0 0 0 0 0 14 0 0 0 0 0 23 0 0 0 0 0 18 0 30 0 0 0 0 0 0 0 0 0 0 0 0 0 0 0 0

K01479 0 0 0 0 24 16 0 9 0 0 0 0 0 0 0 0 0 14 0 0 0 0 0 6 0 0 0 0 0 13 0 0 0 0 0 0 0 0 0 0 0 0 0 0 0 0 0 0

K01480 478 724 645 262 978 798 404 1219 768 818 1142 968 386 926 719 320 919 1382 748 822 1088 1127 1335 1064 473 471 472 308 458 1206 24 1035 648 866 1094 934 308 759 916 668 952 984 510 1170 1161 658 788 1134

K01481 0 0 0 0 0 0 0 0 0 0 0 0 0 0 0 0 0 0 0 0 0 0 0 0 0 0 0 0 0 0 0 0 0 0 0 0 0 0 0 0 0 0 0 0 0 0 0 0

K01482 0 0 0 0 0 0 0 0 0 0 0 0 0 0 0 0 0 0 0 0 0 0 0 0 0 0 0 0 0 0 0 0 0 0 0 0 0 0 0 0 0 0 0 0 0 0 0 0

K01483 0 0 0 0 0 0 0 0 0 0 0 0 0 0 0 0 0 0 0 0 0 0 0 0 0 0 0 0 0 0 0 0 0 0 0 0 0 0 0 0 0 0 0 0 0 0 0 0

K01484 0 0 0 0 0 0 0 0 0 0 0 0 0 0 0 0 0 0 0 0 0 0 0 0 0 0 0 0 0 0 0 0 0 0 0 0 0 0 0 0 0 0 0 0 0 0 0 0

K01485 52 40 107 47 276 216 64 348 84 124 195 86 28 72 108 27 101 222 59 44 106 150 124 195 12 30 21 9 38 292 0 80 50 64 83 77 37 54 90 122 168 145 28 103 96 144 59 230

K01486 99 104 142 47 414 330 72 511 110 124 256 86 28 194 188 74 176 317 59 142 204 186 263 274 35 30 32 71 49 461 0 226 144 161 140 143 37 85 192 171 298 242 103 254 208 184 134 358

K01487 42 200 159 49 158 147 64 144 106 120 225 154 91 132 160 46 234 210 164 205 234 156 234 208 84 102 94 19 116 243 0 164 68 67 182 137 58 236 295 157 201 244 80 337 382 125 184 244

K01488 42 200 159 49 158 147 64 144 106 120 225 154 91 132 160 46 234 210 164 205 234 156 234 208 84 102 94 19 116 243 0 164 68 67 182 137 58 236 295 157 201 244 80 337 382 125 184 244

K01489 42 200 159 49 158 147 64 144 106 120 225 154 91 132 160 46 234 210 164 205 234 156 234 208 84 102 94 19 116 243 0 164 68 67 182 137 58 236 295 157 201 244 80 337 382 125 184 244

K01491 421 820 662 264 770 646 395 870 762 813 1116 1036 450 864 691 292 976 1303 854 886 1118 1098 1306 1044 522 544 533 256 526 1023 24 1036 572 772 1136 928 329 910 1019 654 854 986 486 1254 1334 600 838 1020

K01492 0 0 0 0 0 0 0 0 0 0 0 0 0 0 0 0 0 0 0 0 0 0 0 0 0 0 0 0 0 0 0 0 0 0 0 0 0 0 0 0 0 0 0 0 0 0 0 0

K01493 74 218 232 81 388 317 116 452 146 218 378 199 110 168 238 66 323 390 212 220 300 290 318 344 86 128 107 19 138 465 0 170 96 110 222 164 87 260 360 263 354 349 80 416 453 265 227 416

K01494 478 724 645 262 1002 814 404 1228 768 818 1144 968 386 926 719 320 919 1396 748 822 1088 1127 1335 1086 473 471 472 308 458 1224 24 1068 648 866 1094 934 308 759 916 668 952 984 510 1170 1161 658 788 1134

K01495 0 0 0 0 0 0 0 0 0 0 0 0 0 0 0 0 0 0 0 0 0 0 0 0 0 0 0 0 0 0 0 0 0 0 0 0 0 0 0 0 0 0 0 0 0 0 0 0

K01496 99 104 142 47 414 330 72 511 110 124 256 86 28 194 188 74 176 317 59 142 204 186 263 274 35 30 32 71 49 461 0 226 144 161 140 143 37 85 192 171 298 242 103 254 208 184 134 358

K01497 42 89 54 26 85 98 40 63 40 56 187 50 56 102 63 46 99 116 140 113 140 132 220 174 64 102 83 19 87 112 0 89 15 44 68 104 58 205 170 35 26 87 18 127 198 86 78 188

K01498 0 0 0 0 0 0 0 0 0 0 0 0 0 0 0 0 0 0 0 0 0 0 0 0 0 0 0 0 0 0 0 0 0 0 0 0 0 0 0 0 0 0 0 0 0 0 0 0

K01499 66 40 107 47 306 232 72 356 84 124 197 86 28 72 124 27 101 236 59 62 106 150 124 214 12 30 21 9 38 304 0 84 50 88 83 77 37 54 90 122 168 154 28 103 96 144 59 230

K01500 0 0 0 0 0 0 0 0 0 0 0 0 0 0 0 0 0 0 0 0 0 0 0 0 0 0 0 0 0 0 0 0 0 0 0 0 0 0 0 0 0 0 0 0 0 0 0 0

K01501 0 0 0 0 0 0 0 0 0 0 0 0 0 0 0 0 0 0 0 0 0 0 0 0 0 0 0 0 0 0 0 0 0 0 0 0 0 0 0 0 0 0 0 0 0 0 0 0

K01502 0 0 0 0 0 0 0 0 0 0 0 0 0 0 0 0 0 0 0 0 0 0 0 0 0 0 0 0 0 0 0 0 0 0 0 0 0 0 0 0 0 0 0 0 0 0 0 0

K01503 0 0 0 0 0 0 0 0 0 0 0 0 0 0 0 0 0 0 0 0 0 0 0 0 0 0 0 0 0 0 0 0 0 0 0 0 0 0 0 0 0 0 0 0 0 0 0 0

K01504 0 0 0 0 0 0 0 0 0 0 0 0 0 0 0 0 0 0 0 0 0 0 0 0 0 0 0 0 0 0 0 0 0 0 0 0 0 0 0 0 0 0 0 0 0 0 0 0

K01505 0 0 0 0 0 0 0 0 0 0 0 0 0 0 0 0 0 0 0 0 0 0 0 0 0 0 0 0 0 0 0 0 0 0 0 0 0 0 0 0 0 0 0 0 0 0 0 0

K01506 0 0 0 0 0 0 0 0 0 0 0 0 0 0 0 0 0 0 0 0 0 0 0 0 0 0 0 0 0 0 0 0 0 0 0 0 0 0 0 0 0 0 0 0 0 0 0 0

K01507 520 924 804 312 1160 960 468 1372 873 938 1369 1122 478 1058 878 366 1153 1606 912 1028 1322 1284 1568 1295 558 574 566 328 574 1466 24 1233 716 933 1276 1072 366 994 1210 825 1152 1228 589 1507 1542 783 972 1378

K01509 0 0 0 0 0 0 0 0 0 0 0 0 0 0 0 0 0 0 0 0 0 0 0 0 0 0 0 0 0 0 0 0 0 0 0 0 0 0 0 0 0 0 0 0 0 0 0 0

K01510 0 0 0 0 0 0 0 0 0 0 0 0 0 0 0 0 0 0 0 0 0 0 0 0 0 0 0 0 0 0 0 0 0 0 0 0 0 0 0 0 0 0 0 0 0 0 0 0

K01512 412 684 538 216 720 598 331 880 684 694 950 882 358 854 596 294 818 1174 690 760 982 976 1212 878 462 441 451 300 420 932 24 992 598 778 1011 858 270 704 826 546 784 830 482 1067 1065 514 729 904

K01514 0 0 0 0 1 0 0 0 0 0 0 0 0 0 0 0 0 0 0 0 0 0 0 0 0 0 0 0 0 0 0 0 0 0 0 0 0 0 0 0 0 0 0 0 0 0 0 0

K01515 380 620 503 216 588 484 331 717 657 694 889 882 358 732 532 247 742 1080 690 680 883 941 1072 813 438 441 440 238 409 762 24 842 504 705 954 792 270 674 724 497 653 742 406 916 953 474 654 776

K01516 478 724 645 262 978 798 404 1219 768 818 1142 968 386 926 719 320 919 1382 748 822 1088 1127 1335 1064 473 471 472 308 458 1206 24 1038 648 866 1094 934 308 759 916 668 952 984 510 1170 1161 658 788 1134

K01518 0 0 0 0 0 0 0 0 0 0 0 0 0 0 0 0 0 0 0 0 0 0 0 0 0 0 0 0 0 0 0 0 0 0 0 0 0 0 0 0 0 0 0 0 0 0 0 0

K01519 0 0 0 0 0 0 0 0 0 0 0 0 0 0 0 0 0 0 0 0 0 0 0 0 0 0 0 0 0 0 0 0 0 0 0 0 0 0 0 0 0 0 0 0 0 0 0 0

K01520 404 460 451 214 664 520 340 902 636 698 853 814 296 673 496 228 610 1064 584 538 754 935 962 767 365 368 367 228 330 776 24 698 486 726 856 732 249 493 518 462 620 652 355 682 668 493 530 764

K01521 0 0 0 0 0 0 0 0 0 0 0 0 0 0 0 0 0 0 0 0 0 0 0 0 0 0 0 0 0 0 0 0 0 0 0 0 0 0 0 0 0 0 0 0 0 0 0 0

K01523 66 40 107 47 282 216 72 348 84 124 195 86 28 72 124 27 101 222 59 62 106 150 124 208 12 30 21 9 38 292 0 80 50 88 83 77 37 54 90 122 168 154 28 103 96 144 59 230

K01524 390 460 451 214 682 536 331 911 636 698 858 814 296 673 480 228 610 1078 584 520 754 935 962 776 365 368 367 228 330 794 24 732 486 702 856 732 249 493 518 462 620 644 355 682 668 493 530 764

K01525 0 0 0 0 0 0 0 0 0 0 0 0 0 0 0 0 0 0 0 0 0 0 0 0 0 0 0 0 0 0 0 0 0 0 0 0 0 0 0 0 0 0 0 0 0 0 0 0

K01529 0 0 0 0 0 0 0 0 0 0 0 0 0 0 0 0 0 0 0 0 0 0 0 0 0 0 0 0 0 0 0 0 0 0 0 0 0 0 0 0 0 0 0 0 0 0 0 0

K01531 0 0 0 0 1 0 0 0 0 0 0 0 0 0 0 0 0 0 0 0 0 0 0 0 0 0 0 0 0 0 0 0 0 0 0 0 0 0 0 0 0 0 0 0 0 0 0 0

K01533 492 724 645 262 1007 814 412 1228 768 818 1142 968 386 926 735 320 919 1396 748 841 1088 1127 1335 1100 473 471 472 308 458 1224 24 1068 648 890 1094 934 308 759 916 668 952 992 510 1170 1161 658 788 1134

K01534 471 500 558 260 970 752 412 1258 720 823 1050 900 324 745 619 256 710 1300 644 600 860 1086 1086 998 376 398 388 236 368 1085 24 802 537 814 938 808 286 548 608 584 788 806 383 786 764 636 588 994

K01535 61 222 193 64 213 192 85 184 149 146 264 194 100 166 204 52 246 250 176 234 274 174 273 264 95 107 101 28 134 308 0 210 90 88 224 186 67 266 319 173 216 292 108 362 406 128 200 301

K01537 404 460 451 214 688 536 340 911 636 698 856 814 296 673 496 228 610 1078 584 538 754 935 962 790 365 368 367 228 330 794 24 724 486 726 856 732 249 493 518 462 620 652 355 682 668 493 530 764

K01539 0 0 0 0 0 0 0 0 0 0 0 0 0 0 0 0 0 0 0 0 0 0 0 0 0 0 0 0 0 0 0 4 0 0 0 0 0 0 0 0 0 0 0 0 0 0 0 0

K01541 0 0 0 0 0 0 0 0 0 0 0 0 0 0 0 0 0 0 0 0 0 0 0 0 0 0 0 0 0 0 0 0 0 0 0 0 0 0 0 0 0 0 0 0 0 0 0 0

K01545 0 0 0 0 0 0 0 0 0 0 0 0 0 0 0 0 0 0 0 0 0 0 0 0 0 0 0 0 0 0 0 0 0 0 0 0 0 0 0 0 0 0 0 0 0 0 0 0

K01546 0 0 0 0 1 0 0 0 0 0 0 0 0 0 0 0 0 0 0 0 0 0 0 0 0 0 0 0 0 0 0 0 0 0 0 0 0 0 0 0 0 0 0 0 0 0 0 0

K01547 0 0 0 0 1 0 0 0 0 0 0 0 0 0 0 0 0 0 0 0 0 0 0 0 0 0 0 0 0 0 0 0 0 0 0 0 0 0 0 0 0 0 0 0 0 0 0 0

K01548 0 0 0 0 1 0 0 0 0 0 0 0 0 0 0 0 0 0 0 0 0 0 0 0 0 0 0 0 0 0 0 0 0 0 0 0 0 0 0 0 0 0 0 0 0 0 0 0

K01550 0 0 0 0 0 0 0 0 0 0 0 0 0 0 0 0 0 0 0 0 0 0 0 0 0 0 0 0 0 0 0 0 0 0 0 0 0 0 0 0 0 0 0 0 0 0 0 0

K01551 437 524 486 214 795 634 340 1066 662 698 914 814 296 794 560 275 685 1159 584 618 853 970 1102 832 388 368 378 290 342 946 24 844 580 799 912 798 249 524 620 511 750 740 430 833 780 533 604 891

K01552 34 22 34 15 76 62 21 49 44 26 41 40 10 35 45 6 12 55 12 48 40 18 40 78 10 4 8 9 17 82 0 76 22 46 42 50 8 30 24 16 16 48 28 24 24 4 16 58

K01553 0 0 0 0 0 0 0 0 0 0 0 0 0 0 0 0 0 0 0 0 0 0 0 0 0 0 0 0 0 0 0 0 0 0 0 0 0 0 0 0 0 0 0 0 0 0 0 0

K01554 0 0 0 0 0 0 0 0 0 0 0 0 0 0 0 0 0 0 0 0 0 0 0 0 0 0 0 0 0 0 0 0 0 0 0 0 0 0 0 0 0 0 0 0 0 0 0 0

K01555 0 0 0 0 0 0 0 0 0 0 0 0 0 0 0 0 0 0 0 0 0 0 0 0 0 0 0 0 0 0 0 0 0 0 0 0 0 0 0 0 0 0 0 0 0 0 0 0

K01556 0 0 0 0 0 0 0 0 0 0 0 0 0 0 0 0 0 0 0 0 0 0 0 0 0 0 0 0 0 0 0 0 0 0 0 0 0 0 0 0 0 0 0 0 0 0 0 0

K01557 0 0 0 0 0 0 0 0 0 0 0 0 0 0 0 0 0 0 0 0 0 0 0 0 0 0 0 0 0 0 0 0 0 0 0 0 0 0 0 0 0 0 0 0 0 0 0 0

K01560 42 200 159 49 158 147 64 144 106 120 225 154 91 132 160 46 234 210 164 205 234 156 234 208 84 102 94 19 116 243 0 164 68 67 182 137 58 236 295 157 201 244 80 337 382 125 184 244

K01561 0 0 0 0 0 0 0 0 0 0 0 0 0 0 0 0 0 0 0 0 0 0 0 0 0 0 0 0 0 0 0 0 0 0 0 0 0 0 0 0 0 0 0 0 0 0 0 0

K01563 0 0 0 0 0 0 0 0 0 0 0 0 0 0 0 0 0 0 0 0 0 0 0 0 0 0 0 0 0 0 0 0 0 0 0 0 0 0 0 0 0 0 0 0 0 0 0 0

K01564 0 0 0 0 0 0 0 0 0 0 0 0 0 0 0 0 0 0 0 0 0 0 0 0 0 0 0 0 0 0 0 0 0 0 0 0 0 0 0 0 0 0 0 0 0 0 0 0

K01565 0 0 0 0 0 0 0 0 0 0 0 0 0 0 0 0 0 0 0 0 0 0 0 0 0 0 0 0 0 0 0 0 0 0 0 0 0 0 0 0 0 0 0 0 0 0 0 0

K01567 42 311 264 72 232 196 88 226 170 184 263 258 126 162 256 46 369 303 188 297 328 181 247 243 106 102 104 19 146 374 0 240 121 90 296 170 58 266 420 279 376 401 141 547 564 164 290 298

K01568 0 0 0 0 0 0 0 0 0 0 0 0 0 0 0 0 0 0 0 0 0 0 0 0 0 0 0 0 0 0 0 0 0 0 0 0 0 0 0 0 0 0 0 0 0 0 0 0

K01569 0 0 0 0 0 0 0 0 0 0 0 0 0 0 0 0 0 0 0 0 0 0 0 0 0 0 0 0 0 0 0 0 0 0 0 0 0 0 0 0 0 0 0 0 0 0 0 0

K01571 0 0 0 0 0 0 0 0 0 0 0 0 0 0 0 0 0 0 0 0 0 0 0 0 0 0 0 0 0 0 0 0 0 0 0 0 0 0 0 0 0 0 0 0 0 0 0 0

K01572 42 200 159 49 158 147 64 144 106 120 225 154 91 132 160 46 234 210 164 205 234 156 234 208 84 102 94 19 116 243 0 164 68 67 182 137 58 236 295 157 201 244 80 337 382 125 184 244

K01573 0 0 0 0 0 0 0 0 0 0 0 0 0 0 0 0 0 0 0 0 0 0 0 0 0 0 0 0 0 0 0 0 0 0 0 0 0 0 0 0 0 0 0 0 0 0 0 0

K01574 0 0 0 0 0 0 0 0 0 0 0 0 0 0 0 0 0 0 0 0 0 0 0 0 0 0 0 0 0 0 0 0 0 0 0 0 0 0 0 0 0 0 0 0 0 0 0 0

K01575 0 0 0 0 24 16 0 9 0 0 3 0 0 0 0 0 0 14 0 0 0 0 0 23 0 0 0 0 0 18 0 30 0 0 0 0 0 0 0 0 0 0 0 0 0 0 0 0

K01576 0 0 0 0 0 0 0 0 0 0 0 0 0 0 0 0 0 0 0 0 0 0 0 0 0 0 0 0 0 0 0 0 0 0 0 0 0 0 0 0 0 0 0 0 0 0 0 0

K01577 0 0 0 0 0 0 0 0 0 0 0 0 0 0 0 0 0 0 0 0 0 0 0 0 0 0 0 0 0 0 0 0 0 0 0 0 0 0 0 0 0 0 0 0 0 0 0 0

K01578 0 0 0 0 0 0 0 0 0 0 0 0 0 0 0 0 0 0 0 0 0 0 0 0 0 0 0 0 0 0 0 0 0 0 0 0 0 0 0 0 0 0 0 0 0 0 0 0

K01579 0 0 0 0 0 0 0 0 0 0 0 0 0 0 0 0 0 0 0 0 0 0 0 0 0 0 0 0 0 0 0 0 0 0 0 0 0 0 0 0 0 0 0 0 0 0 0 0

K01580 0 0 0 0 0 0 0 0 0 0 0 0 0 0 0 0 0 0 0 0 0 0 0 0 0 0 0 0 0 0 0 0 0 0 0 0 0 0 0 0 0 0 0 0 0 0 0 0

K01581 0 0 0 0 1 0 0 0 0 0 0 0 0 0 0 0 0 0 0 0 0 0 0 0 0 0 0 0 0 0 0 0 0 0 0 0 0 0 0 0 0 0 0 0 0 0 0 0

K01582 42 200 159 49 182 163 64 154 106 120 225 154 91 132 160 46 234 224 164 205 234 156 234 232 84 102 94 19 116 260 0 194 68 67 182 137 58 236 295 157 201 244 80 337 382 125 184 244

K01583 0 0 0 0 0 0 0 0 0 0 0 0 0 0 0 0 0 0 0 0 0 0 0 0 0 0 0 0 0 0 0 0 0 0 0 0 0 0 0 0 0 0 0 0 0 0 0 0

K01584 0 0 0 0 1 0 0 0 0 0 0 0 0 0 0 0 0 0 0 0 0 0 0 0 0 0 0 0 0 0 0 0 0 0 0 0 0 0 0 0 0 0 0 0 0 0 0 0

K01585 0 0 0 0 0 0 0 0 0 0 0 0 0 0 0 0 0 0 0 0 0 0 0 0 0 0 0 0 0 0 0 0 0 0 0 0 0 0 0 0 0 0 0 0 0 0 0 0

K01586 437 524 486 214 819 650 340 1074 662 698 917 814 296 794 560 275 685 1173 584 618 853 970 1102 855 388 368 378 290 342 963 24 874 580 799 912 798 249 524 620 511 750 740 430 833 780 533 604 891

K01588 478 724 645 262 978 798 404 1219 768 818 1142 968 386 926 719 320 919 1382 748 822 1088 1127 1335 1064 473 471 472 308 458 1206 24 1038 648 866 1094 934 308 759 916 668 952 984 510 1170 1161 658 788 1134

K01589 0 0 0 0 0 0 0 0 0 0 0 0 0 0 0 0 0 0 0 0 0 0 0 0 0 0 0 0 0 0 0 0 0 0 0 0 0 0 0 0 0 0 0 0 0 0 0 0

K01590 0 0 0 0 0 0 0 0 0 0 0 0 0 0 0 0 0 0 0 0 0 0 0 0 0 0 0 0 0 0 0 0 0 0 0 0 0 0 0 0 0 0 0 0 0 0 0 0

K01591 478 724 645 262 978 798 404 1219 768 818 1142 968 386 926 719 320 919 1382 748 822 1088 1127 1335 1064 473 471 472 308 458 1206 24 1038 648 866 1094 934 308 759 916 668 952 984 510 1170 1161 658 788 1134

K01592 140 304 301 96 572 477 136 656 216 244 481 240 119 325 347 119 410 526 223 347 439 342 496 482 120 132 126 90 166 704 0 391 212 228 322 280 96 320 486 328 500 486 182 590 590 308 318 602

K01593 0 0 0 0 0 0 0 0 0 0 0 0 0 0 0 0 0 0 0 0 0 0 0 0 0 0 0 0 0 0 0 0 0 0 0 0 0 0 0 0 0 0 0 0 0 0 0 0

K01594 0 0 0 0 0 0 0 0 0 0 0 0 0 0 0 0 0 0 0 0 0 0 0 0 0 0 0 0 0 0 0 0 0 0 0 0 0 0 0 0 0 0 0 0 0 0 0 0

K01595 14 0 0 0 6 0 8 0 0 0 3 0 0 0 16 0 0 0 0 18 0 0 0 14 0 0 0 0 0 0 0 4 0 24 0 0 0 0 0 0 0 8 0 0 0 0 0 0

K01596 42 200 159 49 158 147 64 144 106 120 225 154 91 132 160 46 234 210 164 205 234 156 234 208 84 102 94 19 116 243 0 164 68 67 182 137 58 236 295 157 201 244 80 337 382 125 184 244

K01597 0 0 0 0 0 0 0 0 0 0 0 0 0 0 0 0 0 0 0 0 0 0 0 0 0 0 0 0 0 0 0 0 0 0 0 0 0 0 0 0 0 0 0 0 0 0 0 0

K01598 0 0 0 0 0 0 0 0 0 0 0 0 0 0 0 0 0 0 0 0 0 0 0 0 0 0 0 0 0 0 0 0 0 0 0 0 0 0 0 0 0 0 0 0 0 0 0 0

K01599 338 420 344 166 406 320 267 564 552 574 661 728 268 601 372 202 508 856 526 476 648 784 838 582 354 338 346 218 292 502 24 648 436 638 772 654 212 438 429 340 452 498 327 580 572 350 470 533

K01601 42 200 159 49 182 163 64 154 106 120 228 154 91 132 160 46 234 224 164 205 234 156 234 232 84 102 94 19 116 260 0 194 68 67 182 137 58 236 295 157 201 244 80 337 382 125 184 244

K01602 0 0 0 0 0 0 0 0 0 0 0 0 0 0 0 0 0 0 0 0 0 0 0 0 0 0 0 0 0 0 0 0 0 0 0 0 0 0 0 0 0 0 0 0 0 0 0 0

K01604 0 0 0 0 0 0 0 0 0 0 0 0 0 0 0 0 0 0 0 0 0 0 0 0 0 0 0 0 0 0 0 0 0 0 0 0 0 0 0 0 0 0 0 0 0 0 0 0

K01605 0 0 0 0 0 0 0 0 0 0 0 0 0 0 0 0 0 0 0 0 0 0 0 0 0 0 0 0 0 0 0 0 0 0 0 0 0 0 0 0 0 0 0 0 0 0 0 0

K01606 0 0 0 0 0 0 0 0 0 0 0 0 0 0 0 0 0 0 0 0 0 0 0 0 0 0 0 0 0 0 0 0 0 0 0 0 0 0 0 0 0 0 0 0 0 0 0 0

K01607 34 22 34 15 54 46 21 40 44 26 38 40 10 35 45 6 12 41 12 48 40 18 40 72 10 4 8 9 17 70 0 72 22 46 42 50 8 30 24 16 16 48 28 24 24 4 16 58

K01608 0 0 0 0 0 0 0 0 0 0 0 0 0 0 0 0 0 0 0 0 0 0 0 0 0 0 0 0 0 0 0 0 0 0 0 0 0 0 0 0 0 0 0 0 0 0 0 0

K01609 437 524 486 214 819 650 340 1074 662 698 917 814 296 794 560 275 685 1173 584 618 853 970 1102 855 388 368 378 290 342 963 24 874 580 799 912 798 249 524 620 511 750 740 430 833 780 533 604 891

K01610 0 0 0 0 0 0 0 0 0 0 0 0 0 0 0 0 0 0 0 0 0 0 0 0 0 0 0 0 0 0 0 0 0 0 0 0 0 0 0 0 0 0 0 0 0 0 0 0

K01611 412 684 538 216 695 582 331 872 684 694 947 882 358 854 596 294 818 1160 690 760 982 976 1212 855 462 441 451 300 420 914 24 958 598 778 1011 858 270 704 826 546 784 830 482 1067 1065 514 729 904

K01612 0 0 0 0 0 0 0 0 0 0 0 0 0 0 0 0 0 0 0 0 0 0 0 0 0 0 0 0 0 0 0 0 0 0 0 0 0 0 0 0 0 0 0 0 0 0 0 0

K01613 352 420 344 166 412 320 276 564 552 574 661 728 268 601 388 202 508 856 526 494 648 784 838 595 354 338 346 218 292 502 24 648 436 662 772 654 212 438 429 340 452 507 327 580 572 350 470 533

K01615 0 0 0 0 0 0 0 0 0 0 0 0 0 0 0 0 0 0 0 0 0 0 0 0 0 0 0 0 0 0 0 0 0 0 0 0 0 0 0 0 0 0 0 0 0 0 0 0

K01616 0 0 0 0 0 0 0 0 0 0 0 0 0 0 0 0 0 0 0 0 0 0 0 0 0 0 0 0 0 0 0 0 0 0 0 0 0 0 0 0 0 0 0 0 0 0 0 0

K01617 0 0 0 0 0 0 0 0 0 0 0 0 0 0 0 0 0 0 0 0 0 0 0 0 0 0 0 0 0 0 0 0 0 0 0 0 0 0 0 0 0 0 0 0 0 0 0 0

K01618 0 0 0 0 0 0 0 0 0 0 0 0 0 0 0 0 0 0 0 0 0 0 0 0 0 0 0 0 0 0 0 0 0 0 0 0 0 0 0 0 0 0 0 0 0 0 0 0

K01619 446 660 610 262 822 667 404 1046 741 818 1078 968 386 804 655 274 844 1274 748 743 988 1092 1196 976 450 471 460 246 447 1019 24 862 554 793 1038 868 308 728 814 619 821 896 434 1020 1049 618 714 1007

K01620 0 0 0 0 0 0 0 0 0 0 0 0 0 0 0 0 0 0 0 0 0 0 0 0 0 0 0 0 0 0 0 0 0 0 0 0 0 0 0 0 0 0 0 0 0 0 0 0

K01621 0 0 0 0 0 0 0 0 0 0 0 0 0 0 0 0 0 0 0 0 0 0 0 0 0 0 0 0 0 0 0 0 0 0 0 0 0 0 0 0 0 0 0 0 0 0 0 0

K01622 586 964 911 358 1394 1144 540 1702 957 1062 1559 1208 506 1130 1002 393 1254 1800 972 1090 1428 1434 1692 1474 569 604 586 336 612 1728 24 1284 767 1021 1359 1148 403 1049 1300 947 1320 1382 617 1610 1638 926 1031 1608

K01623 437 524 486 214 866 682 340 1092 662 698 922 814 296 794 560 275 685 1201 584 618 853 970 1102 901 388 368 378 290 342 998 24 934 580 799 912 798 249 524 620 511 750 740 430 833 780 533 604 891

K01624 338 420 344 166 406 320 267 564 552 574 661 728 268 601 372 202 508 856 526 476 648 784 838 582 354 338 346 218 292 502 24 648 436 638 772 654 212 438 429 340 452 498 327 580 572 350 470 533

K01625 338 420 344 166 406 320 267 564 552 574 661 728 268 601 372 202 508 856 526 476 648 784 838 582 354 338 346 218 292 502 24 648 436 638 772 654 212 438 429 340 452 498 327 580 572 350 470 533

K01626 0 0 0 0 0 0 0 0 0 0 0 0 0 0 0 0 0 0 0 0 0 0 0 0 0 0 0 0 0 0 0 0 0 0 0 0 0 0 0 0 0 0 0 0 0 0 0 0

K01627 0 0 0 0 0 0 0 0 0 0 0 0 0 0 0 0 0 0 0 0 0 0 0 0 0 0 0 0 0 0 0 0 0 0 0 0 0 0 0 0 0 0 0 0 0 0 0 0

K01628 478 724 645 262 978 798 404 1219 768 818 1142 968 386 926 719 320 919 1382 748 822 1088 1127 1335 1064 473 471 472 308 458 1206 24 1038 648 866 1094 934 308 759 916 668 952 984 510 1170 1161 658 788 1134

K01629 0 0 0 0 0 0 0 0 0 0 0 0 0 0 0 0 0 0 0 0 0 0 0 0 0 0 0 0 0 0 0 0 0 0 0 0 0 0 0 0 0 0 0 0 0 0 0 0

K01630 0 0 0 0 0 0 0 0 0 0 0 0 0 0 0 0 0 0 0 0 0 0 0 0 0 0 0 0 0 0 0 0 0 0 0 0 0 0 0 0 0 0 0 0 0 0 0 0

K01631 0 0 0 0 0 0 0 0 0 0 0 0 0 0 0 0 0 0 0 0 0 0 0 0 0 0 0 0 0 0 0 0 0 0 0 0 0 0 0 0 0 0 0 0 0 0 0 0

K01632 0 0 0 0 0 0 0 0 0 0 0 0 0 0 0 0 0 0 0 0 0 0 0 0 0 0 0 0 0 0 0 0 0 0 0 0 0 0 0 0 0 0 0 0 0 0 0 0

K01633 0 0 0 0 0 0 0 0 0 0 0 0 0 0 0 0 0 0 0 0 0 0 0 0 0 0 0 0 0 0 0 0 0 0 0 0 0 0 0 0 0 0 0 0 0 0 0 0

K01634 0 0 0 0 0 0 0 0 0 0 0 0 0 0 0 0 0 0 0 0 0 0 0 0 0 0 0 0 0 0 0 0 0 0 0 0 0 0 0 0 0 0 0 0 0 0 0 0

K01635 0 0 0 0 0 0 0 0 0 0 0 0 0 0 0 0 0 0 0 0 0 0 0 0 0 0 0 0 0 0 0 0 0 0 0 0 0 0 0 0 0 0 0 0 0 0 0 0

K01636 0 0 0 0 0 0 0 0 0 0 0 0 0 0 0 0 0 0 0 0 0 0 0 0 0 0 0 0 0 0 0 0 0 0 0 0 0 0 0 0 0 0 0 0 0 0 0 0

K01637 0 0 0 0 0 0 0 0 0 0 0 0 0 0 0 0 0 0 0 0 0 0 0 0 0 0 0 0 0 0 0 0 0 0 0 0 0 0 0 0 0 0 0 0 0 0 0 0

K01638 0 0 0 0 0 0 0 0 0 0 0 0 0 0 0 0 0 0 0 0 0 0 0 0 0 0 0 0 0 0 0 0 0 0 0 0 0 0 0 0 0 0 0 0 0 0 0 0

K01639 0 0 0 0 0 0 0 0 0 0 0 0 0 0 0 0 0 0 0 0 0 0 0 0 0 0 0 0 0 0 0 0 0 0 0 0 0 0 0 0 0 0 0 0 0 0 0 0

K01640 0 0 0 0 0 0 0 0 0 0 0 0 0 0 0 0 0 0 0 0 0 0 0 0 0 0 0 0 0 0 0 0 0 0 0 0 0 0 0 0 0 0 0 0 0 0 0 0

K01641 0 0 0 0 0 0 0 0 0 0 0 0 0 0 0 0 0 0 0 0 0 0 0 0 0 0 0 0 0 0 0 0 0 0 0 0 0 0 0 0 0 0 0 0 0 0 0 0

K01643 42 311 264 72 232 196 88 226 170 184 263 258 126 162 256 46 369 303 188 297 328 181 247 243 106 102 104 19 146 374 0 240 121 90 296 170 58 266 420 279 376 401 141 547 564 164 290 298

K01644 380 730 608 239 638 516 356 790 722 758 924 986 394 762 628 247 878 1159 713 772 977 966 1086 824 459 441 450 238 438 876 24 888 557 728 1068 825 270 704 850 619 828 900 468 1126 1136 514 760 832

K01646 42 200 159 49 158 147 64 144 106 120 225 154 91 132 160 46 234 210 164 205 234 156 234 208 84 102 94 19 116 243 0 164 68 67 182 137 58 236 295 157 201 244 80 337 382 125 184 244

K01647 492 724 645 262 960 782 412 1210 768 818 1139 968 386 926 735 320 919 1368 748 841 1088 1127 1335 1054 473 471 472 308 458 1188 24 1008 648 890 1094 934 308 759 916 668 952 992 510 1170 1161 658 788 1134

K01648 0 0 0 0 0 0 0 0 0 0 0 0 0 0 0 0 0 0 0 0 0 0 0 0 0 0 0 0 0 0 0 0 0 0 0 0 0 0 0 0 0 0 0 0 0 0 0 0

K01649 437 524 486 214 843 666 340 1084 662 698 919 814 296 794 560 275 685 1187 584 618 853 970 1102 878 388 368 378 290 342 980 24 904 580 799 912 798 249 524 620 511 750 740 430 833 780 533 604 891

K01652 518 564 593 260 1084 850 420 1413 746 823 1109 900 324 866 699 302 786 1381 644 698 958 1121 1225 1054 400 398 399 298 380 1237 24 924 631 912 995 874 286 578 710 633 918 902 458 936 876 676 663 1122

K01653 404 460 451 214 688 536 340 911 636 698 856 814 296 673 496 228 610 1078 584 538 754 935 962 790 365 368 367 228 330 794 24 728 486 726 856 732 249 493 518 462 620 652 355 682 668 493 530 764

K01654 52 40 107 47 252 200 64 338 84 124 192 86 28 72 108 27 101 208 59 44 106 150 124 172 12 30 21 9 38 274 0 50 50 64 83 77 37 54 90 122 168 145 28 103 96 144 59 230

K01655 0 0 0 0 0 0 0 0 0 0 0 0 0 0 0 0 0 0 0 0 0 0 0 0 0 0 0 0 0 0 0 0 0 0 0 0 0 0 0 0 0 0 0 0 0 0 0 0

K01657 437 524 486 214 819 650 340 1074 662 698 917 814 296 794 560 275 685 1173 584 618 853 970 1102 855 388 368 378 290 342 963 24 874 580 799 912 798 249 524 620 511 750 740 430 833 780 533 604 891

K01658 437 524 486 214 819 650 340 1074 662 698 917 814 296 794 560 275 685 1173 584 618 853 970 1102 855 388 368 378 290 342 963 24 874 580 799 912 798 249 524 620 511 750 740 430 833 780 533 604 891

K01659 0 0 0 0 0 0 0 0 0 0 0 0 0 0 0 0 0 0 0 0 0 0 0 0 0 0 0 0 0 0 0 0 0 0 0 0 0 0 0 0 0 0 0 0 0 0 0 0

K01660 0 0 0 0 0 0 0 0 0 0 0 0 0 0 0 0 0 0 0 0 0 0 0 0 0 0 0 0 0 0 0 0 0 0 0 0 0 0 0 0 0 0 0 0 0 0 0 0

K01661 0 0 0 0 0 0 0 0 0 0 0 0 0 0 0 0 0 0 0 0 0 0 0 0 0 0 0 0 0 0 0 0 0 0 0 0 0 0 0 0 0 0 0 0 0 0 0 0

K01662 338 420 344 166 406 320 267 564 552 574 661 728 268 601 372 202 508 856 526 476 648 784 838 582 354 338 346 218 292 502 24 648 436 638 772 654 212 438 429 340 452 498 327 580 572 350 470 533

K01663 0 0 0 0 0 0 0 0 0 0 0 0 0 0 0 0 0 0 0 0 0 0 0 0 0 0 0 0 0 0 0 0 0 0 0 0 0 0 0 0 0 0 0 0 0 0 0 0

K01664 0 0 0 0 0 0 0 0 0 0 0 0 0 0 0 0 0 0 0 0 0 0 0 0 0 0 0 0 0 0 0 0 0 0 0 0 0 0 0 0 0 0 0 0 0 0 0 0

K01665 0 0 0 0 0 0 0 0 0 0 0 0 0 0 0 0 0 0 0 0 0 0 0 0 0 0 0 0 0 0 0 0 0 0 0 0 0 0 0 0 0 0 0 0 0 0 0 0

K01666 0 0 0 0 0 0 0 0 0 0 0 0 0 0 0 0 0 0 0 0 0 0 0 0 0 0 0 0 0 0 0 0 0 0 0 0 0 0 0 0 0 0 0 0 0 0 0 0

K01667 0 0 0 0 0 0 0 0 0 0 0 0 0 0 0 0 0 0 0 0 0 0 0 0 0 0 0 0 0 0 0 0 0 0 0 0 0 0 0 0 0 0 0 0 0 0 0 0

K01668 0 0 0 0 0 0 0 0 0 0 0 0 0 0 0 0 0 0 0 0 0 0 0 0 0 0 0 0 0 0 0 0 0 0 0 0 0 0 0 0 0 0 0 0 0 0 0 0

K01669 352 420 344 166 412 320 276 564 552 574 664 728 268 601 388 202 508 856 526 494 648 784 838 595 354 338 346 218 292 502 24 651 436 662 772 654 212 438 429 340 452 507 327 580 572 350 470 533

K01671 0 0 0 0 0 0 0 0 0 0 0 0 0 0 0 0 0 0 0 0 0 0 0 0 0 0 0 0 0 0 0 0 0 0 0 0 0 0 0 0 0 0 0 0 0 0 0 0

K01672 0 0 0 0 0 0 0 0 0 0 0 0 0 0 0 0 0 0 0 0 0 0 0 0 0 0 0 0 0 0 0 0 0 0 0 0 0 0 0 0 0 0 0 0 0 0 0 0

K01673 404 460 451 214 688 536 340 911 636 698 856 814 296 673 496 228 610 1078 584 538 754 935 962 790 365 368 367 228 330 794 24 728 486 726 856 732 249 493 518 462 620 652 355 682 668 493 530 764

K01674 0 0 0 0 0 0 0 0 0 0 0 0 0 0 0 0 0 0 0 0 0 0 0 0 0 0 0 0 0 0 0 0 0 0 0 0 0 0 0 0 0 0 0 0 0 0 0 0

K01676 0 0 0 0 0 0 0 0 0 0 0 0 0 0 0 0 0 0 0 0 0 0 0 0 0 0 0 0 0 0 0 0 0 0 0 0 0 0 0 0 0 0 0 0 0 0 0 0

K01677 545 764 752 310 1236 997 476 1558 852 942 1334 1054 414 998 842 348 1020 1590 808 885 1193 1278 1458 1249 484 501 493 318 496 1480 24 1089 699 954 1177 1012 344 814 1005 790 1120 1138 538 1273 1257 802 847 1365

K01678 545 764 752 310 1236 997 476 1558 852 942 1334 1054 414 998 842 348 1020 1590 808 885 1193 1278 1458 1249 484 501 493 318 496 1480 24 1089 699 954 1177 1012 344 814 1005 790 1120 1138 538 1273 1257 802 847 1365

K01679 0 0 0 0 0 0 0 0 0 0 0 0 0 0 0 0 0 0 0 0 0 0 0 0 0 0 0 0 0 0 0 0 0 0 0 0 0 0 0 0 0 0 0 0 0 0 0 0

K01681 338 420 344 166 406 320 267 564 552 574 661 728 268 601 372 202 508 856 526 476 648 784 838 582 354 338 346 218 292 502 24 648 436 638 772 654 212 438 429 340 452 498 327 580 572 350 470 533

K01682 0 0 0 0 0 0 0 0 0 0 0 0 0 0 0 0 0 0 0 0 0 0 0 0 0 0 0 0 0 0 0 0 0 0 0 0 0 0 0 0 0 0 0 0 0 0 0 0

K01684 0 0 0 0 0 0 0 0 0 0 0 0 0 0 0 0 0 0 0 0 0 0 0 0 0 0 0 0 0 0 0 0 0 0 0 0 0 0 0 0 0 0 0 0 0 0 0 0

K01685 0 0 0 0 0 0 0 0 0 0 0 0 0 0 0 0 0 0 0 0 0 0 0 0 0 0 0 0 0 0 0 0 0 0 0 0 0 0 0 0 0 0 0 0 0 0 0 0

K01686 0 0 0 0 0 0 0 0 0 0 0 0 0 0 0 0 0 0 0 0 0 0 0 0 0 0 0 0 0 0 0 0 0 0 0 0 0 0 0 0 0 0 0 0 0 0 0 0

K01687 437 524 486 214 819 650 340 1074 662 698 917 814 296 794 560 275 685 1173 584 618 853 970 1102 855 388 368 378 290 342 963 24 874 580 799 912 798 249 524 620 511 750 740 430 833 780 533 604 891

K01689 478 724 645 262 1000 814 404 1228 768 818 1144 968 386 926 719 320 919 1396 748 822 1088 1127 1335 1070 473 471 472 308 458 1219 24 1038 648 866 1094 934 308 759 916 668 952 984 510 1170 1161 658 788 1134

K01690 0 0 0 0 0 0 0 0 0 0 0 0 0 0 0 0 0 0 0 0 0 0 0 0 0 0 0 0 0 0 0 0 0 0 0 0 0 0 0 0 0 0 0 0 0 0 0 0

K01692 380 620 503 216 564 468 331 708 657 694 886 882 358 732 532 247 742 1066 690 680 883 941 1072 790 438 441 440 238 409 745 24 812 504 705 954 792 270 674 724 497 653 742 406 916 953 474 654 776

K01693 437 524 486 214 819 650 340 1074 662 698 917 814 296 794 560 275 685 1173 584 618 853 970 1102 855 388 368 378 290 342 963 24 874 580 799 912 798 249 524 620 511 750 740 430 833 780 533 604 891

K01695 437 524 486 214 819 650 340 1074 662 698 917 814 296 794 560 275 685 1173 584 618 853 970 1102 855 388 368 378 290 342 963 24 874 580 799 912 798 249 524 620 511 750 740 430 833 780 533 604 891

K01696 437 524 486 214 819 650 340 1074 662 698 917 814 296 794 560 275 685 1173 584 618 853 970 1102 855 388 368 378 290 342 963 24 874 580 799 912 798 249 524 620 511 750 740 430 833 780 533 604 891

K01697 0 0 0 0 1 0 0 0 0 0 0 0 0 0 0 0 0 0 0 0 0 0 0 0 0 0 0 0 0 0 0 0 0 0 0 0 0 0 0 0 0 0 0 0 0 0 0 0

K01698 437 524 486 214 819 650 340 1074 662 698 917 814 296 794 560 275 685 1173 584 618 853 970 1102 855 388 368 378 290 342 963 24 874 580 799 912 798 249 524 620 511 750 740 430 833 780 533 604 891

K01699 0 0 0 0 0 0 0 0 0 0 0 0 0 0 0 0 0 0 0 0 0 0 0 0 0 0 0 0 0 0 0 0 0 0 0 0 0 0 0 0 0 0 0 0 0 0 0 0

K01702 0 0 0 0 0 0 0 0 0 0 0 0 0 0 0 0 0 0 0 0 0 0 0 0 0 0 0 0 0 0 0 0 0 0 0 0 0 0 0 0 0 0 0 0 0 0 0 0

K01703 1214 2128 1981 766 2996 2467 1144 3566 2020 2244 3348 2569 1102 2390 2164 832 2742 3838 2107 2385 3090 3024 3618 3170 1222 1310 1266 692 1342 3724 48 2732 1602 2109 2900 2434 864 2334 2895 2051 2842 3007 1314 3557 3658 1978 2246 3460

K01704 1173 2039 1926 740 2910 2369 1104 3504 1979 2188 3161 2519 1046 2289 2100 786 2643 3722 1966 2272 2949 2892 3398 2996 1159 1207 1183 673 1254 3612 48 2642 1587 2065 2832 2330 806 2128 2726 2016 2816 2920 1296 3430 3460 1892 2168 3272

K01705 0 0 0 0 0 0 0 0 0 0 0 0 0 0 0 0 0 0 0 0 0 0 0 0 0 0 0 0 0 0 0 0 0 0 0 0 0 0 0 0 0 0 0 0 0 0 0 0

K01706 0 0 0 0 0 0 0 0 0 0 0 0 0 0 0 0 0 0 0 0 0 0 0 0 0 0 0 0 0 0 0 0 0 0 0 0 0 0 0 0 0 0 0 0 0 0 0 0

K01707 0 0 0 0 0 0 0 0 0 0 0 0 0 0 0 0 0 0 0 0 0 0 0 0 0 0 0 0 0 0 0 0 0 0 0 0 0 0 0 0 0 0 0 0 0 0 0 0

K01708 0 0 0 0 0 0 0 0 0 0 0 0 0 0 0 0 0 0 0 0 0 0 0 0 0 0 0 0 0 0 0 0 0 0 0 0 0 0 0 0 0 0 0 0 0 0 0 0

K01709 0 0 0 0 0 0 0 0 0 0 0 0 0 0 0 0 0 0 0 0 0 0 0 0 0 0 0 0 0 0 0 0 0 0 0 0 0 0 0 0 0 0 0 0 0 0 0 0

K01710 490 564 593 260 1078 850 412 1413 746 823 1111 900 324 866 683 302 786 1381 644 662 958 1121 1225 1040 400 398 399 298 380 1237 24 928 631 862 995 874 286 578 710 633 918 894 458 936 876 676 663 1122

K01711 358 442 378 182 460 366 288 604 595 600 702 769 277 636 417 208 520 897 537 505 688 802 878 654 364 343 354 228 310 572 24 720 458 659 815 704 220 468 453 356 468 547 355 604 596 353 486 590

K01712 42 200 159 49 158 147 64 144 106 120 225 154 91 132 160 46 234 210 164 205 234 156 234 208 84 102 94 19 116 243 0 164 68 67 182 137 58 236 295 157 201 244 80 337 382 125 184 244

K01713 0 0 0 0 23 16 0 9 0 0 0 0 0 0 0 0 0 14 0 0 0 0 0 6 0 0 0 0 0 13 0 4 0 0 0 0 0 0 0 0 0 0 0 0 0 0 0 0

K01714 437 524 486 214 819 650 340 1074 662 698 917 814 296 794 560 275 685 1173 584 618 853 970 1102 855 388 368 378 290 342 963 24 874 580 799 912 798 249 524 620 511 750 740 430 833 780 533 604 891

K01715 0 0 0 0 0 0 0 0 0 0 0 0 0 0 0 0 0 0 0 0 0 0 0 0 0 0 0 0 0 0 0 0 0 0 0 0 0 0 0 0 0 0 0 0 0 0 0 0

K01716 0 0 0 0 0 0 0 0 0 0 0 0 0 0 0 0 0 0 0 0 0 0 0 0 0 0 0 0 0 0 0 0 0 0 0 0 0 0 0 0 0 0 0 0 0 0 0 0

K01718 0 0 0 0 0 0 0 0 0 0 0 0 0 0 0 0 0 0 0 0 0 0 0 0 0 0 0 0 0 0 0 0 0 0 0 0 0 0 0 0 0 0 0 0 0 0 0 0

K01719 404 460 451 214 688 536 340 911 636 698 856 814 296 673 496 228 610 1078 584 538 754 935 962 790 365 368 367 228 330 794 24 724 486 726 856 732 249 493 518 462 620 652 355 682 668 493 530 764

K01720 0 0 0 0 0 0 0 0 0 0 0 0 0 0 0 0 0 0 0 0 0 0 0 0 0 0 0 0 0 0 0 0 0 0 0 0 0 0 0 0 0 0 0 0 0 0 0 0

K01721 0 0 0 0 0 0 0 0 0 0 0 0 0 0 0 0 0 0 0 0 0 0 0 0 0 0 0 0 0 0 0 0 0 0 0 0 0 0 0 0 0 0 0 0 0 0 0 0

K01722 0 0 0 0 0 0 0 0 0 0 0 0 0 0 0 0 0 0 0 0 0 0 0 0 0 0 0 0 0 0 0 0 0 0 0 0 0 0 0 0 0 0 0 0 0 0 0 0

K01724 0 0 0 0 24 16 0 9 0 0 0 0 0 0 0 0 0 14 0 0 0 0 0 23 0 0 0 0 0 18 0 30 0 0 0 0 0 0 0 0 0 0 0 0 0 0 0 0

K01725 0 0 0 0 0 0 0 0 0 0 0 0 0 0 0 0 0 0 0 0 0 0 0 0 0 0 0 0 0 0 0 0 0 0 0 0 0 0 0 0 0 0 0 0 0 0 0 0

K01726 0 0 0 0 0 0 0 0 0 0 0 0 0 0 0 0 0 0 0 0 0 0 0 0 0 0 0 0 0 0 0 0 0 0 0 0 0 0 0 0 0 0 0 0 0 0 0 0

K01727 0 0 0 0 0 0 0 0 0 0 0 0 0 0 0 0 0 0 0 0 0 0 0 0 0 0 0 0 0 0 0 0 0 0 0 0 0 0 0 0 0 0 0 0 0 0 0 0

K01728 0 0 0 0 0 0 0 0 0 0 0 0 0 0 0 0 0 0 0 0 0 0 0 0 0 0 0 0 0 0 0 0 0 0 0 0 0 0 0 0 0 0 0 0 0 0 0 0

K01729 0 0 0 0 0 0 0 0 0 0 0 0 0 0 0 0 0 0 0 0 0 0 0 0 0 0 0 0 0 0 0 0 0 0 0 0 0 0 0 0 0 0 0 0 0 0 0 0

K01730 0 0 0 0 0 0 0 0 0 0 0 0 0 0 0 0 0 0 0 0 0 0 0 0 0 0 0 0 0 0 0 0 0 0 0 0 0 0 0 0 0 0 0 0 0 0 0 0

K01731 0 0 0 0 0 0 0 0 0 0 0 0 0 0 0 0 0 0 0 0 0 0 0 0 0 0 0 0 0 0 0 0 0 0 0 0 0 0 0 0 0 0 0 0 0 0 0 0

K01732 0 0 0 0 0 0 0 0 0 0 0 0 0 0 0 0 0 0 0 0 0 0 0 0 0 0 0 0 0 0 0 0 0 0 0 0 0 0 0 0 0 0 0 0 0 0 0 0

K01733 511 788 680 262 1132 928 404 1392 794 818 1205 968 386 1048 783 367 994 1492 748 902 1186 1162 1474 1152 496 471 484 370 469 1393 24 1214 742 939 1150 1000 308 790 1018 717 1082 1072 584 1320 1273 698 862 1262

K01734 0 0 0 0 0 0 0 0 0 0 0 0 0 0 0 0 0 0 0 0 0 0 0 0 0 0 0 0 0 0 0 0 0 0 0 0 0 0 0 0 0 0 0 0 0 0 0 0

K01735 338 420 344 166 406 320 267 564 552 574 661 728 268 601 372 202 508 856 526 476 648 784 838 582 354 338 346 218 292 502 24 648 436 638 772 654 212 438 429 340 452 498 327 580 572 350 470 533

K01736 478 613 540 239 904 748 379 1138 702 754 1104 864 351 896 622 320 784 1289 725 730 994 1102 1322 1029 452 471 462 308 428 1075 24 963 596 843 980 901 308 728 790 546 776 827 448 960 978 619 682 1080

K01737 478 724 645 262 978 798 404 1219 768 818 1142 968 386 926 719 320 919 1382 748 822 1088 1127 1335 1064 473 471 472 308 458 1206 24 1038 648 866 1094 934 308 759 916 668 952 984 510 1170 1161 658 788 1134

K01738 465 678 683 294 1046 837 446 1354 782 916 1234 1013 405 842 718 294 932 1454 796 739 1054 1224 1280 1115 450 496 474 246 468 1246 24 894 582 811 1078 896 336 753 879 725 974 992 434 1098 1120 758 756 1180

K01739 0 0 0 0 0 0 0 0 0 0 0 0 0 0 0 0 0 0 0 0 0 0 0 0 0 0 0 0 0 0 0 0 0 0 0 0 0 0 0 0 0 0 0 0 0 0 0 0

K01740 86 58 180 79 459 354 116 637 124 223 348 130 46 109 186 48 190 375 106 58 171 284 208 319 12 56 34 9 59 488 0 82 78 106 124 104 66 79 155 228 320 250 28 182 168 284 102 404

K01741 0 0 0 0 0 0 0 0 0 0 0 0 0 0 0 0 0 0 0 0 0 0 0 0 0 0 0 0 0 0 0 0 0 0 0 0 0 0 0 0 0 0 0 0 0 0 0 0

K01743 0 0 0 0 0 0 0 0 0 0 0 0 0 0 0 0 0 0 0 0 0 0 0 0 0 0 0 0 0 0 0 0 0 0 0 0 0 0 0 0 0 0 0 0 0 0 0 0

K01744 0 0 0 0 0 0 0 0 0 0 0 0 0 0 0 0 0 0 0 0 0 0 0 0 0 0 0 0 0 0 0 0 0 0 0 0 0 0 0 0 0 0 0 0 0 0 0 0

K01745 42 200 159 49 158 147 64 144 106 120 225 154 91 132 160 46 234 210 164 205 234 156 234 208 84 102 94 19 116 243 0 164 68 67 182 137 58 236 295 157 201 244 80 337 382 125 184 244

K01746 0 0 0 0 0 0 0 0 0 0 0 0 0 0 0 0 0 0 0 0 0 0 0 0 0 0 0 0 0 0 0 0 0 0 0 0 0 0 0 0 0 0 0 0 0 0 0 0

K01749 437 524 486 214 819 650 340 1074 662 698 917 814 296 794 560 275 685 1173 584 618 853 970 1102 855 388 368 378 290 342 963 24 874 580 799 912 798 249 524 620 511 750 740 430 833 780 533 604 891

K01750 66 86 69 15 202 176 12 212 70 26 102 40 10 156 93 53 88 150 12 128 139 53 179 130 34 4 19 71 28 252 0 222 116 118 99 116 8 60 126 65 146 128 103 175 136 44 90 185

K01751 0 0 0 0 0 0 0 0 0 0 0 0 0 0 0 0 0 0 0 0 0 0 0 0 0 0 0 0 0 0 0 0 0 0 0 0 0 0 0 0 0 0 0 0 0 0 0 0

K01752 42 311 264 72 232 196 88 226 170 184 263 258 126 162 256 46 369 303 188 297 328 181 247 243 106 102 104 19 146 374 0 240 121 90 296 170 58 266 420 279 376 401 141 547 564 164 290 298

K01753 0 0 0 0 0 0 0 0 0 0 0 0 0 0 0 0 0 0 0 0 0 0 0 0 0 0 0 0 0 0 0 0 0 0 0 0 0 0 0 0 0 0 0 0 0 0 0 0

K01754 338 420 344 166 406 320 267 564 552 574 661 728 268 601 372 202 508 856 526 476 648 784 838 582 354 338 346 218 292 502 24 648 436 638 772 654 212 438 429 340 452 498 327 580 572 350 470 533

K01755 437 524 486 214 819 650 340 1074 662 698 917 814 296 794 560 275 685 1173 584 618 853 970 1102 855 388 368 378 290 342 963 24 874 580 799 912 798 249 524 620 511 750 740 430 833 780 533 604 891

K01756 478 724 645 262 978 798 404 1219 768 818 1142 968 386 926 719 320 919 1382 748 822 1088 1127 1335 1064 473 471 472 308 458 1206 24 1038 648 866 1094 934 308 759 916 668 952 984 510 1170 1161 658 788 1134

K01757 0 0 0 0 0 0 0 0 0 0 0 0 0 0 0 0 0 0 0 0 0 0 0 0 0 0 0 0 0 0 0 0 0 0 0 0 0 0 0 0 0 0 0 0 0 0 0 0

K01758 0 0 0 0 1 0 0 0 0 0 0 0 0 0 0 0 0 0 0 0 0 0 0 0 0 0 0 0 0 0 0 0 0 0 0 0 0 0 0 0 0 0 0 0 0 0 0 0

K01759 413 642 537 230 618 513 352 748 700 720 924 923 368 768 576 254 754 1106 701 728 923 958 1112 846 448 446 447 246 426 810 24 858 526 750 997 841 279 704 748 513 668 791 434 941 978 478 670 834

K01760 0 0 0 0 0 0 0 0 0 0 0 0 0 0 0 0 0 0 0 0 0 0 0 0 0 0 0 0 0 0 0 0 0 0 0 0 0 0 0 0 0 0 0 0 0 0 0 0

K01761 0 0 0 0 1 0 0 0 0 0 0 0 0 0 0 0 0 0 0 0 0 0 0 0 0 0 0 0 0 0 0 0 0 0 0 0 0 0 0 0 0 0 0 0 0 0 0 0

K01762 0 0 0 0 0 0 0 0 0 0 0 0 0 0 0 0 0 0 0 0 0 0 0 0 0 0 0 0 0 0 0 0 0 0 0 0 0 0 0 0 0 0 0 0 0 0 0 0

K01766 0 0 0 0 0 0 0 0 0 0 0 0 0 0 0 0 0 0 0 0 0 0 0 0 0 0 0 0 0 0 0 0 0 0 0 0 0 0 0 0 0 0 0 0 0 0 0 0

K01768 338 420 344 166 406 320 267 564 552 574 661 728 268 601 372 202 508 856 526 476 648 784 838 582 354 338 346 218 292 502 24 648 436 638 772 654 212 438 429 340 452 498 327 580 572 350 470 533

K01769 0 0 0 0 0 0 0 0 0 0 0 0 0 0 0 0 0 0 0 0 0 0 0 0 0 0 0 0 0 0 0 0 0 0 0 0 0 0 0 0 0 0 0 0 0 0 0 0

K01770 0 0 0 0 0 0 0 0 0 0 0 0 0 0 0 0 0 0 0 0 0 0 0 0 0 0 0 0 0 0 0 0 0 0 0 0 0 0 0 0 0 0 0 0 0 0 0 0

K01771 0 0 0 0 0 0 0 0 0 0 0 0 0 0 0 0 0 0 0 0 0 0 0 0 0 0 0 0 0 0 0 0 0 0 0 0 0 0 0 0 0 0 0 0 0 0 0 0

K01772 338 420 344 166 406 320 267 564 552 574 661 728 268 601 372 202 508 856 526 476 648 784 838 582 354 338 346 218 292 502 24 648 436 638 772 654 212 438 429 340 452 498 327 580 572 350 470 533

K01774 0 0 0 0 0 0 0 0 0 0 0 0 0 0 0 0 0 0 0 0 0 0 0 0 0 0 0 0 0 0 0 0 0 0 0 0 0 0 0 0 0 0 0 0 0 0 0 0

K01775 338 420 344 166 406 320 267 564 552 574 661 728 268 601 372 202 508 856 526 476 648 784 838 582 354 338 346 218 292 502 24 648 436 638 772 654 212 438 429 340 452 498 327 580 572 350 470 533

K01776 338 420 344 166 406 320 267 564 552 574 661 728 268 601 372 202 508 856 526 476 648 784 838 582 354 338 346 218 292 502 24 648 436 638 772 654 212 438 429 340 452 498 327 580 572 350 470 533

K01777 0 0 0 0 0 0 0 0 0 0 0 0 0 0 0 0 0 0 0 0 0 0 0 0 0 0 0 0 0 0 0 0 0 0 0 0 0 0 0 0 0 0 0 0 0 0 0 0

K01778 404 460 451 214 688 536 340 911 636 698 856 814 296 673 496 228 610 1078 584 538 754 935 962 790 365 368 367 228 330 794 24 728 486 726 856 732 249 493 518 462 620 652 355 682 668 493 530 764

K01779 42 200 159 49 160 147 64 144 106 120 225 154 91 132 160 46 234 210 164 205 234 156 234 208 84 102 94 19 116 243 0 164 68 67 182 137 58 236 295 157 201 244 80 337 382 125 184 244

K01780 0 0 0 0 0 0 0 0 0 0 0 0 0 0 0 0 0 0 0 0 0 0 0 0 0 0 0 0 0 0 0 0 0 0 0 0 0 0 0 0 0 0 0 0 0 0 0 0

K01781 0 0 0 0 0 0 0 0 0 0 0 0 0 0 0 0 0 0 0 0 0 0 0 0 0 0 0 0 0 0 0 0 0 0 0 0 0 0 0 0 0 0 0 0 0 0 0 0

K01782 338 420 344 166 406 320 267 564 552 574 661 728 268 601 372 202 508 856 526 476 648 784 838 582 354 338 346 218 292 502 24 648 436 638 772 654 212 438 429 340 452 498 327 580 572 350 470 533

K01783 380 620 503 216 564 468 331 708 657 694 886 882 358 732 532 247 742 1066 690 680 883 941 1072 790 438 441 440 238 409 745 24 812 504 705 954 792 270 674 724 497 653 742 406 916 953 474 654 776

K01784 957 1448 1290 525 2000 1627 807 2456 1535 1636 2286 1936 773 1852 1438 641 1838 2793 1497 1645 2175 2254 2670 2156 946 942 944 617 916 2442 48 2110 1297 1732 2188 1869 615 1518 1831 1336 1903 1968 1019 2340 2322 1316 1576 2269

K01785 0 0 0 0 0 0 0 0 0 0 0 0 0 0 0 0 0 0 0 0 0 0 0 0 0 0 0 0 0 0 0 0 0 0 0 0 0 0 0 0 0 0 0 0 0 0 0 0

K01786 0 0 0 0 0 0 0 0 0 0 0 0 0 0 0 0 0 0 0 0 0 0 0 0 0 0 0 0 0 0 0 0 0 0 0 0 0 0 0 0 0 0 0 0 0 0 0 0

K01787 0 0 0 0 0 0 0 0 0 0 0 0 0 0 0 0 0 0 0 0 0 0 0 0 0 0 0 0 0 0 0 0 0 0 0 0 0 0 0 0 0 0 0 0 0 0 0 0

K01788 0 0 0 0 0 0 0 0 0 0 0 0 0 0 0 0 0 0 0 0 0 0 0 0 0 0 0 0 0 0 0 0 0 0 0 0 0 0 0 0 0 0 0 0 0 0 0 0

K01789 0 0 0 0 0 0 0 0 0 0 0 0 0 0 0 0 0 0 0 0 0 0 0 0 0 0 0 0 0 0 0 0 0 0 0 0 0 0 0 0 0 0 0 0 0 0 0 0

K01790 484 542 559 246 1030 804 400 1373 702 797 1070 859 314 832 654 296 774 1340 632 650 918 1104 1186 998 390 394 392 290 362 1172 24 879 608 866 952 825 278 548 686 617 903 854 430 912 851 673 647 1064

K01791 498 857 784 301 1144 924 440 1358 876 908 1221 1112 432 991 844 327 1066 1545 784 944 1222 1169 1388 1169 504 476 490 318 504 1432 24 1186 724 910 1250 1018 316 820 1065 806 1142 1181 599 1404 1368 700 910 1247

K01792 0 0 0 0 0 0 0 0 0 0 0 0 0 0 0 0 0 0 0 0 0 0 0 0 0 0 0 0 0 0 0 0 0 0 0 0 0 0 0 0 0 0 0 0 0 0 0 0

K01795 0 0 0 0 0 0 0 0 0 0 0 0 0 0 0 0 0 0 0 0 0 0 0 0 0 0 0 0 0 0 0 0 0 0 0 0 0 0 0 0 0 0 0 0 0 0 0 0

K01796 0 0 0 0 0 0 0 0 0 0 0 0 0 0 0 0 0 0 0 0 0 0 0 0 0 0 0 0 0 0 0 0 0 0 0 0 0 0 0 0 0 0 0 0 0 0 0 0

K01797 0 0 0 0 0 0 0 0 0 0 0 0 0 0 0 0 0 0 0 0 0 0 0 0 0 0 0 0 0 0 0 0 0 0 0 0 0 0 0 0 0 0 0 0 0 0 0 0

K01798 0 0 0 0 0 0 0 0 0 0 0 0 0 0 0 0 0 0 0 0 0 0 0 0 0 0 0 0 0 0 0 0 0 0 0 0 0 0 0 0 0 0 0 0 0 0 0 0

K01799 0 0 0 0 0 0 0 0 0 0 0 0 0 0 0 0 0 0 0 0 0 0 0 0 0 0 0 0 0 0 0 0 0 0 0 0 0 0 0 0 0 0 0 0 0 0 0 0

K01800 0 0 0 0 0 0 0 0 0 0 0 0 0 0 0 0 0 0 0 0 0 0 0 0 0 0 0 0 0 0 0 0 0 0 0 0 0 0 0 0 0 0 0 0 0 0 0 0

K01801 0 0 0 0 0 0 0 0 0 0 0 0 0 0 0 0 0 0 0 0 0 0 0 0 0 0 0 0 0 0 0 0 0 0 0 0 0 0 0 0 0 0 0 0 0 0 0 0

K01802 412 684 538 216 767 630 331 898 684 694 955 882 358 854 596 294 818 1202 690 760 982 976 1212 924 462 441 451 300 420 967 24 1052 598 778 1011 858 270 704 826 546 784 830 482 1067 1065 514 729 904

K01803 478 724 645 262 978 798 404 1219 768 818 1142 968 386 926 719 320 919 1382 748 822 1088 1127 1335 1064 473 471 472 308 458 1206 24 1038 648 866 1094 934 308 759 916 668 952 984 510 1170 1161 658 788 1134

K01804 0 0 0 0 0 0 0 0 0 0 0 0 0 0 0 0 0 0 0 0 0 0 0 0 0 0 0 0 0 0 0 0 0 0 0 0 0 0 0 0 0 0 0 0 0 0 0 0

K01805 0 0 0 0 0 0 0 0 0 0 0 0 0 0 0 0 0 0 0 0 0 0 0 0 0 0 0 0 0 0 0 0 0 0 0 0 0 0 0 0 0 0 0 0 0 0 0 0

K01807 478 724 645 262 978 798 404 1219 768 818 1142 968 386 926 719 320 919 1382 748 822 1088 1127 1335 1064 473 471 472 308 458 1206 24 1038 648 866 1094 934 308 759 916 668 952 984 510 1170 1161 658 788 1134

K01808 0 0 0 0 0 0 0 0 0 0 0 0 0 0 0 0 0 0 0 0 0 0 0 0 0 0 0 0 0 0 0 0 0 0 0 0 0 0 0 0 0 0 0 0 0 0 0 0

K01809 370 484 379 166 560 451 267 736 578 574 725 728 268 722 436 248 584 965 526 555 748 820 978 670 377 338 358 280 304 689 24 820 530 711 829 720 212 469 531 389 582 586 402 730 684 390 545 660

K01810 338 420 344 166 406 320 267 564 552 574 661 728 268 601 372 202 508 856 526 476 648 784 838 582 354 338 346 218 292 502 24 648 436 638 772 654 212 438 429 340 452 498 327 580 572 350 470 533

K01811 0 0 0 0 0 0 0 0 0 0 0 0 0 0 0 0 0 0 0 0 0 0 0 0 0 0 0 0 0 0 0 0 0 0 0 0 0 0 0 0 0 0 0 0 0 0 0 0

K01812 0 0 0 0 0 0 0 0 0 0 0 0 0 0 0 0 0 0 0 0 0 0 0 0 0 0 0 0 0 0 0 0 0 0 0 0 0 0 0 0 0 0 0 0 0 0 0 0

K01813 0 0 0 0 0 0 0 0 0 0 0 0 0 0 0 0 0 0 0 0 0 0 0 0 0 0 0 0 0 0 0 0 0 0 0 0 0 0 0 0 0 0 0 0 0 0 0 0

K01814 504 564 593 260 1102 866 412 1422 746 823 1111 900 324 866 683 302 786 1395 644 680 958 1121 1225 1064 400 398 399 298 380 1254 24 954 631 887 995 874 286 578 710 633 918 894 458 936 876 676 663 1122

K01815 0 0 0 0 0 0 0 0 0 0 0 0 0 0 0 0 0 0 0 0 0 0 0 0 0 0 0 0 0 0 0 0 0 0 0 0 0 0 0 0 0 0 0 0 0 0 0 0

K01816 0 0 0 0 0 0 0 0 0 0 0 0 0 0 0 0 0 0 0 0 0 0 0 0 0 0 0 0 0 0 0 0 0 0 0 0 0 0 0 0 0 0 0 0 0 0 0 0

K01817 437 524 486 214 842 666 340 1084 662 698 917 814 296 794 560 275 685 1187 584 618 853 970 1102 878 388 368 378 290 342 980 24 904 580 799 912 798 249 524 620 511 750 740 430 833 780 533 604 891

K01818 0 0 0 0 0 0 0 0 0 0 0 0 0 0 0 0 0 0 0 0 0 0 0 0 0 0 0 0 0 0 0 0 0 0 0 0 0 0 0 0 0 0 0 0 0 0 0 0

K01819 0 0 0 0 0 0 0 0 0 0 0 0 0 0 0 0 0 0 0 0 0 0 0 0 0 0 0 0 0 0 0 0 0 0 0 0 0 0 0 0 0 0 0 0 0 0 0 0

K01820 0 0 0 0 0 0 0 0 0 0 0 0 0 0 0 0 0 0 0 0 0 0 0 0 0 0 0 0 0 0 0 0 0 0 0 0 0 0 0 0 0 0 0 0 0 0 0 0

K01821 66 40 107 47 284 216 72 348 84 124 195 86 28 72 124 27 101 222 59 62 106 150 124 208 12 30 21 9 38 292 0 80 50 88 83 77 37 54 90 122 168 154 28 103 96 144 59 230

K01822 0 0 0 0 0 0 0 0 0 0 0 0 0 0 0 0 0 0 0 0 0 0 0 0 0 0 0 0 0 0 0 0 0 0 0 0 0 0 0 0 0 0 0 0 0 0 0 0

K01823 478 724 645 262 978 798 404 1219 768 818 1142 968 386 926 719 320 919 1382 748 822 1088 1127 1335 1064 473 471 472 308 458 1206 24 1038 648 866 1094 934 308 759 916 668 952 984 510 1170 1161 658 788 1134

K01825 0 0 0 0 0 0 0 0 0 0 0 0 0 0 0 0 0 0 0 0 0 0 0 0 0 0 0 0 0 0 0 0 0 0 0 0 0 0 0 0 0 0 0 0 0 0 0 0

K01826 0 0 0 0 0 0 0 0 0 0 0 0 0 0 0 0 0 0 0 0 0 0 0 0 0 0 0 0 0 0 0 0 0 0 0 0 0 0 0 0 0 0 0 0 0 0 0 0

K01828 0 0 0 0 0 0 0 0 0 0 0 0 0 0 0 0 0 0 0 0 0 0 0 0 0 0 0 0 0 0 0 0 0 0 0 0 0 0 0 0 0 0 0 0 0 0 0 0

K01829 0 0 0 0 0 0 0 0 0 0 0 0 0 0 0 0 0 0 0 0 0 0 0 0 0 0 0 0 0 0 0 0 0 0 0 0 0 0 0 0 0 0 0 0 0 0 0 0

K01830 0 0 0 0 0 0 0 0 0 0 0 0 0 0 0 0 0 0 0 0 0 0 0 0 0 0 0 0 0 0 0 0 0 0 0 0 0 0 0 0 0 0 0 0 0 0 0 0

K01834 883 1184 1096 476 1688 1350 743 2139 1403 1516 2000 1782 682 1599 1214 549 1528 2474 1333 1360 1842 2062 2297 1876 838 840 839 536 788 2017 48 1796 1135 1592 1950 1666 556 1252 1434 1130 1572 1636 864 1852 1828 1151 1318 1898

K01835 0 0 0 0 24 16 0 9 0 0 3 0 0 0 0 0 0 14 0 0 0 0 0 23 0 0 0 0 0 18 0 26 0 0 0 0 0 0 0 0 0 0 0 0 0 0 0 0

K01837 0 0 0 0 0 0 0 0 0 0 0 0 0 0 0 0 0 0 0 0 0 0 0 0 0 0 0 0 0 0 0 0 0 0 0 0 0 0 0 0 0 0 0 0 0 0 0 0

K01838 0 0 0 0 0 0 0 0 0 0 0 0 0 0 0 0 0 0 0 0 0 0 0 0 0 0 0 0 0 0 0 0 0 0 0 0 0 0 0 0 0 0 0 0 0 0 0 0

K01839 0 0 0 0 0 0 0 0 0 0 0 0 0 0 0 0 0 0 0 0 0 0 0 0 0 0 0 0 0 0 0 0 0 0 0 0 0 0 0 0 0 0 0 0 0 0 0 0

K01840 520 924 804 312 1166 960 476 1372 873 938 1367 1122 478 1058 894 366 1153 1606 912 1028 1322 1284 1568 1292 558 574 566 328 574 1462 24 1203 716 933 1276 1072 366 994 1210 825 1152 1236 589 1507 1542 783 972 1378

K01841 0 0 0 0 0 0 0 0 0 0 0 0 0 0 0 0 0 0 0 0 0 0 0 0 0 0 0 0 0 0 0 0 0 0 0 0 0 0 0 0 0 0 0 0 0 0 0 0

K01842 0 0 0 0 0 0 0 0 0 0 0 0 0 0 0 0 0 0 0 0 0 0 0 0 0 0 0 0 0 0 0 0 0 0 0 0 0 0 0 0 0 0 0 0 0 0 0 0

K01843 0 0 0 0 23 16 0 9 0 0 3 0 0 0 0 0 0 14 0 0 0 0 0 23 0 0 0 0 0 18 0 34 0 0 0 0 0 0 0 0 0 0 0 0 0 0 0 0

K01844 0 0 0 0 0 0 0 0 0 0 0 0 0 0 0 0 0 0 0 0 0 0 0 0 0 0 0 0 0 0 0 0 0 0 0 0 0 0 0 0 0 0 0 0 0 0 0 0

K01845 437 524 486 214 819 650 340 1074 662 698 917 814 296 794 560 275 685 1173 584 618 853 970 1102 855 388 368 378 290 342 963 24 874 580 799 912 798 249 524 620 511 750 740 430 833 780 533 604 891

K01846 0 0 0 0 0 0 0 0 0 0 0 0 0 0 0 0 0 0 0 0 0 0 0 0 0 0 0 0 0 0 0 0 0 0 0 0 0 0 0 0 0 0 0 0 0 0 0 0

K01847 0 0 0 0 0 0 0 0 0 0 0 0 0 0 0 0 0 0 0 0 0 0 0 0 0 0 0 0 0 0 0 0 0 0 0 0 0 0 0 0 0 0 0 0 0 0 0 0

K01848 42 200 159 49 158 147 64 144 106 120 225 154 91 132 160 46 234 210 164 205 234 156 234 208 84 102 94 19 116 243 0 164 68 67 182 137 58 236 295 157 201 244 80 337 382 125 184 244

K01849 42 200 159 49 158 147 64 144 106 120 225 154 91 132 160 46 234 210 164 205 234 156 234 208 84 102 94 19 116 243 0 164 68 67 182 137 58 236 295 157 201 244 80 337 382 125 184 244

K01850 0 0 0 0 0 0 0 0 0 0 0 0 0 0 0 0 0 0 0 0 0 0 0 0 0 0 0 0 0 0 0 0 0 0 0 0 0 0 0 0 0 0 0 0 0 0 0 0

K01851 0 0 0 0 0 0 0 0 0 0 0 0 0 0 0 0 0 0 0 0 0 0 0 0 0 0 0 0 0 0 0 0 0 0 0 0 0 0 0 0 0 0 0 0 0 0 0 0

K01852 0 0 0 0 0 0 0 0 0 0 0 0 0 0 0 0 0 0 0 0 0 0 0 0 0 0 0 0 0 0 0 0 0 0 0 0 0 0 0 0 0 0 0 0 0 0 0 0

K01853 0 0 0 0 0 0 0 0 0 0 0 0 0 0 0 0 0 0 0 0 0 0 0 0 0 0 0 0 0 0 0 0 0 0 0 0 0 0 0 0 0 0 0 0 0 0 0 0

K01854 52 40 107 47 258 200 72 338 84 124 192 86 28 72 124 27 101 208 59 44 106 150 124 186 12 30 21 9 38 274 0 50 50 64 83 77 37 54 90 122 168 154 28 103 96 144 59 230

K01856 0 0 0 0 0 0 0 0 0 0 0 0 0 0 0 0 0 0 0 0 0 0 0 0 0 0 0 0 0 0 0 0 0 0 0 0 0 0 0 0 0 0 0 0 0 0 0 0

K01857 0 0 0 0 0 0 0 0 0 0 0 0 0 0 0 0 0 0 0 0 0 0 0 0 0 0 0 0 0 0 0 0 0 0 0 0 0 0 0 0 0 0 0 0 0 0 0 0

K01858 99 104 142 47 412 330 72 511 110 124 253 86 28 194 188 74 176 317 59 142 204 186 263 256 35 30 32 71 49 456 0 196 144 161 140 143 37 85 192 171 298 242 103 254 208 184 134 358

K01860 0 0 0 0 0 0 0 0 0 0 0 0 0 0 0 0 0 0 0 0 0 0 0 0 0 0 0 0 0 0 0 0 0 0 0 0 0 0 0 0 0 0 0 0 0 0 0 0

K01865 0 0 0 0 0 0 0 0 0 0 0 0 0 0 0 0 0 0 0 0 0 0 0 0 0 0 0 0 0 0 0 0 0 0 0 0 0 0 0 0 0 0 0 0 0 0 0 0

K01866 478 724 645 262 978 798 404 1219 768 818 1142 968 386 926 719 320 919 1382 748 822 1088 1127 1335 1064 473 471 472 308 458 1206 24 1038 648 866 1094 934 308 759 916 668 952 984 510 1170 1161 658 788 1134

K01867 478 724 645 262 978 798 404 1219 768 818 1142 968 386 926 719 320 919 1382 748 822 1088 1127 1335 1064 473 471 472 308 458 1206 24 1038 648 866 1094 934 308 759 916 668 952 984 510 1170 1161 658 788 1134

K01868 478 724 645 262 978 798 404 1219 768 818 1142 968 386 926 719 320 919 1382 748 822 1088 1127 1335 1064 473 471 472 308 458 1206 24 1038 648 866 1094 934 308 759 916 668 952 984 510 1170 1161 658 788 1134

K01869 478 724 645 262 978 798 404 1219 768 818 1142 968 386 926 719 320 919 1382 748 822 1088 1127 1335 1064 473 471 472 308 458 1206 24 1038 648 866 1094 934 308 759 916 668 952 984 510 1170 1161 658 788 1134

K01870 478 724 645 262 978 798 404 1219 768 818 1142 968 386 926 719 320 919 1382 748 822 1088 1127 1335 1064 473 471 472 308 458 1206 24 1038 648 866 1094 934 308 759 916 668 952 984 510 1170 1161 658 788 1134

K01872 478 724 645 262 978 798 404 1219 768 818 1142 968 386 926 719 320 919 1382 748 822 1088 1127 1335 1064 473 471 472 308 458 1206 24 1038 648 866 1094 934 308 759 916 668 952 984 510 1170 1161 658 788 1134

K01873 478 724 645 262 978 798 404 1219 768 818 1142 968 386 926 719 320 919 1382 748 822 1088 1127 1335 1064 473 471 472 308 458 1206 24 1038 648 866 1094 934 308 759 916 668 952 984 510 1170 1161 658 788 1134

K01874 478 724 645 262 978 798 404 1219 768 818 1142 968 386 926 719 320 919 1382 748 822 1088 1127 1335 1064 473 471 472 308 458 1206 24 1038 648 866 1094 934 308 759 916 668 952 984 510 1170 1161 658 788 1134

K01875 478 724 645 262 978 798 404 1219 768 818 1142 968 386 926 719 320 919 1382 748 822 1088 1127 1335 1064 473 471 472 308 458 1206 24 1038 648 866 1094 934 308 759 916 668 952 984 510 1170 1161 658 788 1134

K01876 478 724 645 262 978 798 404 1219 768 818 1142 968 386 926 719 320 919 1382 748 822 1088 1127 1335 1064 473 471 472 308 458 1206 24 1038 648 866 1094 934 308 759 916 668 952 984 510 1170 1161 658 788 1134

K01878 0 0 0 0 0 0 0 0 0 0 0 0 0 0 0 0 0 0 0 0 0 0 0 0 0 0 0 0 0 0 0 0 0 0 0 0 0 0 0 0 0 0 0 0 0 0 0 0

K01879 0 0 0 0 0 0 0 0 0 0 0 0 0 0 0 0 0 0 0 0 0 0 0 0 0 0 0 0 0 0 0 0 0 0 0 0 0 0 0 0 0 0 0 0 0 0 0 0

K01880 478 724 645 262 978 798 404 1219 768 818 1142 968 386 926 719 320 919 1382 748 822 1088 1127 1335 1064 473 471 472 308 458 1206 24 1038 648 866 1094 934 308 759 916 668 952 984 510 1170 1161 658 788 1134

K01881 478 724 645 262 978 798 404 1219 768 818 1142 968 386 926 719 320 919 1382 748 822 1088 1127 1335 1064 473 471 472 308 458 1206 24 1038 648 866 1094 934 308 759 916 668 952 984 510 1170 1161 658 788 1134

K01883 464 724 645 262 948 782 395 1210 768 818 1139 968 386 926 703 320 919 1368 748 804 1088 1127 1335 1027 473 471 472 308 458 1188 24 1008 648 842 1094 934 308 759 916 668 952 976 510 1170 1161 658 788 1134

K01884 0 0 0 0 0 0 0 0 0 0 0 0 0 0 0 0 0 0 0 0 0 0 0 0 0 0 0 0 0 0 0 0 0 0 0 0 0 0 0 0 0 0 0 0 0 0 0 0

K01885 478 724 645 262 978 798 404 1219 768 818 1142 968 386 926 719 320 919 1382 748 822 1088 1127 1335 1064 473 471 472 308 458 1206 24 1038 648 866 1094 934 308 759 916 668 952 984 510 1170 1161 658 788 1134

K01886 0 0 0 0 0 0 0 0 0 0 0 0 0 0 0 0 0 0 0 0 0 0 0 0 0 0 0 0 0 0 0 0 0 0 0 0 0 0 0 0 0 0 0 0 0 0 0 0

K01887 478 724 645 262 978 798 404 1219 768 818 1142 968 386 926 719 320 919 1382 748 822 1088 1127 1335 1064 473 471 472 308 458 1206 24 1038 648 866 1094 934 308 759 916 668 952 984 510 1170 1161 658 788 1134

K01889 478 724 645 262 978 798 404 1219 768 818 1142 968 386 926 719 320 919 1382 748 822 1088 1127 1335 1064 473 471 472 308 458 1206 24 1038 648 866 1094 934 308 759 916 668 952 984 510 1170 1161 658 788 1134

K01890 478 724 645 262 978 798 404 1219 768 818 1142 968 386 926 719 320 919 1382 748 822 1088 1127 1335 1064 473 471 472 308 458 1206 24 1038 648 866 1094 934 308 759 916 668 952 984 510 1170 1161 658 788 1134

K01892 478 724 645 262 978 798 404 1219 768 818 1142 968 386 926 719 320 919 1382 748 822 1088 1127 1335 1064 473 471 472 308 458 1206 24 1038 648 866 1094 934 308 759 916 668 952 984 510 1170 1161 658 788 1134

K01893 412 684 538 216 695 582 331 872 684 694 947 882 358 854 596 294 818 1160 690 760 982 976 1212 855 462 441 451 300 420 914 24 958 598 778 1011 858 270 704 826 546 784 830 482 1067 1065 514 729 904

K01894 0 0 0 0 0 0 0 0 0 0 0 0 0 0 0 0 0 0 0 0 0 0 0 0 0 0 0 0 0 0 0 0 0 0 0 0 0 0 0 0 0 0 0 0 0 0 0 0

K01895 526 746 679 278 1110 891 433 1286 811 844 1188 1008 396 961 780 327 931 1466 760 889 1128 1144 1374 1202 484 476 480 318 475 1324 24 1174 671 936 1136 984 316 789 940 684 967 1041 538 1194 1186 662 804 1192

K01896 0 0 0 0 0 0 0 0 0 0 0 0 0 0 0 0 0 0 0 0 0 0 0 0 0 0 0 0 0 0 0 0 0 0 0 0 0 0 0 0 0 0 0 0 0 0 0 0

K01897 338 420 344 166 430 336 267 572 552 574 664 728 268 601 372 202 508 870 526 476 648 784 838 604 354 338 346 218 292 520 24 678 436 638 772 654 212 438 429 340 452 498 327 580 572 350 470 533

K01902 478 724 645 262 978 798 404 1219 768 818 1142 968 386 926 719 320 919 1382 748 822 1088 1127 1335 1064 473 471 472 308 458 1206 24 1038 648 866 1094 934 308 759 916 668 952 984 510 1170 1161 658 788 1134

K01903 478 724 645 262 978 798 404 1219 768 818 1142 968 386 926 719 320 919 1382 748 822 1088 1127 1335 1064 473 471 472 308 458 1206 24 1038 648 866 1094 934 308 759 916 668 952 984 510 1170 1161 658 788 1134

K01904 0 0 0 0 0 0 0 0 0 0 0 0 0 0 0 0 0 0 0 0 0 0 0 0 0 0 0 0 0 0 0 0 0 0 0 0 0 0 0 0 0 0 0 0 0 0 0 0

K01905 74 376 298 72 363 310 88 390 197 184 324 258 126 283 320 92 444 398 188 376 428 216 386 308 129 102 116 81 157 544 0 386 215 163 352 236 58 296 522 328 506 489 216 698 676 204 364 426

K01906 33 18 73 32 205 154 52 298 40 98 154 45 18 37 78 20 89 167 48 14 66 133 84 130 1 26 13 0 21 209 0 5 28 42 40 28 28 24 66 106 152 105 0 78 72 140 43 173

K01907 0 0 0 0 0 0 0 0 0 0 0 0 0 0 0 0 0 0 0 0 0 0 0 0 0 0 0 0 0 0 0 0 0 0 0 0 0 0 0 0 0 0 0 0 0 0 0 0

K01908 0 0 0 0 0 0 0 0 0 0 0 0 0 0 0 0 0 0 0 0 0 0 0 0 0 0 0 0 0 0 0 0 0 0 0 0 0 0 0 0 0 0 0 0 0 0 0 0

K01909 0 0 0 0 0 0 0 0 0 0 0 0 0 0 0 0 0 0 0 0 0 0 0 0 0 0 0 0 0 0 0 0 0 0 0 0 0 0 0 0 0 0 0 0 0 0 0 0

K01910 0 0 0 0 0 0 0 0 0 0 0 0 0 0 0 0 0 0 0 0 0 0 0 0 0 0 0 0 0 0 0 0 0 0 0 0 0 0 0 0 0 0 0 0 0 0 0 0

K01911 0 0 0 0 0 0 0 0 0 0 0 0 0 0 0 0 0 0 0 0 0 0 0 0 0 0 0 0 0 0 0 0 0 0 0 0 0 0 0 0 0 0 0 0 0 0 0 0

K01912 504 564 593 260 1172 914 412 1449 746 823 1114 900 324 866 683 302 786 1437 644 680 958 1121 1225 1116 400 398 399 298 380 1302 24 1014 631 887 995 874 286 578 710 633 918 894 458 936 876 676 663 1122

K01913 0 0 0 0 0 0 0 0 0 0 0 0 0 0 0 0 0 0 0 0 0 0 0 0 0 0 0 0 0 0 0 0 0 0 0 0 0 0 0 0 0 0 0 0 0 0 0 0

K01914 0 0 0 0 0 0 0 0 0 0 0 0 0 0 0 0 0 0 0 0 0 0 0 0 0 0 0 0 0 0 0 0 0 0 0 0 0 0 0 0 0 0 0 0 0 0 0 0

K01915 891 1362 1221 510 1794 1451 786 2244 1465 1610 2184 1896 764 1696 1329 588 1750 2643 1486 1518 2036 2201 2491 2012 912 938 925 546 888 2190 48 1882 1180 1614 2089 1754 606 1458 1705 1271 1757 1832 916 2165 2186 1272 1486 2084

K01916 478 724 645 262 954 782 404 1210 768 818 1142 968 386 926 719 320 919 1368 748 822 1088 1127 1335 1058 473 471 472 308 458 1193 24 1038 648 866 1094 934 308 759 916 668 952 984 510 1170 1161 658 788 1134

K01917 0 0 0 0 0 0 0 0 0 0 0 0 0 0 0 0 0 0 0 0 0 0 0 0 0 0 0 0 0 0 0 0 0 0 0 0 0 0 0 0 0 0 0 0 0 0 0 0

K01918 0 0 0 0 0 0 0 0 0 0 0 0 0 0 0 0 0 0 0 0 0 0 0 0 0 0 0 0 0 0 0 0 0 0 0 0 0 0 0 0 0 0 0 0 0 0 0 0

K01919 52 40 107 47 252 200 64 338 84 124 192 86 28 72 108 27 101 208 59 44 106 150 124 172 12 30 21 9 38 274 0 50 50 64 83 77 37 54 90 122 168 145 28 103 96 144 59 230

K01920 0 0 0 0 0 0 0 0 0 0 0 0 0 0 0 0 0 0 0 0 0 0 0 0 0 0 0 0 0 0 0 0 0 0 0 0 0 0 0 0 0 0 0 0 0 0 0 0

K01921 338 420 344 166 428 336 267 572 552 574 661 728 268 601 372 202 508 870 526 476 648 784 838 588 354 338 346 218 292 515 24 651 436 638 772 654 212 438 429 340 452 498 327 580 572 350 470 533

K01922 0 0 0 0 0 0 0 0 0 0 0 0 0 0 0 0 0 0 0 0 0 0 0 0 0 0 0 0 0 0 0 0 0 0 0 0 0 0 0 0 0 0 0 0 0 0 0 0

K01923 478 724 645 262 978 798 404 1219 768 818 1142 968 386 926 719 320 919 1382 748 822 1088 1127 1335 1064 473 471 472 308 458 1206 24 1038 648 866 1094 934 308 759 916 668 952 984 510 1170 1161 658 788 1134

K01924 443 500 558 260 916 720 404 1240 720 823 1045 900 324 745 603 256 710 1272 644 564 860 1086 1086 939 376 398 388 236 368 1050 24 748 537 765 938 808 286 548 608 584 788 797 383 786 764 636 588 994

K01925 457 500 558 260 922 720 412 1240 720 823 1045 900 324 745 619 256 710 1272 644 582 860 1086 1086 952 376 398 388 236 368 1050 24 748 537 790 938 808 286 548 608 584 788 806 383 786 764 636 588 994

K01926 0 0 0 0 0 0 0 0 0 0 0 0 0 0 0 0 0 0 0 0 0 0 0 0 0 0 0 0 0 0 0 0 0 0 0 0 0 0 0 0 0 0 0 0 0 0 0 0

K01928 352 420 344 166 412 320 276 564 552 574 661 728 268 601 388 202 508 856 526 494 648 784 838 595 354 338 346 218 292 502 24 648 436 662 772 654 212 438 429 340 452 507 327 580 572 350 470 533

K01929 338 420 344 166 406 320 267 564 552 574 661 728 268 601 372 202 508 856 526 476 648 784 838 582 354 338 346 218 292 502 24 648 436 638 772 654 212 438 429 340 452 498 327 580 572 350 470 533

K01930 0 0 0 0 0 0 0 0 0 0 0 0 0 0 0 0 0 0 0 0 0 0 0 0 0 0 0 0 0 0 0 0 0 0 0 0 0 0 0 0 0 0 0 0 0 0 0 0

K01932 0 0 0 0 0 0 0 0 0 0 0 0 0 0 0 0 0 0 0 0 0 0 0 0 0 0 0 0 0 0 0 0 0 0 0 0 0 0 0 0 0 0 0 0 0 0 0 0

K01933 478 724 645 262 978 798 404 1219 768 818 1142 968 386 926 719 320 919 1382 748 822 1088 1127 1335 1064 473 471 472 308 458 1206 24 1038 648 866 1094 934 308 759 916 668 952 984 510 1170 1161 658 788 1134

K01934 404 460 451 214 688 536 340 911 636 698 856 814 296 673 496 228 610 1078 584 538 754 935 962 790 365 368 367 228 330 794 24 728 486 726 856 732 249 493 518 462 620 652 355 682 668 493 530 764

K01935 33 18 73 32 205 154 52 298 40 98 154 45 18 37 78 20 89 167 48 14 66 133 84 130 1 26 13 0 21 209 0 5 28 42 40 28 28 24 66 106 152 105 0 78 72 140 43 173

K01937 478 724 645 262 978 798 404 1219 768 818 1142 968 386 926 719 320 919 1382 748 822 1088 1127 1335 1064 473 471 472 308 458 1206 24 1038 648 866 1094 934 308 759 916 668 952 984 510 1170 1161 658 788 1134

K01938 42 200 159 49 158 147 64 144 106 120 225 154 91 132 160 46 234 210 164 205 234 156 234 208 84 102 94 19 116 243 0 164 68 67 182 137 58 236 295 157 201 244 80 337 382 125 184 244

K01939 478 724 645 262 978 798 404 1219 768 818 1142 968 386 926 719 320 919 1382 748 822 1088 1127 1335 1064 473 471 472 308 458 1206 24 1038 648 866 1094 934 308 759 916 668 952 984 510 1170 1161 658 788 1134

K01940 437 524 486 214 819 650 340 1074 662 698 917 814 296 794 560 275 685 1173 584 618 853 970 1102 855 388 368 378 290 342 963 24 874 580 799 912 798 249 524 620 511 750 740 430 833 780 533 604 891

K01941 0 0 0 0 0 0 0 0 0 0 0 0 0 0 0 0 0 0 0 0 0 0 0 0 0 0 0 0 0 0 0 0 0 0 0 0 0 0 0 0 0 0 0 0 0 0 0 0

K01945 478 724 645 262 978 798 404 1219 768 818 1142 968 386 926 719 320 919 1382 748 822 1088 1127 1335 1064 473 471 472 308 458 1206 24 1038 648 866 1094 934 308 759 916 668 952 984 510 1170 1161 658 788 1134

K01946 0 0 0 0 0 0 0 0 0 0 0 0 0 0 0 0 0 0 0 0 0 0 0 0 0 0 0 0 0 0 0 0 0 0 0 0 0 0 0 0 0 0 0 0 0 0 0 0

K01947 0 0 0 0 0 0 0 0 0 0 0 0 0 0 0 0 0 0 0 0 0 0 0 0 0 0 0 0 0 0 0 0 0 0 0 0 0 0 0 0 0 0 0 0 0 0 0 0

K01949 0 0 0 0 0 0 0 0 0 0 0 0 0 0 0 0 0 0 0 0 0 0 0 0 0 0 0 0 0 0 0 0 0 0 0 0 0 0 0 0 0 0 0 0 0 0 0 0

K01950 0 0 0 0 23 16 0 9 0 0 0 0 0 0 0 0 0 14 0 0 0 0 0 6 0 0 0 0 0 13 0 0 0 0 0 0 0 0 0 0 0 0 0 0 0 0 0 0

K01951 619 1028 946 358 1550 1274 540 1874 984 1062 1622 1208 506 1251 1066 440 1330 1909 972 1170 1526 1470 1832 1546 592 604 598 398 624 1910 24 1430 861 1094 1416 1214 403 1080 1402 996 1451 1470 692 1760 1750 966 1106 1736

K01952 1065 1688 1556 621 2395 1958 944 2930 1724 1880 2700 2176 892 2056 1721 714 2173 3196 1720 1912 2515 2561 3027 2527 1042 1074 1058 645 1070 2942 48 2296 1416 1887 2453 2083 710 1808 2216 1615 2272 2366 1126 2780 2800 1584 1819 2743

K01953 520 924 804 312 1160 960 468 1372 873 938 1369 1122 478 1058 878 366 1153 1606 912 1028 1322 1284 1568 1295 558 574 566 328 574 1466 24 1230 716 933 1276 1072 366 994 1210 825 1152 1228 589 1507 1542 783 972 1378

K01955 437 524 486 214 819 650 340 1074 662 698 917 814 296 794 560 275 685 1173 584 618 853 970 1102 855 388 368 378 290 342 963 24 874 580 799 912 798 249 524 620 511 750 740 430 833 780 533 604 891

K01956 437 524 486 214 819 650 340 1074 662 698 917 814 296 794 560 275 685 1173 584 618 853 970 1102 855 388 368 378 290 342 963 24 874 580 799 912 798 249 524 620 511 750 740 430 833 780 533 604 891

K01957 0 0 0 0 0 0 0 0 0 0 0 0 0 0 0 0 0 0 0 0 0 0 0 0 0 0 0 0 0 0 0 0 0 0 0 0 0 0 0 0 0 0 0 0 0 0 0 0

K01958 0 0 0 0 0 0 0 0 0 0 0 0 0 0 0 0 0 0 0 0 0 0 0 0 0 0 0 0 0 0 0 0 0 0 0 0 0 0 0 0 0 0 0 0 0 0 0 0

K01959 52 40 107 47 276 216 64 348 84 124 195 86 28 72 108 27 101 222 59 44 106 150 124 195 12 30 21 9 38 292 0 80 50 64 83 77 37 54 90 122 168 145 28 103 96 144 59 230

K01960 140 304 301 96 572 477 136 656 216 244 481 240 119 325 347 119 410 526 223 347 439 342 496 482 120 132 126 90 166 704 0 391 212 228 322 280 96 320 486 328 500 486 182 590 590 308 318 602

K01961 338 420 344 166 406 320 267 564 552 574 661 728 268 601 372 202 508 856 526 476 648 784 838 582 354 338 346 218 292 502 24 648 436 638 772 654 212 438 429 340 452 498 327 580 572 350 470 533

K01962 0 0 0 0 0 0 0 0 0 0 0 0 0 0 0 0 0 0 0 0 0 0 0 0 0 0 0 0 0 0 0 0 0 0 0 0 0 0 0 0 0 0 0 0 0 0 0 0

K01963 0 0 0 0 0 0 0 0 0 0 0 0 0 0 0 0 0 0 0 0 0 0 0 0 0 0 0 0 0 0 0 0 0 0 0 0 0 0 0 0 0 0 0 0 0 0 0 0

K01964 28 0 0 0 13 0 17 0 0 0 0 0 0 0 32 0 0 0 0 37 0 0 0 27 0 0 0 0 0 0 0 0 0 49 0 0 0 0 0 0 0 17 0 0 0 0 0 0

K01965 0 0 0 0 0 0 0 0 0 0 0 0 0 0 0 0 0 0 0 0 0 0 0 0 0 0 0 0 0 0 0 0 0 0 0 0 0 0 0 0 0 0 0 0 0 0 0 0

K01966 0 0 0 0 0 0 0 0 0 0 0 0 0 0 0 0 0 0 0 0 0 0 0 0 0 0 0 0 0 0 0 0 0 0 0 0 0 0 0 0 0 0 0 0 0 0 0 0

K01968 0 0 0 0 0 0 0 0 0 0 0 0 0 0 0 0 0 0 0 0 0 0 0 0 0 0 0 0 0 0 0 0 0 0 0 0 0 0 0 0 0 0 0 0 0 0 0 0

K01969 0 0 0 0 0 0 0 0 0 0 0 0 0 0 0 0 0 0 0 0 0 0 0 0 0 0 0 0 0 0 0 0 0 0 0 0 0 0 0 0 0 0 0 0 0 0 0 0

K01970 0 0 0 0 0 0 0 0 0 0 0 0 0 0 0 0 0 0 0 0 0 0 0 0 0 0 0 0 0 0 0 0 0 0 0 0 0 0 0 0 0 0 0 0 0 0 0 0

K01971 0 0 0 0 24 16 0 9 0 0 0 0 0 0 0 0 0 14 0 0 0 0 0 6 0 0 0 0 0 13 0 0 0 0 0 0 0 0 0 0 0 0 0 0 0 0 0 0

K01972 338 420 344 166 406 320 267 564 552 574 661 728 268 601 372 202 508 856 526 476 648 784 838 582 354 338 346 218 292 502 24 648 436 638 772 654 212 438 429 340 452 498 327 580 572 350 470 533

K01973 0 0 0 0 0 0 0 0 0 0 0 0 0 0 0 0 0 0 0 0 0 0 0 0 0 0 0 0 0 0 0 0 0 0 0 0 0 0 0 0 0 0 0 0 0 0 0 0

K01974 88 264 194 49 319 278 72 317 132 120 286 154 91 253 240 92 310 318 164 303 334 192 373 310 108 102 105 81 128 430 0 340 162 164 238 203 58 266 397 206 332 340 154 488 494 165 258 371

K01975 478 724 645 262 978 798 404 1219 768 818 1142 968 386 926 719 320 919 1382 748 822 1088 1127 1335 1064 473 471 472 308 458 1206 24 1038 648 866 1094 934 308 759 916 668 952 984 510 1170 1161 658 788 1134

K01976 0 0 0 0 0 0 0 0 0 0 0 0 0 0 0 0 0 0 0 0 0 0 0 0 0 0 0 0 0 0 0 0 0 0 0 0 0 0 0 0 0 0 0 0 0 0 0 0

K01989 0 0 0 0 0 0 0 0 0 0 0 0 0 0 0 0 0 0 0 0 0 0 0 0 0 0 0 0 0 0 0 0 0 0 0 0 0 0 0 0 0 0 0 0 0 0 0 0

K01990 1297 2274 1807 710 2392 2002 1078 2835 2200 2226 3113 2842 1176 2728 1991 932 2700 3788 2244 2514 3220 3104 3908 2904 1480 1430 1455 926 1394 3117 72 3174 1884 2422 3258 2759 878 2379 2797 1811 2567 2784 1552 3562 3601 1672 2387 3013

K01991 0 0 0 0 0 0 0 0 0 0 0 0 0 0 0 0 0 0 0 0 0 0 0 0 0 0 0 0 0 0 0 0 0 0 0 0 0 0 0 0 0 0 0 0 0 0 0 0

K01992 830 1301 1056 420 1550 1240 652 1792 1370 1358 1754 1756 671 1642 1189 548 1549 2358 1250 1474 1864 1838 2242 1704 870 784 827 589 770 1900 48 1956 1204 1582 1996 1661 491 1234 1506 1073 1556 1631 973 2032 1956 946 1396 1677

K01993 0 0 0 0 0 0 0 0 0 0 0 0 0 0 0 0 0 0 0 0 0 0 0 0 0 0 0 0 0 0 0 0 0 0 0 0 0 0 0 0 0 0 0 0 0 0 0 0

K01994 0 0 0 0 0 0 0 0 0 0 0 0 0 0 0 0 0 0 0 0 0 0 0 0 0 0 0 0 0 0 0 0 0 0 0 0 0 0 0 0 0 0 0 0 0 0 0 0

K01995 708 904 723 333 942 756 534 1290 1130 1148 1383 1457 535 1324 808 450 1092 1807 1051 1030 1396 1604 1816 1228 730 677 704 499 596 1174 48 1441 966 1349 1602 1375 424 908 960 729 1034 1085 729 1310 1255 739 1016 1194

K01996 708 904 723 333 942 756 534 1290 1130 1148 1383 1457 535 1324 808 450 1092 1807 1051 1030 1396 1604 1816 1228 730 677 704 499 596 1174 48 1441 966 1349 1602 1375 424 908 960 729 1034 1085 729 1310 1255 739 1016 1194

K01997 708 904 723 333 942 756 534 1290 1130 1148 1383 1457 535 1324 808 450 1092 1807 1051 1030 1396 1604 1816 1228 730 677 704 499 596 1174 48 1441 966 1349 1602 1375 424 908 960 729 1034 1085 729 1310 1255 739 1016 1194

K01998 708 904 723 333 942 756 534 1290 1130 1148 1383 1457 535 1324 808 450 1092 1807 1051 1030 1396 1604 1816 1228 730 677 704 499 596 1174 48 1441 966 1349 1602 1375 424 908 960 729 1034 1085 729 1310 1255 739 1016 1194

K01999 708 904 723 333 942 756 534 1290 1130 1148 1386 1457 535 1324 808 450 1092 1807 1051 1030 1396 1604 1816 1245 730 677 704 499 596 1178 48 1468 966 1349 1602 1375 424 908 960 729 1034 1085 729 1310 1255 739 1016 1194

K02000 0 0 0 0 23 16 0 9 0 0 0 0 0 0 0 0 0 14 0 0 0 0 0 23 0 0 0 0 0 18 0 30 0 0 0 0 0 0 0 0 0 0 0 0 0 0 0 0

K02001 0 0 0 0 23 16 0 9 0 0 0 0 0 0 0 0 0 14 0 0 0 0 0 23 0 0 0 0 0 18 0 30 0 0 0 0 0 0 0 0 0 0 0 0 0 0 0 0

K02002 0 0 0 0 23 16 0 9 0 0 3 0 0 0 0 0 0 14 0 0 0 0 0 23 0 0 0 0 0 18 0 30 0 0 0 0 0 0 0 0 0 0 0 0 0 0 0 0

K02003 982 1446 1396 587 2144 1742 875 2662 1636 1786 2458 2062 810 1838 1494 628 1876 2933 1568 1620 2222 2386 2694 2286 944 976 960 573 960 2599 48 2060 1276 1719 2257 1930 660 1572 1842 1425 1956 2056 1000 2317 2330 1423 1576 2430

K02004 941 1246 1237 538 2007 1610 811 2526 1530 1667 2231 1908 720 1706 1335 582 1642 2738 1404 1416 1987 2230 2460 2083 860 874 867 554 844 2369 48 1903 1208 1652 2075 1792 602 1336 1548 1268 1755 1812 920 1980 1949 1298 1392 2186

K02005 338 420 344 166 406 320 267 564 552 574 661 728 268 601 372 202 508 856 526 476 648 784 838 582 354 338 346 218 292 502 24 648 436 638 772 654 212 438 429 340 452 498 327 580 572 350 470 533

K02006 948 1377 1308 532 2106 1667 828 2682 1535 1679 2255 1931 736 1788 1470 616 1828 2830 1404 1546 2100 2255 2534 2136 884 865 874 598 850 2531 48 2053 1310 1730 2160 1793 585 1338 1727 1407 2030 1994 1001 2292 2195 1370 1541 2254

K02007 573 764 752 310 1266 1013 484 1566 852 942 1336 1054 414 998 858 348 1020 1604 808 922 1193 1278 1458 1302 484 501 493 318 496 1502 24 1146 699 1003 1177 1012 344 814 1005 790 1120 1146 538 1273 1257 802 847 1365

K02008 630 980 998 380 1772 1408 573 2168 1027 1131 1635 1243 478 1222 1126 421 1332 2029 890 1100 1492 1488 1735 1620 540 531 536 388 574 2112 24 1481 896 1114 1430 1188 382 929 1322 1083 1593 1536 702 1736 1648 1024 1086 1778

K02009 174 326 335 111 626 522 158 696 260 270 519 280 128 360 392 126 422 568 234 395 479 360 536 538 130 137 134 99 182 769 0 436 235 274 364 330 104 350 510 344 515 534 210 615 614 312 334 659

K02010 0 0 0 0 0 0 0 0 0 0 0 0 0 0 0 0 0 0 0 0 0 0 0 0 0 0 0 0 0 0 0 0 0 0 0 0 0 0 0 0 0 0 0 0 0 0 0 0

K02011 0 0 0 0 0 0 0 0 0 0 0 0 0 0 0 0 0 0 0 0 0 0 0 0 0 0 0 0 0 0 0 0 0 0 0 0 0 0 0 0 0 0 0 0 0 0 0 0

K02012 0 0 0 0 0 0 0 0 0 0 0 0 0 0 0 0 0 0 0 0 0 0 0 0 0 0 0 0 0 0 0 0 0 0 0 0 0 0 0 0 0 0 0 0 0 0 0 0

K02013 423 524 486 214 930 730 331 1120 662 698 930 814 296 794 544 275 685 1243 584 599 853 970 1102 940 388 368 378 290 342 1046 24 994 580 774 912 798 249 524 620 511 750 732 430 833 780 533 604 891

K02014 676 839 688 333 811 641 534 1127 1103 1148 1322 1457 535 1202 744 403 1017 1712 1051 951 1297 1569 1677 1163 707 677 692 437 585 1004 48 1295 872 1276 1545 1309 424 877 858 680 904 997 654 1159 1143 699 941 1066

K02015 423 524 486 214 906 714 331 1110 662 698 927 814 296 794 544 275 685 1229 584 599 853 970 1102 916 388 368 378 290 342 1028 24 964 580 774 912 798 249 524 620 511 750 732 430 833 780 533 604 891

K02016 780 966 864 395 1431 1128 610 1741 1257 1298 1635 1583 572 1430 944 483 1206 2168 1122 1104 1542 1772 1980 1592 752 712 732 517 651 1644 48 1710 1039 1434 1727 1502 470 992 1074 867 1218 1270 785 1437 1376 886 1090 1482

K02017 75 222 193 64 282 240 85 212 149 146 269 194 100 166 204 52 246 292 176 253 274 174 273 316 95 107 101 28 134 356 0 274 90 112 224 186 67 266 319 173 216 292 108 362 406 128 200 301

K02018 470 542 559 246 1047 820 391 1382 702 797 1073 859 314 832 638 296 774 1354 632 632 918 1104 1186 1008 390 394 392 290 362 1190 24 909 608 842 952 825 278 548 686 617 903 845 430 912 851 673 647 1064

K02019 66 40 107 47 258 200 72 338 84 124 192 86 28 72 124 27 101 208 59 62 106 150 124 186 12 30 21 9 38 274 0 50 50 88 83 77 37 54 90 122 168 154 28 103 96 144 59 230

K02020 404 460 451 214 734 568 340 929 636 698 858 814 296 673 496 228 610 1106 584 538 754 935 962 819 365 368 367 228 330 824 24 758 486 726 856 732 249 493 518 462 620 652 355 682 668 493 530 764

K02021 0 0 0 0 0 0 0 0 0 0 0 0 0 0 0 0 0 0 0 0 0 0 0 0 0 0 0 0 0 0 0 0 0 0 0 0 0 0 0 0 0 0 0 0 0 0 0 0

K02022 0 0 0 0 0 0 0 0 0 0 0 0 0 0 0 0 0 0 0 0 0 0 0 0 0 0 0 0 0 0 0 0 0 0 0 0 0 0 0 0 0 0 0 0 0 0 0 0

K02023 42 200 159 49 158 147 64 144 106 120 225 154 91 132 160 46 234 210 164 205 234 156 234 208 84 102 94 19 116 243 0 164 68 67 182 137 58 236 295 157 201 244 80 337 382 125 184 244

K02024 0 0 0 0 0 0 0 0 0 0 0 0 0 0 0 0 0 0 0 0 0 0 0 0 0 0 0 0 0 0 0 0 0 0 0 0 0 0 0 0 0 0 0 0 0 0 0 0

K02025 370 595 484 190 610 484 292 808 643 638 760 832 303 752 532 248 719 1044 549 647 842 844 992 681 398 338 368 280 333 802 24 869 583 734 943 754 212 500 656 511 758 744 464 940 866 428 651 716

K02026 412 684 538 216 695 582 331 872 684 694 947 882 358 854 596 294 818 1160 690 760 982 976 1212 855 462 441 451 300 420 914 24 958 598 778 1011 858 270 704 826 546 784 830 482 1067 1065 514 729 904

K02027 370 484 379 166 536 435 267 727 578 574 722 728 268 722 436 248 584 951 526 555 748 820 978 646 377 338 358 280 304 672 24 794 530 711 829 720 212 469 531 389 582 586 402 730 684 390 545 660

K02028 94 240 266 96 434 362 128 492 190 244 420 240 119 204 267 72 335 432 223 249 340 307 357 404 96 132 114 28 154 534 0 248 118 130 265 214 96 290 384 279 369 389 108 440 478 268 243 474

K02029 432 770 714 286 912 732 420 1137 806 882 1119 1072 422 834 736 274 978 1381 772 816 1082 1116 1209 1020 470 471 471 246 476 1168 24 968 608 792 1152 902 308 759 939 741 996 1044 496 1230 1232 657 820 1062

K02030 465 678 683 294 1044 837 446 1354 782 916 1234 1013 405 842 718 294 932 1454 796 739 1054 1224 1280 1115 450 496 474 246 468 1246 24 898 582 811 1078 896 336 753 879 725 974 992 434 1098 1120 758 756 1180

K02031 572 1118 1016 367 1516 1246 535 1898 1005 1100 1622 1271 532 1246 1102 433 1452 1934 984 1195 1580 1476 1806 1482 603 599 601 390 636 1946 24 1426 892 1047 1487 1198 394 1080 1504 1102 1610 1570 726 1946 1909 1002 1196 1734

K02032 951 1626 1415 559 2006 1664 842 2524 1597 1730 2470 2050 854 1948 1536 680 2060 2906 1650 1784 2370 2393 2864 2238 1020 1040 1030 627 1016 2560 48 2162 1342 1729 2328 1956 665 1724 2102 1477 2088 2155 1070 2652 2679 1438 1744 2455

K02033 910 1537 1360 534 1922 1566 802 2462 1556 1674 2283 2000 799 1847 1474 634 1961 2790 1509 1670 2229 2261 2644 2064 956 938 947 608 928 2448 48 2074 1328 1685 2260 1853 606 1518 1932 1442 2062 2068 1052 2526 2480 1352 1666 2266

K02034 910 1537 1360 534 1922 1566 802 2462 1556 1674 2283 2000 799 1847 1474 634 1961 2790 1509 1670 2229 2261 2644 2064 956 938 947 608 928 2448 48 2074 1328 1685 2260 1853 606 1518 1932 1442 2062 2068 1052 2526 2480 1352 1666 2266

K02035 910 1426 1256 510 1848 1518 778 2380 1492 1610 2245 1896 764 1817 1377 634 1826 2696 1486 1578 2135 2236 2630 2029 936 938 936 608 899 2316 48 1998 1274 1662 2146 1820 606 1488 1807 1320 1888 1911 991 2316 2298 1312 1560 2212

K02036 404 506 413 182 614 496 288 776 622 600 763 769 277 758 481 254 596 1006 537 603 788 838 1018 725 388 343 365 290 320 754 24 869 552 756 872 770 220 499 555 405 598 635 430 754 708 393 561 718

K02037 404 506 413 182 591 480 288 767 622 600 760 769 277 758 481 254 596 992 537 603 788 838 1018 702 388 343 365 290 320 736 24 839 552 756 872 770 220 499 555 405 598 635 430 754 708 393 561 718

K02038 404 506 413 182 614 496 288 776 622 600 763 769 277 758 481 254 596 1006 537 603 788 838 1018 725 388 343 365 290 320 754 24 869 552 756 872 770 220 499 555 405 598 635 430 754 708 393 561 718

K02039 484 546 520 228 879 696 369 1114 706 724 955 854 305 830 620 282 697 1214 596 684 893 988 1141 924 399 373 386 298 358 1028 24 920 603 869 954 847 258 554 644 527 766 797 458 858 804 536 620 948

K02040 418 506 413 182 622 496 296 776 622 600 766 769 277 758 497 254 596 1006 537 622 788 838 1018 738 388 343 365 290 320 754 24 872 552 781 872 770 220 499 555 405 598 644 430 754 708 393 561 718

K02041 0 0 0 0 0 0 0 0 0 0 0 0 0 0 0 0 0 0 0 0 0 0 0 0 0 0 0 0 0 0 0 0 0 0 0 0 0 0 0 0 0 0 0 0 0 0 0 0

K02042 0 0 0 0 0 0 0 0 0 0 0 0 0 0 0 0 0 0 0 0 0 0 0 0 0 0 0 0 0 0 0 0 0 0 0 0 0 0 0 0 0 0 0 0 0 0 0 0

K02043 0 0 0 0 0 0 0 0 0 0 0 0 0 0 0 0 0 0 0 0 0 0 0 0 0 0 0 0 0 0 0 0 0 0 0 0 0 0 0 0 0 0 0 0 0 0 0 0

K02044 338 420 344 166 406 320 267 564 552 574 661 728 268 601 372 202 508 856 526 476 648 784 838 582 354 338 346 218 292 502 24 648 436 638 772 654 212 438 429 340 452 498 327 580 572 350 470 533

K02045 0 0 0 0 0 0 0 0 0 0 0 0 0 0 0 0 0 0 0 0 0 0 0 0 0 0 0 0 0 0 0 0 0 0 0 0 0 0 0 0 0 0 0 0 0 0 0 0

K02046 0 0 0 0 0 0 0 0 0 0 0 0 0 0 0 0 0 0 0 0 0 0 0 0 0 0 0 0 0 0 0 0 0 0 0 0 0 0 0 0 0 0 0 0 0 0 0 0

K02047 0 0 0 0 0 0 0 0 0 0 0 0 0 0 0 0 0 0 0 0 0 0 0 0 0 0 0 0 0 0 0 0 0 0 0 0 0 0 0 0 0 0 0 0 0 0 0 0

K02048 0 0 0 0 0 0 0 0 0 0 0 0 0 0 0 0 0 0 0 0 0 0 0 0 0 0 0 0 0 0 0 0 0 0 0 0 0 0 0 0 0 0 0 0 0 0 0 0

K02049 423 524 486 214 814 650 331 1074 662 698 917 814 296 794 544 275 685 1173 584 599 853 970 1102 842 388 368 378 290 342 963 24 874 580 774 912 798 249 524 620 511 750 732 430 833 780 533 604 891

K02050 423 524 486 214 814 650 331 1074 662 698 917 814 296 794 544 275 685 1173 584 599 853 970 1102 842 388 368 378 290 342 963 24 874 580 774 912 798 249 524 620 511 750 732 430 833 780 533 604 891

K02051 423 524 486 214 814 650 331 1074 662 698 917 814 296 794 544 275 685 1173 584 599 853 970 1102 842 388 368 378 290 342 963 24 870 580 774 912 798 249 524 620 511 750 732 430 833 780 533 604 891

K02052 0 0 0 0 0 0 0 0 0 0 0 0 0 0 0 0 0 0 0 0 0 0 0 0 0 0 0 0 0 0 0 0 0 0 0 0 0 0 0 0 0 0 0 0 0 0 0 0

K02053 0 0 0 0 0 0 0 0 0 0 0 0 0 0 0 0 0 0 0 0 0 0 0 0 0 0 0 0 0 0 0 0 0 0 0 0 0 0 0 0 0 0 0 0 0 0 0 0

K02054 0 0 0 0 0 0 0 0 0 0 0 0 0 0 0 0 0 0 0 0 0 0 0 0 0 0 0 0 0 0 0 0 0 0 0 0 0 0 0 0 0 0 0 0 0 0 0 0

K02055 0 0 0 0 0 0 0 0 0 0 0 0 0 0 0 0 0 0 0 0 0 0 0 0 0 0 0 0 0 0 0 0 0 0 0 0 0 0 0 0 0 0 0 0 0 0 0 0

K02056 370 484 379 166 536 435 267 727 578 574 722 728 268 722 436 248 584 951 526 555 748 820 978 646 377 338 358 280 304 672 24 794 530 711 829 720 212 469 531 389 582 586 402 730 684 390 545 660

K02057 370 484 379 166 536 435 267 727 578 574 722 728 268 722 436 248 584 951 526 555 748 820 978 646 377 338 358 280 304 672 24 794 530 711 829 720 212 469 531 389 582 586 402 730 684 390 545 660

K02058 0 0 0 0 0 0 0 0 0 0 0 0 0 0 0 0 0 0 0 0 0 0 0 0 0 0 0 0 0 0 0 0 0 0 0 0 0 0 0 0 0 0 0 0 0 0 0 0

K02059 46 64 35 0 160 130 8 172 26 0 61 0 0 122 80 46 76 109 0 98 99 36 140 84 24 0 12 62 11 182 0 146 94 98 56 66 0 30 102 49 130 96 75 150 112 40 74 128

K02060 74 264 194 49 312 278 64 317 132 120 289 154 91 253 224 92 310 318 164 284 334 192 373 296 108 102 105 81 128 430 0 340 162 140 238 203 58 266 397 206 332 332 154 488 494 165 258 371

K02061 74 264 194 49 312 278 64 317 132 120 289 154 91 253 224 92 310 318 164 284 334 192 373 296 108 102 105 81 128 430 0 344 162 140 238 203 58 266 397 206 332 332 154 488 494 165 258 371

K02062 0 0 0 0 0 0 0 0 0 0 0 0 0 0 0 0 0 0 0 0 0 0 0 0 0 0 0 0 0 0 0 0 0 0 0 0 0 0 0 0 0 0 0 0 0 0 0 0

K02063 0 0 0 0 0 0 0 0 0 0 0 0 0 0 0 0 0 0 0 0 0 0 0 0 0 0 0 0 0 0 0 0 0 0 0 0 0 0 0 0 0 0 0 0 0 0 0 0

K02064 0 0 0 0 0 0 0 0 0 0 0 0 0 0 0 0 0 0 0 0 0 0 0 0 0 0 0 0 0 0 0 0 0 0 0 0 0 0 0 0 0 0 0 0 0 0 0 0

K02065 338 420 344 166 406 320 267 564 552 574 661 728 268 601 372 202 508 856 526 476 648 784 838 582 354 338 346 218 292 502 24 648 436 638 772 654 212 438 429 340 452 498 327 580 572 350 470 533

K02066 338 420 344 166 406 320 267 564 552 574 661 728 268 601 372 202 508 856 526 476 648 784 838 582 354 338 346 218 292 502 24 648 436 638 772 654 212 438 429 340 452 498 327 580 572 350 470 533

K02067 338 420 344 166 406 320 267 564 552 574 661 728 268 601 372 202 508 856 526 476 648 784 838 582 354 338 346 218 292 502 24 648 436 638 772 654 212 438 429 340 452 498 327 580 572 350 470 533

K02068 0 0 0 0 0 0 0 0 0 0 0 0 0 0 0 0 0 0 0 0 0 0 0 0 0 0 0 0 0 0 0 0 0 0 0 0 0 0 0 0 0 0 0 0 0 0 0 0

K02069 0 0 0 0 0 0 0 0 0 0 0 0 0 0 0 0 0 0 0 0 0 0 0 0 0 0 0 0 0 0 0 0 0 0 0 0 0 0 0 0 0 0 0 0 0 0 0 0

K02071 0 0 0 0 0 0 0 0 0 0 0 0 0 0 0 0 0 0 0 0 0 0 0 0 0 0 0 0 0 0 0 0 0 0 0 0 0 0 0 0 0 0 0 0 0 0 0 0

K02072 0 0 0 0 0 0 0 0 0 0 0 0 0 0 0 0 0 0 0 0 0 0 0 0 0 0 0 0 0 0 0 0 0 0 0 0 0 0 0 0 0 0 0 0 0 0 0 0

K02073 0 0 0 0 0 0 0 0 0 0 0 0 0 0 0 0 0 0 0 0 0 0 0 0 0 0 0 0 0 0 0 0 0 0 0 0 0 0 0 0 0 0 0 0 0 0 0 0

K02074 42 200 159 49 160 147 64 144 106 120 228 154 91 132 160 46 234 210 164 205 234 156 234 208 84 102 94 19 116 243 0 164 68 67 182 137 58 236 295 157 201 244 80 337 382 125 184 244

K02075 42 200 159 49 160 147 64 144 106 120 228 154 91 132 160 46 234 210 164 205 234 156 234 208 84 102 94 19 116 243 0 164 68 67 182 137 58 236 295 157 201 244 80 337 382 125 184 244

K02076 0 0 0 0 0 0 0 0 0 0 0 0 0 0 0 0 0 0 0 0 0 0 0 0 0 0 0 0 0 0 0 0 0 0 0 0 0 0 0 0 0 0 0 0 0 0 0 0

K02077 42 200 159 49 160 147 64 144 106 120 228 154 91 132 160 46 234 210 164 205 234 156 234 208 84 102 94 19 116 243 0 164 68 67 182 137 58 236 295 157 201 244 80 337 382 125 184 244

K02078 338 420 344 166 406 320 267 564 552 574 664 728 268 601 372 202 508 856 526 476 648 784 838 582 354 338 346 218 292 502 24 648 436 638 772 654 212 438 429 340 452 498 327 580 572 350 470 533

K02079 0 0 0 0 0 0 0 0 0 0 0 0 0 0 0 0 0 0 0 0 0 0 0 0 0 0 0 0 0 0 0 0 0 0 0 0 0 0 0 0 0 0 0 0 0 0 0 0

K02080 0 0 0 0 0 0 0 0 0 0 0 0 0 0 0 0 0 0 0 0 0 0 0 0 0 0 0 0 0 0 0 0 0 0 0 0 0 0 0 0 0 0 0 0 0 0 0 0

K02081 0 0 0 0 0 0 0 0 0 0 0 0 0 0 0 0 0 0 0 0 0 0 0 0 0 0 0 0 0 0 0 0 0 0 0 0 0 0 0 0 0 0 0 0 0 0 0 0

K02082 0 0 0 0 0 0 0 0 0 0 0 0 0 0 0 0 0 0 0 0 0 0 0 0 0 0 0 0 0 0 0 0 0 0 0 0 0 0 0 0 0 0 0 0 0 0 0 0

K02083 0 0 0 0 0 0 0 0 0 0 0 0 0 0 0 0 0 0 0 0 0 0 0 0 0 0 0 0 0 0 0 0 0 0 0 0 0 0 0 0 0 0 0 0 0 0 0 0

K02086 0 0 0 0 0 0 0 0 0 0 0 0 0 0 0 0 0 0 0 0 0 0 0 0 0 0 0 0 0 0 0 0 0 0 0 0 0 0 0 0 0 0 0 0 0 0 0 0

K02092 0 0 0 0 0 0 0 0 0 0 0 0 0 0 0 0 0 0 0 0 0 0 0 0 0 0 0 0 0 0 0 0 0 0 0 0 0 0 0 0 0 0 0 0 0 0 0 0

K02093 0 0 0 0 0 0 0 0 0 0 0 0 0 0 0 0 0 0 0 0 0 0 0 0 0 0 0 0 0 0 0 0 0 0 0 0 0 0 0 0 0 0 0 0 0 0 0 0

K02094 0 0 0 0 0 0 0 0 0 0 0 0 0 0 0 0 0 0 0 0 0 0 0 0 0 0 0 0 0 0 0 0 0 0 0 0 0 0 0 0 0 0 0 0 0 0 0 0

K02095 0 0 0 0 0 0 0 0 0 0 0 0 0 0 0 0 0 0 0 0 0 0 0 0 0 0 0 0 0 0 0 0 0 0 0 0 0 0 0 0 0 0 0 0 0 0 0 0

K02096 0 0 0 0 0 0 0 0 0 0 0 0 0 0 0 0 0 0 0 0 0 0 0 0 0 0 0 0 0 0 0 0 0 0 0 0 0 0 0 0 0 0 0 0 0 0 0 0

K02097 0 0 0 0 0 0 0 0 0 0 0 0 0 0 0 0 0 0 0 0 0 0 0 0 0 0 0 0 0 0 0 0 0 0 0 0 0 0 0 0 0 0 0 0 0 0 0 0

K02099 0 0 0 0 0 0 0 0 0 0 0 0 0 0 0 0 0 0 0 0 0 0 0 0 0 0 0 0 0 0 0 0 0 0 0 0 0 0 0 0 0 0 0 0 0 0 0 0

K02100 0 0 0 0 0 0 0 0 0 0 0 0 0 0 0 0 0 0 0 0 0 0 0 0 0 0 0 0 0 0 0 0 0 0 0 0 0 0 0 0 0 0 0 0 0 0 0 0

K02101 0 0 0 0 0 0 0 0 0 0 0 0 0 0 0 0 0 0 0 0 0 0 0 0 0 0 0 0 0 0 0 0 0 0 0 0 0 0 0 0 0 0 0 0 0 0 0 0

K02102 0 0 0 0 0 0 0 0 0 0 0 0 0 0 0 0 0 0 0 0 0 0 0 0 0 0 0 0 0 0 0 0 0 0 0 0 0 0 0 0 0 0 0 0 0 0 0 0

K02103 0 0 0 0 0 0 0 0 0 0 0 0 0 0 0 0 0 0 0 0 0 0 0 0 0 0 0 0 0 0 0 0 0 0 0 0 0 0 0 0 0 0 0 0 0 0 0 0

K02106 0 0 0 0 0 0 0 0 0 0 0 0 0 0 0 0 0 0 0 0 0 0 0 0 0 0 0 0 0 0 0 0 0 0 0 0 0 0 0 0 0 0 0 0 0 0 0 0

K02108 338 420 344 166 406 320 267 564 552 574 661 728 268 601 372 202 508 856 526 476 648 784 838 582 354 338 346 218 292 502 24 648 436 638 772 654 212 438 429 340 452 498 327 580 572 350 470 533

K02109 338 420 344 166 406 320 267 564 552 574 661 728 268 601 372 202 508 856 526 476 648 784 838 582 354 338 346 218 292 502 24 648 436 638 772 654 212 438 429 340 452 498 327 580 572 350 470 533

K02110 338 420 344 166 406 320 267 564 552 574 661 728 268 601 372 202 508 856 526 476 648 784 838 582 354 338 346 218 292 502 24 648 436 638 772 654 212 438 429 340 452 498 327 580 572 350 470 533

K02111 338 420 344 166 406 320 267 564 552 574 661 728 268 601 372 202 508 856 526 476 648 784 838 582 354 338 346 218 292 502 24 648 436 638 772 654 212 438 429 340 452 498 327 580 572 350 470 533

K02112 338 420 344 166 406 320 267 564 552 574 661 728 268 601 372 202 508 856 526 476 648 784 838 582 354 338 346 218 292 502 24 648 436 638 772 654 212 438 429 340 452 498 327 580 572 350 470 533

K02113 0 0 0 0 0 0 0 0 0 0 0 0 0 0 0 0 0 0 0 0 0 0 0 0 0 0 0 0 0 0 0 0 0 0 0 0 0 0 0 0 0 0 0 0 0 0 0 0

K02114 338 420 344 166 406 320 267 564 552 574 661 728 268 601 372 202 508 856 526 476 648 784 838 582 354 338 346 218 292 502 24 648 436 638 772 654 212 438 429 340 452 498 327 580 572 350 470 533

K02115 338 420 344 166 406 320 267 564 552 574 661 728 268 601 372 202 508 856 526 476 648 784 838 582 354 338 346 218 292 502 24 648 436 638 772 654 212 438 429 340 452 498 327 580 572 350 470 533

K02116 0 0 0 0 0 0 0 0 0 0 0 0 0 0 0 0 0 0 0 0 0 0 0 0 0 0 0 0 0 0 0 0 0 0 0 0 0 0 0 0 0 0 0 0 0 0 0 0

K02117 478 724 645 262 978 798 404 1219 768 818 1144 968 386 926 719 320 919 1382 748 822 1088 1127 1335 1064 473 471 472 308 458 1206 24 1042 648 866 1094 934 308 759 916 668 952 984 510 1170 1161 658 788 1134

K02118 478 724 645 262 978 798 404 1219 768 818 1144 968 386 926 719 320 919 1382 748 822 1088 1127 1335 1064 473 471 472 308 458 1206 24 1042 648 866 1094 934 308 759 916 668 952 984 510 1170 1161 658 788 1134

K02119 140 304 301 96 595 493 136 664 216 244 483 240 119 325 347 119 410 540 223 347 439 342 496 488 120 132 126 90 166 717 0 391 212 228 322 280 96 320 486 328 500 486 182 590 590 308 318 602

K02120 478 724 645 262 1000 814 404 1228 768 818 1144 968 386 926 719 320 919 1396 748 822 1088 1127 1335 1086 473 471 472 308 458 1224 24 1068 648 866 1094 934 308 759 916 668 952 984 510 1170 1161 658 788 1134

K02121 619 1028 946 358 1550 1274 540 1874 984 1062 1625 1208 506 1251 1066 440 1330 1909 972 1170 1526 1470 1832 1546 592 604 598 398 624 1910 24 1430 861 1094 1416 1214 403 1080 1402 996 1451 1470 692 1760 1750 966 1106 1736

K02122 140 304 301 96 572 477 136 656 216 244 483 240 119 325 347 119 410 526 223 347 439 342 496 482 120 132 126 90 166 704 0 391 212 228 322 280 96 320 486 328 500 486 182 590 590 308 318 602

K02123 478 724 645 262 978 798 404 1219 768 818 1144 968 386 926 719 320 919 1382 748 822 1088 1127 1335 1064 473 471 472 308 458 1206 24 1042 648 866 1094 934 308 759 916 668 952 984 510 1170 1161 658 788 1134

K02124 437 524 486 214 819 650 340 1074 662 698 919 814 296 794 560 275 685 1173 584 618 853 970 1102 855 388 368 378 290 342 963 24 874 580 799 912 798 249 524 620 511 750 740 430 833 780 533 604 891

K02132 0 0 0 0 0 0 0 0 0 0 0 0 0 0 0 0 0 0 0 0 0 0 0 0 0 0 0 0 0 0 0 0 0 0 0 0 0 0 0 0 0 0 0 0 0 0 0 0

K02137 0 0 0 0 0 0 0 0 0 0 0 0 0 0 0 0 0 0 0 0 0 0 0 0 0 0 0 0 0 0 0 0 0 0 0 0 0 0 0 0 0 0 0 0 0 0 0 0

K02160 0 0 0 0 0 0 0 0 0 0 0 0 0 0 0 0 0 0 0 0 0 0 0 0 0 0 0 0 0 0 0 0 0 0 0 0 0 0 0 0 0 0 0 0 0 0 0 0

K02164 0 0 0 0 0 0 0 0 0 0 0 0 0 0 0 0 0 0 0 0 0 0 0 0 0 0 0 0 0 0 0 0 0 0 0 0 0 0 0 0 0 0 0 0 0 0 0 0

K02167 0 0 0 0 0 0 0 0 0 0 0 0 0 0 0 0 0 0 0 0 0 0 0 0 0 0 0 0 0 0 0 0 0 0 0 0 0 0 0 0 0 0 0 0 0 0 0 0

K02168 0 0 0 0 0 0 0 0 0 0 0 0 0 0 0 0 0 0 0 0 0 0 0 0 0 0 0 0 0 0 0 0 0 0 0 0 0 0 0 0 0 0 0 0 0 0 0 0

K02169 0 0 0 0 0 0 0 0 0 0 0 0 0 0 0 0 0 0 0 0 0 0 0 0 0 0 0 0 0 0 0 0 0 0 0 0 0 0 0 0 0 0 0 0 0 0 0 0

K02170 0 0 0 0 0 0 0 0 0 0 0 0 0 0 0 0 0 0 0 0 0 0 0 0 0 0 0 0 0 0 0 0 0 0 0 0 0 0 0 0 0 0 0 0 0 0 0 0

K02171 0 0 0 0 0 0 0 0 0 0 0 0 0 0 0 0 0 0 0 0 0 0 0 0 0 0 0 0 0 0 0 0 0 0 0 0 0 0 0 0 0 0 0 0 0 0 0 0

K02172 0 0 0 0 0 0 0 0 0 0 0 0 0 0 0 0 0 0 0 0 0 0 0 0 0 0 0 0 0 0 0 0 0 0 0 0 0 0 0 0 0 0 0 0 0 0 0 0

K02173 0 0 0 0 0 0 0 0 0 0 0 0 0 0 0 0 0 0 0 0 0 0 0 0 0 0 0 0 0 0 0 0 0 0 0 0 0 0 0 0 0 0 0 0 0 0 0 0

K02182 0 0 0 0 0 0 0 0 0 0 0 0 0 0 0 0 0 0 0 0 0 0 0 0 0 0 0 0 0 0 0 0 0 0 0 0 0 0 0 0 0 0 0 0 0 0 0 0

K02183 0 0 0 0 0 0 0 0 0 0 0 0 0 0 0 0 0 0 0 0 0 0 0 0 0 0 0 0 0 0 0 0 0 0 0 0 0 0 0 0 0 0 0 0 0 0 0 0

K02188 66 40 107 47 282 216 72 348 84 124 195 86 28 72 124 27 101 222 59 62 106 150 124 208 12 30 21 9 38 292 0 80 50 88 83 77 37 54 90 122 168 154 28 103 96 144 59 230

K02189 66 40 107 47 282 216 72 348 84 124 195 86 28 72 124 27 101 222 59 62 106 150 124 208 12 30 21 9 38 292 0 80 50 88 83 77 37 54 90 122 168 154 28 103 96 144 59 230

K02190 33 18 73 32 205 154 52 298 40 98 154 45 18 37 78 20 89 167 48 14 66 133 84 130 1 26 13 0 21 209 0 5 28 42 40 28 28 24 66 106 152 105 0 78 72 140 43 173

K02191 66 40 107 47 282 216 72 348 84 124 195 86 28 72 124 27 101 222 59 62 106 150 124 208 12 30 21 9 38 292 0 80 50 88 83 77 37 54 90 122 168 154 28 103 96 144 59 230

K02192 0 0 0 0 0 0 0 0 0 0 0 0 0 0 0 0 0 0 0 0 0 0 0 0 0 0 0 0 0 0 0 0 0 0 0 0 0 0 0 0 0 0 0 0 0 0 0 0

K02193 0 0 0 0 0 0 0 0 0 0 0 0 0 0 0 0 0 0 0 0 0 0 0 0 0 0 0 0 0 0 0 0 0 0 0 0 0 0 0 0 0 0 0 0 0 0 0 0

K02194 0 0 0 0 23 16 0 9 0 0 0 0 0 0 0 0 0 14 0 0 0 0 0 23 0 0 0 0 0 18 0 30 0 0 0 0 0 0 0 0 0 0 0 0 0 0 0 0

K02195 0 0 0 0 0 0 0 0 0 0 0 0 0 0 0 0 0 0 0 0 0 0 0 0 0 0 0 0 0 0 0 0 0 0 0 0 0 0 0 0 0 0 0 0 0 0 0 0

K02196 0 0 0 0 0 0 0 0 0 0 0 0 0 0 0 0 0 0 0 0 0 0 0 0 0 0 0 0 0 0 0 0 0 0 0 0 0 0 0 0 0 0 0 0 0 0 0 0

K02197 0 0 0 0 0 0 0 0 0 0 0 0 0 0 0 0 0 0 0 0 0 0 0 0 0 0 0 0 0 0 0 0 0 0 0 0 0 0 0 0 0 0 0 0 0 0 0 0

K02198 0 0 0 0 0 0 0 0 0 0 0 0 0 0 0 0 0 0 0 0 0 0 0 0 0 0 0 0 0 0 0 0 0 0 0 0 0 0 0 0 0 0 0 0 0 0 0 0

K02199 0 0 0 0 0 0 0 0 0 0 0 0 0 0 0 0 0 0 0 0 0 0 0 0 0 0 0 0 0 0 0 0 0 0 0 0 0 0 0 0 0 0 0 0 0 0 0 0

K02200 0 0 0 0 0 0 0 0 0 0 0 0 0 0 0 0 0 0 0 0 0 0 0 0 0 0 0 0 0 0 0 0 0 0 0 0 0 0 0 0 0 0 0 0 0 0 0 0

K02201 140 304 301 96 572 477 136 656 216 244 481 240 119 325 347 119 410 526 223 347 439 342 496 482 120 132 126 90 166 704 0 391 212 228 322 280 96 320 486 328 500 486 182 590 590 308 318 602

K02203 0 0 0 0 0 0 0 0 0 0 0 0 0 0 0 0 0 0 0 0 0 0 0 0 0 0 0 0 0 0 0 0 0 0 0 0 0 0 0 0 0 0 0 0 0 0 0 0

K02204 0 0 0 0 0 0 0 0 0 0 0 0 0 0 0 0 0 0 0 0 0 0 0 0 0 0 0 0 0 0 0 0 0 0 0 0 0 0 0 0 0 0 0 0 0 0 0 0

K02205 0 0 0 0 0 0 0 0 0 0 0 0 0 0 0 0 0 0 0 0 0 0 0 0 0 0 0 0 0 0 0 0 0 0 0 0 0 0 0 0 0 0 0 0 0 0 0 0

K02217 108 240 266 96 418 346 136 483 190 244 420 240 119 204 283 72 335 418 223 268 340 307 357 411 96 132 114 28 154 522 0 245 118 155 265 214 96 290 384 279 369 398 108 440 478 268 243 474

K02221 0 0 0 0 0 0 0 0 0 0 0 0 0 0 0 0 0 0 0 0 0 0 0 0 0 0 0 0 0 0 0 0 0 0 0 0 0 0 0 0 0 0 0 0 0 0 0 0

K02224 536 582 666 292 1356 1052 464 1738 786 922 1270 944 342 904 762 322 875 1590 691 694 1024 1254 1309 1240 401 424 412 298 400 1498 24 1020 659 930 1036 902 314 602 776 739 1071 998 458 1014 947 816 706 1294

K02225 0 0 0 0 0 0 0 0 0 0 0 0 0 0 0 0 0 0 0 0 0 0 0 0 0 0 0 0 0 0 0 0 0 0 0 0 0 0 0 0 0 0 0 0 0 0 0 0

K02226 0 0 0 0 0 0 0 0 0 0 0 0 0 0 0 0 0 0 0 0 0 0 0 0 0 0 0 0 0 0 0 0 0 0 0 0 0 0 0 0 0 0 0 0 0 0 0 0

K02227 437 524 486 214 819 650 340 1074 662 698 917 814 296 794 560 275 685 1173 584 618 853 970 1102 855 388 368 378 290 342 963 24 874 580 799 912 798 249 524 620 511 750 740 430 833 780 533 604 891

K02228 0 0 0 0 0 0 0 0 0 0 0 0 0 0 0 0 0 0 0 0 0 0 0 0 0 0 0 0 0 0 0 0 0 0 0 0 0 0 0 0 0 0 0 0 0 0 0 0

K02229 0 0 0 0 0 0 0 0 0 0 0 0 0 0 0 0 0 0 0 0 0 0 0 0 0 0 0 0 0 0 0 0 0 0 0 0 0 0 0 0 0 0 0 0 0 0 0 0

K02230 498 588 521 214 989 765 390 1238 688 698 978 814 296 916 720 322 760 1268 584 734 952 1006 1241 1001 412 368 390 352 352 1132 24 1020 674 921 968 864 249 554 722 560 881 879 505 984 892 573 678 1018

K02231 338 420 344 166 406 320 267 564 552 574 661 728 268 601 372 202 508 856 526 476 648 784 838 582 354 338 346 218 292 502 24 648 436 638 772 654 212 438 429 340 452 498 327 580 572 350 470 533

K02232 437 524 486 214 819 650 340 1074 662 698 917 814 296 794 560 275 685 1173 584 618 853 970 1102 855 388 368 378 290 342 963 24 874 580 799 912 798 249 524 620 511 750 740 430 833 780 533 604 891

K02233 437 524 486 214 819 650 340 1074 662 698 917 814 296 794 560 275 685 1173 584 618 853 970 1102 855 388 368 378 290 342 963 24 874 580 799 912 798 249 524 620 511 750 740 430 833 780 533 604 891

K02234 0 0 0 0 0 0 0 0 0 0 0 0 0 0 0 0 0 0 0 0 0 0 0 0 0 0 0 0 0 0 0 0 0 0 0 0 0 0 0 0 0 0 0 0 0 0 0 0

K02236 0 0 0 0 0 0 0 0 0 0 0 0 0 0 0 0 0 0 0 0 0 0 0 0 0 0 0 0 0 0 0 0 0 0 0 0 0 0 0 0 0 0 0 0 0 0 0 0

K02237 0 0 0 0 0 0 0 0 0 0 0 0 0 0 0 0 0 0 0 0 0 0 0 0 0 0 0 0 0 0 0 0 0 0 0 0 0 0 0 0 0 0 0 0 0 0 0 0

K02238 0 0 0 0 23 16 0 9 0 0 3 0 0 0 0 0 0 14 0 0 0 0 0 23 0 0 0 0 0 18 0 26 0 0 0 0 0 0 0 0 0 0 0 0 0 0 0 0

K02239 0 0 0 0 0 0 0 0 0 0 0 0 0 0 0 0 0 0 0 0 0 0 0 0 0 0 0 0 0 0 0 0 0 0 0 0 0 0 0 0 0 0 0 0 0 0 0 0

K02240 0 0 0 0 0 0 0 0 0 0 0 0 0 0 0 0 0 0 0 0 0 0 0 0 0 0 0 0 0 0 0 0 0 0 0 0 0 0 0 0 0 0 0 0 0 0 0 0

K02241 0 0 0 0 0 0 0 0 0 0 0 0 0 0 0 0 0 0 0 0 0 0 0 0 0 0 0 0 0 0 0 0 0 0 0 0 0 0 0 0 0 0 0 0 0 0 0 0

K02242 0 0 0 0 0 0 0 0 0 0 0 0 0 0 0 0 0 0 0 0 0 0 0 0 0 0 0 0 0 0 0 0 0 0 0 0 0 0 0 0 0 0 0 0 0 0 0 0

K02243 0 0 0 0 0 0 0 0 0 0 0 0 0 0 0 0 0 0 0 0 0 0 0 0 0 0 0 0 0 0 0 0 0 0 0 0 0 0 0 0 0 0 0 0 0 0 0 0

K02244 0 0 0 0 0 0 0 0 0 0 0 0 0 0 0 0 0 0 0 0 0 0 0 0 0 0 0 0 0 0 0 0 0 0 0 0 0 0 0 0 0 0 0 0 0 0 0 0

K02245 0 0 0 0 0 0 0 0 0 0 0 0 0 0 0 0 0 0 0 0 0 0 0 0 0 0 0 0 0 0 0 0 0 0 0 0 0 0 0 0 0 0 0 0 0 0 0 0

K02246 0 0 0 0 0 0 0 0 0 0 0 0 0 0 0 0 0 0 0 0 0 0 0 0 0 0 0 0 0 0 0 0 0 0 0 0 0 0 0 0 0 0 0 0 0 0 0 0

K02247 0 0 0 0 0 0 0 0 0 0 0 0 0 0 0 0 0 0 0 0 0 0 0 0 0 0 0 0 0 0 0 0 0 0 0 0 0 0 0 0 0 0 0 0 0 0 0 0

K02248 0 0 0 0 0 0 0 0 0 0 0 0 0 0 0 0 0 0 0 0 0 0 0 0 0 0 0 0 0 0 0 0 0 0 0 0 0 0 0 0 0 0 0 0 0 0 0 0

K02249 0 0 0 0 0 0 0 0 0 0 0 0 0 0 0 0 0 0 0 0 0 0 0 0 0 0 0 0 0 0 0 0 0 0 0 0 0 0 0 0 0 0 0 0 0 0 0 0

K02250 0 0 0 0 0 0 0 0 0 0 0 0 0 0 0 0 0 0 0 0 0 0 0 0 0 0 0 0 0 0 0 0 0 0 0 0 0 0 0 0 0 0 0 0 0 0 0 0

K02251 0 0 0 0 0 0 0 0 0 0 0 0 0 0 0 0 0 0 0 0 0 0 0 0 0 0 0 0 0 0 0 0 0 0 0 0 0 0 0 0 0 0 0 0 0 0 0 0

K02252 0 0 0 0 0 0 0 0 0 0 0 0 0 0 0 0 0 0 0 0 0 0 0 0 0 0 0 0 0 0 0 0 0 0 0 0 0 0 0 0 0 0 0 0 0 0 0 0

K02253 0 0 0 0 0 0 0 0 0 0 0 0 0 0 0 0 0 0 0 0 0 0 0 0 0 0 0 0 0 0 0 0 0 0 0 0 0 0 0 0 0 0 0 0 0 0 0 0

K02254 0 0 0 0 0 0 0 0 0 0 0 0 0 0 0 0 0 0 0 0 0 0 0 0 0 0 0 0 0 0 0 0 0 0 0 0 0 0 0 0 0 0 0 0 0 0 0 0

K02255 0 0 0 0 0 0 0 0 0 0 0 0 0 0 0 0 0 0 0 0 0 0 0 0 0 0 0 0 0 0 0 0 0 0 0 0 0 0 0 0 0 0 0 0 0 0 0 0

K02258 0 0 0 0 0 0 0 0 0 0 0 0 0 0 0 0 0 0 0 0 0 0 0 0 0 0 0 0 0 0 0 0 0 0 0 0 0 0 0 0 0 0 0 0 0 0 0 0

K02259 0 0 0 0 0 0 0 0 0 0 0 0 0 0 0 0 0 0 0 0 0 0 0 0 0 0 0 0 0 0 0 0 0 0 0 0 0 0 0 0 0 0 0 0 0 0 0 0

K02266 0 0 0 0 0 0 0 0 0 0 0 0 0 0 0 0 0 0 0 0 0 0 0 0 0 0 0 0 0 0 0 0 0 0 0 0 0 0 0 0 0 0 0 0 0 0 0 0

K02274 338 420 344 166 406 320 267 564 552 574 661 728 268 601 372 202 508 856 526 476 648 784 838 582 354 338 346 218 292 502 24 648 436 638 772 654 212 438 429 340 452 498 327 580 572 350 470 533

K02275 338 420 344 166 406 320 267 564 552 574 661 728 268 601 372 202 508 856 526 476 648 784 838 582 354 338 346 218 292 502 24 648 436 638 772 654 212 438 429 340 452 498 327 580 572 350 470 533

K02276 0 0 0 0 0 0 0 0 0 0 0 0 0 0 0 0 0 0 0 0 0 0 0 0 0 0 0 0 0 0 0 0 0 0 0 0 0 0 0 0 0 0 0 0 0 0 0 0

K02277 0 0 0 0 0 0 0 0 0 0 0 0 0 0 0 0 0 0 0 0 0 0 0 0 0 0 0 0 0 0 0 0 0 0 0 0 0 0 0 0 0 0 0 0 0 0 0 0

K02278 0 0 0 0 0 0 0 0 0 0 0 0 0 0 0 0 0 0 0 0 0 0 0 0 0 0 0 0 0 0 0 0 0 0 0 0 0 0 0 0 0 0 0 0 0 0 0 0

K02279 0 0 0 0 0 0 0 0 0 0 0 0 0 0 0 0 0 0 0 0 0 0 0 0 0 0 0 0 0 0 0 0 0 0 0 0 0 0 0 0 0 0 0 0 0 0 0 0

K02280 0 0 0 0 0 0 0 0 0 0 0 0 0 0 0 0 0 0 0 0 0 0 0 0 0 0 0 0 0 0 0 0 0 0 0 0 0 0 0 0 0 0 0 0 0 0 0 0

K02281 0 0 0 0 0 0 0 0 0 0 0 0 0 0 0 0 0 0 0 0 0 0 0 0 0 0 0 0 0 0 0 0 0 0 0 0 0 0 0 0 0 0 0 0 0 0 0 0

K02282 0 0 0 0 0 0 0 0 0 0 0 0 0 0 0 0 0 0 0 0 0 0 0 0 0 0 0 0 0 0 0 0 0 0 0 0 0 0 0 0 0 0 0 0 0 0 0 0

K02283 486 902 770 296 1084 899 446 1324 830 912 1331 1082 468 1022 834 360 1141 1551 901 980 1282 1266 1529 1216 547 569 558 318 558 1384 24 1161 694 888 1234 1022 358 964 1186 809 1137 1180 561 1482 1518 780 956 1320

K02284 0 0 0 0 0 0 0 0 0 0 0 0 0 0 0 0 0 0 0 0 0 0 0 0 0 0 0 0 0 0 0 0 0 0 0 0 0 0 0 0 0 0 0 0 0 0 0 0

K02285 0 0 0 0 0 0 0 0 0 0 0 0 0 0 0 0 0 0 0 0 0 0 0 0 0 0 0 0 0 0 0 0 0 0 0 0 0 0 0 0 0 0 0 0 0 0 0 0

K02286 0 0 0 0 0 0 0 0 0 0 0 0 0 0 0 0 0 0 0 0 0 0 0 0 0 0 0 0 0 0 0 0 0 0 0 0 0 0 0 0 0 0 0 0 0 0 0 0

K02287 0 0 0 0 0 0 0 0 0 0 0 0 0 0 0 0 0 0 0 0 0 0 0 0 0 0 0 0 0 0 0 0 0 0 0 0 0 0 0 0 0 0 0 0 0 0 0 0

K02288 0 0 0 0 0 0 0 0 0 0 0 0 0 0 0 0 0 0 0 0 0 0 0 0 0 0 0 0 0 0 0 0 0 0 0 0 0 0 0 0 0 0 0 0 0 0 0 0

K02289 0 0 0 0 0 0 0 0 0 0 0 0 0 0 0 0 0 0 0 0 0 0 0 0 0 0 0 0 0 0 0 0 0 0 0 0 0 0 0 0 0 0 0 0 0 0 0 0

K02290 0 0 0 0 0 0 0 0 0 0 0 0 0 0 0 0 0 0 0 0 0 0 0 0 0 0 0 0 0 0 0 0 0 0 0 0 0 0 0 0 0 0 0 0 0 0 0 0

K02291 14 0 0 0 30 16 8 9 0 0 0 0 0 0 16 0 0 14 0 18 0 0 0 20 0 0 0 0 0 13 0 0 0 24 0 0 0 0 0 0 0 8 0 0 0 0 0 0

K02292 0 0 0 0 0 0 0 0 0 0 0 0 0 0 0 0 0 0 0 0 0 0 0 0 0 0 0 0 0 0 0 0 0 0 0 0 0 0 0 0 0 0 0 0 0 0 0 0

K02293 0 0 0 0 0 0 0 0 0 0 0 0 0 0 0 0 0 0 0 0 0 0 0 0 0 0 0 0 0 0 0 0 0 0 0 0 0 0 0 0 0 0 0 0 0 0 0 0

K02294 0 0 0 0 0 0 0 0 0 0 0 0 0 0 0 0 0 0 0 0 0 0 0 0 0 0 0 0 0 0 0 0 0 0 0 0 0 0 0 0 0 0 0 0 0 0 0 0

K02297 0 0 0 0 0 0 0 0 0 0 0 0 0 0 0 0 0 0 0 0 0 0 0 0 0 0 0 0 0 0 0 0 0 0 0 0 0 0 0 0 0 0 0 0 0 0 0 0

K02298 0 0 0 0 0 0 0 0 0 0 0 0 0 0 0 0 0 0 0 0 0 0 0 0 0 0 0 0 0 0 0 0 0 0 0 0 0 0 0 0 0 0 0 0 0 0 0 0

K02299 0 0 0 0 0 0 0 0 0 0 0 0 0 0 0 0 0 0 0 0 0 0 0 0 0 0 0 0 0 0 0 0 0 0 0 0 0 0 0 0 0 0 0 0 0 0 0 0

K02300 0 0 0 0 0 0 0 0 0 0 0 0 0 0 0 0 0 0 0 0 0 0 0 0 0 0 0 0 0 0 0 0 0 0 0 0 0 0 0 0 0 0 0 0 0 0 0 0

K02301 338 420 344 166 406 320 267 564 552 574 661 728 268 601 372 202 508 856 526 476 648 784 838 582 354 338 346 218 292 502 24 648 436 638 772 654 212 438 429 340 452 498 327 580 572 350 470 533

K02302 0 0 0 0 0 0 0 0 0 0 0 0 0 0 0 0 0 0 0 0 0 0 0 0 0 0 0 0 0 0 0 0 0 0 0 0 0 0 0 0 0 0 0 0 0 0 0 0

K02303 437 524 486 214 819 650 340 1074 662 698 917 814 296 794 560 275 685 1173 584 618 853 970 1102 855 388 368 378 290 342 963 24 874 580 799 912 798 249 524 620 511 750 740 430 833 780 533 604 891

K02304 504 564 593 260 1102 866 412 1422 746 823 1111 900 324 866 683 302 786 1395 644 680 958 1121 1225 1064 400 398 399 298 380 1254 24 954 631 887 995 874 286 578 710 633 918 894 458 936 876 676 663 1122

K02305 0 0 0 0 0 0 0 0 0 0 0 0 0 0 0 0 0 0 0 0 0 0 0 0 0 0 0 0 0 0 0 0 0 0 0 0 0 0 0 0 0 0 0 0 0 0 0 0

K02312 0 0 0 0 0 0 0 0 0 0 0 0 0 0 0 0 0 0 0 0 0 0 0 0 0 0 0 0 0 0 0 0 0 0 0 0 0 0 0 0 0 0 0 0 0 0 0 0

K02313 338 420 344 166 406 320 267 564 552 574 661 728 268 601 372 202 508 856 526 476 648 784 838 582 354 338 346 218 292 502 24 648 436 638 772 654 212 438 429 340 452 498 327 580 572 350 470 533

K02314 338 420 344 166 406 320 267 564 552 574 661 728 268 601 372 202 508 856 526 476 648 784 838 582 354 338 346 218 292 502 24 648 436 638 772 654 212 438 429 340 452 498 327 580 572 350 470 533

K02315 0 0 0 0 0 0 0 0 0 0 0 0 0 0 0 0 0 0 0 0 0 0 0 0 0 0 0 0 0 0 0 0 0 0 0 0 0 0 0 0 0 0 0 0 0 0 0 0

K02316 338 420 344 166 406 320 267 564 552 574 661 728 268 601 372 202 508 856 526 476 648 784 838 582 354 338 346 218 292 502 24 648 436 638 772 654 212 438 429 340 452 498 327 580 572 350 470 533

K02317 0 0 0 0 0 0 0 0 0 0 0 0 0 0 0 0 0 0 0 0 0 0 0 0 0 0 0 0 0 0 0 0 0 0 0 0 0 0 0 0 0 0 0 0 0 0 0 0

K02319 619 1028 946 358 1526 1258 540 1866 984 1062 1622 1208 506 1251 1066 440 1330 1895 972 1170 1526 1470 1832 1522 592 604 598 398 624 1892 24 1400 861 1094 1416 1214 403 1080 1402 996 1451 1470 692 1760 1750 966 1106 1736

K02320 0 0 0 0 0 0 0 0 0 0 0 0 0 0 0 0 0 0 0 0 0 0 0 0 0 0 0 0 0 0 0 0 0 0 0 0 0 0 0 0 0 0 0 0 0 0 0 0

K02322 140 304 301 96 572 477 136 656 216 244 481 240 119 325 347 119 410 526 223 347 439 342 496 482 120 132 126 90 166 704 0 391 212 228 322 280 96 320 486 328 500 486 182 590 590 308 318 602

K02323 140 304 301 96 572 477 136 656 216 244 481 240 119 325 347 119 410 526 223 347 439 342 496 482 120 132 126 90 166 704 0 391 212 228 322 280 96 320 486 328 500 486 182 590 590 308 318 602

K02330 0 0 0 0 0 0 0 0 0 0 0 0 0 0 0 0 0 0 0 0 0 0 0 0 0 0 0 0 0 0 0 0 0 0 0 0 0 0 0 0 0 0 0 0 0 0 0 0

K02334 412 684 538 216 718 598 331 880 684 694 947 882 358 854 596 294 818 1174 690 760 982 976 1212 861 462 441 451 300 420 928 24 958 598 778 1011 858 270 704 826 546 784 830 482 1067 1065 514 729 904

K02335 338 420 344 166 406 320 267 564 552 574 661 728 268 601 372 202 508 856 526 476 648 784 838 582 354 338 346 218 292 502 24 648 436 638 772 654 212 438 429 340 452 498 327 580 572 350 470 533

K02336 0 0 0 0 0 0 0 0 0 0 0 0 0 0 0 0 0 0 0 0 0 0 0 0 0 0 0 0 0 0 0 0 0 0 0 0 0 0 0 0 0 0 0 0 0 0 0 0

K02337 338 420 344 166 406 320 267 564 552 574 661 728 268 601 372 202 508 856 526 476 648 784 838 582 354 338 346 218 292 502 24 648 436 638 772 654 212 438 429 340 452 498 327 580 572 350 470 533

K02338 338 420 344 166 406 320 267 564 552 574 661 728 268 601 372 202 508 856 526 476 648 784 838 582 354 338 346 218 292 502 24 648 436 638 772 654 212 438 429 340 452 498 327 580 572 350 470 533

K02339 0 0 0 0 0 0 0 0 0 0 0 0 0 0 0 0 0 0 0 0 0 0 0 0 0 0 0 0 0 0 0 0 0 0 0 0 0 0 0 0 0 0 0 0 0 0 0 0

K02340 0 0 0 0 0 0 0 0 0 0 0 0 0 0 0 0 0 0 0 0 0 0 0 0 0 0 0 0 0 0 0 0 0 0 0 0 0 0 0 0 0 0 0 0 0 0 0 0

K02341 0 0 0 0 0 0 0 0 0 0 0 0 0 0 0 0 0 0 0 0 0 0 0 0 0 0 0 0 0 0 0 0 0 0 0 0 0 0 0 0 0 0 0 0 0 0 0 0

K02342 338 420 344 166 406 320 267 564 552 574 661 728 268 601 372 202 508 856 526 476 648 784 838 582 354 338 346 218 292 502 24 648 436 638 772 654 212 438 429 340 452 498 327 580 572 350 470 533

K02343 338 420 344 166 406 320 267 564 552 574 661 728 268 601 372 202 508 856 526 476 648 784 838 582 354 338 346 218 292 502 24 648 436 638 772 654 212 438 429 340 452 498 327 580 572 350 470 533

K02344 0 0 0 0 0 0 0 0 0 0 0 0 0 0 0 0 0 0 0 0 0 0 0 0 0 0 0 0 0 0 0 0 0 0 0 0 0 0 0 0 0 0 0 0 0 0 0 0

K02345 0 0 0 0 0 0 0 0 0 0 0 0 0 0 0 0 0 0 0 0 0 0 0 0 0 0 0 0 0 0 0 0 0 0 0 0 0 0 0 0 0 0 0 0 0 0 0 0

K02346 0 0 0 0 0 0 0 0 0 0 0 0 0 0 0 0 0 0 0 0 0 0 0 0 0 0 0 0 0 0 0 0 0 0 0 0 0 0 0 0 0 0 0 0 0 0 0 0

K02347 56 200 159 49 188 163 72 154 106 120 228 154 91 132 176 46 234 224 164 224 234 156 234 245 84 102 94 19 116 260 0 194 68 92 182 137 58 236 295 157 201 252 80 337 382 125 184 244

K02348 0 0 0 0 0 0 0 0 0 0 0 0 0 0 0 0 0 0 0 0 0 0 0 0 0 0 0 0 0 0 0 0 0 0 0 0 0 0 0 0 0 0 0 0 0 0 0 0

K02351 0 0 0 0 0 0 0 0 0 0 0 0 0 0 0 0 0 0 0 0 0 0 0 0 0 0 0 0 0 0 0 0 0 0 0 0 0 0 0 0 0 0 0 0 0 0 0 0

K02352 0 0 0 0 0 0 0 0 0 0 0 0 0 0 0 0 0 0 0 0 0 0 0 0 0 0 0 0 0 0 0 0 0 0 0 0 0 0 0 0 0 0 0 0 0 0 0 0

K02355 338 420 344 166 406 320 267 564 552 574 661 728 268 601 372 202 508 856 526 476 648 784 838 582 354 338 346 218 292 502 24 648 436 638 772 654 212 438 429 340 452 498 327 580 572 350 470 533

K02356 338 420 344 166 406 320 267 564 552 574 661 728 268 601 372 202 508 856 526 476 648 784 838 582 354 338 346 218 292 502 24 648 436 638 772 654 212 438 429 340 452 498 327 580 572 350 470 533

K02357 338 420 344 166 406 320 267 564 552 574 661 728 268 601 372 202 508 856 526 476 648 784 838 582 354 338 346 218 292 502 24 648 436 638 772 654 212 438 429 340 452 498 327 580 572 350 470 533

K02358 338 420 344 166 406 320 267 564 552 574 661 728 268 601 372 202 508 856 526 476 648 784 838 582 354 338 346 218 292 502 24 648 436 638 772 654 212 438 429 340 452 498 327 580 572 350 470 533

K02359 0 0 0 0 0 0 0 0 0 0 0 0 0 0 0 0 0 0 0 0 0 0 0 0 0 0 0 0 0 0 0 0 0 0 0 0 0 0 0 0 0 0 0 0 0 0 0 0

K02361 0 0 0 0 0 0 0 0 0 0 0 0 0 0 0 0 0 0 0 0 0 0 0 0 0 0 0 0 0 0 0 0 0 0 0 0 0 0 0 0 0 0 0 0 0 0 0 0

K02362 0 0 0 0 0 0 0 0 0 0 0 0 0 0 0 0 0 0 0 0 0 0 0 0 0 0 0 0 0 0 0 0 0 0 0 0 0 0 0 0 0 0 0 0 0 0 0 0

K02363 0 0 0 0 0 0 0 0 0 0 0 0 0 0 0 0 0 0 0 0 0 0 0 0 0 0 0 0 0 0 0 0 0 0 0 0 0 0 0 0 0 0 0 0 0 0 0 0

K02364 0 0 0 0 0 0 0 0 0 0 0 0 0 0 0 0 0 0 0 0 0 0 0 0 0 0 0 0 0 0 0 0 0 0 0 0 0 0 0 0 0 0 0 0 0 0 0 0

K02371 0 0 0 0 0 0 0 0 0 0 0 0 0 0 0 0 0 0 0 0 0 0 0 0 0 0 0 0 0 0 0 0 0 0 0 0 0 0 0 0 0 0 0 0 0 0 0 0

K02372 338 420 344 166 406 320 267 564 552 574 661 728 268 601 372 202 508 856 526 476 648 784 838 582 354 338 346 218 292 502 24 648 436 638 772 654 212 438 429 340 452 498 327 580 572 350 470 533

K02377 0 0 0 0 0 0 0 0 0 0 0 0 0 0 0 0 0 0 0 0 0 0 0 0 0 0 0 0 0 0 0 0 0 0 0 0 0 0 0 0 0 0 0 0 0 0 0 0

K02379 100 58 180 79 510 386 124 655 124 223 351 130 46 109 202 48 190 403 106 77 171 284 208 362 12 56 34 9 59 518 0 112 78 130 124 104 66 79 155 228 320 258 28 182 168 284 102 404

K02380 0 0 0 0 0 0 0 0 0 0 0 0 0 0 0 0 0 0 0 0 0 0 0 0 0 0 0 0 0 0 0 0 0 0 0 0 0 0 0 0 0 0 0 0 0 0 0 0

K02381 0 0 0 0 0 0 0 0 0 0 0 0 0 0 0 0 0 0 0 0 0 0 0 0 0 0 0 0 0 0 0 0 0 0 0 0 0 0 0 0 0 0 0 0 0 0 0 0

K02382 0 0 0 0 0 0 0 0 0 0 0 0 0 0 0 0 0 0 0 0 0 0 0 0 0 0 0 0 0 0 0 0 0 0 0 0 0 0 0 0 0 0 0 0 0 0 0 0

K02383 0 0 0 0 0 0 0 0 0 0 0 0 0 0 0 0 0 0 0 0 0 0 0 0 0 0 0 0 0 0 0 0 0 0 0 0 0 0 0 0 0 0 0 0 0 0 0 0

K02384 0 0 0 0 0 0 0 0 0 0 0 0 0 0 0 0 0 0 0 0 0 0 0 0 0 0 0 0 0 0 0 0 0 0 0 0 0 0 0 0 0 0 0 0 0 0 0 0

K02385 0 0 0 0 0 0 0 0 0 0 0 0 0 0 0 0 0 0 0 0 0 0 0 0 0 0 0 0 0 0 0 0 0 0 0 0 0 0 0 0 0 0 0 0 0 0 0 0

K02386 0 0 0 0 0 0 0 0 0 0 0 0 0 0 0 0 0 0 0 0 0 0 0 0 0 0 0 0 0 0 0 0 0 0 0 0 0 0 0 0 0 0 0 0 0 0 0 0

K02387 0 0 0 0 0 0 0 0 0 0 0 0 0 0 0 0 0 0 0 0 0 0 0 0 0 0 0 0 0 0 0 0 0 0 0 0 0 0 0 0 0 0 0 0 0 0 0 0

K02388 0 0 0 0 0 0 0 0 0 0 0 0 0 0 0 0 0 0 0 0 0 0 0 0 0 0 0 0 0 0 0 0 0 0 0 0 0 0 0 0 0 0 0 0 0 0 0 0

K02389 0 0 0 0 0 0 0 0 0 0 0 0 0 0 0 0 0 0 0 0 0 0 0 0 0 0 0 0 0 0 0 0 0 0 0 0 0 0 0 0 0 0 0 0 0 0 0 0

K02390 0 0 0 0 0 0 0 0 0 0 0 0 0 0 0 0 0 0 0 0 0 0 0 0 0 0 0 0 0 0 0 0 0 0 0 0 0 0 0 0 0 0 0 0 0 0 0 0

K02391 0 0 0 0 0 0 0 0 0 0 0 0 0 0 0 0 0 0 0 0 0 0 0 0 0 0 0 0 0 0 0 0 0 0 0 0 0 0 0 0 0 0 0 0 0 0 0 0

K02392 338 420 344 166 406 320 267 564 552 574 661 728 268 601 372 202 508 856 526 476 648 784 838 582 354 338 346 218 292 502 24 648 436 638 772 654 212 438 429 340 452 498 327 580 572 350 470 533

K02393 0 0 0 0 0 0 0 0 0 0 0 0 0 0 0 0 0 0 0 0 0 0 0 0 0 0 0 0 0 0 0 0 0 0 0 0 0 0 0 0 0 0 0 0 0 0 0 0

K02394 0 0 0 0 0 0 0 0 0 0 0 0 0 0 0 0 0 0 0 0 0 0 0 0 0 0 0 0 0 0 0 0 0 0 0 0 0 0 0 0 0 0 0 0 0 0 0 0

K02395 0 0 0 0 0 0 0 0 0 0 0 0 0 0 0 0 0 0 0 0 0 0 0 0 0 0 0 0 0 0 0 0 0 0 0 0 0 0 0 0 0 0 0 0 0 0 0 0

K02396 0 0 0 0 0 0 0 0 0 0 0 0 0 0 0 0 0 0 0 0 0 0 0 0 0 0 0 0 0 0 0 0 0 0 0 0 0 0 0 0 0 0 0 0 0 0 0 0

K02397 0 0 0 0 0 0 0 0 0 0 0 0 0 0 0 0 0 0 0 0 0 0 0 0 0 0 0 0 0 0 0 0 0 0 0 0 0 0 0 0 0 0 0 0 0 0 0 0

K02398 0 0 0 0 0 0 0 0 0 0 0 0 0 0 0 0 0 0 0 0 0 0 0 0 0 0 0 0 0 0 0 0 0 0 0 0 0 0 0 0 0 0 0 0 0 0 0 0

K02399 0 0 0 0 0 0 0 0 0 0 0 0 0 0 0 0 0 0 0 0 0 0 0 0 0 0 0 0 0 0 0 0 0 0 0 0 0 0 0 0 0 0 0 0 0 0 0 0

K02400 0 0 0 0 0 0 0 0 0 0 0 0 0 0 0 0 0 0 0 0 0 0 0 0 0 0 0 0 0 0 0 0 0 0 0 0 0 0 0 0 0 0 0 0 0 0 0 0

K02401 0 0 0 0 0 0 0 0 0 0 0 0 0 0 0 0 0 0 0 0 0 0 0 0 0 0 0 0 0 0 0 0 0 0 0 0 0 0 0 0 0 0 0 0 0 0 0 0

K02402 0 0 0 0 0 0 0 0 0 0 0 0 0 0 0 0 0 0 0 0 0 0 0 0 0 0 0 0 0 0 0 0 0 0 0 0 0 0 0 0 0 0 0 0 0 0 0 0

K02403 0 0 0 0 0 0 0 0 0 0 0 0 0 0 0 0 0 0 0 0 0 0 0 0 0 0 0 0 0 0 0 0 0 0 0 0 0 0 0 0 0 0 0 0 0 0 0 0

K02404 0 0 0 0 0 0 0 0 0 0 0 0 0 0 0 0 0 0 0 0 0 0 0 0 0 0 0 0 0 0 0 0 0 0 0 0 0 0 0 0 0 0 0 0 0 0 0 0

K02405 0 0 0 0 0 0 0 0 0 0 0 0 0 0 0 0 0 0 0 0 0 0 0 0 0 0 0 0 0 0 0 0 0 0 0 0 0 0 0 0 0 0 0 0 0 0 0 0

K02406 338 420 344 166 406 320 267 564 552 574 661 728 268 601 372 202 508 856 526 476 648 784 838 582 354 338 346 218 292 502 24 648 436 638 772 654 212 438 429 340 452 498 327 580 572 350 470 533

K02407 0 0 0 0 0 0 0 0 0 0 0 0 0 0 0 0 0 0 0 0 0 0 0 0 0 0 0 0 0 0 0 0 0 0 0 0 0 0 0 0 0 0 0 0 0 0 0 0

K02408 0 0 0 0 0 0 0 0 0 0 0 0 0 0 0 0 0 0 0 0 0 0 0 0 0 0 0 0 0 0 0 0 0 0 0 0 0 0 0 0 0 0 0 0 0 0 0 0

K02409 0 0 0 0 0 0 0 0 0 0 0 0 0 0 0 0 0 0 0 0 0 0 0 0 0 0 0 0 0 0 0 0 0 0 0 0 0 0 0 0 0 0 0 0 0 0 0 0

K02410 0 0 0 0 0 0 0 0 0 0 0 0 0 0 0 0 0 0 0 0 0 0 0 0 0 0 0 0 0 0 0 0 0 0 0 0 0 0 0 0 0 0 0 0 0 0 0 0

K02411 0 0 0 0 0 0 0 0 0 0 0 0 0 0 0 0 0 0 0 0 0 0 0 0 0 0 0 0 0 0 0 0 0 0 0 0 0 0 0 0 0 0 0 0 0 0 0 0

K02412 0 0 0 0 0 0 0 0 0 0 0 0 0 0 0 0 0 0 0 0 0 0 0 0 0 0 0 0 0 0 0 0 0 0 0 0 0 0 0 0 0 0 0 0 0 0 0 0

K02413 0 0 0 0 0 0 0 0 0 0 0 0 0 0 0 0 0 0 0 0 0 0 0 0 0 0 0 0 0 0 0 0 0 0 0 0 0 0 0 0 0 0 0 0 0 0 0 0

K02414 0 0 0 0 0 0 0 0 0 0 0 0 0 0 0 0 0 0 0 0 0 0 0 0 0 0 0 0 0 0 0 0 0 0 0 0 0 0 0 0 0 0 0 0 0 0 0 0

K02415 0 0 0 0 0 0 0 0 0 0 0 0 0 0 0 0 0 0 0 0 0 0 0 0 0 0 0 0 0 0 0 0 0 0 0 0 0 0 0 0 0 0 0 0 0 0 0 0

K02416 0 0 0 0 0 0 0 0 0 0 0 0 0 0 0 0 0 0 0 0 0 0 0 0 0 0 0 0 0 0 0 0 0 0 0 0 0 0 0 0 0 0 0 0 0 0 0 0

K02417 0 0 0 0 0 0 0 0 0 0 0 0 0 0 0 0 0 0 0 0 0 0 0 0 0 0 0 0 0 0 0 0 0 0 0 0 0 0 0 0 0 0 0 0 0 0 0 0

K02418 0 0 0 0 0 0 0 0 0 0 0 0 0 0 0 0 0 0 0 0 0 0 0 0 0 0 0 0 0 0 0 0 0 0 0 0 0 0 0 0 0 0 0 0 0 0 0 0

K02419 0 0 0 0 0 0 0 0 0 0 0 0 0 0 0 0 0 0 0 0 0 0 0 0 0 0 0 0 0 0 0 0 0 0 0 0 0 0 0 0 0 0 0 0 0 0 0 0

K02420 0 0 0 0 0 0 0 0 0 0 0 0 0 0 0 0 0 0 0 0 0 0 0 0 0 0 0 0 0 0 0 0 0 0 0 0 0 0 0 0 0 0 0 0 0 0 0 0

K02421 0 0 0 0 0 0 0 0 0 0 0 0 0 0 0 0 0 0 0 0 0 0 0 0 0 0 0 0 0 0 0 0 0 0 0 0 0 0 0 0 0 0 0 0 0 0 0 0

K02422 0 0 0 0 0 0 0 0 0 0 0 0 0 0 0 0 0 0 0 0 0 0 0 0 0 0 0 0 0 0 0 0 0 0 0 0 0 0 0 0 0 0 0 0 0 0 0 0

K02423 0 0 0 0 0 0 0 0 0 0 0 0 0 0 0 0 0 0 0 0 0 0 0 0 0 0 0 0 0 0 0 0 0 0 0 0 0 0 0 0 0 0 0 0 0 0 0 0

K02424 0 0 0 0 0 0 0 0 0 0 0 0 0 0 0 0 0 0 0 0 0 0 0 0 0 0 0 0 0 0 0 0 0 0 0 0 0 0 0 0 0 0 0 0 0 0 0 0

K02425 0 0 0 0 0 0 0 0 0 0 0 0 0 0 0 0 0 0 0 0 0 0 0 0 0 0 0 0 0 0 0 0 0 0 0 0 0 0 0 0 0 0 0 0 0 0 0 0

K02426 0 0 0 0 0 0 0 0 0 0 0 0 0 0 0 0 0 0 0 0 0 0 0 0 0 0 0 0 0 0 0 0 0 0 0 0 0 0 0 0 0 0 0 0 0 0 0 0

K02427 140 304 301 96 572 477 136 656 216 244 481 240 119 325 347 119 410 526 223 347 439 342 496 482 120 132 126 90 166 704 0 391 212 228 322 280 96 320 486 328 500 486 182 590 590 308 318 602

K02428 0 0 0 0 0 0 0 0 0 0 0 0 0 0 0 0 0 0 0 0 0 0 0 0 0 0 0 0 0 0 0 0 0 0 0 0 0 0 0 0 0 0 0 0 0 0 0 0

K02429 0 0 0 0 0 0 0 0 0 0 0 0 0 0 0 0 0 0 0 0 0 0 0 0 0 0 0 0 0 0 0 0 0 0 0 0 0 0 0 0 0 0 0 0 0 0 0 0

K02430 0 0 0 0 0 0 0 0 0 0 0 0 0 0 0 0 0 0 0 0 0 0 0 0 0 0 0 0 0 0 0 0 0 0 0 0 0 0 0 0 0 0 0 0 0 0 0 0

K02431 0 0 0 0 0 0 0 0 0 0 0 0 0 0 0 0 0 0 0 0 0 0 0 0 0 0 0 0 0 0 0 0 0 0 0 0 0 0 0 0 0 0 0 0 0 0 0 0

K02433 842 984 937 427 1507 1186 679 1986 1298 1397 1772 1628 591 1468 1055 504 1294 2251 1169 1156 1607 1906 2064 1645 754 737 745 517 672 1756 48 1602 1067 1525 1768 1529 498 1016 1139 973 1370 1392 785 1516 1447 1026 1134 1654

K02434 842 984 937 427 1507 1186 679 1986 1298 1397 1772 1628 591 1468 1055 504 1294 2251 1169 1156 1607 1906 2064 1645 754 737 745 517 672 1756 48 1602 1067 1525 1768 1529 498 1016 1139 973 1370 1392 785 1516 1447 1026 1134 1654

K02435 842 984 937 427 1507 1186 679 1986 1298 1397 1772 1628 591 1468 1055 504 1294 2251 1169 1156 1607 1906 2064 1645 754 737 745 517 672 1756 48 1602 1067 1525 1768 1529 498 1016 1139 973 1370 1392 785 1516 1447 1026 1134 1654

K02436 0 0 0 0 0 0 0 0 0 0 0 0 0 0 0 0 0 0 0 0 0 0 0 0 0 0 0 0 0 0 0 0 0 0 0 0 0 0 0 0 0 0 0 0 0 0 0 0

K02437 380 620 503 216 564 468 331 708 657 694 886 882 358 732 532 247 742 1066 690 680 883 941 1072 790 438 441 440 238 409 745 24 812 504 705 954 792 270 674 724 497 653 742 406 916 953 474 654 776

K02438 0 0 0 0 0 0 0 0 0 0 0 0 0 0 0 0 0 0 0 0 0 0 0 0 0 0 0 0 0 0 0 0 0 0 0 0 0 0 0 0 0 0 0 0 0 0 0 0

K02439 0 0 0 0 0 0 0 0 0 0 0 0 0 0 0 0 0 0 0 0 0 0 0 0 0 0 0 0 0 0 0 0 0 0 0 0 0 0 0 0 0 0 0 0 0 0 0 0

K02440 66 40 107 47 282 216 72 348 84 124 195 86 28 72 124 27 101 222 59 62 106 150 124 208 12 30 21 9 38 292 0 80 50 88 83 77 37 54 90 122 168 154 28 103 96 144 59 230

K02441 0 0 0 0 0 0 0 0 0 0 0 0 0 0 0 0 0 0 0 0 0 0 0 0 0 0 0 0 0 0 0 0 0 0 0 0 0 0 0 0 0 0 0 0 0 0 0 0

K02442 0 0 0 0 0 0 0 0 0 0 0 0 0 0 0 0 0 0 0 0 0 0 0 0 0 0 0 0 0 0 0 0 0 0 0 0 0 0 0 0 0 0 0 0 0 0 0 0

K02443 0 0 0 0 0 0 0 0 0 0 0 0 0 0 0 0 0 0 0 0 0 0 0 0 0 0 0 0 0 0 0 0 0 0 0 0 0 0 0 0 0 0 0 0 0 0 0 0

K02444 0 0 0 0 0 0 0 0 0 0 0 0 0 0 0 0 0 0 0 0 0 0 0 0 0 0 0 0 0 0 0 0 0 0 0 0 0 0 0 0 0 0 0 0 0 0 0 0

K02445 0 0 0 0 0 0 0 0 0 0 0 0 0 0 0 0 0 0 0 0 0 0 0 0 0 0 0 0 0 0 0 0 0 0 0 0 0 0 0 0 0 0 0 0 0 0 0 0

K02446 0 0 0 0 0 0 0 0 0 0 0 0 0 0 0 0 0 0 0 0 0 0 0 0 0 0 0 0 0 0 0 0 0 0 0 0 0 0 0 0 0 0 0 0 0 0 0 0

K02448 0 0 0 0 0 0 0 0 0 0 0 0 0 0 0 0 0 0 0 0 0 0 0 0 0 0 0 0 0 0 0 0 0 0 0 0 0 0 0 0 0 0 0 0 0 0 0 0

K02450 0 0 0 0 0 0 0 0 0 0 0 0 0 0 0 0 0 0 0 0 0 0 0 0 0 0 0 0 0 0 0 0 0 0 0 0 0 0 0 0 0 0 0 0 0 0 0 0

K02451 0 0 0 0 0 0 0 0 0 0 0 0 0 0 0 0 0 0 0 0 0 0 0 0 0 0 0 0 0 0 0 0 0 0 0 0 0 0 0 0 0 0 0 0 0 0 0 0

K02452 0 0 0 0 0 0 0 0 0 0 0 0 0 0 0 0 0 0 0 0 0 0 0 0 0 0 0 0 0 0 0 0 0 0 0 0 0 0 0 0 0 0 0 0 0 0 0 0

K02453 0 0 0 0 0 0 0 0 0 0 0 0 0 0 0 0 0 0 0 0 0 0 0 0 0 0 0 0 0 0 0 0 0 0 0 0 0 0 0 0 0 0 0 0 0 0 0 0

K02454 0 0 0 0 0 0 0 0 0 0 0 0 0 0 0 0 0 0 0 0 0 0 0 0 0 0 0 0 0 0 0 0 0 0 0 0 0 0 0 0 0 0 0 0 0 0 0 0

K02455 0 0 0 0 0 0 0 0 0 0 0 0 0 0 0 0 0 0 0 0 0 0 0 0 0 0 0 0 0 0 0 0 0 0 0 0 0 0 0 0 0 0 0 0 0 0 0 0

K02456 0 0 0 0 0 0 0 0 0 0 0 0 0 0 0 0 0 0 0 0 0 0 0 0 0 0 0 0 0 0 0 0 0 0 0 0 0 0 0 0 0 0 0 0 0 0 0 0

K02457 0 0 0 0 0 0 0 0 0 0 0 0 0 0 0 0 0 0 0 0 0 0 0 0 0 0 0 0 0 0 0 0 0 0 0 0 0 0 0 0 0 0 0 0 0 0 0 0

K02458 0 0 0 0 0 0 0 0 0 0 0 0 0 0 0 0 0 0 0 0 0 0 0 0 0 0 0 0 0 0 0 0 0 0 0 0 0 0 0 0 0 0 0 0 0 0 0 0

K02459 0 0 0 0 0 0 0 0 0 0 0 0 0 0 0 0 0 0 0 0 0 0 0 0 0 0 0 0 0 0 0 0 0 0 0 0 0 0 0 0 0 0 0 0 0 0 0 0

K02460 0 0 0 0 0 0 0 0 0 0 0 0 0 0 0 0 0 0 0 0 0 0 0 0 0 0 0 0 0 0 0 0 0 0 0 0 0 0 0 0 0 0 0 0 0 0 0 0

K02461 0 0 0 0 0 0 0 0 0 0 0 0 0 0 0 0 0 0 0 0 0 0 0 0 0 0 0 0 0 0 0 0 0 0 0 0 0 0 0 0 0 0 0 0 0 0 0 0

K02462 0 0 0 0 0 0 0 0 0 0 0 0 0 0 0 0 0 0 0 0 0 0 0 0 0 0 0 0 0 0 0 0 0 0 0 0 0 0 0 0 0 0 0 0 0 0 0 0

K02463 0 0 0 0 0 0 0 0 0 0 0 0 0 0 0 0 0 0 0 0 0 0 0 0 0 0 0 0 0 0 0 0 0 0 0 0 0 0 0 0 0 0 0 0 0 0 0 0

K02464 0 0 0 0 0 0 0 0 0 0 0 0 0 0 0 0 0 0 0 0 0 0 0 0 0 0 0 0 0 0 0 0 0 0 0 0 0 0 0 0 0 0 0 0 0 0 0 0

K02465 0 0 0 0 0 0 0 0 0 0 0 0 0 0 0 0 0 0 0 0 0 0 0 0 0 0 0 0 0 0 0 0 0 0 0 0 0 0 0 0 0 0 0 0 0 0 0 0

K02466 0 0 0 0 0 0 0 0 0 0 0 0 0 0 0 0 0 0 0 0 0 0 0 0 0 0 0 0 0 0 0 0 0 0 0 0 0 0 0 0 0 0 0 0 0 0 0 0

K02467 0 0 0 0 0 0 0 0 0 0 0 0 0 0 0 0 0 0 0 0 0 0 0 0 0 0 0 0 0 0 0 0 0 0 0 0 0 0 0 0 0 0 0 0 0 0 0 0

K02468 0 0 0 0 0 0 0 0 0 0 0 0 0 0 0 0 0 0 0 0 0 0 0 0 0 0 0 0 0 0 0 0 0 0 0 0 0 0 0 0 0 0 0 0 0 0 0 0

K02469 380 620 503 216 588 484 331 717 657 694 889 882 358 732 532 247 742 1080 690 680 883 941 1072 813 438 441 440 238 409 762 24 842 504 705 954 792 270 674 724 497 653 742 406 916 953 474 654 776

K02470 380 620 503 216 588 484 331 717 657 694 889 882 358 732 532 247 742 1080 690 680 883 941 1072 813 438 441 440 238 409 762 24 842 504 705 954 792 270 674 724 497 653 742 406 916 953 474 654 776

K02471 0 0 0 0 0 0 0 0 0 0 0 0 0 0 0 0 0 0 0 0 0 0 0 0 0 0 0 0 0 0 0 0 0 0 0 0 0 0 0 0 0 0 0 0 0 0 0 0

K02472 99 104 142 47 414 330 72 511 110 124 256 86 28 194 188 74 176 317 59 142 204 186 263 274 35 30 32 71 49 461 0 226 144 161 140 143 37 85 192 171 298 242 103 254 208 184 134 358

K02473 0 0 0 0 0 0 0 0 0 0 0 0 0 0 0 0 0 0 0 0 0 0 0 0 0 0 0 0 0 0 0 0 0 0 0 0 0 0 0 0 0 0 0 0 0 0 0 0

K02474 0 0 0 0 0 0 0 0 0 0 3 0 0 0 0 0 0 0 0 0 0 0 0 17 0 0 0 0 0 4 0 34 0 0 0 0 0 0 0 0 0 0 0 0 0 0 0 0

K02475 0 0 0 0 0 0 0 0 0 0 0 0 0 0 0 0 0 0 0 0 0 0 0 0 0 0 0 0 0 0 0 0 0 0 0 0 0 0 0 0 0 0 0 0 0 0 0 0

K02476 0 0 0 0 0 0 0 0 0 0 0 0 0 0 0 0 0 0 0 0 0 0 0 0 0 0 0 0 0 0 0 0 0 0 0 0 0 0 0 0 0 0 0 0 0 0 0 0

K02477 0 0 0 0 0 0 0 0 0 0 0 0 0 0 0 0 0 0 0 0 0 0 0 0 0 0 0 0 0 0 0 0 0 0 0 0 0 0 0 0 0 0 0 0 0 0 0 0

K02478 33 18 73 32 205 154 52 298 40 98 154 45 18 37 78 20 89 167 48 14 66 133 84 130 1 26 13 0 21 209 0 5 28 42 40 28 28 24 66 106 152 105 0 78 72 140 43 173

K02479 0 0 0 0 0 0 0 0 0 0 0 0 0 0 0 0 0 0 0 0 0 0 0 0 0 0 0 0 0 0 0 0 0 0 0 0 0 0 0 0 0 0 0 0 0 0 0 0

K02480 0 0 0 0 0 0 0 0 0 0 0 0 0 0 0 0 0 0 0 0 0 0 0 0 0 0 0 0 0 0 0 0 0 0 0 0 0 0 0 0 0 0 0 0 0 0 0 0

K02481 0 0 0 0 0 0 0 0 0 0 0 0 0 0 0 0 0 0 0 0 0 0 0 0 0 0 0 0 0 0 0 0 0 0 0 0 0 0 0 0 0 0 0 0 0 0 0 0

K02482 0 0 0 0 0 0 0 0 0 0 0 0 0 0 0 0 0 0 0 0 0 0 0 0 0 0 0 0 0 0 0 0 0 0 0 0 0 0 0 0 0 0 0 0 0 0 0 0

K02483 338 420 344 166 406 320 267 564 552 574 661 728 268 601 372 202 508 856 526 476 648 784 838 582 354 338 346 218 292 502 24 648 436 638 772 654 212 438 429 340 452 498 327 580 572 350 470 533

K02484 0 0 0 0 0 0 0 0 0 0 0 0 0 0 0 0 0 0 0 0 0 0 0 0 0 0 0 0 0 0 0 0 0 0 0 0 0 0 0 0 0 0 0 0 0 0 0 0

K02485 56 0 0 0 32 0 42 0 0 0 0 0 0 0 80 0 0 0 0 74 0 0 0 68 0 0 0 0 0 0 0 0 0 98 0 0 0 0 0 0 0 42 0 0 0 0 0 0

K02486 42 0 0 0 20 0 26 0 0 0 0 0 0 0 48 0 0 0 0 56 0 0 0 40 0 0 0 0 0 0 0 0 0 74 0 0 0 0 0 0 0 26 0 0 0 0 0 0

K02487 0 0 0 0 0 0 0 0 0 0 0 0 0 0 0 0 0 0 0 0 0 0 0 0 0 0 0 0 0 0 0 0 0 0 0 0 0 0 0 0 0 0 0 0 0 0 0 0

K02488 0 0 0 0 0 0 0 0 0 0 0 0 0 0 0 0 0 0 0 0 0 0 0 0 0 0 0 0 0 0 0 0 0 0 0 0 0 0 0 0 0 0 0 0 0 0 0 0

K02489 0 0 0 0 0 0 0 0 0 0 0 0 0 0 0 0 0 0 0 0 0 0 0 0 0 0 0 0 0 0 0 0 0 0 0 0 0 0 0 0 0 0 0 0 0 0 0 0

K02490 0 0 0 0 0 0 0 0 0 0 0 0 0 0 0 0 0 0 0 0 0 0 0 0 0 0 0 0 0 0 0 0 0 0 0 0 0 0 0 0 0 0 0 0 0 0 0 0

K02491 0 0 0 0 0 0 0 0 0 0 0 0 0 0 0 0 0 0 0 0 0 0 0 0 0 0 0 0 0 0 0 0 0 0 0 0 0 0 0 0 0 0 0 0 0 0 0 0

K02492 437 524 486 214 819 650 340 1074 662 698 917 814 296 794 560 275 685 1173 584 618 853 970 1102 855 388 368 378 290 342 963 24 874 580 799 912 798 249 524 620 511 750 740 430 833 780 533 604 891

K02493 338 420 344 166 406 320 267 564 552 574 661 728 268 601 372 202 508 856 526 476 648 784 838 582 354 338 346 218 292 502 24 648 436 638 772 654 212 438 429 340 452 498 327 580 572 350 470 533

K02494 0 0 0 0 0 0 0 0 0 0 0 0 0 0 0 0 0 0 0 0 0 0 0 0 0 0 0 0 0 0 0 0 0 0 0 0 0 0 0 0 0 0 0 0 0 0 0 0

K02495 338 420 344 166 406 320 267 564 552 574 661 728 268 601 372 202 508 856 526 476 648 784 838 582 354 338 346 218 292 502 24 648 436 638 772 654 212 438 429 340 452 498 327 580 572 350 470 533

K02496 0 0 0 0 0 0 0 0 0 0 0 0 0 0 0 0 0 0 0 0 0 0 0 0 0 0 0 0 0 0 0 0 0 0 0 0 0 0 0 0 0 0 0 0 0 0 0 0

K02497 0 0 0 0 0 0 0 0 0 0 0 0 0 0 0 0 0 0 0 0 0 0 0 0 0 0 0 0 0 0 0 0 0 0 0 0 0 0 0 0 0 0 0 0 0 0 0 0

K02498 0 0 0 0 0 0 0 0 0 0 0 0 0 0 0 0 0 0 0 0 0 0 0 0 0 0 0 0 0 0 0 0 0 0 0 0 0 0 0 0 0 0 0 0 0 0 0 0

K02499 0 0 0 0 0 0 0 0 0 0 0 0 0 0 0 0 0 0 0 0 0 0 0 0 0 0 0 0 0 0 0 0 0 0 0 0 0 0 0 0 0 0 0 0 0 0 0 0

K02500 437 524 486 214 819 650 340 1074 662 698 917 814 296 794 560 275 685 1173 584 618 853 970 1102 855 388 368 378 290 342 963 24 874 580 799 912 798 249 524 620 511 750 740 430 833 780 533 604 891

K02501 437 524 486 214 820 650 340 1074 662 698 917 814 296 794 560 275 685 1173 584 618 853 970 1102 855 388 368 378 290 342 963 24 874 580 799 912 798 249 524 620 511 750 740 430 833 780 533 604 891

K02502 0 0 0 0 0 0 0 0 0 0 0 0 0 0 0 0 0 0 0 0 0 0 0 0 0 0 0 0 0 0 0 0 0 0 0 0 0 0 0 0 0 0 0 0 0 0 0 0

K02503 370 484 379 166 536 435 267 727 578 574 722 728 268 722 436 248 584 951 526 555 748 820 978 646 377 338 358 280 304 672 24 794 530 711 829 720 212 469 531 389 582 586 402 730 684 390 545 660

K02504 0 0 0 0 0 0 0 0 0 0 0 0 0 0 0 0 0 0 0 0 0 0 0 0 0 0 0 0 0 0 0 0 0 0 0 0 0 0 0 0 0 0 0 0 0 0 0 0

K02505 0 0 0 0 0 0 0 0 0 0 0 0 0 0 0 0 0 0 0 0 0 0 0 0 0 0 0 0 0 0 0 0 0 0 0 0 0 0 0 0 0 0 0 0 0 0 0 0

K02506 0 0 0 0 0 0 0 0 0 0 0 0 0 0 0 0 0 0 0 0 0 0 0 0 0 0 0 0 0 0 0 0 0 0 0 0 0 0 0 0 0 0 0 0 0 0 0 0

K02507 0 0 0 0 0 0 0 0 0 0 0 0 0 0 0 0 0 0 0 0 0 0 0 0 0 0 0 0 0 0 0 0 0 0 0 0 0 0 0 0 0 0 0 0 0 0 0 0

K02508 0 0 0 0 0 0 0 0 0 0 0 0 0 0 0 0 0 0 0 0 0 0 0 0 0 0 0 0 0 0 0 0 0 0 0 0 0 0 0 0 0 0 0 0 0 0 0 0

K02509 0 0 0 0 0 0 0 0 0 0 0 0 0 0 0 0 0 0 0 0 0 0 0 0 0 0 0 0 0 0 0 0 0 0 0 0 0 0 0 0 0 0 0 0 0 0 0 0

K02510 0 0 0 0 0 0 0 0 0 0 0 0 0 0 0 0 0 0 0 0 0 0 0 0 0 0 0 0 0 0 0 0 0 0 0 0 0 0 0 0 0 0 0 0 0 0 0 0

K02511 0 0 0 0 0 0 0 0 0 0 0 0 0 0 0 0 0 0 0 0 0 0 0 0 0 0 0 0 0 0 0 0 0 0 0 0 0 0 0 0 0 0 0 0 0 0 0 0

K02517 0 0 0 0 0 0 0 0 0 0 0 0 0 0 0 0 0 0 0 0 0 0 0 0 0 0 0 0 0 0 0 0 0 0 0 0 0 0 0 0 0 0 0 0 0 0 0 0

K02518 338 420 344 166 406 320 267 564 552 574 661 728 268 601 372 202 508 856 526 476 648 784 838 582 354 338 346 218 292 502 24 648 436 638 772 654 212 438 429 340 452 498 327 580 572 350 470 533

K02519 338 420 344 166 406 320 267 564 552 574 661 728 268 601 372 202 508 856 526 476 648 784 838 582 354 338 346 218 292 502 24 648 436 638 772 654 212 438 429 340 452 498 327 580 572 350 470 533

K02520 338 420 344 166 406 320 267 564 552 574 661 728 268 601 372 202 508 856 526 476 648 784 838 582 354 338 346 218 292 502 24 648 436 638 772 654 212 438 429 340 452 498 327 580 572 350 470 533

K02521 0 0 0 0 0 0 0 0 0 0 0 0 0 0 0 0 0 0 0 0 0 0 0 0 0 0 0 0 0 0 0 0 0 0 0 0 0 0 0 0 0 0 0 0 0 0 0 0

K02523 338 420 344 166 406 320 267 564 552 574 661 728 268 601 372 202 508 856 526 476 648 784 838 582 354 338 346 218 292 502 24 648 436 638 772 654 212 438 429 340 452 498 327 580 572 350 470 533

K02525 0 0 0 0 0 0 0 0 0 0 0 0 0 0 0 0 0 0 0 0 0 0 0 0 0 0 0 0 0 0 0 0 0 0 0 0 0 0 0 0 0 0 0 0 0 0 0 0

K02526 0 0 0 0 0 0 0 0 0 0 0 0 0 0 0 0 0 0 0 0 0 0 0 0 0 0 0 0 0 0 0 0 0 0 0 0 0 0 0 0 0 0 0 0 0 0 0 0

K02527 0 0 0 0 0 0 0 0 0 0 0 0 0 0 0 0 0 0 0 0 0 0 0 0 0 0 0 0 0 0 0 0 0 0 0 0 0 0 0 0 0 0 0 0 0 0 0 0

K02528 478 724 645 262 978 798 404 1219 768 818 1142 968 386 926 719 320 919 1382 748 822 1088 1127 1335 1064 473 471 472 308 458 1206 24 1038 648 866 1094 934 308 759 916 668 952 984 510 1170 1161 658 788 1134

K02529 338 420 344 166 406 320 267 564 552 574 661 728 268 601 372 202 508 856 526 476 648 784 838 582 354 338 346 218 292 502 24 648 436 638 772 654 212 438 429 340 452 498 327 580 572 350 470 533

K02530 0 0 0 0 0 0 0 0 0 0 0 0 0 0 0 0 0 0 0 0 0 0 0 0 0 0 0 0 0 0 0 0 0 0 0 0 0 0 0 0 0 0 0 0 0 0 0 0

K02531 0 0 0 0 0 0 0 0 0 0 0 0 0 0 0 0 0 0 0 0 0 0 0 0 0 0 0 0 0 0 0 0 0 0 0 0 0 0 0 0 0 0 0 0 0 0 0 0

K02532 0 0 0 0 0 0 0 0 0 0 0 0 0 0 0 0 0 0 0 0 0 0 0 0 0 0 0 0 0 0 0 0 0 0 0 0 0 0 0 0 0 0 0 0 0 0 0 0

K02533 0 0 0 0 0 0 0 0 0 0 0 0 0 0 0 0 0 0 0 0 0 0 0 0 0 0 0 0 0 0 0 0 0 0 0 0 0 0 0 0 0 0 0 0 0 0 0 0

K02535 338 420 344 166 406 320 267 564 552 574 661 728 268 601 372 202 508 856 526 476 648 784 838 582 354 338 346 218 292 502 24 648 436 638 772 654 212 438 429 340 452 498 327 580 572 350 470 533

K02536 338 420 344 166 406 320 267 564 552 574 661 728 268 601 372 202 508 856 526 476 648 784 838 582 354 338 346 218 292 502 24 648 436 638 772 654 212 438 429 340 452 498 327 580 572 350 470 533

K02538 0 0 0 0 0 0 0 0 0 0 0 0 0 0 0 0 0 0 0 0 0 0 0 0 0 0 0 0 0 0 0 0 0 0 0 0 0 0 0 0 0 0 0 0 0 0 0 0

K02545 0 0 0 0 0 0 0 0 0 0 0 0 0 0 0 0 0 0 0 0 0 0 0 0 0 0 0 0 0 0 0 0 0 0 0 0 0 0 0 0 0 0 0 0 0 0 0 0

K02546 0 0 0 0 0 0 0 0 0 0 0 0 0 0 0 0 0 0 0 0 0 0 0 0 0 0 0 0 0 0 0 0 0 0 0 0 0 0 0 0 0 0 0 0 0 0 0 0

K02547 0 0 0 0 0 0 0 0 0 0 0 0 0 0 0 0 0 0 0 0 0 0 0 0 0 0 0 0 0 0 0 0 0 0 0 0 0 0 0 0 0 0 0 0 0 0 0 0

K02548 438 528 447 196 666 542 309 816 665 626 802 810 286 792 526 261 608 1047 548 651 828 855 1057 780 398 348 373 298 338 819 24 918 575 802 914 820 229 529 579 421 614 684 458 779 732 396 577 776

K02549 0 0 0 0 0 0 0 0 0 0 0 0 0 0 0 0 0 0 0 0 0 0 0 0 0 0 0 0 0 0 0 0 0 0 0 0 0 0 0 0 0 0 0 0 0 0 0 0

K02550 0 0 0 0 0 0 0 0 0 0 0 0 0 0 0 0 0 0 0 0 0 0 0 0 0 0 0 0 0 0 0 0 0 0 0 0 0 0 0 0 0 0 0 0 0 0 0 0

K02551 0 0 0 0 0 0 0 0 0 0 0 0 0 0 0 0 0 0 0 0 0 0 0 0 0 0 0 0 0 0 0 0 0 0 0 0 0 0 0 0 0 0 0 0 0 0 0 0

K02552 0 0 0 0 0 0 0 0 0 0 0 0 0 0 0 0 0 0 0 0 0 0 0 0 0 0 0 0 0 0 0 0 0 0 0 0 0 0 0 0 0 0 0 0 0 0 0 0

K02553 0 0 0 0 0 0 0 0 0 0 0 0 0 0 0 0 0 0 0 0 0 0 0 0 0 0 0 0 0 0 0 0 0 0 0 0 0 0 0 0 0 0 0 0 0 0 0 0

K02554 0 0 0 0 0 0 0 0 0 0 0 0 0 0 0 0 0 0 0 0 0 0 0 0 0 0 0 0 0 0 0 0 0 0 0 0 0 0 0 0 0 0 0 0 0 0 0 0

K02556 0 0 0 0 0 0 0 0 0 0 0 0 0 0 0 0 0 0 0 0 0 0 0 0 0 0 0 0 0 0 0 0 0 0 0 0 0 0 0 0 0 0 0 0 0 0 0 0

K02557 338 420 344 166 406 320 267 564 552 574 661 728 268 601 372 202 508 856 526 476 648 784 838 582 354 338 346 218 292 502 24 648 436 638 772 654 212 438 429 340 452 498 327 580 572 350 470 533

K02558 0 0 0 0 0 0 0 0 0 0 0 0 0 0 0 0 0 0 0 0 0 0 0 0 0 0 0 0 0 0 0 0 0 0 0 0 0 0 0 0 0 0 0 0 0 0 0 0

K02560 0 0 0 0 0 0 0 0 0 0 0 0 0 0 0 0 0 0 0 0 0 0 0 0 0 0 0 0 0 0 0 0 0 0 0 0 0 0 0 0 0 0 0 0 0 0 0 0

K02562 0 0 0 0 0 0 0 0 0 0 0 0 0 0 0 0 0 0 0 0 0 0 0 0 0 0 0 0 0 0 0 0 0 0 0 0 0 0 0 0 0 0 0 0 0 0 0 0

K02563 404 460 451 214 664 520 340 902 636 698 853 814 296 673 496 228 610 1064 584 538 754 935 962 767 365 368 367 228 330 776 24 698 486 726 856 732 249 493 518 462 620 652 355 682 668 493 530 764

K02564 0 0 0 0 0 0 0 0 0 0 0 0 0 0 0 0 0 0 0 0 0 0 0 0 0 0 0 0 0 0 0 0 0 0 0 0 0 0 0 0 0 0 0 0 0 0 0 0

K02565 0 0 0 0 0 0 0 0 0 0 0 0 0 0 0 0 0 0 0 0 0 0 0 0 0 0 0 0 0 0 0 0 0 0 0 0 0 0 0 0 0 0 0 0 0 0 0 0

K02566 0 0 0 0 0 0 0 0 0 0 0 0 0 0 0 0 0 0 0 0 0 0 0 0 0 0 0 0 0 0 0 0 0 0 0 0 0 0 0 0 0 0 0 0 0 0 0 0

K02567 0 0 0 0 0 0 0 0 0 0 0 0 0 0 0 0 0 0 0 0 0 0 0 0 0 0 0 0 0 0 0 0 0 0 0 0 0 0 0 0 0 0 0 0 0 0 0 0

K02568 0 0 0 0 0 0 0 0 0 0 0 0 0 0 0 0 0 0 0 0 0 0 0 0 0 0 0 0 0 0 0 0 0 0 0 0 0 0 0 0 0 0 0 0 0 0 0 0

K02569 0 0 0 0 0 0 0 0 0 0 0 0 0 0 0 0 0 0 0 0 0 0 0 0 0 0 0 0 0 0 0 0 0 0 0 0 0 0 0 0 0 0 0 0 0 0 0 0

K02570 0 0 0 0 0 0 0 0 0 0 0 0 0 0 0 0 0 0 0 0 0 0 0 0 0 0 0 0 0 0 0 0 0 0 0 0 0 0 0 0 0 0 0 0 0 0 0 0

K02571 0 0 0 0 0 0 0 0 0 0 0 0 0 0 0 0 0 0 0 0 0 0 0 0 0 0 0 0 0 0 0 0 0 0 0 0 0 0 0 0 0 0 0 0 0 0 0 0

K02572 0 0 0 0 0 0 0 0 0 0 0 0 0 0 0 0 0 0 0 0 0 0 0 0 0 0 0 0 0 0 0 0 0 0 0 0 0 0 0 0 0 0 0 0 0 0 0 0

K02573 0 0 0 0 0 0 0 0 0 0 0 0 0 0 0 0 0 0 0 0 0 0 0 0 0 0 0 0 0 0 0 0 0 0 0 0 0 0 0 0 0 0 0 0 0 0 0 0

K02574 0 0 0 0 0 0 0 0 0 0 0 0 0 0 0 0 0 0 0 0 0 0 0 0 0 0 0 0 0 0 0 0 0 0 0 0 0 0 0 0 0 0 0 0 0 0 0 0

K02575 0 0 0 0 0 0 0 0 0 0 0 0 0 0 0 0 0 0 0 0 0 0 0 0 0 0 0 0 0 0 0 0 0 0 0 0 0 0 0 0 0 0 0 0 0 0 0 0

K02584 0 0 0 0 0 0 0 0 0 0 0 0 0 0 0 0 0 0 0 0 0 0 0 0 0 0 0 0 0 0 0 0 0 0 0 0 0 0 0 0 0 0 0 0 0 0 0 0

K02585 66 40 107 47 282 216 72 348 84 124 195 86 28 72 124 27 101 222 59 62 106 150 124 208 12 30 21 9 38 292 0 80 50 88 83 77 37 54 90 122 168 154 28 103 96 144 59 230

K02586 34 22 34 15 56 46 21 40 44 26 41 40 10 35 45 6 12 41 12 48 40 18 40 72 10 4 8 9 17 70 0 76 22 46 42 50 8 30 24 16 16 48 28 24 24 4 16 58

K02587 48 22 34 15 61 46 30 40 44 26 38 40 10 35 61 6 12 41 12 66 40 18 40 69 10 4 8 9 17 65 0 49 22 70 42 50 8 30 24 16 16 57 28 24 24 4 16 58

K02588 132 126 176 62 468 376 94 551 154 150 297 126 38 228 232 80 188 358 70 190 244 204 302 346 46 34 40 80 66 530 0 302 167 206 182 192 46 115 216 187 314 290 131 278 232 187 150 416

K02589 14 0 0 0 8 0 8 0 0 0 0 0 0 0 16 0 0 0 0 18 0 0 0 14 0 0 0 0 0 0 0 4 0 24 0 0 0 0 0 0 0 8 0 0 0 0 0 0

K02590 14 0 0 0 8 0 8 0 0 0 0 0 0 0 16 0 0 0 0 18 0 0 0 14 0 0 0 0 0 0 0 4 0 24 0 0 0 0 0 0 0 8 0 0 0 0 0 0

K02591 48 22 34 15 56 46 21 40 44 26 41 40 10 35 45 6 12 41 12 66 40 18 40 72 10 4 8 9 17 70 0 76 22 70 42 50 8 30 24 16 16 48 28 24 24 4 16 58

K02592 0 0 0 0 8 0 8 0 0 0 0 0 0 0 16 0 0 0 0 0 0 0 0 14 0 0 0 0 0 0 0 4 0 0 0 0 0 0 0 0 0 8 0 0 0 0 0 0

K02593 0 0 0 0 0 0 0 0 0 0 0 0 0 0 0 0 0 0 0 0 0 0 0 0 0 0 0 0 0 0 0 0 0 0 0 0 0 0 0 0 0 0 0 0 0 0 0 0

K02594 0 0 0 0 0 0 0 0 0 0 0 0 0 0 0 0 0 0 0 0 0 0 0 0 0 0 0 0 0 0 0 0 0 0 0 0 0 0 0 0 0 0 0 0 0 0 0 0

K02595 0 0 0 0 0 0 0 0 0 0 0 0 0 0 0 0 0 0 0 0 0 0 0 0 0 0 0 0 0 0 0 0 0 0 0 0 0 0 0 0 0 0 0 0 0 0 0 0

K02596 0 0 0 0 0 0 0 0 0 0 0 0 0 0 0 0 0 0 0 0 0 0 0 0 0 0 0 0 0 0 0 0 0 0 0 0 0 0 0 0 0 0 0 0 0 0 0 0

K02597 0 0 0 0 0 0 0 0 0 0 0 0 0 0 0 0 0 0 0 0 0 0 0 0 0 0 0 0 0 0 0 0 0 0 0 0 0 0 0 0 0 0 0 0 0 0 0 0

K02598 0 0 0 0 0 0 0 0 0 0 0 0 0 0 0 0 0 0 0 0 0 0 0 0 0 0 0 0 0 0 0 0 0 0 0 0 0 0 0 0 0 0 0 0 0 0 0 0

K02600 478 724 645 262 1024 830 404 1237 768 818 1144 968 386 926 719 320 919 1410 748 822 1088 1127 1335 1092 473 471 472 308 458 1236 24 1068 648 866 1094 934 308 759 916 668 952 984 510 1170 1161 658 788 1134

K02601 478 724 645 262 978 798 404 1219 768 818 1142 968 386 926 719 320 919 1382 748 822 1088 1127 1335 1064 473 471 472 308 458 1206 24 1038 648 866 1094 934 308 759 916 668 952 984 510 1170 1161 658 788 1134

K02609 0 0 0 0 0 0 0 0 0 0 0 0 0 0 0 0 0 0 0 0 0 0 0 0 0 0 0 0 0 0 0 0 0 0 0 0 0 0 0 0 0 0 0 0 0 0 0 0

K02610 0 0 0 0 0 0 0 0 0 0 0 0 0 0 0 0 0 0 0 0 0 0 0 0 0 0 0 0 0 0 0 0 0 0 0 0 0 0 0 0 0 0 0 0 0 0 0 0

K02611 0 0 0 0 0 0 0 0 0 0 0 0 0 0 0 0 0 0 0 0 0 0 0 0 0 0 0 0 0 0 0 0 0 0 0 0 0 0 0 0 0 0 0 0 0 0 0 0

K02612 0 0 0 0 0 0 0 0 0 0 0 0 0 0 0 0 0 0 0 0 0 0 0 0 0 0 0 0 0 0 0 0 0 0 0 0 0 0 0 0 0 0 0 0 0 0 0 0

K02613 0 0 0 0 0 0 0 0 0 0 0 0 0 0 0 0 0 0 0 0 0 0 0 0 0 0 0 0 0 0 0 0 0 0 0 0 0 0 0 0 0 0 0 0 0 0 0 0

K02614 52 40 107 47 276 216 64 348 84 124 195 86 28 72 108 27 101 222 59 44 106 150 124 195 12 30 21 9 38 292 0 80 50 64 83 77 37 54 90 122 168 145 28 103 96 144 59 230

K02615 0 0 0 0 0 0 0 0 0 0 0 0 0 0 0 0 0 0 0 0 0 0 0 0 0 0 0 0 0 0 0 0 0 0 0 0 0 0 0 0 0 0 0 0 0 0 0 0

K02616 0 0 0 0 0 0 0 0 0 0 0 0 0 0 0 0 0 0 0 0 0 0 0 0 0 0 0 0 0 0 0 0 0 0 0 0 0 0 0 0 0 0 0 0 0 0 0 0

K02617 0 0 0 0 0 0 0 0 0 0 0 0 0 0 0 0 0 0 0 0 0 0 0 0 0 0 0 0 0 0 0 0 0 0 0 0 0 0 0 0 0 0 0 0 0 0 0 0

K02618 0 0 0 0 0 0 0 0 0 0 0 0 0 0 0 0 0 0 0 0 0 0 0 0 0 0 0 0 0 0 0 0 0 0 0 0 0 0 0 0 0 0 0 0 0 0 0 0

K02619 0 0 0 0 0 0 0 0 0 0 0 0 0 0 0 0 0 0 0 0 0 0 0 0 0 0 0 0 0 0 0 0 0 0 0 0 0 0 0 0 0 0 0 0 0 0 0 0

K02621 0 0 0 0 0 0 0 0 0 0 0 0 0 0 0 0 0 0 0 0 0 0 0 0 0 0 0 0 0 0 0 0 0 0 0 0 0 0 0 0 0 0 0 0 0 0 0 0

K02622 0 0 0 0 0 0 0 0 0 0 0 0 0 0 0 0 0 0 0 0 0 0 0 0 0 0 0 0 0 0 0 0 0 0 0 0 0 0 0 0 0 0 0 0 0 0 0 0

K02623 0 0 0 0 0 0 0 0 0 0 0 0 0 0 0 0 0 0 0 0 0 0 0 0 0 0 0 0 0 0 0 0 0 0 0 0 0 0 0 0 0 0 0 0 0 0 0 0

K02624 0 0 0 0 0 0 0 0 0 0 0 0 0 0 0 0 0 0 0 0 0 0 0 0 0 0 0 0 0 0 0 0 0 0 0 0 0 0 0 0 0 0 0 0 0 0 0 0

K02625 0 0 0 0 0 0 0 0 0 0 0 0 0 0 0 0 0 0 0 0 0 0 0 0 0 0 0 0 0 0 0 0 0 0 0 0 0 0 0 0 0 0 0 0 0 0 0 0

K02626 140 304 301 96 572 477 136 656 216 244 481 240 119 325 347 119 410 526 223 347 439 342 496 482 120 132 126 90 166 704 0 391 212 228 322 280 96 320 486 328 500 486 182 590 590 308 318 602

K02628 0 0 0 0 0 0 0 0 0 0 0 0 0 0 0 0 0 0 0 0 0 0 0 0 0 0 0 0 0 0 0 0 0 0 0 0 0 0 0 0 0 0 0 0 0 0 0 0

K02629 0 0 0 0 0 0 0 0 0 0 0 0 0 0 0 0 0 0 0 0 0 0 0 0 0 0 0 0 0 0 0 0 0 0 0 0 0 0 0 0 0 0 0 0 0 0 0 0

K02630 0 0 0 0 0 0 0 0 0 0 0 0 0 0 0 0 0 0 0 0 0 0 0 0 0 0 0 0 0 0 0 0 0 0 0 0 0 0 0 0 0 0 0 0 0 0 0 0

K02631 0 0 0 0 0 0 0 0 0 0 0 0 0 0 0 0 0 0 0 0 0 0 0 0 0 0 0 0 0 0 0 0 0 0 0 0 0 0 0 0 0 0 0 0 0 0 0 0

K02632 0 0 0 0 0 0 0 0 0 0 0 0 0 0 0 0 0 0 0 0 0 0 0 0 0 0 0 0 0 0 0 0 0 0 0 0 0 0 0 0 0 0 0 0 0 0 0 0

K02634 0 0 0 0 0 0 0 0 0 0 0 0 0 0 0 0 0 0 0 0 0 0 0 0 0 0 0 0 0 0 0 0 0 0 0 0 0 0 0 0 0 0 0 0 0 0 0 0

K02635 0 0 0 0 0 0 0 0 0 0 0 0 0 0 0 0 0 0 0 0 0 0 0 0 0 0 0 0 0 0 0 0 0 0 0 0 0 0 0 0 0 0 0 0 0 0 0 0

K02636 0 0 0 0 0 0 0 0 0 0 0 0 0 0 0 0 0 0 0 0 0 0 0 0 0 0 0 0 0 0 0 0 0 0 0 0 0 0 0 0 0 0 0 0 0 0 0 0

K02637 0 0 0 0 0 0 0 0 0 0 0 0 0 0 0 0 0 0 0 0 0 0 0 0 0 0 0 0 0 0 0 0 0 0 0 0 0 0 0 0 0 0 0 0 0 0 0 0

K02638 0 0 0 0 0 0 0 0 0 0 0 0 0 0 0 0 0 0 0 0 0 0 0 0 0 0 0 0 0 0 0 0 0 0 0 0 0 0 0 0 0 0 0 0 0 0 0 0

K02639 0 0 0 0 0 0 0 0 0 0 0 0 0 0 0 0 0 0 0 0 0 0 0 0 0 0 0 0 0 0 0 0 0 0 0 0 0 0 0 0 0 0 0 0 0 0 0 0

K02640 0 0 0 0 0 0 0 0 0 0 0 0 0 0 0 0 0 0 0 0 0 0 0 0 0 0 0 0 0 0 0 0 0 0 0 0 0 0 0 0 0 0 0 0 0 0 0 0

K02641 0 0 0 0 0 0 0 0 0 0 0 0 0 0 0 0 0 0 0 0 0 0 0 0 0 0 0 0 0 0 0 0 0 0 0 0 0 0 0 0 0 0 0 0 0 0 0 0

K02642 0 0 0 0 0 0 0 0 0 0 0 0 0 0 0 0 0 0 0 0 0 0 0 0 0 0 0 0 0 0 0 0 0 0 0 0 0 0 0 0 0 0 0 0 0 0 0 0

K02643 0 0 0 0 0 0 0 0 0 0 0 0 0 0 0 0 0 0 0 0 0 0 0 0 0 0 0 0 0 0 0 0 0 0 0 0 0 0 0 0 0 0 0 0 0 0 0 0

K02647 0 0 0 0 0 0 0 0 0 0 0 0 0 0 0 0 0 0 0 0 0 0 0 0 0 0 0 0 0 0 0 0 0 0 0 0 0 0 0 0 0 0 0 0 0 0 0 0

K02650 0 0 0 0 0 0 0 0 0 0 0 0 0 0 0 0 0 0 0 0 0 0 0 0 0 0 0 0 0 0 0 0 0 0 0 0 0 0 0 0 0 0 0 0 0 0 0 0

K02651 0 0 0 0 0 0 0 0 0 0 0 0 0 0 0 0 0 0 0 0 0 0 0 0 0 0 0 0 0 0 0 0 0 0 0 0 0 0 0 0 0 0 0 0 0 0 0 0

K02652 338 420 344 166 406 320 267 564 552 574 661 728 268 601 372 202 508 856 526 476 648 784 838 582 354 338 346 218 292 502 24 648 436 638 772 654 212 438 429 340 452 498 327 580 572 350 470 533

K02653 338 420 344 166 406 320 267 564 552 574 661 728 268 601 372 202 508 856 526 476 648 784 838 582 354 338 346 218 292 502 24 648 436 638 772 654 212 438 429 340 452 498 327 580 572 350 470 533

K02654 338 420 344 166 406 320 267 564 552 574 661 728 268 601 372 202 508 856 526 476 648 784 838 582 354 338 346 218 292 502 24 648 436 638 772 654 212 438 429 340 452 498 327 580 572 350 470 533

K02655 0 0 0 0 0 0 0 0 0 0 0 0 0 0 0 0 0 0 0 0 0 0 0 0 0 0 0 0 0 0 0 0 0 0 0 0 0 0 0 0 0 0 0 0 0 0 0 0

K02656 0 0 0 0 0 0 0 0 0 0 0 0 0 0 0 0 0 0 0 0 0 0 0 0 0 0 0 0 0 0 0 0 0 0 0 0 0 0 0 0 0 0 0 0 0 0 0 0

K02657 0 0 0 0 0 0 0 0 0 0 0 0 0 0 0 0 0 0 0 0 0 0 0 0 0 0 0 0 0 0 0 0 0 0 0 0 0 0 0 0 0 0 0 0 0 0 0 0

K02658 0 0 0 0 0 0 0 0 0 0 3 0 0 0 0 0 0 0 0 0 0 0 0 17 0 0 0 0 0 4 0 30 0 0 0 0 0 0 0 0 0 0 0 0 0 0 0 0

K02659 0 0 0 0 0 0 0 0 0 0 0 0 0 0 0 0 0 0 0 0 0 0 0 0 0 0 0 0 0 0 0 0 0 0 0 0 0 0 0 0 0 0 0 0 0 0 0 0

K02660 0 0 0 0 0 0 0 0 0 0 0 0 0 0 0 0 0 0 0 0 0 0 0 0 0 0 0 0 0 0 0 0 0 0 0 0 0 0 0 0 0 0 0 0 0 0 0 0

K02661 0 0 0 0 0 0 0 0 0 0 0 0 0 0 0 0 0 0 0 0 0 0 0 0 0 0 0 0 0 0 0 0 0 0 0 0 0 0 0 0 0 0 0 0 0 0 0 0

K02662 0 0 0 0 0 0 0 0 0 0 0 0 0 0 0 0 0 0 0 0 0 0 0 0 0 0 0 0 0 0 0 0 0 0 0 0 0 0 0 0 0 0 0 0 0 0 0 0

K02663 0 0 0 0 0 0 0 0 0 0 0 0 0 0 0 0 0 0 0 0 0 0 0 0 0 0 0 0 0 0 0 0 0 0 0 0 0 0 0 0 0 0 0 0 0 0 0 0

K02664 0 0 0 0 0 0 0 0 0 0 0 0 0 0 0 0 0 0 0 0 0 0 0 0 0 0 0 0 0 0 0 0 0 0 0 0 0 0 0 0 0 0 0 0 0 0 0 0

K02665 0 0 0 0 0 0 0 0 0 0 0 0 0 0 0 0 0 0 0 0 0 0 0 0 0 0 0 0 0 0 0 0 0 0 0 0 0 0 0 0 0 0 0 0 0 0 0 0

K02666 0 0 0 0 0 0 0 0 0 0 0 0 0 0 0 0 0 0 0 0 0 0 0 0 0 0 0 0 0 0 0 0 0 0 0 0 0 0 0 0 0 0 0 0 0 0 0 0

K02667 0 0 0 0 0 0 0 0 0 0 0 0 0 0 0 0 0 0 0 0 0 0 0 0 0 0 0 0 0 0 0 0 0 0 0 0 0 0 0 0 0 0 0 0 0 0 0 0

K02668 0 0 0 0 0 0 0 0 0 0 0 0 0 0 0 0 0 0 0 0 0 0 0 0 0 0 0 0 0 0 0 0 0 0 0 0 0 0 0 0 0 0 0 0 0 0 0 0

K02669 338 420 344 166 406 320 267 564 552 574 661 728 268 601 372 202 508 856 526 476 648 784 838 582 354 338 346 218 292 502 24 648 436 638 772 654 212 438 429 340 452 498 327 580 572 350 470 533

K02670 0 0 0 0 0 0 0 0 0 0 0 0 0 0 0 0 0 0 0 0 0 0 0 0 0 0 0 0 0 0 0 0 0 0 0 0 0 0 0 0 0 0 0 0 0 0 0 0

K02671 0 0 0 0 0 0 0 0 0 0 0 0 0 0 0 0 0 0 0 0 0 0 0 0 0 0 0 0 0 0 0 0 0 0 0 0 0 0 0 0 0 0 0 0 0 0 0 0

K02672 0 0 0 0 0 0 0 0 0 0 0 0 0 0 0 0 0 0 0 0 0 0 0 0 0 0 0 0 0 0 0 0 0 0 0 0 0 0 0 0 0 0 0 0 0 0 0 0

K02673 0 0 0 0 0 0 0 0 0 0 0 0 0 0 0 0 0 0 0 0 0 0 0 0 0 0 0 0 0 0 0 0 0 0 0 0 0 0 0 0 0 0 0 0 0 0 0 0

K02674 0 0 0 0 0 0 0 0 0 0 0 0 0 0 0 0 0 0 0 0 0 0 0 0 0 0 0 0 0 0 0 0 0 0 0 0 0 0 0 0 0 0 0 0 0 0 0 0

K02675 0 0 0 0 0 0 0 0 0 0 0 0 0 0 0 0 0 0 0 0 0 0 0 0 0 0 0 0 0 0 0 0 0 0 0 0 0 0 0 0 0 0 0 0 0 0 0 0

K02676 0 0 0 0 0 0 0 0 0 0 0 0 0 0 0 0 0 0 0 0 0 0 0 0 0 0 0 0 0 0 0 0 0 0 0 0 0 0 0 0 0 0 0 0 0 0 0 0

K02679 0 0 0 0 0 0 0 0 0 0 0 0 0 0 0 0 0 0 0 0 0 0 0 0 0 0 0 0 0 0 0 0 0 0 0 0 0 0 0 0 0 0 0 0 0 0 0 0

K02680 0 0 0 0 0 0 0 0 0 0 0 0 0 0 0 0 0 0 0 0 0 0 0 0 0 0 0 0 0 0 0 0 0 0 0 0 0 0 0 0 0 0 0 0 0 0 0 0

K02681 0 0 0 0 0 0 0 0 0 0 0 0 0 0 0 0 0 0 0 0 0 0 0 0 0 0 0 0 0 0 0 0 0 0 0 0 0 0 0 0 0 0 0 0 0 0 0 0

K02682 0 0 0 0 0 0 0 0 0 0 0 0 0 0 0 0 0 0 0 0 0 0 0 0 0 0 0 0 0 0 0 0 0 0 0 0 0 0 0 0 0 0 0 0 0 0 0 0

K02683 140 304 301 96 572 477 136 656 216 244 481 240 119 325 347 119 410 526 223 347 439 342 496 482 120 132 126 90 166 704 0 391 212 228 322 280 96 320 486 328 500 486 182 590 590 308 318 602

K02685 140 304 301 96 572 477 136 656 216 244 481 240 119 325 347 119 410 526 223 347 439 342 496 482 120 132 126 90 166 704 0 391 212 228 322 280 96 320 486 328 500 486 182 590 590 308 318 602

K02686 0 0 0 0 0 0 0 0 0 0 0 0 0 0 0 0 0 0 0 0 0 0 0 0 0 0 0 0 0 0 0 0 0 0 0 0 0 0 0 0 0 0 0 0 0 0 0 0

K02687 338 420 344 166 406 320 267 564 552 574 661 728 268 601 372 202 508 856 526 476 648 784 838 582 354 338 346 218 292 502 24 648 436 638 772 654 212 438 429 340 452 498 327 580 572 350 470 533

K02688 0 0 0 0 0 0 0 0 0 0 0 0 0 0 0 0 0 0 0 0 0 0 0 0 0 0 0 0 0 0 0 0 0 0 0 0 0 0 0 0 0 0 0 0 0 0 0 0

K02689 0 0 0 0 0 0 0 0 0 0 0 0 0 0 0 0 0 0 0 0 0 0 0 0 0 0 0 0 0 0 0 0 0 0 0 0 0 0 0 0 0 0 0 0 0 0 0 0

K02690 0 0 0 0 0 0 0 0 0 0 0 0 0 0 0 0 0 0 0 0 0 0 0 0 0 0 0 0 0 0 0 0 0 0 0 0 0 0 0 0 0 0 0 0 0 0 0 0

K02691 0 0 0 0 0 0 0 0 0 0 0 0 0 0 0 0 0 0 0 0 0 0 0 0 0 0 0 0 0 0 0 0 0 0 0 0 0 0 0 0 0 0 0 0 0 0 0 0

K02692 0 0 0 0 0 0 0 0 0 0 0 0 0 0 0 0 0 0 0 0 0 0 0 0 0 0 0 0 0 0 0 0 0 0 0 0 0 0 0 0 0 0 0 0 0 0 0 0

K02693 0 0 0 0 0 0 0 0 0 0 0 0 0 0 0 0 0 0 0 0 0 0 0 0 0 0 0 0 0 0 0 0 0 0 0 0 0 0 0 0 0 0 0 0 0 0 0 0

K02694 0 0 0 0 0 0 0 0 0 0 0 0 0 0 0 0 0 0 0 0 0 0 0 0 0 0 0 0 0 0 0 0 0 0 0 0 0 0 0 0 0 0 0 0 0 0 0 0

K02696 0 0 0 0 0 0 0 0 0 0 0 0 0 0 0 0 0 0 0 0 0 0 0 0 0 0 0 0 0 0 0 0 0 0 0 0 0 0 0 0 0 0 0 0 0 0 0 0

K02697 0 0 0 0 0 0 0 0 0 0 0 0 0 0 0 0 0 0 0 0 0 0 0 0 0 0 0 0 0 0 0 0 0 0 0 0 0 0 0 0 0 0 0 0 0 0 0 0

K02698 0 0 0 0 0 0 0 0 0 0 0 0 0 0 0 0 0 0 0 0 0 0 0 0 0 0 0 0 0 0 0 0 0 0 0 0 0 0 0 0 0 0 0 0 0 0 0 0

K02699 0 0 0 0 0 0 0 0 0 0 0 0 0 0 0 0 0 0 0 0 0 0 0 0 0 0 0 0 0 0 0 0 0 0 0 0 0 0 0 0 0 0 0 0 0 0 0 0

K02700 0 0 0 0 0 0 0 0 0 0 0 0 0 0 0 0 0 0 0 0 0 0 0 0 0 0 0 0 0 0 0 0 0 0 0 0 0 0 0 0 0 0 0 0 0 0 0 0

K02702 0 0 0 0 0 0 0 0 0 0 0 0 0 0 0 0 0 0 0 0 0 0 0 0 0 0 0 0 0 0 0 0 0 0 0 0 0 0 0 0 0 0 0 0 0 0 0 0

K02703 0 0 0 0 0 0 0 0 0 0 0 0 0 0 0 0 0 0 0 0 0 0 0 0 0 0 0 0 0 0 0 0 0 0 0 0 0 0 0 0 0 0 0 0 0 0 0 0

K02704 0 0 0 0 0 0 0 0 0 0 0 0 0 0 0 0 0 0 0 0 0 0 0 0 0 0 0 0 0 0 0 0 0 0 0 0 0 0 0 0 0 0 0 0 0 0 0 0

K02705 0 0 0 0 0 0 0 0 0 0 0 0 0 0 0 0 0 0 0 0 0 0 0 0 0 0 0 0 0 0 0 0 0 0 0 0 0 0 0 0 0 0 0 0 0 0 0 0

K02706 0 0 0 0 0 0 0 0 0 0 0 0 0 0 0 0 0 0 0 0 0 0 0 0 0 0 0 0 0 0 0 0 0 0 0 0 0 0 0 0 0 0 0 0 0 0 0 0

K02707 0 0 0 0 0 0 0 0 0 0 0 0 0 0 0 0 0 0 0 0 0 0 0 0 0 0 0 0 0 0 0 0 0 0 0 0 0 0 0 0 0 0 0 0 0 0 0 0

K02708 0 0 0 0 0 0 0 0 0 0 0 0 0 0 0 0 0 0 0 0 0 0 0 0 0 0 0 0 0 0 0 0 0 0 0 0 0 0 0 0 0 0 0 0 0 0 0 0

K02709 0 0 0 0 0 0 0 0 0 0 0 0 0 0 0 0 0 0 0 0 0 0 0 0 0 0 0 0 0 0 0 0 0 0 0 0 0 0 0 0 0 0 0 0 0 0 0 0

K02710 0 0 0 0 0 0 0 0 0 0 0 0 0 0 0 0 0 0 0 0 0 0 0 0 0 0 0 0 0 0 0 0 0 0 0 0 0 0 0 0 0 0 0 0 0 0 0 0

K02711 0 0 0 0 0 0 0 0 0 0 0 0 0 0 0 0 0 0 0 0 0 0 0 0 0 0 0 0 0 0 0 0 0 0 0 0 0 0 0 0 0 0 0 0 0 0 0 0

K02712 0 0 0 0 0 0 0 0 0 0 0 0 0 0 0 0 0 0 0 0 0 0 0 0 0 0 0 0 0 0 0 0 0 0 0 0 0 0 0 0 0 0 0 0 0 0 0 0

K02713 0 0 0 0 0 0 0 0 0 0 0 0 0 0 0 0 0 0 0 0 0 0 0 0 0 0 0 0 0 0 0 0 0 0 0 0 0 0 0 0 0 0 0 0 0 0 0 0

K02714 0 0 0 0 0 0 0 0 0 0 0 0 0 0 0 0 0 0 0 0 0 0 0 0 0 0 0 0 0 0 0 0 0 0 0 0 0 0 0 0 0 0 0 0 0 0 0 0

K02715 0 0 0 0 0 0 0 0 0 0 0 0 0 0 0 0 0 0 0 0 0 0 0 0 0 0 0 0 0 0 0 0 0 0 0 0 0 0 0 0 0 0 0 0 0 0 0 0

K02716 0 0 0 0 0 0 0 0 0 0 0 0 0 0 0 0 0 0 0 0 0 0 0 0 0 0 0 0 0 0 0 0 0 0 0 0 0 0 0 0 0 0 0 0 0 0 0 0

K02717 0 0 0 0 0 0 0 0 0 0 0 0 0 0 0 0 0 0 0 0 0 0 0 0 0 0 0 0 0 0 0 0 0 0 0 0 0 0 0 0 0 0 0 0 0 0 0 0

K02718 0 0 0 0 0 0 0 0 0 0 0 0 0 0 0 0 0 0 0 0 0 0 0 0 0 0 0 0 0 0 0 0 0 0 0 0 0 0 0 0 0 0 0 0 0 0 0 0

K02719 0 0 0 0 0 0 0 0 0 0 0 0 0 0 0 0 0 0 0 0 0 0 0 0 0 0 0 0 0 0 0 0 0 0 0 0 0 0 0 0 0 0 0 0 0 0 0 0

K02720 0 0 0 0 0 0 0 0 0 0 0 0 0 0 0 0 0 0 0 0 0 0 0 0 0 0 0 0 0 0 0 0 0 0 0 0 0 0 0 0 0 0 0 0 0 0 0 0

K02722 0 0 0 0 0 0 0 0 0 0 0 0 0 0 0 0 0 0 0 0 0 0 0 0 0 0 0 0 0 0 0 0 0 0 0 0 0 0 0 0 0 0 0 0 0 0 0 0

K02723 0 0 0 0 0 0 0 0 0 0 0 0 0 0 0 0 0 0 0 0 0 0 0 0 0 0 0 0 0 0 0 0 0 0 0 0 0 0 0 0 0 0 0 0 0 0 0 0

K02724 0 0 0 0 0 0 0 0 0 0 0 0 0 0 0 0 0 0 0 0 0 0 0 0 0 0 0 0 0 0 0 0 0 0 0 0 0 0 0 0 0 0 0 0 0 0 0 0

K02742 0 0 0 0 0 0 0 0 0 0 0 0 0 0 0 0 0 0 0 0 0 0 0 0 0 0 0 0 0 0 0 0 0 0 0 0 0 0 0 0 0 0 0 0 0 0 0 0

K02744 0 0 0 0 0 0 0 0 0 0 0 0 0 0 0 0 0 0 0 0 0 0 0 0 0 0 0 0 0 0 0 0 0 0 0 0 0 0 0 0 0 0 0 0 0 0 0 0

K02745 0 0 0 0 0 0 0 0 0 0 0 0 0 0 0 0 0 0 0 0 0 0 0 0 0 0 0 0 0 0 0 0 0 0 0 0 0 0 0 0 0 0 0 0 0 0 0 0

K02746 0 0 0 0 0 0 0 0 0 0 0 0 0 0 0 0 0 0 0 0 0 0 0 0 0 0 0 0 0 0 0 0 0 0 0 0 0 0 0 0 0 0 0 0 0 0 0 0

K02747 0 0 0 0 0 0 0 0 0 0 0 0 0 0 0 0 0 0 0 0 0 0 0 0 0 0 0 0 0 0 0 0 0 0 0 0 0 0 0 0 0 0 0 0 0 0 0 0

K02749 0 0 0 0 0 0 0 0 0 0 0 0 0 0 0 0 0 0 0 0 0 0 0 0 0 0 0 0 0 0 0 0 0 0 0 0 0 0 0 0 0 0 0 0 0 0 0 0

K02750 0 0 0 0 0 0 0 0 0 0 0 0 0 0 0 0 0 0 0 0 0 0 0 0 0 0 0 0 0 0 0 0 0 0 0 0 0 0 0 0 0 0 0 0 0 0 0 0

K02752 0 0 0 0 0 0 0 0 0 0 0 0 0 0 0 0 0 0 0 0 0 0 0 0 0 0 0 0 0 0 0 0 0 0 0 0 0 0 0 0 0 0 0 0 0 0 0 0

K02753 0 0 0 0 0 0 0 0 0 0 0 0 0 0 0 0 0 0 0 0 0 0 0 0 0 0 0 0 0 0 0 0 0 0 0 0 0 0 0 0 0 0 0 0 0 0 0 0

K02755 0 0 0 0 0 0 0 0 0 0 0 0 0 0 0 0 0 0 0 0 0 0 0 0 0 0 0 0 0 0 0 0 0 0 0 0 0 0 0 0 0 0 0 0 0 0 0 0

K02756 0 0 0 0 0 0 0 0 0 0 0 0 0 0 0 0 0 0 0 0 0 0 0 0 0 0 0 0 0 0 0 0 0 0 0 0 0 0 0 0 0 0 0 0 0 0 0 0

K02757 0 0 0 0 0 0 0 0 0 0 0 0 0 0 0 0 0 0 0 0 0 0 0 0 0 0 0 0 0 0 0 0 0 0 0 0 0 0 0 0 0 0 0 0 0 0 0 0

K02759 0 0 0 0 0 0 0 0 0 0 0 0 0 0 0 0 0 0 0 0 0 0 0 0 0 0 0 0 0 0 0 0 0 0 0 0 0 0 0 0 0 0 0 0 0 0 0 0

K02760 0 0 0 0 0 0 0 0 0 0 0 0 0 0 0 0 0 0 0 0 0 0 0 0 0 0 0 0 0 0 0 0 0 0 0 0 0 0 0 0 0 0 0 0 0 0 0 0

K02761 0 0 0 0 0 0 0 0 0 0 0 0 0 0 0 0 0 0 0 0 0 0 0 0 0 0 0 0 0 0 0 0 0 0 0 0 0 0 0 0 0 0 0 0 0 0 0 0

K02763 0 0 0 0 0 0 0 0 0 0 0 0 0 0 0 0 0 0 0 0 0 0 0 0 0 0 0 0 0 0 0 0 0 0 0 0 0 0 0 0 0 0 0 0 0 0 0 0

K02764 0 0 0 0 0 0 0 0 0 0 0 0 0 0 0 0 0 0 0 0 0 0 0 0 0 0 0 0 0 0 0 0 0 0 0 0 0 0 0 0 0 0 0 0 0 0 0 0

K02765 0 0 0 0 0 0 0 0 0 0 0 0 0 0 0 0 0 0 0 0 0 0 0 0 0 0 0 0 0 0 0 0 0 0 0 0 0 0 0 0 0 0 0 0 0 0 0 0

K02768 0 0 0 0 0 0 0 0 0 0 0 0 0 0 0 0 0 0 0 0 0 0 0 0 0 0 0 0 0 0 0 0 0 0 0 0 0 0 0 0 0 0 0 0 0 0 0 0

K02769 0 0 0 0 0 0 0 0 0 0 0 0 0 0 0 0 0 0 0 0 0 0 0 0 0 0 0 0 0 0 0 0 0 0 0 0 0 0 0 0 0 0 0 0 0 0 0 0

K02770 0 0 0 0 0 0 0 0 0 0 0 0 0 0 0 0 0 0 0 0 0 0 0 0 0 0 0 0 0 0 0 0 0 0 0 0 0 0 0 0 0 0 0 0 0 0 0 0

K02771 0 0 0 0 0 0 0 0 0 0 0 0 0 0 0 0 0 0 0 0 0 0 0 0 0 0 0 0 0 0 0 0 0 0 0 0 0 0 0 0 0 0 0 0 0 0 0 0

K02773 0 0 0 0 0 0 0 0 0 0 0 0 0 0 0 0 0 0 0 0 0 0 0 0 0 0 0 0 0 0 0 0 0 0 0 0 0 0 0 0 0 0 0 0 0 0 0 0

K02774 0 0 0 0 0 0 0 0 0 0 0 0 0 0 0 0 0 0 0 0 0 0 0 0 0 0 0 0 0 0 0 0 0 0 0 0 0 0 0 0 0 0 0 0 0 0 0 0

K02775 0 0 0 0 0 0 0 0 0 0 0 0 0 0 0 0 0 0 0 0 0 0 0 0 0 0 0 0 0 0 0 0 0 0 0 0 0 0 0 0 0 0 0 0 0 0 0 0

K02777 0 0 0 0 0 0 0 0 0 0 0 0 0 0 0 0 0 0 0 0 0 0 0 0 0 0 0 0 0 0 0 0 0 0 0 0 0 0 0 0 0 0 0 0 0 0 0 0

K02778 0 0 0 0 0 0 0 0 0 0 0 0 0 0 0 0 0 0 0 0 0 0 0 0 0 0 0 0 0 0 0 0 0 0 0 0 0 0 0 0 0 0 0 0 0 0 0 0

K02779 0 0 0 0 0 0 0 0 0 0 0 0 0 0 0 0 0 0 0 0 0 0 0 0 0 0 0 0 0 0 0 0 0 0 0 0 0 0 0 0 0 0 0 0 0 0 0 0

K02781 0 0 0 0 0 0 0 0 0 0 0 0 0 0 0 0 0 0 0 0 0 0 0 0 0 0 0 0 0 0 0 0 0 0 0 0 0 0 0 0 0 0 0 0 0 0 0 0

K02782 0 0 0 0 0 0 0 0 0 0 0 0 0 0 0 0 0 0 0 0 0 0 0 0 0 0 0 0 0 0 0 0 0 0 0 0 0 0 0 0 0 0 0 0 0 0 0 0

K02783 0 0 0 0 0 0 0 0 0 0 0 0 0 0 0 0 0 0 0 0 0 0 0 0 0 0 0 0 0 0 0 0 0 0 0 0 0 0 0 0 0 0 0 0 0 0 0 0

K02784 0 0 0 0 0 0 0 0 0 0 0 0 0 0 0 0 0 0 0 0 0 0 0 0 0 0 0 0 0 0 0 0 0 0 0 0 0 0 0 0 0 0 0 0 0 0 0 0

K02786 0 0 0 0 0 0 0 0 0 0 0 0 0 0 0 0 0 0 0 0 0 0 0 0 0 0 0 0 0 0 0 0 0 0 0 0 0 0 0 0 0 0 0 0 0 0 0 0

K02787 0 0 0 0 0 0 0 0 0 0 0 0 0 0 0 0 0 0 0 0 0 0 0 0 0 0 0 0 0 0 0 0 0 0 0 0 0 0 0 0 0 0 0 0 0 0 0 0

K02788 0 0 0 0 0 0 0 0 0 0 0 0 0 0 0 0 0 0 0 0 0 0 0 0 0 0 0 0 0 0 0 0 0 0 0 0 0 0 0 0 0 0 0 0 0 0 0 0

K02790 0 0 0 0 0 0 0 0 0 0 0 0 0 0 0 0 0 0 0 0 0 0 0 0 0 0 0 0 0 0 0 0 0 0 0 0 0 0 0 0 0 0 0 0 0 0 0 0

K02791 0 0 0 0 0 0 0 0 0 0 0 0 0 0 0 0 0 0 0 0 0 0 0 0 0 0 0 0 0 0 0 0 0 0 0 0 0 0 0 0 0 0 0 0 0 0 0 0

K02793 0 0 0 0 0 0 0 0 0 0 0 0 0 0 0 0 0 0 0 0 0 0 0 0 0 0 0 0 0 0 0 0 0 0 0 0 0 0 0 0 0 0 0 0 0 0 0 0

K02794 0 0 0 0 0 0 0 0 0 0 0 0 0 0 0 0 0 0 0 0 0 0 0 0 0 0 0 0 0 0 0 0 0 0 0 0 0 0 0 0 0 0 0 0 0 0 0 0

K02795 0 0 0 0 0 0 0 0 0 0 0 0 0 0 0 0 0 0 0 0 0 0 0 0 0 0 0 0 0 0 0 0 0 0 0 0 0 0 0 0 0 0 0 0 0 0 0 0

K02796 0 0 0 0 0 0 0 0 0 0 0 0 0 0 0 0 0 0 0 0 0 0 0 0 0 0 0 0 0 0 0 0 0 0 0 0 0 0 0 0 0 0 0 0 0 0 0 0

K02798 0 0 0 0 0 0 0 0 0 0 0 0 0 0 0 0 0 0 0 0 0 0 0 0 0 0 0 0 0 0 0 0 0 0 0 0 0 0 0 0 0 0 0 0 0 0 0 0

K02799 0 0 0 0 0 0 0 0 0 0 0 0 0 0 0 0 0 0 0 0 0 0 0 0 0 0 0 0 0 0 0 0 0 0 0 0 0 0 0 0 0 0 0 0 0 0 0 0

K02800 0 0 0 0 0 0 0 0 0 0 0 0 0 0 0 0 0 0 0 0 0 0 0 0 0 0 0 0 0 0 0 0 0 0 0 0 0 0 0 0 0 0 0 0 0 0 0 0

K02802 0 0 0 0 0 0 0 0 0 0 0 0 0 0 0 0 0 0 0 0 0 0 0 0 0 0 0 0 0 0 0 0 0 0 0 0 0 0 0 0 0 0 0 0 0 0 0 0

K02803 0 0 0 0 0 0 0 0 0 0 0 0 0 0 0 0 0 0 0 0 0 0 0 0 0 0 0 0 0 0 0 0 0 0 0 0 0 0 0 0 0 0 0 0 0 0 0 0

K02804 0 0 0 0 0 0 0 0 0 0 0 0 0 0 0 0 0 0 0 0 0 0 0 0 0 0 0 0 0 0 0 0 0 0 0 0 0 0 0 0 0 0 0 0 0 0 0 0

K02805 0 0 0 0 0 0 0 0 0 0 0 0 0 0 0 0 0 0 0 0 0 0 0 0 0 0 0 0 0 0 0 0 0 0 0 0 0 0 0 0 0 0 0 0 0 0 0 0

K02806 0 0 0 0 0 0 0 0 0 0 0 0 0 0 0 0 0 0 0 0 0 0 0 0 0 0 0 0 0 0 0 0 0 0 0 0 0 0 0 0 0 0 0 0 0 0 0 0

K02808 0 0 0 0 0 0 0 0 0 0 0 0 0 0 0 0 0 0 0 0 0 0 0 0 0 0 0 0 0 0 0 0 0 0 0 0 0 0 0 0 0 0 0 0 0 0 0 0

K02809 0 0 0 0 0 0 0 0 0 0 0 0 0 0 0 0 0 0 0 0 0 0 0 0 0 0 0 0 0 0 0 0 0 0 0 0 0 0 0 0 0 0 0 0 0 0 0 0

K02810 0 0 0 0 0 0 0 0 0 0 0 0 0 0 0 0 0 0 0 0 0 0 0 0 0 0 0 0 0 0 0 0 0 0 0 0 0 0 0 0 0 0 0 0 0 0 0 0

K02812 0 0 0 0 0 0 0 0 0 0 0 0 0 0 0 0 0 0 0 0 0 0 0 0 0 0 0 0 0 0 0 0 0 0 0 0 0 0 0 0 0 0 0 0 0 0 0 0

K02813 0 0 0 0 0 0 0 0 0 0 0 0 0 0 0 0 0 0 0 0 0 0 0 0 0 0 0 0 0 0 0 0 0 0 0 0 0 0 0 0 0 0 0 0 0 0 0 0

K02814 0 0 0 0 0 0 0 0 0 0 0 0 0 0 0 0 0 0 0 0 0 0 0 0 0 0 0 0 0 0 0 0 0 0 0 0 0 0 0 0 0 0 0 0 0 0 0 0

K02815 0 0 0 0 0 0 0 0 0 0 0 0 0 0 0 0 0 0 0 0 0 0 0 0 0 0 0 0 0 0 0 0 0 0 0 0 0 0 0 0 0 0 0 0 0 0 0 0

K02817 0 0 0 0 0 0 0 0 0 0 0 0 0 0 0 0 0 0 0 0 0 0 0 0 0 0 0 0 0 0 0 0 0 0 0 0 0 0 0 0 0 0 0 0 0 0 0 0

K02818 0 0 0 0 0 0 0 0 0 0 0 0 0 0 0 0 0 0 0 0 0 0 0 0 0 0 0 0 0 0 0 0 0 0 0 0 0 0 0 0 0 0 0 0 0 0 0 0

K02819 0 0 0 0 0 0 0 0 0 0 0 0 0 0 0 0 0 0 0 0 0 0 0 0 0 0 0 0 0 0 0 0 0 0 0 0 0 0 0 0 0 0 0 0 0 0 0 0

K02821 0 0 0 0 0 0 0 0 0 0 0 0 0 0 0 0 0 0 0 0 0 0 0 0 0 0 0 0 0 0 0 0 0 0 0 0 0 0 0 0 0 0 0 0 0 0 0 0

K02822 0 0 0 0 0 0 0 0 0 0 0 0 0 0 0 0 0 0 0 0 0 0 0 0 0 0 0 0 0 0 0 0 0 0 0 0 0 0 0 0 0 0 0 0 0 0 0 0

K02823 478 724 645 262 978 798 404 1219 768 818 1142 968 386 926 719 320 919 1382 748 822 1088 1127 1335 1064 473 471 472 308 458 1206 24 1038 648 866 1094 934 308 759 916 668 952 984 510 1170 1161 658 788 1134

K02824 33 18 73 32 228 170 52 308 40 98 156 45 18 37 78 20 89 181 48 14 66 133 84 136 1 26 13 0 21 222 0 8 28 42 40 28 28 24 66 106 152 105 0 78 72 140 43 173

K02825 0 0 0 0 0 0 0 0 0 0 0 0 0 0 0 0 0 0 0 0 0 0 0 0 0 0 0 0 0 0 0 0 0 0 0 0 0 0 0 0 0 0 0 0 0 0 0 0

K02826 0 0 0 0 0 0 0 0 0 0 0 0 0 0 0 0 0 0 0 0 0 0 0 0 0 0 0 0 0 0 0 0 0 0 0 0 0 0 0 0 0 0 0 0 0 0 0 0

K02827 0 0 0 0 0 0 0 0 0 0 0 0 0 0 0 0 0 0 0 0 0 0 0 0 0 0 0 0 0 0 0 0 0 0 0 0 0 0 0 0 0 0 0 0 0 0 0 0

K02828 0 0 0 0 0 0 0 0 0 0 0 0 0 0 0 0 0 0 0 0 0 0 0 0 0 0 0 0 0 0 0 0 0 0 0 0 0 0 0 0 0 0 0 0 0 0 0 0

K02829 0 0 0 0 0 0 0 0 0 0 0 0 0 0 0 0 0 0 0 0 0 0 0 0 0 0 0 0 0 0 0 0 0 0 0 0 0 0 0 0 0 0 0 0 0 0 0 0

K02834 338 420 344 166 406 320 267 564 552 574 661 728 268 601 372 202 508 856 526 476 648 784 838 582 354 338 346 218 292 502 24 648 436 638 772 654 212 438 429 340 452 498 327 580 572 350 470 533

K02835 338 420 344 166 406 320 267 564 552 574 661 728 268 601 372 202 508 856 526 476 648 784 838 582 354 338 346 218 292 502 24 648 436 638 772 654 212 438 429 340 452 498 327 580 572 350 470 533

K02836 338 420 344 166 406 320 267 564 552 574 661 728 268 601 372 202 508 856 526 476 648 784 838 582 354 338 346 218 292 502 24 648 436 638 772 654 212 438 429 340 452 498 327 580 572 350 470 533

K02837 0 0 0 0 0 0 0 0 0 0 0 0 0 0 0 0 0 0 0 0 0 0 0 0 0 0 0 0 0 0 0 0 0 0 0 0 0 0 0 0 0 0 0 0 0 0 0 0

K02838 338 420 344 166 406 320 267 564 552 574 661 728 268 601 372 202 508 856 526 476 648 784 838 582 354 338 346 218 292 502 24 648 436 638 772 654 212 438 429 340 452 498 327 580 572 350 470 533

K02839 0 0 0 0 0 0 0 0 0 0 0 0 0 0 0 0 0 0 0 0 0 0 0 0 0 0 0 0 0 0 0 0 0 0 0 0 0 0 0 0 0 0 0 0 0 0 0 0

K02840 0 0 0 0 0 0 0 0 0 0 0 0 0 0 0 0 0 0 0 0 0 0 0 0 0 0 0 0 0 0 0 0 0 0 0 0 0 0 0 0 0 0 0 0 0 0 0 0

K02841 0 0 0 0 0 0 0 0 0 0 0 0 0 0 0 0 0 0 0 0 0 0 0 0 0 0 0 0 0 0 0 0 0 0 0 0 0 0 0 0 0 0 0 0 0 0 0 0

K02843 338 420 344 166 406 320 267 564 552 574 661 728 268 601 372 202 508 856 526 476 648 784 838 582 354 338 346 218 292 502 24 648 436 638 772 654 212 438 429 340 452 498 327 580 572 350 470 533

K02844 0 0 0 0 0 0 0 0 0 0 0 0 0 0 0 0 0 0 0 0 0 0 0 0 0 0 0 0 0 0 0 0 0 0 0 0 0 0 0 0 0 0 0 0 0 0 0 0

K02846 0 0 0 0 0 0 0 0 0 0 0 0 0 0 0 0 0 0 0 0 0 0 0 0 0 0 0 0 0 0 0 0 0 0 0 0 0 0 0 0 0 0 0 0 0 0 0 0

K02847 0 0 0 0 0 0 0 0 0 0 0 0 0 0 0 0 0 0 0 0 0 0 0 0 0 0 0 0 0 0 0 0 0 0 0 0 0 0 0 0 0 0 0 0 0 0 0 0

K02848 0 0 0 0 0 0 0 0 0 0 0 0 0 0 0 0 0 0 0 0 0 0 0 0 0 0 0 0 0 0 0 0 0 0 0 0 0 0 0 0 0 0 0 0 0 0 0 0

K02849 0 0 0 0 0 0 0 0 0 0 0 0 0 0 0 0 0 0 0 0 0 0 0 0 0 0 0 0 0 0 0 0 0 0 0 0 0 0 0 0 0 0 0 0 0 0 0 0

K02850 0 0 0 0 0 0 0 0 0 0 0 0 0 0 0 0 0 0 0 0 0 0 0 0 0 0 0 0 0 0 0 0 0 0 0 0 0 0 0 0 0 0 0 0 0 0 0 0

K02851 0 0 0 0 0 0 0 0 0 0 0 0 0 0 0 0 0 0 0 0 0 0 0 0 0 0 0 0 0 0 0 0 0 0 0 0 0 0 0 0 0 0 0 0 0 0 0 0

K02852 0 0 0 0 0 0 0 0 0 0 0 0 0 0 0 0 0 0 0 0 0 0 0 0 0 0 0 0 0 0 0 0 0 0 0 0 0 0 0 0 0 0 0 0 0 0 0 0

K02853 0 0 0 0 0 0 0 0 0 0 0 0 0 0 0 0 0 0 0 0 0 0 0 0 0 0 0 0 0 0 0 0 0 0 0 0 0 0 0 0 0 0 0 0 0 0 0 0

K02854 0 0 0 0 0 0 0 0 0 0 0 0 0 0 0 0 0 0 0 0 0 0 0 0 0 0 0 0 0 0 0 0 0 0 0 0 0 0 0 0 0 0 0 0 0 0 0 0

K02855 0 0 0 0 0 0 0 0 0 0 0 0 0 0 0 0 0 0 0 0 0 0 0 0 0 0 0 0 0 0 0 0 0 0 0 0 0 0 0 0 0 0 0 0 0 0 0 0

K02856 0 0 0 0 0 0 0 0 0 0 0 0 0 0 0 0 0 0 0 0 0 0 0 0 0 0 0 0 0 0 0 0 0 0 0 0 0 0 0 0 0 0 0 0 0 0 0 0

K02858 140 304 301 96 572 477 136 656 216 244 481 240 119 325 347 119 410 526 223 347 439 342 496 482 120 132 126 90 166 704 0 391 212 228 322 280 96 320 486 328 500 486 182 590 590 308 318 602

K02859 0 0 0 0 0 0 0 0 0 0 0 0 0 0 0 0 0 0 0 0 0 0 0 0 0 0 0 0 0 0 0 0 0 0 0 0 0 0 0 0 0 0 0 0 0 0 0 0

K02860 0 0 0 0 0 0 0 0 0 0 0 0 0 0 0 0 0 0 0 0 0 0 0 0 0 0 0 0 0 0 0 0 0 0 0 0 0 0 0 0 0 0 0 0 0 0 0 0

K02862 0 0 0 0 0 0 0 0 0 0 0 0 0 0 0 0 0 0 0 0 0 0 0 0 0 0 0 0 0 0 0 0 0 0 0 0 0 0 0 0 0 0 0 0 0 0 0 0

K02863 478 724 645 262 978 798 404 1219 768 818 1142 968 386 926 719 320 919 1382 748 822 1088 1127 1335 1064 473 471 472 308 458 1206 24 1038 648 866 1094 934 308 759 916 668 952 984 510 1170 1161 658 788 1134

K02864 478 724 645 262 978 798 404 1219 768 818 1142 968 386 926 719 320 919 1382 748 822 1088 1127 1335 1064 473 471 472 308 458 1206 24 1038 648 866 1094 934 308 759 916 668 952 984 510 1170 1161 658 788 1134

K02866 140 304 301 96 572 477 136 656 216 244 481 240 119 325 347 119 410 526 223 347 439 342 496 482 120 132 126 90 166 704 0 391 212 228 322 280 96 320 486 328 500 486 182 590 590 308 318 602

K02867 478 724 645 262 978 798 404 1219 768 818 1142 968 386 926 719 320 919 1382 748 822 1088 1127 1335 1064 473 471 472 308 458 1206 24 1038 648 866 1094 934 308 759 916 668 952 984 510 1170 1161 658 788 1134

K02869 140 304 301 96 572 477 136 656 216 244 481 240 119 325 347 119 410 526 223 347 439 342 496 482 120 132 126 90 166 704 0 391 212 228 322 280 96 320 486 328 500 486 182 590 590 308 318 602

K02871 478 724 645 262 971 798 395 1219 768 818 1142 968 386 926 703 320 919 1382 748 822 1088 1127 1335 1050 473 471 472 308 458 1206 24 1038 648 866 1094 934 308 759 916 668 952 976 510 1170 1161 658 788 1134

K02873 0 0 0 0 0 0 0 0 0 0 0 0 0 0 0 0 0 0 0 0 0 0 0 0 0 0 0 0 0 0 0 0 0 0 0 0 0 0 0 0 0 0 0 0 0 0 0 0

K02874 478 724 645 262 978 798 404 1219 768 818 1142 968 386 926 719 320 919 1382 748 822 1088 1127 1335 1064 473 471 472 308 458 1206 24 1038 648 866 1094 934 308 759 916 668 952 984 510 1170 1161 658 788 1134

K02875 99 104 142 47 390 314 72 502 110 124 253 86 28 194 188 74 176 303 59 142 204 186 263 250 35 30 32 71 49 444 0 196 144 161 140 143 37 85 192 171 298 242 103 254 208 184 134 358

K02876 478 724 645 262 978 798 404 1219 768 818 1142 968 386 926 719 320 919 1382 748 822 1088 1127 1335 1064 473 471 472 308 458 1206 24 1038 648 866 1094 934 308 759 916 668 952 984 510 1170 1161 658 788 1134

K02877 140 304 301 96 572 477 136 656 216 244 481 240 119 325 347 119 410 526 223 347 439 342 496 482 120 132 126 90 166 704 0 391 212 228 322 280 96 320 486 328 500 486 182 590 590 308 318 602

K02878 338 420 344 166 406 320 267 564 552 574 661 728 268 601 372 202 508 856 526 476 648 784 838 582 354 338 346 218 292 502 24 648 436 638 772 654 212 438 429 340 452 498 327 580 572 350 470 533

K02879 338 420 344 166 406 320 267 564 552 574 661 728 268 601 372 202 508 856 526 476 648 784 838 582 354 338 346 218 292 502 24 648 436 638 772 654 212 438 429 340 452 498 327 580 572 350 470 533

K02881 478 724 645 262 978 798 404 1219 768 818 1142 968 386 926 719 320 919 1382 748 822 1088 1127 1335 1064 473 471 472 308 458 1206 24 1038 648 866 1094 934 308 759 916 668 952 984 510 1170 1161 658 788 1134

K02883 140 304 301 96 572 477 136 656 216 244 481 240 119 325 347 119 410 526 223 347 439 342 496 482 120 132 126 90 166 704 0 391 212 228 322 280 96 320 486 328 500 486 182 590 590 308 318 602

K02884 338 420 344 166 406 320 267 564 552 574 661 728 268 601 372 202 508 856 526 476 648 784 838 582 354 338 346 218 292 502 24 648 436 638 772 654 212 438 429 340 452 498 327 580 572 350 470 533

K02885 140 304 301 96 572 477 136 656 216 244 481 240 119 325 347 119 410 526 223 347 439 342 496 482 120 132 126 90 166 704 0 391 212 228 322 280 96 320 486 328 500 486 182 590 590 308 318 602

K02886 478 724 645 262 978 798 404 1219 768 818 1142 968 386 926 719 320 919 1382 748 822 1088 1127 1335 1064 473 471 472 308 458 1206 24 1038 648 866 1094 934 308 759 916 668 952 984 510 1170 1161 658 788 1134

K02887 338 420 344 166 406 320 267 564 552 574 661 728 268 601 372 202 508 856 526 476 648 784 838 582 354 338 346 218 292 502 24 648 436 638 772 654 212 438 429 340 452 498 327 580 572 350 470 533

K02888 338 420 344 166 406 320 267 564 552 574 661 728 268 601 372 202 508 856 526 476 648 784 838 582 354 338 346 218 292 502 24 648 436 638 772 654 212 438 429 340 452 498 327 580 572 350 470 533

K02889 140 304 301 96 572 477 136 656 216 244 481 240 119 325 347 119 410 526 223 347 439 342 496 482 120 132 126 90 166 704 0 391 212 228 322 280 96 320 486 328 500 486 182 590 590 308 318 602

K02890 478 724 645 262 978 798 404 1219 768 818 1142 968 386 926 719 320 919 1382 748 822 1088 1127 1335 1064 473 471 472 308 458 1206 24 1038 648 866 1094 934 308 759 916 668 952 984 510 1170 1161 658 788 1134

K02892 478 724 645 262 978 798 404 1219 768 818 1142 968 386 926 719 320 919 1382 748 822 1088 1127 1335 1064 473 471 472 308 458 1206 24 1038 648 866 1094 934 308 759 916 668 952 984 510 1170 1161 658 788 1134

K02895 478 724 645 262 978 798 404 1219 768 818 1142 968 386 926 719 320 919 1382 748 822 1088 1127 1335 1064 473 471 472 308 458 1206 24 1038 648 866 1094 934 308 759 916 668 952 984 510 1170 1161 658 788 1134

K02896 140 304 301 96 572 477 136 656 216 244 481 240 119 325 347 119 410 526 223 347 439 342 496 482 120 132 126 90 166 704 0 391 212 228 322 280 96 320 486 328 500 486 182 590 590 308 318 602

K02897 0 0 0 0 0 0 0 0 0 0 0 0 0 0 0 0 0 0 0 0 0 0 0 0 0 0 0 0 0 0 0 0 0 0 0 0 0 0 0 0 0 0 0 0 0 0 0 0

K02899 338 420 344 166 406 320 267 564 552 574 661 728 268 601 372 202 508 856 526 476 648 784 838 582 354 338 346 218 292 502 24 648 436 638 772 654 212 438 429 340 452 498 327 580 572 350 470 533

K02902 338 420 344 166 406 320 267 564 552 574 661 728 268 601 372 202 508 856 526 476 648 784 838 582 354 338 346 218 292 502 24 648 436 638 772 654 212 438 429 340 452 498 327 580 572 350 470 533

K02904 478 724 645 262 978 798 404 1219 768 818 1142 968 386 926 719 320 919 1382 748 822 1088 1127 1335 1064 473 471 472 308 458 1206 24 1038 648 866 1094 934 308 759 916 668 952 984 510 1170 1161 658 788 1134

K02906 478 724 645 262 978 798 404 1219 768 818 1142 968 386 926 719 320 919 1382 748 822 1088 1127 1335 1064 473 471 472 308 458 1206 24 1038 648 866 1094 934 308 759 916 668 952 984 510 1170 1161 658 788 1134

K02907 478 724 645 262 978 798 404 1219 768 818 1142 968 386 926 719 320 919 1382 748 822 1088 1127 1335 1064 473 471 472 308 458 1206 24 1038 648 866 1094 934 308 759 916 668 952 984 510 1170 1161 658 788 1134

K02908 140 304 301 96 595 493 136 664 216 244 481 240 119 325 347 119 410 540 223 347 439 342 496 488 120 132 126 90 166 717 0 391 212 228 322 280 96 320 486 328 500 486 182 590 590 308 318 602

K02909 338 420 344 166 406 320 267 564 552 574 661 728 268 601 372 202 508 856 526 476 648 784 838 582 354 338 346 218 292 502 24 648 436 638 772 654 212 438 429 340 452 498 327 580 572 350 470 533

K02910 140 304 301 96 572 477 136 656 216 244 481 240 119 325 347 119 410 526 223 347 439 342 496 482 120 132 126 90 166 704 0 391 212 228 322 280 96 320 486 328 500 486 182 590 590 308 318 602

K02911 338 420 344 166 406 320 267 564 552 574 661 728 268 601 372 202 508 856 526 476 648 784 838 582 354 338 346 218 292 502 24 648 436 638 772 654 212 438 429 340 452 498 327 580 572 350 470 533

K02912 140 304 301 96 572 477 136 656 216 244 481 240 119 325 347 119 410 526 223 347 439 342 496 482 120 132 126 90 166 704 0 391 212 228 322 280 96 320 486 328 500 486 182 590 590 308 318 602

K02913 338 420 344 166 406 320 267 564 552 574 661 728 268 601 372 202 508 856 526 476 648 784 838 582 354 338 346 218 292 502 24 648 436 638 772 654 212 438 429 340 452 498 327 580 572 350 470 533

K02914 0 0 0 0 0 0 0 0 0 0 0 0 0 0 0 0 0 0 0 0 0 0 0 0 0 0 0 0 0 0 0 0 0 0 0 0 0 0 0 0 0 0 0 0 0 0 0 0

K02915 99 104 142 47 390 314 72 502 110 124 253 86 28 194 188 74 176 303 59 142 204 186 263 250 35 30 32 71 49 444 0 196 144 161 140 143 37 85 192 171 298 242 103 254 208 184 134 358

K02916 338 420 344 166 406 320 267 564 552 574 661 728 268 601 372 202 508 856 526 476 648 784 838 582 354 338 346 218 292 502 24 648 436 638 772 654 212 438 429 340 452 498 327 580 572 350 470 533

K02917 0 0 0 0 0 0 0 0 0 0 0 0 0 0 0 0 0 0 0 0 0 0 0 0 0 0 0 0 0 0 0 0 0 0 0 0 0 0 0 0 0 0 0 0 0 0 0 0

K02919 0 0 0 0 0 0 0 0 0 0 0 0 0 0 0 0 0 0 0 0 0 0 0 0 0 0 0 0 0 0 0 0 0 0 0 0 0 0 0 0 0 0 0 0 0 0 0 0

K02921 140 304 301 96 572 477 136 656 216 244 481 240 119 325 347 119 410 526 223 347 439 342 496 482 120 132 126 90 166 704 0 391 212 228 322 280 96 320 486 328 500 486 182 590 590 308 318 602

K02922 140 304 301 96 572 477 136 656 216 244 481 240 119 325 347 119 410 526 223 347 439 342 496 482 120 132 126 90 166 704 0 391 212 228 322 280 96 320 486 328 500 486 182 590 590 308 318 602

K02924 140 304 301 96 572 477 136 656 216 244 481 240 119 325 347 119 410 526 223 347 439 342 496 482 120 132 126 90 166 704 0 391 212 228 322 280 96 320 486 328 500 486 182 590 590 308 318 602

K02926 338 420 344 166 406 320 267 564 552 574 661 728 268 601 372 202 508 856 526 476 648 784 838 582 354 338 346 218 292 502 24 648 436 638 772 654 212 438 429 340 452 498 327 580 572 350 470 533

K02927 140 304 301 96 572 477 136 656 216 244 481 240 119 325 347 119 410 526 223 347 439 342 496 482 120 132 126 90 166 704 0 391 212 228 322 280 96 320 486 328 500 486 182 590 590 308 318 602

K02928 0 0 0 0 0 0 0 0 0 0 0 0 0 0 0 0 0 0 0 0 0 0 0 0 0 0 0 0 0 0 0 0 0 0 0 0 0 0 0 0 0 0 0 0 0 0 0 0

K02929 140 304 301 96 572 477 136 656 216 244 481 240 119 325 347 119 410 526 223 347 439 342 496 482 120 132 126 90 166 704 0 391 212 228 322 280 96 320 486 328 500 486 182 590 590 308 318 602

K02930 140 304 301 96 572 477 136 656 216 244 481 240 119 325 347 119 410 526 223 347 439 342 496 482 120 132 126 90 166 704 0 391 212 228 322 280 96 320 486 328 500 486 182 590 590 308 318 602

K02931 478 724 645 262 978 798 404 1219 768 818 1142 968 386 926 719 320 919 1382 748 822 1088 1127 1335 1064 473 471 472 308 458 1206 24 1038 648 866 1094 934 308 759 916 668 952 984 510 1170 1161 658 788 1134

K02933 478 724 645 262 978 798 404 1219 768 818 1142 968 386 926 719 320 919 1382 748 822 1088 1127 1335 1064 473 471 472 308 458 1206 24 1038 648 866 1094 934 308 759 916 668 952 984 510 1170 1161 658 788 1134

K02935 338 420 344 166 406 320 267 564 552 574 661 728 268 601 372 202 508 856 526 476 648 784 838 582 354 338 346 218 292 502 24 648 436 638 772 654 212 438 429 340 452 498 327 580 572 350 470 533

K02936 140 304 301 96 572 477 136 656 216 244 481 240 119 325 347 119 410 526 223 347 439 342 496 482 120 132 126 90 166 704 0 391 212 228 322 280 96 320 486 328 500 486 182 590 590 308 318 602

K02939 338 420 344 166 406 320 267 564 552 574 661 728 268 601 372 202 508 856 526 476 648 784 838 582 354 338 346 218 292 502 24 648 436 638 772 654 212 438 429 340 452 498 327 580 572 350 470 533

K02942 0 0 0 0 0 0 0 0 0 0 0 0 0 0 0 0 0 0 0 0 0 0 0 0 0 0 0 0 0 0 0 0 0 0 0 0 0 0 0 0 0 0 0 0 0 0 0 0

K02944 140 304 301 96 572 477 136 656 216 244 481 240 119 325 347 119 410 526 223 347 439 342 496 482 120 132 126 90 166 704 0 391 212 228 322 280 96 320 486 328 500 486 182 590 590 308 318 602

K02945 338 420 344 166 406 320 267 564 552 574 661 728 268 601 372 202 508 856 526 476 648 784 838 582 354 338 346 218 292 502 24 648 436 638 772 654 212 438 429 340 452 498 327 580 572 350 470 533

K02946 478 724 645 262 978 798 404 1219 768 818 1142 968 386 926 719 320 919 1382 748 822 1088 1127 1335 1064 473 471 472 308 458 1206 24 1038 648 866 1094 934 308 759 916 668 952 984 510 1170 1161 658 788 1134

K02948 478 724 645 262 978 798 404 1219 768 818 1142 968 386 926 719 320 919 1382 748 822 1088 1127 1335 1064 473 471 472 308 458 1206 24 1038 648 866 1094 934 308 759 916 668 952 984 510 1170 1161 658 788 1134

K02950 478 724 645 262 978 798 404 1219 768 818 1142 968 386 926 719 320 919 1382 748 822 1088 1127 1335 1064 473 471 472 308 458 1206 24 1038 648 866 1094 934 308 759 916 668 952 984 510 1170 1161 658 788 1134

K02952 478 724 645 262 978 798 404 1219 768 818 1142 968 386 926 719 320 919 1382 748 822 1088 1127 1335 1064 473 471 472 308 458 1206 24 1038 648 866 1094 934 308 759 916 668 952 984 510 1170 1161 658 788 1134

K02954 478 724 645 262 978 798 404 1219 768 818 1142 968 386 926 719 320 919 1382 748 822 1088 1127 1335 1064 473 471 472 308 458 1206 24 1038 648 866 1094 934 308 759 916 668 952 984 510 1170 1161 658 788 1134

K02956 478 724 645 262 978 798 404 1219 768 818 1142 968 386 926 719 320 919 1382 748 822 1088 1127 1335 1064 473 471 472 308 458 1206 24 1038 648 866 1094 934 308 759 916 668 952 984 510 1170 1161 658 788 1134

K02959 338 420 344 166 406 320 267 564 552 574 661 728 268 601 372 202 508 856 526 476 648 784 838 582 354 338 346 218 292 502 24 648 436 638 772 654 212 438 429 340 452 498 327 580 572 350 470 533

K02961 478 724 645 262 978 798 404 1219 768 818 1142 968 386 926 719 320 919 1382 748 822 1088 1127 1335 1064 473 471 472 308 458 1206 24 1038 648 866 1094 934 308 759 916 668 952 984 510 1170 1161 658 788 1134

K02962 140 304 301 96 573 477 136 656 216 244 481 240 119 325 347 119 410 526 223 347 439 342 496 482 120 132 126 90 166 704 0 391 212 228 322 280 96 320 486 328 500 486 182 590 590 308 318 602

K02963 338 420 344 166 406 320 267 564 552 574 661 728 268 601 372 202 508 856 526 476 648 784 838 582 354 338 346 218 292 502 24 648 436 638 772 654 212 438 429 340 452 498 327 580 572 350 470 533

K02965 478 724 645 262 978 798 404 1219 768 818 1142 968 386 926 719 320 919 1382 748 822 1088 1127 1335 1064 473 471 472 308 458 1206 24 1038 648 866 1094 934 308 759 916 668 952 984 510 1170 1161 658 788 1134

K02966 140 304 301 96 572 477 136 656 216 244 481 240 119 325 347 119 410 526 223 347 439 342 496 482 120 132 126 90 166 704 0 391 212 228 322 280 96 320 486 328 500 486 182 590 590 308 318 602

K02967 478 724 645 262 978 798 404 1219 768 818 1142 968 386 926 719 320 919 1382 748 822 1088 1127 1335 1064 473 471 472 308 458 1206 24 1038 648 866 1094 934 308 759 916 668 952 984 510 1170 1161 658 788 1134

K02968 338 420 344 166 406 320 267 564 552 574 661 728 268 601 372 202 508 856 526 476 648 784 838 582 354 338 346 218 292 502 24 648 436 638 772 654 212 438 429 340 452 498 327 580 572 350 470 533

K02970 338 420 344 166 406 320 267 564 552 574 661 728 268 601 372 202 508 856 526 476 648 784 838 582 354 338 346 218 292 502 24 648 436 638 772 654 212 438 429 340 452 498 327 580 572 350 470 533

K02972 0 0 0 0 0 0 0 0 0 0 0 0 0 0 0 0 0 0 0 0 0 0 0 0 0 0 0 0 0 0 0 0 0 0 0 0 0 0 0 0 0 0 0 0 0 0 0 0

K02974 140 304 301 96 595 493 136 664 216 244 481 240 119 325 347 119 410 540 223 347 439 342 496 488 120 132 126 90 166 717 0 394 212 228 322 280 96 320 486 328 500 486 182 590 590 308 318 602

K02975 0 0 0 0 0 0 0 0 0 0 0 0 0 0 0 0 0 0 0 0 0 0 0 0 0 0 0 0 0 0 0 0 0 0 0 0 0 0 0 0 0 0 0 0 0 0 0 0

K02976 0 0 0 0 0 0 0 0 0 0 0 0 0 0 0 0 0 0 0 0 0 0 0 0 0 0 0 0 0 0 0 0 0 0 0 0 0 0 0 0 0 0 0 0 0 0 0 0

K02977 140 304 301 96 572 477 136 656 216 244 481 240 119 325 347 119 410 526 223 347 439 342 496 482 120 132 126 90 166 704 0 391 212 228 322 280 96 320 486 328 500 486 182 590 590 308 318 602

K02978 140 304 301 96 572 477 136 656 216 244 481 240 119 325 347 119 410 526 223 347 439 342 496 482 120 132 126 90 166 704 0 391 212 228 322 280 96 320 486 328 500 486 182 590 590 308 318 602

K02979 140 304 301 96 572 477 136 656 216 244 481 240 119 325 347 119 410 526 223 347 439 342 496 482 120 132 126 90 166 704 0 391 212 228 322 280 96 320 486 328 500 486 182 590 590 308 318 602

K02982 478 724 645 262 978 798 404 1219 768 818 1142 968 386 926 719 320 919 1382 748 822 1088 1127 1335 1064 473 471 472 308 458 1206 24 1038 648 866 1094 934 308 759 916 668 952 984 510 1170 1161 658 788 1134

K02983 0 0 0 0 0 0 0 0 0 0 0 0 0 0 0 0 0 0 0 0 0 0 0 0 0 0 0 0 0 0 0 0 0 0 0 0 0 0 0 0 0 0 0 0 0 0 0 0

K02984 140 304 301 96 572 477 136 656 216 244 481 240 119 325 347 119 410 526 223 347 439 342 496 482 120 132 126 90 166 704 0 391 212 228 322 280 96 320 486 328 500 486 182 590 590 308 318 602

K02986 478 724 645 262 978 798 404 1219 768 818 1142 968 386 926 719 320 919 1382 748 822 1088 1127 1335 1064 473 471 472 308 458 1206 24 1038 648 866 1094 934 308 759 916 668 952 984 510 1170 1161 658 788 1134

K02987 140 304 301 96 572 477 136 656 216 244 481 240 119 325 347 119 410 526 223 347 439 342 496 482 120 132 126 90 166 704 0 391 212 228 322 280 96 320 486 328 500 486 182 590 590 308 318 602

K02988 478 724 645 262 978 798 404 1219 768 818 1142 968 386 926 719 320 919 1382 748 822 1088 1127 1335 1064 473 471 472 308 458 1206 24 1038 648 866 1094 934 308 759 916 668 952 984 510 1170 1161 658 788 1134

K02990 338 420 344 166 406 320 267 564 552 574 661 728 268 601 372 202 508 856 526 476 648 784 838 582 354 338 346 218 292 502 24 648 436 638 772 654 212 438 429 340 452 498 327 580 572 350 470 533

K02991 140 304 301 96 572 477 136 656 216 244 481 240 119 325 347 119 410 526 223 347 439 342 496 482 120 132 126 90 166 704 0 391 212 228 322 280 96 320 486 328 500 486 182 590 590 308 318 602

K02992 478 724 645 262 978 798 404 1219 768 818 1142 968 386 926 719 320 919 1382 748 822 1088 1127 1335 1064 473 471 472 308 458 1206 24 1038 648 866 1094 934 308 759 916 668 952 984 510 1170 1161 658 788 1134

K02994 478 724 645 262 978 798 404 1219 768 818 1142 968 386 926 719 320 919 1382 748 822 1088 1127 1335 1064 473 471 472 308 458 1206 24 1038 648 866 1094 934 308 759 916 668 952 984 510 1170 1161 658 788 1134

K02995 140 304 301 96 572 477 136 656 216 244 481 240 119 325 347 119 410 526 223 347 439 342 496 482 120 132 126 90 166 704 0 391 212 228 322 280 96 320 486 328 500 486 182 590 590 308 318 602

K02996 478 724 645 262 978 798 404 1219 768 818 1142 968 386 926 719 320 919 1382 748 822 1088 1127 1335 1064 473 471 472 308 458 1206 24 1038 648 866 1094 934 308 759 916 668 952 984 510 1170 1161 658 788 1134

K03014 0 0 0 0 0 0 0 0 0 0 0 0 0 0 0 0 0 0 0 0 0 0 0 0 0 0 0 0 0 0 0 0 0 0 0 0 0 0 0 0 0 0 0 0 0 0 0 0

K03027 0 0 0 0 0 0 0 0 0 0 0 0 0 0 0 0 0 0 0 0 0 0 0 0 0 0 0 0 0 0 0 0 0 0 0 0 0 0 0 0 0 0 0 0 0 0 0 0

K03039 0 0 0 0 0 0 0 0 0 0 0 0 0 0 0 0 0 0 0 0 0 0 0 0 0 0 0 0 0 0 0 0 0 0 0 0 0 0 0 0 0 0 0 0 0 0 0 0

K03040 338 420 344 166 406 320 267 564 552 574 661 728 268 601 372 202 508 856 526 476 648 784 838 582 354 338 346 218 292 502 24 648 436 638 772 654 212 438 429 340 452 498 327 580 572 350 470 533

K03041 140 304 301 96 602 493 145 664 216 244 481 240 119 325 363 119 410 540 223 347 439 342 496 502 120 132 126 90 166 717 0 391 212 228 322 280 96 320 486 328 500 494 182 590 590 308 318 602

K03042 140 304 301 96 595 493 136 664 216 244 481 240 119 325 347 119 410 540 223 347 439 342 496 488 120 132 126 90 166 717 0 391 212 228 322 280 96 320 486 328 500 486 182 590 590 308 318 602

K03043 338 420 344 166 406 320 267 564 552 574 661 728 268 601 372 202 508 856 526 476 648 784 838 582 354 338 346 218 292 502 24 648 436 638 772 654 212 438 429 340 452 498 327 580 572 350 470 533

K03044 99 104 142 47 436 346 72 520 110 124 256 86 28 194 188 74 176 331 59 142 204 186 263 280 35 30 32 71 49 474 0 226 144 161 140 143 37 85 192 171 298 242 103 254 208 184 134 358

K03045 99 104 142 47 436 346 72 520 110 124 256 86 28 194 188 74 176 331 59 142 204 186 263 280 35 30 32 71 49 474 0 226 144 161 140 143 37 85 192 171 298 242 103 254 208 184 134 358

K03046 338 420 344 166 406 320 267 564 552 574 661 728 268 601 372 202 508 856 526 476 648 784 838 582 354 338 346 218 292 502 24 648 436 638 772 654 212 438 429 340 452 498 327 580 572 350 470 533

K03047 140 304 301 96 572 477 136 656 216 244 481 240 119 325 347 119 410 526 223 347 439 342 496 482 120 132 126 90 166 704 0 391 212 228 322 280 96 320 486 328 500 486 182 590 590 308 318 602

K03048 0 0 0 0 0 0 0 0 0 0 0 0 0 0 0 0 0 0 0 0 0 0 0 0 0 0 0 0 0 0 0 0 0 0 0 0 0 0 0 0 0 0 0 0 0 0 0 0

K03049 140 304 301 96 572 477 136 656 216 244 481 240 119 325 347 119 410 526 223 347 439 342 496 482 120 132 126 90 166 704 0 391 212 228 322 280 96 320 486 328 500 486 182 590 590 308 318 602

K03050 140 304 301 96 572 477 136 656 216 244 481 240 119 325 347 119 410 526 223 347 439 342 496 482 120 132 126 90 166 704 0 391 212 228 322 280 96 320 486 328 500 486 182 590 590 308 318 602

K03051 140 304 301 96 572 477 136 656 216 244 481 240 119 325 347 119 410 526 223 347 439 342 496 482 120 132 126 90 166 704 0 391 212 228 322 280 96 320 486 328 500 486 182 590 590 308 318 602

K03052 0 0 0 0 0 0 0 0 0 0 0 0 0 0 0 0 0 0 0 0 0 0 0 0 0 0 0 0 0 0 0 0 0 0 0 0 0 0 0 0 0 0 0 0 0 0 0 0

K03053 140 304 301 96 595 493 136 664 216 244 481 240 119 325 347 119 410 540 223 347 439 342 496 488 120 132 126 90 166 717 0 391 212 228 322 280 96 320 486 328 500 486 182 590 590 308 318 602

K03054 0 0 0 0 0 0 0 0 0 0 0 0 0 0 0 0 0 0 0 0 0 0 0 0 0 0 0 0 0 0 0 0 0 0 0 0 0 0 0 0 0 0 0 0 0 0 0 0

K03055 140 304 301 96 572 477 136 656 216 244 481 240 119 325 347 119 410 526 223 347 439 342 496 482 120 132 126 90 166 704 0 391 212 228 322 280 96 320 486 328 500 486 182 590 590 308 318 602

K03056 140 304 301 96 572 477 136 656 216 244 481 240 119 325 347 119 410 526 223 347 439 342 496 482 120 132 126 90 166 704 0 391 212 228 322 280 96 320 486 328 500 486 182 590 590 308 318 602

K03057 193 344 408 143 824 676 200 994 300 368 673 325 147 397 454 146 512 734 282 391 544 493 620 654 131 162 147 99 204 978 0 442 263 292 404 357 132 375 576 450 668 630 210 694 686 452 376 832

K03058 140 304 301 96 572 477 136 656 216 244 481 240 119 325 347 119 410 526 223 347 439 342 496 482 120 132 126 90 166 704 0 391 212 228 322 280 96 320 486 328 500 486 182 590 590 308 318 602

K03059 99 104 142 47 414 330 72 511 110 124 256 86 28 194 188 74 176 317 59 142 204 186 263 274 35 30 32 71 49 461 0 226 144 161 140 143 37 85 192 171 298 242 103 254 208 184 134 358

K03060 0 0 0 0 0 0 0 0 0 0 0 0 0 0 0 0 0 0 0 0 0 0 0 0 0 0 0 0 0 0 0 0 0 0 0 0 0 0 0 0 0 0 0 0 0 0 0 0

K03065 0 0 0 0 0 0 0 0 0 0 0 0 0 0 0 0 0 0 0 0 0 0 0 0 0 0 0 0 0 0 0 0 0 0 0 0 0 0 0 0 0 0 0 0 0 0 0 0

K03070 338 420 344 166 406 320 267 564 552 574 661 728 268 601 372 202 508 856 526 476 648 784 838 582 354 338 346 218 292 502 24 648 436 638 772 654 212 438 429 340 452 498 327 580 572 350 470 533

K03071 0 0 0 0 0 0 0 0 0 0 0 0 0 0 0 0 0 0 0 0 0 0 0 0 0 0 0 0 0 0 0 0 0 0 0 0 0 0 0 0 0 0 0 0 0 0 0 0

K03072 437 524 486 214 819 650 340 1074 662 698 917 814 296 794 560 275 685 1173 584 618 853 970 1102 855 388 368 378 290 342 963 24 874 580 799 912 798 249 524 620 511 750 740 430 833 780 533 604 891

K03073 338 420 344 166 406 320 267 564 552 574 661 728 268 601 372 202 508 856 526 476 648 784 838 582 354 338 346 218 292 502 24 648 436 638 772 654 212 438 429 340 452 498 327 580 572 350 470 533

K03074 437 524 486 214 819 650 340 1074 662 698 917 814 296 794 560 275 685 1173 584 618 853 970 1102 855 388 368 378 290 342 963 24 874 580 799 912 798 249 524 620 511 750 740 430 833 780 533 604 891

K03075 338 420 344 166 406 320 267 564 552 574 661 728 268 601 372 202 508 856 526 476 648 784 838 582 354 338 346 218 292 502 24 648 436 638 772 654 212 438 429 340 452 498 327 580 572 350 470 533

K03076 478 724 645 262 978 798 404 1219 768 818 1142 968 386 926 719 320 919 1382 748 822 1088 1127 1335 1064 473 471 472 308 458 1206 24 1038 648 866 1094 934 308 759 916 668 952 984 510 1170 1161 658 788 1134

K03077 0 0 0 0 0 0 0 0 0 0 0 0 0 0 0 0 0 0 0 0 0 0 0 0 0 0 0 0 0 0 0 0 0 0 0 0 0 0 0 0 0 0 0 0 0 0 0 0

K03078 0 0 0 0 0 0 0 0 0 0 0 0 0 0 0 0 0 0 0 0 0 0 0 0 0 0 0 0 0 0 0 0 0 0 0 0 0 0 0 0 0 0 0 0 0 0 0 0

K03079 0 0 0 0 0 0 0 0 0 0 0 0 0 0 0 0 0 0 0 0 0 0 0 0 0 0 0 0 0 0 0 0 0 0 0 0 0 0 0 0 0 0 0 0 0 0 0 0

K03080 0 0 0 0 0 0 0 0 0 0 0 0 0 0 0 0 0 0 0 0 0 0 0 0 0 0 0 0 0 0 0 0 0 0 0 0 0 0 0 0 0 0 0 0 0 0 0 0

K03081 0 0 0 0 0 0 0 0 0 0 0 0 0 0 0 0 0 0 0 0 0 0 0 0 0 0 0 0 0 0 0 0 0 0 0 0 0 0 0 0 0 0 0 0 0 0 0 0

K03082 0 0 0 0 0 0 0 0 0 0 0 0 0 0 0 0 0 0 0 0 0 0 0 0 0 0 0 0 0 0 0 0 0 0 0 0 0 0 0 0 0 0 0 0 0 0 0 0

K03086 338 420 344 166 406 320 267 564 552 574 661 728 268 601 372 202 508 856 526 476 648 784 838 582 354 338 346 218 292 502 24 648 436 638 772 654 212 438 429 340 452 498 327 580 572 350 470 533

K03087 0 0 0 0 0 0 0 0 0 0 0 0 0 0 0 0 0 0 0 0 0 0 0 0 0 0 0 0 0 0 0 0 0 0 0 0 0 0 0 0 0 0 0 0 0 0 0 0

K03088 676 839 688 333 811 641 534 1127 1103 1148 1322 1457 535 1202 744 403 1017 1712 1051 951 1297 1569 1677 1163 707 677 692 437 585 1004 48 1295 872 1276 1545 1309 424 877 858 680 904 997 654 1159 1143 699 941 1066

K03089 0 0 0 0 0 0 0 0 0 0 0 0 0 0 0 0 0 0 0 0 0 0 0 0 0 0 0 0 0 0 0 0 0 0 0 0 0 0 0 0 0 0 0 0 0 0 0 0

K03090 0 0 0 0 0 0 0 0 0 0 0 0 0 0 0 0 0 0 0 0 0 0 0 0 0 0 0 0 0 0 0 0 0 0 0 0 0 0 0 0 0 0 0 0 0 0 0 0

K03091 0 0 0 0 0 0 0 0 0 0 0 0 0 0 0 0 0 0 0 0 0 0 0 0 0 0 0 0 0 0 0 0 0 0 0 0 0 0 0 0 0 0 0 0 0 0 0 0

K03092 0 0 0 0 0 0 0 0 0 0 0 0 0 0 0 0 0 0 0 0 0 0 0 0 0 0 0 0 0 0 0 0 0 0 0 0 0 0 0 0 0 0 0 0 0 0 0 0

K03093 0 0 0 0 0 0 0 0 0 0 0 0 0 0 0 0 0 0 0 0 0 0 0 0 0 0 0 0 0 0 0 0 0 0 0 0 0 0 0 0 0 0 0 0 0 0 0 0

K03095 0 0 0 0 0 0 0 0 0 0 0 0 0 0 0 0 0 0 0 0 0 0 0 0 0 0 0 0 0 0 0 0 0 0 0 0 0 0 0 0 0 0 0 0 0 0 0 0

K03098 0 0 0 0 0 0 0 0 0 0 0 0 0 0 0 0 0 0 0 0 0 0 0 0 0 0 0 0 0 0 0 0 0 0 0 0 0 0 0 0 0 0 0 0 0 0 0 0

K03100 338 420 344 166 406 320 267 564 552 574 661 728 268 601 372 202 508 856 526 476 648 784 838 582 354 338 346 218 292 502 24 648 436 638 772 654 212 438 429 340 452 498 327 580 572 350 470 533

K03101 338 420 344 166 406 320 267 564 552 574 661 728 268 601 372 202 508 856 526 476 648 784 838 582 354 338 346 218 292 502 24 648 436 638 772 654 212 438 429 340 452 498 327 580 572 350 470 533

K03105 140 304 301 96 572 477 136 656 216 244 481 240 119 325 347 119 410 526 223 347 439 342 496 482 120 132 126 90 166 704 0 391 212 228 322 280 96 320 486 328 500 486 182 590 590 308 318 602

K03106 478 724 645 262 978 798 404 1219 768 818 1142 968 386 926 719 320 919 1382 748 822 1088 1127 1335 1064 473 471 472 308 458 1206 24 1038 648 866 1094 934 308 759 916 668 952 984 510 1170 1161 658 788 1134

K03110 478 724 645 262 978 798 404 1219 768 818 1142 968 386 926 719 320 919 1382 748 822 1088 1127 1335 1064 473 471 472 308 458 1206 24 1038 648 866 1094 934 308 759 916 668 952 984 510 1170 1161 658 788 1134

K03111 338 420 344 166 406 320 267 564 552 574 661 728 268 601 372 202 508 856 526 476 648 784 838 582 354 338 346 218 292 502 24 648 436 638 772 654 212 438 429 340 452 498 327 580 572 350 470 533

K03112 0 0 0 0 0 0 0 0 0 0 0 0 0 0 0 0 0 0 0 0 0 0 0 0 0 0 0 0 0 0 0 0 0 0 0 0 0 0 0 0 0 0 0 0 0 0 0 0

K03113 478 724 645 262 978 798 404 1219 768 818 1142 968 386 926 719 320 919 1382 748 822 1088 1127 1335 1064 473 471 472 308 458 1206 24 1038 648 866 1094 934 308 759 916 668 952 984 510 1170 1161 658 788 1134

K03116 370 484 379 166 536 435 267 727 578 574 722 728 268 722 436 248 584 951 526 555 748 820 978 646 377 338 358 280 304 672 24 794 530 711 829 720 212 469 531 389 582 586 402 730 684 390 545 660

K03117 0 0 0 0 0 0 0 0 0 0 0 0 0 0 0 0 0 0 0 0 0 0 0 0 0 0 0 0 0 0 0 0 0 0 0 0 0 0 0 0 0 0 0 0 0 0 0 0

K03118 338 420 344 166 406 320 267 564 552 574 661 728 268 601 372 202 508 856 526 476 648 784 838 582 354 338 346 218 292 502 24 648 436 638 772 654 212 438 429 340 452 498 327 580 572 350 470 533

K03119 0 0 0 0 0 0 0 0 0 0 0 0 0 0 0 0 0 0 0 0 0 0 0 0 0 0 0 0 0 0 0 0 0 0 0 0 0 0 0 0 0 0 0 0 0 0 0 0

K03120 140 304 301 96 597 493 136 664 216 244 486 240 119 325 347 119 410 540 223 347 439 342 496 522 120 132 126 90 166 726 0 454 212 228 322 280 96 320 486 328 500 486 182 590 590 308 318 602

K03124 586 1006 912 344 1472 1213 519 1826 940 1036 1581 1167 496 1216 1021 433 1318 1854 960 1122 1486 1452 1792 1467 582 599 590 390 606 1828 24 1354 838 1048 1373 1165 394 1050 1378 980 1436 1421 664 1736 1726 963 1090 1678

K03136 113 104 142 47 414 330 72 511 110 124 256 86 28 194 188 74 176 317 59 160 204 186 263 274 35 30 32 71 49 461 0 226 144 186 140 143 37 85 192 171 298 242 103 254 208 184 134 358

K03146 108 286 228 64 366 323 85 357 176 146 327 194 100 288 268 98 322 360 176 332 374 210 412 352 118 107 113 90 144 495 0 386 184 186 281 252 67 296 421 222 347 380 182 512 518 168 274 428

K03147 545 764 752 310 1260 1013 476 1566 852 942 1336 1054 414 998 842 348 1020 1604 808 885 1193 1278 1458 1272 484 501 493 318 496 1498 24 1119 699 954 1177 1012 344 814 1005 790 1120 1138 538 1273 1257 802 847 1365

K03148 0 0 0 0 0 0 0 0 0 0 0 0 0 0 0 0 0 0 0 0 0 0 0 0 0 0 0 0 0 0 0 0 0 0 0 0 0 0 0 0 0 0 0 0 0 0 0 0

K03149 0 0 0 0 0 0 0 0 0 0 0 0 0 0 0 0 0 0 0 0 0 0 0 0 0 0 0 0 0 0 0 0 0 0 0 0 0 0 0 0 0 0 0 0 0 0 0 0

K03150 0 0 0 0 0 0 0 0 0 0 0 0 0 0 0 0 0 0 0 0 0 0 0 0 0 0 0 0 0 0 0 0 0 0 0 0 0 0 0 0 0 0 0 0 0 0 0 0

K03151 478 724 645 262 978 798 404 1219 768 818 1144 968 386 926 719 320 919 1382 748 822 1088 1127 1335 1064 473 471 472 308 458 1206 24 1038 648 866 1094 934 308 759 916 668 952 984 510 1170 1161 658 788 1134

K03152 0 0 0 0 0 0 0 0 0 0 0 0 0 0 0 0 0 0 0 0 0 0 0 0 0 0 0 0 0 0 0 0 0 0 0 0 0 0 0 0 0 0 0 0 0 0 0 0

K03153 0 0 0 0 0 0 0 0 0 0 0 0 0 0 0 0 0 0 0 0 0 0 0 0 0 0 0 0 0 0 0 0 0 0 0 0 0 0 0 0 0 0 0 0 0 0 0 0

K03154 404 460 451 214 665 520 340 902 636 698 853 814 296 673 496 228 610 1064 584 538 754 935 962 767 365 368 367 228 330 776 24 698 486 726 856 732 249 493 518 462 620 652 355 682 668 493 530 764

K03162 0 0 0 0 0 0 0 0 0 0 0 0 0 0 0 0 0 0 0 0 0 0 0 0 0 0 0 0 0 0 0 0 0 0 0 0 0 0 0 0 0 0 0 0 0 0 0 0

K03163 0 0 0 0 0 0 0 0 0 0 0 0 0 0 0 0 0 0 0 0 0 0 0 0 0 0 0 0 0 0 0 0 0 0 0 0 0 0 0 0 0 0 0 0 0 0 0 0

K03166 140 304 301 96 572 477 136 656 216 244 481 240 119 325 347 119 410 526 223 347 439 342 496 482 120 132 126 90 166 704 0 391 212 228 322 280 96 320 486 328 500 486 182 590 590 308 318 602

K03167 140 304 301 96 572 477 136 656 216 244 481 240 119 325 347 119 410 526 223 347 439 342 496 482 120 132 126 90 166 704 0 391 212 228 322 280 96 320 486 328 500 486 182 590 590 308 318 602

K03168 478 724 645 262 978 798 404 1219 768 818 1142 968 386 926 719 320 919 1382 748 822 1088 1127 1335 1064 473 471 472 308 458 1206 24 1038 648 866 1094 934 308 759 916 668 952 984 510 1170 1161 658 788 1134

K03169 0 0 0 0 0 0 0 0 0 0 0 0 0 0 0 0 0 0 0 0 0 0 0 0 0 0 0 0 0 0 0 0 0 0 0 0 0 0 0 0 0 0 0 0 0 0 0 0

K03170 444 860 678 239 900 746 356 1116 775 758 1046 986 394 1006 756 340 1028 1349 713 932 1175 1036 1364 954 506 441 474 362 460 1215 24 1180 745 874 1182 957 270 766 1054 717 1089 1076 618 1428 1360 594 910 1086

K03177 478 724 645 262 978 798 404 1219 768 818 1142 968 386 926 719 320 919 1382 748 822 1088 1127 1335 1064 473 471 472 308 458 1206 24 1035 648 866 1094 934 308 759 916 668 952 984 510 1170 1161 658 788 1134

K03179 478 724 645 262 978 798 404 1219 768 818 1142 968 386 926 719 320 919 1382 748 822 1088 1127 1335 1064 473 471 472 308 458 1206 24 1042 648 866 1094 934 308 759 916 668 952 984 510 1170 1161 658 788 1134

K03181 0 0 0 0 0 0 0 0 0 0 0 0 0 0 0 0 0 0 0 0 0 0 0 0 0 0 0 0 0 0 0 0 0 0 0 0 0 0 0 0 0 0 0 0 0 0 0 0

K03182 358 442 378 182 452 366 280 604 595 600 700 769 277 636 401 208 520 897 537 505 688 802 878 624 364 343 354 228 310 567 24 693 458 659 815 704 220 468 453 356 468 538 355 604 596 353 486 590

K03183 792 1304 1041 431 1259 1050 662 1580 1340 1387 1833 1765 717 1586 1127 540 1560 2226 1379 1440 1865 1918 2284 1645 900 882 891 537 829 1660 48 1770 1102 1483 1966 1649 541 1378 1550 1043 1436 1573 888 1984 2018 989 1384 1680

K03184 0 0 0 0 0 0 0 0 0 0 0 0 0 0 0 0 0 0 0 0 0 0 0 0 0 0 0 0 0 0 0 0 0 0 0 0 0 0 0 0 0 0 0 0 0 0 0 0

K03185 0 0 0 0 0 0 0 0 0 0 0 0 0 0 0 0 0 0 0 0 0 0 0 0 0 0 0 0 0 0 0 0 0 0 0 0 0 0 0 0 0 0 0 0 0 0 0 0

K03186 478 724 645 262 978 798 404 1219 768 818 1142 968 386 926 719 320 919 1382 748 822 1088 1127 1335 1064 473 471 472 308 458 1206 24 1038 648 866 1094 934 308 759 916 668 952 984 510 1170 1161 658 788 1134

K03187 0 0 0 0 0 0 0 0 0 0 0 0 0 0 0 0 0 0 0 0 0 0 0 0 0 0 0 0 0 0 0 0 0 0 0 0 0 0 0 0 0 0 0 0 0 0 0 0

K03188 0 0 0 0 0 0 0 0 0 0 0 0 0 0 0 0 0 0 0 0 0 0 0 0 0 0 0 0 0 0 0 0 0 0 0 0 0 0 0 0 0 0 0 0 0 0 0 0

K03189 0 0 0 0 0 0 0 0 0 0 0 0 0 0 0 0 0 0 0 0 0 0 0 0 0 0 0 0 0 0 0 0 0 0 0 0 0 0 0 0 0 0 0 0 0 0 0 0

K03190 0 0 0 0 0 0 0 0 0 0 0 0 0 0 0 0 0 0 0 0 0 0 0 0 0 0 0 0 0 0 0 0 0 0 0 0 0 0 0 0 0 0 0 0 0 0 0 0

K03191 0 0 0 0 0 0 0 0 0 0 0 0 0 0 0 0 0 0 0 0 0 0 0 0 0 0 0 0 0 0 0 0 0 0 0 0 0 0 0 0 0 0 0 0 0 0 0 0

K03192 0 0 0 0 0 0 0 0 0 0 0 0 0 0 0 0 0 0 0 0 0 0 0 0 0 0 0 0 0 0 0 0 0 0 0 0 0 0 0 0 0 0 0 0 0 0 0 0

K03194 0 0 0 0 0 0 0 0 0 0 0 0 0 0 0 0 0 0 0 0 0 0 0 0 0 0 0 0 0 0 0 0 0 0 0 0 0 0 0 0 0 0 0 0 0 0 0 0

K03195 0 0 0 0 0 0 0 0 0 0 0 0 0 0 0 0 0 0 0 0 0 0 0 0 0 0 0 0 0 0 0 0 0 0 0 0 0 0 0 0 0 0 0 0 0 0 0 0

K03196 0 0 0 0 0 0 0 0 0 0 0 0 0 0 0 0 0 0 0 0 0 0 0 0 0 0 0 0 0 0 0 0 0 0 0 0 0 0 0 0 0 0 0 0 0 0 0 0

K03197 0 0 0 0 0 0 0 0 0 0 0 0 0 0 0 0 0 0 0 0 0 0 0 0 0 0 0 0 0 0 0 0 0 0 0 0 0 0 0 0 0 0 0 0 0 0 0 0

K03198 0 0 0 0 0 0 0 0 0 0 0 0 0 0 0 0 0 0 0 0 0 0 0 0 0 0 0 0 0 0 0 0 0 0 0 0 0 0 0 0 0 0 0 0 0 0 0 0

K03199 0 0 0 0 0 0 0 0 0 0 0 0 0 0 0 0 0 0 0 0 0 0 0 0 0 0 0 0 0 0 0 0 0 0 0 0 0 0 0 0 0 0 0 0 0 0 0 0

K03200 0 0 0 0 0 0 0 0 0 0 0 0 0 0 0 0 0 0 0 0 0 0 0 0 0 0 0 0 0 0 0 0 0 0 0 0 0 0 0 0 0 0 0 0 0 0 0 0

K03201 0 0 0 0 0 0 0 0 0 0 0 0 0 0 0 0 0 0 0 0 0 0 0 0 0 0 0 0 0 0 0 0 0 0 0 0 0 0 0 0 0 0 0 0 0 0 0 0

K03202 0 0 0 0 0 0 0 0 0 0 0 0 0 0 0 0 0 0 0 0 0 0 0 0 0 0 0 0 0 0 0 0 0 0 0 0 0 0 0 0 0 0 0 0 0 0 0 0

K03203 0 0 0 0 0 0 0 0 0 0 0 0 0 0 0 0 0 0 0 0 0 0 0 0 0 0 0 0 0 0 0 0 0 0 0 0 0 0 0 0 0 0 0 0 0 0 0 0

K03204 0 0 0 0 0 0 0 0 0 0 0 0 0 0 0 0 0 0 0 0 0 0 0 0 0 0 0 0 0 0 0 0 0 0 0 0 0 0 0 0 0 0 0 0 0 0 0 0

K03205 0 0 0 0 0 0 0 0 0 0 0 0 0 0 0 0 0 0 0 0 0 0 0 0 0 0 0 0 0 0 0 0 0 0 0 0 0 0 0 0 0 0 0 0 0 0 0 0

K03206 0 0 0 0 0 0 0 0 0 0 0 0 0 0 0 0 0 0 0 0 0 0 0 0 0 0 0 0 0 0 0 0 0 0 0 0 0 0 0 0 0 0 0 0 0 0 0 0

K03207 0 0 0 0 0 0 0 0 0 0 0 0 0 0 0 0 0 0 0 0 0 0 0 0 0 0 0 0 0 0 0 0 0 0 0 0 0 0 0 0 0 0 0 0 0 0 0 0

K03208 0 0 0 0 0 0 0 0 0 0 0 0 0 0 0 0 0 0 0 0 0 0 0 0 0 0 0 0 0 0 0 0 0 0 0 0 0 0 0 0 0 0 0 0 0 0 0 0

K03210 0 0 0 0 0 0 0 0 0 0 0 0 0 0 0 0 0 0 0 0 0 0 0 0 0 0 0 0 0 0 0 0 0 0 0 0 0 0 0 0 0 0 0 0 0 0 0 0

K03212 0 0 0 0 0 0 0 0 0 0 0 0 0 0 0 0 0 0 0 0 0 0 0 0 0 0 0 0 0 0 0 0 0 0 0 0 0 0 0 0 0 0 0 0 0 0 0 0

K03214 0 0 0 0 0 0 0 0 0 0 0 0 0 0 0 0 0 0 0 0 0 0 0 0 0 0 0 0 0 0 0 0 0 0 0 0 0 0 0 0 0 0 0 0 0 0 0 0

K03215 0 0 0 0 0 0 0 0 0 0 0 0 0 0 0 0 0 0 0 0 0 0 0 0 0 0 0 0 0 0 0 0 0 0 0 0 0 0 0 0 0 0 0 0 0 0 0 0

K03216 0 0 0 0 0 0 0 0 0 0 0 0 0 0 0 0 0 0 0 0 0 0 0 0 0 0 0 0 0 0 0 0 0 0 0 0 0 0 0 0 0 0 0 0 0 0 0 0

K03217 338 420 344 166 406 320 267 564 552 574 661 728 268 601 372 202 508 856 526 476 648 784 838 582 354 338 346 218 292 502 24 648 436 638 772 654 212 438 429 340 452 498 327 580 572 350 470 533

K03218 338 420 344 166 406 320 267 564 552 574 661 728 268 601 372 202 508 856 526 476 648 784 838 582 354 338 346 218 292 502 24 648 436 638 772 654 212 438 429 340 452 498 327 580 572 350 470 533

K03219 0 0 0 0 0 0 0 0 0 0 0 0 0 0 0 0 0 0 0 0 0 0 0 0 0 0 0 0 0 0 0 0 0 0 0 0 0 0 0 0 0 0 0 0 0 0 0 0

K03220 0 0 0 0 0 0 0 0 0 0 0 0 0 0 0 0 0 0 0 0 0 0 0 0 0 0 0 0 0 0 0 0 0 0 0 0 0 0 0 0 0 0 0 0 0 0 0 0

K03221 0 0 0 0 0 0 0 0 0 0 0 0 0 0 0 0 0 0 0 0 0 0 0 0 0 0 0 0 0 0 0 0 0 0 0 0 0 0 0 0 0 0 0 0 0 0 0 0

K03222 0 0 0 0 0 0 0 0 0 0 0 0 0 0 0 0 0 0 0 0 0 0 0 0 0 0 0 0 0 0 0 0 0 0 0 0 0 0 0 0 0 0 0 0 0 0 0 0

K03223 0 0 0 0 0 0 0 0 0 0 0 0 0 0 0 0 0 0 0 0 0 0 0 0 0 0 0 0 0 0 0 0 0 0 0 0 0 0 0 0 0 0 0 0 0 0 0 0

K03224 0 0 0 0 0 0 0 0 0 0 0 0 0 0 0 0 0 0 0 0 0 0 0 0 0 0 0 0 0 0 0 0 0 0 0 0 0 0 0 0 0 0 0 0 0 0 0 0

K03225 0 0 0 0 0 0 0 0 0 0 0 0 0 0 0 0 0 0 0 0 0 0 0 0 0 0 0 0 0 0 0 0 0 0 0 0 0 0 0 0 0 0 0 0 0 0 0 0

K03226 0 0 0 0 0 0 0 0 0 0 0 0 0 0 0 0 0 0 0 0 0 0 0 0 0 0 0 0 0 0 0 0 0 0 0 0 0 0 0 0 0 0 0 0 0 0 0 0

K03227 0 0 0 0 0 0 0 0 0 0 0 0 0 0 0 0 0 0 0 0 0 0 0 0 0 0 0 0 0 0 0 0 0 0 0 0 0 0 0 0 0 0 0 0 0 0 0 0

K03228 0 0 0 0 0 0 0 0 0 0 0 0 0 0 0 0 0 0 0 0 0 0 0 0 0 0 0 0 0 0 0 0 0 0 0 0 0 0 0 0 0 0 0 0 0 0 0 0

K03229 0 0 0 0 0 0 0 0 0 0 0 0 0 0 0 0 0 0 0 0 0 0 0 0 0 0 0 0 0 0 0 0 0 0 0 0 0 0 0 0 0 0 0 0 0 0 0 0

K03230 0 0 0 0 0 0 0 0 0 0 0 0 0 0 0 0 0 0 0 0 0 0 0 0 0 0 0 0 0 0 0 0 0 0 0 0 0 0 0 0 0 0 0 0 0 0 0 0

K03231 525 788 680 262 1115 912 412 1382 794 818 1203 968 386 1048 799 367 994 1478 748 920 1186 1162 1474 1142 496 471 484 370 469 1376 24 1184 742 964 1150 1000 308 790 1018 717 1082 1080 584 1320 1273 698 862 1262

K03232 140 304 301 96 572 477 136 656 216 244 481 240 119 325 347 119 410 526 223 347 439 342 496 482 120 132 126 90 166 704 0 391 212 228 322 280 96 320 486 328 500 486 182 590 590 308 318 602

K03234 174 322 374 128 777 631 188 954 256 342 634 284 138 362 426 140 500 694 270 362 504 476 580 612 120 158 139 90 186 913 0 396 240 270 362 308 124 345 552 434 652 590 182 669 661 448 360 774

K03236 140 304 301 96 596 493 136 664 216 244 483 240 119 325 347 119 410 540 223 347 439 342 496 505 120 132 126 90 166 722 0 418 212 228 322 280 96 320 486 328 500 486 182 590 590 308 318 602

K03237 140 304 301 96 572 477 136 656 216 244 481 240 119 325 347 119 410 526 223 347 439 342 496 482 120 132 126 90 166 704 0 391 212 228 322 280 96 320 486 328 500 486 182 590 590 308 318 602

K03238 140 304 301 96 572 477 136 656 216 244 481 240 119 325 347 119 410 526 223 347 439 342 496 482 120 132 126 90 166 704 0 391 212 228 322 280 96 320 486 328 500 486 182 590 590 308 318 602

K03239 0 0 0 0 6 0 8 0 0 0 0 0 0 0 16 0 0 0 0 0 0 0 0 14 0 0 0 0 0 0 0 0 0 0 0 0 0 0 0 0 0 8 0 0 0 0 0 0

K03242 140 304 301 96 572 477 136 656 216 244 481 240 119 325 347 119 410 526 223 347 439 342 496 482 120 132 126 90 166 704 0 391 212 228 322 280 96 320 486 328 500 486 182 590 590 308 318 602

K03243 140 304 301 96 572 477 136 656 216 244 481 240 119 325 347 119 410 526 223 347 439 342 496 482 120 132 126 90 166 704 0 391 212 228 322 280 96 320 486 328 500 486 182 590 590 308 318 602

K03263 140 304 301 96 572 477 136 656 216 244 481 240 119 325 347 119 410 526 223 347 439 342 496 482 120 132 126 90 166 704 0 391 212 228 322 280 96 320 486 328 500 486 182 590 590 308 318 602

K03264 140 304 301 96 572 477 136 656 216 244 481 240 119 325 347 119 410 526 223 347 439 342 496 482 120 132 126 90 166 704 0 391 212 228 322 280 96 320 486 328 500 486 182 590 590 308 318 602

K03265 140 304 301 96 572 477 136 656 216 244 481 240 119 325 347 119 410 526 223 347 439 342 496 482 120 132 126 90 166 704 0 391 212 228 322 280 96 320 486 328 500 486 182 590 590 308 318 602

K03268 0 0 0 0 0 0 0 0 0 0 0 0 0 0 0 0 0 0 0 0 0 0 0 0 0 0 0 0 0 0 0 0 0 0 0 0 0 0 0 0 0 0 0 0 0 0 0 0

K03269 0 0 0 0 0 0 0 0 0 0 0 0 0 0 0 0 0 0 0 0 0 0 0 0 0 0 0 0 0 0 0 0 0 0 0 0 0 0 0 0 0 0 0 0 0 0 0 0

K03270 0 0 0 0 0 0 0 0 0 0 0 0 0 0 0 0 0 0 0 0 0 0 0 0 0 0 0 0 0 0 0 0 0 0 0 0 0 0 0 0 0 0 0 0 0 0 0 0

K03271 0 0 0 0 0 0 0 0 0 0 0 0 0 0 0 0 0 0 0 0 0 0 0 0 0 0 0 0 0 0 0 0 0 0 0 0 0 0 0 0 0 0 0 0 0 0 0 0

K03272 0 0 0 0 0 0 0 0 0 0 0 0 0 0 0 0 0 0 0 0 0 0 0 0 0 0 0 0 0 0 0 0 0 0 0 0 0 0 0 0 0 0 0 0 0 0 0 0

K03273 0 0 0 0 0 0 0 0 0 0 0 0 0 0 0 0 0 0 0 0 0 0 0 0 0 0 0 0 0 0 0 0 0 0 0 0 0 0 0 0 0 0 0 0 0 0 0 0

K03274 0 0 0 0 0 0 0 0 0 0 0 0 0 0 0 0 0 0 0 0 0 0 0 0 0 0 0 0 0 0 0 0 0 0 0 0 0 0 0 0 0 0 0 0 0 0 0 0

K03275 0 0 0 0 0 0 0 0 0 0 0 0 0 0 0 0 0 0 0 0 0 0 0 0 0 0 0 0 0 0 0 0 0 0 0 0 0 0 0 0 0 0 0 0 0 0 0 0

K03276 0 0 0 0 0 0 0 0 0 0 0 0 0 0 0 0 0 0 0 0 0 0 0 0 0 0 0 0 0 0 0 0 0 0 0 0 0 0 0 0 0 0 0 0 0 0 0 0

K03277 0 0 0 0 0 0 0 0 0 0 0 0 0 0 0 0 0 0 0 0 0 0 0 0 0 0 0 0 0 0 0 0 0 0 0 0 0 0 0 0 0 0 0 0 0 0 0 0

K03278 0 0 0 0 0 0 0 0 0 0 0 0 0 0 0 0 0 0 0 0 0 0 0 0 0 0 0 0 0 0 0 0 0 0 0 0 0 0 0 0 0 0 0 0 0 0 0 0

K03279 0 0 0 0 0 0 0 0 0 0 0 0 0 0 0 0 0 0 0 0 0 0 0 0 0 0 0 0 0 0 0 0 0 0 0 0 0 0 0 0 0 0 0 0 0 0 0 0

K03280 0 0 0 0 0 0 0 0 0 0 0 0 0 0 0 0 0 0 0 0 0 0 0 0 0 0 0 0 0 0 0 0 0 0 0 0 0 0 0 0 0 0 0 0 0 0 0 0

K03281 338 420 344 166 430 336 267 572 552 574 664 728 268 601 372 202 508 870 526 476 648 784 838 604 354 338 346 218 292 520 24 678 436 638 772 654 212 438 429 340 452 498 327 580 572 350 470 533

K03282 0 0 0 0 2 0 0 0 0 0 3 0 0 0 0 0 0 0 0 0 0 0 0 0 0 0 0 0 0 0 0 0 0 0 0 0 0 0 0 0 0 0 0 0 0 0 0 0

K03284 380 620 503 216 611 500 331 726 657 694 891 882 358 732 532 247 742 1094 690 680 883 941 1072 836 438 441 440 238 409 780 24 872 504 705 954 792 270 674 724 497 653 742 406 916 953 474 654 776

K03285 0 0 0 0 0 0 0 0 0 0 0 0 0 0 0 0 0 0 0 0 0 0 0 0 0 0 0 0 0 0 0 0 0 0 0 0 0 0 0 0 0 0 0 0 0 0 0 0

K03286 0 0 0 0 0 0 0 0 0 0 0 0 0 0 0 0 0 0 0 0 0 0 0 0 0 0 0 0 0 0 0 0 0 0 0 0 0 0 0 0 0 0 0 0 0 0 0 0

K03287 0 0 0 0 0 0 0 0 0 0 0 0 0 0 0 0 0 0 0 0 0 0 0 0 0 0 0 0 0 0 0 0 0 0 0 0 0 0 0 0 0 0 0 0 0 0 0 0

K03288 0 0 0 0 0 0 0 0 0 0 0 0 0 0 0 0 0 0 0 0 0 0 0 0 0 0 0 0 0 0 0 0 0 0 0 0 0 0 0 0 0 0 0 0 0 0 0 0

K03289 0 0 0 0 0 0 0 0 0 0 0 0 0 0 0 0 0 0 0 0 0 0 0 0 0 0 0 0 0 0 0 0 0 0 0 0 0 0 0 0 0 0 0 0 0 0 0 0

K03290 0 0 0 0 0 0 0 0 0 0 0 0 0 0 0 0 0 0 0 0 0 0 0 0 0 0 0 0 0 0 0 0 0 0 0 0 0 0 0 0 0 0 0 0 0 0 0 0

K03291 0 0 0 0 0 0 0 0 0 0 0 0 0 0 0 0 0 0 0 0 0 0 0 0 0 0 0 0 0 0 0 0 0 0 0 0 0 0 0 0 0 0 0 0 0 0 0 0

K03292 0 0 0 0 0 0 0 0 0 0 0 0 0 0 0 0 0 0 0 0 0 0 0 0 0 0 0 0 0 0 0 0 0 0 0 0 0 0 0 0 0 0 0 0 0 0 0 0

K03293 0 0 0 0 0 0 0 0 0 0 0 0 0 0 0 0 0 0 0 0 0 0 0 0 0 0 0 0 0 0 0 0 0 0 0 0 0 0 0 0 0 0 0 0 0 0 0 0

K03294 370 484 379 166 562 451 267 736 578 574 725 728 268 722 436 248 584 965 526 555 748 820 978 670 377 338 358 280 304 689 24 824 530 711 829 720 212 469 531 389 582 586 402 730 684 390 545 660

K03295 0 0 0 0 1 0 0 0 0 0 0 0 0 0 0 0 0 0 0 0 0 0 0 0 0 0 0 0 0 0 0 4 0 0 0 0 0 0 0 0 0 0 0 0 0 0 0 0

K03296 338 420 344 166 406 320 267 564 552 574 661 728 268 601 372 202 508 856 526 476 648 784 838 582 354 338 346 218 292 502 24 648 436 638 772 654 212 438 429 340 452 498 327 580 572 350 470 533

K03297 33 18 73 32 205 154 52 298 40 98 154 45 18 37 78 20 89 167 48 14 66 133 84 130 1 26 13 0 21 209 0 5 28 42 40 28 28 24 66 106 152 105 0 78 72 140 43 173

K03298 0 0 0 0 0 0 0 0 0 0 0 0 0 0 0 0 0 0 0 0 0 0 0 0 0 0 0 0 0 0 0 0 0 0 0 0 0 0 0 0 0 0 0 0 0 0 0 0

K03299 0 0 0 0 0 0 0 0 0 0 0 0 0 0 0 0 0 0 0 0 0 0 0 0 0 0 0 0 0 0 0 0 0 0 0 0 0 0 0 0 0 0 0 0 0 0 0 0

K03300 0 0 0 0 0 0 0 0 0 0 0 0 0 0 0 0 0 0 0 0 0 0 0 0 0 0 0 0 0 0 0 0 0 0 0 0 0 0 0 0 0 0 0 0 0 0 0 0

K03301 0 0 0 0 0 0 0 0 0 0 0 0 0 0 0 0 0 0 0 0 0 0 0 0 0 0 0 0 0 0 0 0 0 0 0 0 0 0 0 0 0 0 0 0 0 0 0 0

K03302 0 0 0 0 0 0 0 0 0 0 0 0 0 0 0 0 0 0 0 0 0 0 0 0 0 0 0 0 0 0 0 0 0 0 0 0 0 0 0 0 0 0 0 0 0 0 0 0

K03303 0 0 0 0 1 0 0 0 0 0 0 0 0 0 0 0 0 0 0 0 0 0 0 0 0 0 0 0 0 0 0 0 0 0 0 0 0 0 0 0 0 0 0 0 0 0 0 0

K03304 0 0 0 0 0 0 0 0 0 0 0 0 0 0 0 0 0 0 0 0 0 0 0 0 0 0 0 0 0 0 0 0 0 0 0 0 0 0 0 0 0 0 0 0 0 0 0 0

K03305 0 0 0 0 0 0 0 0 0 0 0 0 0 0 0 0 0 0 0 0 0 0 0 0 0 0 0 0 0 0 0 0 0 0 0 0 0 0 0 0 0 0 0 0 0 0 0 0

K03306 426 795 642 239 801 647 364 962 748 758 990 986 394 884 708 294 953 1268 713 870 1076 1001 1225 943 482 441 462 300 450 1068 24 1094 651 826 1125 891 270 735 952 668 958 996 543 1277 1248 554 835 959

K03307 437 524 486 214 842 666 340 1084 662 698 919 814 296 794 560 275 685 1187 584 618 853 970 1102 878 388 368 378 290 342 980 24 904 580 799 912 798 249 524 620 511 750 740 430 833 780 533 604 891

K03308 204 134 433 190 1142 877 282 1582 290 544 844 306 112 255 450 116 469 931 260 132 408 700 499 757 26 136 81 18 139 1192 0 146 185 254 288 236 160 182 376 562 794 605 56 442 406 707 247 980

K03309 0 0 0 0 0 0 0 0 0 0 0 0 0 0 0 0 0 0 0 0 0 0 0 0 0 0 0 0 0 0 0 0 0 0 0 0 0 0 0 0 0 0 0 0 0 0 0 0

K03310 33 18 73 32 205 154 52 298 40 98 154 45 18 37 78 20 89 167 48 14 66 133 84 130 1 26 13 0 21 209 0 5 28 42 40 28 28 24 66 106 152 105 0 78 72 140 43 173

K03311 0 0 0 0 0 0 0 0 0 0 0 0 0 0 0 0 0 0 0 0 0 0 0 0 0 0 0 0 0 0 0 0 0 0 0 0 0 0 0 0 0 0 0 0 0 0 0 0

K03312 0 0 0 0 0 0 0 0 0 0 0 0 0 0 0 0 0 0 0 0 0 0 0 0 0 0 0 0 0 0 0 0 0 0 0 0 0 0 0 0 0 0 0 0 0 0 0 0

K03313 0 0 0 0 0 0 0 0 0 0 0 0 0 0 0 0 0 0 0 0 0 0 0 0 0 0 0 0 0 0 0 0 0 0 0 0 0 0 0 0 0 0 0 0 0 0 0 0

K03314 0 0 0 0 0 0 0 0 0 0 0 0 0 0 0 0 0 0 0 0 0 0 0 0 0 0 0 0 0 0 0 0 0 0 0 0 0 0 0 0 0 0 0 0 0 0 0 0

K03315 0 0 0 0 0 0 0 0 0 0 0 0 0 0 0 0 0 0 0 0 0 0 0 0 0 0 0 0 0 0 0 0 0 0 0 0 0 0 0 0 0 0 0 0 0 0 0 0

K03316 0 0 0 0 0 0 0 0 0 0 0 0 0 0 0 0 0 0 0 0 0 0 0 0 0 0 0 0 0 0 0 0 0 0 0 0 0 0 0 0 0 0 0 0 0 0 0 0

K03317 0 0 0 0 0 0 0 0 0 0 0 0 0 0 0 0 0 0 0 0 0 0 0 0 0 0 0 0 0 0 0 0 0 0 0 0 0 0 0 0 0 0 0 0 0 0 0 0

K03319 0 0 0 0 0 0 0 0 0 0 0 0 0 0 0 0 0 0 0 0 0 0 0 0 0 0 0 0 0 0 0 0 0 0 0 0 0 0 0 0 0 0 0 0 0 0 0 0

K03320 460 660 610 262 854 683 412 1056 741 818 1083 968 386 804 671 274 844 1288 748 762 988 1092 1196 1029 450 471 460 246 447 1041 24 922 554 818 1038 868 308 728 814 619 821 904 434 1020 1049 618 714 1007

K03321 0 0 0 0 0 0 0 0 0 0 0 0 0 0 0 0 0 0 0 0 0 0 0 0 0 0 0 0 0 0 0 0 0 0 0 0 0 0 0 0 0 0 0 0 0 0 0 0

K03322 0 0 0 0 0 0 0 0 0 0 0 0 0 0 0 0 0 0 0 0 0 0 0 0 0 0 0 0 0 0 0 0 0 0 0 0 0 0 0 0 0 0 0 0 0 0 0 0

K03324 0 0 0 0 23 16 0 9 0 0 0 0 0 0 0 0 0 14 0 0 0 0 0 6 0 0 0 0 0 13 0 0 0 0 0 0 0 0 0 0 0 0 0 0 0 0 0 0

K03325 14 0 0 0 30 16 8 9 0 0 3 0 0 0 16 0 0 14 0 18 0 0 0 36 0 0 0 0 0 18 0 30 0 24 0 0 0 0 0 0 0 8 0 0 0 0 0 0

K03326 0 0 0 0 0 0 0 0 0 0 0 0 0 0 0 0 0 0 0 0 0 0 0 0 0 0 0 0 0 0 0 0 0 0 0 0 0 0 0 0 0 0 0 0 0 0 0 0

K03327 0 0 0 0 0 0 0 0 0 0 0 0 0 0 0 0 0 0 0 0 0 0 0 0 0 0 0 0 0 0 0 0 0 0 0 0 0 0 0 0 0 0 0 0 0 0 0 0

K03328 14 0 0 0 13 0 17 0 0 0 0 0 0 0 32 0 0 0 0 18 0 0 0 27 0 0 0 0 0 0 0 4 0 24 0 0 0 0 0 0 0 17 0 0 0 0 0 0

K03329 0 0 0 0 0 0 0 0 0 0 0 0 0 0 0 0 0 0 0 0 0 0 0 0 0 0 0 0 0 0 0 0 0 0 0 0 0 0 0 0 0 0 0 0 0 0 0 0

K03330 140 304 301 96 572 477 136 656 216 244 481 240 119 325 347 119 410 526 223 347 439 342 496 482 120 132 126 90 166 704 0 391 212 228 322 280 96 320 486 328 500 486 182 590 590 308 318 602

K03331 0 0 0 0 0 0 0 0 0 0 0 0 0 0 0 0 0 0 0 0 0 0 0 0 0 0 0 0 0 0 0 0 0 0 0 0 0 0 0 0 0 0 0 0 0 0 0 0

K03332 0 0 0 0 0 0 0 0 0 0 0 0 0 0 0 0 0 0 0 0 0 0 0 0 0 0 0 0 0 0 0 0 0 0 0 0 0 0 0 0 0 0 0 0 0 0 0 0

K03333 0 0 0 0 0 0 0 0 0 0 0 0 0 0 0 0 0 0 0 0 0 0 0 0 0 0 0 0 0 0 0 0 0 0 0 0 0 0 0 0 0 0 0 0 0 0 0 0

K03335 0 0 0 0 0 0 0 0 0 0 0 0 0 0 0 0 0 0 0 0 0 0 0 0 0 0 0 0 0 0 0 0 0 0 0 0 0 0 0 0 0 0 0 0 0 0 0 0

K03336 0 0 0 0 0 0 0 0 0 0 0 0 0 0 0 0 0 0 0 0 0 0 0 0 0 0 0 0 0 0 0 0 0 0 0 0 0 0 0 0 0 0 0 0 0 0 0 0

K03337 0 0 0 0 0 0 0 0 0 0 0 0 0 0 0 0 0 0 0 0 0 0 0 0 0 0 0 0 0 0 0 0 0 0 0 0 0 0 0 0 0 0 0 0 0 0 0 0

K03338 0 0 0 0 0 0 0 0 0 0 0 0 0 0 0 0 0 0 0 0 0 0 0 0 0 0 0 0 0 0 0 0 0 0 0 0 0 0 0 0 0 0 0 0 0 0 0 0

K03339 0 0 0 0 0 0 0 0 0 0 0 0 0 0 0 0 0 0 0 0 0 0 0 0 0 0 0 0 0 0 0 0 0 0 0 0 0 0 0 0 0 0 0 0 0 0 0 0

K03340 0 0 0 0 0 0 0 0 0 0 0 0 0 0 0 0 0 0 0 0 0 0 0 0 0 0 0 0 0 0 0 0 0 0 0 0 0 0 0 0 0 0 0 0 0 0 0 0

K03341 0 0 0 0 0 0 0 0 0 0 0 0 0 0 0 0 0 0 0 0 0 0 0 0 0 0 0 0 0 0 0 0 0 0 0 0 0 0 0 0 0 0 0 0 0 0 0 0

K03342 0 0 0 0 0 0 0 0 0 0 0 0 0 0 0 0 0 0 0 0 0 0 0 0 0 0 0 0 0 0 0 0 0 0 0 0 0 0 0 0 0 0 0 0 0 0 0 0

K03343 0 0 0 0 0 0 0 0 0 0 0 0 0 0 0 0 0 0 0 0 0 0 0 0 0 0 0 0 0 0 0 0 0 0 0 0 0 0 0 0 0 0 0 0 0 0 0 0

K03346 0 0 0 0 0 0 0 0 0 0 0 0 0 0 0 0 0 0 0 0 0 0 0 0 0 0 0 0 0 0 0 0 0 0 0 0 0 0 0 0 0 0 0 0 0 0 0 0

K03366 0 0 0 0 0 0 0 0 0 0 0 0 0 0 0 0 0 0 0 0 0 0 0 0 0 0 0 0 0 0 0 0 0 0 0 0 0 0 0 0 0 0 0 0 0 0 0 0

K03367 0 0 0 0 0 0 0 0 0 0 0 0 0 0 0 0 0 0 0 0 0 0 0 0 0 0 0 0 0 0 0 0 0 0 0 0 0 0 0 0 0 0 0 0 0 0 0 0

K03379 0 0 0 0 0 0 0 0 0 0 0 0 0 0 0 0 0 0 0 0 0 0 0 0 0 0 0 0 0 0 0 0 0 0 0 0 0 0 0 0 0 0 0 0 0 0 0 0

K03380 0 0 0 0 0 0 0 0 0 0 0 0 0 0 0 0 0 0 0 0 0 0 0 0 0 0 0 0 0 0 0 0 0 0 0 0 0 0 0 0 0 0 0 0 0 0 0 0

K03381 0 0 0 0 0 0 0 0 0 0 0 0 0 0 0 0 0 0 0 0 0 0 0 0 0 0 0 0 0 0 0 0 0 0 0 0 0 0 0 0 0 0 0 0 0 0 0 0

K03382 0 0 0 0 0 0 0 0 0 0 0 0 0 0 0 0 0 0 0 0 0 0 0 0 0 0 0 0 0 0 0 0 0 0 0 0 0 0 0 0 0 0 0 0 0 0 0 0

K03383 0 0 0 0 0 0 0 0 0 0 0 0 0 0 0 0 0 0 0 0 0 0 0 0 0 0 0 0 0 0 0 0 0 0 0 0 0 0 0 0 0 0 0 0 0 0 0 0

K03384 0 0 0 0 0 0 0 0 0 0 0 0 0 0 0 0 0 0 0 0 0 0 0 0 0 0 0 0 0 0 0 0 0 0 0 0 0 0 0 0 0 0 0 0 0 0 0 0

K03385 0 0 0 0 0 0 0 0 0 0 0 0 0 0 0 0 0 0 0 0 0 0 0 0 0 0 0 0 0 0 0 0 0 0 0 0 0 0 0 0 0 0 0 0 0 0 0 0

K03386 426 684 538 216 724 598 340 880 684 694 950 882 358 854 612 294 818 1174 690 778 982 976 1212 892 462 441 451 300 420 932 24 988 598 802 1011 858 270 704 826 546 784 839 482 1067 1065 514 729 904

K03387 0 0 0 0 24 16 0 9 0 0 3 0 0 0 0 0 0 14 0 0 0 0 0 23 0 0 0 0 0 18 0 30 0 0 0 0 0 0 0 0 0 0 0 0 0 0 0 0

K03388 498 746 679 278 1024 843 416 1259 811 844 1180 1008 396 961 748 327 931 1424 760 852 1128 1144 1374 1106 484 476 480 318 475 1271 24 1084 671 887 1136 984 316 789 940 684 967 1024 538 1194 1186 662 804 1192

K03389 160 326 335 111 619 522 149 696 260 270 519 280 128 360 376 126 422 568 234 376 479 360 536 524 130 137 134 99 182 769 0 436 235 249 364 330 104 350 510 344 515 526 210 615 614 312 334 659

K03390 160 326 335 111 619 522 149 696 260 270 519 280 128 360 376 126 422 568 234 376 479 360 536 524 130 137 134 99 182 769 0 436 235 249 364 330 104 350 510 344 515 526 210 615 614 312 334 659

K03391 0 0 0 0 0 0 0 0 0 0 0 0 0 0 0 0 0 0 0 0 0 0 0 0 0 0 0 0 0 0 0 0 0 0 0 0 0 0 0 0 0 0 0 0 0 0 0 0

K03392 0 0 0 0 0 0 0 0 0 0 0 0 0 0 0 0 0 0 0 0 0 0 0 0 0 0 0 0 0 0 0 0 0 0 0 0 0 0 0 0 0 0 0 0 0 0 0 0

K03393 0 0 0 0 0 0 0 0 0 0 0 0 0 0 0 0 0 0 0 0 0 0 0 0 0 0 0 0 0 0 0 0 0 0 0 0 0 0 0 0 0 0 0 0 0 0 0 0

K03394 504 564 593 260 1102 866 412 1422 746 823 1111 900 324 866 683 302 786 1395 644 680 958 1121 1225 1064 400 398 399 298 380 1254 24 954 631 887 995 874 286 578 710 633 918 894 458 936 876 676 663 1122

K03395 0 0 0 0 0 0 0 0 0 0 0 0 0 0 0 0 0 0 0 0 0 0 0 0 0 0 0 0 0 0 0 0 0 0 0 0 0 0 0 0 0 0 0 0 0 0 0 0

K03396 0 0 0 0 0 0 0 0 0 0 0 0 0 0 0 0 0 0 0 0 0 0 0 0 0 0 0 0 0 0 0 0 0 0 0 0 0 0 0 0 0 0 0 0 0 0 0 0

K03397 0 0 0 0 0 0 0 0 0 0 0 0 0 0 0 0 0 0 0 0 0 0 0 0 0 0 0 0 0 0 0 0 0 0 0 0 0 0 0 0 0 0 0 0 0 0 0 0

K03399 34 22 34 15 78 62 21 49 44 26 41 40 10 35 45 6 12 55 12 48 40 18 40 78 10 4 8 9 17 82 0 76 22 46 42 50 8 30 24 16 16 48 28 24 24 4 16 58

K03400 0 0 0 0 0 0 0 0 0 0 0 0 0 0 0 0 0 0 0 0 0 0 0 0 0 0 0 0 0 0 0 0 0 0 0 0 0 0 0 0 0 0 0 0 0 0 0 0

K03402 0 0 0 0 0 0 0 0 0 0 0 0 0 0 0 0 0 0 0 0 0 0 0 0 0 0 0 0 0 0 0 0 0 0 0 0 0 0 0 0 0 0 0 0 0 0 0 0

K03403 0 0 0 0 0 0 0 0 0 0 0 0 0 0 0 0 0 0 0 0 0 0 0 0 0 0 0 0 0 0 0 0 0 0 0 0 0 0 0 0 0 0 0 0 0 0 0 0

K03404 14 0 0 0 37 16 17 9 0 0 3 0 0 0 32 0 0 14 0 18 0 0 0 50 0 0 0 0 0 18 0 30 0 24 0 0 0 0 0 0 0 17 0 0 0 0 0 0

K03405 0 0 0 0 6 0 8 0 0 0 0 0 0 0 16 0 0 0 0 0 0 0 0 14 0 0 0 0 0 0 0 0 0 0 0 0 0 0 0 0 0 8 0 0 0 0 0 0

K03406 1046 1323 1067 500 1466 1156 801 1899 1681 1722 2057 2186 802 1924 1180 651 1601 2733 1576 1506 2044 2389 2655 1908 1084 1016 1050 718 888 1758 72 2205 1402 1987 2374 2030 636 1346 1389 1069 1486 1584 1056 1889 1826 1088 1486 1726

K03407 338 420 344 166 452 352 267 582 552 574 666 728 268 601 372 202 508 884 526 476 648 784 838 610 354 338 346 218 292 532 24 681 436 638 772 654 212 438 429 340 452 498 327 580 572 350 470 533

K03408 338 420 344 166 502 384 267 600 552 574 674 728 268 601 372 202 508 912 526 476 648 784 838 656 354 338 346 218 292 568 24 734 436 638 772 654 212 438 429 340 452 498 327 580 572 350 470 533

K03409 0 0 0 0 0 0 0 0 0 0 0 0 0 0 0 0 0 0 0 0 0 0 0 0 0 0 0 0 0 0 0 0 0 0 0 0 0 0 0 0 0 0 0 0 0 0 0 0

K03410 0 0 0 0 24 16 0 9 0 0 5 0 0 0 0 0 0 14 0 0 0 0 0 23 0 0 0 0 0 18 0 34 0 0 0 0 0 0 0 0 0 0 0 0 0 0 0 0

K03411 0 0 0 0 24 16 0 9 0 0 3 0 0 0 0 0 0 14 0 0 0 0 0 23 0 0 0 0 0 18 0 30 0 0 0 0 0 0 0 0 0 0 0 0 0 0 0 0

K03412 338 420 344 166 452 352 267 582 552 574 666 728 268 601 372 202 508 884 526 476 648 784 838 610 354 338 346 218 292 532 24 678 436 638 772 654 212 438 429 340 452 498 327 580 572 350 470 533

K03413 454 884 697 264 878 745 395 1025 789 813 1180 1036 450 986 755 339 1052 1384 854 965 1216 1133 1445 1086 546 544 545 318 536 1175 24 1152 666 845 1193 994 329 940 1121 703 984 1074 561 1404 1446 640 913 1148

K03414 0 0 0 0 0 0 0 0 0 0 0 0 0 0 0 0 0 0 0 0 0 0 0 0 0 0 0 0 0 0 0 0 0 0 0 0 0 0 0 0 0 0 0 0 0 0 0 0

K03415 0 0 0 0 0 0 0 0 0 0 0 0 0 0 0 0 0 0 0 0 0 0 0 0 0 0 0 0 0 0 0 0 0 0 0 0 0 0 0 0 0 0 0 0 0 0 0 0

K03416 0 0 0 0 0 0 0 0 0 0 0 0 0 0 0 0 0 0 0 0 0 0 0 0 0 0 0 0 0 0 0 0 0 0 0 0 0 0 0 0 0 0 0 0 0 0 0 0

K03417 0 0 0 0 0 0 0 0 0 0 0 0 0 0 0 0 0 0 0 0 0 0 0 0 0 0 0 0 0 0 0 0 0 0 0 0 0 0 0 0 0 0 0 0 0 0 0 0

K03418 0 0 0 0 0 0 0 0 0 0 0 0 0 0 0 0 0 0 0 0 0 0 0 0 0 0 0 0 0 0 0 0 0 0 0 0 0 0 0 0 0 0 0 0 0 0 0 0

K03420 140 304 301 96 596 493 136 664 216 244 483 240 119 325 347 119 410 540 223 347 439 342 496 505 120 132 126 90 166 722 0 421 212 228 322 280 96 320 486 328 500 486 182 590 590 308 318 602

K03421 66 40 107 47 282 216 72 348 84 124 195 86 28 72 124 27 101 222 59 62 106 150 124 208 12 30 21 9 38 292 0 80 50 88 83 77 37 54 90 122 168 154 28 103 96 144 59 230

K03422 132 126 176 62 490 392 94 560 154 150 294 126 38 228 232 80 188 372 70 190 244 204 302 335 46 34 40 80 66 539 0 276 167 206 182 192 46 115 216 187 314 290 131 278 232 187 150 416

K03423 0 0 0 0 0 0 0 0 0 0 0 0 0 0 0 0 0 0 0 0 0 0 0 0 0 0 0 0 0 0 0 0 0 0 0 0 0 0 0 0 0 0 0 0 0 0 0 0

K03424 522 582 666 292 1252 988 455 1702 786 922 1260 944 342 904 746 322 875 1534 691 676 1024 1254 1309 1134 401 424 412 298 400 1428 24 900 659 905 1036 902 314 602 776 739 1071 990 458 1014 947 816 706 1294

K03425 0 0 0 0 0 0 0 0 0 0 0 0 0 0 0 0 0 0 0 0 0 0 0 0 0 0 0 0 0 0 0 0 0 0 0 0 0 0 0 0 0 0 0 0 0 0 0 0

K03426 52 40 107 47 253 200 64 338 84 124 195 86 28 72 108 27 101 208 59 44 106 150 124 189 12 30 21 9 38 278 0 84 50 64 83 77 37 54 90 122 168 145 28 103 96 144 59 230

K03427 460 660 610 262 846 683 404 1056 741 818 1081 968 386 804 655 274 844 1288 748 762 988 1092 1196 998 450 471 460 246 447 1036 24 892 554 818 1038 868 308 728 814 619 821 896 434 1020 1049 618 714 1007

K03428 0 0 0 0 0 0 0 0 0 0 0 0 0 0 0 0 0 0 0 0 0 0 0 0 0 0 0 0 0 0 0 0 0 0 0 0 0 0 0 0 0 0 0 0 0 0 0 0

K03429 0 0 0 0 0 0 0 0 0 0 0 0 0 0 0 0 0 0 0 0 0 0 0 0 0 0 0 0 0 0 0 0 0 0 0 0 0 0 0 0 0 0 0 0 0 0 0 0

K03430 0 0 0 0 0 0 0 0 0 0 0 0 0 0 0 0 0 0 0 0 0 0 0 0 0 0 0 0 0 0 0 0 0 0 0 0 0 0 0 0 0 0 0 0 0 0 0 0

K03431 478 724 645 262 978 798 404 1219 768 818 1142 968 386 926 719 320 919 1382 748 822 1088 1127 1335 1064 473 471 472 308 458 1206 24 1038 648 866 1094 934 308 759 916 668 952 984 510 1170 1161 658 788 1134

K03432 140 304 301 96 572 477 136 656 216 244 481 240 119 325 347 119 410 526 223 347 439 342 496 482 120 132 126 90 166 704 0 391 212 228 322 280 96 320 486 328 500 486 182 590 590 308 318 602

K03433 478 724 645 262 978 798 404 1219 768 818 1142 968 386 926 719 320 919 1382 748 822 1088 1127 1335 1064 473 471 472 308 458 1206 24 1038 648 866 1094 934 308 759 916 668 952 984 510 1170 1161 658 788 1134

K03434 0 0 0 0 0 0 0 0 0 0 0 0 0 0 0 0 0 0 0 0 0 0 0 0 0 0 0 0 0 0 0 0 0 0 0 0 0 0 0 0 0 0 0 0 0 0 0 0

K03435 0 0 0 0 0 0 0 0 0 0 0 0 0 0 0 0 0 0 0 0 0 0 0 0 0 0 0 0 0 0 0 0 0 0 0 0 0 0 0 0 0 0 0 0 0 0 0 0

K03436 0 0 0 0 0 0 0 0 0 0 0 0 0 0 0 0 0 0 0 0 0 0 0 0 0 0 0 0 0 0 0 0 0 0 0 0 0 0 0 0 0 0 0 0 0 0 0 0

K03437 0 0 0 0 0 0 0 0 0 0 0 0 0 0 0 0 0 0 0 0 0 0 0 0 0 0 0 0 0 0 0 0 0 0 0 0 0 0 0 0 0 0 0 0 0 0 0 0

K03438 338 420 344 166 406 320 267 564 552 574 661 728 268 601 372 202 508 856 526 476 648 784 838 582 354 338 346 218 292 502 24 648 436 638 772 654 212 438 429 340 452 498 327 580 572 350 470 533

K03439 0 0 0 0 0 0 0 0 0 0 0 0 0 0 0 0 0 0 0 0 0 0 0 0 0 0 0 0 0 0 0 0 0 0 0 0 0 0 0 0 0 0 0 0 0 0 0 0

K03442 338 420 344 166 406 320 267 564 552 574 661 728 268 601 372 202 508 856 526 476 648 784 838 582 354 338 346 218 292 502 24 648 436 638 772 654 212 438 429 340 452 498 327 580 572 350 470 533

K03444 0 0 0 0 0 0 0 0 0 0 0 0 0 0 0 0 0 0 0 0 0 0 0 0 0 0 0 0 0 0 0 0 0 0 0 0 0 0 0 0 0 0 0 0 0 0 0 0

K03445 0 0 0 0 0 0 0 0 0 0 0 0 0 0 0 0 0 0 0 0 0 0 0 0 0 0 0 0 0 0 0 0 0 0 0 0 0 0 0 0 0 0 0 0 0 0 0 0

K03446 0 0 0 0 0 0 0 0 0 0 0 0 0 0 0 0 0 0 0 0 0 0 0 0 0 0 0 0 0 0 0 0 0 0 0 0 0 0 0 0 0 0 0 0 0 0 0 0

K03449 0 0 0 0 0 0 0 0 0 0 0 0 0 0 0 0 0 0 0 0 0 0 0 0 0 0 0 0 0 0 0 0 0 0 0 0 0 0 0 0 0 0 0 0 0 0 0 0

K03451 0 0 0 0 0 0 0 0 0 0 0 0 0 0 0 0 0 0 0 0 0 0 0 0 0 0 0 0 0 0 0 0 0 0 0 0 0 0 0 0 0 0 0 0 0 0 0 0

K03452 0 0 0 0 0 0 0 0 0 0 0 0 0 0 0 0 0 0 0 0 0 0 0 0 0 0 0 0 0 0 0 0 0 0 0 0 0 0 0 0 0 0 0 0 0 0 0 0

K03453 47 18 73 32 206 154 52 298 40 98 154 45 18 37 78 20 89 167 48 33 66 133 84 130 1 26 13 0 21 209 0 5 28 67 40 28 28 24 66 106 152 105 0 78 72 140 43 173

K03455 338 420 344 166 415 320 276 564 552 574 664 728 268 601 388 202 508 856 526 476 648 784 838 612 354 338 346 218 292 506 24 678 436 638 772 654 212 438 429 340 452 507 327 580 572 350 470 533

K03457 0 0 0 0 0 0 0 0 0 0 0 0 0 0 0 0 0 0 0 0 0 0 0 0 0 0 0 0 0 0 0 0 0 0 0 0 0 0 0 0 0 0 0 0 0 0 0 0

K03458 0 0 0 0 0 0 0 0 0 0 0 0 0 0 0 0 0 0 0 0 0 0 0 0 0 0 0 0 0 0 0 0 0 0 0 0 0 0 0 0 0 0 0 0 0 0 0 0

K03459 0 0 0 0 0 0 0 0 0 0 0 0 0 0 0 0 0 0 0 0 0 0 0 0 0 0 0 0 0 0 0 0 0 0 0 0 0 0 0 0 0 0 0 0 0 0 0 0

K03462 52 40 107 47 252 200 64 338 84 124 192 86 28 72 108 27 101 208 59 44 106 150 124 172 12 30 21 9 38 274 0 50 50 64 83 77 37 54 90 122 168 145 28 103 96 144 59 230

K03464 0 0 0 0 0 0 0 0 0 0 0 0 0 0 0 0 0 0 0 0 0 0 0 0 0 0 0 0 0 0 0 0 0 0 0 0 0 0 0 0 0 0 0 0 0 0 0 0

K03465 380 620 503 216 564 468 331 708 657 694 886 882 358 732 532 247 742 1066 690 680 883 941 1072 790 438 441 440 238 409 745 24 812 504 705 954 792 270 674 724 497 653 742 406 916 953 474 654 776

K03466 0 0 0 0 0 0 0 0 0 0 0 0 0 0 0 0 0 0 0 0 0 0 0 0 0 0 0 0 0 0 0 0 0 0 0 0 0 0 0 0 0 0 0 0 0 0 0 0

K03469 338 420 344 166 430 336 267 572 552 574 664 728 268 601 372 202 508 870 526 476 648 784 838 604 354 338 346 218 292 520 24 678 436 638 772 654 212 438 429 340 452 498 327 580 572 350 470 533

K03470 478 724 645 262 978 798 404 1219 768 818 1142 968 386 926 719 320 919 1382 748 822 1088 1127 1335 1064 473 471 472 308 458 1206 24 1038 648 866 1094 934 308 759 916 668 952 984 510 1170 1161 658 788 1134

K03471 20 22 34 15 47 46 12 40 44 26 38 40 10 35 29 6 12 41 12 30 40 18 40 42 10 4 8 9 17 65 0 46 22 21 42 50 8 30 24 16 16 40 28 24 24 4 16 58

K03472 0 0 0 0 0 0 0 0 0 0 0 0 0 0 0 0 0 0 0 0 0 0 0 0 0 0 0 0 0 0 0 0 0 0 0 0 0 0 0 0 0 0 0 0 0 0 0 0

K03473 0 0 0 0 0 0 0 0 0 0 0 0 0 0 0 0 0 0 0 0 0 0 0 0 0 0 0 0 0 0 0 0 0 0 0 0 0 0 0 0 0 0 0 0 0 0 0 0

K03474 0 0 0 0 0 0 0 0 0 0 0 0 0 0 0 0 0 0 0 0 0 0 0 0 0 0 0 0 0 0 0 0 0 0 0 0 0 0 0 0 0 0 0 0 0 0 0 0

K03475 0 0 0 0 0 0 0 0 0 0 0 0 0 0 0 0 0 0 0 0 0 0 0 0 0 0 0 0 0 0 0 0 0 0 0 0 0 0 0 0 0 0 0 0 0 0 0 0

K03476 0 0 0 0 0 0 0 0 0 0 0 0 0 0 0 0 0 0 0 0 0 0 0 0 0 0 0 0 0 0 0 0 0 0 0 0 0 0 0 0 0 0 0 0 0 0 0 0

K03477 0 0 0 0 0 0 0 0 0 0 0 0 0 0 0 0 0 0 0 0 0 0 0 0 0 0 0 0 0 0 0 0 0 0 0 0 0 0 0 0 0 0 0 0 0 0 0 0

K03478 0 0 0 0 0 0 0 0 0 0 0 0 0 0 0 0 0 0 0 0 0 0 0 0 0 0 0 0 0 0 0 0 0 0 0 0 0 0 0 0 0 0 0 0 0 0 0 0

K03480 0 0 0 0 0 0 0 0 0 0 0 0 0 0 0 0 0 0 0 0 0 0 0 0 0 0 0 0 0 0 0 0 0 0 0 0 0 0 0 0 0 0 0 0 0 0 0 0

K03481 0 0 0 0 0 0 0 0 0 0 0 0 0 0 0 0 0 0 0 0 0 0 0 0 0 0 0 0 0 0 0 0 0 0 0 0 0 0 0 0 0 0 0 0 0 0 0 0

K03482 0 0 0 0 0 0 0 0 0 0 0 0 0 0 0 0 0 0 0 0 0 0 0 0 0 0 0 0 0 0 0 0 0 0 0 0 0 0 0 0 0 0 0 0 0 0 0 0

K03483 0 0 0 0 0 0 0 0 0 0 0 0 0 0 0 0 0 0 0 0 0 0 0 0 0 0 0 0 0 0 0 0 0 0 0 0 0 0 0 0 0 0 0 0 0 0 0 0

K03484 0 0 0 0 0 0 0 0 0 0 0 0 0 0 0 0 0 0 0 0 0 0 0 0 0 0 0 0 0 0 0 0 0 0 0 0 0 0 0 0 0 0 0 0 0 0 0 0

K03485 0 0 0 0 0 0 0 0 0 0 0 0 0 0 0 0 0 0 0 0 0 0 0 0 0 0 0 0 0 0 0 0 0 0 0 0 0 0 0 0 0 0 0 0 0 0 0 0

K03486 0 0 0 0 0 0 0 0 0 0 0 0 0 0 0 0 0 0 0 0 0 0 0 0 0 0 0 0 0 0 0 0 0 0 0 0 0 0 0 0 0 0 0 0 0 0 0 0

K03487 0 0 0 0 0 0 0 0 0 0 0 0 0 0 0 0 0 0 0 0 0 0 0 0 0 0 0 0 0 0 0 0 0 0 0 0 0 0 0 0 0 0 0 0 0 0 0 0

K03488 0 0 0 0 0 0 0 0 0 0 0 0 0 0 0 0 0 0 0 0 0 0 0 0 0 0 0 0 0 0 0 0 0 0 0 0 0 0 0 0 0 0 0 0 0 0 0 0

K03489 0 0 0 0 0 0 0 0 0 0 0 0 0 0 0 0 0 0 0 0 0 0 0 0 0 0 0 0 0 0 0 0 0 0 0 0 0 0 0 0 0 0 0 0 0 0 0 0

K03490 0 0 0 0 0 0 0 0 0 0 0 0 0 0 0 0 0 0 0 0 0 0 0 0 0 0 0 0 0 0 0 0 0 0 0 0 0 0 0 0 0 0 0 0 0 0 0 0

K03491 0 0 0 0 0 0 0 0 0 0 0 0 0 0 0 0 0 0 0 0 0 0 0 0 0 0 0 0 0 0 0 0 0 0 0 0 0 0 0 0 0 0 0 0 0 0 0 0

K03492 0 0 0 0 0 0 0 0 0 0 0 0 0 0 0 0 0 0 0 0 0 0 0 0 0 0 0 0 0 0 0 0 0 0 0 0 0 0 0 0 0 0 0 0 0 0 0 0

K03493 0 0 0 0 0 0 0 0 0 0 0 0 0 0 0 0 0 0 0 0 0 0 0 0 0 0 0 0 0 0 0 0 0 0 0 0 0 0 0 0 0 0 0 0 0 0 0 0

K03495 338 420 344 166 406 320 267 564 552 574 661 728 268 601 372 202 508 856 526 476 648 784 838 582 354 338 346 218 292 502 24 648 436 638 772 654 212 438 429 340 452 498 327 580 572 350 470 533

K03496 437 524 486 214 819 650 340 1074 662 698 917 814 296 794 560 275 685 1173 584 618 853 970 1102 855 388 368 378 290 342 963 24 874 580 799 912 798 249 524 620 511 750 740 430 833 780 533 604 891

K03497 338 420 344 166 406 320 267 564 552 574 661 728 268 601 372 202 508 856 526 476 648 784 838 582 354 338 346 218 292 502 24 648 436 638 772 654 212 438 429 340 452 498 327 580 572 350 470 533

K03498 531 764 752 310 1254 1013 468 1566 852 942 1334 1054 414 998 826 348 1020 1604 808 866 1193 1278 1458 1242 484 501 493 318 496 1493 24 1089 699 930 1177 1012 344 814 1005 790 1120 1129 538 1273 1257 802 847 1365

K03499 518 768 713 292 1072 888 428 1299 854 870 1219 1049 406 996 777 334 943 1464 772 882 1168 1162 1414 1148 494 480 487 326 492 1336 24 1130 694 908 1179 1034 324 819 964 700 982 1064 566 1219 1210 665 820 1250

K03500 412 684 538 216 695 582 331 872 684 694 947 882 358 854 596 294 818 1160 690 760 982 976 1212 855 462 441 451 300 420 914 24 958 598 778 1011 858 270 704 826 546 784 830 482 1067 1065 514 729 904

K03501 338 420 344 166 406 320 267 564 552 574 661 728 268 601 372 202 508 856 526 476 648 784 838 582 354 338 346 218 292 502 24 648 436 638 772 654 212 438 429 340 452 498 327 580 572 350 470 533

K03502 0 0 0 0 0 0 0 0 0 0 0 0 0 0 0 0 0 0 0 0 0 0 0 0 0 0 0 0 0 0 0 0 0 0 0 0 0 0 0 0 0 0 0 0 0 0 0 0

K03503 0 0 0 0 0 0 0 0 0 0 0 0 0 0 0 0 0 0 0 0 0 0 0 0 0 0 0 0 0 0 0 0 0 0 0 0 0 0 0 0 0 0 0 0 0 0 0 0

K03516 0 0 0 0 0 0 0 0 0 0 0 0 0 0 0 0 0 0 0 0 0 0 0 0 0 0 0 0 0 0 0 0 0 0 0 0 0 0 0 0 0 0 0 0 0 0 0 0

K03517 478 724 645 262 978 798 404 1219 768 818 1142 968 386 926 719 320 919 1382 748 822 1088 1127 1335 1064 473 471 472 308 458 1206 24 1038 648 866 1094 934 308 759 916 668 952 984 510 1170 1161 658 788 1134

K03518 0 0 0 0 0 0 0 0 0 0 0 0 0 0 0 0 0 0 0 0 0 0 0 0 0 0 0 0 0 0 0 0 0 0 0 0 0 0 0 0 0 0 0 0 0 0 0 0

K03519 0 0 0 0 0 0 0 0 0 0 0 0 0 0 0 0 0 0 0 0 0 0 0 0 0 0 0 0 0 0 0 0 0 0 0 0 0 0 0 0 0 0 0 0 0 0 0 0

K03520 0 0 0 0 0 0 0 0 0 0 0 0 0 0 0 0 0 0 0 0 0 0 0 0 0 0 0 0 0 0 0 0 0 0 0 0 0 0 0 0 0 0 0 0 0 0 0 0

K03521 338 420 344 166 406 320 267 564 552 574 661 728 268 601 372 202 508 856 526 476 648 784 838 582 354 338 346 218 292 502 24 648 436 638 772 654 212 438 429 340 452 498 327 580 572 350 470 533

K03522 412 684 538 216 695 582 331 872 684 694 947 882 358 854 596 294 818 1160 690 760 982 976 1212 855 462 441 451 300 420 914 24 958 598 778 1011 858 270 704 826 546 784 830 482 1067 1065 514 729 904

K03523 34 22 34 15 78 62 21 49 44 26 41 40 10 35 45 6 12 55 12 48 40 18 40 78 10 4 8 9 17 82 0 76 22 46 42 50 8 30 24 16 16 48 28 24 24 4 16 58

K03524 478 724 645 262 978 798 404 1219 768 818 1142 968 386 926 719 320 919 1382 748 822 1088 1127 1335 1064 473 471 472 308 458 1206 24 1038 648 866 1094 934 308 759 916 668 952 984 510 1170 1161 658 788 1134

K03525 338 420 344 166 406 320 267 564 552 574 661 728 268 601 372 202 508 856 526 476 648 784 838 582 354 338 346 218 292 502 24 648 436 638 772 654 212 438 429 340 452 498 327 580 572 350 470 533

K03526 338 420 344 166 406 320 267 564 552 574 661 728 268 601 372 202 508 856 526 476 648 784 838 582 354 338 346 218 292 502 24 648 436 638 772 654 212 438 429 340 452 498 327 580 572 350 470 533

K03527 0 0 0 0 0 0 0 0 0 0 0 0 0 0 0 0 0 0 0 0 0 0 0 0 0 0 0 0 0 0 0 0 0 0 0 0 0 0 0 0 0 0 0 0 0 0 0 0

K03528 0 0 0 0 0 0 0 0 0 0 0 0 0 0 0 0 0 0 0 0 0 0 0 0 0 0 0 0 0 0 0 0 0 0 0 0 0 0 0 0 0 0 0 0 0 0 0 0

K03529 412 684 538 216 719 598 331 880 684 694 950 882 358 854 596 294 818 1174 690 760 982 976 1212 878 462 441 451 300 420 932 24 988 598 778 1011 858 270 704 826 546 784 830 482 1067 1065 514 729 904

K03530 380 620 503 216 564 468 331 708 657 694 886 882 358 732 532 247 742 1066 690 680 883 941 1072 790 438 441 440 238 409 745 24 812 504 705 954 792 270 674 724 497 653 742 406 916 953 474 654 776

K03531 636 1388 1157 410 1608 1369 596 1825 1110 1176 1880 1430 660 1442 1262 504 1696 2120 1240 1517 1890 1632 2175 1777 750 778 764 428 818 2122 24 1708 946 1140 1696 1412 483 1496 1902 1188 1685 1804 823 2332 2418 1073 1414 1992

K03532 0 0 0 0 0 0 0 0 0 0 0 0 0 0 0 0 0 0 0 0 0 0 0 0 0 0 0 0 0 0 0 0 0 0 0 0 0 0 0 0 0 0 0 0 0 0 0 0

K03533 0 0 0 0 0 0 0 0 0 0 0 0 0 0 0 0 0 0 0 0 0 0 0 0 0 0 0 0 0 0 0 0 0 0 0 0 0 0 0 0 0 0 0 0 0 0 0 0

K03534 0 0 0 0 0 0 0 0 0 0 0 0 0 0 0 0 0 0 0 0 0 0 0 0 0 0 0 0 0 0 0 0 0 0 0 0 0 0 0 0 0 0 0 0 0 0 0 0

K03535 0 0 0 0 0 0 0 0 0 0 0 0 0 0 0 0 0 0 0 0 0 0 0 0 0 0 0 0 0 0 0 0 0 0 0 0 0 0 0 0 0 0 0 0 0 0 0 0

K03536 0 0 0 0 0 0 0 0 0 0 0 0 0 0 0 0 0 0 0 0 0 0 0 0 0 0 0 0 0 0 0 0 0 0 0 0 0 0 0 0 0 0 0 0 0 0 0 0

K03537 99 104 142 47 414 330 72 511 110 124 256 86 28 194 188 74 176 317 59 142 204 186 263 274 35 30 32 71 49 461 0 226 144 161 140 143 37 85 192 171 298 242 103 254 208 184 134 358

K03538 140 304 301 96 572 477 136 656 216 244 481 240 119 325 347 119 410 526 223 347 439 342 496 482 120 132 126 90 166 704 0 391 212 228 322 280 96 320 486 328 500 486 182 590 590 308 318 602

K03539 99 104 142 47 414 330 72 511 110 124 256 86 28 194 188 74 176 317 59 142 204 186 263 274 35 30 32 71 49 461 0 226 144 161 140 143 37 85 192 171 298 242 103 254 208 184 134 358

K03540 140 304 301 96 572 477 136 656 216 244 481 240 119 325 347 119 410 526 223 347 439 342 496 482 120 132 126 90 166 704 0 391 212 228 322 280 96 320 486 328 500 486 182 590 590 308 318 602

K03543 0 0 0 0 0 0 0 0 0 0 0 0 0 0 0 0 0 0 0 0 0 0 0 0 0 0 0 0 0 0 0 0 0 0 0 0 0 0 0 0 0 0 0 0 0 0 0 0

K03544 338 420 344 166 406 320 267 564 552 574 661 728 268 601 372 202 508 856 526 476 648 784 838 582 354 338 346 218 292 502 24 648 436 638 772 654 212 438 429 340 452 498 327 580 572 350 470 533

K03545 338 420 344 166 406 320 267 564 552 574 661 728 268 601 372 202 508 856 526 476 648 784 838 582 354 338 346 218 292 502 24 648 436 638 772 654 212 438 429 340 452 498 327 580 572 350 470 533

K03546 478 724 645 262 954 782 404 1210 768 818 1142 968 386 926 719 320 919 1368 748 822 1088 1127 1335 1040 473 471 472 308 458 1188 24 1008 648 866 1094 934 308 759 916 668 952 984 510 1170 1161 658 788 1134

K03547 0 0 0 0 0 0 0 0 0 0 0 0 0 0 0 0 0 0 0 0 0 0 0 0 0 0 0 0 0 0 0 0 0 0 0 0 0 0 0 0 0 0 0 0 0 0 0 0

K03548 0 0 0 0 0 0 0 0 0 0 0 0 0 0 0 0 0 0 0 0 0 0 0 0 0 0 0 0 0 0 0 0 0 0 0 0 0 0 0 0 0 0 0 0 0 0 0 0

K03549 0 0 0 0 1 0 0 0 0 0 3 0 0 0 0 0 0 0 0 0 0 0 0 0 0 0 0 0 0 0 0 0 0 0 0 0 0 0 0 0 0 0 0 0 0 0 0 0

K03550 338 420 344 166 406 320 267 564 552 574 664 728 268 601 372 202 508 856 526 476 648 784 838 582 354 338 346 218 292 502 24 651 436 638 772 654 212 438 429 340 452 498 327 580 572 350 470 533

K03551 338 420 344 166 406 320 267 564 552 574 664 728 268 601 372 202 508 856 526 476 648 784 838 582 354 338 346 218 292 502 24 651 436 638 772 654 212 438 429 340 452 498 327 580 572 350 470 533

K03552 99 104 142 47 390 314 72 502 110 124 253 86 28 194 188 74 176 303 59 142 204 186 263 250 35 30 32 71 49 444 0 196 144 161 140 143 37 85 192 171 298 242 103 254 208 184 134 358

K03553 338 420 344 166 406 320 267 564 552 574 661 728 268 601 372 202 508 856 526 476 648 784 838 582 354 338 346 218 292 502 24 648 436 638 772 654 212 438 429 340 452 498 327 580 572 350 470 533

K03554 0 0 0 0 0 0 0 0 0 0 0 0 0 0 0 0 0 0 0 0 0 0 0 0 0 0 0 0 0 0 0 0 0 0 0 0 0 0 0 0 0 0 0 0 0 0 0 0

K03555 380 620 503 216 588 484 331 717 657 694 889 882 358 732 532 247 742 1080 690 680 883 941 1072 813 438 441 440 238 409 762 24 842 504 705 954 792 270 674 724 497 653 742 406 916 953 474 654 776

K03556 0 0 0 0 0 0 0 0 0 0 0 0 0 0 0 0 0 0 0 0 0 0 0 0 0 0 0 0 0 0 0 0 0 0 0 0 0 0 0 0 0 0 0 0 0 0 0 0

K03557 0 0 0 0 0 0 0 0 0 0 0 0 0 0 0 0 0 0 0 0 0 0 0 0 0 0 0 0 0 0 0 0 0 0 0 0 0 0 0 0 0 0 0 0 0 0 0 0

K03558 0 0 0 0 0 0 0 0 0 0 0 0 0 0 0 0 0 0 0 0 0 0 0 0 0 0 0 0 0 0 0 0 0 0 0 0 0 0 0 0 0 0 0 0 0 0 0 0

K03559 404 460 451 214 664 520 340 902 636 698 853 814 296 673 496 228 610 1064 584 538 754 935 962 767 365 368 367 228 330 776 24 698 486 726 856 732 249 493 518 462 620 652 355 682 668 493 530 764

K03560 0 0 0 0 0 0 0 0 0 0 0 0 0 0 0 0 0 0 0 0 0 0 0 0 0 0 0 0 0 0 0 0 0 0 0 0 0 0 0 0 0 0 0 0 0 0 0 0

K03561 404 460 451 214 664 520 340 902 636 698 853 814 296 673 496 228 610 1064 584 538 754 935 962 767 365 368 367 228 330 776 24 698 486 726 856 732 249 493 518 462 620 652 355 682 668 493 530 764

K03562 0 0 0 0 0 0 0 0 0 0 0 0 0 0 0 0 0 0 0 0 0 0 0 0 0 0 0 0 0 0 0 0 0 0 0 0 0 0 0 0 0 0 0 0 0 0 0 0

K03563 0 0 0 0 0 0 0 0 0 0 0 0 0 0 0 0 0 0 0 0 0 0 0 0 0 0 0 0 0 0 0 0 0 0 0 0 0 0 0 0 0 0 0 0 0 0 0 0

K03564 412 684 538 216 695 582 331 872 684 694 950 882 358 854 596 294 818 1160 690 760 982 976 1212 855 462 441 451 300 420 914 24 962 598 778 1011 858 270 704 826 546 784 830 482 1067 1065 514 729 904

K03565 0 0 0 0 0 0 0 0 0 0 0 0 0 0 0 0 0 0 0 0 0 0 0 0 0 0 0 0 0 0 0 0 0 0 0 0 0 0 0 0 0 0 0 0 0 0 0 0

K03566 0 0 0 0 0 0 0 0 0 0 0 0 0 0 0 0 0 0 0 0 0 0 0 0 0 0 0 0 0 0 0 0 0 0 0 0 0 0 0 0 0 0 0 0 0 0 0 0

K03567 0 0 0 0 0 0 0 0 0 0 0 0 0 0 0 0 0 0 0 0 0 0 0 0 0 0 0 0 0 0 0 0 0 0 0 0 0 0 0 0 0 0 0 0 0 0 0 0

K03568 520 924 804 312 1136 944 468 1364 873 938 1367 1122 478 1058 878 366 1153 1592 912 1028 1322 1284 1568 1272 558 574 566 328 574 1449 24 1203 716 933 1276 1072 366 994 1210 825 1152 1228 589 1507 1542 783 972 1378

K03569 404 460 451 214 664 520 340 902 636 698 853 814 296 673 496 228 610 1064 584 538 754 935 962 767 365 368 367 228 330 776 24 698 486 726 856 732 249 493 518 462 620 652 355 682 668 493 530 764

K03570 0 0 0 0 0 0 0 0 0 0 0 0 0 0 0 0 0 0 0 0 0 0 0 0 0 0 0 0 0 0 0 0 0 0 0 0 0 0 0 0 0 0 0 0 0 0 0 0

K03571 0 0 0 0 0 0 0 0 0 0 0 0 0 0 0 0 0 0 0 0 0 0 0 0 0 0 0 0 0 0 0 0 0 0 0 0 0 0 0 0 0 0 0 0 0 0 0 0

K03572 42 200 159 49 182 163 64 154 106 120 228 154 91 132 160 46 234 224 164 205 234 156 234 232 84 102 94 19 116 260 0 194 68 67 182 137 58 236 295 157 201 244 80 337 382 125 184 244

K03573 0 0 0 0 0 0 0 0 0 0 0 0 0 0 0 0 0 0 0 0 0 0 0 0 0 0 0 0 0 0 0 0 0 0 0 0 0 0 0 0 0 0 0 0 0 0 0 0

K03574 634 982 984 390 1582 1282 600 1992 998 1160 1710 1252 524 1166 1096 414 1343 1953 1019 1123 1493 1567 1776 1578 570 629 600 336 634 1914 24 1228 795 1088 1400 1176 432 1074 1366 1053 1473 1495 617 1688 1710 1066 1074 1782

K03575 338 420 344 166 436 336 276 572 552 574 664 728 268 601 388 202 508 870 526 476 648 784 838 618 354 338 346 218 292 520 24 678 436 638 772 654 212 438 429 340 452 507 327 580 572 350 470 533

K03576 0 0 0 0 0 0 0 0 0 0 0 0 0 0 0 0 0 0 0 0 0 0 0 0 0 0 0 0 0 0 0 0 0 0 0 0 0 0 0 0 0 0 0 0 0 0 0 0

K03577 0 0 0 0 0 0 0 0 0 0 0 0 0 0 0 0 0 0 0 0 0 0 0 0 0 0 0 0 0 0 0 0 0 0 0 0 0 0 0 0 0 0 0 0 0 0 0 0

K03578 0 0 0 0 0 0 0 0 0 0 0 0 0 0 0 0 0 0 0 0 0 0 0 0 0 0 0 0 0 0 0 0 0 0 0 0 0 0 0 0 0 0 0 0 0 0 0 0

K03579 0 0 0 0 0 0 0 0 0 0 0 0 0 0 0 0 0 0 0 0 0 0 0 0 0 0 0 0 0 0 0 0 0 0 0 0 0 0 0 0 0 0 0 0 0 0 0 0

K03580 0 0 0 0 0 0 0 0 0 0 0 0 0 0 0 0 0 0 0 0 0 0 0 0 0 0 0 0 0 0 0 0 0 0 0 0 0 0 0 0 0 0 0 0 0 0 0 0

K03581 0 0 0 0 0 0 0 0 0 0 0 0 0 0 0 0 0 0 0 0 0 0 0 0 0 0 0 0 0 0 0 0 0 0 0 0 0 0 0 0 0 0 0 0 0 0 0 0

K03582 0 0 0 0 0 0 0 0 0 0 0 0 0 0 0 0 0 0 0 0 0 0 0 0 0 0 0 0 0 0 0 0 0 0 0 0 0 0 0 0 0 0 0 0 0 0 0 0

K03583 0 0 0 0 0 0 0 0 0 0 0 0 0 0 0 0 0 0 0 0 0 0 0 0 0 0 0 0 0 0 0 0 0 0 0 0 0 0 0 0 0 0 0 0 0 0 0 0

K03584 0 0 0 0 0 0 0 0 0 0 0 0 0 0 0 0 0 0 0 0 0 0 0 0 0 0 0 0 0 0 0 0 0 0 0 0 0 0 0 0 0 0 0 0 0 0 0 0

K03585 0 0 0 0 0 0 0 0 0 0 0 0 0 0 0 0 0 0 0 0 0 0 0 0 0 0 0 0 0 0 0 0 0 0 0 0 0 0 0 0 0 0 0 0 0 0 0 0

K03586 0 0 0 0 0 0 0 0 0 0 0 0 0 0 0 0 0 0 0 0 0 0 0 0 0 0 0 0 0 0 0 0 0 0 0 0 0 0 0 0 0 0 0 0 0 0 0 0

K03587 338 420 344 166 406 320 267 564 552 574 661 728 268 601 372 202 508 856 526 476 648 784 838 582 354 338 346 218 292 502 24 648 436 638 772 654 212 438 429 340 452 498 327 580 572 350 470 533

K03588 338 420 344 166 406 320 267 564 552 574 661 728 268 601 372 202 508 856 526 476 648 784 838 582 354 338 346 218 292 502 24 648 436 638 772 654 212 438 429 340 452 498 327 580 572 350 470 533

K03589 0 0 0 0 0 0 0 0 0 0 0 0 0 0 0 0 0 0 0 0 0 0 0 0 0 0 0 0 0 0 0 0 0 0 0 0 0 0 0 0 0 0 0 0 0 0 0 0

K03590 0 0 0 0 0 0 0 0 0 0 0 0 0 0 0 0 0 0 0 0 0 0 0 0 0 0 0 0 0 0 0 0 0 0 0 0 0 0 0 0 0 0 0 0 0 0 0 0

K03591 0 0 0 0 0 0 0 0 0 0 0 0 0 0 0 0 0 0 0 0 0 0 0 0 0 0 0 0 0 0 0 0 0 0 0 0 0 0 0 0 0 0 0 0 0 0 0 0

K03592 520 924 804 312 1136 944 468 1364 873 938 1367 1122 478 1058 878 366 1153 1592 912 1028 1322 1284 1568 1272 558 574 566 328 574 1449 24 1203 716 933 1276 1072 366 994 1210 825 1152 1228 589 1507 1542 783 972 1378

K03593 506 924 804 312 1106 928 459 1354 873 938 1364 1122 478 1058 862 366 1153 1578 912 1009 1322 1284 1568 1236 558 574 566 328 574 1432 24 1176 716 908 1276 1072 366 994 1210 825 1152 1220 589 1507 1542 783 972 1378

K03594 372 442 378 182 459 366 288 604 595 600 700 769 277 636 417 208 520 897 537 524 688 802 878 637 364 343 354 228 310 567 24 693 458 684 815 704 220 468 453 356 468 547 355 604 596 353 486 590

K03595 338 420 344 166 406 320 267 564 552 574 661 728 268 601 372 202 508 856 526 476 648 784 838 582 354 338 346 218 292 502 24 648 436 638 772 654 212 438 429 340 452 498 327 580 572 350 470 533

K03596 338 420 344 166 406 320 267 564 552 574 661 728 268 601 372 202 508 856 526 476 648 784 838 582 354 338 346 218 292 502 24 648 436 638 772 654 212 438 429 340 452 498 327 580 572 350 470 533

K03597 0 0 0 0 0 0 0 0 0 0 0 0 0 0 0 0 0 0 0 0 0 0 0 0 0 0 0 0 0 0 0 0 0 0 0 0 0 0 0 0 0 0 0 0 0 0 0 0

K03598 0 0 0 0 0 0 0 0 0 0 0 0 0 0 0 0 0 0 0 0 0 0 0 0 0 0 0 0 0 0 0 0 0 0 0 0 0 0 0 0 0 0 0 0 0 0 0 0

K03599 0 0 0 0 0 0 0 0 0 0 0 0 0 0 0 0 0 0 0 0 0 0 0 0 0 0 0 0 0 0 0 0 0 0 0 0 0 0 0 0 0 0 0 0 0 0 0 0

K03600 0 0 0 0 0 0 0 0 0 0 0 0 0 0 0 0 0 0 0 0 0 0 0 0 0 0 0 0 0 0 0 0 0 0 0 0 0 0 0 0 0 0 0 0 0 0 0 0

K03601 371 438 417 198 634 490 318 871 592 672 817 774 286 638 450 222 598 1037 573 490 714 918 922 734 354 364 359 218 314 728 24 682 464 680 813 682 240 463 494 446 604 604 327 658 643 490 514 706

K03602 371 438 417 198 634 490 318 871 592 672 817 774 286 638 450 222 598 1037 573 490 714 918 922 734 354 364 359 218 314 728 24 682 464 680 813 682 240 463 494 446 604 604 327 658 643 490 514 706

K03603 0 0 0 0 0 0 0 0 0 0 0 0 0 0 0 0 0 0 0 0 0 0 0 0 0 0 0 0 0 0 0 0 0 0 0 0 0 0 0 0 0 0 0 0 0 0 0 0

K03604 0 0 0 0 0 0 0 0 0 0 0 0 0 0 0 0 0 0 0 0 0 0 0 0 0 0 0 0 0 0 0 0 0 0 0 0 0 0 0 0 0 0 0 0 0 0 0 0

K03605 0 0 0 0 0 0 0 0 0 0 0 0 0 0 0 0 0 0 0 0 0 0 0 0 0 0 0 0 0 0 0 4 0 0 0 0 0 0 0 0 0 0 0 0 0 0 0 0

K03606 0 0 0 0 0 0 0 0 0 0 0 0 0 0 0 0 0 0 0 0 0 0 0 0 0 0 0 0 0 0 0 0 0 0 0 0 0 0 0 0 0 0 0 0 0 0 0 0

K03607 0 0 0 0 0 0 0 0 0 0 0 0 0 0 0 0 0 0 0 0 0 0 0 0 0 0 0 0 0 0 0 0 0 0 0 0 0 0 0 0 0 0 0 0 0 0 0 0

K03608 0 0 0 0 0 0 0 0 0 0 0 0 0 0 0 0 0 0 0 0 0 0 0 0 0 0 0 0 0 0 0 0 0 0 0 0 0 0 0 0 0 0 0 0 0 0 0 0

K03609 404 506 413 182 637 512 288 785 622 600 763 769 277 758 481 254 596 1020 537 603 788 838 1018 731 388 343 365 290 320 767 24 872 552 756 872 770 220 499 555 405 598 635 430 754 708 393 561 718

K03610 0 0 0 0 0 0 0 0 0 0 0 0 0 0 0 0 0 0 0 0 0 0 0 0 0 0 0 0 0 0 0 0 0 0 0 0 0 0 0 0 0 0 0 0 0 0 0 0

K03611 0 0 0 0 0 0 0 0 0 0 0 0 0 0 0 0 0 0 0 0 0 0 0 0 0 0 0 0 0 0 0 0 0 0 0 0 0 0 0 0 0 0 0 0 0 0 0 0

K03612 0 0 0 0 0 0 0 0 0 0 0 0 0 0 0 0 0 0 0 0 0 0 0 0 0 0 0 0 0 0 0 0 0 0 0 0 0 0 0 0 0 0 0 0 0 0 0 0

K03613 0 0 0 0 0 0 0 0 0 0 0 0 0 0 0 0 0 0 0 0 0 0 0 0 0 0 0 0 0 0 0 0 0 0 0 0 0 0 0 0 0 0 0 0 0 0 0 0

K03614 0 0 0 0 0 0 0 0 0 0 0 0 0 0 0 0 0 0 0 0 0 0 0 0 0 0 0 0 0 0 0 0 0 0 0 0 0 0 0 0 0 0 0 0 0 0 0 0

K03615 0 0 0 0 0 0 0 0 0 0 0 0 0 0 0 0 0 0 0 0 0 0 0 0 0 0 0 0 0 0 0 0 0 0 0 0 0 0 0 0 0 0 0 0 0 0 0 0

K03616 0 0 0 0 0 0 0 0 0 0 0 0 0 0 0 0 0 0 0 0 0 0 0 0 0 0 0 0 0 0 0 0 0 0 0 0 0 0 0 0 0 0 0 0 0 0 0 0

K03617 0 0 0 0 0 0 0 0 0 0 0 0 0 0 0 0 0 0 0 0 0 0 0 0 0 0 0 0 0 0 0 0 0 0 0 0 0 0 0 0 0 0 0 0 0 0 0 0

K03618 0 0 0 0 0 0 0 0 0 0 0 0 0 0 0 0 0 0 0 0 0 0 0 0 0 0 0 0 0 0 0 0 0 0 0 0 0 0 0 0 0 0 0 0 0 0 0 0

K03619 0 0 0 0 0 0 0 0 0 0 0 0 0 0 0 0 0 0 0 0 0 0 0 0 0 0 0 0 0 0 0 0 0 0 0 0 0 0 0 0 0 0 0 0 0 0 0 0

K03620 0 0 0 0 0 0 0 0 0 0 0 0 0 0 0 0 0 0 0 0 0 0 0 0 0 0 0 0 0 0 0 0 0 0 0 0 0 0 0 0 0 0 0 0 0 0 0 0

K03621 338 420 344 166 406 320 267 564 552 574 661 728 268 601 372 202 508 856 526 476 648 784 838 582 354 338 346 218 292 502 24 648 436 638 772 654 212 438 429 340 452 498 327 580 572 350 470 533

K03622 108 286 228 64 367 323 85 357 176 146 327 194 100 288 268 98 322 360 176 332 374 210 412 352 118 107 113 90 144 495 0 386 184 186 281 252 67 296 421 222 347 380 182 512 518 168 274 428

K03623 33 18 73 32 205 154 52 298 40 98 154 45 18 37 78 20 89 167 48 14 66 133 84 130 1 26 13 0 21 209 0 5 28 42 40 28 28 24 66 106 152 105 0 78 72 140 43 173

K03624 0 0 0 0 0 0 0 0 0 0 0 0 0 0 0 0 0 0 0 0 0 0 0 0 0 0 0 0 0 0 0 0 0 0 0 0 0 0 0 0 0 0 0 0 0 0 0 0

K03625 338 420 344 166 406 320 267 564 552 574 661 728 268 601 372 202 508 856 526 476 648 784 838 582 354 338 346 218 292 502 24 648 436 638 772 654 212 438 429 340 452 498 327 580 572 350 470 533

K03626 140 304 301 96 572 477 136 656 216 244 481 240 119 325 347 119 410 526 223 347 439 342 496 482 120 132 126 90 166 704 0 391 212 228 322 280 96 320 486 328 500 486 182 590 590 308 318 602

K03627 140 304 301 96 572 477 136 656 216 244 481 240 119 325 347 119 410 526 223 347 439 342 496 482 120 132 126 90 166 704 0 391 212 228 322 280 96 320 486 328 500 486 182 590 590 308 318 602

K03628 0 0 0 0 0 0 0 0 0 0 0 0 0 0 0 0 0 0 0 0 0 0 0 0 0 0 0 0 0 0 0 0 0 0 0 0 0 0 0 0 0 0 0 0 0 0 0 0

K03629 0 0 0 0 0 0 0 0 0 0 0 0 0 0 0 0 0 0 0 0 0 0 0 0 0 0 0 0 0 0 0 0 0 0 0 0 0 0 0 0 0 0 0 0 0 0 0 0

K03630 0 0 0 0 24 16 0 9 0 0 3 0 0 0 0 0 0 14 0 0 0 0 0 23 0 0 0 0 0 18 0 30 0 0 0 0 0 0 0 0 0 0 0 0 0 0 0 0

K03631 338 420 344 166 406 320 267 564 552 574 661 728 268 601 372 202 508 856 526 476 648 784 838 582 354 338 346 218 292 502 24 648 436 638 772 654 212 438 429 340 452 498 327 580 572 350 470 533

K03632 0 0 0 0 0 0 0 0 0 0 0 0 0 0 0 0 0 0 0 0 0 0 0 0 0 0 0 0 0 0 0 0 0 0 0 0 0 0 0 0 0 0 0 0 0 0 0 0

K03633 0 0 0 0 0 0 0 0 0 0 0 0 0 0 0 0 0 0 0 0 0 0 0 0 0 0 0 0 0 0 0 0 0 0 0 0 0 0 0 0 0 0 0 0 0 0 0 0

K03634 0 0 0 0 0 0 0 0 0 0 0 0 0 0 0 0 0 0 0 0 0 0 0 0 0 0 0 0 0 0 0 0 0 0 0 0 0 0 0 0 0 0 0 0 0 0 0 0

K03635 478 724 645 262 1000 814 404 1228 768 818 1144 968 386 926 719 320 919 1396 748 822 1088 1127 1335 1086 473 471 472 308 458 1224 24 1068 648 866 1094 934 308 759 916 668 952 984 510 1170 1161 658 788 1134

K03636 454 884 697 264 900 761 395 1034 789 813 1177 1036 450 986 755 339 1052 1398 854 965 1216 1133 1445 1110 546 544 545 318 536 1192 24 1182 666 845 1193 994 329 940 1121 703 984 1074 561 1404 1446 640 913 1148

K03637 478 724 645 262 978 798 404 1219 768 818 1142 968 386 926 719 320 919 1382 748 822 1088 1127 1335 1064 473 471 472 308 458 1206 24 1038 648 866 1094 934 308 759 916 668 952 984 510 1170 1161 658 788 1134

K03638 478 724 645 262 978 798 404 1219 768 818 1142 968 386 926 719 320 919 1382 748 822 1088 1127 1335 1064 473 471 472 308 458 1206 24 1038 648 866 1094 934 308 759 916 668 952 984 510 1170 1161 658 788 1134

K03639 478 724 645 262 978 798 404 1219 768 818 1142 968 386 926 719 320 919 1382 748 822 1088 1127 1335 1064 473 471 472 308 458 1206 24 1038 648 866 1094 934 308 759 916 668 952 984 510 1170 1161 658 788 1134

K03640 0 0 0 0 0 0 0 0 0 0 0 0 0 0 0 0 0 0 0 0 0 0 0 0 0 0 0 0 0 0 0 0 0 0 0 0 0 0 0 0 0 0 0 0 0 0 0 0

K03641 0 0 0 0 0 0 0 0 0 0 0 0 0 0 0 0 0 0 0 0 0 0 0 0 0 0 0 0 0 0 0 0 0 0 0 0 0 0 0 0 0 0 0 0 0 0 0 0

K03642 338 420 344 166 406 320 267 564 552 574 661 728 268 601 372 202 508 856 526 476 648 784 838 582 354 338 346 218 292 502 24 648 436 638 772 654 212 438 429 340 452 498 327 580 572 350 470 533

K03643 0 0 0 0 0 0 0 0 0 0 0 0 0 0 0 0 0 0 0 0 0 0 0 0 0 0 0 0 0 0 0 0 0 0 0 0 0 0 0 0 0 0 0 0 0 0 0 0

K03644 338 420 344 166 406 320 267 564 552 574 661 728 268 601 372 202 508 856 526 476 648 784 838 582 354 338 346 218 292 502 24 648 436 638 772 654 212 438 429 340 452 498 327 580 572 350 470 533

K03645 0 0 0 0 0 0 0 0 0 0 0 0 0 0 0 0 0 0 0 0 0 0 0 0 0 0 0 0 0 0 0 0 0 0 0 0 0 0 0 0 0 0 0 0 0 0 0 0

K03646 0 0 0 0 0 0 0 0 0 0 0 0 0 0 0 0 0 0 0 0 0 0 0 0 0 0 0 0 0 0 0 0 0 0 0 0 0 0 0 0 0 0 0 0 0 0 0 0

K03647 0 0 0 0 0 0 0 0 0 0 0 0 0 0 0 0 0 0 0 0 0 0 0 0 0 0 0 0 0 0 0 0 0 0 0 0 0 0 0 0 0 0 0 0 0 0 0 0

K03648 33 18 73 32 205 154 52 298 40 98 154 45 18 37 78 20 89 167 48 14 66 133 84 130 1 26 13 0 21 209 0 5 28 42 40 28 28 24 66 106 152 105 0 78 72 140 43 173

K03649 0 0 0 0 1 0 0 0 0 0 0 0 0 0 0 0 0 0 0 0 0 0 0 0 0 0 0 0 0 0 0 0 0 0 0 0 0 0 0 0 0 0 0 0 0 0 0 0

K03650 338 420 344 166 406 320 267 564 552 574 661 728 268 601 372 202 508 856 526 476 648 784 838 582 354 338 346 218 292 502 24 648 436 638 772 654 212 438 429 340 452 498 327 580 572 350 470 533

K03651 0 0 0 0 0 0 0 0 0 0 0 0 0 0 0 0 0 0 0 0 0 0 0 0 0 0 0 0 0 0 0 0 0 0 0 0 0 0 0 0 0 0 0 0 0 0 0 0

K03652 42 200 159 49 182 163 64 154 106 120 225 154 91 132 160 46 234 224 164 205 234 156 234 214 84 102 94 19 116 256 0 164 68 67 182 137 58 236 295 157 201 244 80 337 382 125 184 244

K03653 116 464 353 98 448 408 128 452 238 239 511 308 182 384 383 138 544 514 328 490 568 348 606 482 192 205 199 100 244 656 0 475 230 207 420 340 117 502 692 363 532 576 234 824 875 290 442 614

K03654 338 420 344 166 452 352 267 582 552 574 664 728 268 601 372 202 508 884 526 476 648 784 838 610 354 338 346 218 292 532 24 681 436 638 772 654 212 438 429 340 452 498 327 580 572 350 470 533

K03655 338 420 344 166 476 368 267 590 552 574 666 728 268 601 372 202 508 898 526 476 648 784 838 634 354 338 346 218 292 550 24 708 436 638 772 654 212 438 429 340 452 498 327 580 572 350 470 533

K03656 0 0 0 0 0 0 0 0 0 0 0 0 0 0 0 0 0 0 0 0 0 0 0 0 0 0 0 0 0 0 0 0 0 0 0 0 0 0 0 0 0 0 0 0 0 0 0 0

K03657 493 678 683 294 1057 837 464 1354 782 916 1234 1013 405 842 750 294 932 1454 796 776 1054 1224 1280 1142 450 496 474 246 468 1246 24 898 582 860 1078 896 336 753 879 725 974 1010 434 1098 1120 758 756 1180

K03658 33 18 73 32 205 154 52 298 40 98 154 45 18 37 78 20 89 167 48 14 66 133 84 130 1 26 13 0 21 209 0 5 28 42 40 28 28 24 66 106 152 105 0 78 72 140 43 173

K03660 166 144 249 94 696 546 145 858 194 249 450 171 56 266 311 100 278 539 118 204 310 336 386 482 46 60 53 80 87 752 0 307 195 249 222 220 74 140 281 293 466 395 131 356 304 327 192 588

K03664 338 420 344 166 406 320 267 564 552 574 661 728 268 601 372 202 508 856 526 476 648 784 838 582 354 338 346 218 292 502 24 648 436 638 772 654 212 438 429 340 452 498 327 580 572 350 470 533

K03665 412 684 538 216 695 582 331 872 684 694 947 882 358 854 596 294 818 1160 690 760 982 976 1212 855 462 441 451 300 420 914 24 958 598 778 1011 858 270 704 826 546 784 830 482 1067 1065 514 729 904

K03666 0 0 0 0 0 0 0 0 0 0 0 0 0 0 0 0 0 0 0 0 0 0 0 0 0 0 0 0 0 0 0 0 0 0 0 0 0 0 0 0 0 0 0 0 0 0 0 0

K03667 0 0 0 0 0 0 0 0 0 0 0 0 0 0 0 0 0 0 0 0 0 0 0 0 0 0 0 0 0 0 0 0 0 0 0 0 0 0 0 0 0 0 0 0 0 0 0 0

K03668 0 0 0 0 0 0 0 0 0 0 0 0 0 0 0 0 0 0 0 0 0 0 0 0 0 0 0 0 0 0 0 0 0 0 0 0 0 0 0 0 0 0 0 0 0 0 0 0

K03669 0 0 0 0 0 0 0 0 0 0 0 0 0 0 0 0 0 0 0 0 0 0 0 0 0 0 0 0 0 0 0 0 0 0 0 0 0 0 0 0 0 0 0 0 0 0 0 0

K03670 0 0 0 0 0 0 0 0 0 0 0 0 0 0 0 0 0 0 0 0 0 0 0 0 0 0 0 0 0 0 0 0 0 0 0 0 0 0 0 0 0 0 0 0 0 0 0 0

K03671 370 484 379 166 608 483 267 754 578 574 727 728 268 722 436 248 584 993 526 555 748 820 978 716 377 338 358 280 304 724 24 887 530 711 829 720 212 469 531 389 582 586 402 730 684 390 545 660

K03672 0 0 0 0 0 0 0 0 0 0 0 0 0 0 0 0 0 0 0 0 0 0 0 0 0 0 0 0 0 0 0 0 0 0 0 0 0 0 0 0 0 0 0 0 0 0 0 0

K03673 0 0 0 0 0 0 0 0 0 0 0 0 0 0 0 0 0 0 0 0 0 0 0 0 0 0 0 0 0 0 0 0 0 0 0 0 0 0 0 0 0 0 0 0 0 0 0 0

K03674 0 0 0 0 0 0 0 0 0 0 0 0 0 0 0 0 0 0 0 0 0 0 0 0 0 0 0 0 0 0 0 0 0 0 0 0 0 0 0 0 0 0 0 0 0 0 0 0

K03675 0 0 0 0 0 0 0 0 0 0 0 0 0 0 0 0 0 0 0 0 0 0 0 0 0 0 0 0 0 0 0 0 0 0 0 0 0 0 0 0 0 0 0 0 0 0 0 0

K03676 0 0 0 0 0 0 0 0 0 0 0 0 0 0 0 0 0 0 0 0 0 0 0 0 0 0 0 0 0 0 0 0 0 0 0 0 0 0 0 0 0 0 0 0 0 0 0 0

K03677 0 0 0 0 0 0 0 0 0 0 0 0 0 0 0 0 0 0 0 0 0 0 0 0 0 0 0 0 0 0 0 0 0 0 0 0 0 0 0 0 0 0 0 0 0 0 0 0

K03679 140 304 301 96 548 461 136 646 216 244 478 240 119 325 347 119 410 512 223 347 439 342 496 459 120 132 126 90 166 686 0 361 212 228 322 280 96 320 486 328 500 486 182 590 590 308 318 602

K03680 74 264 194 49 314 278 64 317 132 120 289 154 91 253 224 92 310 318 164 284 334 192 373 296 108 102 105 81 128 430 0 340 162 140 238 203 58 266 397 206 332 332 154 488 494 165 258 371

K03683 0 0 0 0 0 0 0 0 0 0 0 0 0 0 0 0 0 0 0 0 0 0 0 0 0 0 0 0 0 0 0 0 0 0 0 0 0 0 0 0 0 0 0 0 0 0 0 0

K03684 0 0 0 0 0 0 0 0 0 0 0 0 0 0 0 0 0 0 0 0 0 0 0 0 0 0 0 0 0 0 0 0 0 0 0 0 0 0 0 0 0 0 0 0 0 0 0 0

K03685 390 460 451 214 706 552 331 920 636 698 856 814 296 673 480 228 610 1092 584 520 754 935 962 800 365 368 367 228 330 811 24 754 486 702 856 732 249 493 518 462 620 644 355 682 668 493 530 764

K03686 446 660 610 262 848 683 404 1056 741 818 1081 968 386 804 655 274 844 1288 748 743 988 1092 1196 998 450 471 460 246 447 1036 24 892 554 793 1038 868 308 728 814 619 821 896 434 1020 1049 618 714 1007

K03687 446 660 610 262 848 683 404 1056 741 818 1081 968 386 804 655 274 844 1288 748 743 988 1092 1196 998 450 471 460 246 447 1036 24 892 554 793 1038 868 308 728 814 619 821 896 434 1020 1049 618 714 1007

K03688 100 58 180 79 488 370 124 646 124 223 348 130 46 109 202 48 190 389 106 77 171 284 208 338 12 56 34 9 59 500 0 82 78 130 124 104 66 79 155 228 320 258 28 182 168 284 102 404

K03689 0 0 0 0 0 0 0 0 0 0 0 0 0 0 0 0 0 0 0 0 0 0 0 0 0 0 0 0 0 0 0 0 0 0 0 0 0 0 0 0 0 0 0 0 0 0 0 0

K03690 0 0 0 0 0 0 0 0 0 0 0 0 0 0 0 0 0 0 0 0 0 0 0 0 0 0 0 0 0 0 0 0 0 0 0 0 0 0 0 0 0 0 0 0 0 0 0 0

K03692 0 0 0 0 0 0 0 0 0 0 0 0 0 0 0 0 0 0 0 0 0 0 0 0 0 0 0 0 0 0 0 0 0 0 0 0 0 0 0 0 0 0 0 0 0 0 0 0

K03693 0 0 0 0 0 0 0 0 0 0 0 0 0 0 0 0 0 0 0 0 0 0 0 0 0 0 0 0 0 0 0 0 0 0 0 0 0 0 0 0 0 0 0 0 0 0 0 0

K03694 0 0 0 0 0 0 0 0 0 0 0 0 0 0 0 0 0 0 0 0 0 0 0 0 0 0 0 0 0 0 0 0 0 0 0 0 0 0 0 0 0 0 0 0 0 0 0 0

K03695 338 420 344 166 406 320 267 564 552 574 661 728 268 601 372 202 508 856 526 476 648 784 838 582 354 338 346 218 292 502 24 648 436 638 772 654 212 438 429 340 452 498 327 580 572 350 470 533

K03696 0 0 0 0 0 0 0 0 0 0 0 0 0 0 0 0 0 0 0 0 0 0 0 0 0 0 0 0 0 0 0 0 0 0 0 0 0 0 0 0 0 0 0 0 0 0 0 0

K03697 0 0 0 0 0 0 0 0 0 0 0 0 0 0 0 0 0 0 0 0 0 0 0 0 0 0 0 0 0 0 0 0 0 0 0 0 0 0 0 0 0 0 0 0 0 0 0 0

K03698 61 222 193 64 228 208 76 194 149 146 264 194 100 166 188 52 246 264 176 234 274 174 273 274 95 107 101 28 134 326 0 240 90 88 224 186 67 266 319 173 216 284 108 362 406 128 200 301

K03699 33 18 73 32 252 186 52 316 40 98 156 45 18 37 78 20 89 195 48 14 66 133 84 159 1 26 13 0 21 240 0 35 28 42 40 28 28 24 66 106 152 105 0 78 72 140 43 173

K03700 0 0 0 0 0 0 0 0 0 0 0 0 0 0 0 0 0 0 0 0 0 0 0 0 0 0 0 0 0 0 0 0 0 0 0 0 0 0 0 0 0 0 0 0 0 0 0 0

K03701 438 478 524 246 893 690 391 1210 676 797 1009 859 314 710 574 249 698 1245 632 552 820 1068 1046 920 366 394 380 228 352 1002 24 733 514 768 896 759 278 518 584 568 772 757 355 761 739 633 572 936

K03702 404 460 451 214 688 536 340 911 636 698 856 814 296 673 496 228 610 1078 584 538 754 935 962 790 365 368 367 228 330 794 24 728 486 726 856 732 249 493 518 462 620 652 355 682 668 493 530 764

K03703 404 460 451 214 688 536 340 911 636 698 856 814 296 673 496 228 610 1078 584 538 754 935 962 790 365 368 367 228 330 794 24 728 486 726 856 732 249 493 518 462 620 652 355 682 668 493 530 764

K03704 338 420 344 166 406 320 267 564 552 574 661 728 268 601 372 202 508 856 526 476 648 784 838 582 354 338 346 218 292 502 24 648 436 638 772 654 212 438 429 340 452 498 327 580 572 350 470 533

K03705 0 0 0 0 0 0 0 0 0 0 0 0 0 0 0 0 0 0 0 0 0 0 0 0 0 0 0 0 0 0 0 0 0 0 0 0 0 0 0 0 0 0 0 0 0 0 0 0

K03706 0 0 0 0 0 0 0 0 0 0 0 0 0 0 0 0 0 0 0 0 0 0 0 0 0 0 0 0 0 0 0 0 0 0 0 0 0 0 0 0 0 0 0 0 0 0 0 0

K03707 0 0 0 0 0 0 0 0 0 0 0 0 0 0 0 0 0 0 0 0 0 0 0 0 0 0 0 0 0 0 0 0 0 0 0 0 0 0 0 0 0 0 0 0 0 0 0 0

K03708 0 0 0 0 0 0 0 0 0 0 0 0 0 0 0 0 0 0 0 0 0 0 0 0 0 0 0 0 0 0 0 0 0 0 0 0 0 0 0 0 0 0 0 0 0 0 0 0

K03709 553 942 877 344 1387 1130 519 1680 914 1036 1523 1167 496 1094 957 386 1242 1787 960 1042 1388 1416 1652 1448 558 599 579 328 596 1693 24 1268 744 976 1316 1099 394 1019 1276 931 1305 1333 589 1586 1614 923 1015 1551

K03710 0 0 0 0 0 0 0 0 0 0 0 0 0 0 0 0 0 0 0 0 0 0 0 0 0 0 0 0 0 0 0 0 0 0 0 0 0 0 0 0 0 0 0 0 0 0 0 0

K03711 338 420 344 166 406 320 267 564 552 574 661 728 268 601 372 202 508 856 526 476 648 784 838 582 354 338 346 218 292 502 24 648 436 638 772 654 212 438 429 340 452 498 327 580 572 350 470 533

K03712 20 22 34 15 47 46 12 40 44 26 41 40 10 35 29 6 12 41 12 30 40 18 40 42 10 4 8 9 17 65 0 46 22 21 42 50 8 30 24 16 16 40 28 24 24 4 16 58

K03713 0 0 0 0 0 0 0 0 0 0 0 0 0 0 0 0 0 0 0 0 0 0 0 0 0 0 0 0 0 0 0 0 0 0 0 0 0 0 0 0 0 0 0 0 0 0 0 0

K03715 0 0 0 0 0 0 0 0 0 0 0 0 0 0 0 0 0 0 0 0 0 0 0 0 0 0 0 0 0 0 0 0 0 0 0 0 0 0 0 0 0 0 0 0 0 0 0 0

K03716 0 0 0 0 0 0 0 0 0 0 0 0 0 0 0 0 0 0 0 0 0 0 0 0 0 0 0 0 0 0 0 0 0 0 0 0 0 0 0 0 0 0 0 0 0 0 0 0

K03717 0 0 0 0 0 0 0 0 0 0 0 0 0 0 0 0 0 0 0 0 0 0 0 0 0 0 0 0 0 0 0 0 0 0 0 0 0 0 0 0 0 0 0 0 0 0 0 0

K03718 478 835 750 286 1050 846 428 1300 832 882 1177 1072 422 956 816 320 1054 1476 772 914 1182 1152 1348 1081 494 471 482 308 488 1332 24 1084 702 889 1208 968 308 790 1041 790 1126 1141 571 1380 1344 697 894 1190

K03719 0 0 0 0 0 0 0 0 0 0 0 0 0 0 0 0 0 0 0 0 0 0 0 0 0 0 0 0 0 0 0 0 0 0 0 0 0 0 0 0 0 0 0 0 0 0 0 0

K03720 0 0 0 0 0 0 0 0 0 0 0 0 0 0 0 0 0 0 0 0 0 0 0 0 0 0 0 0 0 0 0 0 0 0 0 0 0 0 0 0 0 0 0 0 0 0 0 0

K03721 0 0 0 0 0 0 0 0 0 0 0 0 0 0 0 0 0 0 0 0 0 0 0 0 0 0 0 0 0 0 0 0 0 0 0 0 0 0 0 0 0 0 0 0 0 0 0 0

K03722 0 0 0 0 0 0 0 0 0 0 0 0 0 0 0 0 0 0 0 0 0 0 0 0 0 0 0 0 0 0 0 0 0 0 0 0 0 0 0 0 0 0 0 0 0 0 0 0

K03723 338 420 344 166 406 320 267 564 552 574 661 728 268 601 372 202 508 856 526 476 648 784 838 582 354 338 346 218 292 502 24 648 436 638 772 654 212 438 429 340 452 498 327 580 572 350 470 533

K03724 580 988 839 312 1298 1075 476 1536 900 938 1430 1122 478 1179 958 412 1228 1701 912 1144 1421 1319 1708 1374 581 574 577 390 586 1636 24 1382 810 1055 1332 1138 366 1025 1312 874 1283 1324 664 1658 1654 823 1046 1506

K03725 66 40 107 47 282 216 72 348 84 124 195 86 28 72 124 27 101 222 59 62 106 150 124 208 12 30 21 9 38 292 0 80 50 88 83 77 37 54 90 122 168 154 28 103 96 144 59 230

K03726 140 304 301 96 572 477 136 656 216 244 481 240 119 325 347 119 410 526 223 347 439 342 496 482 120 132 126 90 166 704 0 391 212 228 322 280 96 320 486 328 500 486 182 590 590 308 318 602

K03727 0 0 0 0 0 0 0 0 0 0 0 0 0 0 0 0 0 0 0 0 0 0 0 0 0 0 0 0 0 0 0 0 0 0 0 0 0 0 0 0 0 0 0 0 0 0 0 0

K03731 0 0 0 0 0 0 0 0 0 0 0 0 0 0 0 0 0 0 0 0 0 0 0 0 0 0 0 0 0 0 0 0 0 0 0 0 0 0 0 0 0 0 0 0 0 0 0 0

K03732 0 0 0 0 0 0 0 0 0 0 0 0 0 0 0 0 0 0 0 0 0 0 0 0 0 0 0 0 0 0 0 0 0 0 0 0 0 0 0 0 0 0 0 0 0 0 0 0

K03733 338 420 344 166 430 336 267 572 552 574 664 728 268 601 372 202 508 870 526 476 648 784 838 604 354 338 346 218 292 520 24 678 436 638 772 654 212 438 429 340 452 498 327 580 572 350 470 533

K03734 0 0 0 0 0 0 0 0 0 0 0 0 0 0 0 0 0 0 0 0 0 0 0 0 0 0 0 0 0 0 0 0 0 0 0 0 0 0 0 0 0 0 0 0 0 0 0 0

K03735 0 0 0 0 0 0 0 0 0 0 0 0 0 0 0 0 0 0 0 0 0 0 0 0 0 0 0 0 0 0 0 0 0 0 0 0 0 0 0 0 0 0 0 0 0 0 0 0

K03736 0 0 0 0 0 0 0 0 0 0 0 0 0 0 0 0 0 0 0 0 0 0 0 0 0 0 0 0 0 0 0 0 0 0 0 0 0 0 0 0 0 0 0 0 0 0 0 0

K03737 0 0 0 0 0 0 0 0 0 0 0 0 0 0 0 0 0 0 0 0 0 0 0 0 0 0 0 0 0 0 0 0 0 0 0 0 0 0 0 0 0 0 0 0 0 0 0 0

K03738 454 995 802 288 950 794 420 1106 854 877 1213 1140 485 1016 852 339 1187 1478 877 1057 1310 1158 1458 1121 567 544 555 318 566 1306 24 1228 719 868 1307 1028 329 970 1246 825 1160 1232 622 1614 1630 678 1019 1202

K03739 0 0 0 0 0 0 0 0 0 0 0 0 0 0 0 0 0 0 0 0 0 0 0 0 0 0 0 0 0 0 0 0 0 0 0 0 0 0 0 0 0 0 0 0 0 0 0 0

K03740 0 0 0 0 0 0 0 0 0 0 0 0 0 0 0 0 0 0 0 0 0 0 0 0 0 0 0 0 0 0 0 0 0 0 0 0 0 0 0 0 0 0 0 0 0 0 0 0

K03741 352 420 344 166 460 352 276 582 552 574 664 728 268 601 388 202 508 884 526 494 648 784 838 624 354 338 346 218 292 532 24 678 436 662 772 654 212 438 429 340 452 507 327 580 572 350 470 533

K03742 380 620 503 216 564 468 331 708 657 694 886 882 358 732 532 247 742 1066 690 680 883 941 1072 790 438 441 440 238 409 745 24 812 504 705 954 792 270 674 724 497 653 742 406 916 953 474 654 776

K03743 0 0 0 0 0 0 0 0 0 0 0 0 0 0 0 0 0 0 0 0 0 0 0 0 0 0 0 0 0 0 0 0 0 0 0 0 0 0 0 0 0 0 0 0 0 0 0 0

K03744 52 40 107 47 276 216 64 348 84 124 195 86 28 72 108 27 101 222 59 44 106 150 124 195 12 30 21 9 38 292 0 80 50 64 83 77 37 54 90 122 168 145 28 103 96 144 59 230

K03745 0 0 0 0 0 0 0 0 0 0 0 0 0 0 0 0 0 0 0 0 0 0 0 0 0 0 0 0 0 0 0 0 0 0 0 0 0 0 0 0 0 0 0 0 0 0 0 0

K03746 0 0 0 0 0 0 0 0 0 0 0 0 0 0 0 0 0 0 0 0 0 0 0 0 0 0 0 0 0 0 0 0 0 0 0 0 0 0 0 0 0 0 0 0 0 0 0 0

K03747 0 0 0 0 0 0 0 0 0 0 0 0 0 0 0 0 0 0 0 0 0 0 0 0 0 0 0 0 0 0 0 0 0 0 0 0 0 0 0 0 0 0 0 0 0 0 0 0

K03748 0 0 0 0 0 0 0 0 0 0 0 0 0 0 0 0 0 0 0 0 0 0 0 0 0 0 0 0 0 0 0 0 0 0 0 0 0 0 0 0 0 0 0 0 0 0 0 0

K03749 0 0 0 0 0 0 0 0 0 0 0 0 0 0 0 0 0 0 0 0 0 0 0 0 0 0 0 0 0 0 0 0 0 0 0 0 0 0 0 0 0 0 0 0 0 0 0 0

K03750 545 764 752 310 1260 1013 476 1566 852 942 1336 1054 414 998 842 348 1020 1604 808 885 1193 1278 1458 1255 484 501 493 318 496 1493 24 1089 699 954 1177 1012 344 814 1005 790 1120 1138 538 1273 1257 802 847 1365

K03751 0 0 0 0 0 0 0 0 0 0 0 0 0 0 0 0 0 0 0 0 0 0 0 0 0 0 0 0 0 0 0 0 0 0 0 0 0 0 0 0 0 0 0 0 0 0 0 0

K03752 478 724 645 262 978 798 404 1219 768 818 1142 968 386 926 719 320 919 1382 748 822 1088 1127 1335 1064 473 471 472 308 458 1206 24 1038 648 866 1094 934 308 759 916 668 952 984 510 1170 1161 658 788 1134

K03753 478 724 645 262 954 782 404 1210 768 818 1139 968 386 926 719 320 919 1368 748 822 1088 1127 1335 1040 473 471 472 308 458 1188 24 1008 648 866 1094 934 308 759 916 668 952 984 510 1170 1161 658 788 1134

K03755 0 0 0 0 0 0 0 0 0 0 0 0 0 0 0 0 0 0 0 0 0 0 0 0 0 0 0 0 0 0 0 0 0 0 0 0 0 0 0 0 0 0 0 0 0 0 0 0

K03756 0 0 0 0 0 0 0 0 0 0 0 0 0 0 0 0 0 0 0 0 0 0 0 0 0 0 0 0 0 0 0 0 0 0 0 0 0 0 0 0 0 0 0 0 0 0 0 0

K03757 0 0 0 0 0 0 0 0 0 0 0 0 0 0 0 0 0 0 0 0 0 0 0 0 0 0 0 0 0 0 0 0 0 0 0 0 0 0 0 0 0 0 0 0 0 0 0 0

K03758 0 0 0 0 0 0 0 0 0 0 0 0 0 0 0 0 0 0 0 0 0 0 0 0 0 0 0 0 0 0 0 0 0 0 0 0 0 0 0 0 0 0 0 0 0 0 0 0

K03759 0 0 0 0 0 0 0 0 0 0 0 0 0 0 0 0 0 0 0 0 0 0 0 0 0 0 0 0 0 0 0 0 0 0 0 0 0 0 0 0 0 0 0 0 0 0 0 0

K03760 0 0 0 0 0 0 0 0 0 0 0 0 0 0 0 0 0 0 0 0 0 0 0 0 0 0 0 0 0 0 0 0 0 0 0 0 0 0 0 0 0 0 0 0 0 0 0 0

K03761 0 0 0 0 0 0 0 0 0 0 0 0 0 0 0 0 0 0 0 0 0 0 0 0 0 0 0 0 0 0 0 0 0 0 0 0 0 0 0 0 0 0 0 0 0 0 0 0

K03762 0 0 0 0 0 0 0 0 0 0 0 0 0 0 0 0 0 0 0 0 0 0 0 0 0 0 0 0 0 0 0 0 0 0 0 0 0 0 0 0 0 0 0 0 0 0 0 0

K03763 33 18 73 32 205 154 52 298 40 98 154 45 18 37 78 20 89 167 48 14 66 133 84 130 1 26 13 0 21 209 0 5 28 42 40 28 28 24 66 106 152 105 0 78 72 140 43 173

K03764 0 0 0 0 0 0 0 0 0 0 0 0 0 0 0 0 0 0 0 0 0 0 0 0 0 0 0 0 0 0 0 0 0 0 0 0 0 0 0 0 0 0 0 0 0 0 0 0

K03765 0 0 0 0 0 0 0 0 0 0 0 0 0 0 0 0 0 0 0 0 0 0 0 0 0 0 0 0 0 0 0 0 0 0 0 0 0 0 0 0 0 0 0 0 0 0 0 0

K03767 0 0 0 0 0 0 0 0 0 0 0 0 0 0 0 0 0 0 0 0 0 0 0 0 0 0 0 0 0 0 0 0 0 0 0 0 0 0 0 0 0 0 0 0 0 0 0 0

K03768 404 460 451 214 664 520 340 902 636 698 853 814 296 673 496 228 610 1064 584 538 754 935 962 767 365 368 367 228 330 776 24 698 486 726 856 732 249 493 518 462 620 652 355 682 668 493 530 764

K03769 0 0 0 0 1 0 0 0 0 0 0 0 0 0 0 0 0 0 0 0 0 0 0 17 0 0 0 0 0 4 0 30 0 0 0 0 0 0 0 0 0 0 0 0 0 0 0 0

K03770 0 0 0 0 0 0 0 0 0 0 0 0 0 0 0 0 0 0 0 0 0 0 0 0 0 0 0 0 0 0 0 0 0 0 0 0 0 0 0 0 0 0 0 0 0 0 0 0

K03771 0 0 0 0 0 0 0 0 0 0 0 0 0 0 0 0 0 0 0 0 0 0 0 0 0 0 0 0 0 0 0 0 0 0 0 0 0 0 0 0 0 0 0 0 0 0 0 0

K03772 0 0 0 0 0 0 0 0 0 0 0 0 0 0 0 0 0 0 0 0 0 0 0 0 0 0 0 0 0 0 0 0 0 0 0 0 0 0 0 0 0 0 0 0 0 0 0 0

K03773 0 0 0 0 0 0 0 0 0 0 0 0 0 0 0 0 0 0 0 0 0 0 0 0 0 0 0 0 0 0 0 0 0 0 0 0 0 0 0 0 0 0 0 0 0 0 0 0

K03774 0 0 0 0 0 0 0 0 0 0 0 0 0 0 0 0 0 0 0 0 0 0 0 0 0 0 0 0 0 0 0 0 0 0 0 0 0 0 0 0 0 0 0 0 0 0 0 0

K03775 437 524 486 214 819 650 340 1074 662 698 917 814 296 794 560 275 685 1173 584 618 853 970 1102 855 388 368 378 290 342 963 24 874 580 799 912 798 249 524 620 511 750 740 430 833 780 533 604 891

K03776 0 0 0 0 0 0 0 0 0 0 0 0 0 0 0 0 0 0 0 0 0 0 0 0 0 0 0 0 0 0 0 0 0 0 0 0 0 0 0 0 0 0 0 0 0 0 0 0

K03777 0 0 0 0 0 0 0 0 0 0 0 0 0 0 0 0 0 0 0 0 0 0 0 0 0 0 0 0 0 0 0 0 0 0 0 0 0 0 0 0 0 0 0 0 0 0 0 0

K03778 0 0 0 0 0 0 0 0 0 0 0 0 0 0 0 0 0 0 0 0 0 0 0 0 0 0 0 0 0 0 0 0 0 0 0 0 0 0 0 0 0 0 0 0 0 0 0 0

K03779 0 0 0 0 0 0 0 0 0 0 0 0 0 0 0 0 0 0 0 0 0 0 0 0 0 0 0 0 0 0 0 0 0 0 0 0 0 0 0 0 0 0 0 0 0 0 0 0

K03780 0 0 0 0 0 0 0 0 0 0 0 0 0 0 0 0 0 0 0 0 0 0 0 0 0 0 0 0 0 0 0 0 0 0 0 0 0 0 0 0 0 0 0 0 0 0 0 0

K03781 0 0 0 0 23 16 0 9 0 0 0 0 0 0 0 0 0 14 0 0 0 0 0 23 0 0 0 0 0 18 0 30 0 0 0 0 0 0 0 0 0 0 0 0 0 0 0 0

K03782 399 752 642 254 686 562 368 830 766 784 965 1027 404 798 657 254 890 1200 724 802 1017 983 1125 866 470 446 458 246 456 941 24 933 580 749 1111 874 279 734 874 635 844 940 496 1151 1160 517 776 889

K03783 0 0 0 0 0 0 0 0 0 0 0 0 0 0 0 0 0 0 0 0 0 0 0 0 0 0 0 0 0 0 0 0 0 0 0 0 0 0 0 0 0 0 0 0 0 0 0 0

K03784 0 0 0 0 0 0 0 0 0 0 0 0 0 0 0 0 0 0 0 0 0 0 0 0 0 0 0 0 0 0 0 0 0 0 0 0 0 0 0 0 0 0 0 0 0 0 0 0

K03785 99 104 142 47 414 330 72 511 110 124 256 86 28 194 188 74 176 317 59 142 204 186 263 274 35 30 32 71 49 461 0 226 144 161 140 143 37 85 192 171 298 242 103 254 208 184 134 358

K03786 0 0 0 0 0 0 0 0 0 0 0 0 0 0 0 0 0 0 0 0 0 0 0 0 0 0 0 0 0 0 0 0 0 0 0 0 0 0 0 0 0 0 0 0 0 0 0 0

K03787 470 542 559 246 1024 804 391 1373 702 797 1070 859 314 832 638 296 774 1340 632 632 918 1104 1186 985 390 394 392 290 362 1172 24 879 608 842 952 825 278 548 686 617 903 845 430 912 851 673 647 1064

K03788 0 0 0 0 0 0 0 0 0 0 0 0 0 0 0 0 0 0 0 0 0 0 0 0 0 0 0 0 0 0 0 0 0 0 0 0 0 0 0 0 0 0 0 0 0 0 0 0

K03789 478 724 645 262 1000 814 404 1228 768 818 1142 968 386 926 719 320 919 1396 748 822 1088 1127 1335 1086 473 471 472 308 458 1224 24 1068 648 866 1094 934 308 759 916 668 952 984 510 1170 1161 658 788 1134

K03790 0 0 0 0 0 0 0 0 0 0 0 0 0 0 0 0 0 0 0 0 0 0 0 0 0 0 0 0 0 0 0 0 0 0 0 0 0 0 0 0 0 0 0 0 0 0 0 0

K03791 0 0 0 0 0 0 0 0 0 0 0 0 0 0 0 0 0 0 0 0 0 0 0 0 0 0 0 0 0 0 0 0 0 0 0 0 0 0 0 0 0 0 0 0 0 0 0 0

K03793 0 0 0 0 0 0 0 0 0 0 0 0 0 0 0 0 0 0 0 0 0 0 0 0 0 0 0 0 0 0 0 0 0 0 0 0 0 0 0 0 0 0 0 0 0 0 0 0

K03794 0 0 0 0 0 0 0 0 0 0 0 0 0 0 0 0 0 0 0 0 0 0 0 0 0 0 0 0 0 0 0 0 0 0 0 0 0 0 0 0 0 0 0 0 0 0 0 0

K03795 66 40 107 47 282 216 72 348 84 124 195 86 28 72 124 27 101 222 59 62 106 150 124 208 12 30 21 9 38 292 0 80 50 88 83 77 37 54 90 122 168 154 28 103 96 144 59 230

K03796 0 0 0 0 0 0 0 0 0 0 0 0 0 0 0 0 0 0 0 0 0 0 0 0 0 0 0 0 0 0 0 0 0 0 0 0 0 0 0 0 0 0 0 0 0 0 0 0

K03797 338 420 344 166 406 320 267 564 552 574 661 728 268 601 372 202 508 856 526 476 648 784 838 582 354 338 346 218 292 502 24 648 436 638 772 654 212 438 429 340 452 498 327 580 572 350 470 533

K03798 338 420 344 166 406 320 267 564 552 574 661 728 268 601 372 202 508 856 526 476 648 784 838 582 354 338 346 218 292 502 24 648 436 638 772 654 212 438 429 340 452 498 327 580 572 350 470 533

K03799 512 746 679 278 1031 843 424 1259 811 844 1180 1008 396 961 764 327 931 1424 760 870 1128 1144 1374 1119 484 476 480 318 475 1271 24 1084 671 912 1136 984 316 789 940 684 967 1032 538 1194 1186 662 804 1192

K03800 42 200 159 49 158 147 64 144 106 120 225 154 91 132 160 46 234 210 164 205 234 156 234 208 84 102 94 19 116 243 0 164 68 67 182 137 58 236 295 157 201 244 80 337 382 125 184 244

K03801 0 0 0 0 0 0 0 0 0 0 0 0 0 0 0 0 0 0 0 0 0 0 0 0 0 0 0 0 0 0 0 0 0 0 0 0 0 0 0 0 0 0 0 0 0 0 0 0

K03802 0 0 0 0 0 0 0 0 0 0 0 0 0 0 0 0 0 0 0 0 0 0 0 0 0 0 0 0 0 0 0 0 0 0 0 0 0 0 0 0 0 0 0 0 0 0 0 0

K03803 0 0 0 0 0 0 0 0 0 0 0 0 0 0 0 0 0 0 0 0 0 0 0 0 0 0 0 0 0 0 0 0 0 0 0 0 0 0 0 0 0 0 0 0 0 0 0 0

K03804 0 0 0 0 0 0 0 0 0 0 0 0 0 0 0 0 0 0 0 0 0 0 0 0 0 0 0 0 0 0 0 0 0 0 0 0 0 0 0 0 0 0 0 0 0 0 0 0

K03805 0 0 0 0 0 0 0 0 0 0 0 0 0 0 0 0 0 0 0 0 0 0 0 0 0 0 0 0 0 0 0 0 0 0 0 0 0 0 0 0 0 0 0 0 0 0 0 0

K03806 0 0 0 0 0 0 0 0 0 0 0 0 0 0 0 0 0 0 0 0 0 0 0 0 0 0 0 0 0 0 0 0 0 0 0 0 0 0 0 0 0 0 0 0 0 0 0 0

K03807 0 0 0 0 0 0 0 0 0 0 0 0 0 0 0 0 0 0 0 0 0 0 0 0 0 0 0 0 0 0 0 0 0 0 0 0 0 0 0 0 0 0 0 0 0 0 0 0

K03808 0 0 0 0 0 0 0 0 0 0 0 0 0 0 0 0 0 0 0 0 0 0 0 0 0 0 0 0 0 0 0 0 0 0 0 0 0 0 0 0 0 0 0 0 0 0 0 0

K03809 0 0 0 0 0 0 0 0 0 0 0 0 0 0 0 0 0 0 0 0 0 0 0 0 0 0 0 0 0 0 0 0 0 0 0 0 0 0 0 0 0 0 0 0 0 0 0 0

K03810 0 0 0 0 0 0 0 0 0 0 0 0 0 0 0 0 0 0 0 0 0 0 0 0 0 0 0 0 0 0 0 0 0 0 0 0 0 0 0 0 0 0 0 0 0 0 0 0

K03811 0 0 0 0 0 0 0 0 0 0 0 0 0 0 0 0 0 0 0 0 0 0 0 0 0 0 0 0 0 0 0 0 0 0 0 0 0 0 0 0 0 0 0 0 0 0 0 0

K03812 0 0 0 0 0 0 0 0 0 0 0 0 0 0 0 0 0 0 0 0 0 0 0 0 0 0 0 0 0 0 0 0 0 0 0 0 0 0 0 0 0 0 0 0 0 0 0 0

K03813 0 0 0 0 0 0 0 0 0 0 0 0 0 0 0 0 0 0 0 0 0 0 0 0 0 0 0 0 0 0 0 0 0 0 0 0 0 0 0 0 0 0 0 0 0 0 0 0

K03814 0 0 0 0 0 0 0 0 0 0 0 0 0 0 0 0 0 0 0 0 0 0 0 0 0 0 0 0 0 0 0 0 0 0 0 0 0 0 0 0 0 0 0 0 0 0 0 0

K03815 0 0 0 0 0 0 0 0 0 0 0 0 0 0 0 0 0 0 0 0 0 0 0 0 0 0 0 0 0 0 0 0 0 0 0 0 0 0 0 0 0 0 0 0 0 0 0 0

K03816 0 0 0 0 0 0 0 0 0 0 0 0 0 0 0 0 0 0 0 0 0 0 0 0 0 0 0 0 0 0 0 0 0 0 0 0 0 0 0 0 0 0 0 0 0 0 0 0

K03817 0 0 0 0 0 0 0 0 0 0 0 0 0 0 0 0 0 0 0 0 0 0 0 0 0 0 0 0 0 0 0 0 0 0 0 0 0 0 0 0 0 0 0 0 0 0 0 0

K03818 0 0 0 0 0 0 0 0 0 0 0 0 0 0 0 0 0 0 0 0 0 0 0 0 0 0 0 0 0 0 0 0 0 0 0 0 0 0 0 0 0 0 0 0 0 0 0 0

K03819 0 0 0 0 0 0 0 0 0 0 0 0 0 0 0 0 0 0 0 0 0 0 0 0 0 0 0 0 0 0 0 0 0 0 0 0 0 0 0 0 0 0 0 0 0 0 0 0

K03820 338 420 344 166 406 320 267 564 552 574 661 728 268 601 372 202 508 856 526 476 648 784 838 582 354 338 346 218 292 502 24 648 436 638 772 654 212 438 429 340 452 498 327 580 572 350 470 533

K03821 0 0 0 0 0 0 0 0 0 0 0 0 0 0 0 0 0 0 0 0 0 0 0 0 0 0 0 0 0 0 0 0 0 0 0 0 0 0 0 0 0 0 0 0 0 0 0 0

K03822 0 0 0 0 0 0 0 0 0 0 0 0 0 0 0 0 0 0 0 0 0 0 0 0 0 0 0 0 0 0 0 0 0 0 0 0 0 0 0 0 0 0 0 0 0 0 0 0

K03823 0 0 0 0 1 0 0 0 0 0 3 0 0 0 0 0 0 0 0 0 0 0 0 17 0 0 0 0 0 4 0 30 0 0 0 0 0 0 0 0 0 0 0 0 0 0 0 0

K03824 0 0 0 0 0 0 0 0 0 0 0 0 0 0 0 0 0 0 0 0 0 0 0 0 0 0 0 0 0 0 0 4 0 0 0 0 0 0 0 0 0 0 0 0 0 0 0 0

K03825 0 0 0 0 0 0 0 0 0 0 0 0 0 0 0 0 0 0 0 0 0 0 0 0 0 0 0 0 0 0 0 0 0 0 0 0 0 0 0 0 0 0 0 0 0 0 0 0

K03826 0 0 0 0 0 0 0 0 0 0 0 0 0 0 0 0 0 0 0 0 0 0 0 0 0 0 0 0 0 0 0 0 0 0 0 0 0 0 0 0 0 0 0 0 0 0 0 0

K03827 0 0 0 0 0 0 0 0 0 0 0 0 0 0 0 0 0 0 0 0 0 0 0 0 0 0 0 0 0 0 0 0 0 0 0 0 0 0 0 0 0 0 0 0 0 0 0 0

K03828 0 0 0 0 0 0 0 0 0 0 0 0 0 0 0 0 0 0 0 0 0 0 0 0 0 0 0 0 0 0 0 0 0 0 0 0 0 0 0 0 0 0 0 0 0 0 0 0

K03829 0 0 0 0 0 0 0 0 0 0 0 0 0 0 0 0 0 0 0 0 0 0 0 0 0 0 0 0 0 0 0 0 0 0 0 0 0 0 0 0 0 0 0 0 0 0 0 0

K03830 0 0 0 0 0 0 0 0 0 0 0 0 0 0 0 0 0 0 0 0 0 0 0 0 0 0 0 0 0 0 0 0 0 0 0 0 0 0 0 0 0 0 0 0 0 0 0 0

K03831 0 0 0 0 0 0 0 0 0 0 0 0 0 0 0 0 0 0 0 0 0 0 0 0 0 0 0 0 0 0 0 0 0 0 0 0 0 0 0 0 0 0 0 0 0 0 0 0

K03832 338 420 344 166 406 320 267 564 552 574 661 728 268 601 372 202 508 856 526 476 648 784 838 582 354 338 346 218 292 502 24 648 436 638 772 654 212 438 429 340 452 498 327 580 572 350 470 533

K03833 0 0 0 0 0 0 0 0 0 0 0 0 0 0 0 0 0 0 0 0 0 0 0 0 0 0 0 0 0 0 0 0 0 0 0 0 0 0 0 0 0 0 0 0 0 0 0 0

K03834 0 0 0 0 0 0 0 0 0 0 0 0 0 0 0 0 0 0 0 0 0 0 0 0 0 0 0 0 0 0 0 0 0 0 0 0 0 0 0 0 0 0 0 0 0 0 0 0

K03835 0 0 0 0 0 0 0 0 0 0 0 0 0 0 0 0 0 0 0 0 0 0 0 0 0 0 0 0 0 0 0 0 0 0 0 0 0 0 0 0 0 0 0 0 0 0 0 0

K03836 0 0 0 0 0 0 0 0 0 0 0 0 0 0 0 0 0 0 0 0 0 0 0 0 0 0 0 0 0 0 0 0 0 0 0 0 0 0 0 0 0 0 0 0 0 0 0 0

K03837 0 0 0 0 0 0 0 0 0 0 0 0 0 0 0 0 0 0 0 0 0 0 0 0 0 0 0 0 0 0 0 0 0 0 0 0 0 0 0 0 0 0 0 0 0 0 0 0

K03838 0 0 0 0 0 0 0 0 0 0 0 0 0 0 0 0 0 0 0 0 0 0 0 0 0 0 0 0 0 0 0 0 0 0 0 0 0 0 0 0 0 0 0 0 0 0 0 0

K03839 0 0 0 0 1 0 0 0 0 0 0 0 0 0 0 0 0 0 0 0 0 0 0 0 0 0 0 0 0 0 0 4 0 0 0 0 0 0 0 0 0 0 0 0 0 0 0 0

K03840 0 0 0 0 0 0 0 0 0 0 0 0 0 0 0 0 0 0 0 0 0 0 0 0 0 0 0 0 0 0 0 0 0 0 0 0 0 0 0 0 0 0 0 0 0 0 0 0

K03841 0 0 0 0 1 0 0 0 0 0 3 0 0 0 0 0 0 0 0 0 0 0 0 17 0 0 0 0 0 4 0 30 0 0 0 0 0 0 0 0 0 0 0 0 0 0 0 0

K03850 0 0 0 0 0 0 0 0 0 0 0 0 0 0 0 0 0 0 0 0 0 0 0 0 0 0 0 0 0 0 0 0 0 0 0 0 0 0 0 0 0 0 0 0 0 0 0 0

K03851 0 0 0 0 0 0 0 0 0 0 0 0 0 0 0 0 0 0 0 0 0 0 0 0 0 0 0 0 0 0 0 0 0 0 0 0 0 0 0 0 0 0 0 0 0 0 0 0

K03852 0 0 0 0 0 0 0 0 0 0 0 0 0 0 0 0 0 0 0 0 0 0 0 0 0 0 0 0 0 0 0 0 0 0 0 0 0 0 0 0 0 0 0 0 0 0 0 0

K03855 0 0 0 0 0 0 0 0 0 0 0 0 0 0 0 0 0 0 0 0 0 0 0 0 0 0 0 0 0 0 0 0 0 0 0 0 0 0 0 0 0 0 0 0 0 0 0 0

K03856 0 0 0 0 0 0 0 0 0 0 0 0 0 0 0 0 0 0 0 0 0 0 0 0 0 0 0 0 0 0 0 0 0 0 0 0 0 0 0 0 0 0 0 0 0 0 0 0

K03862 0 0 0 0 0 0 0 0 0 0 0 0 0 0 0 0 0 0 0 0 0 0 0 0 0 0 0 0 0 0 0 0 0 0 0 0 0 0 0 0 0 0 0 0 0 0 0 0

K03863 0 0 0 0 0 0 0 0 0 0 0 0 0 0 0 0 0 0 0 0 0 0 0 0 0 0 0 0 0 0 0 0 0 0 0 0 0 0 0 0 0 0 0 0 0 0 0 0

K03867 0 0 0 0 0 0 0 0 0 0 0 0 0 0 0 0 0 0 0 0 0 0 0 0 0 0 0 0 0 0 0 0 0 0 0 0 0 0 0 0 0 0 0 0 0 0 0 0

K03881 0 0 0 0 0 0 0 0 0 0 0 0 0 0 0 0 0 0 0 0 0 0 0 0 0 0 0 0 0 0 0 0 0 0 0 0 0 0 0 0 0 0 0 0 0 0 0 0

K03884 0 0 0 0 0 0 0 0 0 0 0 0 0 0 0 0 0 0 0 0 0 0 0 0 0 0 0 0 0 0 0 0 0 0 0 0 0 0 0 0 0 0 0 0 0 0 0 0

K03885 478 724 645 262 954 782 404 1210 768 818 1139 968 386 926 719 320 919 1368 748 822 1088 1127 1335 1040 473 471 472 308 458 1188 24 1008 648 866 1094 934 308 759 916 668 952 984 510 1170 1161 658 788 1134

K03886 0 0 0 0 0 0 0 0 0 0 0 0 0 0 0 0 0 0 0 0 0 0 0 0 0 0 0 0 0 0 0 0 0 0 0 0 0 0 0 0 0 0 0 0 0 0 0 0

K03887 0 0 0 0 0 0 0 0 0 0 0 0 0 0 0 0 0 0 0 0 0 0 0 0 0 0 0 0 0 0 0 0 0 0 0 0 0 0 0 0 0 0 0 0 0 0 0 0

K03888 0 0 0 0 0 0 0 0 0 0 0 0 0 0 0 0 0 0 0 0 0 0 0 0 0 0 0 0 0 0 0 0 0 0 0 0 0 0 0 0 0 0 0 0 0 0 0 0

K03889 0 0 0 0 0 0 0 0 0 0 0 0 0 0 0 0 0 0 0 0 0 0 0 0 0 0 0 0 0 0 0 0 0 0 0 0 0 0 0 0 0 0 0 0 0 0 0 0

K03890 0 0 0 0 0 0 0 0 0 0 0 0 0 0 0 0 0 0 0 0 0 0 0 0 0 0 0 0 0 0 0 0 0 0 0 0 0 0 0 0 0 0 0 0 0 0 0 0

K03891 0 0 0 0 0 0 0 0 0 0 0 0 0 0 0 0 0 0 0 0 0 0 0 0 0 0 0 0 0 0 0 0 0 0 0 0 0 0 0 0 0 0 0 0 0 0 0 0

K03892 479 728 606 246 849 705 373 970 770 746 1029 964 378 924 686 306 842 1270 712 856 1062 1012 1290 1012 482 450 466 318 454 1080 24 1106 643 869 1096 956 288 764 874 578 814 928 538 1116 1114 522 761 1019

K03893 0 0 0 0 0 0 0 0 0 0 0 0 0 0 0 0 0 0 0 0 0 0 0 0 0 0 0 0 0 0 0 0 0 0 0 0 0 0 0 0 0 0 0 0 0 0 0 0

K03894 0 0 0 0 0 0 0 0 0 0 0 0 0 0 0 0 0 0 0 0 0 0 0 0 0 0 0 0 0 0 0 0 0 0 0 0 0 0 0 0 0 0 0 0 0 0 0 0

K03895 0 0 0 0 0 0 0 0 0 0 0 0 0 0 0 0 0 0 0 0 0 0 0 0 0 0 0 0 0 0 0 0 0 0 0 0 0 0 0 0 0 0 0 0 0 0 0 0

K03896 0 0 0 0 0 0 0 0 0 0 0 0 0 0 0 0 0 0 0 0 0 0 0 0 0 0 0 0 0 0 0 0 0 0 0 0 0 0 0 0 0 0 0 0 0 0 0 0

K03897 0 0 0 0 0 0 0 0 0 0 0 0 0 0 0 0 0 0 0 0 0 0 0 0 0 0 0 0 0 0 0 0 0 0 0 0 0 0 0 0 0 0 0 0 0 0 0 0

K03918 42 200 159 49 158 147 64 144 106 120 225 154 91 132 160 46 234 210 164 205 234 156 234 208 84 102 94 19 116 243 0 164 68 67 182 137 58 236 295 157 201 244 80 337 382 125 184 244

K03919 0 0 0 0 0 0 0 0 0 0 0 0 0 0 0 0 0 0 0 0 0 0 0 0 0 0 0 0 0 0 0 0 0 0 0 0 0 0 0 0 0 0 0 0 0 0 0 0

K03921 0 0 0 0 0 0 0 0 0 0 0 0 0 0 0 0 0 0 0 0 0 0 0 0 0 0 0 0 0 0 0 0 0 0 0 0 0 0 0 0 0 0 0 0 0 0 0 0

K03922 0 0 0 0 0 0 0 0 0 0 0 0 0 0 0 0 0 0 0 0 0 0 0 0 0 0 0 0 0 0 0 0 0 0 0 0 0 0 0 0 0 0 0 0 0 0 0 0

K03923 0 0 0 0 0 0 0 0 0 0 0 0 0 0 0 0 0 0 0 0 0 0 0 0 0 0 0 0 0 0 0 0 0 0 0 0 0 0 0 0 0 0 0 0 0 0 0 0

K03924 412 684 538 216 742 614 331 890 684 694 952 882 358 854 596 294 818 1188 690 760 982 976 1212 901 462 441 451 300 420 950 24 1022 598 778 1011 858 270 704 826 546 784 830 482 1067 1065 514 729 904

K03925 0 0 0 0 0 0 0 0 0 0 0 0 0 0 0 0 0 0 0 0 0 0 0 0 0 0 0 0 0 0 0 0 0 0 0 0 0 0 0 0 0 0 0 0 0 0 0 0

K03926 384 484 379 166 567 451 276 736 578 574 725 728 268 722 452 248 584 965 526 574 748 820 978 683 377 338 358 280 304 689 24 824 530 736 829 720 212 469 531 389 582 595 402 730 684 390 545 660

K03927 0 0 0 0 1 0 0 0 0 0 0 0 0 0 0 0 0 0 0 0 0 0 0 0 0 0 0 0 0 0 0 0 0 0 0 0 0 0 0 0 0 0 0 0 0 0 0 0

K03928 0 0 0 0 0 0 0 0 0 0 0 0 0 0 0 0 0 0 0 0 0 0 0 0 0 0 0 0 0 0 0 0 0 0 0 0 0 0 0 0 0 0 0 0 0 0 0 0

K03930 0 0 0 0 0 0 0 0 0 0 0 0 0 0 0 0 0 0 0 0 0 0 0 0 0 0 0 0 0 0 0 0 0 0 0 0 0 0 0 0 0 0 0 0 0 0 0 0

K03931 0 0 0 0 0 0 0 0 0 0 0 0 0 0 0 0 0 0 0 0 0 0 0 0 0 0 0 0 0 0 0 0 0 0 0 0 0 0 0 0 0 0 0 0 0 0 0 0

K03932 0 0 0 0 0 0 0 0 0 0 0 0 0 0 0 0 0 0 0 0 0 0 0 0 0 0 0 0 0 0 0 0 0 0 0 0 0 0 0 0 0 0 0 0 0 0 0 0

K03933 0 0 0 0 0 0 0 0 0 0 0 0 0 0 0 0 0 0 0 0 0 0 0 0 0 0 0 0 0 0 0 0 0 0 0 0 0 0 0 0 0 0 0 0 0 0 0 0

K03935 0 0 0 0 0 0 0 0 0 0 0 0 0 0 0 0 0 0 0 0 0 0 0 0 0 0 0 0 0 0 0 0 0 0 0 0 0 0 0 0 0 0 0 0 0 0 0 0

K03936 0 0 0 0 0 0 0 0 0 0 0 0 0 0 0 0 0 0 0 0 0 0 0 0 0 0 0 0 0 0 0 0 0 0 0 0 0 0 0 0 0 0 0 0 0 0 0 0

K03940 0 0 0 0 0 0 0 0 0 0 0 0 0 0 0 0 0 0 0 0 0 0 0 0 0 0 0 0 0 0 0 0 0 0 0 0 0 0 0 0 0 0 0 0 0 0 0 0

K03941 0 0 0 0 0 0 0 0 0 0 0 0 0 0 0 0 0 0 0 0 0 0 0 0 0 0 0 0 0 0 0 0 0 0 0 0 0 0 0 0 0 0 0 0 0 0 0 0

K03943 0 0 0 0 0 0 0 0 0 0 0 0 0 0 0 0 0 0 0 0 0 0 0 0 0 0 0 0 0 0 0 0 0 0 0 0 0 0 0 0 0 0 0 0 0 0 0 0

K03955 0 0 0 0 0 0 0 0 0 0 0 0 0 0 0 0 0 0 0 0 0 0 0 0 0 0 0 0 0 0 0 0 0 0 0 0 0 0 0 0 0 0 0 0 0 0 0 0

K03969 0 0 0 0 0 0 0 0 0 0 0 0 0 0 0 0 0 0 0 0 0 0 0 0 0 0 0 0 0 0 0 0 0 0 0 0 0 0 0 0 0 0 0 0 0 0 0 0

K03970 0 0 0 0 0 0 0 0 0 0 0 0 0 0 0 0 0 0 0 0 0 0 0 0 0 0 0 0 0 0 0 0 0 0 0 0 0 0 0 0 0 0 0 0 0 0 0 0

K03971 0 0 0 0 0 0 0 0 0 0 0 0 0 0 0 0 0 0 0 0 0 0 0 0 0 0 0 0 0 0 0 0 0 0 0 0 0 0 0 0 0 0 0 0 0 0 0 0

K03972 0 0 0 0 0 0 0 0 0 0 0 0 0 0 0 0 0 0 0 0 0 0 0 0 0 0 0 0 0 0 0 0 0 0 0 0 0 0 0 0 0 0 0 0 0 0 0 0

K03973 0 0 0 0 23 16 0 9 0 0 3 0 0 0 0 0 0 14 0 0 0 0 0 23 0 0 0 0 0 18 0 30 0 0 0 0 0 0 0 0 0 0 0 0 0 0 0 0

K03974 0 0 0 0 0 0 0 0 0 0 0 0 0 0 0 0 0 0 0 0 0 0 0 0 0 0 0 0 0 0 0 0 0 0 0 0 0 0 0 0 0 0 0 0 0 0 0 0

K03975 0 0 0 0 2 0 0 0 0 0 3 0 0 0 0 0 0 0 0 0 0 0 0 0 0 0 0 0 0 0 0 0 0 0 0 0 0 0 0 0 0 0 0 0 0 0 0 0

K03976 0 0 0 0 0 0 0 0 0 0 0 0 0 0 0 0 0 0 0 0 0 0 0 0 0 0 0 0 0 0 0 0 0 0 0 0 0 0 0 0 0 0 0 0 0 0 0 0

K03977 338 420 344 166 406 320 267 564 552 574 661 728 268 601 372 202 508 856 526 476 648 784 838 582 354 338 346 218 292 502 24 648 436 638 772 654 212 438 429 340 452 498 327 580 572 350 470 533

K03978 0 0 0 0 0 0 0 0 0 0 0 0 0 0 0 0 0 0 0 0 0 0 0 0 0 0 0 0 0 0 0 0 0 0 0 0 0 0 0 0 0 0 0 0 0 0 0 0

K03979 338 420 344 166 406 320 267 564 552 574 661 728 268 601 372 202 508 856 526 476 648 784 838 582 354 338 346 218 292 502 24 648 436 638 772 654 212 438 429 340 452 498 327 580 572 350 470 533

K03980 338 420 344 166 406 320 267 564 552 574 661 728 268 601 372 202 508 856 526 476 648 784 838 582 354 338 346 218 292 502 24 648 436 638 772 654 212 438 429 340 452 498 327 580 572 350 470 533

K03981 0 0 0 0 0 0 0 0 0 0 0 0 0 0 0 0 0 0 0 0 0 0 0 0 0 0 0 0 0 0 0 0 0 0 0 0 0 0 0 0 0 0 0 0 0 0 0 0

K04013 0 0 0 0 0 0 0 0 0 0 0 0 0 0 0 0 0 0 0 0 0 0 0 0 0 0 0 0 0 0 0 0 0 0 0 0 0 0 0 0 0 0 0 0 0 0 0 0

K04014 0 0 0 0 0 0 0 0 0 0 0 0 0 0 0 0 0 0 0 0 0 0 0 0 0 0 0 0 0 0 0 0 0 0 0 0 0 0 0 0 0 0 0 0 0 0 0 0

K04015 0 0 0 0 0 0 0 0 0 0 0 0 0 0 0 0 0 0 0 0 0 0 0 0 0 0 0 0 0 0 0 0 0 0 0 0 0 0 0 0 0 0 0 0 0 0 0 0

K04016 0 0 0 0 0 0 0 0 0 0 0 0 0 0 0 0 0 0 0 0 0 0 0 0 0 0 0 0 0 0 0 0 0 0 0 0 0 0 0 0 0 0 0 0 0 0 0 0

K04017 0 0 0 0 0 0 0 0 0 0 0 0 0 0 0 0 0 0 0 0 0 0 0 0 0 0 0 0 0 0 0 0 0 0 0 0 0 0 0 0 0 0 0 0 0 0 0 0

K04018 0 0 0 0 0 0 0 0 0 0 0 0 0 0 0 0 0 0 0 0 0 0 0 0 0 0 0 0 0 0 0 0 0 0 0 0 0 0 0 0 0 0 0 0 0 0 0 0

K04019 0 0 0 0 0 0 0 0 0 0 0 0 0 0 0 0 0 0 0 0 0 0 0 0 0 0 0 0 0 0 0 0 0 0 0 0 0 0 0 0 0 0 0 0 0 0 0 0

K04020 0 0 0 0 0 0 0 0 0 0 0 0 0 0 0 0 0 0 0 0 0 0 0 0 0 0 0 0 0 0 0 0 0 0 0 0 0 0 0 0 0 0 0 0 0 0 0 0

K04021 0 0 0 0 0 0 0 0 0 0 0 0 0 0 0 0 0 0 0 0 0 0 0 0 0 0 0 0 0 0 0 0 0 0 0 0 0 0 0 0 0 0 0 0 0 0 0 0

K04022 0 0 0 0 0 0 0 0 0 0 0 0 0 0 0 0 0 0 0 0 0 0 0 0 0 0 0 0 0 0 0 0 0 0 0 0 0 0 0 0 0 0 0 0 0 0 0 0

K04023 0 0 0 0 0 0 0 0 0 0 0 0 0 0 0 0 0 0 0 0 0 0 0 0 0 0 0 0 0 0 0 0 0 0 0 0 0 0 0 0 0 0 0 0 0 0 0 0

K04024 0 0 0 0 0 0 0 0 0 0 0 0 0 0 0 0 0 0 0 0 0 0 0 0 0 0 0 0 0 0 0 0 0 0 0 0 0 0 0 0 0 0 0 0 0 0 0 0

K04025 0 0 0 0 0 0 0 0 0 0 0 0 0 0 0 0 0 0 0 0 0 0 0 0 0 0 0 0 0 0 0 0 0 0 0 0 0 0 0 0 0 0 0 0 0 0 0 0

K04026 0 0 0 0 0 0 0 0 0 0 0 0 0 0 0 0 0 0 0 0 0 0 0 0 0 0 0 0 0 0 0 0 0 0 0 0 0 0 0 0 0 0 0 0 0 0 0 0

K04027 0 0 0 0 0 0 0 0 0 0 0 0 0 0 0 0 0 0 0 0 0 0 0 0 0 0 0 0 0 0 0 0 0 0 0 0 0 0 0 0 0 0 0 0 0 0 0 0

K04028 0 0 0 0 0 0 0 0 0 0 0 0 0 0 0 0 0 0 0 0 0 0 0 0 0 0 0 0 0 0 0 0 0 0 0 0 0 0 0 0 0 0 0 0 0 0 0 0

K04029 0 0 0 0 0 0 0 0 0 0 0 0 0 0 0 0 0 0 0 0 0 0 0 0 0 0 0 0 0 0 0 0 0 0 0 0 0 0 0 0 0 0 0 0 0 0 0 0

K04030 0 0 0 0 0 0 0 0 0 0 0 0 0 0 0 0 0 0 0 0 0 0 0 0 0 0 0 0 0 0 0 0 0 0 0 0 0 0 0 0 0 0 0 0 0 0 0 0

K04031 0 0 0 0 0 0 0 0 0 0 0 0 0 0 0 0 0 0 0 0 0 0 0 0 0 0 0 0 0 0 0 0 0 0 0 0 0 0 0 0 0 0 0 0 0 0 0 0

K04032 0 0 0 0 0 0 0 0 0 0 0 0 0 0 0 0 0 0 0 0 0 0 0 0 0 0 0 0 0 0 0 0 0 0 0 0 0 0 0 0 0 0 0 0 0 0 0 0

K04033 0 0 0 0 0 0 0 0 0 0 0 0 0 0 0 0 0 0 0 0 0 0 0 0 0 0 0 0 0 0 0 0 0 0 0 0 0 0 0 0 0 0 0 0 0 0 0 0

K04034 14 0 0 0 6 0 8 0 0 0 0 0 0 0 16 0 0 0 0 18 0 0 0 14 0 0 0 0 0 0 0 0 0 24 0 0 0 0 0 0 0 8 0 0 0 0 0 0

K04035 33 18 73 32 228 170 52 308 40 98 154 45 18 37 78 20 89 181 48 14 66 133 84 136 1 26 13 0 21 222 0 5 28 42 40 28 28 24 66 106 152 105 0 78 72 140 43 173

K04036 0 0 0 0 0 0 0 0 0 0 0 0 0 0 0 0 0 0 0 0 0 0 0 0 0 0 0 0 0 0 0 0 0 0 0 0 0 0 0 0 0 0 0 0 0 0 0 0

K04037 0 0 0 0 0 0 0 0 0 0 0 0 0 0 0 0 0 0 0 0 0 0 0 0 0 0 0 0 0 0 0 0 0 0 0 0 0 0 0 0 0 0 0 0 0 0 0 0

K04038 0 0 0 0 0 0 0 0 0 0 0 0 0 0 0 0 0 0 0 0 0 0 0 0 0 0 0 0 0 0 0 0 0 0 0 0 0 0 0 0 0 0 0 0 0 0 0 0

K04039 0 0 0 0 0 0 0 0 0 0 0 0 0 0 0 0 0 0 0 0 0 0 0 0 0 0 0 0 0 0 0 0 0 0 0 0 0 0 0 0 0 0 0 0 0 0 0 0

K04040 0 0 0 0 0 0 0 0 0 0 0 0 0 0 0 0 0 0 0 0 0 0 0 0 0 0 0 0 0 0 0 0 0 0 0 0 0 0 0 0 0 0 0 0 0 0 0 0

K04041 0 0 0 0 0 0 0 0 0 0 0 0 0 0 0 0 0 0 0 0 0 0 0 0 0 0 0 0 0 0 0 0 0 0 0 0 0 0 0 0 0 0 0 0 0 0 0 0
[truncated: 455,849 more chars]
